# Solvent-controlled self-assembly of tetrapodal [4 + 4] phosphate organic molecular cage

**DOI:** 10.1038/s41598-020-61813-6

**Published:** 2020-03-13

**Authors:** Gen-Feng Feng, Jiao Geng, Fan-Da Feng, Wei Huang

**Affiliations:** 1 State Key Laboratory of Coordination Chemistry, School of Chemistry and Chemical Engineering, Nanjing University, Nanjing, Jiangsu Province 210093 P.R. China; 2 Shenzhen Research Institute of Nanjing University, Shenzhen, Guangdong Province 518057 P.R. China

**Keywords:** Structure elucidation, Self-assembly

## Abstract

Two flexible subcomponents, namely tris(4-formylphenyl)phosphate and tris(2-aminoethyl)amine, are assembled into a tetrapodal [4 + 4] cage depending on the solvent effect. Single-crystal structure analysis reveals that the caivity is surrounded by four phosphate uints. Good selectivity of CO_2_ adsorption over CH_4_ is demonstrated by the gas adsorption experiment.

## Introduction

Discrete molecular architectures, especially those organic cage compounds, have received intensive attention in recent years^1–8^. These intriguing compounds featuring aesthetic geometry and intrinsic cavities display potential applications in gas or organic molecular separation^9–14^, catalysis^15–18^, porous liquids^19,20^ and detection^21–23^. Synthetic control on the formation of versatile cages with given topologies is crucial before exploring their applications. Due to the characteristic of self-healing, dynamic covalent chemistry has been demonstrated as a powerful approach to synthesize these sophisticated cages from simple precursors. Among them, imine condensation, boronicester or boroxine formation and alkyne metathesis are the most frequently used type of dynamic bond formation^24^.

Using reversible covalent chemistry to construct organic molecular cages (OMCs), external stimuli, such as solvent, pH, temperature, catalysts, steric and electronic factors, are all worthy of enough attention^25^. Sometimes, different solvents could govern the self-assembly behavior to form cage products with different geometry. For example, Liu and Warmuth described a solvent-dependent method to selectively synthesize tetrahedral, octahedral and square antiprismatic cages from the same sub-components^26^. On the other hand, choice of appropriate building blocks or precursors in the self-assembly process is believed as a key and important factor to access the desired and functionalized OMCs^27,28^. In the family of tritopic building blocks or precursors, 1,3,5-triformylbenzene and triptycene triamine occupy a special status owing to their excellent ability to form various OMCs with different partners^1,29–31^.

A semi-flexible phosphate based trialdehyde had been proved to be a practicable precursor containing P=O functional site and a functionalized [2 + 3] imine OMC was successfully obtained by our group^32^. As a part of our ongoing research on OMCs, we aim to further investigate and demonstrate that the semi-flexible precursor could also satisfy the requirements of different geometrical organic cage assembly. So we have changed the amine component from previous ditopic linker (cyclohexanediamine) to a flexible tritopic linker [tris(2-aminoethyl)amine]. For multi-component systems comprising competitive reactions, increasing the number of reactive ending groups and the flexibility of the amine linker is bound to complicate the self-assembly process. To address this challenge, a solvent-controlled method was tried and applied to simplify the self-assembly process towards the desired OMC in this paper.

## Results and Discussion

**Figure 1:**
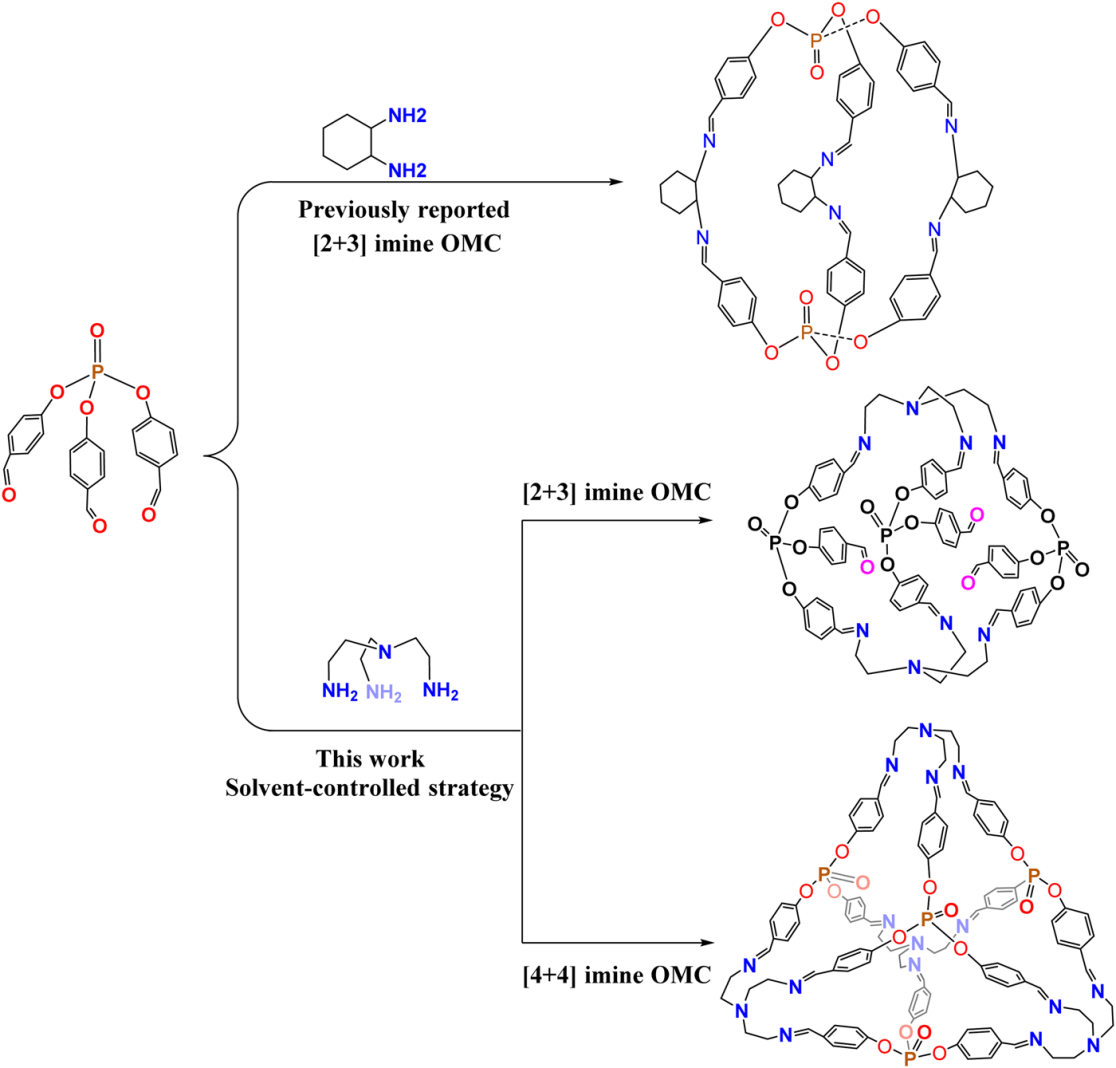
Different OMCs based on the phosphate trialdehyde.

**Figure 2:**
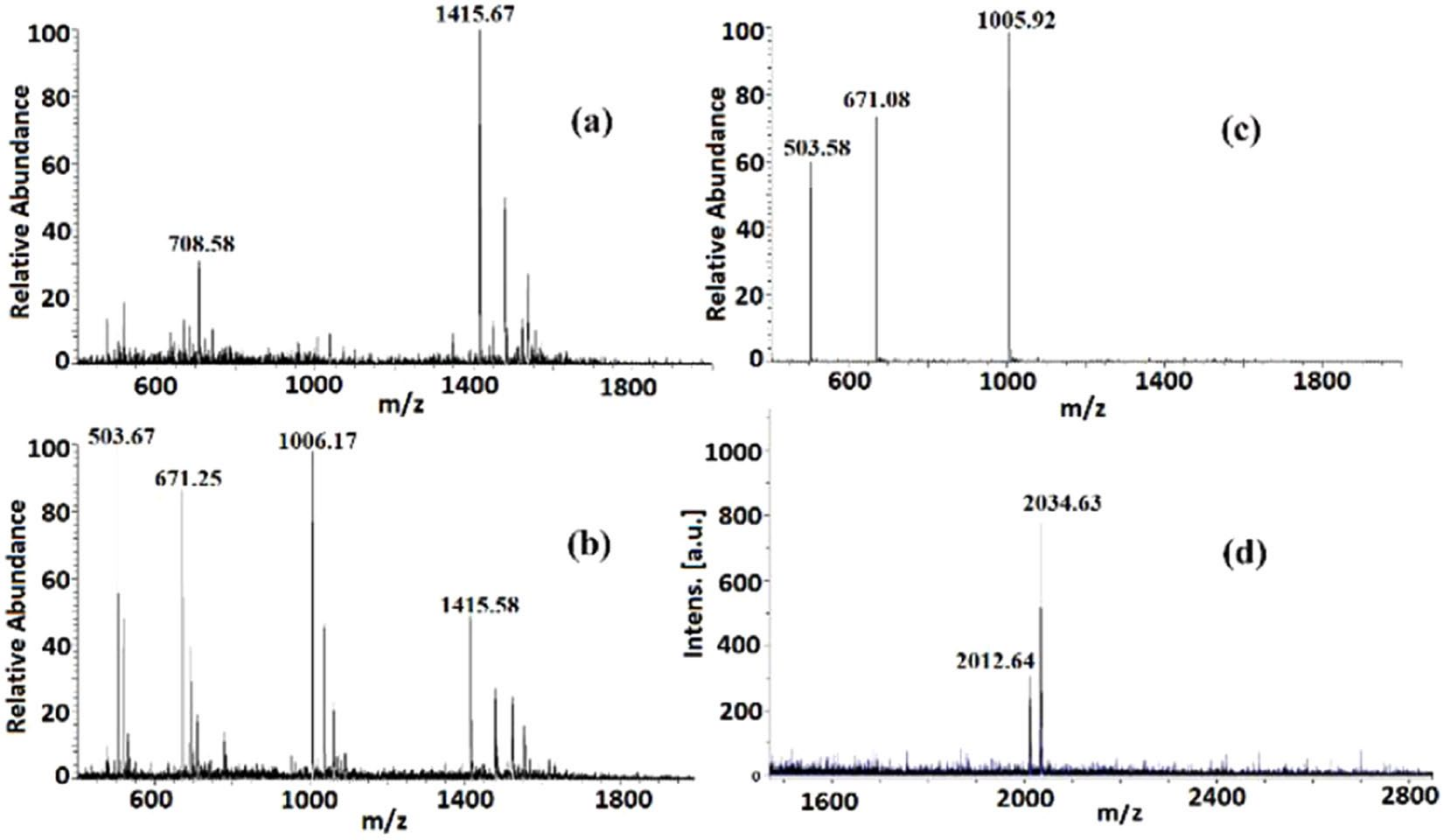
Solvent Effects on the cage assembly. **(a)** EIS-MS in acetonitrile; **(b)** EIS-MS in mixture of acetonitrile and chloroform; **(c)** EIS-MS in chloroform; **(d)** MALDI-TOF-MS in chloroform.

Schiff-base condensation generally has great compatibility with different solvents, and different solvents have large influences on the final molecular crystallization and solid-state crystal packing^33,34^. Acetonitrile/chloroform (v/v = 5:1) was adopted as a mixed-solvent in the synthesis of previous [2 + 3] phosphate cage just because of its suitability to grow single crystals. Actually, both acetonitrile and chloroform were effective solvents to yield the [2 + 3] cage. However, in the following self-assembly reaction between two tritopic precursors [phosphate trialdehyde and tris(2-aminoethyl)amine], it is found that the type of reaction solvents could dramatically impact the self-assembled outcomes (Fig. 1). In our experiments, acetonitrile as a single solvent for equimolar self-assembly is firstly tried since two tritopic precursors with a 1:1 molar ratio could theoretically form a [4 + 4] molecular cage. Nevertheless, the ESI-MS spectrum does not give the expected [4 + 4] assembled results, in which two positive peaks at *m/z* = 1415.67 and 708.58 are present corresponding to the full and half peaks of a [2 + 3] molecular cage with three unreacted aldehyde groups (Fig. 2a). Furthermore, the ^1^H NMR spectrum of this species evidences the existence of the aldehyde group (Fig. S1). The combination of ESI-MS and ^1^H NMR analyses suggests that one aldehyde group from every phosphate trialdehyde precursor is not involved in the cage construction and six-fold imine condensation produces a credible [2 + 3] cage structure. This half-way self-assembly could be ascribed to the poor solubility for the [2 + 3] cage product in acetonitrile, which tends to precipitate from the reaction solution and terminate the further imine condensation.

Considering that chlorohydrocarbon solvents are often used in the cage syntheses^11,16,21–23,35,36^, a mixture of 1:1 acetonitrile and chloroform is then used for this reaction. Although the formation of partial [2 + 3] cage still occurs, the successful assembly of a [4 + 4] molecular age is achieved which can be verified by the presence of new ESI-MS peaks at *m/z* = 1006.17, 671.25 and 503.67 with the isotopic distribution patterns separated by 0.50 ± 0.01, 0.33 ± 0.01 and 0.25 ± 0.01 Da (Figs. 2b and S2), corresponding to 1/2, 1/3 and 1/4 of the molecular weight of the [4 + 4] cage. In order to improve the yield of [4 + 4] cage, pure chloroform is explored, where the ESI-MS spectrum clearly exhibits three positive peaks originating from the [4 + 4] cage excluding the peaks of half-way [2 + 3] cage (Fig. 2c). The formation of [4 + 4] cage was further confirmed by a peak at *m/z* = 2012.64 in the MALDI-TOF-MS spectrum (Fig. 2d). In addition, ^1^H NMR spectrum provides reliable proofs for the formation of this [4 + 4] cage, where only one set of signals could be observed for this symmetrical structure (Fig. S3).

**Figure 3:**
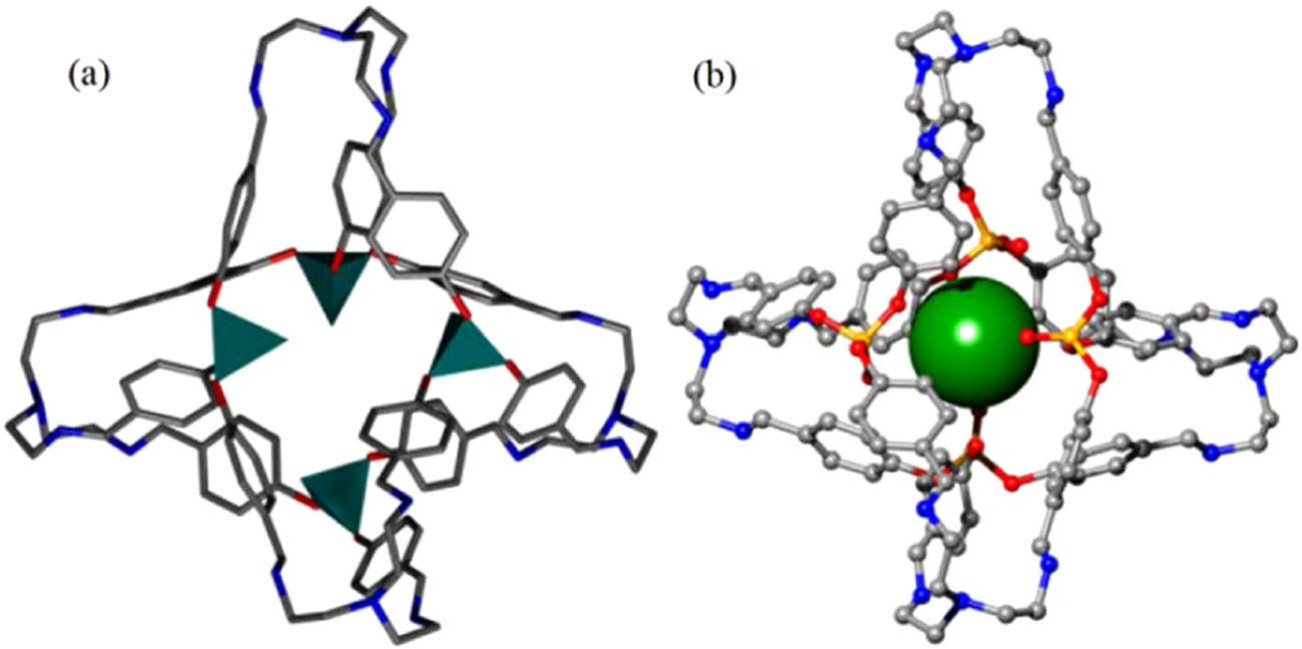
(**a**) Crystal structure of the cage (phosphate units are represented by four tetrahedrons); **(b)** the size of the cage (represented by a green ball in a radius of 2.0 Å).

The molecular structure of [4 + 4] cage was further verified by single-crystal X-ray diffraction studies. Slow evaporation of the reaction solution affords the suitable single crystals for the X-ray diffraction determination. Crystallographic analysis shows that this [4 + 4] cage has a tetrapodal structure with symmetry (Fig. 3). The shape of the cage could also be regarded as tetrahedral shape with the tertiary amine nitrogens of the amine linkers as four vertexes. In both [4 + 4] cage and previously reported [2 + 3] cage, the phosphate tetrahedron comprised by four oxygen atoms is very rigid (Fig. 3a). However, three P-O single bonds of the phosphate tetrahedron could be rotated along the axial direction freely, which would result in the three benzene rings pointing toward different orientations during the construction of OMCs with cyclohexanediamine and tris(2-aminoethyl)amine. Herein we use the distance between the O atom of P=O double bond and the centroid of benzene ring to illustrate the discrepancy between the conformation of the cages. This parameter is the same as 3.85 Å for three benzene rings in the previous [2 + 3] cage exhibiting the typical tripod configuration, while it changes to 3.94, 4.10 and 5.13 Å in the current [4 + 4] cage indicating two different orientations in forming a more complicated cage with flexible tris(2-aminoethyl)amine. Unlike the previous [2 + 3] cage, the P=O bonds does not point straight toward the cage centre in this [4 + 4] cage and the window of the cage cavity was partially occupied by the oxygen atoms of the P=O units (Fig. 3b). In addition, a cavity with a 4.0 Å diameter taking into account the van der Waals radii of the atoms is found in the cage, which is comparable with that of some gas molecules such as CO_2_, CH_4_ and N_2_^37^. In addition, the thermal stability of this [4 + 4] cage is evaluated by thermogravimetric analysis (TGA), in which the decomposition temperature is high up to 300 °C (Fig. S4). This work is believed to give a representative example to illustrate the semi-flexible and versatile traits of this phosphate trialdehyde.

**Figure 4:**
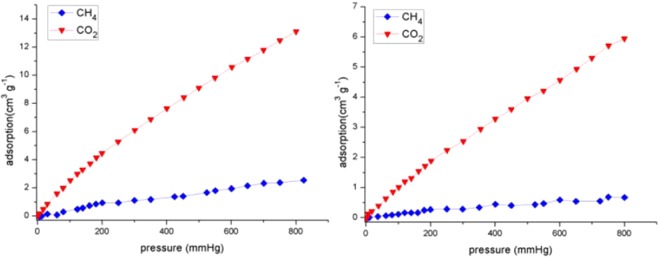
CO_2_ (red) and CH_4_ (blue) adsorption isotherms at 273 (left) and 298 K (right).

Given that gas sorption phenomena have been revealed on discrete organic molecular solids, gas adsorption properties of this [4 + 4] cage were explored. After thermal activation for 12 h at 80 °C under the high vacuum, the crystalline sample has lost its crystallinity/solvents and became amorphous (Figs. S5 and S6). The N_2_ adsorption isotherms of the cage at 77 K indicate a low BET surface area (less than 10 m^2^/g), which is similar to other discrete cage compounds^38–41^. Gas adsorption experiments at 273 and 298 K reveal that the cage has selective adsorption of CO_2_ over CH_4_. As illustrated in Fig. 4, it can absorb 12.46 and 5.71 cm^3^ g^−1^ of CO_2_ at 273 and 298 K and 1.0 bar, whereas small amounts of CH_4_ uptake can be observed at 1 bar (2.36 and 0.68 cm^3^ g^−1^ at 273 and 298 K). The selectivities of CO_2_ over CH_4_ estimated from Henry’s constants are 7.10 and 6.52 at 298 and 273 K (Table S2), respectively, which are comparable with other previously reported OMCs (For CO_2_ vs CH4 adsorption and separation of some selected OMCs, see Table S3). The reason for the high selectivities of CO_2_ over CH_4_ is probably due to the presence of polar functional P=O groups for CO_2_ adsorption^12,42,43^.

## Conclusion

In summary, one novel [4 + 4] phosphate OMC can be efficiently synthesized in a one pot reaction through solvent-controlled multicomponent imine condensation between tris(4-formylphenyl)phosphate and tris(2-aminoethyl)amine. It is concluded that acetonitrile is beneficial to the assembly of [2 + 3] halfway cage, while chloroform can promote the full conversion of the functional groups on two reactants. Structurally, this cage possesses one distinctive cavity constituted by four phosphate units. Furthermore, this [4 + 4] phosphate OMC displays good selectivity of CO_2_ adsorption over CH_4_. Thus, the strategy we offered here to synthesize the [4 + 4] phosphate cage is believed to be instructive for designing new type functionalized organic cages and host molecules in supramolecular chemistry.

## Supplementary information

Supplementary information

Supplementary information2
