# Supplementary material for: Solvent-controlled self-assembly of tetrapodal [4 + 4] phosphate organic molecular cage
Source: Sci Rep. 2020 Mar 13;10:4712. doi: 10.1038/s41598-020-61813-6 (PMC7070053; doi:10.1038/s41598-020-61813-6)
Supplement: Supplementary file 2 — Supplementary information2 [file 41598_2020_61813_MOESM2_ESM.pdf]

Electronic Supplementary Information

Solvent-controlled self-assembly of tetrapodal [4+4] phosphate organic molecular cage

Gen-Feng Feng,[a] Jiao Geng,[a] Fan-Da Feng[a] and Wei Huang[*,a,b]

[a] State Key Laboratory of Coordination Chemistry, School of Chemistry and Chemical Engineering, Nanjing University, Nanjing, Jiangsu Province, 210093, P. R. China

[b] Shenzhen Research Institute of Nanjing University, Shenzhen, Guangdong Province, 518057, P. R. China

*corresponding author: whuang@nju.edu.cn

S1. General

All chemicals were purchased from commercial corporations and used without further purification unless otherwise noted. NMR spectra spectroscopic measurements were performed on Bruker 400 NMR spectrometer with tetramethylsilane (TMS) as the internal standard at room temperature. Mass spectrometric data were determined with a ThermoFisher Scientific LCQ Fleet mass spectrometer (ESI-MS) and a Bruker Autoflex II MALDI-TOF mass spectrometer (MALDI-TOF-MS). Infrared spectra (4000-400 cm^{-1}) were collected on a Nicolet FT-IR 170X spectrophotometer at 25 °C using KBr plates. Thermal analyses were performed on a Universal V3.9A TA Instruments from room temperature to 600 °C with a heating rate of 10 °C/min under flowing nitrogen. In the gas sorption measurement, ultra-high-purity grade CH_4 (> 99.999 %) and CO_2 gases (99.995 % purity) were used throughout the adsorption experiments. Low-pressure CH_4 and CO_2 adsorption measurements (up to 1 bar) were performed on Micromeritics ASAP 2020 M+C surface area analyzer.

S2. Synthesis of [4+4] cage

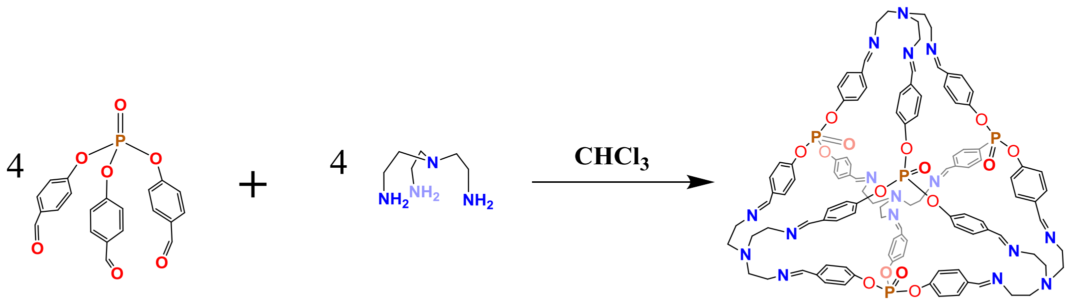

Tris(4-formylphenyl) phosphate was prepared as previously reported.[1] Twelve-fold cycloimination reaction involving tris(4-formylphenyl) phosphate and tris[2-(dimethylamino)ethyl]amine could yield [4+4] cage. Tris(4-formylphenyl) phosphate (50.0 mg, 0.12 mmol) was dissolved in 30 mL chloroform at room temperature, and then tris[2-(dimethylamino)ethyl]amine (17.8 mg, 0.12 mmol) was added. The solution was stirred for 48 h at room temperature. After that, the mixture was filtered to a beaker and *n*-hexane was layered. The cage product (41.0 mg) was obtained as colorless crystals by slow evaporation in air at room temperature with a yield of 68 %.

S3. Solvent control experiment

Acetonitrile, the mixture of acetonitrile and chloroform (v/v = 1:1), and pure chloroform was applied to the self-assembly process using the above mentioned procedure. ESI-MS was used to monitor the cycloimination reaction.

S4. X-Ray data collection and structural determination

A single-crystal sample of [4+4] cage was covered with glue and mounted on glass fibers and then used for data collection. Crystallographic data were collected on Bruker D8 CMOS detectors at 123 K. The structure was solved using direct methods and all refined on F^2 with the SHELX-2014 software package. It is found that the solvent molecules were highly disordered. Attempts to locate and refine the solvent peaks were unsuccessful. So contributions to scattering due to these solvent molecules were removed using the SQUEEZE routine of PLATON. The summary of the crystal data, experimental details and refinement results for the [4+4] cage is listed in Table S1.

Table S1. Crystal data and structure refinement for the [4+4] cage

Empirical formula	$C_{108}H_{108}N_{16}O_{16}P_4$
Formula weight	2009.98
Temperature	123(2) K
Wavelength	0.71073 Å
Crystal system	Tetragonal
Space group	$I\bar{4}$
a	22.556(4) Å
b	22.556(4) Å
c	12.858(2) Å
α	90°
β	90°
γ	90°
Volume	6542(3) Å^3
Z	2
Density (calculated)	1.020 g/cm^3
Absorption coefficient	0.116 mm^{-1}
F(000)	2112
Crystal size	0.12 x 0.10 x 0.10 mm^3

Theta range for data collection	2.554 to 24.993°
Index ranges	-25 ≤ *h* ≤ 26, -26 ≤ *k* ≤25, -15 ≤ *l* ≤13
Reflections collected	20734
Independent reflections	5760 [R_{int} = 0.0816]
Completeness	99.7 %
Refinement method	Full-matrix least-squares on F^2
Data / parameters	5760 / 0 / 326
Goodness-of-fit on F^2	1.004
Final *R* indices [*I*>2sigma(*I*)]	R_1 = 0.0984, wR_2 = 0.2243
R indices (all data)	R_1 = 0.1504, wR_2 = 0.2453
Largest diff. peak and hole (e·Å^{-3})	0.421 / -0.349

Table S2. Gas sorption quantities for CO_2 and CH_4, and CO_2/CH_4 selectivity

cage	T (K)	CO_2 uptake at 1 bar (cm^3 g^{-1})	CH_4 uptake at 1 bar (cm^3 g^{-1})	CO_2/CH_4 selectivity	
				Simple (ideal)	Henry's law
	298	5.71	0.68	8.39	7.10
	273	12.46	2.36	5.27	6.52

Table S3. Summary of CO_2 uptake, CH_4 uptake and ideal selectivity (CO_2/CH_4) in selected porous materials

Compound	CO_2 uptake (mmol g^{-1})	CH_4 uptake (mmol g^{-1})	Ideal selectivity (CO_2/CH_4)	Reference
[4+4]	4.1[a]	1.3 [a]	3.2	2
[4+6]-*t*Bu	2.7[a]	0.7[a]	3.9	3,4
[4+6]-exo	3.4[a]	0.7[a]	4.9	5
[2+3] cage	2.7[b]	0.7[b]	3.9	6
[2+3]-extend	3.3[b]	0.3[b]	11	6
CC1α'	1.27[c]	0.55[c]	2.3	7
CC2	3.0[c]	1.13[c]	2.7	7
CC3	2.47[c]	1.53[c]	1.6	7
CC6	0.9[d]	n.d.		8
ZCs	0.1-0.25[e]	n.d.		9
[4+6]-triazine	38.6[f]	16.4[f]	2.4	10
PB-2			47.9[g]	11
Amide cage (-OMe)	47.8	14.0	20.41	12
Brominated amide cage	45.8	12.6	17.63	12
Nitrated amide cage	46.3	11.2	28.56	12

Amide cage (-OH)	45.8	11.2	14.38	12

[a] Measured at 273 K, 1 bar; [b] at 298 K, 1 bar; [c] at 275 K, 1 bar; [d] at 300 K, 1.2 bar; [e] at 293 K, 1 bar; [f] cm^3g^{-1}, at 273 K, 1.08 bar; [g] IAST selectivity, at 273 K, 1 bar.

S5. Additional Figures

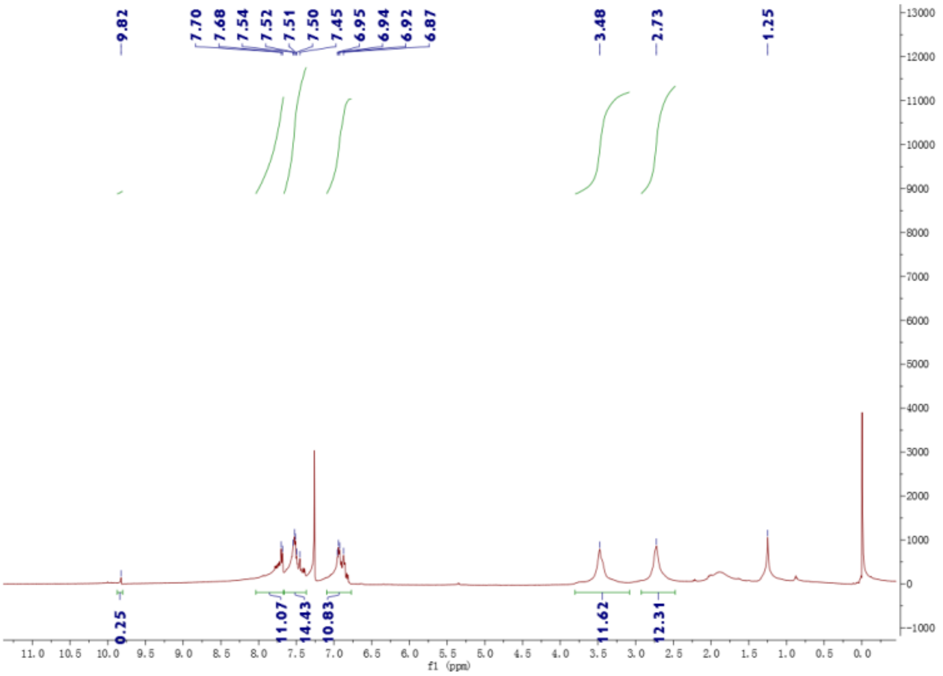

Figure S1. ^{1}H NMR ($CDCl_3$) spectrum of the [2+3] product isolated from acetonitrile.

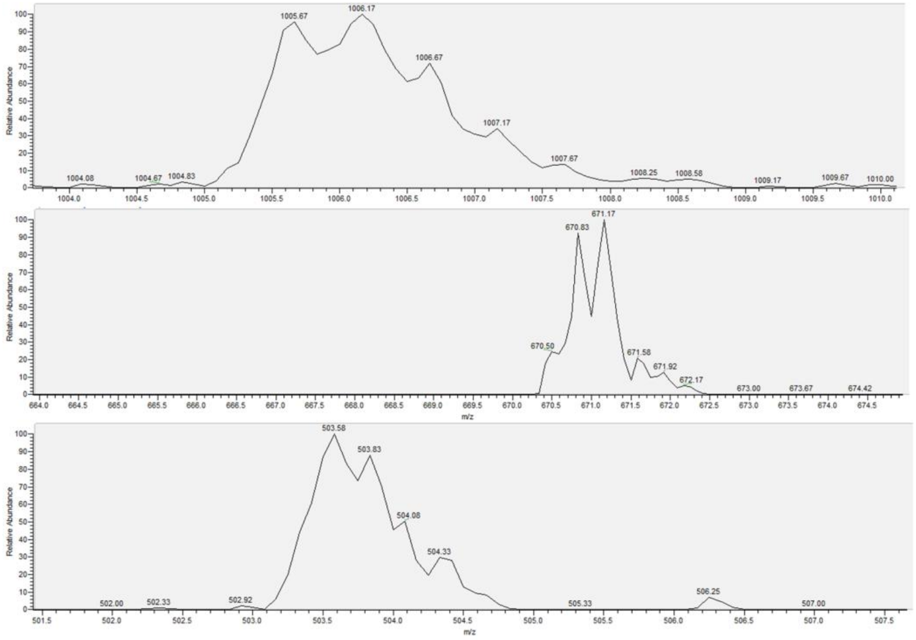

Figure S2. The amplified 1/2, 1/3 and 1/4 peaks of [4+4] cage.

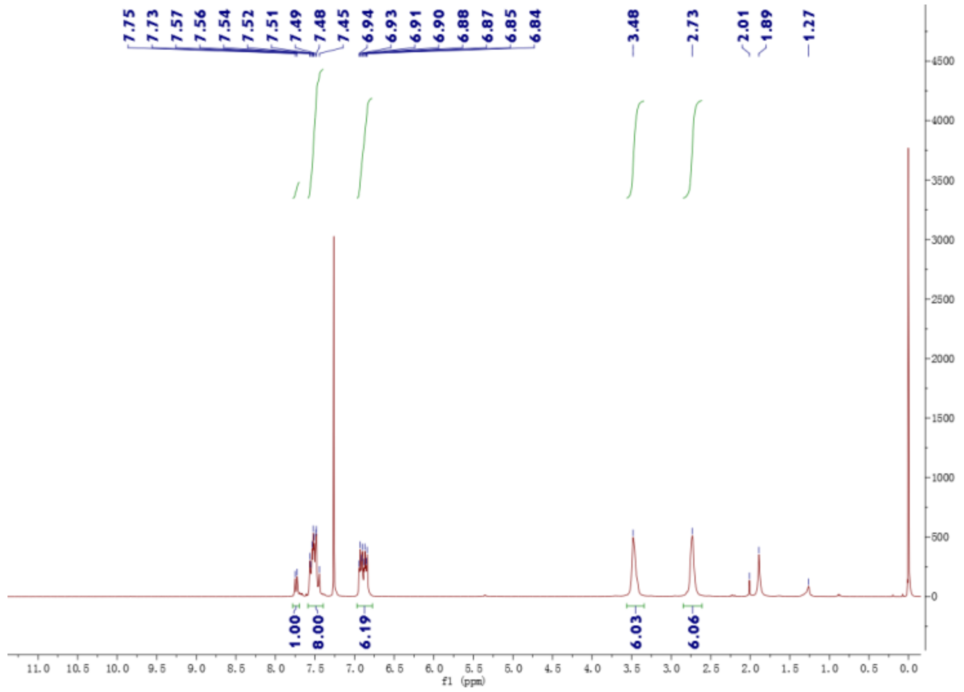

Figure S3. ^{1}H NMR ($CDCl_3$) spectrum of [4+4] cage.

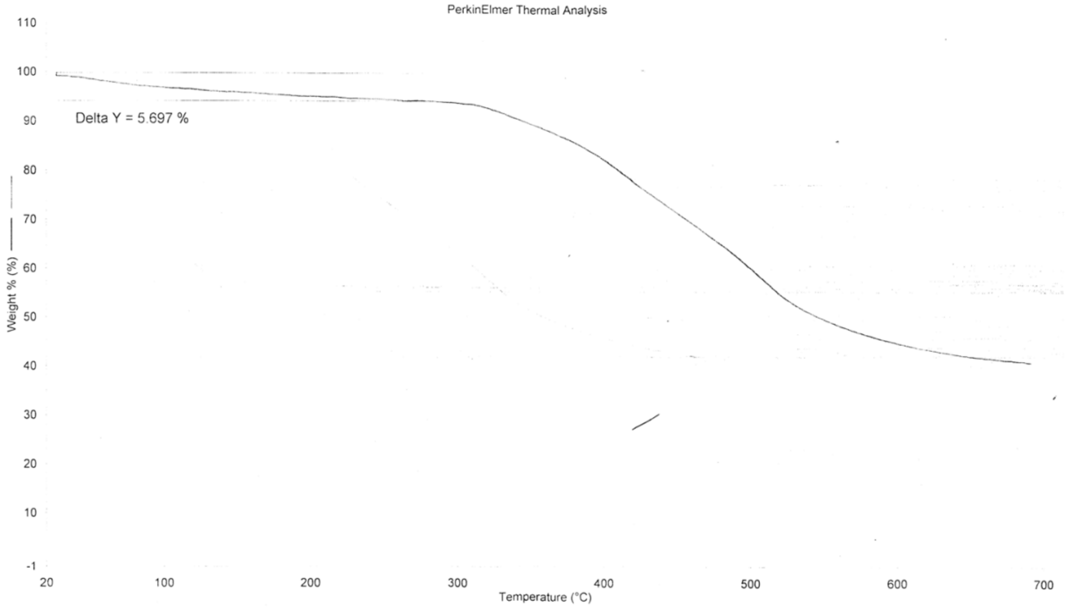

Figure S4. Thermogravimetric (TG) data for [4+4] cage.

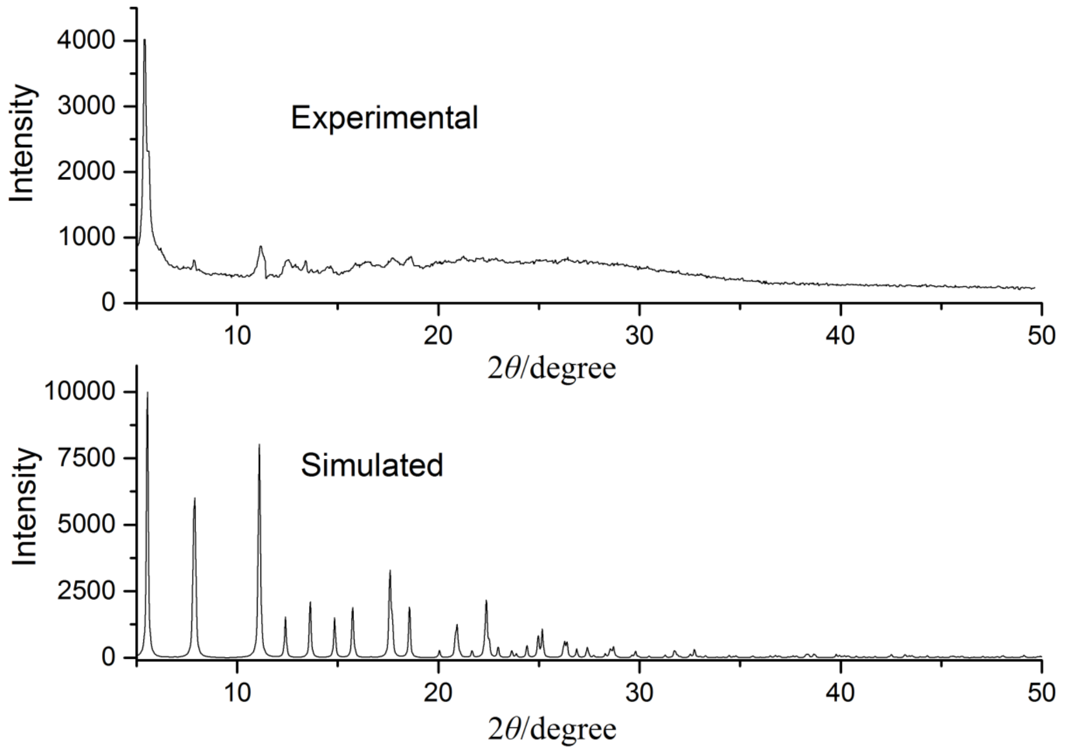

Figure S5. Powder X-ray diffraction patterns for [4+4] cage.

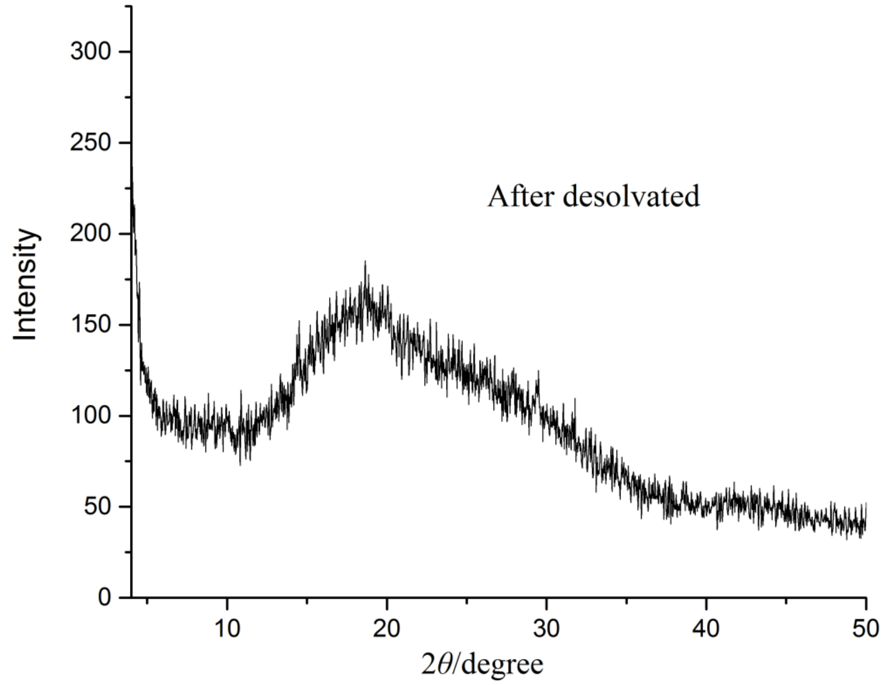

Figure S6. Powder X-ray diffraction pattern for [4+4] cage.

S6. Henry's constant fitting

Although there are deviations, low pressure range of the adsorption isotherm shows linear trend which corresponds to Henry's law behavior. Thus Henry's constant can be calculated from a linear fit to the initial part of the isotherm.[13,14]

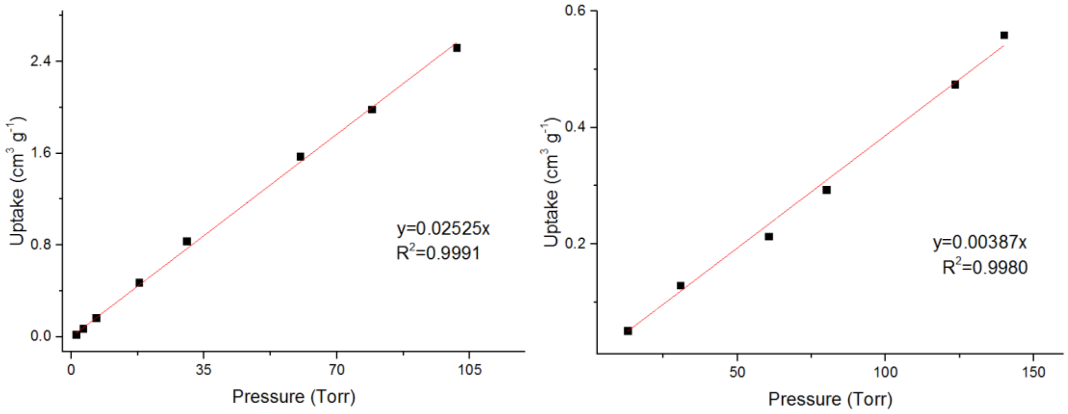

Figure S7. Fitting of CO_2 (left) and CH_4 (right) adsorption isotherms at 273 K.

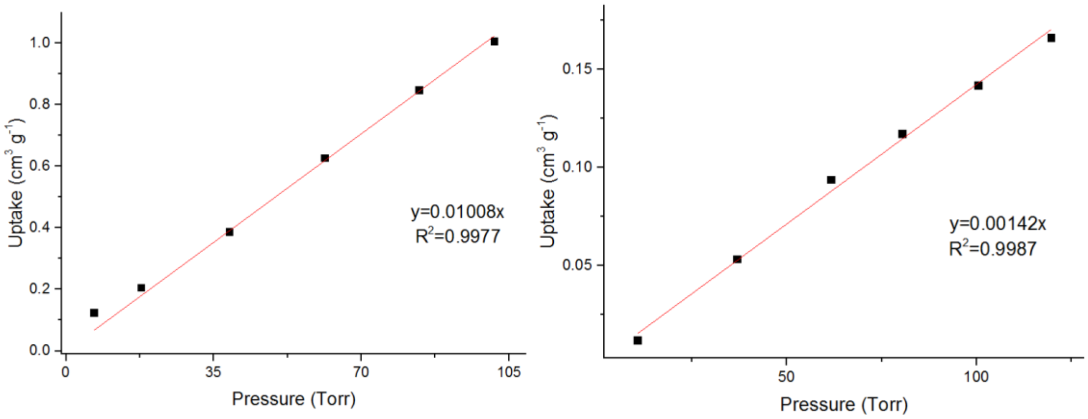

Figure S8. Fitting of CO_2 (left) and CH_4 (right) adsorption isotherms at 298 K.

S7. References:

1. Feng, G. *et al.* Cavity partition and functionalization of a [2+3] organic molecular cage by inserting polar P=O bonds. *Chem. Commun.* **52**, 9267–9270 (2016)

2. Elbert, M.*et al.* Synthesis of a rigid C_{3v} -symmetric tris -salicylaldehyde as a precursor for a highly

porous molecular cube. *Chem.–Eur. J.* **20**, 16707–16720 (2014)

3. Schneider, M. W. *et al.* Periphery‑substituted [4+6] salicylbisimine cage compounds with exceptionally high surface areas: influence of the molecular structure on nitrogen sorption properties. *Chem.–Eur. J.* **18**, 836–847 (2012)

4. Schneider, M. W. *et al.* Post‑modification of the interior of porous shape‑persistent organic cage compounds. *Angew. Chem., Int. Ed.* **52**, 3611–3615 (2013)

5. Schneider, M. W. *et al*. A shape-persistent exo-functionalized [4+6] imine cage compound with a very high specific surface area. *Chem. Commun.* **48**, 9861–9863 (2012)

6. Schneider, M. W. *et al.* Exo‑functionalized shape‑persistent [2+3] cage compounds: influence of molecular rigidity on formation and permanent porosity. *Chem.–Eur. J.* **18**, 4156–4160 (2012)

7. Tozawa, T. *et al.* Porous organic cages. *Nat. Mater.* **8**, 973–978 (2009).

8. Jiang, S. *et al.* Selective gas sorption in a [2+3] propeller cage crystal. *Chem. Commun.* **47**, 8919–8921 (2011).

9. Jin, Y. *et al.* Highly CO_2-selective organic molecular cages: what determines the CO_2 selectivity. *J. Am. Chem. Soc.* **133**, 6650–6658 (2011)

10. Ding, H. *et al.* Targeted synthesis of a large triazine-based [4+6] organic molecular cage: structure, porosity and gas separation. *Chem. Commun.* **51**, 1976–1979 (2015)

11. Hong, S. *et al.* Porphyrin boxes: rationally designed porous organic cages. *Angew. Chem. Int. Ed.* **54**, 13241–13244 (2015)

12. Bhat, A. S. *et al.* Transformation of a [4+6] Salicylbisimine Cage to Chemically Robust Amide Cages. *Angew. Chem. Int. Ed.* **58**, 8819 –8823 (2019)

13. Wang, H. *et al.* The first example of commensurate adsorption of atomic gas in a MOF and effective separation of xenon from other noble gases. *Chem. Sci.* **5**, 620–624 (2013)

14. Wright, J. S. *et al.* Highly selective CO_2 vs. N_2 adsorption in the cavity of a molecular coordination cage. *Chem. Commun.* **53**, 4398–4401 (2017)

S8. Crystallographic information file for tetrapodal [4+4] phosphate organic molecular cage

```
data_2_sqd

_audit_creation_method            'SHELXL-2014/7'
_shelx_SHELXL_version_number      '2014/7'
_chemical_name_systematic         ?
_chemical_name_common             ?
_chemical_melting_point           ?
_chemical_formula_moiety
'C108 H108 N16 O16 P4'
_chemical_formula_sum
 'C108 H108 N16 O16 P4'
_chemical_formula_weight          2009.98

loop_
 _atom_type_symbol
 _atom_type_description
 _atom_type_scat_dispersion_real
 _atom_type_scat_dispersion_imag
 _atom_type_scat_source
 'C'  'C'   0.0033   0.0016
 'International Tables Vol C Tables 4.2.6.8 and 6.1.1.4'
 'H'  'H'   0.0000   0.0000
 'International Tables Vol C Tables 4.2.6.8 and 6.1.1.4'
 'N'  'N'   0.0061   0.0033
 'International Tables Vol C Tables 4.2.6.8 and 6.1.1.4'
 'O'  'O'   0.0106   0.0060
 'International Tables Vol C Tables 4.2.6.8 and 6.1.1.4'
 'P'  'P'   0.1023   0.0942
 'International Tables Vol C Tables 4.2.6.8 and 6.1.1.4'

_space_group_crystal_system       tetragonal
_space_group_IT_number            82
_space_group_name_H-M_alt         'I -4'
_space_group_name_Hall            'I -4'

_shelx_space_group_comment
;
The symmetry employed for this shelxl refinement is uniquely defined
by the following loop, which should always be used as a source of
```

```
symmetry information in preference to the above space-group names.
They are only intended as comments.
;

loop_
 _space_group_symop_operation_xyz
 'x, y, z'
 '-x, -y, z'
 'y, -x, -z'
 '-y, x, -z'
 'x+1/2, y+1/2, z+1/2'
 '-x+1/2, -y+1/2, z+1/2'
 'y+1/2, -x+1/2, -z+1/2'
 '-y+1/2, x+1/2, -z+1/2'

_cell_length_a                    22.556(4)
_cell_length_b                    22.556(4)
_cell_length_c                    12.858(2)
_cell_angle_alpha                 90
_cell_angle_beta                  90
_cell_angle_gamma                 90
_cell_volume                      6542(3)
_cell_formula_units_Z             2
_cell_measurement_temperature     123(2)
_cell_measurement_reflns_used     5313
_cell_measurement_theta_min       2.554
_cell_measurement_theta_max       27.334

_exptl_crystal_description        block
_exptl_crystal_colour             colourless
_exptl_crystal_density_meas       ?
_exptl_crystal_density_method     'not measured'
_exptl_crystal_density_diffrn     1.020
_exptl_crystal_F_000              2112
_exptl_transmission_factor_min    ?
_exptl_transmission_factor_max    ?
_exptl_crystal_size_max           0.120
_exptl_crystal_size_mid           0.100
_exptl_crystal_size_min           0.100
_exptl_absorpt_coefficient_mu     0.116
_shelx_estimated_absorpt_T_min    0.986
_shelx_estimated_absorpt_T_max    0.989
_exptl_absorpt_correction_type    multi-scan
_exptl_absorpt_correction_T_min   0.986
```

```
_exptl_absorpt_correction_T_max   0.989
_exptl_absorpt_process_details    'SADABS, Bruker(2000)'
_exptl_absorpt_special_details    ?
_diffrn_ambient_temperature       123(2)
_diffrn_radiation_wavelength      0.71073
_diffrn_radiation_type            MoK\a
_diffrn_source                    'fine-focus sealed tube'
_diffrn_measurement_device_type   'Bruker Smart Apex CCD area detector'
_diffrn_measurement_method        '\f and \w scans'
_diffrn_detector_area_resol_mean  ?
_diffrn_reflns_number             20734
_diffrn_reflns_av_unetI/netI      0.1064
_diffrn_reflns_av_R_equivalents   0.0816
_diffrn_reflns_limit_h_min        -25
_diffrn_reflns_limit_h_max        26
_diffrn_reflns_limit_k_min        -26
_diffrn_reflns_limit_k_max        25
_diffrn_reflns_limit_l_min        -15
_diffrn_reflns_limit_l_max        13
_diffrn_reflns_theta_min          2.554
_diffrn_reflns_theta_max          24.993
_diffrn_reflns_theta_full         24.993
_diffrn_measured_fraction_theta_max   0.997
_diffrn_measured_fraction_theta_full  0.997
_diffrn_reflns_Laue_measured_fraction_max    0.997
_diffrn_reflns_Laue_measured_fraction_full   0.997
_diffrn_reflns_point_group_measured_fraction_max   0.998
_diffrn_reflns_point_group_measured_fraction_full  0.998
_reflns_number_total              5760
_reflns_number_gt                 3190
_reflns_threshold_expression      'I > 2\s(I)'
_reflns_Friedel_coverage          0.907
_reflns_Friedel_fraction_max      0.999
_reflns_Friedel_fraction_full     0.999

_reflns_special_details
;
 Reflections were merged by SHELXL according to the crystal
 class for the calculation of statistics and refinement.

 _reflns_Friedel_fraction is defined as the number of unique
 Friedel pairs measured divided by the number that would be
 possible theoretically, ignoring centric projections and
 systematic absences.
```

```
;

_computing_data_collection        'SMART (BRUKER 2000)'
_computing_cell_refinement        SMART
_computing_data_reduction         'SAINT (BRUKER 2000)'
_computing_structure_solution     'SHELXTL (BRUKER 2000)'
_computing_structure_refinement 'SHELXL-2014/7 (Sheldrick, 2014)'
_computing_molecular_graphics     'SHELXL-2014/7 (Sheldrick, 2014)'
_computing_publication_material   'SHELXL-2014/7 (Sheldrick, 2014)'
_refine_special_details
;
 Refinement of F^2^ against ALL reflections.  The weighted R-factor wR and
 goodness of fit S are based on F^2^, conventional R-factors R are based
 on F, with F set to zero for negative F^2^. The threshold expression of
 F^2^ > 2sigma(F^2^) is used only for calculating R-factors(gt) etc. and is
 not relevant to the choice of reflections for refinement.  R-factors based
 on F^2^ are statistically about twice as large as those based on F, and R-
 factors based on ALL data will be even larger.
;
_refine_ls_structure_factor_coef  Fsqd
_refine_ls_matrix_type            full
_refine_ls_weighting_scheme       calc
_refine_ls_weighting_details
'w=1/[\s^2^(Fo^2^)+(0.0928P)^2^+17.8890P] where P=(Fo^2^+2Fc^2^)/3'
_atom_sites_solution_primary      direct
_atom_sites_solution_secondary    difmap
_atom_sites_solution_hydrogens    geom
_refine_ls_hydrogen_treatment     constr
_refine_ls_extinction_method      none
_refine_ls_extinction_coef        .
_refine_ls_number_reflns          5760
_refine_ls_number_parameters      326
_refine_ls_number_restraints      0
_refine_ls_R_factor_all           0.1504
_refine_ls_R_factor_gt            0.0984
_refine_ls_wR_factor_ref          0.2453
_refine_ls_wR_factor_gt           0.2243
_refine_ls_goodness_of_fit_ref    1.004
_refine_ls_restrained_S_all       1.004
_refine_ls_shift/su_max           0.000
_refine_ls_shift/su_mean          0.000

loop_
 _atom_site_label
```

```
 _atom_site_type_symbol
 _atom_site_fract_x
 _atom_site_fract_y
 _atom_site_fract_z
 _atom_site_U_iso_or_equiv
 _atom_site_adp_type
 _atom_site_occupancy
 _atom_site_site_symmetry_order
 _atom_site_calc_flag
 _atom_site_refinement_flags_posn
 _atom_site_refinement_flags_adp
 _atom_site_refinement_flags_occupancy
 _atom_site_disorder_assembly
 _atom_site_disorder_group
C1 C 0.0766(5) 0.1100(4) 0.2078(7) 0.051(3) Uani 1 1 d . . . . .
C2 C 0.1184(5) 0.0882(6) 0.1504(9) 0.077(4) Uani 1 1 d . . . . .
H2 H 0.1091 0.0665 0.0893 0.092 Uiso 1 1 calc R U . . .
C3 C 0.1761(5) 0.0970(6) 0.1787(10) 0.082(4) Uani 1 1 d . . . . .
H3 H 0.2064 0.0836 0.1331 0.099 Uiso 1 1 calc R U . . .
C4 C 0.1927(5) 0.1246(4) 0.2713(8) 0.051(2) Uani 1 1 d . . . . .
C5 C 0.1478(5) 0.1486(4) 0.3301(9) 0.063(3) Uani 1 1 d . . . . .
H5 H 0.1561 0.1698 0.3922 0.076 Uiso 1 1 calc R U . . .
C6 C 0.0888(5) 0.1408(4) 0.2957(8) 0.061(3) Uani 1 1 d . . . . .
H6 H 0.0572 0.1574 0.3349 0.073 Uiso 1 1 calc R U . . .
C7 C 0.2545(5) 0.1311(4) 0.2936(8) 0.057(3) Uani 1 1 d . . . . .
H7 H 0.2827 0.1138 0.2479 0.068 Uiso 1 1 calc R U . . .
C8 C -0.0932(5) 0.1079(5) 0.3302(8) 0.060(3) Uani 1 1 d . . . . .
C9 C -0.0716(6) 0.0515(5) 0.3481(10) 0.082(4) Uani 1 1 d . . . . .
H9 H -0.0324 0.0397 0.3310 0.098 Uiso 1 1 calc R U . . .
C10 C -0.1139(7) 0.0128(6) 0.3949(12) 0.101(5) Uani 1 1 d . . . . .
H10 H -0.1036 -0.0279 0.4002 0.121 Uiso 1 1 calc R U . . .
C11 C -0.1654(5) 0.0287(5) 0.4310(9) 0.061(3) Uani 1 1 d . . . . .
C12 C -0.1827(5) 0.0901(6) 0.4105(9) 0.071(3) Uani 1 1 d . . . . .
H12 H -0.2206 0.1033 0.4328 0.085 Uiso 1 1 calc R U . . .
C13 C -0.1490(5) 0.1260(5) 0.3642(7) 0.053(3) Uani 1 1 d . . . . .
H13 H -0.1616 0.1656 0.3525 0.064 Uiso 1 1 calc R U . . .
C14 C -0.2022(5) -0.0123(6) 0.4878(7) 0.067(3) Uani 1 1 d . . . . .
H14 H -0.1937 -0.0536 0.4861 0.080 Uiso 1 1 calc R U . . .
C15 C -0.0011(5) 0.2309(5) 0.0504(8) 0.068(3) Uani 1 1 d . . . . .
C16 C -0.0287(5) 0.2828(5) 0.0286(10) 0.076(4) Uani 1 1 d . . . . .
H16 H -0.0553 0.2994 0.0780 0.092 Uiso 1 1 calc R U . . .
C17 C -0.0190(5) 0.3115(5) -0.0630(7) 0.050(3) Uani 1 1 d . . . . .
H17 H -0.0372 0.3490 -0.0739 0.060 Uiso 1 1 calc R U . . .
C18 C 0.0160(6) 0.2882(5) -0.1401(10) 0.071(3) Uani 1 1 d . . . . .
```

C19 C 0.0423(5) 0.2311(6) -0.1189(10) 0.071(3) Uani 1 1 d . . . . .
H19 H 0.0659 0.2117 -0.1699 0.086 Uiso 1 1 calc R U . . .
C20 C 0.0326(5) 0.2053(5) -0.0235(8) 0.059(3) Uani 1 1 d . . . . .
H20 H 0.0503 0.1679 -0.0092 0.071 Uiso 1 1 calc R U . . .
C21 C 0.0237(5) 0.3189(6) -0.2414(9) 0.064(3) Uani 1 1 d . . . . .
H21 H 0.0458 0.2996 -0.2944 0.076 Uiso 1 1 calc R U . . .
C22 C 0.3361(6) 0.1616(6) 0.3874(11) 0.083(4) Uani 1 1 d . . . . .
H22A H 0.3551 0.1428 0.3264 0.099 Uiso 1 1 calc R U . . .
H22B H 0.3493 0.2035 0.3899 0.099 Uiso 1 1 calc R U . . .
C23 C 0.3550(6) 0.1328(6) 0.4775(10) 0.080(4) Uani 1 1 d . . . . .
H23A H 0.3293 0.1452 0.5360 0.096 Uiso 1 1 calc R U . . .
H23B H 0.3959 0.1457 0.4936 0.096 Uiso 1 1 calc R U . . .
C24 C 0.3443(5) 0.0342(8) 0.5668(8) 0.099(5) Uani 1 1 d . . . . .
H24A H 0.3699 0.0514 0.6216 0.118 Uiso 1 1 calc R U . . .
H24B H 0.3554 -0.0079 0.5580 0.118 Uiso 1 1 calc R U . . .
C25 C 0.2779(6) 0.0386(7) 0.6008(10) 0.084(4) Uani 1 1 d . . . . .
H25A H 0.2739 0.0311 0.6763 0.101 Uiso 1 1 calc R U . . .
H25B H 0.2618 0.0784 0.5851 0.101 Uiso 1 1 calc R U . . .
C26 C 0.4100(5) 0.0412(6) 0.4197(8) 0.070(3) Uani 1 1 d . . . . .
H26A H 0.4402 0.0335 0.4739 0.084 Uiso 1 1 calc R U . . .
H26B H 0.4263 0.0705 0.3701 0.084 Uiso 1 1 calc R U . . .
C27 C 0.0171(6) 0.3951(6) -0.3619(9) 0.087(4) Uani 1 1 d . . . . .
H27A H 0.0410 0.3671 -0.4039 0.104 Uiso 1 1 calc R U . . .
H27B H 0.0406 0.4317 -0.3515 0.104 Uiso 1 1 calc R U . . .
N1 N 0.2733(4) 0.1600(4) 0.3746(8) 0.070(3) Uani 1 1 d . . . . .
N2 N -0.2466(4) 0.0068(5) 0.5410(7) 0.074(3) Uani 1 1 d . . . . .
N3 N 0.0029(4) 0.3680(4) -0.2596(6) 0.055(2) Uani 1 1 d . . . . .
N4 N 0.3538(4) 0.0657(5) 0.4697(7) 0.070(3) Uani 1 1 d . . . . .
O1 O 0.0186(3) 0.1002(3) 0.1738(6) 0.071(2) Uani 1 1 d . . . . .
O2 O -0.0561(4) 0.1489(4) 0.2822(6) 0.077(2) Uani 1 1 d . . . . .
O3 O -0.0093(3) 0.2016(4) 0.1519(5) 0.070(2) Uani 1 1 d . . . . .
O4 O -0.0797(4) 0.1218(4) 0.1008(7) 0.095(3) Uani 1 1 d . . . . .
P1 P -0.03444(15) 0.14358(14) 0.16743(19) 0.0622(8) Uani 1 1 d . . . . .

loop_
 _atom_site_aniso_label
 _atom_site_aniso_U_11
 _atom_site_aniso_U_22
 _atom_site_aniso_U_33
 _atom_site_aniso_U_23
 _atom_site_aniso_U_13
 _atom_site_aniso_U_12
C1 0.066(8) 0.047(6) 0.041(6) 0.003(5) 0.004(5) 0.004(5)
C2 0.050(7) 0.110(10) 0.071(8) -0.028(7) -0.020(6) 0.031(7)

C3 0.061(8) 0.113(10) 0.072(8) -0.019(7) 0.010(6) 0.034(7)
C4 0.057(7) 0.041(6) 0.054(6) -0.007(5) 0.004(5) 0.007(5)
C5 0.080(8) 0.052(6) 0.057(6) -0.013(5) 0.031(6) 0.000(6)
C6 0.059(7) 0.058(7) 0.065(7) 0.000(5) 0.034(5) 0.004(5)
C7 0.067(8) 0.036(6) 0.067(7) -0.010(5) 0.003(6) 0.015(5)
C8 0.081(8) 0.048(7) 0.050(6) 0.005(5) 0.010(6) -0.013(6)
C9 0.094(10) 0.061(8) 0.090(9) 0.002(7) 0.041(7) -0.001(7)
C10 0.122(13) 0.065(9) 0.116(12) -0.013(8) 0.042(10) -0.005(9)
C11 0.061(7) 0.046(7) 0.075(7) -0.029(5) 0.025(6) -0.018(5)
C12 0.039(7) 0.101(11) 0.073(8) -0.010(7) 0.012(6) 0.002(7)
C13 0.064(7) 0.044(6) 0.052(6) -0.004(4) 0.011(5) 0.016(5)
C14 0.077(9) 0.097(9) 0.027(5) -0.017(5) -0.001(5) -0.010(7)
C15 0.078(8) 0.075(9) 0.050(7) -0.017(6) -0.012(6) -0.020(7)
C16 0.071(8) 0.055(8) 0.103(10) 0.040(7) -0.049(7) -0.021(6)
C17 0.058(7) 0.049(6) 0.043(6) 0.000(5) -0.007(5) -0.014(5)
C18 0.081(9) 0.056(7) 0.077(9) -0.003(6) -0.021(7) -0.022(7)
C19 0.050(7) 0.085(9) 0.079(9) -0.021(7) -0.019(6) -0.001(6)
C20 0.081(8) 0.061(7) 0.036(6) 0.011(5) -0.020(5) -0.019(6)
C21 0.061(7) 0.067(8) 0.063(7) -0.039(6) 0.005(5) 0.000(6)
C22 0.078(9) 0.068(8) 0.102(10) 0.018(7) -0.022(7) -0.025(7)
C23 0.072(9) 0.095(10) 0.074(9) -0.028(7) -0.009(7) -0.031(7)
C24 0.033(6) 0.232(17) 0.031(6) 0.002(8) 0.001(4) -0.028(8)
C25 0.068(9) 0.101(10) 0.083(9) -0.003(8) 0.000(7) -0.013(7)
C26 0.039(6) 0.124(11) 0.047(6) 0.010(7) 0.000(5) 0.001(6)
C27 0.106(11) 0.095(10) 0.059(8) -0.003(6) 0.008(7) -0.042(8)
N1 0.044(6) 0.066(6) 0.100(8) -0.005(5) -0.006(5) -0.007(5)
N2 0.067(7) 0.100(8) 0.054(6) -0.008(5) -0.013(5) -0.015(6)
N3 0.067(6) 0.049(6) 0.051(5) -0.010(4) 0.008(4) -0.027(5)
N4 0.051(6) 0.112(9) 0.048(5) -0.004(5) 0.003(4) -0.013(6)
O1 0.081(6) 0.074(5) 0.057(4) 0.004(4) 0.000(4) -0.013(4)
O2 0.086(6) 0.085(6) 0.059(4) -0.010(4) 0.001(4) -0.027(5)
O3 0.052(4) 0.111(7) 0.046(4) 0.000(4) -0.010(3) -0.010(4)
O4 0.088(6) 0.101(7) 0.096(7) 0.022(5) -0.020(5) -0.020(5)
P1 0.088(2) 0.067(2) 0.0318(12) -0.0005(13) -0.0061(14) 0.0055(18)

_geom_special_details
;
All esds (except the esd in the dihedral angle between two l.s. planes)
are estimated using the full covariance matrix. The cell esds are taken
into account individually in the estimation of esds in distances, angles
and torsion angles; correlations between esds in cell parameters are only
used when they are defined by crystal symmetry. An approximate (isotropic)
treatment of cell esds is used for estimating esds involving l.s. planes.
;

```
loop_
 _geom_bond_atom_site_label_1
 _geom_bond_atom_site_label_2
 _geom_bond_distance
 _geom_bond_site_symmetry_2
 _geom_bond_publ_flag
C1 C2 1.294(14) . ?
C1 C6 1.356(14) . ?
C1 O1 1.397(12) . ?
C2 C3 1.365(16) . ?
C2 H2 0.9500 . ?
C3 C4 1.395(16) . ?
C3 H3 0.9500 . ?
C4 C5 1.375(13) . ?
C4 C7 1.431(15) . ?
C5 C6 1.411(15) . ?
C5 H5 0.9500 . ?
C6 H6 0.9500 . ?
C7 N1 1.299(13) . ?
C7 H7 0.9500 . ?
C8 C9 1.383(15) . ?
C8 O2 1.392(12) . ?
C8 C13 1.392(15) . ?
C9 C10 1.426(17) . ?
C9 H9 0.9500 . ?
C10 C11 1.302(16) . ?
C10 H10 0.9500 . ?
C11 C14 1.442(15) . ?
C11 C12 1.463(16) . ?
C12 C13 1.262(15) . ?
C12 H12 0.9500 . ?
C13 H13 0.9500 . ?
C14 N2 1.288(14) . ?
C14 H14 0.9500 . ?
C15 C20 1.348(15) . ?
C15 C16 1.355(16) . ?
C15 O3 1.473(13) . ?
C16 C17 1.361(14) . ?
C16 H16 0.9500 . ?
C17 C18 1.371(16) . ?
C17 H17 0.9500 . ?
C18 C19 1.445(17) . ?
C18 C21 1.485(17) . ?
```

C19 C20 1.375(15) . ?
C19 H19 0.9500 . ?
C20 H20 0.9500 . ?
C21 N3 1.225(13) . ?
C21 H21 0.9500 . ?
C22 C23 1.394(17) . ?
C22 N1 1.427(14) . ?
C22 H22A 0.9900 . ?
C22 H22B 0.9900 . ?
C23 N4 1.518(15) . ?
C23 H23A 0.9900 . ?
C23 H23B 0.9900 . ?
C24 N4 1.452(14) . ?
C24 C25 1.563(16) . ?
C24 H24A 0.9900 . ?
C24 H24B 0.9900 . ?
C25 N2 1.463(15) 2 ?
C25 H25A 0.9900 . ?
C25 H25B 0.9900 . ?
C26 N4 1.524(14) . ?
C26 C27 1.546(17) 3 ?
C26 H26A 0.9900 . ?
C26 H26B 0.9900 . ?
C27 N3 1.486(14) . ?
C27 C26 1.546(17) 4 ?
C27 H27A 0.9900 . ?
C27 H27B 0.9900 . ?
N2 C25 1.463(15) 2 ?
O1 P1 1.546(8) . ?
O2 P1 1.559(8) . ?
O3 P1 1.442(8) . ?
O4 P1 1.421(9) . ?

loop_
 _geom_angle_atom_site_label_1
 _geom_angle_atom_site_label_2
 _geom_angle_atom_site_label_3
 _geom_angle
 _geom_angle_site_symmetry_1
 _geom_angle_site_symmetry_3
 _geom_angle_publ_flag
C2 C1 C6 121.4(11) . . ?
C2 C1 O1 116.4(9) . . ?
C6 C1 O1 122.2(9) . . ?

C1 C2 C3 119.2(11) . . ?
C1 C2 H2 120.4 . . ?
C3 C2 H2 120.4 . . ?
C2 C3 C4 123.2(10) . . ?
C2 C3 H3 118.4 . . ?
C4 C3 H3 118.4 . . ?
C5 C4 C3 116.6(10) . . ?
C5 C4 C7 124.6(10) . . ?
C3 C4 C7 118.6(9) . . ?
C4 C5 C6 118.2(10) . . ?
C4 C5 H5 120.9 . . ?
C6 C5 H5 120.9 . . ?
C1 C6 C5 121.1(10) . . ?
C1 C6 H6 119.4 . . ?
C5 C6 H6 119.4 . . ?
N1 C7 C4 122.0(10) . . ?
N1 C7 H7 119.0 . . ?
C4 C7 H7 119.0 . . ?
C9 C8 O2 118.2(10) . . ?
C9 C8 C13 122.5(10) . . ?
O2 C8 C13 119.2(10) . . ?
C8 C9 C10 113.4(12) . . ?
C8 C9 H9 123.3 . . ?
C10 C9 H9 123.3 . . ?
C11 C10 C9 125.4(13) . . ?
C11 C10 H10 117.3 . . ?
C9 C10 H10 117.3 . . ?
C10 C11 C14 121.2(12) . . ?
C10 C11 C12 115.8(11) . . ?
C14 C11 C12 123.0(10) . . ?
C13 C12 C11 122.2(10) . . ?
C13 C12 H12 118.9 . . ?
C11 C12 H12 118.9 . . ?
C12 C13 C8 120.3(10) . . ?
C12 C13 H13 119.9 . . ?
C8 C13 H13 119.9 . . ?
N2 C14 C11 120.1(12) . . ?
N2 C14 H14 119.9 . . ?
C11 C14 H14 119.9 . . ?
C20 C15 C16 118.9(12) . . ?
C20 C15 O3 120.2(11) . . ?
C16 C15 O3 120.9(11) . . ?
C15 C16 C17 121.1(14) . . ?
C15 C16 H16 119.5 . . ?

C17 C16 H16 119.5 . . ?
C16 C17 C18 122.4(12) . . ?
C16 C17 H17 118.8 . . ?
C18 C17 H17 118.8 . . ?
C17 C18 C19 116.2(11) . . ?
C17 C18 C21 121.6(11) . . ?
C19 C18 C21 122.1(12) . . ?
C20 C19 C18 118.7(12) . . ?
C20 C19 H19 120.7 . . ?
C18 C19 H19 120.7 . . ?
C15 C20 C19 122.5(11) . . ?
C15 C20 H20 118.7 . . ?
C19 C20 H20 118.7 . . ?
N3 C21 C18 122.9(10) . . ?
N3 C21 H21 118.5 . . ?
C18 C21 H21 118.5 . . ?
C23 C22 N1 112.8(11) . . ?
C23 C22 H22A 109.0 . . ?
N1 C22 H22A 109.0 . . ?
C23 C22 H22B 109.0 . . ?
N1 C22 H22B 109.0 . . ?
H22A C22 H22B 107.8 . . ?
C22 C23 N4 113.9(10) . . ?
C22 C23 H23A 108.8 . . ?
N4 C23 H23A 108.8 . . ?
C22 C23 H23B 108.8 . . ?
N4 C23 H23B 108.8 . . ?
H23A C23 H23B 107.7 . . ?
N4 C24 C25 110.6(10) . . ?
N4 C24 H24A 109.5 . . ?
C25 C24 H24A 109.5 . . ?
N4 C24 H24B 109.5 . . ?
C25 C24 H24B 109.5 . . ?
H24A C24 H24B 108.1 . . ?
N2 C25 C24 105.7(11) 2 . ?
N2 C25 H25A 110.6 2 . ?
C24 C25 H25A 110.6 . . ?
N2 C25 H25B 110.6 2 . ?
C24 C25 H25B 110.6 . . ?
H25A C25 H25B 108.7 . . ?
N4 C26 C27 109.3(9) . 3 ?
N4 C26 H26A 109.8 . . ?
C27 C26 H26A 109.8 3 . ?
N4 C26 H26B 109.8 . . ?

C27 C26 H26B 109.8 3 . ?
H26A C26 H26B 108.3 . . ?
N3 C27 C26 109.4(10) . 4 ?
N3 C27 H27A 109.8 . . ?
C26 C27 H27A 109.8 4 . ?
N3 C27 H27B 109.8 . . ?
C26 C27 H27B 109.8 4 . ?
H27A C27 H27B 108.2 . . ?
C7 N1 C22 115.4(11) . . ?
C14 N2 C25 114.8(12) . 2 ?
C21 N3 C27 117.3(10) . . ?
C24 N4 C23 115.7(10) . . ?
C24 N4 C26 107.9(9) . . ?
C23 N4 C26 112.1(9) . . ?
C1 O1 P1 129.9(7) . . ?
C8 O2 P1 123.9(7) . . ?
P1 O3 C15 125.4(6) . . ?
O4 P1 O3 120.9(5) . . ?
O4 P1 O1 111.7(5) . . ?
O3 P1 O1 106.1(4) . . ?
O4 P1 O2 111.8(5) . . ?
O3 P1 O2 100.7(4) . . ?
O1 P1 O2 104.0(5) . . ?

```
_refine_diff_density_max    0.421
_refine_diff_density_min   -0.349
_refine_diff_density_rms    0.079

_shelx_res_file
;

    2_sqd.res created by SHELXL-2014/7

TITL 2 in I-4
CELL 0.71073  22.55610  22.55610  12.85750  90.0000  90.0000  90.0000
ZERR   2.00   0.00430   0.00430   0.00240   0.0000   0.0000   0.0000
LATT -2
SYMM -X, -Y, Z
SYMM Y, -X, -Z
SYMM -Y, X, -Z
SFAC C  H  N  O  P
UNIT 216 216 32 32 8
ACTA
OMIT     2   8   0
```

```
OMIT    -1   3   0
OMIT    -2   5   1
OMIT     5   2   1
OMIT   0.00  50.00
TWIN
SIZE 0.10 0.10 0.12
L.S. 5
BOND $H
FMAP 2
PLAN 5
TEMP -150.000
WGHT    0.092800    17.889000
BASF   0.47755
FVAR       1.10310
MOLE 1
C1    1    0.076563    0.109962    0.207785    11.00000    0.06618    0.04658 =
         0.04134    0.00336    0.00386    0.00420
C2    1    0.118410    0.088248    0.150380    11.00000    0.04985    0.10959 =
         0.07087   -0.02794   -0.02042    0.03056
AFIX  43
H2    2    0.109098    0.066480    0.089273    11.00000   -1.20000
AFIX   0
C3    1    0.176091    0.096999    0.178693    11.00000    0.06067    0.11340 =
         0.07225   -0.01873    0.01000    0.03393
AFIX  43
H3    2    0.206410    0.083612    0.133100    11.00000   -1.20000
AFIX   0
C4    1    0.192686    0.124633    0.271281    11.00000    0.05714    0.04096 =
         0.05394   -0.00675    0.00435    0.00744
C5    1    0.147751    0.148562    0.330099    11.00000    0.07972    0.05201 =
         0.05746   -0.01315    0.03148   -0.00002
AFIX  43
H5    2    0.156136    0.169779    0.392207    11.00000   -1.20000
AFIX   0
C6    1    0.088844    0.140763    0.295748    11.00000    0.05869    0.05841 =
         0.06528   -0.00017    0.03438    0.00365
AFIX  43
H6    2    0.057242    0.157383    0.334936    11.00000   -1.20000
AFIX   0
C7    1    0.254509    0.131118    0.293641    11.00000    0.06737    0.03601 =
         0.06651   -0.00961    0.00341    0.01468
AFIX  43
H7    2    0.282658    0.113751    0.247897    11.00000   -1.20000
AFIX   0
```

```
C8    1   -0.093247    0.107914    0.330160    11.00000    0.08051    0.04830 =
        0.04981    0.00504    0.00964   -0.01337
C9    1   -0.071578    0.051495    0.348142    11.00000    0.09397    0.06084 =
        0.09031    0.00198    0.04144   -0.00096
AFIX  43
H9    2   -0.032352    0.039672    0.331005    11.00000   -1.20000
AFIX   0
C10   1   -0.113870    0.012777    0.394935    11.00000    0.12208    0.06515 =
        0.11595   -0.01337    0.04153   -0.00455
AFIX  43
H10   2   -0.103574   -0.027949    0.400248    11.00000   -1.20000
AFIX   0
C11   1   -0.165428    0.028663    0.430967    11.00000    0.06126    0.04621 =
        0.07529   -0.02875    0.02458   -0.01764
C12   1   -0.182741    0.090077    0.410498    11.00000    0.03931    0.10136 =
        0.07284   -0.00974    0.01168    0.00242
AFIX  43
H12   2   -0.220647    0.103316    0.432804    11.00000   -1.20000
AFIX   0
C13   1   -0.148982    0.126035    0.364192    11.00000    0.06354    0.04407 =
        0.05194   -0.00354    0.01060    0.01636
AFIX  43
H13   2   -0.161646    0.165646    0.352496    11.00000   -1.20000
AFIX   0
C14   1   -0.202209   -0.012337    0.487844    11.00000    0.07658    0.09659 =
        0.02666   -0.01739   -0.00106   -0.01021
AFIX  43
H14   2   -0.193654   -0.053565    0.486107    11.00000   -1.20000
AFIX   0
C15   1   -0.001113    0.230882    0.050441    11.00000    0.07818    0.07497 =
        0.04994   -0.01714   -0.01158   -0.02021
C16   1   -0.028723    0.282775    0.028588    11.00000    0.07075    0.05516 =
        0.10286    0.03988   -0.04890   -0.02092
AFIX  43
H16   2   -0.055272    0.299397    0.077982    11.00000   -1.20000
AFIX   0
C17   1   -0.019030    0.311460   -0.062978    11.00000    0.05814    0.04896 =
        0.04325    0.00021   -0.00701   -0.01436
AFIX  43
H17   2   -0.037169    0.348959   -0.073882    11.00000   -1.20000
AFIX   0
C18   1    0.015956    0.288211   -0.140055    11.00000    0.08111    0.05626 =
        0.07680   -0.00316   -0.02060   -0.02193
C19   1    0.042318    0.231076   -0.118938    11.00000    0.04982    0.08528 =
```

```
          0.07885    -0.02132    -0.01877    -0.00105
AFIX  43
H19   2     0.065881     0.211734    -0.169920    11.00000    -1.20000
AFIX   0
C20   1     0.032604     0.205268    -0.023541    11.00000     0.08076     0.06066 =
          0.03637     0.01121    -0.01989    -0.01900
AFIX  43
H20   2     0.050341     0.167948    -0.009231    11.00000    -1.20000
AFIX   0
C21   1     0.023700     0.318870    -0.241362    11.00000     0.06094     0.06721 =
          0.06270    -0.03906     0.00479     0.00039
AFIX  43
H21   2     0.045767     0.299567    -0.294408    11.00000    -1.20000
AFIX   0
C22   1     0.336114     0.161641     0.387423    11.00000     0.07753     0.06843 =
          0.10206     0.01777    -0.02218    -0.02505
AFIX  23
H22A  2     0.355073     0.142770     0.326379    11.00000    -1.20000
H22B  2     0.349289     0.203483     0.389907    11.00000    -1.20000
AFIX   0
C23   1     0.354964     0.132824     0.477462    11.00000     0.07187     0.09501 =
          0.07408    -0.02774    -0.00918    -0.03072
AFIX  23
H23A  2     0.329315     0.145215     0.536041    11.00000    -1.20000
H23B  2     0.395915     0.145668     0.493576    11.00000    -1.20000
AFIX   0
C24   1     0.344313     0.034230     0.566767    11.00000     0.03348     0.23191 =
          0.03067     0.00163     0.00148    -0.02823
AFIX  23
H24A  2     0.369895     0.051447     0.621562    11.00000    -1.20000
H24B  2     0.355406    -0.007941     0.558009    11.00000    -1.20000
AFIX   0
C25   1     0.277944     0.038588     0.600800    11.00000     0.06810     0.10103 =
          0.08319    -0.00324    -0.00026    -0.01294
AFIX  23
H25A  2     0.273923     0.031079     0.676334    11.00000    -1.20000
H25B  2     0.261839     0.078430     0.585145    11.00000    -1.20000
AFIX   0
C26   1     0.409955     0.041162     0.419671    11.00000     0.03915     0.12373 =
          0.04744     0.00984     0.00034     0.00083
AFIX  23
H26A  2     0.440178     0.033477     0.473854    11.00000    -1.20000
H26B  2     0.426324     0.070478     0.370083    11.00000    -1.20000
AFIX   0
```

```
C27   1    0.017056    0.395061   -0.361899    11.00000    0.10623    0.09542 =
         0.05940   -0.00289    0.00840   -0.04186
AFIX  23
H27A  2    0.040951    0.367110   -0.403934    11.00000   -1.20000
H27B  2    0.040561    0.431651   -0.351505    11.00000   -1.20000
AFIX   0
N1    3    0.273312    0.160025    0.374576    11.00000    0.04434    0.06640 =
         0.09989   -0.00505   -0.00584   -0.00703
N2    3   -0.246636    0.006824    0.540997    11.00000    0.06665    0.10043 =
         0.05410   -0.00849   -0.01259   -0.01511
N3    3    0.002888    0.367961   -0.259553    11.00000    0.06678    0.04858 =
         0.05057   -0.01045    0.00805   -0.02686
N4    3    0.353803    0.065683    0.469651    11.00000    0.05098    0.11207 =
         0.04772   -0.00402    0.00317   -0.01331
O1    4    0.018555    0.100236    0.173787    11.00000    0.08128    0.07370 =
         0.05662    0.00402    0.00032   -0.01293
O2    4   -0.056134    0.148911    0.282177    11.00000    0.08649    0.08500 =
         0.05905   -0.00961    0.00109   -0.02722
O3    4   -0.009266    0.201649    0.151856    11.00000    0.05243    0.11093 =
         0.04604   -0.00030   -0.01020   -0.00974
O4    4   -0.079725    0.121803    0.100799    11.00000    0.08833    0.10067 =
         0.09633    0.02225   -0.01985   -0.02003
P1    5   -0.034440    0.143578    0.167427    11.00000    0.08808    0.06680 =
         0.03178   -0.00048   -0.00608    0.00554
HKLF 4

REM  2 in I-4
REM R1 =  0.0984 for    3190 Fo > 4sig(Fo)  and  0.1504 for all    5760 data
REM    326 parameters refined using      0 restraints

END

WGHT      0.1659      0.0000

REM Highest difference peak  0.421,  deepest hole -0.349,  1-sigma level  0.079
Q1    1  -0.1038  0.0972  0.2268  11.00000  0.05    0.42
Q2    1   0.0036  0.1693  0.0478  11.00000  0.05    0.40
Q3    1  -0.1009  0.0493  0.2466  11.00000  0.05    0.34
Q4    1   0.0013  0.1678  0.1810  11.00000  0.05    0.30
Q5    1   0.0100  0.1308  0.2400  11.00000  0.05    0.30
;
_shelx_res_checksum   10767

_shelx_hkl_file
```

;
-6 0 0 41801 2815
73453 57.70 -2.43 1.0000
6 0 0 38116 2790
68545 57.70 2.43 1.0000
6 0 0 39069 2814
69821 57.70 2.43 1.0000
6 0 0 40204 2789
71336 57.70 2.43 1.0000
-8 0 0 19606 330
6579 -51.08 1.64 1.0000
8 0 0 19653 295
6605 -51.08 -1.64 1.0000
8 0 0 19621 335
6586 -51.08 -1.64 1.0000
8 0 0 20611 296
7165 -51.08 -1.64 1.0000
10 0 0 2577 156
955 17.96 2.24 1.0000
12 0 0 1 142
472 17.80 0.55 1.0000
14 0 0 874 152
348 -9.46 -0.18 1.0000
16 0 0 2299 215
1167 11.95 -0.28 1.0000
18 0 0 962 229
521 7.11 -0.50 1.0000
20 0 0 132 227
65 -3.10 0.38 1.0000
22 0 0 419 255
249 -4.06 -0.02 1.0000
24 0 0 2 276
15 -2.15 -0.06 1.0000
26 0 0 52 326
62 0.59 -0.07 1.0000
25 -1 0 152 313
253 -3.09 -0.14 1.0000
23 -1 0 33 251
-73 4.95 0.14 1.0000
21 -1 0 7 226
-1 2.29 -0.22 1.0000
19 -1 0 31 195
-115 4.77 0.75 1.0000
17 -1 0 61 231

1281	-24.25	-0.55	1.0000	
15	-1	0	2	165
156	9.68	1.14	1.0000	
13	-1	0	266	169
2117	-25.74	0.18	1.0000	
13	-1	0	213	227
1960	-25.74	0.18	1.0000	
11	-1	0	1413	136
499	-13.21	0.45	1.0000	
9	-1	0	777	224
3413	26.46	-1.15	1.0000	
9	-1	0	830	180
3522	26.46	-1.15	1.0000	
7	-1	0	803	381
7889	-52.44	-0.92	1.0000	
7	-1	0	1081	353
8719	-52.44	-0.92	1.0000	
7	-1	0	1004	351
8498	-52.44	-0.92	1.0000	
-7	1	0	819	378
7942	-52.44	0.92	1.0000	
5	-1	0	14827	579
13819	3.65	0.87	1.0000	
5	-1	0	14575	558
13576	3.65	0.87	1.0000	
-5	1	0	14999	577
13987	3.65	-0.87	1.0000	
3	-1	0	63456	2077
51916	20.85	0.23	1.0000	
3	-1	0	64382	2088
52754	20.85	0.23	1.0000	
-3	1	0	65389	2088
53666	20.85	-0.23	1.0000	
-3	-1	0	481	72
836	6.05	0.01	1.0000	
-3	-1	0	617	58
1013	6.05	0.01	1.0000	
-3	-1	0	787	55
1227	6.05	0.01	1.0000	
3	1	0	631	60
1030	6.05	-0.01	1.0000	
3	1	0	497	73
857	6.05	-0.01	1.0000	
-5	-1	0	17867	1436

37015 -50.90 1.20 1.0000
-5 -1 0 16068 1412
34404 -50.90 1.20 1.0000
5 1 0 16752 1413
35400 -50.90 -1.20 1.0000
5 1 0 17181 1437
36022 -50.90 -1.20 1.0000
-7 -1 0 757 409
8737 57.18 0.07 1.0000
-7 -1 0 955 374
9382 57.18 0.07 1.0000
7 1 0 567 408
8058 57.18 -0.07 1.0000
7 1 0 976 376
9449 57.18 -0.07 1.0000
9 1 0 495 90
910 -9.40 1.81 1.0000
9 1 0 433 140
821 -9.40 1.81 1.0000
11 1 0 158 151
753 -12.88 -0.84 1.0000
13 1 0 2014 158
372 22.20 0.32 1.0000
15 1 0 229 280
2217 -27.71 -0.49 1.0000
17 1 0 272 202
187 9.99 2.06 1.0000
19 1 0 5 207
-234 1.96 -0.51 1.0000
21 1 0 18 230
-203 -3.66 0.18 1.0000
23 1 0 34 256
-110 5.04 0.02 1.0000
25 1 0 22 293
95 4.42 0.08 1.0000
26 -2 0 2 326
-256 1.32 -0.02 1.0000
24 -2 0 2 278
-44 -1.15 0.02 1.0000
22 -2 0 150 224
90 2.39 0.01 1.0000
20 -2 0 914 238
619 -4.63 -0.26 1.0000
18 -2 0 5 191

-87 -1.86 0.20 1.0000
16 -2 0 57 177
397 23.82 0.27 1.0000
14 -2 0 1600 155
475 15.79 -0.01 1.0000
14 -2 0 1557 110
452 15.79 -0.01 1.0000
12 -2 0 92 89
324 7.31 0.14 1.0000
12 -2 0 81 131
304 7.31 0.14 1.0000
10 -2 0 1088 220
3031 19.11 -1.39 1.0000
10 -2 0 1090 178
3035 19.11 -1.39 1.0000
8 -2 0 1479 140
1169 -9.74 5.98 1.0000
8 -2 0 1926 104
1594 -9.74 5.98 1.0000
-8 2 0 1418 132
1107 -9.74 -5.98 1.0000
6 -2 0 19664 349
8188 43.13 -1.73 1.0000
6 -2 0 19925 376
8357 43.13 -1.73 1.0000
-6 2 0 20101 373
8469 43.13 1.73 1.0000
4 -2 0 40567 2379
59450 -36.77 0.32 1.0000
4 -2 0 40467 2363
59330 -36.77 0.32 1.0000
-4 2 0 41951 2379
61125 -36.77 -0.32 1.0000
2 -2 0 177262 915
22478 235.00 0.30 1.0000
-2 2 0 179253 916
23191 235.00 -0.30 1.0000
-2 -2 0 183304 994
24646 235.05 0.68 1.0000
2 2 0 182737 997
24439 235.05 -0.68 1.0000
2 2 0 187339 989
26140 235.05 -0.68 1.0000
-4 -2 0 91988 2773

72251	29.91	-0.93	1.0000	
-4	-2	0	84831	2753
65925	29.91	-0.93	1.0000	
-4	-2	0	85664	2750
66659	29.91	-0.93	1.0000	
4	2	0	90441	2754
70880	29.91	0.93	1.0000	
4	2	0	93429	2775
73528	29.91	0.93	1.0000	
4	2	0	90214	2751
70679	29.91	0.93	1.0000	
-6	-2	0	15730	1651
42790	-70.60	-0.86	1.0000	
-6	-2	0	14886	1620
41391	-70.60	-0.86	1.0000	
-6	-2	0	13665	1618
39337	-70.60	-0.86	1.0000	
6	2	0	14650	1620
40999	-70.60	0.86	1.0000	
6	2	0	13997	1622
39902	-70.60	0.86	1.0000	
6	2	0	14701	1651
41084	-70.60	0.86	1.0000	
-8	-2	0	6985	166
1736	36.33	-0.21	1.0000	
-8	-2	0	7984	116
2251	36.33	-0.21	1.0000	
8	2	0	7254	172
1872	36.33	0.21	1.0000	
8	2	0	7817	120
2163	36.33	0.21	1.0000	
10	2	0	56	150
890	-32.35	-0.51	1.0000	
12	2	0	3116	443
8230	-30.25	0.70	1.0000	
14	2	0	5896	687
14202	36.75	0.15	1.0000	
16	2	0	240	217
852	-11.87	-0.29	1.0000	
18	2	0	107	216
221	-3.93	-0.30	1.0000	
20	2	0	282	244
428	3.39	-0.20	1.0000	
22	2	0	476	258

304 -3.79 0.02 1.0000
24 2 0 25 298
72 -3.01 0.15 1.0000
26 2 0 6 367
-339 -2.18 -0.15 1.0000
25 -3 0 22 288
-28 4.06 0.01 1.0000
23 -3 0 2 253
82 -6.64 -0.20 1.0000
21 -3 0 52 227
212 6.34 0.28 1.0000
19 -3 0 826 223
485 -5.82 -0.05 1.0000
17 -3 0 1682 212
1617 -0.69 0.07 1.0000
17 -3 0 2117 295
2044 -0.69 0.07 1.0000
15 -3 0 200 162
265 -26.35 -0.11 1.0000
15 -3 0 277 113
189 -26.35 -0.11 1.0000
13 -3 0 1089 132
78 -21.08 0.48 1.0000
13 -3 0 1358 99
162 -21.08 0.48 1.0000
11 -3 0 791 137
1488 -9.04 0.96 1.0000
11 -3 0 471 175
1033 -9.04 0.96 1.0000
9 -3 0 9097 313
5872 -16.26 -1.14 1.0000
7 -3 0 112 95
1219 21.11 -0.95 1.0000
7 -3 0 157 127
1360 21.11 -0.95 1.0000
-7 3 0 206 131
1498 21.11 0.95 1.0000
5 -3 0 492 80
436 1.13 0.38 1.0000
5 -3 0 673 58
607 1.13 0.38 1.0000
-5 3 0 480 79
425 1.13 -0.38 1.0000
3 -3 0 6068 1461

35986	-96.92	-0.07	1.0000	
-3	3	0	6541	1462
37125	-96.92	0.07	1.0000	
1	-3	0	638	79
1040	6.05	0.40	1.0000	
1	-3	0	647	63
1051	6.05	0.40	1.0000	
-1	3	0	689	80
1104	6.05	-0.40	1.0000	
-1	-3	0	78508	2703
65608	20.85	-0.10	1.0000	
1	3	0	88438	2712
74710	20.85	0.10	1.0000	
1	3	0	78675	2703
65760	20.85	0.10	1.0000	
-3	-3	0	7149	1656
38546	-96.90	0.00	1.0000	
3	3	0	12045	1675
49075	-96.90	0.00	1.0000	
3	3	0	7001	1656
38200	-96.90	0.00	1.0000	
-5	-3	0	61801	3038
79698	29.22	-0.12	1.0000	
-5	-3	0	54719	3006
71627	29.22	-0.12	1.0000	
-5	-3	0	57329	3014
74609	29.22	-0.12	1.0000	
5	3	0	60968	3038
78752	29.22	0.12	1.0000	
5	3	0	59520	3013
77105	29.22	0.12	1.0000	
5	3	0	59518	3008
77103	29.22	0.12	1.0000	
-7	-3	0	951	95
1577	7.70	-0.15	1.0000	
-7	-3	0	1274	122
1987	7.70	-0.15	1.0000	
-7	-3	0	1118	158
1791	7.70	-0.15	1.0000	
7	3	0	768	121
1339	7.70	0.15	1.0000	
7	3	0	1106	170
1775	7.70	0.15	1.0000	
7	3	0	934	99

1556	7.70	0.15	1.0000	
-9	-3	0	8619	279
5939	13.67	0.34	1.0000	
9	3	0	9631	348
6784	13.67	-0.34	1.0000	
9	3	0	9409	287
6598	13.67	-0.34	1.0000	
11	3	0	3487	133
191	39.21	0.53	1.0000	
13	3	0	2376	191
1015	-14.64	0.09	1.0000	
15	3	0	2152	282
1959	1.86	1.00	1.0000	
17	3	0	230	197
86	5.21	-0.75	1.0000	
19	3	0	141	234
329	-5.37	0.87	1.0000	
21	3	0	449	254
141	8.12	0.32	1.0000	
23	3	0	623	326
578	0.80	-0.12	1.0000	
25	3	0	453	357
465	-0.24	-0.01	1.0000	
26	-4	0	94	325
116	-0.91	0.29	1.0000	
24	-4	0	176	276
263	2.55	-0.06	1.0000	
22	-4	0	70	231
-40	-7.24	-0.12	1.0000	
20	-4	0	434	251
771	6.01	0.17	1.0000	
20	-4	0	812	198
1255	6.01	0.17	1.0000	
18	-4	0	1604	253
2400	7.75	0.11	1.0000	
18	-4	0	1614	330
2412	7.75	0.11	1.0000	
16	-4	0	13	117
-40	-3.12	-0.16	1.0000	
16	-4	0	92	167
36	-3.12	-0.16	1.0000	
14	-4	0	1	181
1668	34.77	0.28	1.0000	
14	-4	0	4	235

1768 34.77 0.28 1.0000
12 -4 0 1099 131
79 36.44 -0.01 1.0000
10 -4 0 8698 229
3159 -32.15 1.98 1.0000
8 -4 0 5280 823
19258 57.31 -1.52 1.0000
6 -4 0 506 433
9457 -64.77 1.99 1.0000
6 -4 0 584 404
9783 -64.77 1.99 1.0000
-6 4 0 549 431
9639 -64.77 -1.99 1.0000
4 -4 0 9042 1151
28045 62.74 0.45 1.0000
-4 4 0 9363 1152
28608 62.74 -0.45 1.0000
2 -4 0 86940 2720
67733 29.99 0.26 1.0000
2 -4 0 85593 2707
66545 29.99 0.26 1.0000
-2 4 0 91292 2723
71581 29.99 -0.26 1.0000
-2 -4 0 61805 3157
84703 -36.78 -0.10 1.0000
-2 -4 0 55740 3145
77577 -36.78 -0.10 1.0000
2 4 0 55401 3145
77176 -36.78 0.10 1.0000
-4 -4 0 9122 1149
28196 62.77 0.63 1.0000
4 4 0 9751 1148
29294 62.77 -0.63 1.0000
-8 -4 0 175 186
3943 -42.95 -0.68 1.0000
-8 -4 0 190 207
4011 -42.95 -0.68 1.0000
8 4 0 231 203
4192 -42.95 0.68 1.0000
8 4 0 212 191
4111 -42.95 0.68 1.0000
8 4 0 168 256
3907 -42.95 0.68 1.0000
-10 -4 0 71 86

483 -26.35 -0.62 1.0000
10 4 0 66 91
498 -26.35 0.62 1.0000
10 4 0 58 91
521 -26.35 0.62 1.0000
10 4 0 85 154
449 -26.35 0.62 1.0000
12 4 0 1852 221
1480 3.95 -0.10 1.0000
14 4 0 1055 172
-142 -28.16 -0.31 1.0000
16 4 0 20 210
268 18.05 -0.11 1.0000
18 4 0 40 232
49 -0.59 0.29 1.0000
20 4 0 198 268
415 -5.45 -0.23 1.0000
22 4 0 407 290
516 -2.20 -0.09 1.0000
24 4 0 23 310
88 -3.97 -0.10 1.0000
26 4 0 193 367
224 -0.92 0.13 1.0000
25 -5 0 15 294
-57 -3.27 -0.52 1.0000
23 -5 0 320 293
891 10.36 0.16 1.0000
21 -5 0 362 169
62 -9.68 -0.38 1.0000
21 -5 0 125 232
-50 -9.68 -0.38 1.0000
19 -5 0 2 158
-85 -1.11 -0.09 1.0000
19 -5 0 2 200
-76 -1.11 -0.09 1.0000
17 -5 0 85 140
152 -2.69 0.33 1.0000
17 -5 0 170 193
260 -2.69 0.33 1.0000
15 -5 0 1709 114
159 -24.92 0.32 1.0000
15 -5 0 1706 161
158 -24.92 0.32 1.0000
13 -5 0 872 215

1788 -11.05 -0.18 1.0000
11 -5 0 2100 284
4290 -17.04 -0.74 1.0000
9 -5 0 6394 280
4759 -9.52 0.18 1.0000
7 -5 0 4509 303
6638 12.40 0.97 1.0000
7 -5 0 4972 344
7197 12.40 0.97 1.0000
5 -5 0 880 113
706 -2.70 -0.83 1.0000
-5 5 0 974 115
791 -2.70 0.83 1.0000
3 -5 0 54657 2932
71575 29.25 0.02 1.0000
3 -5 0 57022 2950
74278 29.25 0.02 1.0000
-3 5 0 59311 2954
76887 29.25 -0.02 1.0000
1 -5 0 16620 1382
35225 -50.94 -0.70 1.0000
1 -5 0 16684 1401
35317 -50.94 -0.70 1.0000
-1 5 0 15356 1380
33374 -50.94 0.70 1.0000
-1 -5 0 15829 607
14773 3.70 1.26 1.0000
1 5 0 16799 621
15712 3.70 -1.26 1.0000
1 5 0 15035 606
14008 3.70 -1.26 1.0000
-3 -5 0 421 77
372 1.07 0.23 1.0000
-3 -5 0 553 102
496 1.07 0.23 1.0000
3 5 0 651 78
590 1.07 -0.23 1.0000
3 5 0 463 105
412 1.07 -0.23 1.0000
-5 -5 0 643 97
496 -2.71 -1.01 1.0000
-5 -5 0 900 112
724 -2.71 -1.01 1.0000
5 5 0 704 88

549 -2.71 1.01 1.0000
5 5 0 1034 136
845 -2.71 1.01 1.0000
-7 -5 0 2600 90
481 25.20 -0.41 1.0000
7 5 0 2516 76
445 25.20 0.41 1.0000
7 5 0 2138 117
295 25.20 0.41 1.0000
-9 -5 0 3 131
698 24.22 0.19 1.0000
-9 -5 0 31 87
1121 24.22 0.19 1.0000
9 5 0 5 118
895 24.22 -0.19 1.0000
9 5 0 1 155
832 24.22 -0.19 1.0000
9 5 0 39 98
1167 24.22 -0.19 1.0000
-11 -5 0 42 126
616 15.91 0.30 1.0000
11 5 0 65 172
697 15.91 -0.30 1.0000
11 5 0 52 131
653 15.91 -0.30 1.0000
13 5 0 5632 395
6219 3.30 0.35 1.0000
15 5 0 17 205
401 13.78 0.07 1.0000
17 5 0 525 228
72 -12.76 0.42 1.0000
19 5 0 2609 381
2929 2.64 -0.16 1.0000
21 5 0 82 268
100 0.84 0.23 1.0000
23 5 0 0 281
-121 -0.39 0.15 1.0000
25 5 0 431 342
549 2.31 0.00 1.0000
26 -6 0 2 302
-137 1.27 -0.13 1.0000
24 -6 0 31 289
113 -4.35 0.04 1.0000
22 -6 0 10 258

145	7.71	0.31	1.0000	
22	-6	0	3	202
110	7.71	0.31	1.0000	
20	-6	0	802	261
1534	-9.41	-0.05	1.0000	
20	-6	0	705	278
1399	-9.41	-0.05	1.0000	
18	-6	0	3660	353
3202	3.39	-0.08	1.0000	
18	-6	0	3941	293
3465	3.39	-0.08	1.0000	
16	-6	0	1000	133
-93	27.42	0.02	1.0000	
16	-6	0	1397	171
33	27.42	0.02	1.0000	
14	-6	0	40	146
44	-0.29	-0.10	1.0000	
12	-6	0	4100	267
3455	4.55	0.57	1.0000	
10	-6	0	40	250
3541	57.05	0.52	1.0000	
8	-6	0	14904	172
1762	-69.47	-0.03	1.0000	
6	-6	0	318	80
827	40.39	-0.08	1.0000	
6	-6	0	399	123
708	40.39	-0.08	1.0000	
-6	6	0	611	107
479	40.39	0.08	1.0000	
2	-6	0	14737	1669
41135	-70.58	0.55	1.0000	
2	-6	0	14132	1647
40120	-70.58	0.55	1.0000	
-2	6	0	15656	1675
42660	-70.58	-0.55	1.0000	
0	-6	0	39948	2746
71052	57.80	1.99	1.0000	
0	-6	0	40916	2758
72341	57.80	1.99	1.0000	
0	6	0	35190	2742
64652	57.80	-1.99	1.0000	
-2	-6	0	21310	411
9291	43.00	-1.60	1.0000	
-2	-6	0	23030	433

10438 43.00 -1.60 1.0000
2 6 0 21354 411
9318 43.00 1.60 1.0000
2 6 0 21877 428
9665 43.00 1.60 1.0000
-4 -6 0 1023 514
11370 -64.67 2.34 1.0000
4 6 0 1245 516
12085 -64.67 -2.34 1.0000
4 6 0 1746 545
13556 -64.67 -2.34 1.0000
-6 -6 0 672 115
428 40.30 -0.40 1.0000
-6 -6 0 612 84
479 40.30 -0.40 1.0000
6 6 0 467 130
635 40.30 0.40 1.0000
6 6 0 570 95
523 40.30 0.40 1.0000
-8 -6 0 15814 1505
34544 -52.11 0.51 1.0000
-8 -6 0 17638 1527
37216 -52.11 0.51 1.0000
8 6 0 18637 1510
38663 -52.11 -0.51 1.0000
8 6 0 18542 1567
38526 -52.11 -0.51 1.0000
8 6 0 20330 1526
41085 -52.11 -0.51 1.0000
-10 -6 0 125 160
921 -16.60 0.01 1.0000
-10 -6 0 294 98
1318 -16.60 0.01 1.0000
10 6 0 195 135
1097 -16.60 -0.01 1.0000
10 6 0 443 119
1616 -16.60 -0.01 1.0000
10 6 0 298 202
1326 -16.60 -0.01 1.0000
-12 -6 0 389 98
-18 -17.09 0.01 1.0000
12 6 0 987 153
137 -17.09 -0.01 1.0000
12 6 0 1132 96

194 -17.09 -0.01 1.0000
-14 -6 0 13 107
127 6.59 -0.24 1.0000
14 6 0 86 193
284 6.59 0.24 1.0000
16 6 0 1066 317
1849 -8.98 0.01 1.0000
18 6 0 1345 351
2367 10.38 -0.19 1.0000
20 6 0 1496 310
1484 -0.13 0.10 1.0000
22 6 0 34 257
1 -5.90 0.05 1.0000
24 6 0 266 311
286 0.52 -0.07 1.0000
26 6 0 14 327
-267 -3.27 -0.07 1.0000
25 -7 0 293 307
179 3.23 0.19 1.0000
23 -7 0 26 295
361 -12.07 -0.75 1.0000
23 -7 0 80 218
525 -12.07 -0.75 1.0000
21 -7 0 509 192
71 12.25 0.09 1.0000
21 -7 0 200 218
-84 12.25 0.09 1.0000
19 -7 0 2186 269
2149 0.35 -0.02 1.0000
19 -7 0 1461 277
1430 0.35 -0.02 1.0000
17 -7 0 6050 555
11809 -26.78 -0.19 1.0000
17 -7 0 5274 621
10714 -26.78 -0.19 1.0000
15 -7 0 105 201
845 16.33 -0.05 1.0000
13 -7 0 2435 364
5704 -22.69 0.08 1.0000
11 -7 0 5305 460
8766 -18.02 -0.35 1.0000
9 -7 0 3394 131
599 29.35 0.43 1.0000
7 -7 0 1953 254

4123	-17.35	-0.40	1.0000	
5	-7	0	2331	105
370	25.18	0.38	1.0000	
5	-7	0	2471	77
427	25.18	0.38	1.0000	
3	-7	0	640	141
1167	7.69	0.12	1.0000	
3	-7	0	1002	115
1642	7.69	0.12	1.0000	
-3	7	0	532	156
1020	7.69	-0.12	1.0000	
1	-7	0	1005	434
9531	57.15	0.13	1.0000	
1	-7	0	922	404
9273	57.15	0.13	1.0000	
1	-7	0	981	424
9457	57.15	0.13	1.0000	
-1	7	0	748	400
8700	57.15	-0.13	1.0000	
-1	-7	0	1133	398
8884	-52.53	-0.75	1.0000	
-1	-7	0	1131	383
8879	-52.53	-0.75	1.0000	
1	7	0	996	379
8494	-52.53	0.75	1.0000	
1	7	0	995	398
8489	-52.53	0.75	1.0000	
-3	-7	0	202	119
1484	21.06	-1.29	1.0000	
3	7	0	386	123
1934	21.06	1.29	1.0000	
-5	-7	0	5896	396
8308	12.44	0.86	1.0000	
5	7	0	6244	397
8718	12.44	-0.86	1.0000	
5	7	0	7591	435
10298	12.44	-0.86	1.0000	
-7	-7	0	2214	241
4497	-17.34	0.71	1.0000	
7	7	0	2170	234
4435	-17.34	-0.71	1.0000	
7	7	0	2470	298
4859	-17.34	-0.71	1.0000	
-9	-7	0	7512	132

861 49.72 -0.44 1.0000
-9 -7 0 7393 82
821 49.72 -0.44 1.0000
9 7 0 6872 169
652 49.72 0.44 1.0000
9 7 0 7371 96
812 49.72 0.44 1.0000
9 7 0 7076 126
716 49.72 0.44 1.0000
11 7 0 1052 182
559 -7.62 0.15 1.0000
11 7 0 1331 117
767 -7.62 0.15 1.0000
11 7 0 1414 121
830 -7.62 0.15 1.0000
13 7 0 1789 271
4764 23.17 0.12 1.0000
13 7 0 1699 351
4616 23.17 0.12 1.0000
15 7 0 4373 420
5825 -8.83 -0.28 1.0000
17 7 0 1232 295
1758 -5.92 0.10 1.0000
19 7 0 963 283
880 -1.19 0.43 1.0000
21 7 0 799 289
553 -4.11 -0.12 1.0000
23 7 0 165 289
152 0.46 0.09 1.0000
25 7 0 103 329
51 -2.60 -0.03 1.0000
24 -8 0 529 337
886 5.86 0.33 1.0000
24 -8 0 766 284
1186 5.86 0.33 1.0000
22 -8 0 67 251
-68 -7.08 -0.32 1.0000
22 -8 0 521 229
215 -7.08 -0.32 1.0000
20 -8 0 0 175
13 3.14 -0.10 1.0000
20 -8 0 13 216
-98 3.14 -0.10 1.0000
18 -8 0 276 200

109	5.35	0.15	1.0000	
18	-8	0	38	159
-60	5.35	0.15	1.0000	
16	-8	0	4816	350
3837	-6.46	0.06	1.0000	
14	-8	0	28	155
356	20.94	0.18	1.0000	
12	-8	0	44	147
295	20.62	0.02	1.0000	
10	-8	0	8	204
1707	-38.33	-0.41	1.0000	
8	-8	0	125	241
3384	40.75	0.47	1.0000	
6	-8	0	18265	1567
38099	-52.05	0.10	1.0000	
4	-8	0	185	242
3982	-42.90	0.17	1.0000	
2	-8	0	7630	179
2066	36.32	0.26	1.0000	
2	-8	0	7683	146
2094	36.32	0.26	1.0000	
0	-8	0	19729	333
6628	-51.19	-1.73	1.0000	
0	-8	0	20096	312
6842	-51.19	-1.73	1.0000	
0	8	0	19841	311
6695	-51.19	1.73	1.0000	
-2	-8	0	1752	120
1463	-9.45	6.29	1.0000	
-2	-8	0	1571	162
1290	-9.45	6.29	1.0000	
2	8	0	1357	155
1080	-9.45	-6.29	1.0000	
2	8	0	1898	122
1598	-9.45	-6.29	1.0000	
-4	-8	0	6554	898
21567	57.13	-1.52	1.0000	
4	8	0	6832	897
22074	57.13	1.52	1.0000	
-6	-8	0	16697	165
2419	-69.52	0.44	1.0000	
6	8	0	16638	164
2380	-69.52	-0.44	1.0000	
6	8	0	16498	210

2327	-69.52	-0.44	1.0000	
-8	-8	0	64	214
3038	40.85	0.23	1.0000	
8	8	0	68	199
3064	40.85	-0.23	1.0000	
8	8	0	185	267
3687	40.85	-0.23	1.0000	
-10	-8	0	3760	84
689	-30.40	-0.32	1.0000	
-10	-8	0	2940	134
367	-30.40	-0.32	1.0000	
10	8	0	2510	167
226	-30.40	0.32	1.0000	
10	8	0	3581	115
614	-30.40	0.32	1.0000	
10	8	0	3620	91
630	-30.40	0.32	1.0000	
12	8	0	240	105
-41	-13.43	-0.29	1.0000	
12	8	0	240	167
-228	-13.43	-0.29	1.0000	
12	8	0	490	86
44	-13.43	-0.29	1.0000	
14	8	0	903	242
1025	1.71	-0.01	1.0000	
14	8	0	1132	166
1269	1.71	-0.01	1.0000	
16	8	0	1073	231
463	9.74	0.27	1.0000	
18	8	0	784	254
588	3.24	0.05	1.0000	
20	8	0	569	274
585	0.30	-0.10	1.0000	
22	8	0	15	269
-50	3.32	0.00	1.0000	
24	8	0	20	302
-79	-3.89	0.03	1.0000	
25	-9	0	13	323
-102	-3.18	-0.01	1.0000	
23	-9	0	369	258
635	5.19	0.30	1.0000	
23	-9	0	283	291
520	5.19	0.30	1.0000	
21	-9	0	35	234

-161 -5.13 -0.34 1.0000
21 -9 0 0 229
29 -5.13 -0.34 1.0000
19 -9 0 15 171
54 -3.05 -0.11 1.0000
19 -9 0 12 213
-40 -3.05 -0.11 1.0000
17 -9 0 1198 343
3287 19.69 -0.24 1.0000
15 -9 0 181 185
295 -26.54 -0.16 1.0000
13 -9 0 627 159
410 -4.16 -0.07 1.0000
11 -9 0 2676 386
6578 25.46 0.54 1.0000
9 -9 0 1270 144
418 -38.38 -0.82 1.0000
7 -9 0 7721 152
928 49.76 0.33 1.0000
5 -9 0 4 144
895 24.25 -0.06 1.0000
3 -9 0 8684 327
6006 13.60 -0.19 1.0000
1 -9 0 475 147
920 -9.39 1.42 1.0000
1 9 0 617 217
3056 26.38 1.47 1.0000
1 9 0 729 187
3298 26.38 1.47 1.0000
3 9 0 10120 347
6683 -16.34 0.84 1.0000
3 9 0 10019 315
6601 -16.34 0.84 1.0000
5 9 0 7075 277
5345 -9.54 -0.41 1.0000
5 9 0 7761 329
5944 -9.54 -0.41 1.0000
7 9 0 3825 165
793 29.32 0.58 1.0000
7 9 0 3394 107
603 29.32 0.58 1.0000
9 9 0 1407 169
459 -38.45 -1.11 1.0000
9 9 0 1415 108

401 -38.45 -1.11 1.0000
11 9 0 3418 447
10402 37.73 0.23 1.0000
11 9 0 2490 507
8729 37.73 0.23 1.0000
11 9 0 2746 414
9203 37.73 0.23 1.0000
13 9 0 1390 153
921 -6.01 0.02 1.0000
13 9 0 1389 236
920 -6.01 0.02 1.0000
15 9 0 1418 339
2110 -7.17 0.19 1.0000
17 9 0 678 182
493 3.33 0.13 1.0000
17 9 0 291 248
175 3.33 0.13 1.0000
19 9 0 106 273
25 -13.25 -0.17 1.0000
21 9 0 92 286
249 -5.36 0.24 1.0000
23 9 0 0 326
-7 0.23 -0.02 1.0000
25 9 0 12 348
-178 -3.01 0.03 1.0000
24 -10 0 3 296
-26 -0.75 -1.26 1.0000
24 -10 0 129 292
169 -0.75 -1.26 1.0000
22 -10 0 1 233
-243 0.83 0.20 1.0000
22 -10 0 141 262
119 0.83 0.20 1.0000
20 -10 0 606 257
564 0.76 -0.01 1.0000
20 -10 0 795 229
747 0.76 -0.01 1.0000
18 -10 0 21 196
-149 -3.95 -0.01 1.0000
16 -10 0 1547 283
1629 0.89 -0.10 1.0000
14 -10 0 3752 289
2424 10.42 -0.06 1.0000
12 -10 0 245 160

439 -4.57 -0.39 1.0000
10 -10 0 11377 758
16313 18.25 0.42 1.0000
8 -10 0 3113 145
433 -30.33 0.22 1.0000
6 -10 0 247 179
1232 -16.78 -0.50 1.0000
4 -10 0 26 144
638 -26.29 0.43 1.0000
2 -10 0 125 145
687 -32.41 -0.20 1.0000
0 -10 0 2806 188
1105 18.04 2.44 1.0000
0 10 0 2369 125
872 18.04 -2.44 1.0000
2 10 0 872 223
2647 18.99 1.66 1.0000
2 10 0 1054 185
2958 18.99 1.66 1.0000
4 10 0 9436 213
3618 -32.09 -2.30 1.0000
4 10 0 9454 257
3629 -32.09 -2.30 1.0000
6 10 0 12 229
3894 57.05 -0.86 1.0000
8 10 0 33 252
2499 -38.38 0.39 1.0000
8 10 0 0 179
1932 -38.38 0.39 1.0000
10 10 0 12203 724
17299 18.25 -0.01 1.0000
10 10 0 11202 774
16103 18.25 -0.01 1.0000
12 10 0 123 111
331 6.15 0.30 1.0000
12 10 0 147 207
369 6.15 0.30 1.0000
12 10 0 126 136
336 6.15 0.30 1.0000
14 10 0 199 253
519 -7.51 0.06 1.0000
14 10 0 327 172
715 -7.51 0.06 1.0000
16 10 0 117 269

559 11.11 -0.20 1.0000
16 10 0 221 195
766 11.11 -0.20 1.0000
18 10 0 189 205
211 -0.68 0.12 1.0000
18 10 0 160 257
181 -0.68 0.12 1.0000
20 10 0 38 289
318 10.10 0.22 1.0000
22 10 0 215 319
211 0.13 0.02 1.0000
24 10 0 153 350
131 0.82 -0.19 1.0000
23 -11 0 21 279
-23 3.97 0.38 1.0000
23 -11 0 21 261
-391 3.97 0.38 1.0000
21 -11 0 20 238
24 -0.38 -0.11 1.0000
21 -11 0 389 236
406 -0.38 -0.11 1.0000
19 -11 0 3 206
-155 -1.26 -0.65 1.0000
17 -11 0 1892 331
2535 5.93 0.30 1.0000
15 -11 0 256 188
259 -0.08 -0.13 1.0000
13 -11 0 888 188
680 3.23 0.09 1.0000
11 -11 0 1758 155
299 -21.36 -0.34 1.0000
9 -11 0 2935 502
9535 37.68 0.21 1.0000
7 -11 0 1132 157
623 -7.53 -0.26 1.0000
5 -11 0 139 168
907 15.90 0.34 1.0000
3 -11 0 3402 137
171 39.23 0.41 1.0000
1 -11 0 252 174
949 -12.85 -1.49 1.0000
1 11 0 1651 147
642 -13.28 -0.47 1.0000
1 11 0 1583 123

600 -13.28 -0.47 1.0000
3 11 0 809 208
1516 -9.07 -1.11 1.0000
3 11 0 811 151
1519 -9.07 -1.11 1.0000
5 11 0 2394 267
4717 -17.11 0.89 1.0000
5 11 0 2666 317
5096 -17.11 0.89 1.0000
7 11 0 5980 449
9638 -18.07 -0.23 1.0000
9 11 0 2926 361
6971 25.49 0.25 1.0000
9 11 0 3121 428
7270 25.49 0.25 1.0000
11 11 0 904 156
29 -21.39 -0.34 1.0000
11 11 0 1167 124
90 -21.39 -0.34 1.0000
13 11 0 203 214
177 -0.82 -0.03 1.0000
13 11 0 184 149
159 -0.82 -0.03 1.0000
15 11 0 15 232
-22 3.30 0.24 1.0000
15 11 0 208 168
128 3.30 0.24 1.0000
17 11 0 29 197
380 -12.25 0.08 1.0000
17 11 0 1 266
176 -12.25 0.08 1.0000
19 11 0 134 291
71 -2.74 0.11 1.0000
19 11 0 492 219
362 -2.74 0.11 1.0000
21 11 0 192 304
317 -3.43 -0.07 1.0000
23 11 0 93 352
329 -7.35 0.06 1.0000
22 -12 0 3 251
-214 1.00 -1.22 1.0000
22 -12 0 217 262
274 1.00 -1.22 1.0000
20 -12 0 10 230

-127	2.71	0.46	1.0000	
18	-12	0	498	208
127	-9.59	-0.45	1.0000	
16	-12	0	505	249
1026	8.28	0.00	1.0000	
14	-12	0	855	215
887	-0.47	-0.15	1.0000	
12	-12	0	877	199
823	0.81	0.27	1.0000	
10	-12	0	121	166
330	6.20	-0.11	1.0000	
8	-12	0	241	135
-111	-13.47	-0.19	1.0000	
6	-12	0	1330	133
283	-17.07	0.52	1.0000	
4	-12	0	1510	195
1179	3.92	-0.65	1.0000	
2	-12	0	3574	475
8975	-30.30	0.78	1.0000	
0	-12	0	47	185
748	17.78	0.81	1.0000	
0	12	0	16	110
599	17.78	-0.81	1.0000	
2	12	0	26	135
182	7.27	-0.32	1.0000	
2	12	0	40	94
217	7.27	-0.32	1.0000	
4	12	0	1768	140
-16	36.45	0.27	1.0000	
4	12	0	889	93
150	36.45	0.27	1.0000	
6	12	0	4568	244
3891	4.53	-0.54	1.0000	
6	12	0	4787	292
4093	4.53	-0.54	1.0000	
8	12	0	87	188
208	20.57	-0.14	1.0000	
8	12	0	12	116
413	20.57	-0.14	1.0000	
10	12	0	179	192
348	-4.58	0.08	1.0000	
10	12	0	273	132
475	-4.58	0.08	1.0000	
12	12	0	1124	170

1061 0.82 0.13 1.0000
12 12 0 588 212
543 0.82 0.13 1.0000
14 12 0 1518 217
364 17.23 0.12 1.0000
14 12 0 1518 180
364 17.23 0.12 1.0000
16 12 0 0 189
66 -6.62 0.04 1.0000
16 12 0 6 256
101 -6.62 0.04 1.0000
18 12 0 662 251
1551 11.84 0.03 1.0000
18 12 0 281 322
925 11.84 0.03 1.0000
20 12 0 6 296
-251 2.18 0.09 1.0000
20 12 0 321 246
237 2.18 0.09 1.0000
22 12 0 2 329
-203 1.18 -0.03 1.0000
23 -13 0 225 317
383 -3.97 -0.11 1.0000
23 -13 0 83 278
187 -3.97 -0.11 1.0000
21 -13 0 8 250
-76 2.40 0.22 1.0000
19 -13 0 512 308
1588 14.94 0.49 1.0000
17 -13 0 37 203
180 -16.45 -0.60 1.0000
15 -13 0 3 183
228 11.65 0.09 1.0000
13 -13 0 1517 279
1712 -2.10 0.00 1.0000
11 -13 0 72 152
57 -0.80 -0.28 1.0000
9 -13 0 963 173
580 -6.03 0.21 1.0000
7 -13 0 1542 328
4354 23.16 -0.01 1.0000
5 -13 0 5405 371
5996 3.40 -0.27 1.0000
3 -13 0 2403 191

1033	-14.63	-0.01	1.0000	
1	-13	0	2049	169
387	22.21	0.33	1.0000	
1	13	0	171	173
1827	-25.73	0.21	1.0000	
3	13	0	588	143
-51	-21.02	-0.09	1.0000	
3	13	0	588	89
-50	-21.02	-0.09	1.0000	
5	13	0	792	231
1679	-11.13	-0.10	1.0000	
5	13	0	845	179
1756	-11.13	-0.10	1.0000	
7	13	0	3316	405
7019	-22.71	-0.09	1.0000	
7	13	0	2604	342
5964	-22.71	-0.09	1.0000	
9	13	0	694	138
461	-4.22	-0.03	1.0000	
9	13	0	646	205
422	-4.22	-0.03	1.0000	
11	13	0	787	157
593	3.22	0.19	1.0000	
11	13	0	482	215
333	3.22	0.19	1.0000	
13	13	0	1437	245
1626	-2.10	0.11	1.0000	
13	13	0	1187	280
1359	-2.10	0.11	1.0000	
15	13	0	394	199
447	-1.13	0.00	1.0000	
15	13	0	296	213
343	-1.13	0.00	1.0000	
17	13	0	198	251
57	-5.66	-0.02	1.0000	
17	13	0	726	206
417	-5.66	-0.02	1.0000	
19	13	0	10	282
111	-6.36	0.05	1.0000	
19	13	0	7	228
99	-6.36	0.05	1.0000	
21	13	0	11	337
36	-2.39	0.06	1.0000	
23	13	0	260	381

378 2.87 0.24 1.0000
22 -14 0 721 325
763 -0.66 0.52 1.0000
20 -14 0 778 257
420 -9.55 -1.78 1.0000
18 -14 0 152 235
478 8.25 0.31 1.0000
16 -14 0 153 203
110 -1.62 0.09 1.0000
14 -14 0 2 178
94 -7.26 -0.13 1.0000
12 -14 0 1204 190
219 17.26 0.04 1.0000
10 -14 0 324 200
711 -7.50 0.13 1.0000
8 -14 0 921 203
1046 1.73 -0.05 1.0000
6 -14 0 85 170
285 6.63 0.04 1.0000
4 -14 0 1648 155
155 -28.41 0.89 1.0000
2 -14 0 5574 677
13733 36.87 -0.30 1.0000
0 -14 0 1277 180
617 -9.45 -0.43 1.0000
2 14 0 1084 153
221 15.68 -0.51 1.0000
2 14 0 1262 108
305 15.68 -0.51 1.0000
4 14 0 6 231
1410 34.74 -0.38 1.0000
4 14 0 16 171
1304 34.74 -0.38 1.0000
6 14 0 56 112
61 -0.31 0.13 1.0000
6 14 0 145 173
154 -0.31 0.13 1.0000
8 14 0 72 179
248 20.98 0.01 1.0000
8 14 0 1 142
537 20.98 0.01 1.0000
10 14 0 3895 247
2540 10.41 0.00 1.0000
10 14 0 3959 322

2592 10.41 0.00 1.0000
12 14 0 679 249
711 -0.53 -0.11 1.0000
12 14 0 950 189
988 -0.53 -0.11 1.0000
14 14 0 71 232
-7 -7.29 -0.05 1.0000
14 14 0 83 184
306 -7.29 -0.05 1.0000
16 14 0 5 253
50 8.09 0.20 1.0000
16 14 0 17 199
181 8.09 0.20 1.0000
18 14 0 205 244
374 4.35 0.15 1.0000
18 14 0 25 223
-86 4.35 0.15 1.0000
20 14 0 1 266
-155 -0.71 -0.03 1.0000
20 14 0 396 278
364 -0.71 -0.03 1.0000
22 14 0 214 339
360 3.77 -0.10 1.0000
21 -15 0 563 283
383 3.60 0.25 1.0000
19 -15 0 548 240
321 -4.77 0.28 1.0000
17 -15 0 41 242
486 13.53 -0.04 1.0000
15 -15 0 637 255
1256 -8.85 -0.15 1.0000
13 -15 0 219 194
258 -1.09 -0.03 1.0000
11 -15 0 374 182
267 3.27 0.26 1.0000
9 -15 0 1502 291
2207 -7.12 -0.20 1.0000
7 -15 0 4965 429
6516 -8.89 0.06 1.0000
5 -15 0 8 167
175 13.87 -0.27 1.0000
3 -15 0 2014 276
1790 2.23 -0.22 1.0000
1 -15 0 249 293

2305 -27.94 0.17 1.0000
-1 -15 0 19 207
253 9.96 0.33 1.0000
3 15 0 435 156
93 -26.43 0.11 1.0000
3 15 0 386 114
118 -26.43 0.11 1.0000
5 15 0 1549 117
113 -24.90 -0.05 1.0000
5 15 0 1372 174
69 -24.90 -0.05 1.0000
7 15 0 75 162
753 16.27 0.02 1.0000
7 15 0 135 212
924 16.27 0.02 1.0000
9 15 0 182 159
295 -26.58 0.13 1.0000
9 15 0 87 204
456 -26.58 0.13 1.0000
11 15 0 116 179
118 -0.09 0.21 1.0000
11 15 0 0 220
-80 -0.09 0.21 1.0000
13 15 0 180 203
-8 11.62 0.09 1.0000
13 15 0 28 205
66 11.62 0.09 1.0000
15 15 0 629 305
1244 -8.84 -0.04 1.0000
15 15 0 964 310
1701 -8.84 -0.04 1.0000
17 15 0 38 273
-22 5.34 0.04 1.0000
17 15 0 38 244
-150 5.34 0.04 1.0000
19 15 0 380 287
390 -0.21 0.06 1.0000
19 15 0 0 264
-68 -0.21 0.06 1.0000
21 15 0 6 287
6 0.02 0.15 1.0000
21 15 0 352 319
351 0.02 0.15 1.0000
20 -16 0 61 258

102 1.97 0.34 1.0000
18 -16 0 176 255
366 -5.09 -0.52 1.0000
16 -16 0 353 209
376 0.52 -0.26 1.0000
14 -16 0 0 190
85 8.06 0.17 1.0000
12 -16 0 27 182
164 -6.58 0.03 1.0000
10 -16 0 210 214
745 11.10 0.02 1.0000
8 -16 0 1240 203
571 9.81 0.19 1.0000
6 -16 0 930 287
1674 -9.03 0.25 1.0000
4 -16 0 55 170
182 18.10 -0.32 1.0000
2 -16 0 365 235
1093 -12.09 0.31 1.0000
0 -16 0 2047 228
1003 11.77 0.19 1.0000
4 16 0 215 174
123 -3.13 0.14 1.0000
4 16 0 167 131
88 -3.13 0.14 1.0000
6 16 0 1944 211
156 27.40 0.16 1.0000
6 16 0 1249 138
14 27.40 0.16 1.0000
8 16 0 4849 369
3866 -6.47 -0.20 1.0000
8 16 0 5450 323
4404 -6.47 -0.20 1.0000
10 16 0 1787 274
1868 0.83 -0.30 1.0000
10 16 0 1774 315
1855 0.83 -0.30 1.0000
12 16 0 643 232
1222 8.32 0.14 1.0000
12 16 0 414 277
897 8.32 0.14 1.0000
14 16 0 586 280
497 -1.66 -0.08 1.0000
14 16 0 341 241

274 -1.66 -0.08 1.0000
16 16 0 210 257
225 0.44 -0.04 1.0000
16 16 0 820 265
850 0.44 -0.04 1.0000
18 16 0 554 309
918 -5.87 -0.11 1.0000
18 16 0 31 302
153 -5.87 -0.11 1.0000
20 16 0 15 324
-111 3.40 -0.06 1.0000
20 16 0 266 324
410 3.40 -0.06 1.0000
19 -17 0 15 249
-36 3.36 -0.38 1.0000
17 -17 0 19 228
3 -2.36 0.41 1.0000
15 -17 0 38 196
-200 5.34 0.17 1.0000
13 -17 0 76 205
5 -5.64 -0.10 1.0000
11 -17 0 2 207
160 -12.24 0.01 1.0000
9 -17 0 481 210
327 3.33 0.07 1.0000
7 -17 0 964 255
1429 -5.85 -0.25 1.0000
5 -17 0 213 189
-11 -12.66 0.15 1.0000
3 -17 0 64 193
5 4.98 0.22 1.0000
1 -17 0 63 207
144 10.81 -0.33 1.0000
-1 -17 0 75 284
1358 -24.45 -0.07 1.0000
5 17 0 219 223
319 -2.65 -0.33 1.0000
5 17 0 382 167
511 -2.65 -0.33 1.0000
7 17 0 6520 674
12475 -26.82 -0.23 1.0000
7 17 0 6138 604
11944 -26.82 -0.23 1.0000
9 17 0 1150 349

```
3204    19.67     0.37  1.0000
   9  17    0     1076      313
3081    19.67     0.37  1.0000
  11  17    0     1693      319
2307     5.97     0.17  1.0000
  11  17    0     2019      334
2685     5.97     0.17  1.0000
  13  17    0       97      257
86  -16.51   -0.34  1.0000
  13  17    0      363      229
-277  -16.51    -0.34  1.0000
  15  17    0      383      297
1238    13.53     0.13  1.0000
  15  17    0      228      313
944    13.53     0.13  1.0000
  17  17    0      310      304
224    -2.31     0.16  1.0000
  17  17    0      409      277
310    -2.31     0.16  1.0000
  19  17    0      290      319
461     3.84     0.18  1.0000
  19  17    0      825      346
1099     3.84     0.18  1.0000
  18 -18    0        4      245
-122     1.63    -0.15  1.0000
  16 -18    0      192      249
423    -5.81    -0.20  1.0000
  14 -18    0       25      214
-22     4.37    -0.11  1.0000
  12 -18    0      224      234
819    11.85     0.07  1.0000
  10 -18    0        1      198
-50    -0.64     0.06  1.0000
   8 -18    0      802      231
605     3.23     0.20  1.0000
   6 -18    0      856      306
1700    10.38     0.00  1.0000
   4 -18    0        0      186
-98    -0.48    -0.14  1.0000
   2 -18    0      147      231
283    -4.08     0.05  1.0000
   0 -18    0      910      246
491     6.94    -0.03  1.0000
  -2 -18    0      152      245
```

104 -1.86 0.04 1.0000
4 18 0 1437 327
2195 7.75 -0.21 1.0000
6 18 0 3810 300
3341 3.40 0.19 1.0000
6 18 0 3501 370
3052 3.40 0.19 1.0000
8 18 0 276 188
109 5.36 0.18 1.0000
8 18 0 245 240
90 5.36 0.18 1.0000
10 18 0 21 220
-1 -3.97 -0.21 1.0000
10 18 0 21 203
-24 -3.97 -0.21 1.0000
12 18 0 996 260
413 -9.76 -0.50 1.0000
12 18 0 687 231
224 -9.76 -0.50 1.0000
14 18 0 0 263
98 8.35 0.45 1.0000
14 18 0 101 275
388 8.35 0.45 1.0000
16 18 0 455 325
752 -5.27 -0.54 1.0000
16 18 0 387 306
663 -5.27 -0.54 1.0000
18 18 0 4 325
-239 1.65 -0.09 1.0000
18 18 0 11 338
27 1.65 -0.09 1.0000
17 -19 0 487 283
701 3.83 -0.02 1.0000
15 -19 0 3 214
4 -0.18 0.13 1.0000
13 -19 0 44 217
196 -6.36 0.14 1.0000
11 -19 0 37 214
9 -2.70 -0.04 1.0000
9 -19 0 235 201
-125 -13.29 0.15 1.0000
7 -19 0 876 239
803 -1.09 0.00 1.0000
5 -19 0 2602 348

2921 2.63 0.04 1.0000
3 -19 0 1 213
25 -5.05 -0.15 1.0000
1 -19 0 4 220
-10 1.76 0.05 1.0000
-1 -19 0 34 221
-55 5.05 -0.01 1.0000
-3 -19 0 755 275
434 -5.76 0.11 1.0000
5 19 0 139 213
173 -1.18 0.18 1.0000
7 19 0 1499 297
1470 0.33 -0.04 1.0000
7 19 0 2022 280
1988 0.33 -0.04 1.0000
9 19 0 12 245
-179 -3.05 -0.25 1.0000
9 19 0 163 216
266 -3.05 -0.25 1.0000
11 19 0 245 249
295 -1.29 -0.30 1.0000
11 19 0 137 249
175 -1.29 -0.30 1.0000
13 19 0 1035 393
2451 15.02 0.42 1.0000
13 19 0 730 357
1966 15.02 0.42 1.0000
15 19 0 399 298
212 -4.68 0.05 1.0000
15 19 0 878 322
587 -4.68 0.05 1.0000
17 19 0 440 340
298 3.30 0.19 1.0000
17 19 0 580 335
415 3.30 0.19 1.0000
16 -20 0 15 261
-52 3.38 -0.11 1.0000
14 -20 0 161 242
141 -0.70 0.03 1.0000
12 -20 0 184 232
122 2.18 0.06 1.0000
10 -20 0 46 242
341 10.15 -0.07 1.0000
8 -20 0 400 244

416 0.35 -0.03 1.0000
6 -20 0 1397 299
1386 -0.13 -0.06 1.0000
4 -20 0 32 227
146 -5.56 0.21 1.0000
2 -20 0 154 255
267 3.41 -0.07 1.0000
0 -20 0 276 247
174 -2.98 0.05 1.0000
-2 -20 0 1453 304
1064 -4.77 0.01 1.0000
6 20 0 705 281
1397 -9.38 -0.41 1.0000
8 20 0 13 235
-189 3.10 -0.13 1.0000
10 20 0 571 310
530 0.76 0.16 1.0000
10 20 0 1151 264
1092 0.76 0.16 1.0000
12 20 0 11 263
-4 2.80 0.37 1.0000
12 20 0 11 267
-63 2.80 0.37 1.0000
14 20 0 136 266
-67 -9.98 -1.54 1.0000
14 20 0 226 308
31 -9.98 -1.54 1.0000
16 20 0 310 316
401 2.09 0.23 1.0000
16 20 0 40 344
77 2.09 0.23 1.0000
15 -21 0 127 275
126 0.03 -0.11 1.0000
13 -21 0 41 245
83 -2.36 0.03 1.0000
11 -21 0 149 244
263 -3.47 0.15 1.0000
9 -21 0 38 229
-30 -5.33 -0.05 1.0000
7 -21 0 23 221
-8 -4.12 0.12 1.0000
5 -21 0 186 239
216 0.92 0.02 1.0000
3 -21 0 416 245

119 8.22 -0.11 1.0000
1 -21 0 18 234
-296 -3.65 -0.03 1.0000
-1 -21 0 109 257
169 2.23 -0.03 1.0000
-3 -21 0 55 280
-40 6.44 0.07 1.0000
7 21 0 198 229
-48 12.18 0.37 1.0000
9 21 0 35 256
-248 -5.11 -0.53 1.0000
11 21 0 0 297
-58 -0.44 -0.16 1.0000
13 21 0 90 287
44 2.46 0.24 1.0000
15 21 0 349 300
209 3.68 0.48 1.0000
14 -22 0 325 292
500 3.76 -0.06 1.0000
12 -22 0 519 273
459 1.18 -0.09 1.0000
10 -22 0 294 270
289 0.14 0.08 1.0000
8 -22 0 76 243
157 3.32 0.06 1.0000
6 -22 0 139 255
344 -5.87 -0.08 1.0000
4 -22 0 296 269
390 -2.21 0.05 1.0000
2 -22 0 19 244
-234 -3.80 0.04 1.0000
0 -22 0 22 254
-3 -4.05 0.11 1.0000
-2 -22 0 8 249
-287 2.37 -0.03 1.0000
-4 -22 0 71 309
-27 -7.30 0.17 1.0000
8 22 0 463 252
180 -7.03 -0.14 1.0000
10 22 0 273 287
246 0.74 0.14 1.0000
12 22 0 3 307
-176 0.97 -1.01 1.0000
14 22 0 389 322

415 -0.57 0.37 1.0000
13 -23 0 446 296
596 2.86 0.06 1.0000
11 -23 0 88 286
319 -7.33 0.05 1.0000
9 -23 0 32 256
29 0.25 -0.06 1.0000
7 -23 0 0 235
-285 0.48 0.05 1.0000
5 -23 0 124 251
134 -0.39 -0.01 1.0000
3 -23 0 844 284
795 0.74 0.02 1.0000
1 -23 0 34 269
-65 5.08 0.01 1.0000
-1 -23 0 33 285
-120 4.97 0.03 1.0000
-3 -23 0 77 298
271 -6.67 0.12 1.0000
-5 -23 0 74 335
421 10.34 -0.31 1.0000
9 23 0 241 294
462 5.16 0.32 1.0000
11 23 0 21 309
-133 3.98 0.43 1.0000
13 23 0 350 354
543 -3.98 -0.07 1.0000
10 -24 0 296 297
265 0.79 -0.01 1.0000
8 -24 0 20 264
-10 -3.91 -0.01 1.0000
6 -24 0 0 257
-44 0.52 0.06 1.0000
4 -24 0 152 274
285 -3.93 -0.10 1.0000
2 -24 0 323 297
458 -2.98 0.17 1.0000
0 -24 0 6 298
-131 -2.17 -0.12 1.0000
-2 -24 0 338 334
390 -1.17 0.00 1.0000
-4 -24 0 9 279
-325 2.56 0.21 1.0000
-6 -24 0 77 342

191 -4.38 -0.29 1.0000
10 24 0 23 301
37 -0.55 -1.00 1.0000
9 -25 0 12 269
-323 -3.01 0.03 1.0000
7 -25 0 9 276
-282 -2.60 -0.01 1.0000
5 -25 0 202 272
284 2.29 0.03 1.0000
3 -25 0 85 313
90 -0.25 0.04 1.0000
1 -25 0 220 312
395 4.36 -0.07 1.0000
-1 -25 0 226 321
347 -3.10 0.24 1.0000
-3 -25 0 0 328
25 4.02 -0.17 1.0000
-5 -25 0 121 335
64 -3.29 0.72 1.0000
-7 -25 0 15 377
-145 3.30 -0.19 1.0000
6 -26 0 14 294
-2 -3.29 0.00 1.0000
4 -26 0 134 311
160 -0.92 0.03 1.0000
2 -26 0 139 317
204 -2.16 -0.01 1.0000
0 -26 0 0 319
-110 0.57 0.05 1.0000
-2 -26 0 26 346
43 1.30 0.10 1.0000
-4 -26 0 52 351
69 -0.89 -0.45 1.0000
-6 -26 0 434 373
379 1.19 0.07 1.0000
-5 -26 1 23 350
67 -3.88 3.66 1.0000
-3 -26 1 7 327
-294 -0.01 -2.31 1.0000
-1 -26 1 77 339
119 1.79 0.71 1.0000
1 -26 1 155 330
127 -1.48 -0.55 1.0000
3 -26 1 1 319

-93 0.92 0.34 1.0000
5 -26 1 0 308
-62 -0.46 -0.34 1.0000
-6 -25 1 481 402
573 -1.81 0.03 1.0000
-4 -25 1 527 352
456 3.13 0.86 1.0000
-2 -25 1 5 303
-279 0.32 1.89 1.0000
0 -25 1 200 317
200 -0.29 0.05 1.0000
2 -25 1 299 290
224 1.99 2.80 1.0000
4 -25 1 11 279
-283 -1.94 2.18 1.0000
6 -25 1 11 284
-204 -2.02 -2.02 1.0000
8 -25 1 1 276
-142 0.46 0.69 1.0000
11 24 -1 208 307
294 -1.98 -1.48 1.0000
9 24 -1 21 296
34 0.15 1.05 1.0000
-5 -24 1 393 335
234 2.92 -3.54 1.0000
-3 -24 1 315 314
413 -2.78 -0.30 1.0000
-1 -24 1 4 294
-57 -1.31 1.13 1.0000
1 -24 1 9 301
54 1.78 -3.43 1.0000
3 -24 1 722 316
1064 -4.55 -2.17 1.0000
5 -24 1 4 265
-249 1.65 -0.18 1.0000
7 -24 1 4 255
-174 -0.45 1.63 1.0000
9 -24 1 204 282
361 -5.24 -1.87 1.0000
11 -24 1 132 287
261 -1.72 3.66 1.0000
12 23 -1 1 354
-48 0.60 -0.16 1.0000
12 23 -1 1 314

-72 0.60 -0.16 1.0000
10 23 -1 170 305
285 2.39 -2.48 1.0000
10 23 -1 249 324
385 2.39 -2.48 1.0000
8 23 -1 7 258
-224 1.42 1.87 1.0000
-4 -23 1 101 292
153 1.35 2.71 1.0000
-2 -23 1 3 271
-47 1.26 -0.66 1.0000
0 -23 1 54 288
30 3.35 3.81 1.0000
2 -23 1 83 272
104 -1.80 3.65 1.0000
4 -23 1 4 254
-481 -1.15 1.37 1.0000
6 -23 1 64 266
40 1.14 2.90 1.0000
8 -23 1 192 266
143 -1.44 -2.22 1.0000
10 -23 1 32 264
86 1.24 -3.38 1.0000
12 -23 1 153 287
158 1.27 -0.10 1.0000
15 22 -1 349 346
611 6.27 3.03 1.0000
15 22 -1 149 418
322 6.27 3.03 1.0000
13 22 -1 203 335
482 -6.65 -5.01 1.0000
13 22 -1 388 373
763 -6.65 -5.01 1.0000
11 22 -1 16 295
-147 1.37 3.18 1.0000
11 22 -1 143 325
64 1.37 3.18 1.0000
9 22 -1 164 284
160 -0.43 0.57 1.0000
9 22 -1 652 294
644 -0.43 0.57 1.0000
7 22 -1 108 246
47 -3.57 0.26 1.0000
7 22 -1 215 234

126   -3.57   0.26 1.0000
  -5 -22   1   249   318
162   -1.95   -2.26 1.0000
  -3 -22   1   220   271
206   -1.91   2.25 1.0000
  -1 -22   1   17   279
32   -1.62   -4.18 1.0000
   1 -22   1   87   247
48   -3.05   0.67 1.0000
   3 -22   1   173   273
386   6.80   -2.48 1.0000
   5 -22   1   852   273
569   -6.43   -4.07 1.0000
   7 -22   1   16   225
-160   -3.33   0.87 1.0000
   9 -22   1   183   254
288   0.98   4.14 1.0000
  11 -22   1   26   261
-8   -4.06   1.72 1.0000
  13 -22   1   223   267
219   0.06   0.25 1.0000
  15 -22   1   12   290
-36   -1.12   2.73 1.0000
  16 21   -1   479   322
387   0.19   -2.31 1.0000
  16 21   -1   498   389
404   0.19   -2.31 1.0000
  14 21   -1   92   356
167   -3.37   -0.30 1.0000
  14 21   -1   483   309
645   -3.37   -0.30 1.0000
  12 21   -1   45   300
-20   5.81   0.59 1.0000
  12 21   -1   45   273
-21   5.81   0.59 1.0000
  10 21   -1   150   275
113   2.10   0.87 1.0000
  10 21   -1   7   275
-229   2.10   0.87 1.0000
   8 21   -1   351   250
195   -3.49   -4.15 1.0000
   8 21   -1   684   242
464   -3.49   -4.15 1.0000
   6 21   -1   16   216

```
203      8.87    -0.21  1.0000
  -4 -21    1       143       292
174    -1.14     1.47  1.0000
  -2 -21    1       667       298
810      3.01     1.07  1.0000
   0 -21    1       216       279
295      0.84     2.08  1.0000
   2 -21    1       412       269
443    -1.60     1.16  1.0000
   4 -21    1       634       268
687    -1.64     4.37  1.0000
   6 -21    1       858       268
733    -3.25    -0.34  1.0000
   8 -21    1        25       224
-26      3.73    -2.21  1.0000
  10 -21    1       255       255
331    -1.46    -2.88  1.0000
  12 -21    1       437       267
683    -0.60    -4.50  1.0000
  14 -21    1        39       255
104      3.28    -2.08  1.0000
  16 -21    1        11       269
-36    -0.05     2.90  1.0000
  17  20   -1        77       389
189      0.76     4.35  1.0000
  17  20   -1       328       341
533      0.76     4.35  1.0000
  15  20   -1       489       360
639    -6.06    -1.35  1.0000
  15  20   -1       260       291
360    -6.06    -1.35  1.0000
  13  20   -1       120       313
-36      8.41     4.38  1.0000
  13  20   -1         2       272
133      8.41     4.38  1.0000
  11  20   -1       469       289
607    -2.82    -1.43  1.0000
  11  20   -1        36       247
77    -2.82    -1.43  1.0000
   9  20   -1        92       265
-16    -7.51    -3.56  1.0000
   9  20   -1       124       228
219    -7.51    -3.56  1.0000
   7  20   -1       165       243
```

```
488     6.17     6.88  1.0000
   7  20  -1      486       230
996     6.17     6.88  1.0000
   5  20  -1      125       184
94  -13.15   -3.74  1.0000
   3  20  -1       91       167
45     0.76     2.59  1.0000
  -3 -20   1      116       276
63     0.76    -2.59  1.0000
  -1 -20   1      480       279
420     1.30    -0.54  1.0000
   1 -20   1      706       282
756    -2.30    -0.16  1.0000
   3 -20   1      467       248
352     1.07    -2.41  1.0000
   5 -20   1      833       299
1363     6.61     2.42  1.0000
   7 -20   1       94       222
165    -2.79    -1.39  1.0000
   9 -20   1      372       244
677    -4.56     4.49  1.0000
  11 -20   1      209       251
396     1.45     4.56  1.0000
  13 -20   1       64       243
193    -3.24     4.27  1.0000
  15 -20   1      541       270
486     0.88     0.62  1.0000
  17 -20   1       65       269
175     2.42    -3.81  1.0000
  18  19  -1      189       390
327     2.73    -2.95  1.0000
  18  19  -1      221       352
368     2.73    -2.95  1.0000
  16  19  -1      601       360
795     1.27     3.14  1.0000
  16  19  -1       36       296
92     1.27     3.14  1.0000
  14  19  -1      301       280
514     4.77    -6.33  1.0000
  14  19  -1      444       328
709     4.77    -6.33  1.0000
  12  19  -1       30       259
-48    -3.26    -3.50  1.0000
  12  19  -1       30       242
```

-338	-3.26	-3.50	1.0000	
10	19	-1	100	245
143	-1.97	0.14	1.0000	
10	19	-1	293	241
364	-1.97	0.14	1.0000	
8	19	-1	123	240
55	11.78	3.48	1.0000	
8	19	-1	120	202
78	11.78	3.48	1.0000	
6	19	-1	347	176
196	-5.68	-3.73	1.0000	
6	19	-1	315	215
170	-5.68	-3.73	1.0000	
4	19	-1	1069	284
1997	7.26	7.79	1.0000	
2	19	-1	248	165
393	0.57	-4.53	1.0000	
-2	-19	1	547	272
761	0.57	4.53	1.0000	
0	-19	1	103	240
206	-3.93	0.63	1.0000	
2	-19	1	410	236
329	0.61	-1.74	1.0000	
4	-19	1	176	231
212	-4.36	2.74	1.0000	
6	-19	1	537	240
732	-1.79	-3.76	1.0000	
8	-19	1	38	218
224	-7.38	-2.22	1.0000	
10	-19	1	31	221
186	3.31	-6.26	1.0000	
12	-19	1	53	232
165	5.59	-3.00	1.0000	
14	-19	1	57	221
-65	-4.95	-4.31	1.0000	
16	-19	1	154	243
130	2.02	0.35	1.0000	
18	-19	1	175	266
276	1.92	4.60	1.0000	
19	18	-1	287	386
421	1.97	4.63	1.0000	
19	18	-1	34	335
-309	1.97	4.63	1.0000	
17	18	-1	633	340

762 -0.22 -3.58 1.0000
17 18 -1 682 353
816 -0.22 -3.58 1.0000
15 18 -1 1546 351
1618 0.09 -0.81 1.0000
15 18 -1 2089 387
2173 0.09 -0.81 1.0000
13 18 -1 118 242
166 -3.68 7.16 1.0000
13 18 -1 69 261
71 -3.68 7.16 1.0000
11 18 -1 16 226
-46 2.92 -1.92 1.0000
11 18 -1 16 225
-48 2.92 -1.92 1.0000
9 18 -1 107 202
-32 5.62 6.96 1.0000
9 18 -1 228 229
23 5.62 6.96 1.0000
7 18 -1 492 330
2017 -18.49 -9.46 1.0000
7 18 -1 379 279
1774 -18.49 -9.46 1.0000
5 18 -1 1224 191
896 11.37 -4.56 1.0000
5 18 -1 1116 242
796 11.37 -4.56 1.0000
3 18 -1 224 156
290 -7.35 -0.44 1.0000
1 18 -1 21 141
210 -7.08 -5.56 1.0000
-1 -18 1 108 217
-75 -7.08 5.56 1.0000
1 -18 1 104 261
932 20.69 -2.03 1.0000
3 -18 1 67 217
237 -6.64 -0.20 1.0000
5 -18 1 253 222
284 1.90 1.99 1.0000
7 -18 1 256 199
105 12.85 5.70 1.0000
9 -18 1 448 220
412 -4.70 3.67 1.0000
11 -18 1 426 230

375 -4.18 3.13 1.0000
13 -18 1 211 234
425 -0.96 6.44 1.0000
15 -18 1 109 235
180 1.68 -2.17 1.0000
17 -18 1 42 229
31 -2.98 2.24 1.0000
19 -18 1 82 272
180 2.69 -3.06 1.0000
20 17 -1 306 378
511 2.49 -3.68 1.0000
20 17 -1 29 337
111 2.49 -3.68 1.0000
18 17 -1 20 280
-61 -3.09 2.39 1.0000
18 17 -1 437 350
451 -3.09 2.39 1.0000
16 17 -1 119 288
248 -0.19 -4.26 1.0000
16 17 -1 38 303
121 -0.19 -4.26 1.0000
14 17 -1 1025 283
923 3.64 4.63 1.0000
14 17 -1 948 298
848 3.64 4.63 1.0000
12 17 -1 1253 279
1018 -2.70 -2.61 1.0000
12 17 -1 982 252
775 -2.70 -2.61 1.0000
10 17 -1 459 198
232 2.51 -5.62 1.0000
10 17 -1 50 198
-73 2.51 -5.62 1.0000
8 17 -1 2417 329
2300 -0.98 0.97 1.0000
8 17 -1 2004 280
1898 -0.98 0.97 1.0000
6 17 -1 868 187
694 3.30 15.30 1.0000
6 17 -1 1216 252
1079 3.30 15.30 1.0000
4 17 -1 1899 300
1911 -0.07 0.13 1.0000
4 17 -1 1621 243

1632 -0.07 0.13 1.0000
2 17 -1 1397 258
2490 0.16 10.93 1.0000
-2 -17 1 1685 365
2870 0.16 -10.93 1.0000
0 17 -1 576 170
935 -1.98 8.44 1.0000
0 -17 1 527 250
872 -1.98 -8.44 1.0000
2 -17 1 955 308
1908 -8.77 11.93 1.0000
4 -17 1 682 263
1459 -1.44 -10.99 1.0000
6 -17 1 319 213
513 -7.42 1.33 1.0000
8 -17 1 104 182
13 2.78 -5.40 1.0000
10 -17 1 512 207
315 -2.29 -6.47 1.0000
12 -17 1 399 213
306 -1.90 -2.93 1.0000
14 -17 1 461 217
291 10.46 -0.46 1.0000
16 -17 1 439 229
410 -3.10 0.85 1.0000
18 -17 1 325 258
421 -0.24 -3.75 1.0000
20 -17 1 30 275
110 0.82 4.38 1.0000
21 16 -1 805 390
1018 -0.11 3.08 1.0000
21 16 -1 13 339
-184 -0.11 3.08 1.0000
19 16 -1 6 314
-231 2.03 0.36 1.0000
19 16 -1 11 331
3 2.03 0.36 1.0000
17 16 -1 13 245
-183 -3.02 0.79 1.0000
17 16 -1 385 296
355 -3.02 0.79 1.0000
15 16 -1 229 284
702 8.82 5.37 1.0000
15 16 -1 112 284

477	8.82	5.37	1.0000	
13	16	-1	300	238
621	0.75	-10.96	1.0000	
13	16	-1	321	280
655	0.75	-10.96	1.0000	
11	16	-1	491	226
120	-3.12	9.28	1.0000	
11	16	-1	128	195
-47	-3.12	9.28	1.0000	
9	16	-1	589	211
268	3.00	-6.25	1.0000	
9	16	-1	563	179
251	3.00	-6.25	1.0000	
7	16	-1	1371	255
2179	7.23	-7.23	1.0000	
7	16	-1	1119	298
1854	7.23	-7.23	1.0000	
5	16	-1	656	181
882	-10.67	-4.76	1.0000	
5	16	-1	604	233
814	-10.67	-4.76	1.0000	
3	16	-1	562	122
8	2.52	-19.85	1.0000	
1	16	-1	787	145
436	15.77	-6.15	1.0000	
-1	-16	1	1158	226
817	15.77	6.15	1.0000	
-1	16	-1	616	111
53	-16.69	-1.50	1.0000	
1	-16	1	374	179
-128	-16.69	1.50	1.0000	
3	-16	1	2095	222
708	26.45	5.51	1.0000	
5	-16	1	1508	272
1562	8.72	-3.44	1.0000	
7	-16	1	847	249
1384	-15.89	3.22	1.0000	
9	-16	1	387	179
-14	15.18	7.75	1.0000	
11	-16	1	182	187
162	-4.82	3.06	1.0000	
13	-16	1	138	203
57	-7.11	-0.79	1.0000	
15	-16	1	278	219

338 0.34 2.86 1.0000
17 -16 1 225 232
382 -0.08 -4.00 1.0000
19 -16 1 148 255
245 1.15 3.07 1.0000
21 -16 1 8 273
-65 0.22 -2.45 1.0000
22 15 -1 8 318
17 -1.10 2.86 1.0000
22 15 -1 621 384
752 -1.10 2.86 1.0000
20 15 -1 1254 363
1171 0.83 0.63 1.0000
20 15 -1 241 322
205 0.83 0.63 1.0000
18 15 -1 10 284
-91 1.65 -2.18 1.0000
18 15 -1 377 313
502 1.65 -2.18 1.0000
16 15 -1 272 276
340 0.24 3.06 1.0000
16 15 -1 416 282
501 0.24 3.06 1.0000
14 15 -1 632 295
1394 -12.77 -6.29 1.0000
14 15 -1 1038 324
2006 -12.77 -6.29 1.0000
12 15 -1 70 246
227 6.05 2.47 1.0000
12 15 -1 57 199
-91 6.05 2.47 1.0000
10 15 -1 160 170
160 3.43 6.70 1.0000
10 15 -1 202 210
214 3.43 6.70 1.0000
8 15 -1 2134 300
3476 -17.46 5.82 1.0000
8 15 -1 3076 355
4714 -17.46 5.82 1.0000
6 15 -1 1985 169
804 14.05 6.58 1.0000
6 15 -1 2155 215
916 14.05 6.58 1.0000
4 15 -1 925 141

473 -5.84 17.52 1.0000
4 15 -1 891 193
439 -5.84 17.52 1.0000
2 15 -1 966 189
1050 -4.64 16.20 1.0000
0 15 -1 921 158
966 0.15 -2.30 1.0000
0 -15 1 1535 272
1590 0.15 2.30 1.0000
2 -15 1 1869 209
531 -5.99 -18.61 1.0000
2 -15 1 1613 97
392 -5.99 -18.61 1.0000
4 -15 1 256 164
30 -11.79 11.77 1.0000
6 -15 1 1488 254
1458 2.88 -0.34 1.0000
8 -15 1 657 170
240 -1.63 -9.66 1.0000
10 -15 1 661 206
588 3.26 4.97 1.0000
12 -15 1 822 249
1248 7.64 -7.09 1.0000
14 -15 1 1200 248
1134 -4.33 -2.34 1.0000
16 -15 1 17 197
70 8.75 5.32 1.0000
18 -15 1 1210 278
1287 0.16 -0.95 1.0000
20 -15 1 257 256
356 -6.08 -1.38 1.0000
22 -15 1 152 320
324 6.25 2.98 1.0000
21 14 -1 20 289
-206 3.26 -2.09 1.0000
21 14 -1 169 362
291 3.26 -2.09 1.0000
19 14 -1 351 312
316 -4.81 -4.34 1.0000
19 14 -1 56 244
-143 -4.81 -4.34 1.0000
17 14 -1 403 231
236 10.55 -0.45 1.0000
17 14 -1 600 289

427	10.55	-0.45	1.0000	
15	14	-1	1274	299
1205	-4.37	-2.38	1.0000	
15	14	-1	1760	301
1685	-4.37	-2.38	1.0000	
13	14	-1	1269	277
1839	6.12	8.00	1.0000	
13	14	-1	940	290
1425	6.12	8.00	1.0000	
11	14	-1	119	190
789	14.24	-8.50	1.0000	
11	14	-1	166	254
917	14.24	-8.50	1.0000	
9	14	-1	1326	150
413	-11.91	-8.37	1.0000	
9	14	-1	1143	199
312	-11.91	-8.37	1.0000	
7	14	-1	3282	392
6702	20.53	-6.25	1.0000	
7	14	-1	2196	331
5101	20.53	-6.25	1.0000	
5	14	-1	1760	283
2257	-6.22	-23.05	1.0000	
5	14	-1	1855	213
2388	-6.22	-23.05	1.0000	
3	14	-1	1787	270
2726	2.20	-11.46	1.0000	
3	14	-1	1994	225
2985	2.20	-11.46	1.0000	
1	14	-1	1388	208
2458	2.80	-10.86	1.0000	
-1	-14	1	1447	285
2537	2.80	10.86	1.0000	
-1	14	-1	487	179
1662	12.93	-11.09	1.0000	
1	-14	1	346	236
1379	12.93	11.09	1.0000	
3	-14	1	1419	113
956	-1.86	-5.55	1.0000	
3	-14	1	1059	193
665	-1.86	-5.55	1.0000	
5	-14	1	254	170
233	1.65	1.59	1.0000	
7	-14	1	208	151

59   18.10   -1.98 1.0000
9 -14   1   32   189
409   -20.90   -4.37 1.0000
11 -14   1   577   179
344   0.70   5.20 1.0000
13 -14   1   58   177
-23   4.74   4.58 1.0000
15 -14   1   836   302
1711   -12.79   -6.32 1.0000
17 -14   1   478   225
400   3.56   4.43 1.0000
19 -14   1   69   231
138   4.85   -6.24 1.0000
21 -14   1   67   269
131   -3.30   -0.21 1.0000
22 13   -1   829   374
823   0.15   0.32 1.0000
22 13   -1   18   355
17   0.15   0.32 1.0000
20 13   -1   172   334
365   -3.28   4.29 1.0000
20 13   -1   39   287
-250   -3.28   4.29 1.0000
18 13   -1   287   304
537   -1.06   6.51 1.0000
18 13   -1   177   266
377   -1.06   6.51 1.0000
16 13   -1   221   240
136   -6.94   -0.69 1.0000
16 13   -1   250   220
163   -6.94   -0.69 1.0000
14 13   -1   58   234
-1   4.76   4.59 1.0000
14 13   -1   58   196
-47   4.76   4.59 1.0000
12 13   -1   91   219
27   -7.34   -1.11 1.0000
12 13   -1   243   168
195   -7.34   -1.11 1.0000
10 13   -1   4137   349
4125   -16.66   -0.28 1.0000
10 13   -1   3459   282
3414   -16.66   -0.28 1.0000
8 13   -1   895   220

2019 23.70 13.95 1.0000
6 13 -1 110 172
975 -31.25 10.93 1.0000
6 13 -1 186 182
711 -31.25 10.93 1.0000
4 13 -1 4918 495
10195 17.78 21.96 1.0000
4 13 -1 5343 539
10808 17.78 21.96 1.0000
2 13 -1 4092 287
5062 -1.55 9.71 1.0000
2 13 -1 3716 323
4639 -1.55 9.71 1.0000
0 13 -1 1027 111
321 -14.30 8.58 1.0000
0 -13 1 1022 164
333 -14.30 -8.58 1.0000
-2 13 -1 2342 237
3564 6.52 -7.53 1.0000
2 -13 1 2318 297
3535 6.52 7.53 1.0000
2 -13 1 2528 186
3793 6.52 7.53 1.0000
4 -13 1 179 155
380 -5.96 -1.17 1.0000
6 -13 1 422 157
484 -2.04 2.94 1.0000
8 -13 1 2078 318
3974 5.09 14.51 1.0000
10 -13 1 97 176
446 6.05 -8.38 1.0000
12 -13 1 184 185
190 6.07 -2.90 1.0000
14 -13 1 777 235
1216 6.12 8.02 1.0000
16 -13 1 85 202
191 0.81 -11.05 1.0000
18 -13 1 72 214
4 -3.69 7.35 1.0000
20 -13 1 2 246
101 8.45 4.14 1.0000
22 -13 1 257 314
570 -6.66 -5.10 1.0000
23 12 -1 2 362

```
-53     1.27    -0.07  1.0000
  21   12   -1     1535      365
1977    -0.56    -4.56  1.0000
  21   12   -1       78      345
199    -0.56    -4.56  1.0000
  19   12   -1       53      291
-118     5.55    -2.98  1.0000
  19   12   -1      274      276
509     5.55    -2.98  1.0000
  17   12   -1      556      228
448    -1.90    -2.97  1.0000
  17   12   -1      153      266
94    -1.90    -2.97  1.0000
  15   12   -1      928      284
1392     7.63    -7.18  1.0000
  15   12   -1     1129      254
1647     7.63    -7.18  1.0000
  13   12   -1      187      199
192     6.19    -2.90  1.0000
  13   12   -1      279      182
302     6.19    -2.90  1.0000
  11   12   -1      169      146
186    -4.82    -0.90  1.0000
  11   12   -1      143      187
155    -4.82    -0.90  1.0000
   9   12   -1     3068      165
997    35.26    -1.40  1.0000
   9   12   -1     3572      240
1421    35.26    -1.40  1.0000
   7   12   -1     1539      332
6035   -31.52   -12.98  1.0000
   5   12   -1     1230      231
2107    24.11   -17.95  1.0000
   5   12   -1     1047      180
1728    24.11   -17.95  1.0000
   3   12   -1     2567      236
2205     2.59   -19.93  1.0000
   3   12   -1     2475      188
2109     2.59   -19.93  1.0000
   1   12   -1    15059      920
21744   -24.39   -14.39  1.0000
  -1   12   -1     3025      302
5723    22.98     7.47  1.0000
   1  -12    1     2910      359
```

5559 22.98 -7.47 1.0000
-3 12 -1 6406 304
5775 -8.02 3.41 1.0000
3 -12 1 6707 361
6063 -8.02 -3.41 1.0000
3 -12 1 6173 262
5552 -8.02 -3.41 1.0000
5 -12 1 1723 185
996 -2.26 -14.69 1.0000
7 -12 1 4736 288
3500 -4.83 -7.02 1.0000
9 -12 1 3419 306
3697 2.81 -5.62 1.0000
11 -12 1 108 176
382 -11.05 2.92 1.0000
13 -12 1 326 175
274 -7.26 -1.32 1.0000
15 -12 1 29 192
139 6.01 2.46 1.0000
17 -12 1 709 217
528 -2.80 -2.72 1.0000
19 -12 1 64 211
6 -3.18 -3.59 1.0000
21 -12 1 173 255
71 5.70 0.68 1.0000
23 -12 1 98 300
110 0.72 -0.14 1.0000
23 -12 1 216 282
233 0.72 -0.14 1.0000
24 11 -1 21 356
-52 -1.75 3.56 1.0000
22 11 -1 26 297
-235 -4.10 1.65 1.0000
20 11 -1 668 283
985 1.55 4.63 1.0000
20 11 -1 154 298
321 1.55 4.63 1.0000
18 11 -1 592 256
537 -4.14 3.07 1.0000
18 11 -1 433 291
381 -4.14 3.07 1.0000
16 11 -1 253 251
237 -4.77 3.04 1.0000
16 11 -1 266 223

251   -4.77   3.04 1.0000
  14   11   -1   749   246
472   0.75   5.39 1.0000
  14   11   -1   459   189
248   0.75   5.39 1.0000
  12   11   -1   164   162
504   -11.00   2.94 1.0000
  12   11   -1   153   181
482   -11.00   2.94 1.0000
  10   11   -1   205   125
134   4.50   3.20 1.0000
  10   11   -1   196   181
126   4.50   3.20 1.0000
   8   11   -1   2119   324
5051   -21.62   -5.55 1.0000
   8   11   -1   1410   264
3910   -21.62   -5.55 1.0000
   6   11   -1   771   259
4242   30.07   14.38 1.0000
   4   11   -1   1596   267
4621   -23.32   8.82 1.0000
   4   11   -1   1727   306
4844   -23.32   8.82 1.0000
   2   11   -1   190   140
836   12.56   7.46 1.0000
   2   11   -1   326   170
1118   12.56   7.46 1.0000
   0   11   -1   6355   371
7715   20.28   16.58 1.0000
   0 -11   1   6157   426
7486   20.28   -16.58 1.0000
  -2   11   -1   2556   360
7476   -27.41   -18.18 1.0000
   2 -11   1   2610   413
7584   -27.41   18.18 1.0000
   2 -11   1   2311   314
7060   -27.41   18.18 1.0000
   4 -11   1   621   143
2363   19.16   23.22 1.0000
   4 -11   1   625   227
2374   19.16   23.22 1.0000
   6 -11   1   8129   509
10139   -3.45   9.19 1.0000
   8 -11   1   1802   226

```
1894     2.16     9.83  1.0000
  10 -11    1      684      236
1771    17.10     0.41  1.0000
  12 -11    1       60      149
56    -4.78    -1.00  1.0000
  14 -11    1       87      197
679    14.02    -8.59  1.0000
  16 -11    1      539      179
144    -3.04     9.30  1.0000
  18 -11    1        7      198
31      3.08    -1.98  1.0000
  20 -11    1      263      256
369    -2.80    -1.46  1.0000
  22 -11    1       16      270
-41      1.47     3.17  1.0000
  22 -11    1       16      237
-43      1.47     3.17  1.0000
  24 -11    1       12      285
39    -2.06    -1.51  1.0000
  24 -11    1        4      288
21    -2.06    -1.51  1.0000
  23  10   -1       15      349
53      1.22    -3.31  1.0000
  21  10   -1     1047      306
1205    -1.46    -2.87  1.0000
  21  10   -1      225      306
296    -1.46    -2.87  1.0000
  19  10   -1      205      253
501      3.39    -6.18  1.0000
  19  10   -1       66      272
-58      3.39    -6.18  1.0000
  17  10   -1     1246      228
947    -2.34    -6.45  1.0000
  17  10   -1      599      274
385    -2.34    -6.45  1.0000
  15  10   -1      895      282
817      3.26     4.98  1.0000
  15  10   -1      869      198
791      3.26     4.98  1.0000
  13  10   -1       95      218
441      6.19    -8.29  1.0000
  13  10   -1       83      162
413      6.19    -8.29  1.0000
  11  10   -1      845      276
```

2028 16.97 0.41 1.0000
11 10 -1 587 206
1591 16.97 0.41 1.0000
9 10 -1 2719 322
5071 -19.72 1.41 1.0000
9 10 -1 2190 274
4321 -19.72 1.41 1.0000
7 10 -1 4835 435
9209 34.21 -11.38 1.0000
5 10 -1 4932 314
6087 -26.67 2.64 1.0000
3 10 -1 2951 128
770 -18.69 -14.56 1.0000
3 10 -1 2821 149
703 -18.69 -14.56 1.0000
1 10 -1 2267 332
6071 25.81 -7.13 1.0000
1 10 -1 2301 308
6127 25.81 -7.13 1.0000
-1 10 -1 13698 383
7920 -49.02 -10.54 1.0000
1 -10 1 14375 429
8506 -49.02 10.54 1.0000
-3 10 -1 4007 446
9826 11.83 28.90 1.0000
3 -10 1 3923 480
9689 11.83 -28.90 1.0000
3 -10 1 4000 410
9811 11.83 -28.90 1.0000
5 -10 1 3928 184
1469 6.32 -20.17 1.0000
7 -10 1 3616 143
462 -25.52 -21.72 1.0000
9 -10 1 2913 257
3240 1.77 -2.15 1.0000
11 -10 1 156 145
95 4.23 3.16 1.0000
13 -10 1 2599 272
2494 -16.58 -0.40 1.0000
15 -10 1 125 171
115 3.42 6.49 1.0000
17 -10 1 50 182
-11 2.45 -5.59 1.0000
19 -10 1 121 214

171 -2.10 0.20 1.0000
21 -10 1 74 221
46 2.21 0.84 1.0000
21 -10 1 417 269
351 2.21 0.84 1.0000
23 -10 1 66 254
144 2.37 -2.58 1.0000
23 -10 1 16 263
-113 2.37 -2.58 1.0000
24 9 -1 40 342
-280 -5.12 -1.93 1.0000
22 9 -1 24 316
-10 1.12 4.08 1.0000
20 9 -1 204 301
442 -4.66 4.44 1.0000
18 9 -1 46 256
-15 -4.57 3.65 1.0000
18 9 -1 553 218
520 -4.57 3.65 1.0000
16 9 -1 391 231
-9 15.32 7.71 1.0000
16 9 -1 391 180
-91 15.32 7.71 1.0000
14 9 -1 18 233
662 -20.84 -4.52 1.0000
14 9 -1 16 169
616 -20.84 -4.52 1.0000
12 9 -1 2744 323
3004 2.94 -5.72 1.0000
12 9 -1 3232 199
3518 2.94 -5.72 1.0000
12 9 -1 3281 259
3569 2.94 -5.72 1.0000
10 9 -1 3172 303
3514 1.82 -2.15 1.0000
10 9 -1 3323 242
3673 1.82 -2.15 1.0000
8 9 -1 11741 549
12754 -9.08 0.44 1.0000
8 9 -1 9282 509
10175 -9.08 0.44 1.0000
6 9 -1 6482 348
7118 -3.58 -7.12 1.0000
4 9 -1 1238 229

4068 33.95 -2.43 1.0000
2 9 -1 1072 115
623 0.69 9.91 1.0000
2 9 -1 1410 139
897 0.69 9.91 1.0000
0 9 -1 10320 654
15299 5.41 -21.09 1.0000
0 -9 1 10417 693
15413 5.41 21.09 1.0000
-2 9 -1 1152 251
4960 31.00 6.26 1.0000
2 -9 1 1030 212
4700 31.00 -6.26 1.0000
2 -9 1 1077 288
4800 31.00 -6.26 1.0000
4 -9 1 6656 258
3920 -25.48 17.77 1.0000
4 -9 1 7074 196
4274 -25.48 17.77 1.0000
6 -9 1 4762 551
11735 15.18 42.45 1.0000
8 -9 1 256 119
87 8.96 -4.82 1.0000
10 -9 1 1734 272
3657 -19.92 1.77 1.0000
12 -9 1 2810 175
787 35.25 -1.37 1.0000
14 -9 1 1282 169
383 -11.82 -8.57 1.0000
16 -9 1 556 178
238 3.29 -6.32 1.0000
18 -9 1 395 199
93 5.65 6.91 1.0000
20 -9 1 54 175
24 -7.51 -3.51 1.0000
20 -9 1 91 228
-97 -7.51 -3.51 1.0000
22 -9 1 151 259
147 -0.43 0.62 1.0000
22 -9 1 177 215
173 -0.43 0.62 1.0000
24 -9 1 47 288
67 0.28 1.10 1.0000
25 8 -1 151 337

174	0.44	0.65	1.0000	
23	8	-1	10	289
-20	-1.49	-2.29	1.0000	
21	8	-1	440	268
262	3.67	-2.04	1.0000	
19	8	-1	55	245
265	-7.41	-2.25	1.0000	
17	8	-1	389	192
187	2.63	-5.42	1.0000	
17	8	-1	107	215
15	2.63	-5.42	1.0000	
15	8	-1	613	230
213	-1.55	-9.59	1.0000	
15	8	-1	461	161
125	-1.55	-9.59	1.0000	
13	8	-1	1537	331
3242	4.97	14.79	1.0000	
13	8	-1	1924	263
3796	4.97	14.79	1.0000	
11	8	-1	2087	199
2210	2.29	9.85	1.0000	
11	8	-1	1958	147
2073	2.29	9.85	1.0000	
11	8	-1	1929	247
2042	2.29	9.85	1.0000	
9	8	-1	209	97
46	9.09	-5.03	1.0000	
9	8	-1	362	149
181	9.09	-5.03	1.0000	
7	8	-1	7491	317
6219	-15.81	26.36	1.0000	
7	8	-1	5932	275
4662	-15.81	26.36	1.0000	
5	8	-1	2504	92
432	-0.19	-25.58	1.0000	
-5	-8	1	3170	119
737	-0.19	25.58	1.0000	
3	8	-1	4743	179
2820	-14.31	2.74	1.0000	
-3	-8	1	5602	206
3489	-14.31	-2.74	1.0000	
-3	-8	1	4585	178
2694	-14.31	-2.74	1.0000	
1	8	-1	20561	763

18458 -47.73 1.89 1.0000
1 8 -1 20925 780
18831 -47.73 1.89 1.0000
-1 -8 1 20110 777
18007 -47.73 -1.89 1.0000
-1 -8 1 19990 763
17884 -47.73 -1.89 1.0000
-1 8 -1 13013 393
8770 17.78 11.56 1.0000
1 -8 1 13044 360
8788 17.78 -11.56 1.0000
1 -8 1 13068 426
8808 17.78 -11.56 1.0000
1 -8 1 12930 393
8694 17.78 -11.56 1.0000
1 -8 1 13114 415
8846 17.78 -11.56 1.0000
-3 8 -1 139 101
700 -13.65 5.67 1.0000
3 -8 1 104 70
620 -13.65 -5.67 1.0000
3 -8 1 193 136
844 -13.65 -5.67 1.0000
3 -8 1 100 100
608 -13.65 -5.67 1.0000
5 -8 1 8181 810
19036 -33.23 -24.35 1.0000
5 -8 1 7789 762
18434 -33.23 -24.35 1.0000
7 -8 1 8540 386
7462 33.71 -20.43 1.0000
9 -8 1 8834 484
9737 -8.79 0.10 1.0000
11 -8 1 877 249
2994 -21.68 -5.83 1.0000
13 -8 1 739 236
1703 23.73 14.32 1.0000
15 -8 1 1305 283
2336 -17.68 5.91 1.0000
17 -8 1 1785 296
1707 -1.01 0.74 1.0000
19 -8 1 202 207
-142 11.85 3.40 1.0000
19 -8 1 122 168

108 11.85 3.40 1.0000
21 -8 1 39 222
-200 -3.40 -4.24 1.0000
21 -8 1 318 200
172 -3.40 -4.24 1.0000
23 -8 1 205 282
267 1.37 1.84 1.0000
25 -8 1 16 322
-6 3.34 1.06 1.0000
24 7 -1 152 308
155 -0.47 1.59 1.0000
22 7 -1 178 272
143 -3.28 0.84 1.0000
20 7 -1 373 277
507 -2.80 -1.42 1.0000
18 7 -1 236 232
47 13.11 5.67 1.0000
18 7 -1 235 195
42 13.11 5.67 1.0000
16 7 -1 623 260
1042 -15.85 3.19 1.0000
16 7 -1 890 206
1445 -15.85 3.19 1.0000
14 7 -1 133 144
141 18.22 -2.11 1.0000
14 7 -1 254 176
36 18.22 -2.11 1.0000
12 7 -1 5155 312
3872 -4.44 -7.11 1.0000
12 7 -1 4459 202
3272 -4.44 -7.11 1.0000
12 7 -1 5740 258
4381 -4.44 -7.11 1.0000
10 7 -1 2907 156
242 -25.59 -21.29 1.0000
10 7 -1 4197 96
699 -25.59 -21.29 1.0000
10 7 -1 3523 117
441 -25.59 -21.29 1.0000
8 7 -1 8491 343
7435 34.06 -20.75 1.0000
8 7 -1 8387 391
7323 34.06 -20.75 1.0000
-8 -7 1 7708 362

6561	34.06	20.75	1.0000	
6	7	-1	45	84
321	-12.30	-7.25	1.0000	
6	7	-1	61	108
405	-12.30	-7.25	1.0000	
-6	-7	1	34	98
317	-12.30	7.25	1.0000	
4	7	-1	6544	350
7760	4.23	5.00	1.0000	
-4	-7	1	6391	350
7594	4.23	-5.00	1.0000	
2	7	-1	18555	1284
36214	61.45	-5.10	1.0000	
2	7	-1	12119	1260
26491	61.45	-5.10	1.0000	
0	7	-1	26938	960
23544	-11.23	9.74	1.0000	
0	-7	1	26109	986
22767	-11.23	-9.74	1.0000	
0	-7	1	27056	973
23655	-11.23	-9.74	1.0000	
0	-7	1	27361	959
23942	-11.23	-9.74	1.0000	
-2	7	-1	1917	175
1867	12.77	-34.00	1.0000	
-2	7	-1	1946	132
1920	12.77	-34.00	1.0000	
2	-7	1	1879	99
1918	12.77	34.00	1.0000	
2	-7	1	1933	134
2019	12.77	34.00	1.0000	
2	-7	1	1626	163
1408	12.77	34.00	1.0000	
-4	7	-1	4874	338
7221	23.29	-13.26	1.0000	
-4	7	-1	5222	378
7670	23.29	-13.26	1.0000	
4	-7	1	5169	367
7569	23.29	13.26	1.0000	
4	-7	1	5216	322
7629	23.29	13.26	1.0000	
4	-7	1	5286	344
7719	23.29	13.26	1.0000	
6	-7	1	3114	322

7134	-23.01	-11.64	1.0000	
6	-7	1	2617	304
6366	-23.01	-11.64	1.0000	
6	-7	1	3229	355
7310	-23.01	-11.64	1.0000	
8	-7	1	4880	201
3503	-15.67	27.48	1.0000	
8	-7	1	5045	243
3667	-15.67	27.48	1.0000	
10	-7	1	3332	377
6854	34.38	-11.16	1.0000	
12	-7	1	1037	323
4988	-31.51	-13.21	1.0000	
14	-7	1	1519	319
4064	20.70	-6.38	1.0000	
16	-7	1	1230	288
2010	7.25	-7.35	1.0000	
18	-7	1	398	314
1801	-18.45	-9.27	1.0000	
18	-7	1	551	248
2123	-18.45	-9.27	1.0000	
20	-7	1	400	195
880	6.30	7.01	1.0000	
20	-7	1	330	265
772	6.30	7.01	1.0000	
22	-7	1	335	278
228	-3.52	0.44	1.0000	
24	-7	1	284	312
428	-4.49	-4.75	1.0000	
25	6	-1	221	348
329	-1.95	-2.06	1.0000	
23	6	-1	50	289
26	1.01	3.05	1.0000	
21	6	-1	553	275
455	-3.21	-0.37	1.0000	
19	6	-1	129	241
226	-1.81	-3.73	1.0000	
19	6	-1	700	212
918	-1.81	-3.73	1.0000	
17	6	-1	490	198
735	-7.53	1.52	1.0000	
17	6	-1	568	257
835	-7.53	1.52	1.0000	
15	6	-1	1770	283

1722	2.81	-0.15	1.0000		
15	6	-1	1703	225	
1655	2.81	-0.15	1.0000		
13	6	-1	857	183	
934	-2.18	2.74	1.0000		
13	6	-1	1241	217	
1336	-2.18	2.74	1.0000		
11	6	-1	9029	469	
11078	-3.40	8.92	1.0000		
11	6	-1	8387	439	
10365	-3.40	8.92	1.0000		
11	6	-1	7801	514	
9712	-3.40	8.92	1.0000		
-11	-6	1	7648	470	
9541	-3.40	-8.92	1.0000		
9	6	-1	4603	461	
11456	15.31	42.44	1.0000		
9	6	-1	4902	494	
11966	15.31	42.44	1.0000		
9	6	-1	4273	535	
10885	15.31	42.44	1.0000		
-9	-6	1	3717	493	
9905	15.31	-42.44	1.0000		
7	6	-1	2930	333	
6820	-23.13	-11.23	1.0000		
7	6	-1	3816	376	
8150	-23.13	-11.23	1.0000		
-7	-6	1	2873	350	
6729	-23.13	11.23	1.0000		
5	6	-1	14789	926	
21905	-14.70	23.53	1.0000		
-5	-6	1	16018	932	
23410	-14.70	-23.53	1.0000		
3	6	-1	1822	155	
2659	-1.59	-11.50	1.0000		
-3	-6	1	1631	156	
2408	-1.59	11.50	1.0000		
1	6	-1	22478	1913	
42604	-33.56	-39.28	1.0000		
1	6	-1	31411	1931	
54685	-33.56	-39.28	1.0000		
-1	-6	1	23234	1914	
43667	-33.56	39.28	1.0000		
-1	-6	1	29005	1926	

51501 -33.56 39.28 1.0000
-1 6 -1 35184 1795
44948 -0.01 21.18 1.0000
1 -6 1 34750 1814
44457 -0.01 -21.18 1.0000
1 -6 1 36439 1795
46365 -0.01 -21.18 1.0000
1 -6 1 34531 1773
44209 -0.01 -21.18 1.0000
-3 6 -1 36974 1003
25582 -31.35 10.39 1.0000
-3 6 -1 34136 974
23215 -31.35 10.39 1.0000
3 -6 1 33833 959
22957 -31.35 -10.39 1.0000
3 -6 1 34905 994
23848 -31.35 -10.39 1.0000
3 -6 1 35903 976
24680 -31.35 -10.39 1.0000
-5 6 -1 7676 839
19597 44.85 7.35 1.0000
5 -6 1 7385 800
19137 44.85 -7.35 1.0000
5 -6 1 8088 838
20258 44.85 -7.35 1.0000
5 -6 1 8090 813
20262 44.85 -7.35 1.0000
7 -6 1 42 65
193 -11.97 -6.22 1.0000
7 -6 1 42 93
204 -11.97 -6.22 1.0000
9 -6 1 5372 324
5936 -4.56 -7.76 1.0000
9 -6 1 5122 279
5671 -4.56 -7.76 1.0000
11 -6 1 326 245
3043 29.89 15.03 1.0000
11 -6 1 270 189
2852 29.89 15.03 1.0000
13 -6 1 125 173
927 -31.36 11.35 1.0000
15 -6 1 1923 192
756 14.05 6.73 1.0000
17 -6 1 685 205

499 3.56 15.27 1.0000
17 -6 1 832 168
668 3.56 15.27 1.0000
19 -6 1 576 219
383 -5.65 -3.85 1.0000
21 -6 1 102 232
-178 8.76 -0.43 1.0000
23 -6 1 430 274
393 1.16 0.35 1.0000
25 -6 1 4 287
-238 -1.67 0.07 1.0000
26 5 -1 112 349
99 -0.59 -0.40 1.0000
24 5 -1 140 317
106 1.74 -0.20 1.0000
22 5 -1 1158 310
834 -6.44 -4.16 1.0000
20 5 -1 679 298
1175 6.79 2.36 1.0000
18 5 -1 327 247
350 1.67 2.24 1.0000
18 5 -1 344 167
368 1.67 2.24 1.0000
16 5 -1 1959 317
2064 8.95 -3.73 1.0000
16 5 -1 1666 228
1753 8.95 -3.73 1.0000
14 5 -1 217 191
208 2.03 1.44 1.0000
14 5 -1 33 131
25 2.03 1.44 1.0000
-14 -5 1 11 102
3 2.03 -1.44 1.0000
12 5 -1 1541 173
869 -2.36 -14.30 1.0000
12 5 -1 2079 234
1316 -2.36 -14.30 1.0000
-12 -5 1 1626 132
950 -2.36 14.30 1.0000
10 5 -1 3811 109
1395 6.34 -20.20 1.0000
10 5 -1 3292 158
1088 6.34 -20.20 1.0000
10 5 -1 3394 202

1147	6.34	-20.20	1.0000	
-10	-5	1	3477	160
1196	6.34	20.20	1.0000	
8	5	-1	7564	785
18007	-32.97	-24.27	1.0000	
8	5	-1	8254	755
19063	-32.97	-24.27	1.0000	
8	5	-1	7842	730
18435	-32.97	-24.27	1.0000	
-8	-5	1	6768	752
16768	-32.97	24.27	1.0000	
6	5	-1	7723	895
19749	45.11	-7.03	1.0000	
-6	-5	1	11637	929
25763	45.11	7.03	1.0000	
-6	-5	1	8138	901
20404	45.11	7.03	1.0000	
4	5	-1	10444	274
5472	21.29	-12.81	1.0000	
-4	-5	1	11529	284
6264	21.29	12.81	1.0000	
2	5	-1	32626	1939
48417	-34.61	17.35	1.0000	
-2	-5	1	33122	1940
49075	-34.61	-17.35	1.0000	
0	5	-1	11518	925
22197	93.31	21.52	1.0000	
0	-5	1	11611	926
22531	93.31	-21.52	1.0000	
0	-5	1	12300	934
23923	93.31	-21.52	1.0000	
-2	5	-1	16825	969
24413	-20.15	11.10	1.0000	
2	-5	1	15248	961
22506	-20.15	-11.10	1.0000	
-4	5	-1	9355	359
7965	-6.72	3.34	1.0000	
-4	5	-1	8544	331
7217	-6.72	3.34	1.0000	
-4	5	-1	9382	323
7990	-6.72	3.34	1.0000	
4	-5	1	8584	354
7263	-6.72	-3.34	1.0000	
4	-5	1	9004	333

7650 -6.72 -3.34 1.0000
4 -5 1 9278 323
7903 -6.72 -3.34 1.0000
-6 5 -1 11943 792
18703 -14.11 -24.58 1.0000
6 -5 1 12015 762
18794 -14.11 24.58 1.0000
6 -5 1 11472 758
18106 -14.11 24.58 1.0000
6 -5 1 12289 793
19140 -14.11 24.58 1.0000
8 -5 1 2375 101
350 -0.92 -26.05 1.0000
8 -5 1 2539 70
415 -0.92 -26.05 1.0000
10 -5 1 4025 299
5037 -25.44 3.57 1.0000
10 -5 1 3920 243
4908 -25.44 3.57 1.0000
12 -5 1 801 181
1124 24.39 -18.24 1.0000
14 -5 1 1600 250
2053 -5.97 -23.10 1.0000
16 -5 1 625 146
829 -10.79 -4.57 1.0000
16 -5 1 663 208
879 -10.79 -4.57 1.0000
18 -5 1 1276 247
933 11.39 -4.73 1.0000
20 -5 1 124 230
161 -13.02 -3.60 1.0000
22 -5 1 638 258
489 -1.84 2.40 1.0000
24 -5 1 27 267
-117 2.83 3.52 1.0000
26 -5 1 335 334
526 -3.82 -3.69 1.0000
25 4 -1 6 332
18 -2.00 2.09 1.0000
23 4 -1 98 292
139 -1.12 1.26 1.0000
21 4 -1 732 307
792 -1.80 4.57 1.0000
19 4 -1 140 261

```
173    -4.58     2.74  1.0000
  17    4   -1      585      208
1308    -1.15   -11.05  1.0000
  17    4   -1      629      296
1374    -1.15   -11.05  1.0000
  15    4   -1      288      150
19   -12.26    12.01  1.0000
  15    4   -1      241      203
51   -12.26    12.01  1.0000
 -15   -4    1      392      117
-102   -12.26   -12.01  1.0000
  13    4   -1      577      197
911    -5.94    -1.08  1.0000
  13    4   -1      197      138
402    -5.94    -1.08  1.0000
 -13   -4    1      214      111
424    -5.94     1.08  1.0000
  11    4   -1      517      193
2077    19.36    23.20  1.0000
  11    4   -1      524      240
2097    19.36    23.20  1.0000
 -11   -4    1      536      167
2128    19.36   -23.20  1.0000
   9    4   -1     7333      249
4517   -25.47    17.62  1.0000
   9    4   -1     7471      289
4635   -25.47    17.62  1.0000
   9    4   -1     7140      211
4352   -25.47    17.62  1.0000
  -9   -4    1     7418      237
4606   -25.47   -17.62  1.0000
   7    4   -1     6433      405
9167    23.16    12.94  1.0000
   7    4   -1     5067      346
7436    23.16    12.94  1.0000
   7    4   -1     5459      375
7938    23.16    12.94  1.0000
  -7   -4    1     5643      375
8205    23.16   -12.94  1.0000
  -7   -4    1     6419      402
9183    23.16   -12.94  1.0000
   5    4   -1     8539      361
7198    -6.90    -3.94  1.0000
  -5   -4    1    10600      370
```

9091	-6.90	3.94	1.0000	
3	4	-1	15598	1317
29880	-60.15	-21.20	1.0000	
3	4	-1	20357	1328
36801	-60.15	-21.20	1.0000	
-3	-4	1	17126	1320
32208	-60.15	21.20	1.0000	
1	4	-1	1151	736
15510	79.54	3.66	1.0000	
-1	-4	1	2902	746
20966	79.54	-3.66	1.0000	
-1	4	-1	95519	1631
41556	-79.11	-59.59	1.0000	
-1	4	-1	91401	1616
38812	-79.11	-59.59	1.0000	
-1	4	-1	95461	1644
41517	-79.11	-59.59	1.0000	
1	-4	1	96253	1631
41995	-79.11	59.59	1.0000	
1	-4	1	94341	1617
40715	-79.11	59.59	1.0000	
1	-4	1	95972	1642
41806	-79.11	59.59	1.0000	
-3	4	-1	20410	1491
36070	46.14	-18.87	1.0000	
-3	4	-1	20445	1498
36118	46.14	-18.87	1.0000	
-3	4	-1	22855	1516
39362	46.14	-18.87	1.0000	
3	-4	1	22556	1515
38979	46.14	18.87	1.0000	
3	-4	1	22398	1499
38768	46.14	18.87	1.0000	
3	-4	1	21069	1492
36979	46.14	18.87	1.0000	
-5	4	-1	9315	247
4897	20.05	11.97	1.0000	
-5	4	-1	9356	218
4927	20.05	11.97	1.0000	
5	-4	1	8995	220
4668	20.05	-11.97	1.0000	
5	-4	1	9409	218
4968	20.05	-11.97	1.0000	
5	-4	1	9288	248

4880	20.05	-11.97	1.0000	
-7	4	-1	4828	288
5604	4.13	-3.33	1.0000	
-7	4	-1	4861	254
5640	4.13	-3.33	1.0000	
7	-4	1	5004	254
5793	4.13	3.33	1.0000	
7	-4	1	4694	256
5459	4.13	3.33	1.0000	
7	-4	1	4965	292
5751	4.13	3.33	1.0000	
9	-4	1	942	230
3489	33.69	-0.34	1.0000	
11	-4	1	1099	269
3912	-23.99	10.09	1.0000	
13	-4	1	4655	504
9738	17.67	21.66	1.0000	
15	-4	1	955	175
498	-5.73	17.54	1.0000	
17	-4	1	1268	245
1309	-0.02	0.50	1.0000	
19	-4	1	969	329
1864	7.21	7.87	1.0000	
21	-4	1	107	235
136	-1.06	-1.57	1.0000	
23	-4	1	237	271
315	1.48	-2.68	1.0000	
25	-4	1	15	302
-5	3.16	-0.96	1.0000	
26	3	-1	1	372
-301	0.93	0.30	1.0000	
24	3	-1	418	354
691	-4.64	-2.19	1.0000	
22	3	-1	272	299
534	6.84	-2.39	1.0000	
20	3	-1	736	260
605	1.25	-2.14	1.0000	
18	3	-1	123	225
335	-6.50	-0.47	1.0000	
18	3	-1	162	188
399	-6.50	-0.47	1.0000	
16	3	-1	2166	179
743	26.54	5.83	1.0000	
16	3	-1	1996	201

613	26.54	5.83	1.0000	
14	3	-1	1469	164
1014	-1.74	-5.34	1.0000	
14	3	-1	1304	191
878	-1.74	-5.34	1.0000	
-14	-3	1	1094	135
707	-1.74	5.34	1.0000	
12	3	-1	6517	360
5830	-7.88	-3.69	1.0000	
12	3	-1	6546	316
5857	-7.88	-3.69	1.0000	
-12	-3	1	6802	298
6101	-7.88	3.69	1.0000	
10	3	-1	3948	426
9703	11.84	-28.76	1.0000	
10	3	-1	4494	493
10549	11.84	-28.76	1.0000	
10	3	-1	3939	450
9688	11.84	-28.76	1.0000	
-10	-3	1	4226	443
10140	11.84	28.76	1.0000	
8	3	-1	92	124
566	-13.48	-5.32	1.0000	
8	3	-1	154	76
730	-13.48	-5.32	1.0000	
8	3	-1	228	138
898	-13.48	-5.32	1.0000	
-8	-3	1	56	114
425	-13.48	5.32	1.0000	
-8	-3	1	147	106
700	-13.48	5.32	1.0000	
6	3	-1	34876	991
23907	-31.41	-10.06	1.0000	
6	3	-1	34699	1007
23760	-31.41	-10.06	1.0000	
6	3	-1	36389	972
25172	-31.41	-10.06	1.0000	
-6	-3	1	35902	1008
24770	-31.41	10.06	1.0000	
-6	-3	1	35555	989
24480	-31.41	10.06	1.0000	
4	3	-1	21423	1611
37405	46.39	18.53	1.0000	
-4	-3	1	26031	1613

43517	46.39	-18.53	1.0000	
2	3	-1	61253	1884
42492	40.74	48.58	1.0000	
2	3	-1	68352	1885
48652	40.74	48.58	1.0000	
-2	-3	1	72863	1888
52705	40.74	-48.58	1.0000	
-2	-3	1	65544	1887
46313	40.74	-48.58	1.0000	
0	3	-1	38056	1478
36006	-48.19	-7.12	1.0000	
0	-3	1	39165	1478
37164	-48.19	7.12	1.0000	
0	-3	1	40484	1481
38504	-48.19	7.12	1.0000	
-2	3	-1	19194	2110
52311	78.24	67.31	1.0000	
-2	3	-1	21124	2121
55786	78.24	67.31	1.0000	
-2	3	-1	18699	2104
51404	78.24	67.31	1.0000	
2	-3	1	20216	2110
54261	78.24	-67.31	1.0000	
2	-3	1	19043	2104
52133	78.24	-67.31	1.0000	
2	-3	1	20407	2120
54603	78.24	-67.31	1.0000	
-4	3	-1	11414	959
23620	-59.14	22.17	1.0000	
-4	3	-1	11883	978
24363	-59.14	22.17	1.0000	
4	-3	1	11874	978
24286	-59.14	-22.17	1.0000	
4	-3	1	11242	958
23285	-59.14	-22.17	1.0000	
4	-3	1	12020	960
24515	-59.14	-22.17	1.0000	
-6	3	-1	1337	140
1912	-1.47	9.75	1.0000	
-6	3	-1	1592	116
2222	-1.47	9.75	1.0000	
6	-3	1	1472	115
2090	-1.47	-9.75	1.0000	
6	-3	1	1426	110

2033 -1.47 -9.75 1.0000
6 -3 1 1335 147
1922 -1.47 -9.75 1.0000
-8 3 -1 3982 134
2374 -11.99 -4.11 1.0000
8 -3 1 3741 174
2190 -11.99 4.11 1.0000
-10 3 -1 3224 99
821 -18.47 16.33 1.0000
10 -3 1 2986 132
704 -18.47 -16.33 1.0000
12 -3 1 2585 220
2243 2.17 -18.78 1.0000
14 -3 1 1898 269
2886 2.55 -11.32 1.0000
16 -3 1 904 181
248 2.48 -19.93 1.0000
18 -3 1 206 203
260 -7.13 -0.45 1.0000
20 -3 1 102 214
52 0.73 2.70 1.0000
22 -3 1 12 234
-31 -1.97 -2.32 1.0000
24 -3 1 232 283
317 -2.68 0.42 1.0000
26 -3 1 32 315
31 -0.06 2.27 1.0000
25 2 -1 15 353
-133 1.94 2.77 1.0000
23 2 -1 71 268
86 -1.72 3.56 1.0000
21 2 -1 623 267
646 -1.73 0.91 1.0000
19 2 -1 296 234
241 0.49 -1.38 1.0000
17 2 -1 736 256
1627 -9.35 12.00 1.0000
17 2 -1 686 266
1547 -9.35 12.00 1.0000
15 2 -1 1431 187
291 -5.83 -19.08 1.0000
15 2 -1 1855 152
514 -5.83 -19.08 1.0000
13 2 -1 2308 238

3545	6.44	7.87	1.0000	
13	2	-1	2475	294
3751	6.44	7.87	1.0000	
-13	-2	1	2419	225
3682	6.44	-7.87	1.0000	
11	2	-1	2662	415
7705	-27.50	18.40	1.0000	
11	2	-1	2820	371
7977	-27.50	18.40	1.0000	
-11	-2	1	2853	366
8018	-27.50	-18.40	1.0000	
9	2	-1	1068	246
4768	30.89	-6.44	1.0000	
9	2	-1	1103	224
4842	30.89	-6.44	1.0000	
9	2	-1	1065	280
4763	30.89	-6.44	1.0000	
-9	-2	1	1077	239
4790	30.89	6.44	1.0000	
7	2	-1	1733	91
1637	12.93	33.72	1.0000	
7	2	-1	1531	146
1199	12.93	33.72	1.0000	
-7	-2	1	1625	110
1295	12.93	-33.72	1.0000	
-7	-2	1	1532	134
1080	12.93	-33.72	1.0000	
5	2	-1	15535	908
22812	-20.26	-10.64	1.0000	
-5	-2	1	14270	903
21273	-20.26	10.64	1.0000	
3	2	-1	22320	2341
57899	78.48	-66.98	1.0000	
3	2	-1	19887	2344
53570	78.48	-66.98	1.0000	
-3	-2	1	22190	2346
57570	78.48	66.98	1.0000	
-3	-2	1	28301	2344
67971	78.48	66.98	1.0000	
1	2	-1	30944	110
1975	-108.62	-40.59	1.0000	
1	2	-1	32378	114
2365	-108.62	-40.59	1.0000	
-1	-2	1	32333	114

2344	-108.62	40.59	1.0000	
-1	-2	1	31251	110
2049	-108.62	40.59	1.0000	
-1	2	-1	19584	74
1218	90.60	-27.25	1.0000	
-1	2	-1	19080	62
1083	90.60	-27.25	1.0000	
-1	2	-1	18935	68
1045	90.60	-27.25	1.0000	
1	-2	1	19351	68
1121	90.60	27.25	1.0000	
1	-2	1	20199	75
1351	90.60	27.25	1.0000	
1	-2	1	19381	62
1129	90.60	27.25	1.0000	
-3	2	-1	57970	1705
40055	40.37	-49.78	1.0000	
-3	2	-1	64065	1721
45352	40.37	-49.78	1.0000	
-3	2	-1	60252	1708
42033	40.37	-49.78	1.0000	
3	-2	1	60890	1708
42486	40.37	49.78	1.0000	
3	-2	1	63573	1721
44818	40.37	49.78	1.0000	
3	-2	1	63395	1707
44663	40.37	49.78	1.0000	
-5	2	-1	30677	1806
45621	-33.70	-17.23	1.0000	
-5	2	-1	30586	1789
45509	-33.70	-17.23	1.0000	
5	-2	1	30690	1789
45588	-33.70	17.23	1.0000	
5	-2	1	30052	1807
44806	-33.70	17.23	1.0000	
5	-2	1	29670	1782
44336	-33.70	17.23	1.0000	
-7	2	-1	12712	1055
26629	59.15	4.09	1.0000	
-7	2	-1	11567	1028
24854	59.15	4.09	1.0000	
7	-2	1	11171	1027
24096	59.15	-4.09	1.0000	
7	-2	1	12905	1059

26788	59.15	-4.09	1.0000	
-9	2	-1	1413	89
754	-1.62	-10.72	1.0000	
9	-2	1	1503	138
812	-1.62	10.72	1.0000	
-11	2	-1	196	103
885	13.44	-7.35	1.0000	
11	-2	1	186	151
862	13.44	7.35	1.0000	
13	-2	1	3953	327
5050	-1.80	11.02	1.0000	
15	-2	1	1026	206
1113	-4.40	16.70	1.0000	
17	-2	1	1250	302
2261	0.01	10.66	1.0000	
19	-2	1	311	221
477	0.70	-4.53	1.0000	
21	-2	1	726	266
876	3.20	-0.97	1.0000	
23	-2	1	64	260
90	1.31	0.71	1.0000	
25	-2	1	5	297
-46	0.43	-1.83	1.0000	
26	1	-1	3	335
-161	-1.43	-0.47	1.0000	
24	1	-1	378	312
565	1.77	-3.37	1.0000	
22	1	-1	274	267
202	-3.14	0.84	1.0000	
20	1	-1	753	261
806	-2.41	-0.21	1.0000	
18	1	-1	274	273
1488	21.11	-1.84	1.0000	
18	1	-1	262	254
1457	21.11	-1.84	1.0000	
16	1	-1	979	173
218	-16.37	1.83	1.0000	
16	1	-1	1154	144
313	-16.37	1.83	1.0000	
14	1	-1	530	236
1730	12.92	10.98	1.0000	
14	1	-1	506	186
1685	12.92	10.98	1.0000	
12	1	-1	3440	356

6288	23.12	-7.15	1.0000	
12	1	-1	3174	301
5912	23.12	-7.15	1.0000	
-12	-1	1	3061	296
5749	23.12	7.15	1.0000	
10	1	-1	14146	428
8296	-48.77	10.59	1.0000	
10	1	-1	14779	389
8846	-48.77	10.59	1.0000	
-10	-1	1	14565	387
8662	-48.77	-10.59	1.0000	
8	1	-1	13704	434
9345	17.73	-11.41	1.0000	
8	1	-1	13636	404
9288	17.73	-11.41	1.0000	
8	1	-1	13482	391
9160	17.73	-11.41	1.0000	
-8	-1	1	13597	402
9264	17.73	11.41	1.0000	
-8	-1	1	13741	431
9384	17.73	11.41	1.0000	
6	1	-1	34961	1817
44669	0.02	-21.13	1.0000	
6	1	-1	34197	1798
43804	0.02	-21.13	1.0000	
-6	-1	1	36888	1816
46844	0.02	21.13	1.0000	
-6	-1	1	35922	1800
45754	0.02	21.13	1.0000	
4	1	-1	89764	1568
37793	-79.28	59.23	1.0000	
4	1	-1	93591	1578
40333	-79.28	59.23	1.0000	
-4	-1	1	93363	1570
40233	-79.28	-59.23	1.0000	
-4	-1	1	92689	1577
39784	-79.28	-59.23	1.0000	
2	1	-1	18295	68
862	90.63	27.12	1.0000	
2	1	-1	19206	71
1089	90.63	27.12	1.0000	
-2	-1	1	18817	67
1020	90.63	-27.12	1.0000	
-2	-1	1	19440	70

1185 90.63 -27.12 1.0000
-2 1 -1 31243 118
2099 -108.22 40.30 1.0000
-2 1 -1 32723 112
2511 -108.22 40.30 1.0000
-2 1 -1 32859 115
2550 -108.22 40.30 1.0000
2 -1 1 33301 115
2688 -108.22 -40.30 1.0000
2 -1 1 31513 120
2180 -108.22 -40.30 1.0000
2 -1 1 33375 115
2710 -108.22 -40.30 1.0000
-4 1 -1 712 547
13561 79.04 -4.33 1.0000
-4 1 -1 642 560
13231 79.04 -4.33 1.0000
4 -1 1 571 560
12900 79.04 4.33 1.0000
4 -1 1 888 547
14338 79.04 4.33 1.0000
-6 1 -1 23466 1723
44611 -33.22 40.85 1.0000
-6 1 -1 21509 1699
41882 -33.22 40.85 1.0000
6 -1 1 21778 1699
42241 -33.22 -40.85 1.0000
6 -1 1 22046 1722
42616 -33.22 -40.85 1.0000
-8 1 -1 20307 722
17878 -45.47 -2.82 1.0000
-8 1 -1 20082 754
17651 -45.47 -2.82 1.0000
8 -1 1 20135 757
17695 -45.47 2.82 1.0000
8 -1 1 19875 722
17432 -45.47 2.82 1.0000
-10 1 -1 3190 325
7426 26.10 4.72 1.0000
10 -1 1 2891 373
6952 26.10 -4.72 1.0000
-12 1 -1 14954 897
21232 -23.40 15.13 1.0000
12 -1 1 15976 957

22490 -23.40 -15.13 1.0000
14 -1 1 1144 240
2141 2.20 -10.96 1.0000
16 -1 1 647 168
282 15.81 -6.37 1.0000
18 -1 1 17 202
193 -7.11 -5.26 1.0000
20 -1 1 85 217
60 1.10 0.76 1.0000
22 -1 1 144 251
230 -1.54 4.18 1.0000
24 -1 1 345 292
288 -1.38 -1.17 1.0000
26 -1 1 5 303
-352 1.79 -0.63 1.0000
25 0 -1 0 313
-69 -0.25 -0.25 1.0000
23 0 -1 33 258
-215 3.30 3.76 1.0000
21 0 -1 355 258
461 0.84 2.22 1.0000
19 0 -1 77 222
163 -3.88 0.42 1.0000
19 0 -1 172 176
295 -3.88 0.42 1.0000
17 0 -1 704 215
1125 -2.33 -8.41 1.0000
17 0 -1 607 237
997 -2.33 -8.41 1.0000
15 0 -1 924 206
995 -0.10 2.77 1.0000
15 0 -1 1267 187
1351 -0.10 2.77 1.0000
13 0 -1 1013 106
327 -14.51 -8.87 1.0000
13 0 -1 592 132
69 -14.51 -8.87 1.0000
11 0 -1 6832 377
8302 20.22 -16.69 1.0000
11 0 -1 6627 420
8062 20.22 -16.69 1.0000
9 0 -1 9767 654
14668 5.30 21.44 1.0000
9 0 -1 11200 667

```
16424     5.30    21.44  1.0000
    9    0   -1    11360      700
16619     5.30    21.44  1.0000
   -9    0    1    10986      665
16169     5.30   -21.44  1.0000
    7    0   -1    27063      965
23668   -10.95    -9.70  1.0000
    7    0   -1    27379      986
23965   -10.95    -9.70  1.0000
   -7    0    1    27354      983
23940   -10.95     9.70  1.0000
   -7    0    1    27144      965
23743   -10.95     9.70  1.0000
    5    0   -1    12647      987
24578    93.26   -21.44  1.0000
    5    0   -1    12379      976
24049    93.26   -21.44  1.0000
   -5    0    1    12184      975
23517    93.26    21.44  1.0000
   -5    0    1    12935      986
24998    93.26    21.44  1.0000
    3    0   -1    40359     1493
38355   -48.15     7.16  1.0000
    3    0   -1    38353     1490
36318   -48.15     7.16  1.0000
   -3    0    1    40580     1492
38548   -48.15    -7.16  1.0000
   -3    0    1    39029     1490
36974   -48.15    -7.16  1.0000
   -3    0   -1    37907     1426
35687   -48.08     7.56  1.0000
   -3    0   -1    37465     1416
35239   -48.08     7.56  1.0000
    3    0    1    39188     1417
36954   -48.08    -7.56  1.0000
    3    0    1    37514     1426
35259   -48.08    -7.56  1.0000
   -5    0   -1    11025      861
21471    92.74   -22.71  1.0000
   -5    0   -1    10957      879
21328    92.74   -22.71  1.0000
    5    0    1    11429      861
22176    92.74    22.71  1.0000
    5    0    1    10831      880
```

```
20921    92.74    22.71  1.0000
  -7    0   -1    26353       923
22252   -11.57   -11.92  1.0000
  -7    0   -1    27646       952
23446   -11.57   -11.92  1.0000
   7    0    1    27824       956
23609   -11.57    11.92  1.0000
   7    0    1    26579       924
22459   -11.57    11.92  1.0000
  -9    0   -1     9537       618
14629     5.36    22.54  1.0000
   9    0    1    10060       661
15285     5.36   -22.54  1.0000
 -11    0   -1     6347       354
7875    18.83   -17.12  1.0000
  11    0    1     6413       414
7949    18.83    17.12  1.0000
 -13    0   -1     1125        95
422   -14.30    -9.17  1.0000
  13    0    1      889       141
243   -14.30     9.17  1.0000
  15    0    1     1096       207
1123     0.27    -1.81  1.0000
  17    0    1      636       235
1024    -1.85     8.88  1.0000
  19    0    1      207       242
341    -3.66    -0.84  1.0000
  21    0    1        6       238
-98     0.75    -2.00  1.0000
  23    0    1      139       277
129     3.48    -3.84  1.0000
  25    0    1        0       285
-245    -0.12    -0.03  1.0000
  26   -1   -1        0       317
5     1.80     0.61  1.0000
  24   -1   -1      210       300
162    -1.54     1.29  1.0000
  22   -1   -1       27       244
-30    -1.50    -4.20  1.0000
  20   -1   -1      373       189
324     1.24    -0.47  1.0000
  20   -1   -1      566       243
505     1.24    -0.47  1.0000
  18   -1   -1       25       156
```

238	-7.23		5.99	1.0000
18	-1	-1	20	195
219	-7.23		5.99	1.0000
16	-1	-1	1074	148
757	15.99		5.81	1.0000
16	-1	-1	707	172
369	15.99		5.81	1.0000
14	-1	-1	1725	211
2934	3.11		11.11	1.0000
14	-1	-1	1469	257
2596	3.11		11.11	1.0000
12	-1	-1	16724	983
23637	-24.12		14.47	1.0000
12	-1	-1	16987	1029
23954	-24.12		14.47	1.0000
10	-1	-1	2842	363
7034	25.94		7.21	1.0000
10	-1	-1	2794	330
6959	25.94		7.21	1.0000
8	-1	-1	20445	742
18324	-47.78		-1.98	1.0000
8	-1	-1	20072	765
17942	-47.78		-1.98	1.0000
-8	1	1	19737	760
17585	-47.78		1.98	1.0000
6	-1	-1	24651	1802
45527	-33.53		39.10	1.0000
6	-1	-1	22619	1787
42735	-33.53		39.10	1.0000
-6	1	1	25411	1801
46544	-33.53		-39.10	1.0000
4	-1	-1	893	564
14471	79.53		-3.30	1.0000
4	-1	-1	614	566
13214	79.53		-3.30	1.0000
-4	1	1	677	566
13529	79.53		3.30	1.0000
2	-1	-1	31506	107
2093	-108.68		40.89	1.0000
2	-1	-1	31216	104
2016	-108.68		40.89	1.0000
-2	1	1	31601	104
2126	-108.68		-40.89	1.0000
-2	-1	-1	18183	54

823	90.56	27.44	1.0000	
-2	-1	-1	18162	63
818	90.56	27.44	1.0000	
2	1	1	18478	54
920	90.56	-27.44	1.0000	
2	1	1	18338	67
886	90.56	-27.44	1.0000	
-4	-1	-1	92643	1585
39603	-78.83	59.69	1.0000	
-4	-1	-1	92320	1601
39388	-78.83	59.69	1.0000	
4	1	1	96297	1586
42096	-78.83	-59.69	1.0000	
4	1	1	92036	1602
39251	-78.83	-59.69	1.0000	
-6	-1	-1	33083	1766
42929	1.28	-21.95	1.0000	
-6	-1	-1	36042	1793
46290	1.28	-21.95	1.0000	
6	1	1	34074	1793
44056	1.28	21.95	1.0000	
6	1	1	34881	1766
44973	1.28	21.95	1.0000	
-8	-1	-1	13635	449
9457	16.96	-11.24	1.0000	
-8	-1	-1	14623	413
10289	16.96	-11.24	1.0000	
8	1	1	13485	452
9339	16.96	11.24	1.0000	
8	1	1	14024	413
9792	16.96	11.24	1.0000	
-10	-1	-1	13768	354
8271	-48.52	9.14	1.0000	
10	1	1	13161	407
7740	-48.52	-9.14	1.0000	
-12	-1	-1	3565	307
6633	23.21	-8.29	1.0000	
12	1	1	3449	376
6468	23.21	8.29	1.0000	
14	1	1	469	235
1636	12.96	-11.21	1.0000	
16	1	1	693	173
81	-16.75	-1.55	1.0000	
18	1	1	165	260

1103 20.21 1.85 1.0000
20 1 1 474 249
516 -2.16 0.09 1.0000
22 1 1 14 243
-51 -3.21 -0.40 1.0000
24 1 1 88 286
194 1.84 3.59 1.0000
26 1 1 81 302
62 -1.47 0.47 1.0000
25 -2 -1 393 331
313 0.38 1.85 1.0000
23 -2 -1 3 258
-118 1.24 -0.88 1.0000
21 -2 -1 308 197
402 3.02 1.00 1.0000
21 -2 -1 488 251
607 3.02 1.00 1.0000
19 -2 -1 239 214
384 0.52 4.60 1.0000
19 -2 -1 275 178
429 0.52 4.60 1.0000
17 -2 -1 1866 324
3091 0.31 -10.81 1.0000
17 -2 -1 1810 255
3019 0.31 -10.81 1.0000
15 -2 -1 1283 229
1468 -4.87 -15.96 1.0000
15 -2 -1 1154 183
1309 -4.87 -15.96 1.0000
13 -2 -1 4078 330
5027 -1.68 -9.67 1.0000
13 -2 -1 4206 283
5170 -1.68 -9.67 1.0000
11 -2 -1 334 174
1130 12.47 -7.49 1.0000
11 -2 -1 294 138
1052 12.47 -7.49 1.0000
9 -2 -1 1593 147
1069 0.76 -9.85 1.0000
9 -2 -1 1631 122
1101 0.76 -9.85 1.0000
7 -2 -1 12149 1075
26632 61.14 5.31 1.0000
7 -2 -1 11212 1056

25141 61.14 5.31 1.0000
-7 2 1 12138 1071
26475 61.14 -5.31 1.0000
5 -2 -1 28785 1743
43657 -34.45 -17.51 1.0000
-5 2 1 28675 1740
43472 -34.45 17.51 1.0000
3 -2 -1 59139 1638
40768 40.77 -48.74 1.0000
-3 2 1 60218 1638
41601 40.77 48.74 1.0000
1 -2 -1 18314 64
895 90.59 -27.13 1.0000
-1 2 1 18427 65
895 90.59 27.13 1.0000
-1 -2 -1 30922 104
1996 -108.25 -40.66 1.0000
1 2 1 30585 104
1901 -108.25 40.66 1.0000
-3 -2 -1 18963 2043
52264 78.28 -67.88 1.0000
-3 -2 -1 19088 2059
52493 78.28 -67.88 1.0000
3 2 1 17457 2057
49370 78.28 67.88 1.0000
3 2 1 19523 2043
53190 78.28 67.88 1.0000
-5 -2 -1 13267 840
20298 -20.55 -11.73 1.0000
5 2 1 13257 841
20287 -20.55 11.73 1.0000
-7 -2 -1 1827 106
1795 11.90 35.05 1.0000
-7 -2 -1 1647 147
1412 11.90 35.05 1.0000
7 2 1 1825 161
1662 11.90 -35.05 1.0000
7 2 1 1908 109
1825 11.90 -35.05 1.0000
-9 -2 -1 1057 230
4862 31.47 -7.31 1.0000
9 2 1 1055 286
4863 31.47 7.31 1.0000
9 2 1 1124 237

5009 31.47 7.31 1.0000
-11 -2 -1 3240 369
8685 -27.62 18.06 1.0000
11 2 1 2785 433
7904 -27.62 -18.06 1.0000
-13 -2 -1 2349 202
3603 6.57 7.81 1.0000
13 2 1 2512 297
3805 6.57 -7.81 1.0000
15 2 1 1666 192
423 -6.00 18.59 1.0000
17 2 1 1124 304
2138 -8.61 -11.51 1.0000
19 2 1 257 220
184 0.62 2.10 1.0000
21 2 1 359 253
393 -1.35 -1.21 1.0000
23 2 1 21 255
-50 -1.82 -3.57 1.0000
25 2 1 16 310
-34 2.00 -2.80 1.0000
26 -3 -1 206 336
220 -0.26 -2.08 1.0000
24 -3 -1 259 298
355 -2.76 -0.56 1.0000
22 -3 -1 170 240
156 -2.00 2.36 1.0000
20 -3 -1 205 226
136 0.70 -2.47 1.0000
20 -3 -1 9 164
-24 0.70 -2.47 1.0000
18 -3 -1 54 156
27 -7.21 0.32 1.0000
18 -3 -1 366 216
476 -7.21 0.32 1.0000
16 -3 -1 787 129
176 2.46 19.99 1.0000
16 -3 -1 793 169
181 2.46 19.99 1.0000
14 -3 -1 1905 264
2901 2.48 11.42 1.0000
14 -3 -1 2003 221
3024 2.48 11.42 1.0000
12 -3 -1 2788 188

```
2473     2.47    19.90  1.0000
  12  -3  -1     2656      224
2335     2.47    19.90  1.0000
  10  -3  -1     2691      129
645  -18.58   14.46  1.0000
  10  -3  -1     2618      130
609  -18.58   14.46  1.0000
   8  -3  -1     4544      183
2663  -14.11   -3.05  1.0000
  -8   3   1     4318      178
2493  -14.11    3.05  1.0000
   6  -3  -1     1288      142
1970   -1.47   11.57  1.0000
  -6   3   1     1192      135
1860   -1.47  -11.57  1.0000
   4  -3  -1    12153     1003
24790  -60.10   21.59  1.0000
  -4   3   1    12065     1002
24587  -60.10  -21.59  1.0000
   2  -3  -1    18018     2053
50387   78.60   67.65  1.0000
  -2   3   1    19467     2056
53157   78.60  -67.65  1.0000
   0  -3  -1    37158     1392
34916  -48.13   -7.53  1.0000
   0   3   1    37231     1393
35021  -48.13    7.53  1.0000
  -2  -3  -1    56110     1551
38306   40.55   50.08  1.0000
  -2  -3  -1    58086     1565
40012   40.55   50.08  1.0000
   2   3   1    57297     1565
39428   40.55  -50.08  1.0000
  -4  -3  -1    23299     1589
40136   46.08   19.36  1.0000
  -4  -3  -1    24499     1615
41734   46.08   19.36  1.0000
   4   3   1    22122     1586
38543   46.08  -19.36  1.0000
   4   3   1    22667     1613
39274   46.08  -19.36  1.0000
   4   3   1    24275     1590
41419   46.08  -19.36  1.0000
  -6  -3  -1    34331     1001
```

23295 -31.13 -10.83 1.0000
-6 -3 -1 36522 970
25118 -31.13 -10.83 1.0000
6 3 1 37377 971
25838 -31.13 10.83 1.0000
6 3 1 33463 1001
22582 -31.13 10.83 1.0000
-8 -3 -1 167 82
824 -13.32 -7.20 1.0000
8 3 1 201 157
894 -13.32 7.20 1.0000
8 3 1 175 92
833 -13.32 7.20 1.0000
-10 -3 -1 4095 426
9859 11.90 -28.37 1.0000
10 3 1 4197 490
10021 11.90 28.37 1.0000
-12 -3 -1 7125 294
6341 -8.11 -4.07 1.0000
12 3 1 7008 377
6230 -8.11 4.07 1.0000
-14 -3 -1 1350 115
867 -2.18 -5.95 1.0000
14 3 1 1335 199
855 -2.18 5.95 1.0000
16 3 1 1437 189
236 26.15 -5.43 1.0000
18 3 1 120 217
331 -6.64 0.01 1.0000
20 3 1 392 241
289 0.81 2.37 1.0000
22 3 1 43 296
149 6.80 2.50 1.0000
24 3 1 756 353
1110 -4.58 2.24 1.0000
26 3 1 1 337
-141 0.90 -0.28 1.0000
25 -4 -1 349 305
295 3.31 0.73 1.0000
23 -4 -1 243 269
323 1.34 2.66 1.0000
23 -4 -1 32 223
59 1.34 2.66 1.0000
21 -4 -1 7 187

12 -1.09 1.45 1.0000

21 -4 -1 62 232

81 -1.09 1.45 1.0000

19 -4 -1 938 255

1808 7.17 -7.73 1.0000

19 -4 -1 1283 328

2277 7.17 -7.73 1.0000

17 -4 -1 1698 234

1695 -0.16 0.02 1.0000

17 -4 -1 1737 291

1734 -0.16 0.02 1.0000

15 -4 -1 1038 179

576 -5.80 -17.57 1.0000

15 -4 -1 917 135

455 -5.80 -17.57 1.0000

13 -4 -1 5379 495

10850 17.86 -21.84 1.0000

13 -4 -1 5083 534

10425 17.86 -21.84 1.0000

11 -4 -1 1228 269

3967 -23.31 -8.60 1.0000

11 -4 -1 1376 240

4232 -23.31 -8.60 1.0000

9 -4 -1 902 221

3353 33.90 2.13 1.0000

7 -4 -1 4796 293

5845 4.30 -4.97 1.0000

-7 4 1 4619 291

5649 4.30 4.97 1.0000

5 -4 -1 9532 244

4795 21.32 12.96 1.0000

-5 4 1 9578 245

4830 21.32 -12.96 1.0000

3 -4 -1 23834 1655

40725 46.32 -18.89 1.0000

-3 4 1 24754 1656

41963 46.32 18.89 1.0000

1 -4 -1 89281 1504

37522 -79.10 -59.32 1.0000

-1 -4 -1 649 538

13284 79.07 4.22 1.0000

1 4 1 485 536

12442 79.07 -4.22 1.0000

-3 -4 -1 11853 951

```
24400  -59.21  -22.60  1.0000
  -3  -4  -1   11401      972
23683  -59.21  -22.60  1.0000
  -3  -4  -1   11206      945
23372  -59.21  -22.60  1.0000
   3   4   1   11375      973
23703  -59.21   22.60  1.0000
  -5  -4  -1    8471      296
7168   -6.64   -3.05  1.0000
  -5  -4  -1    7934      339
6674   -6.64   -3.05  1.0000
   5   4   1    8230      340
6938   -6.64    3.05  1.0000
   5   4   1    8531      297
7215   -6.64    3.05  1.0000
  -7  -4  -1    5233      336
7723   23.68   13.29  1.0000
  -7  -4  -1    5213      381
7697   23.68   13.29  1.0000
   7   4   1    5369      386
7932   23.68  -13.29  1.0000
   7   4   1    5633      340
8271   23.68  -13.29  1.0000
  -9  -4  -1    6816      202
4073  -26.03   17.81  1.0000
   9   4   1    7038      280
4279  -26.03  -17.81  1.0000
   9   4   1    7031      210
4273  -26.03  -17.81  1.0000
 -11  -4  -1     592      143
2291   19.05   23.31  1.0000
  11   4   1     633      246
2397   19.05  -23.31  1.0000
 -13  -4  -1     409      104
706   -6.11   -1.16  1.0000
  13   4   1     334      188
601   -6.11    1.16  1.0000
 -15  -4  -1     215      101
213  -11.74   11.40  1.0000
  15   4   1     193      201
167  -11.74  -11.40  1.0000
  17   4   1     971      303
1842   -1.21   10.78  1.0000
  19   4   1      64      247
```

74 -4.44 -2.50 1.0000
21 4 1 223 280
236 -1.63 -4.07 1.0000
23 4 1 140 313
191 -1.01 -1.43 1.0000
25 4 1 60 341
102 -1.79 -2.12 1.0000
26 -5 -1 40 311
-355 -4.22 3.54 1.0000
24 -5 -1 180 283
80 2.77 -3.47 1.0000
22 -5 -1 352 251
234 -2.05 -2.62 1.0000
22 -5 -1 625 201
465 -2.05 -2.62 1.0000
20 -5 -1 132 228
201 -13.24 3.58 1.0000
20 -5 -1 130 178
192 -13.24 3.58 1.0000
18 -5 -1 1207 230
877 11.56 4.66 1.0000
18 -5 -1 1230 197
898 11.56 4.66 1.0000
16 -5 -1 646 168
873 -10.62 4.82 1.0000
16 -5 -1 657 212
888 -10.62 4.82 1.0000
14 -5 -1 1769 261
2287 -6.26 23.14 1.0000
14 -5 -1 1944 217
2527 -6.26 23.14 1.0000
12 -5 -1 876 200
1446 24.30 17.86 1.0000
10 -5 -1 3451 274
4326 -26.62 -2.98 1.0000
8 -5 -1 2236 103
320 -0.07 25.79 1.0000
6 -5 -1 11995 768
18504 -14.94 -23.73 1.0000
-6 5 1 11305 767
17634 -14.94 23.73 1.0000
4 -5 -1 8809 347
7438 -6.86 3.59 1.0000
-4 5 1 8731 347

7376	-6.86	-3.59	1.0000	
2	-5	-1	15265	964
22691	-20.52	11.41	1.0000	
-2	5	1	16547	970
24248	-20.52	-11.41	1.0000	
0	-5	-1	11208	903
21726	92.79	22.70	1.0000	
0	5	1	11548	906
22569	92.79	-22.70	1.0000	
-2	-5	-1	30785	1744
45662	-33.61	17.34	1.0000	
-2	-5	-1	27987	1757
42218	-33.61	17.34	1.0000	
2	5	1	29360	1762
43960	-33.61	-17.34	1.0000	
-4	-5	-1	8646	255
4405	20.17	-11.94	1.0000	
-4	-5	-1	9506	214
5026	20.17	-11.94	1.0000	
-4	-5	-1	8743	203
4475	20.17	-11.94	1.0000	
4	5	1	8697	253
4440	20.17	11.94	1.0000	
4	5	1	9364	211
4921	20.17	11.94	1.0000	
-6	-5	-1	7754	824
19688	44.68	-7.66	1.0000	
-6	-5	-1	7302	777
18963	44.68	-7.66	1.0000	
-6	-5	-1	7915	782
19944	44.68	-7.66	1.0000	
6	5	1	7568	780
19383	44.68	7.66	1.0000	
6	5	1	7290	777
18937	44.68	7.66	1.0000	
6	5	1	7323	821
18990	44.68	7.66	1.0000	
-8	-5	-1	7234	736
17646	-33.58	-24.26	1.0000	
8	5	1	7807	739
18536	-33.58	24.26	1.0000	
8	5	1	7621	792
18248	-33.58	24.26	1.0000	
-10	-5	-1	4078	118

1521	6.45	-20.60	1.0000	
10	5	1	4313	128
1666	6.45	20.60	1.0000	
10	5	1	3700	208
1294	6.45	20.60	1.0000	
-12	-5	-1	1899	114
1140	-2.37	-14.86	1.0000	
12	5	1	1690	200
979	-2.37	14.86	1.0000	
-14	-5	-1	350	98
338	1.89	1.42	1.0000	
14	5	1	294	198
281	1.89	-1.42	1.0000	
16	5	1	1729	288
1771	8.58	3.19	1.0000	
18	5	1	429	264
461	1.62	-1.89	1.0000	
20	5	1	913	318
1464	6.62	-2.36	1.0000	
22	5	1	78	277
-26	-6.49	4.08	1.0000	
24	5	1	3	305
-79	1.55	0.24	1.0000	
26	5	1	122	354
109	-0.43	0.38	1.0000	
25	-6	-1	71	314
108	-1.62	-0.53	1.0000	
25	-6	-1	4	291
-64	-1.62	-0.53	1.0000	
23	-6	-1	97	235
81	1.10	-0.26	1.0000	
23	-6	-1	146	271
127	1.10	-0.26	1.0000	
21	-6	-1	3	178
73	8.99	0.48	1.0000	
21	-6	-1	5	240
65	8.99	0.48	1.0000	
19	-6	-1	567	183
390	-5.75	3.54	1.0000	
19	-6	-1	584	215
405	-5.75	3.54	1.0000	
17	-6	-1	890	184
741	3.40	-15.14	1.0000	
17	-6	-1	950	226

809 3.40 -15.14 1.0000
15 -6 -1 1761 151
660 14.08 -6.49 1.0000
15 -6 -1 1964 191
790 14.08 -6.49 1.0000
13 -6 -1 138 172
859 -31.22 -10.96 1.0000
11 -6 -1 501 261
3582 30.23 -14.70 1.0000
9 -6 -1 5316 324
5856 -3.83 7.09 1.0000
7 -6 -1 34 99
293 -12.16 6.90 1.0000
5 -6 -1 7518 798
19388 44.98 7.46 1.0000
-5 6 1 6780 797
18198 44.98 -7.46 1.0000
3 -6 -1 34593 1000
23583 -31.24 10.52 1.0000
-3 6 1 36112 1009
24843 -31.24 -10.52 1.0000
1 -6 -1 30860 1672
40366 1.37 21.89 1.0000
1 -6 -1 32562 1664
42309 1.37 21.89 1.0000
-1 -6 -1 22605 1727
43330 -33.40 -40.62 1.0000
-1 -6 -1 22537 1712
43235 -33.40 -40.62 1.0000
1 6 1 21904 1727
42370 -33.40 40.62 1.0000
-3 -6 -1 1597 133
2233 -1.35 -9.77 1.0000
3 6 1 1876 133
2554 -1.35 9.77 1.0000
-5 -6 -1 11667 735
18433 -14.28 24.83 1.0000
-5 -6 -1 11177 783
17808 -14.28 24.83 1.0000
-5 -6 -1 12170 747
19070 -14.28 24.83 1.0000
5 6 1 11340 783
18017 -14.28 -24.83 1.0000
5 6 1 11405 744

18100 -14.28 -24.83 1.0000
-7 -6 -1 3306 310
7408 -23.05 -11.45 1.0000
-7 -6 -1 2901 297
6790 -23.05 -11.45 1.0000
7 6 1 2972 305
6896 -23.05 11.45 1.0000
7 6 1 3012 299
6958 -23.05 11.45 1.0000
7 6 1 2677 361
6440 -23.05 11.45 1.0000
-9 -6 -1 4176 458
10727 15.47 42.52 1.0000
-9 -6 -1 4276 471
10904 15.47 42.52 1.0000
9 6 1 3973 526
10377 15.47 -42.52 1.0000
9 6 1 4300 461
10956 15.47 -42.52 1.0000
9 6 1 4517 460
11332 15.47 -42.52 1.0000
-11 -6 -1 8607 447
10687 -3.56 9.25 1.0000
11 6 1 8012 525
10022 -3.56 -9.25 1.0000
-13 -6 -1 762 114
840 -2.07 2.78 1.0000
13 6 1 711 199
783 -2.07 -2.78 1.0000
-15 -6 -1 1533 154
1499 2.52 -0.22 1.0000
15 6 1 1859 287
1822 2.52 0.22 1.0000
17 6 1 370 244
608 -7.58 -0.93 1.0000
21 6 1 635 268
531 -3.21 0.41 1.0000
23 6 1 13 278
-58 1.16 -2.86 1.0000
25 6 1 11 322
-259 -1.96 2.07 1.0000
24 -7 -1 191 259
318 -4.82 4.63 1.0000
24 -7 -1 153 316

```
262    -4.82     4.63  1.0000
  22   -7   -1        58       250
16    -3.74    -0.69  1.0000
  22   -7   -1        19       218
-23    -3.74    -0.69  1.0000
  20   -7   -1       247       231
630     6.40    -6.82  1.0000
  20   -7   -1       421       203
902     6.40    -6.82  1.0000
  18   -7   -1       446       265
1930   -18.78     9.19  1.0000
  18   -7   -1       407       315
1846   -18.78     9.19  1.0000
  16   -7   -1      1190       284
1939     7.28     7.11  1.0000
  16   -7   -1      1148       246
1884     7.28     7.11  1.0000
  14   -7   -1      1757       330
4438    20.66     6.22  1.0000
  12   -7   -1      1028       320
4966   -31.63    12.95  1.0000
  10   -7   -1      4009       420
8061    34.28    11.78  1.0000
   8   -7   -1      5321       260
4073   -15.86   -26.61  1.0000
   6   -7   -1      2807       340
6582   -23.01    11.00  1.0000
   4   -7   -1      5990       410
8719    23.52   -12.91  1.0000
  -4    7    1      5944       421
8627    23.52    12.91  1.0000
   2   -7   -1      1518       145
963    11.99   -34.72  1.0000
   0   -7   -1     25506       870
21467   -11.21    11.90  1.0000
   0   -7   -1     24793       886
20811   -11.21    11.90  1.0000
   0    7    1     25650       889
21601   -11.21   -11.90  1.0000
  -2   -7   -1     13334      1049
27366    58.94    -4.04  1.0000
  -2   -7   -1     12114      1069
25496    58.94    -4.04  1.0000
   2    7    1     11517      1065
```

24703	58.94	4.04	1.0000	
-4	-7	-1	4708	239
5461	4.10	3.26	1.0000	
-4	-7	-1	5647	264
6469	4.10	3.26	1.0000	
-4	-7	-1	4115	306
4820	4.10	3.26	1.0000	
4	7	1	5333	260
6134	4.10	-3.26	1.0000	
4	7	1	4006	301
4702	4.10	-3.26	1.0000	
-6	-7	-1	68	69
352	-11.76	-5.91	1.0000	
-6	-7	-1	72	61
366	-11.76	-5.91	1.0000	
6	7	1	58	70
360	-11.76	5.91	1.0000	
-8	-7	-1	8480	321
7416	33.75	-20.55	1.0000	
-8	-7	-1	8305	302
7228	33.75	-20.55	1.0000	
8	7	1	7075	372
5874	33.75	20.55	1.0000	
8	7	1	8083	305
6960	33.75	20.55	1.0000	
8	7	1	8054	313
6929	33.75	20.55	1.0000	
-10	-7	-1	3776	82
516	-25.59	-21.77	1.0000	
-10	-7	-1	3017	106
262	-25.59	-21.77	1.0000	
10	7	1	3091	81
284	-25.59	21.77	1.0000	
10	7	1	3841	97
540	-25.59	21.77	1.0000	
10	7	1	2466	153
119	-25.59	21.77	1.0000	
-12	-7	-1	4935	195
3673	-5.02	-6.93	1.0000	
12	7	1	4818	303
3571	-5.02	6.93	1.0000	
-14	-7	-1	80	103
374	18.02	-2.02	1.0000	
14	7	1	143	173

119 18.02 2.02 1.0000
16 7 1 864 265
1432 -15.74 -2.97 1.0000
18 7 1 391 245
329 13.02 -5.83 1.0000
20 7 1 423 278
577 -3.00 1.30 1.0000
22 7 1 16 278
-10 -3.34 -0.70 1.0000
24 7 1 59 299
61 -0.50 -1.60 1.0000
25 -8 -1 110 333
186 3.18 -0.32 1.0000
25 -8 -1 93 290
164 3.18 -0.32 1.0000
23 -8 -1 201 286
276 1.58 -2.35 1.0000
23 -8 -1 71 247
116 1.58 -2.35 1.0000
21 -8 -1 345 219
182 -3.80 3.58 1.0000
21 -8 -1 340 235
178 -3.80 3.58 1.0000
19 -8 -1 124 197
106 11.96 -3.19 1.0000
19 -8 -1 123 218
93 11.96 -3.19 1.0000
17 -8 -1 1904 297
1812 -0.85 -0.87 1.0000
17 -8 -1 1895 252
1803 -0.85 -0.87 1.0000
15 -8 -1 1395 288
2469 -17.45 -5.78 1.0000
13 -8 -1 764 242
1717 23.85 -13.86 1.0000
11 -8 -1 875 247
2951 -21.57 5.18 1.0000
9 -8 -1 8985 485
9763 -8.69 -0.77 1.0000
7 -8 -1 7876 359
6769 34.08 20.93 1.0000
5 -8 -1 6818 738
16911 -33.34 24.12 1.0000
3 -8 -1 93 117

594	-13.11	6.86	1.0000	
-3	8	1	35	111
383	-13.11	-6.86	1.0000	
1	-8	-1	13435	450
9339	16.77	11.23	1.0000	
1	-8	-1	13637	439
9509	16.77	11.23	1.0000	
1	-8	-1	13368	412
9283	16.77	11.23	1.0000	
-1	-8	-1	18620	730
16186	-45.52	2.75	1.0000	
-1	-8	-1	19998	713
17578	-45.52	2.75	1.0000	
1	8	1	20004	738
17594	-45.52	-2.75	1.0000	
-3	-8	-1	4051	148
2438	-11.75	4.36	1.0000	
3	8	1	3289	184
1854	-11.75	-4.36	1.0000	
3	8	1	4156	147
2518	-11.75	-4.36	1.0000	
-5	-8	-1	2994	100
601	-1.00	-26.20	1.0000	
5	8	1	2921	95
569	-1.00	26.20	1.0000	
-7	-8	-1	5510	193
4104	-15.64	27.74	1.0000	
-7	-8	-1	5626	219
4219	-15.64	27.74	1.0000	
7	8	1	5386	216
4017	-15.64	-27.74	1.0000	
-9	-8	-1	524	92
332	8.43	-4.61	1.0000	
-9	-8	-1	486	74
298	8.43	-4.61	1.0000	
9	8	1	359	77
178	8.43	4.61	1.0000	
9	8	1	232	152
72	8.43	4.61	1.0000	
9	8	1	349	84
169	8.43	4.61	1.0000	
-11	-8	-1	1699	198
1780	2.12	9.55	1.0000	
-11	-8	-1	1935	139

2030	2.12	9.55	1.0000	
11	8	1	2018	253
2106	2.12	-9.55	1.0000	
11	8	1	1861	158
1940	2.12	-9.55	1.0000	
-13	-8	-1	1770	203
3560	5.37	14.59	1.0000	
13	8	1	1871	334
3705	5.37	-14.59	1.0000	
-15	-8	-1	1084	117
530	-1.75	-9.72	1.0000	
15	8	1	503	202
147	-1.75	9.72	1.0000	
17	8	1	49	219
-115	2.57	5.49	1.0000	
19	8	1	44	237
244	-7.42	2.48	1.0000	
21	8	1	178	254
70	3.69	2.28	1.0000	
23	8	1	10	270
-64	-1.38	2.35	1.0000	
25	8	1	1	304
-76	0.49	-0.82	1.0000	
24	-9	-1	34	301
63	0.51	-1.74	1.0000	
24	-9	-1	6	285
20	0.51	-1.74	1.0000	
22	-9	-1	459	248
441	-0.38	0.05	1.0000	
22	-9	-1	453	256
435	-0.38	0.05	1.0000	
20	-9	-1	59	243
101	-7.68	3.15	1.0000	
20	-9	-1	92	198
-295	-7.68	3.15	1.0000	
18	-9	-1	111	205
-87	5.69	-7.13	1.0000	
18	-9	-1	461	185
121	5.69	-7.13	1.0000	
16	-9	-1	326	192
102	3.14	6.21	1.0000	
14	-9	-1	1234	171
365	-11.89	8.25	1.0000	
12	-9	-1	3197	194

1086	35.47	1.61	1.0000	
10	-9	-1	2141	299
4307	-20.00	-1.50	1.0000	
8	-9	-1	380	127
197	8.56	4.79	1.0000	
6	-9	-1	4138	510
10682	15.51	-42.57	1.0000	
4	-9	-1	6990	264
4256	-25.92	-17.65	1.0000	
2	-9	-1	962	281
4643	31.32	7.46	1.0000	
2	9	1	1652	149
941	-1.53	-10.74	1.0000	
-4	-9	-1	854	190
3301	33.69	0.05	1.0000	
4	9	1	763	238
3112	33.69	-0.05	1.0000	
4	9	1	894	189
3442	33.69	-0.05	1.0000	
-6	-9	-1	5512	296
6040	-4.92	-7.71	1.0000	
6	9	1	5438	290
5961	-4.92	7.71	1.0000	
-8	-9	-1	9386	448
10213	-8.41	0.40	1.0000	
8	9	1	9510	444
10354	-8.41	-0.40	1.0000	
8	9	1	8437	491
9228	-8.41	-0.40	1.0000	
-10	-9	-1	2935	217
3263	2.18	-2.03	1.0000	
-10	-9	-1	2538	168
2844	2.18	-2.03	1.0000	
10	9	1	2791	197
3111	2.18	2.03	1.0000	
10	9	1	3046	286
3381	2.18	2.03	1.0000	
10	9	1	2573	180
2881	2.18	2.03	1.0000	
-12	-9	-1	2234	254
2425	2.60	-5.73	1.0000	
-12	-9	-1	2749	175
2966	2.60	-5.73	1.0000	
12	9	1	2670	304

2891	2.60	5.73	1.0000	
12	9	1	3127	205
3370	2.60	5.73	1.0000	
-14	-9	-1	70	133
1011	-20.97	-4.39	1.0000	
14	9	1	18	222
507	-20.97	4.39	1.0000	
14	9	1	37	139
856	-20.97	4.39	1.0000	
16	9	1	384	223
-28	15.15	-7.68	1.0000	
18	9	1	218	261
170	-4.72	-3.47	1.0000	
20	9	1	515	314
860	-4.49	-4.43	1.0000	
22	9	1	25	297
-21	1.00	-4.18	1.0000	
24	9	1	40	326
-396	-5.15	1.86	1.0000	
23	-10	-1	14	266
-14	1.82	2.69	1.0000	
23	-10	-1	24	270
70	1.82	2.69	1.0000	
21	-10	-1	132	256
96	2.46	-1.37	1.0000	
21	-10	-1	104	233
71	2.46	-1.37	1.0000	
19	-10	-1	162	229
216	-2.09	-0.38	1.0000	
19	-10	-1	455	219
544	-2.09	-0.38	1.0000	
17	-10	-1	133	191
26	2.37	5.53	1.0000	
15	-10	-1	165	172
160	3.44	-6.78	1.0000	
13	-10	-1	2639	273
2577	-16.68	0.10	1.0000	
11	-10	-1	252	140
172	4.42	-3.01	1.0000	
9	-10	-1	2680	254
2990	2.23	1.98	1.0000	
7	-10	-1	2826	130
217	-25.59	21.37	1.0000	
5	-10	-1	3979	183

1454	6.40	20.69	1.0000	
3	-10	-1	3980	481
9658	11.90	28.24	1.0000	
1	-10	-1	13925	435
8389	-48.26	-9.25	1.0000	
1	10	1	3154	382
7404	26.26	4.77	1.0000	
3	10	1	2579	137
513	-18.58	16.22	1.0000	
5	10	1	4028	248
5094	-25.32	-3.85	1.0000	
5	10	1	3069	308
3891	-25.32	-3.85	1.0000	
7	10	1	3671	344
7505	34.48	11.52	1.0000	
7	10	1	3132	407
6629	34.48	11.52	1.0000	
9	10	1	1320	266
3079	-20.27	-1.81	1.0000	
9	10	1	1628	219
3561	-20.27	-1.81	1.0000	
-11	-10	-1	837	142
2027	17.15	0.52	1.0000	
11	10	1	727	288
1834	17.15	-0.52	1.0000	
11	10	1	906	182
2129	17.15	-0.52	1.0000	
-13	-10	-1	179	112
617	5.73	-8.62	1.0000	
13	10	1	32	225
283	5.73	8.62	1.0000	
13	10	1	139	124
540	5.73	8.62	1.0000	
-15	-10	-1	874	130
814	3.50	4.84	1.0000	
15	10	1	946	151
891	3.50	-4.84	1.0000	
15	10	1	827	255
773	3.50	-4.84	1.0000	
17	10	1	635	271
423	-2.20	6.51	1.0000	
19	10	1	67	265
-264	3.12	6.37	1.0000	
21	10	1	99	294

144	-1.44	3.02	1.0000	
23	10	1	41	329
100	1.24	3.38	1.0000	
24	-11	-1	4	315
15	-1.61	1.04	1.0000	
24	-11	-1	327	296
407	-1.61	1.04	1.0000	
22	-11	-1	24	255
-49	1.70	-3.92	1.0000	
22	-11	-1	24	257
-49	1.70	-3.92	1.0000	
20	-11	-1	64	236
124	-3.06	1.61	1.0000	
20	-11	-1	332	229
463	-3.06	1.61	1.0000	
18	-11	-1	17	204
-24	3.04	1.84	1.0000	
16	-11	-1	593	196
178	-2.99	-9.13	1.0000	
14	-11	-1	90	207
726	14.42	8.56	1.0000	
12	-11	-1	88	153
86	-4.85	0.75	1.0000	
10	-11	-1	1011	253
2289	17.05	-0.51	1.0000	
8	-11	-1	1638	221
1716	2.24	-9.61	1.0000	
6	-11	-1	8218	511
10214	-3.55	-9.08	1.0000	
4	-11	-1	570	217
2230	19.18	-23.30	1.0000	
2	-11	-1	3074	445
8424	-27.67	-18.38	1.0000	
0	-11	-1	5996	427
7501	18.77	17.34	1.0000	
2	11	1	135	164
727	13.30	-7.32	1.0000	
4	11	1	735	260
3175	-23.92	-9.90	1.0000	
6	11	1	399	206
3293	30.02	-15.33	1.0000	
6	11	1	218	277
2684	30.02	-15.33	1.0000	
8	11	1	691	196

2631 -21.74 5.45 1.0000
8 11 1 699 267
2647 -21.74 5.45 1.0000
10 11 1 160 98
95 4.18 -2.92 1.0000
10 11 1 35 157
-167 4.18 -2.92 1.0000
-12 -11 -1 156 100
485 -10.76 3.07 1.0000
12 11 1 152 131
482 -10.76 -3.07 1.0000
12 11 1 137 205
450 -10.76 -3.07 1.0000
-14 -11 -1 762 118
491 0.67 5.18 1.0000
14 11 1 415 237
222 0.67 -5.18 1.0000
14 11 1 502 138
287 0.67 -5.18 1.0000
16 11 1 44 257
-17 -4.98 -2.89 1.0000
18 11 1 540 285
482 -4.19 -3.09 1.0000
20 11 1 111 283
253 1.50 -4.50 1.0000
22 11 1 25 328
-45 -4.04 -1.68 1.0000
24 11 1 113 354
238 -1.90 -3.68 1.0000
23 -12 -1 161 287
161 0.13 -0.06 1.0000
23 -12 -1 579 323
580 0.13 -0.06 1.0000
21 -12 -1 430 252
287 5.33 -0.11 1.0000
21 -12 -1 38 254
-60 5.33 -0.11 1.0000
19 -12 -1 24 223
-97 -3.14 2.84 1.0000
17 -12 -1 680 221
515 -2.63 1.95 1.0000
15 -12 -1 53 178
-5 5.89 -2.20 1.0000
13 -12 -1 271 171

224 -7.38 1.22 1.0000
11 -12 -1 85 164
329 -10.69 -3.14 1.0000
9 -12 -1 2385 270
2603 2.70 5.78 1.0000
7 -12 -1 4779 279
3554 -4.63 6.97 1.0000
5 -12 -1 1968 204
1224 -2.29 14.45 1.0000
3 -12 -1 6965 373
6142 -8.11 4.33 1.0000
1 -12 -1 3553 393
6616 23.48 8.05 1.0000
3 12 1 1931 225
1597 2.06 18.74 1.0000
5 12 1 774 145
1171 24.49 18.10 1.0000
5 12 1 707 185
917 24.49 18.10 1.0000
7 12 1 1035 279
5002 -31.72 13.16 1.0000
7 12 1 1009 360
4944 -31.72 13.16 1.0000
9 12 1 2768 213
733 35.50 1.61 1.0000
9 12 1 2843 161
794 35.50 1.61 1.0000
11 12 1 130 200
138 -4.82 0.85 1.0000
11 12 1 328 127
361 -4.82 0.85 1.0000
13 12 1 289 140
320 6.06 3.04 1.0000
13 12 1 103 187
91 6.06 3.04 1.0000
15 12 1 1068 221
1574 7.67 7.22 1.0000
15 12 1 938 292
1409 7.67 7.22 1.0000
17 12 1 18 266
-73 -1.98 3.13 1.0000
19 12 1 55 288
-139 5.65 3.03 1.0000
21 12 1 29 319

-127	-0.63	4.63	1.0000	
23	12	1	3	341
-187	1.39	0.13	1.0000	
22	-13	-1	382	284
724	-6.41	4.19	1.0000	
22	-13	-1	66	287
220	-6.41	4.19	1.0000	
20	-13	-1	9	250
77	9.00	-4.72	1.0000	
18	-13	-1	58	209
34	-3.70	-6.56	1.0000	
16	-13	-1	142	218
324	0.62	10.61	1.0000	
14	-13	-1	964	251
1464	6.34	-8.11	1.0000	
12	-13	-1	276	186
302	6.18	2.99	1.0000	
10	-13	-1	110	180
479	5.87	8.53	1.0000	
8	-13	-1	1741	302
3563	5.26	-14.93	1.0000	
6	-13	-1	807	185
866	-2.29	-2.58	1.0000	
4	-13	-1	354	160
624	-6.17	0.95	1.0000	
2	-13	-1	2238	294
3495	6.63	-8.06	1.0000	
0	-13	-1	470	164
12	-14.65	9.32	1.0000	
2	13	1	3698	324
4750	-1.83	-10.99	1.0000	
4	13	1	4043	480
8832	17.78	-21.55	1.0000	
6	13	1	150	130
832	-31.35	-11.34	1.0000	
6	13	1	298	185
509	-31.35	-11.34	1.0000	
8	13	1	842	193
1931	23.88	-14.23	1.0000	
8	13	1	754	274
1730	23.88	-14.23	1.0000	
10	13	1	2523	306
2440	-16.68	0.21	1.0000	
10	13	1	2867	225

```
2805  -16.68     0.21  1.0000
  12  13    1      235      214
178   -7.37     1.46  1.0000
  12  13    1      474      146
427   -7.37     1.46  1.0000
  14  13    1       57      187
-142     4.75    -4.47  1.0000
  14  13    1       57      150
-113     4.75    -4.47  1.0000
  16  13    1      159      235
78   -7.06     0.70  1.0000
  16  13    1      493      173
395   -7.06     0.70  1.0000
  18  13    1       67      276
192   -1.01    -6.48  1.0000
  20  13    1       36      292
-320    -3.30    -4.06  1.0000
  22  13    1      471      367
460   -0.09    -0.24  1.0000
  21 -14   -1      164      280
286   -3.92    -0.05  1.0000
  21 -14   -1      402      276
586   -3.92    -0.05  1.0000
  19 -14   -1      122      249
229     4.15     6.03  1.0000
  17 -14   -1      637      237
558     3.69    -4.49  1.0000
  15 -14   -1      576      258
1297  -12.69     5.98  1.0000
  13 -14   -1      352      191
136     4.77    -4.46  1.0000
  11 -14   -1      486      185
269     0.71    -5.37  1.0000
   9 -14   -1       36      209
847  -20.94     4.51  1.0000
   7 -14   -1      238      156
42   18.15     2.13  1.0000
   5 -14   -1      147      162
146     2.32    -1.28  1.0000
   3 -14   -1     1376      195
897    -1.88     5.90  1.0000
   1 -14   -1      324      239
1342    12.89  -11.24  1.0000
   3  14    1     1702      268
```

```
2652     2.74    11.23  1.0000
   5  14    1     1223      255
1510    -6.03    23.17  1.0000
   7  14    1     1368      333
3832    20.82     6.34  1.0000
   7  14    1     1546      262
4127    20.82     6.34  1.0000
   9  14    1     1210      135
345   -11.87     8.46  1.0000
   9  14    1     1099      213
286   -11.87     8.46  1.0000
  11  14    1      149      170
884    14.20     8.64  1.0000
  11  14    1       62      240
613    14.20     8.64  1.0000
  13  14    1      879      234
1353     6.34    -8.09  1.0000
  13  14    1      619      285
1008     6.34    -8.09  1.0000
  15  14    1     1195      297
1127    -4.55     2.46  1.0000
  15  14    1     1494      251
1422    -4.55     2.46  1.0000
  17  14    1      523      208
348    10.51     0.54  1.0000
  17  14    1      153      220
3    10.51     0.54  1.0000
  19  14    1      226      224
208    -4.90     4.43  1.0000
  19  14    1       57      254
4    -4.90     4.43  1.0000
  21  14    1       49      317
118     3.30     1.89  1.0000
  22 -15   -1       95      308
219     6.27    -2.55  1.0000
  22 -15   -1      109      287
244     6.27    -2.55  1.0000
  20 -15   -1      255      260
345    -5.46     1.04  1.0000
  18 -15   -1      893      277
913     0.18     0.24  1.0000
  16 -15   -1       60      218
357     9.03    -5.12  1.0000
  14 -15   -1     1119      249
```

1056	-4.61	2.44	1.0000	
12	-15	-1	662	232
1053	7.70	7.29	1.0000	
10	-15	-1	687	208
629	3.49	-4.94	1.0000	
8	-15	-1	727	181
281	-1.61	9.68	1.0000	
6	-15	-1	1647	250
1591	2.51	0.00	1.0000	
4	-15	-1	185	162
147	-12.13	-11.86	1.0000	
2	-15	-1	1246	195
202	-6.12	18.97	1.0000	
0	-15	-1	1116	238
1154	0.32	-2.31	1.0000	
4	15	1	658	190
198	-5.79	-17.57	1.0000	
6	15	1	1593	201
549	14.07	-6.68	1.0000	
8	15	1	1392	318
2483	-17.66	-5.88	1.0000	
10	15	1	100	216
74	3.39	-6.53	1.0000	
10	15	1	159	144
153	3.39	-6.53	1.0000	
12	15	1	112	170
292	5.89	-2.24	1.0000	
12	15	1	52	241
185	5.89	-2.24	1.0000	
14	15	1	757	264
1591	-12.82	6.01	1.0000	
14	15	1	700	304
1503	-12.82	6.01	1.0000	
16	15	1	61	265
87	0.26	-2.96	1.0000	
16	15	1	307	203
375	0.26	-2.96	1.0000	
18	15	1	11	299
-1	1.52	2.42	1.0000	
20	15	1	567	296
516	0.76	-0.59	1.0000	
22	15	1	299	301
383	-1.20	-2.53	1.0000	
21	-16	-1	536	294

457 0.04 1.96 1.0000
19 -16 -1 71 251
133 1.03 -2.76 1.0000
17 -16 -1 78 231
182 -0.50 4.16 1.0000
15 -16 -1 228 213
292 0.23 -3.17 1.0000
13 -16 -1 98 202
26 -6.87 0.66 1.0000
11 -16 -1 278 203
276 -4.92 -2.90 1.0000
9 -16 -1 389 183
-25 15.29 -7.64 1.0000
7 -16 -1 564 225
967 -15.77 -3.06 1.0000
5 -16 -1 1481 251
1547 8.66 3.62 1.0000
3 -16 -1 1614 195
338 26.57 -5.73 1.0000
1 -16 -1 812 184
141 -16.32 -1.56 1.0000
-1 -16 -1 999 227
665 15.78 -5.97 1.0000
3 16 1 848 187
230 2.51 20.10 1.0000
5 16 1 335 218
431 -10.73 4.55 1.0000
7 16 1 954 294
1638 7.23 7.24 1.0000
9 16 1 517 210
212 3.41 6.25 1.0000
11 16 1 337 227
54 -2.92 -9.12 1.0000
11 16 1 596 183
180 -2.92 -9.12 1.0000
13 16 1 264 274
552 0.57 10.74 1.0000
13 16 1 261 213
546 0.57 10.74 1.0000
15 16 1 74 261
392 9.02 -5.16 1.0000
17 16 1 266 298
239 -3.09 -0.84 1.0000
19 16 1 79 304

```
56     2.16   -0.62  1.0000
  21  16    1        12       336
-78    -0.03   -3.01  1.0000
  20 -17   -1        81       265
193     0.56   -4.35  1.0000
  18 -17   -1       328       272
418     0.10    3.21  1.0000
  16 -17   -1        12       207
-57    -2.96   -0.78  1.0000
  14 -17   -1       402       222
233    10.59    0.51  1.0000
  12 -17   -1       178       200
116    -2.01    3.12  1.0000
  10 -17   -1       607       220
399    -2.20    6.51  1.0000
   8 -17   -1       322       192
145     2.48    5.50  1.0000
   4 -17   -1       904       296
1749    -0.91   10.91  1.0000
   2 -17   -1      1005       309
2028    -9.11  -11.87  1.0000
   0 -17   -1       439       237
760    -2.09    8.56  1.0000
  -2 -17   -1      1294       355
2316     0.12   10.61  1.0000
   4  17    1      1429       259
1457     0.03   -0.31  1.0000
   6  17    1       868       239
735     3.71  -15.12  1.0000
   8  17    1      1689       278
1624    -0.91   -0.63  1.0000
  10  17    1       181       220
51     2.28    5.50  1.0000
  12  17    1       656       266
485    -2.79    2.10  1.0000
  14  17    1       779       299
700     3.65   -4.35  1.0000
  16  17    1       221       305
375    -0.43    3.99  1.0000
  18  17    1       308       289
308    -3.22   -2.24  1.0000
  20  17    1        18       343
91     2.23    4.06  1.0000
  19 -18   -1       151       284
```

```
274      2.74      2.89  1.0000
  17 -18  -1       104       247
96    -3.30    -2.35  1.0000
  15 -18  -1       166       235
257      1.47      2.46  1.0000
  13 -18  -1       196       240
407    -1.10    -6.57  1.0000
  11 -18  -1        58       219
18    -4.15    -3.01  1.0000
   9 -18  -1       248       206
202    -4.68    -3.53  1.0000
   7 -18  -1       280       190
-11    13.30    -5.76  1.0000
   5 -18  -1       405       223
428      1.56    -2.17  1.0000
   3 -18  -1       117       223
334    -6.76      0.28  1.0000
   1 -18  -1       118       264
985    20.83      1.95  1.0000
  -1 -18  -1       131       244
463    -6.98    -5.63  1.0000
   5  18   1      1567       261
1201     11.50      4.82  1.0000
   7  18   1       486       348
2004   -18.75      9.03  1.0000
   9  18   1       109       223
-168      5.63    -7.11  1.0000
  11  18   1        17       226
-69      3.12      1.81  1.0000
  13  18   1       185       270
259    -3.61    -6.75  1.0000
  15  18   1      2449       438
2490      0.07      0.35  1.0000
  17  18   1        15       309
-40    -0.02      3.30  1.0000
  19  18   1       146       334
236      1.85    -4.54  1.0000
  18 -19  -1        66       262
122      1.83    -4.53  1.0000
  16 -19  -1         7       233
-46      2.16    -0.64  1.0000
  14 -19  -1        56       228
-222    -4.75      4.45  1.0000
  12 -19  -1        26       203
```

105	5.65	3.00	1.0000	
10	-19	-1	44	229
216	3.29	6.27	1.0000	
8	-19	-1	9	217
144	-7.40	2.50	1.0000	
6	-19	-1	248	228
385	-1.66	3.82	1.0000	
4	-19	-1	319	228
387	-4.52	-2.59	1.0000	
2	-19	-1	465	241
378	0.77	1.66	1.0000	
0	-19	-1	145	241
263	-3.85	-0.68	1.0000	
-2	-19	-1	443	283
648	0.65	-4.76	1.0000	
6	19	1	490	243
319	-5.71	3.64	1.0000	
8	19	1	207	242
-77	12.08	-3.13	1.0000	
10	19	1	283	252
353	-2.09	-0.37	1.0000	
12	19	1	630	259
408	-3.20	2.92	1.0000	
14	19	1	281	273
466	4.18	6.09	1.0000	
16	19	1	48	308
98	1.02	-2.68	1.0000	
18	19	1	379	349
566	2.61	2.99	1.0000	
17	-20	-1	27	237
-271	2.34	3.89	1.0000	
15	-20	-1	531	268
483	0.72	-0.57	1.0000	
13	-20	-1	37	220
-128	-3.30	-4.09	1.0000	
11	-20	-1	45	229
148	1.51	-4.58	1.0000	
9	-20	-1	302	249
579	-4.58	-4.42	1.0000	
7	-20	-1	384	242
533	-3.04	1.35	1.0000	
5	-20	-1	659	282
1148	6.80	-2.34	1.0000	
3	-20	-1	733	264

```
595     1.14     2.26  1.0000
   1 -20  -1      786      290
847    -2.31     0.05  1.0000
  -1 -20  -1      535      275
463     1.23     0.79  1.0000
  -3 -20  -1      142      273
82     0.79     2.60  1.0000
   5  20   1      194      235
7  -13.13     3.43  1.0000
   7  20   1      261      270
660     6.41    -6.96  1.0000
   9  20   1       60      255
106    -7.70     3.07  1.0000
  11  20   1       56      244
116    -3.14     1.71  1.0000
  13  20   1       37      278
312     8.96    -4.67  1.0000
  15  20   1      419      301
550    -5.52     0.88  1.0000
  17  20   1      121      322
254     0.70    -4.33  1.0000
  16 -21  -1      115      277
206    -0.10    -3.14  1.0000
  14 -21  -1       19      241
-302     3.26     1.89  1.0000
  12 -21  -1      185      258
363    -0.61     4.69  1.0000
  10 -21  -1       13      238
20    -1.41     3.02  1.0000
   8 -21  -1      366      240
204     3.74     2.05  1.0000
   6 -21  -1      373      237
295    -3.08     0.38  1.0000
   4 -21  -1      384      260
413    -1.73    -4.32  1.0000
   2 -21  -1      584      273
619    -1.59    -1.12  1.0000
   0 -21  -1       79      255
129     0.82    -2.10  1.0000
  -2 -21  -1      514      278
637     3.13    -0.95  1.0000
  -4 -21  -1       84      292
109    -1.07    -1.54  1.0000
   6  21   1       20      242
```

218 8.91 0.67 1.0000
8 21 1 36 247
-256 -3.66 3.67 1.0000
10 21 1 36 287
15 2.49 -1.37 1.0000
12 21 1 457 283
313 5.48 -0.19 1.0000
14 21 1 224 307
366 -3.97 0.04 1.0000
16 21 1 643 333
557 -0.10 2.09 1.0000
15 -22 -1 140 269
200 -1.15 -2.71 1.0000
13 -22 -1 0 250
-171 -0.01 -0.31 1.0000
11 -22 -1 159 259
174 -4.06 -1.67 1.0000
9 -22 -1 46 239
99 1.07 -4.15 1.0000
7 -22 -1 15 230
-10 -3.32 -0.68 1.0000
5 -22 -1 973 265
665 -6.55 4.15 1.0000
3 -22 -1 164 269
376 6.95 2.43 1.0000
1 -22 -1 158 262
109 -3.15 -0.58 1.0000
-1 -22 -1 26 261
-100 -1.43 4.19 1.0000
-3 -22 -1 80 286
65 -2.03 -2.39 1.0000
7 22 1 301 265
206 -3.67 -0.79 1.0000
9 22 1 132 280
120 -0.50 -0.01 1.0000
11 22 1 201 299
89 1.75 -3.89 1.0000
13 22 1 168 316
410 -6.53 4.23 1.0000
15 22 1 236 328
440 6.37 -2.56 1.0000
12 -23 -1 238 298
245 1.41 0.08 1.0000
10 -23 -1 16 261

-6 1.24 3.29 1.0000
8 -23 -1 10 251
-51 -1.46 2.33 1.0000
6 -23 -1 14 254
-82 1.13 -3.03 1.0000
4 -23 -1 218 270
282 -1.03 -1.47 1.0000
2 -23 -1 627 299
718 -1.81 -3.44 1.0000
0 -23 -1 35 274
-292 3.42 -3.86 1.0000
-2 -23 -1 3 280
-86 1.26 0.90 1.0000
-4 -23 -1 96 337
145 1.39 -2.66 1.0000
8 23 1 556 288
681 1.61 -2.32 1.0000
10 23 1 134 307
232 1.89 2.73 1.0000
12 23 1 280 343
282 0.12 -0.02 1.0000
11 -24 -1 57 301
151 -1.99 -3.62 1.0000
9 -24 -1 189 292
331 -5.03 1.90 1.0000
7 -24 -1 123 269
128 -0.52 -1.61 1.0000
5 -24 -1 245 269
199 1.70 0.28 1.0000
3 -24 -1 1192 340
1635 -4.70 2.20 1.0000
1 -24 -1 1 283
23 1.89 3.46 1.0000
-1 -24 -1 285 308
227 -1.61 -1.34 1.0000
-3 -24 -1 196 311
280 -2.65 0.65 1.0000
-5 -24 -1 25 364
-30 2.71 3.40 1.0000
9 24 1 203 293
265 0.60 -1.68 1.0000
11 24 1 137 318
193 -1.66 1.10 1.0000
8 -25 -1 196 300

227 0.48 -0.81 1.0000
6 -25 -1 0 298
14 -2.00 1.98 1.0000
4 -25 -1 227 298
309 -1.86 -2.09 1.0000
2 -25 -1 16 290
-14 1.96 -2.83 1.0000
0 -25 -1 59 316
64 -0.18 0.24 1.0000
-2 -25 -1 5 311
-108 0.45 -1.82 1.0000
-4 -25 -1 408 345
348 3.35 -0.77 1.0000
-6 -25 -1 707 414
808 -1.47 0.60 1.0000
5 -26 -1 427 310
402 -0.51 0.39 1.0000
3 -26 -1 111 302
136 0.92 -0.29 1.0000
1 -26 -1 307 327
275 -1.47 0.37 1.0000
-1 -26 -1 5 328
-186 1.82 -0.57 1.0000
-3 -26 -1 95 340
102 -0.31 1.97 1.0000
-5 -26 -1 219 384
382 -4.23 -3.61 1.0000
-4 -26 2 7 358
-281 -0.83 -2.07 1.0000
-2 -26 2 268 356
415 3.61 -1.16 1.0000
0 -26 2 4 313
-311 -0.34 1.73 1.0000
2 -26 2 429 333
551 -1.30 2.27 1.0000
4 -26 2 134 299
128 0.47 -1.33 1.0000
9 25 -2 98 391
147 0.57 -2.16 1.0000
-7 -25 2 193 391
225 2.69 0.78 1.0000
-5 -25 2 272 338
270 -0.62 0.21 1.0000
-3 -25 2 30 296

-223 -2.80 3.79 1.0000
-1 -25 2 136 320
97 -0.39 2.74 1.0000
1 -25 2 15 289
-64 1.44 -3.04 1.0000
3 -25 2 147 304
124 -3.24 3.23 1.0000
5 -25 2 145 299
165 1.59 -1.26 1.0000
7 -25 2 211 304
197 0.72 1.24 1.0000
9 -25 2 21 286
-34 -3.04 2.51 1.0000
10 24 -2 4 373
7 0.49 2.41 1.0000
10 24 -2 62 304
94 0.49 2.41 1.0000
8 24 -2 261 327
306 0.09 2.59 1.0000
6 24 -2 456 284
611 -2.82 1.00 1.0000
-6 -24 2 284 366
408 -2.82 -1.00 1.0000
-4 -24 2 55 313
189 5.08 -2.37 1.0000
-2 -24 2 5 307
-87 0.02 -1.94 1.0000
0 -24 2 517 319
459 0.59 1.10 1.0000
2 -24 2 345 302
436 2.39 -1.45 1.0000
4 -24 2 137 283
235 -0.27 3.48 1.0000
6 -24 2 9 259
-86 -2.59 -0.35 1.0000
8 -24 2 147 297
173 -1.79 -4.06 1.0000
10 -24 2 9 291
-41 -2.22 -1.27 1.0000
13 23 -2 316 402
244 -1.79 -0.59 1.0000
13 23 -2 191 337
136 -1.79 -0.59 1.0000
11 23 -2 244 330

305   1.98   -1.41 1.0000
  11   23 -2   8   355
-259   1.98   -1.41 1.0000
   9   23 -2   488   319
542   0.76   -0.97 1.0000
   9   23 -2   65   283
85   0.76   -0.97 1.0000
   7   23 -2   107   272
266   -2.85   -4.48 1.0000
   5   23 -2   143   248
223   0.91   -2.42 1.0000
  -5 -23   2   9   320
-37   0.91   2.42 1.0000
   3   23 -2   17   193
-90   -0.50   -3.52 1.0000
  -3 -23   2   30   287
8   -0.50   3.52 1.0000
  -1 -23   2   190   282
166   -0.48   0.87 1.0000
   1 -23   2   371   298
496   -0.94   6.24 1.0000
   3 -23   2   34   258
-157   -1.23   -4.91 1.0000
   5 -23   2   86   269
121   -4.44   0.82 1.0000
   7 -23   2   26   257
-109   1.71   4.05 1.0000
   9 -23   2   17   247
-43   2.75   -2.27 1.0000
  11 -23   2   334   298
517   0.18   4.44 1.0000
  13 -23   2   23   267
63   -0.31   2.81 1.0000
  14   22 -2   418   409
383   -2.70   -0.30 1.0000
  14   22 -2   317   337
285   -2.70   -0.30 1.0000
  12   22 -2   287   356
261   0.38   0.87 1.0000
  12   22 -2   118   334
102   0.38   0.87 1.0000
  10   22 -2   98   308
149   1.20   2.60 1.0000
  10   22 -2   339   295

434 1.20 2.60 1.0000
8 22 -2 237 265
213 0.43 0.58 1.0000
8 22 -2 378 273
347 0.43 0.58 1.0000
6 22 -2 678 244
316 4.88 7.21 1.0000
4 22 -2 140 217
269 -2.14 3.39 1.0000
-4 -22 2 30 298
101 -2.14 -3.39 1.0000
2 22 -2 345 196
386 -0.16 -1.09 1.0000
-2 -22 2 143 277
169 -0.16 1.09 1.0000
0 -22 2 424 277
170 2.84 -6.00 1.0000
2 -22 2 46 257
183 -1.02 6.86 1.0000
4 -22 2 1 238
-239 0.43 -0.65 1.0000
6 -22 2 534 281
606 1.69 -1.36 1.0000
8 -22 2 180 257
266 -3.56 2.01 1.0000
10 -22 2 57 241
-144 -5.17 -4.05 1.0000
12 -22 2 110 244
208 -0.71 -3.55 1.0000
14 -22 2 425 288
423 0.82 -0.36 1.0000
15 21 -2 19 306
44 0.85 2.17 1.0000
15 21 -2 609 413
735 0.85 2.17 1.0000
13 21 -2 59 358
157 4.14 -2.82 1.0000
13 21 -2 133 306
273 4.14 -2.82 1.0000
11 21 -2 0 300
-15 0.00 -0.27 1.0000
11 21 -2 0 276
-166 0.00 -0.27 1.0000
9 21 -2 19 256

15 -2.97 -4.89 1.0000

9 21 -2 26 256

61 -2.97 -4.89 1.0000

7 21 -2 214 254

265 0.11 -2.10 1.0000

7 21 -2 285 235

344 0.11 -2.10 1.0000

5 21 -2 50 207

62 -4.14 -6.98 1.0000

-5 -21 2 88 297

-284 -4.14 6.98 1.0000

3 21 -2 487 194

284 1.82 -4.40 1.0000

-3 -21 2 746 282

489 1.82 4.40 1.0000

1 21 -2 304 175

281 0.54 0.25 1.0000

-1 -21 2 378 265

352 0.54 -0.25 1.0000

1 -21 2 7 250

15 -4.08 2.00 1.0000

3 -21 2 14 233

-34 2.93 -1.40 1.0000

5 -21 2 4 227

-76 0.28 -1.72 1.0000

7 -21 2 48 242

11 -7.17 -3.66 1.0000

9 -21 2 351 265

714 4.55 5.24 1.0000

11 -21 2 329 261

398 1.49 0.63 1.0000

13 -21 2 62 263

114 2.45 -0.09 1.0000

15 -21 2 261 262

136 0.67 3.89 1.0000

16 20 -2 357 325

428 0.20 1.67 1.0000

16 20 -2 98 391

137 0.20 1.67 1.0000

14 20 -2 967 296

686 -2.00 -5.66 1.0000

14 20 -2 816 344

558 -2.00 -5.66 1.0000

12 20 -2 59 267

```
157     2.10     4.18  1.0000
  12  20  -2        29       299
-82     2.10     4.18  1.0000
  10  20  -2      1111       292
1163    -1.31     1.25  1.0000
  10  20  -2       389       291
419    -1.31     1.25  1.0000
   8  20  -2        28       249
-131      1.94     4.19  1.0000
   8  20  -2        29       218
2     1.94     4.19  1.0000
   6  20  -2        30       228
32      1.37     7.58  1.0000
   6  20  -2        79       186
-23      1.37     7.58  1.0000
   4  20  -2       496       195
461    -2.49     1.61  1.0000
  -4 -20   2       783       309
741    -2.49    -1.61  1.0000
   2  20  -2       151       186
411     2.92     6.33  1.0000
  -2 -20   2        46       261
218     2.92    -6.33  1.0000
   0  20  -2       305       183
719    -1.33    -8.02  1.0000
   0 -20   2       634       308
1193    -1.33     8.02  1.0000
   2 -20   2       500       255
248     1.21    -5.62  1.0000
   4 -20   2       121       229
-12    -0.78     9.52  1.0000
   6 -20   2       179       248
401    -3.19     5.12  1.0000
   8 -20   2       807       256
535    -0.05    -4.90  1.0000
  10 -20   2         4       233
-96    -1.11     1.46  1.0000
  12 -20   2       454       246
411    -4.22    -2.17  1.0000
  14 -20   2        37       247
-11    -4.57    -2.58  1.0000
  16 -20   2       175       271
382     5.50     0.37  1.0000
  17  19  -2       559       388
```

601 2.41 -1.53 1.0000
17 19 -2 11 310
-101 2.41 -1.53 1.0000
15 19 -2 737 356
803 -2.23 -0.85 1.0000
15 19 -2 266 266
304 -2.23 -0.85 1.0000
13 19 -2 207 261
229 -0.58 3.31 1.0000
13 19 -2 280 307
307 -0.58 3.31 1.0000
11 19 -2 301 252
182 0.86 -5.04 1.0000
11 19 -2 615 273
447 0.86 -5.04 1.0000
9 19 -2 381 223
284 -1.82 -1.48 1.0000
9 19 -2 223 274
150 -1.82 -1.48 1.0000
7 19 -2 1022 291
1493 0.34 -5.84 1.0000
7 19 -2 585 280
951 0.34 -5.84 1.0000
5 19 -2 44 186
237 2.63 -7.20 1.0000
3 19 -2 2500 321
3192 -2.14 5.23 1.0000
-3 -19 2 3119 439
3887 -2.14 -5.23 1.0000
1 19 -2 297 187
610 -1.10 -9.99 1.0000
-1 -19 2 350 275
693 -1.10 9.99 1.0000
-1 19 -2 353 137
39 5.93 10.56 1.0000
1 -19 2 489 244
102 5.93 -10.56 1.0000
3 -19 2 420 243
436 -7.35 -1.01 1.0000
5 -19 2 739 230
401 3.52 -5.20 1.0000
7 -19 2 101 211
-134 6.36 -5.96 1.0000
9 -19 2 140 218

```
234    -7.95     0.98  1.0000
  11 -19    2      462       223
307     4.08     0.10  1.0000
  13 -19    2      108       237
151     3.63     5.13  1.0000
  15 -19    2        8       242
-15    -1.29    -2.10  1.0000
  17 -19    2        1       268
25     0.79     3.68  1.0000
  18  18   -2        4       323
-5    -1.31     1.06  1.0000
  18  18   -2     1223       406
1159    -1.31     1.06  1.0000
  16  18   -2      149       354
249    -4.95    -3.30  1.0000
  16  18   -2       47       291
-252    -4.95    -3.30  1.0000
  14  18   -2      498       265
249     8.40     5.11  1.0000
  14  18   -2      827       297
524     8.40     5.11  1.0000
  12  18   -2     1644       370
2186    -2.91    -4.58  1.0000
  12  18   -2     2054       376
2656    -2.91    -4.58  1.0000
  10  18   -2      532       227
395     3.20    -0.30  1.0000
  10  18   -2      679       260
523     3.20    -0.30  1.0000
   8  18   -2     1057       285
1133     2.41    12.95  1.0000
   8  18   -2     1001       231
1068     2.41    12.95  1.0000
   6  18   -2     2138       293
2420   -12.83    -6.31  1.0000
   6  18   -2     1828       333
2068   -12.83    -6.31  1.0000
   4  18   -2       82       187
542     6.31    10.75  1.0000
   2  18   -2       78       146
-27    -6.23    -4.43  1.0000
  -2 -18    2      592       249
321    -6.23     4.43  1.0000
   0  18   -2        8       138
```

-142 -2.44 0.20 1.0000
0 -18 2 328 223
307 -2.44 -0.20 1.0000
-2 18 -2 25 158
442 6.24 -13.33 1.0000
2 -18 2 20 229
411 6.24 13.33 1.0000
4 -18 2 364 229
295 -2.84 0.67 1.0000
6 -18 2 364 235
653 -1.36 7.81 1.0000
8 -18 2 455 239
888 -1.75 7.13 1.0000
10 -18 2 48 199
147 -5.54 -6.81 1.0000
12 -18 2 7 205
-96 2.06 0.96 1.0000
14 -18 2 247 226
177 -1.18 -2.56 1.0000
16 -18 2 23 223
-67 -2.15 -3.59 1.0000
18 -18 2 245 287
217 -1.40 1.04 1.0000
19 17 -2 84 414
177 0.87 3.60 1.0000
19 17 -2 18 301
-92 0.87 3.60 1.0000
17 17 -2 41 320
17 -0.47 1.96 1.0000
17 17 -2 556 366
453 -0.47 1.96 1.0000
15 17 -2 83 291
292 6.53 -2.49 1.0000
15 17 -2 327 328
679 6.53 -2.49 1.0000
13 17 -2 462 283
269 -3.61 2.54 1.0000
13 17 -2 393 265
217 -3.61 2.54 1.0000
11 17 -2 2502 378
3318 -7.36 0.73 1.0000
11 17 -2 2211 362
2980 -7.36 0.73 1.0000
9 17 -2 94 215

-9 8.12 -2.15 1.0000
9 17 -2 398 199
340 8.12 -2.15 1.0000
7 17 -2 228 233
353 -1.65 -5.16 1.0000
7 17 -2 184 174
294 -1.65 -5.16 1.0000
5 17 -2 615 218
571 5.29 7.03 1.0000
5 17 -2 391 171
333 5.29 7.03 1.0000
3 17 -2 308 142
-54 5.26 -14.28 1.0000
1 17 -2 483 243
1810 4.53 18.51 1.0000
-1 -17 2 565 322
1966 4.53 -18.51 1.0000
-1 17 -2 2436 243
2097 -2.94 -1.29 1.0000
1 -17 2 2189 298
1869 -2.94 1.29 1.0000
-3 17 -2 665 220
1458 7.04 10.31 1.0000
3 -17 2 908 289
1808 7.04 -10.31 1.0000
5 -17 2 2068 363
3797 -4.16 -14.54 1.0000
7 -17 2 34 185
-18 -0.17 5.06 1.0000
9 -17 2 393 264
1330 11.92 -8.15 1.0000
11 -17 2 603 230
803 -6.95 3.29 1.0000
13 -17 2 289 235
489 0.12 5.11 1.0000
15 -17 2 37 223
84 3.29 1.94 1.0000
17 -17 2 193 246
137 -0.36 1.89 1.0000
19 -17 2 22 265
22 2.31 -1.58 1.0000
20 16 -2 69 338
216 5.58 0.45 1.0000
20 16 -2 652 422

1021 5.58 0.45 1.0000
18 16 -2 24 286
-238 -2.20 -3.62 1.0000
18 16 -2 668 380
758 -2.20 -3.62 1.0000
16 16 -2 139 258
107 3.01 -0.74 1.0000
16 16 -2 196 321
159 3.01 -0.74 1.0000
14 16 -2 544 272
576 -6.33 -2.78 1.0000
14 16 -2 732 289
780 -6.33 -2.78 1.0000
12 16 -2 815 279
1391 6.45 -4.25 1.0000
12 16 -2 531 279
1009 6.45 -4.25 1.0000
10 16 -2 1427 297
1647 3.10 6.04 1.0000
10 16 -2 2196 333
2480 3.10 6.04 1.0000
8 16 -2 377 307
1889 -15.16 -16.30 1.0000
8 16 -2 250 272
1570 -15.16 -16.30 1.0000
6 16 -2 88 243
808 11.09 18.19 1.0000
6 16 -2 89 182
811 11.09 18.19 1.0000
4 16 -2 675 251
2280 -20.22 -8.93 1.0000
4 16 -2 719 289
2366 -20.22 -8.93 1.0000
2 16 -2 639 173
859 -2.92 -2.29 1.0000
-2 -16 2 794 251
1037 -2.92 2.29 1.0000
0 16 -2 879 152
543 4.10 -3.67 1.0000
0 -16 2 523 197
273 4.10 3.67 1.0000
-2 16 -2 740 116
70 -11.76 -11.36 1.0000
2 -16 2 356 179

-31 -11.76 11.36 1.0000
4 -16 2 296 215
525 9.51 17.85 1.0000
6 -16 2 395 208
409 1.72 -0.82 1.0000
8 -16 2 800 211
567 -10.93 0.93 1.0000
10 -16 2 63 192
293 8.18 6.46 1.0000
12 -16 2 173 238
873 -8.96 -13.47 1.0000
14 -16 2 87 212
72 2.59 -0.18 1.0000
16 -16 2 13 215
-24 3.04 -0.71 1.0000
18 -16 2 29 257
41 -4.93 -3.24 1.0000
20 -16 2 3 273
11 0.11 1.60 1.0000
21 15 -2 112 320
38 0.62 3.83 1.0000
21 15 -2 1045 436
781 0.62 3.83 1.0000
19 15 -2 7 304
-261 -1.34 -1.96 1.0000
19 15 -2 493 382
394 -1.34 -1.96 1.0000
17 15 -2 665 352
861 3.44 1.89 1.0000
17 15 -2 172 289
273 3.44 1.89 1.0000
15 15 -2 1780 302
1047 -6.54 11.52 1.0000
15 15 -2 899 266
369 -6.54 11.52 1.0000
13 15 -2 175 266
453 1.75 -7.67 1.0000
13 15 -2 172 255
448 1.75 -7.67 1.0000
11 15 -2 206 222
598 5.55 11.16 1.0000
11 15 -2 364 268
887 5.55 11.16 1.0000
9 15 -2 570 188

518	-6.45	3.30	1.0000	
9	15	-2	463	208
409	-6.45	3.30	1.0000	
7	15	-2	2946	247
1746	16.19	6.13	1.0000	
7	15	-2	3321	287
2053	16.19	6.13	1.0000	
5	15	-2	103	149
407	-0.35	-9.13	1.0000	
5	15	-2	40	196
263	-0.35	-9.13	1.0000	
3	15	-2	892	208
1389	10.21	13.30	1.0000	
1	15	-2	2169	240
2749	9.27	-5.38	1.0000	
-1	-15	2	2227	321
2817	9.27	5.38	1.0000	
-1	15	-2	168	150
717	1.48	14.50	1.0000	
1	-15	2	167	220
719	1.48	-14.50	1.0000	
-3	15	-2	178	187
1054	1.64	16.74	1.0000	
3	-15	2	81	114
787	1.64	-16.74	1.0000	
3	-15	2	260	227
1244	1.64	-16.74	1.0000	
5	-15	2	195	168
90	3.11	2.41	1.0000	
7	-15	2	187	177
278	0.16	-15.65	1.0000	
9	-15	2	1773	228
1077	-2.19	7.76	1.0000	
11	-15	2	1	178
221	6.91	10.41	1.0000	
13	-15	2	37	190
-22	0.61	-5.27	1.0000	
15	-15	2	1278	230
656	-6.59	11.46	1.0000	
17	-15	2	40	235
201	6.49	-2.47	1.0000	
19	-15	2	7	248
-6	-2.26	-0.71	1.0000	
21	-15	2	63	282

107	0.92	2.12	1.0000	
22	14	-2	1165	458
1148	0.83	-0.17	1.0000	
22	14	-2	228	308
220	0.83	-0.17	1.0000	
20	14	-2	266	390
346	-4.55	-2.68	1.0000	
20	14	-2	46	272
55	-4.55	-2.68	1.0000	
18	14	-2	12	241
-188	-1.20	-2.69	1.0000	
18	14	-2	989	336
841	-1.20	-2.69	1.0000	
16	14	-2	336	297
313	2.62	-0.16	1.0000	
16	14	-2	256	268
235	2.62	-0.16	1.0000	
14	14	-2	526	261
419	0.65	-11.97	1.0000	
14	14	-2	191	246
-226	0.65	-11.97	1.0000	
12	14	-2	269	247
425	1.61	3.58	1.0000	
12	14	-2	297	234
460	1.61	3.58	1.0000	
10	14	-2	900	219
158	-7.00	-19.91	1.0000	
10	14	-2	684	172
28	-7.00	-19.91	1.0000	
8	14	-2	11	182
121	4.29	6.96	1.0000	
8	14	-2	13	152
44	4.29	6.96	1.0000	
6	14	-2	14	215
1214	-20.54	-17.63	1.0000	
6	14	-2	20	228
1268	-20.54	-17.63	1.0000	
4	14	-2	1054	161
1003	2.53	6.00	1.0000	
4	14	-2	1078	208
1027	2.53	6.00	1.0000	
2	14	-2	65	125
436	-10.09	4.60	1.0000	
0	14	-2	67	131

```
565   -11.02  -21.43  1.0000
   0 -14    2      103       174
396   -11.02   21.43  1.0000
  -2  14   -2      700       180
1233    11.08   -3.31  1.0000
   2 -14    2      685       209
1218    11.08    3.31  1.0000
  -4  14   -2      933       136
692    -8.52   -3.07  1.0000
   4 -14    2      754       183
529    -8.52    3.07  1.0000
   6 -14    2      593       252
1915     2.13   17.79  1.0000
   8 -14    2      171       206
782    11.71   -6.08  1.0000
  10 -14    2      406       268
1966   -14.16  -16.00  1.0000
  12 -14    2      313       201
437     6.51    5.22  1.0000
  14 -14    2      190       178
-90     0.78  -11.91  1.0000
  16 -14    2      599       238
633    -6.29   -2.73  1.0000
  18 -14    2      128       214
-29     8.39    5.10  1.0000
  20 -14    2      746       274
495    -1.94   -5.74  1.0000
  22 -14    2      176       289
149    -2.90   -0.27  1.0000
  23  13   -2      777       449
963    -0.41    2.75  1.0000
  23  13   -2       10       354
-430    -0.41    2.75  1.0000
  21  13   -2      646       402
798     2.48   -0.08  1.0000
  21  13   -2      239       333
334     2.48   -0.08  1.0000
  19  13   -2       55       279
-266     3.78    5.18  1.0000
  19  13   -2      136       342
195     3.78    5.18  1.0000
  17  13   -2       71       232
175     0.17    5.05  1.0000
  17  13   -2      598       300
```

872 0.17 5.05 1.0000
15 13 -2 566 252
466 0.71 -5.31 1.0000
15 13 -2 390 258
302 0.71 -5.31 1.0000
13 13 -2 2645 413
5830 -1.01 22.19 1.0000
13 13 -2 2212 437
5175 -1.01 22.19 1.0000
11 13 -2 2819 318
2595 1.75 -2.04 1.0000
11 13 -2 2905 288
2677 1.75 -2.04 1.0000
9 13 -2 3825 346
5486 7.81 10.64 1.0000
7 13 -2 1823 200
1388 -1.28 6.97 1.0000
7 13 -2 2265 248
1781 -1.28 6.97 1.0000
5 13 -2 1000 209
2020 20.88 2.84 1.0000
5 13 -2 1258 254
2436 20.88 2.84 1.0000
3 13 -2 2680 193
1754 5.63 -6.88 1.0000
3 13 -2 2631 225
1715 5.63 -6.88 1.0000
1 13 -2 1977 193
2021 0.18 2.76 1.0000
-1 -13 2 2085 269
2134 0.18 -2.76 1.0000
-1 13 -2 737 131
771 10.78 6.40 1.0000
1 -13 2 504 172
505 10.78 -6.40 1.0000
-3 13 -2 2869 209
2656 -4.70 4.60 1.0000
3 -13 2 3199 266
2983 -4.70 -4.60 1.0000
3 -13 2 2803 162
2597 -4.70 -4.60 1.0000
-5 13 -2 653 119
638 -5.00 10.08 1.0000
5 -13 2 484 154

466 -5.00 -10.08 1.0000
7 -13 2 706 201
964 4.97 4.02 1.0000
9 -13 2 516 151
88 -6.66 9.65 1.0000
11 -13 2 47 173
200 6.46 -1.33 1.0000
13 -13 2 2190 390
5118 -0.98 22.05 1.0000
15 -13 2 175 212
448 1.57 -7.67 1.0000
17 -13 2 394 225
216 -3.71 2.48 1.0000
19 -13 2 15 217
-181 -0.57 3.31 1.0000
21 -13 2 33 251
-56 4.17 -2.71 1.0000
23 -13 2 161 306
112 -1.73 -0.59 1.0000
22 12 -2 918 402
1179 -0.58 -3.61 1.0000
22 12 -2 174 364
295 -0.58 -3.61 1.0000
20 12 -2 1561 350
1505 -4.35 -2.20 1.0000
20 12 -2 399 338
356 -4.35 -2.20 1.0000
18 12 -2 240 292
318 2.00 1.00 1.0000
18 12 -2 2 271
12 2.00 1.00 1.0000
16 12 -2 449 309
1450 -8.93 -13.43 1.0000
16 12 -2 730 315
1944 -8.93 -13.43 1.0000
14 12 -2 244 219
339 6.71 5.15 1.0000
14 12 -2 239 211
332 6.71 5.15 1.0000
12 12 -2 762 163
-17 -8.07 -22.54 1.0000
12 12 -2 762 206
-29 -8.07 -22.54 1.0000
10 12 -2 1333 182

1110 -0.22 -2.82 1.0000
10 12 -2 915 211
732 -0.22 -2.82 1.0000
8 12 -2 279 157
777 4.00 -10.81 1.0000
8 12 -2 676 243
1407 4.00 -10.81 1.0000
6 12 -2 5048 245
3226 -11.95 -3.14 1.0000
4 12 -2 9718 451
9005 -12.04 1.33 1.0000
4 12 -2 10235 482
9508 -12.04 1.33 1.0000
2 12 -2 115 101
116 -5.26 -6.20 1.0000
2 12 -2 104 137
98 -5.26 -6.20 1.0000
0 12 -2 249 122
596 -4.41 19.72 1.0000
0 -12 2 319 178
816 -4.41 -19.72 1.0000
-2 12 -2 1296 212
2883 -1.09 -18.55 1.0000
2 -12 2 1461 264
3134 -1.09 18.55 1.0000
-4 12 -2 2380 249
4309 11.76 -8.86 1.0000
4 -12 2 2498 300
4466 11.76 8.86 1.0000
4 -12 2 1696 201
3367 11.76 8.86 1.0000
6 -12 2 4054 245
2604 2.65 -18.73 1.0000
8 -12 2 172 151
98 0.74 3.07 1.0000
10 -12 2 521 188
911 7.03 -6.12 1.0000
12 -12 2 1266 165
71 -8.13 -22.56 1.0000
14 -12 2 118 177
225 1.48 3.63 1.0000
16 -12 2 570 243
1060 6.46 -4.17 1.0000
18 -12 2 933 280

1348 -2.75 -4.61 1.0000
20 -12 2 51 241
142 2.06 4.22 1.0000
22 -12 2 180 287
161 0.41 0.82 1.0000
19 11 -2 24 302
-154 4.22 0.06 1.0000
19 11 -2 567 316
392 4.22 0.06 1.0000
17 11 -2 558 310
747 -6.92 3.28 1.0000
17 11 -2 1278 320
1589 -6.92 3.28 1.0000
15 11 -2 206 211
-44 6.96 10.30 1.0000
15 11 -2 25 256
369 6.96 10.30 1.0000
13 11 -2 57 175
-102 6.44 -1.28 1.0000
13 11 -2 5 188
35 6.44 -1.28 1.0000
11 11 -2 2871 329
3935 -4.40 21.28 1.0000
11 11 -2 2617 292
3610 -4.40 21.28 1.0000
9 11 -2 759 157
903 8.71 4.22 1.0000
9 11 -2 954 195
1129 8.71 4.22 1.0000
7 11 -2 1593 197
1774 -0.34 -1.89 1.0000
5 11 -2 134 112
198 1.20 -2.21 1.0000
5 11 -2 201 137
279 1.20 -2.21 1.0000
3 11 -2 2296 154
852 15.46 -13.27 1.0000
3 11 -2 2569 178
1034 15.46 -13.27 1.0000
1 11 -2 2715 204
2748 8.41 -14.40 1.0000
-1 11 -2 2403 187
2506 -4.35 -3.75 1.0000
1 -11 2 2215 236

2316	-4.35	3.75	1.0000	
-3 11	-2	673	157	
1435	-9.43	-4.53	1.0000	
3 -11	2	717	198	
1499	-9.43	4.53	1.0000	
3 -11	2	767	114	
1572	-9.43	4.53	1.0000	
-5 11	-2	4140	313	
6102	-6.08	10.28	1.0000	
5 -11	2	3950	352	
5871	-6.08	-10.28	1.0000	
7 -11	2	1334	306	
4452	-17.78	19.92	1.0000	
9 -11	2	3084	233	
2119	11.10	-3.47	1.0000	
11 -11	2	1955	270	
2769	-4.63	21.40	1.0000	
13 -11	2	2785	285	
2541	1.79	-2.22	1.0000	
15 -11	2	153	206	
479	5.64	11.00	1.0000	
17 -11	2	1806	341	
2514	-7.44	0.74	1.0000	
19 -11	2	303	231	
185	0.80	-5.01	1.0000	
21 -11	2	0	247	
-57	0.10	-0.29	1.0000	
23 -11	2	88	257	
122	1.89	-1.34	1.0000	
23 -11	2	67	283	
97	1.89	-1.34	1.0000	
22 10	-2	1058	389	
1516	-5.18	-4.05	1.0000	
22 10	-2	58	321	
-109	-5.18	-4.05	1.0000	
20 10	-2	39	294	
49	-0.97	1.51	1.0000	
20 10	-2	1177	326	
1242	-0.97	1.51	1.0000	
18 10	-2	389	252	
735	-5.41	-6.89	1.0000	
18 10	-2	86	280	
241	-5.41	-6.89	1.0000	
16 10	-2	98	222	

380 8.19 6.41 1.0000
16 10 -2 275 284
715 8.19 6.41 1.0000
14 10 -2 160 268
1335 -13.92 -16.02 1.0000
14 10 -2 253 286
1592 -13.92 -16.02 1.0000
12 10 -2 616 233
1033 7.12 -6.00 1.0000
12 10 -2 644 184
1071 7.12 -6.00 1.0000
10 10 -2 1303 179
1001 5.28 -6.55 1.0000
10 10 -2 1289 190
988 5.28 -6.55 1.0000
8 10 -2 867 264
4062 -21.74 -20.30 1.0000
6 10 -2 4312 234
3560 27.44 12.73 1.0000
4 10 -2 1040 121
629 -19.32 11.05 1.0000
4 10 -2 1041 147
630 -19.32 11.05 1.0000
2 10 -2 8715 297
5654 -12.12 10.92 1.0000
2 10 -2 8426 316
5422 -12.12 10.92 1.0000
0 10 -2 457 94
175 7.63 7.29 1.0000
0 -10 2 527 129
222 7.63 -7.29 1.0000
-2 10 -2 5888 422
9123 -14.03 19.52 1.0000
2 -10 2 5822 381
9002 -14.03 -19.52 1.0000
2 -10 2 5823 455
9003 -14.03 -19.52 1.0000
-4 10 -2 5459 421
9222 19.25 -0.19 1.0000
4 -10 2 5675 456
9499 19.25 0.19 1.0000
4 -10 2 4892 387
8477 19.25 0.19 1.0000
-6 10 -2 1442 128

813 15.39 -6.70 1.0000
6 -10 2 1388 150
768 15.39 6.70 1.0000
8 -10 2 2 164
747 -2.26 -24.05 1.0000
10 -10 2 1623 192
1278 5.53 -6.74 1.0000
12 -10 2 1096 186
887 -0.25 -2.93 1.0000
14 -10 2 831 175
114 -7.07 -19.88 1.0000
16 -10 2 1081 254
1259 3.03 5.87 1.0000
18 -10 2 279 208
182 3.30 -0.17 1.0000
20 -10 2 577 262
611 -1.43 1.24 1.0000
22 -10 2 6 209
17 1.34 2.63 1.0000
22 -10 2 12 243
-88 1.34 2.63 1.0000
24 -10 2 358 308
432 0.51 2.31 1.0000
25 9 -2 298 384
389 -2.97 2.45 1.0000
21 9 -2 1605 341
2314 4.68 5.17 1.0000
19 9 -2 53 283
34 -8.12 0.96 1.0000
19 9 -2 916 260
1251 -8.12 0.96 1.0000
17 9 -2 330 305
1221 11.97 -8.28 1.0000
17 9 -2 610 303
1720 11.97 -8.28 1.0000
15 9 -2 1997 236
1248 -2.21 7.82 1.0000
15 9 -2 1573 277
918 -2.21 7.82 1.0000
13 9 -2 549 111
102 -6.70 9.60 1.0000
13 9 -2 182 147
-176 -6.70 9.60 1.0000
13 9 -2 285 203

12	-6.70	9.60	1.0000	
11	9	-2	3122	228
2139	11.28	-3.55	1.0000	
11	9	-2	3388	269
2364	11.28	-3.55	1.0000	
9	9	-2	35	147
527	-14.88	19.21	1.0000	
9	9	-2	12	139
742	-14.88	19.21	1.0000	
7	9	-2	4202	298
4907	7.11	-8.93	1.0000	
7	9	-2	3225	254
3827	7.11	-8.93	1.0000	
5	9	-2	1709	179
1943	0.67	-24.88	1.0000	
3	9	-2	5505	521
10766	-8.46	-24.30	1.0000	
3	9	-2	7037	546
12871	-8.46	-24.30	1.0000	
1	9	-2	7291	537
12140	13.52	-17.41	1.0000	
-1	9	-2	9526	190
2821	-7.01	-39.82	1.0000	
1	-9	2	9699	225
2925	-7.01	39.82	1.0000	
-3	9	-2	5297	223
3841	-4.07	12.89	1.0000	
3	-9	2	5712	257
4194	-4.07	-12.89	1.0000	
3	-9	2	5241	187
3786	-4.07	-12.89	1.0000	
-5	9	-2	3998	111
549	-20.38	-27.98	1.0000	
5	-9	2	4418	94
710	-20.38	27.98	1.0000	
5	-9	2	4498	140
742	-20.38	27.98	1.0000	
7	-9	2	10952	468
9408	-4.74	-24.73	1.0000	
9	-9	2	13	156
735	-15.08	19.28	1.0000	
11	-9	2	620	166
747	8.38	4.33	1.0000	
13	-9	2	3418	354

4956 7.62 10.50 1.0000
15 -9 2 419 182
366 -6.32 3.29 1.0000
17 -9 2 263 199
195 8.15 -2.19 1.0000
19 -9 2 374 233
279 -1.71 -1.54 1.0000
21 -9 2 58 233
129 -3.06 -4.90 1.0000
21 -9 2 26 197
60 -3.06 -4.90 1.0000
23 -9 2 326 281
370 0.78 -0.95 1.0000
25 -9 2 6 316
-173 0.57 -2.01 1.0000
24 8 -2 26 307
-267 -1.78 -4.06 1.0000
20 8 -2 724 272
469 -0.15 -4.91 1.0000
18 8 -2 756 265
1300 -1.61 7.25 1.0000
18 8 -2 250 274
594 -1.61 7.25 1.0000
16 8 -2 624 259
405 -10.72 0.88 1.0000
16 8 -2 239 212
60 -10.72 0.88 1.0000
14 8 -2 588 261
1540 11.80 -6.02 1.0000
14 8 -2 242 195
930 11.80 -6.02 1.0000
12 8 -2 52 141
14 1.04 3.02 1.0000
12 8 -2 167 173
90 1.04 3.02 1.0000
12 8 -2 172 96
94 1.04 3.02 1.0000
10 8 -2 5 192
865 -2.03 -23.96 1.0000
10 8 -2 11 151
943 -2.03 -23.96 1.0000
8 8 -2 1777 236
3567 32.43 12.08 1.0000
8 8 -2 1922 266

```
3838   32.43   12.08  1.0000
   6   8  -2    4241      484
9434  -37.52    7.96  1.0000
   6   8  -2    6111      517
12327  -37.52    7.96  1.0000
   4   8  -2    7353      241
4043   19.15   -1.65  1.0000
   4   8  -2    6762      221
3608   19.15   -1.65  1.0000
   2   8  -2    8026      116
750     4.16   56.35  1.0000
   2   8  -2    7123      114
472     4.16   56.35  1.0000
  -2  -8   2    7476      126
588     4.16  -56.35  1.0000
   0   8  -2    3051      370
8432  -31.61    5.68  1.0000
   0  -8   2    2765      397
7939  -31.61   -5.68  1.0000
   0  -8   2    2372      379
7259  -31.61   -5.68  1.0000
  -2   8  -2    4600      104
1811   38.92   12.36  1.0000
  -2   8  -2    4750      146
1941   38.92   12.36  1.0000
   2  -8   2    4731      177
1926   38.92  -12.36  1.0000
   2  -8   2    4530      107
1752   38.92  -12.36  1.0000
  -4   8  -2   12557      342
7376  -17.01   27.14  1.0000
  -4   8  -2   12263      316
7143  -17.01   27.14  1.0000
   4  -8   2   12350      318
7202  -17.01  -27.14  1.0000
   4  -8   2   12897      370
7637  -17.01  -27.14  1.0000
  -6   8  -2     577      117
978     2.65   -6.26  1.0000
  -6   8  -2     668       99
1096    2.65   -6.26  1.0000
   6  -8   2     455      148
818     2.65    6.26  1.0000
   8  -8   2    1865      261
```

```
3796    32.90    12.02  1.0000
  10   -8    2      903       285
4161   -21.90   -20.35  1.0000
  12   -8    2      331       184
869     4.08  -10.79  1.0000
  14   -8    2        8       148
65     4.39     6.80  1.0000
  16   -8    2      152       242
1275   -15.08   -16.36  1.0000
  18   -8    2     1016       257
1082     2.38    12.85  1.0000
  20   -8    2      211       220
191     1.94     4.37  1.0000
  22   -8    2      395       271
360     0.64     0.57  1.0000
  24   -8    2      120       300
149     0.03     2.48  1.0000
  25    7   -2       10       343
6     0.72     1.17  1.0000
  21    7   -2       35       272
94   -7.27    -3.59  1.0000
  21    7   -2      479       279
826    -7.27    -3.59  1.0000
  19    7   -2      106       242
-75     6.57    -6.04  1.0000
  19    7   -2      913       232
494     6.57    -6.04  1.0000
  17    7   -2       33       196
-160    -0.23     4.97  1.0000
  17    7   -2       82       238
35   -0.23     4.97  1.0000
  15    7   -2      172       201
158     0.05   -15.79  1.0000
  15    7   -2      262       174
491     0.05   -15.79  1.0000
  13    7   -2      340       153
509     5.13     3.94  1.0000
  13    7   -2      435       191
630     5.13     3.94  1.0000
  11    7   -2     1490       278
4734   -17.78    19.91  1.0000
  11    7   -2     1232       220
4260   -17.78    19.91  1.0000
  11    7   -2     1247       321
```

4289 -17.78 19.91 1.0000
9 7 -2 12072 519
10572 -4.39 -24.86 1.0000
9 7 -2 12079 490
10579 -4.39 -24.86 1.0000
7 7 -2 1257 118
236 -17.56 3.43 1.0000
7 7 -2 1060 100
154 -17.56 3.43 1.0000
5 7 -2 4442 188
2272 2.45 -24.89 1.0000
5 7 -2 4281 165
2148 2.45 -24.89 1.0000
-5 -7 2 4232 176
2107 2.45 24.89 1.0000
-5 -7 2 4453 196
2277 2.45 24.89 1.0000
3 7 -2 2502 128
1129 17.13 -28.92 1.0000
3 7 -2 2384 146
1004 17.13 -28.92 1.0000
-3 -7 2 2806 147
1387 17.13 28.92 1.0000
-3 -7 2 2557 143
1129 17.13 28.92 1.0000
1 7 -2 7266 821
19436 -14.69 -64.22 1.0000
1 7 -2 7821 815
20455 -14.69 -64.22 1.0000
-1 -7 2 7432 814
19620 -14.69 64.22 1.0000
-1 -7 2 6992 818
18801 -14.69 64.22 1.0000
-1 7 -2 5800 391
9871 7.84 19.55 1.0000
-1 7 -2 5741 424
9793 7.84 19.55 1.0000
1 -7 2 5560 423
9555 7.84 -19.55 1.0000
1 -7 2 5235 434
9126 7.84 -19.55 1.0000
1 -7 2 5234 442
9124 7.84 -19.55 1.0000
1 -7 2 5677 392

9709 7.84 -19.55 1.0000
-3 7 -2 13519 1422
34672 -13.91 -59.22 1.0000
-3 7 -2 14224 1443
35797 -13.91 -59.22 1.0000
-3 7 -2 14095 1468
35592 -13.91 -59.22 1.0000
3 -7 2 14583 1423
36361 -13.91 59.22 1.0000
3 -7 2 13801 1460
35119 -13.91 59.22 1.0000
3 -7 2 15108 1445
37188 -13.91 59.22 1.0000
-5 7 -2 4090 298
6297 -2.18 37.47 1.0000
-5 7 -2 4121 283
6342 -2.18 37.47 1.0000
5 -7 2 4390 332
6776 -2.18 -37.47 1.0000
5 -7 2 3909 284
6065 -2.18 -37.47 1.0000
5 -7 2 4198 302
6494 -2.18 -37.47 1.0000
-7 7 -2 1496 78
344 -17.42 -3.84 1.0000
7 -7 2 1698 89
446 -17.42 3.84 1.0000
7 -7 2 1410 106
303 -17.42 3.84 1.0000
-9 7 -2 2882 189
3431 6.43 8.57 1.0000
9 -7 2 2625 199
3152 6.43 -8.57 1.0000
9 -7 2 3064 249
3642 6.43 -8.57 1.0000
11 -7 2 1535 214
1729 -0.03 -2.10 1.0000
13 -7 2 1702 209
1276 -1.32 7.02 1.0000
15 -7 2 3417 278
2122 16.16 6.23 1.0000
17 -7 2 151 208
255 -1.73 -5.33 1.0000
19 -7 2 473 242

809	0.52		-5.84	1.0000	
21	-7	2	96		234
133	-0.01		-2.15	1.0000	
23	-7	2	199		290
400	-2.88		-4.36	1.0000	
25	-7	2	11		293
-82	2.75		-0.93	1.0000	
24	6	-2	146		318
136	-2.55		-0.34	1.0000	
20	6	-2	26		263
128	-2.91		5.06	1.0000	
20	6	-2	293		208
553	-2.91		5.06	1.0000	
18	6	-2	87		188
224	-1.49		7.85	1.0000	
18	6	-2	187		261
398	-1.49		7.85	1.0000	
16	6	-2	141		173
144	1.96		-0.76	1.0000	
16	6	-2	179		215
183	1.96		-0.76	1.0000	
14	6	-2	274		233
1263	2.25		17.70	1.0000	
14	6	-2	422		280
1577	2.25		17.70	1.0000	
12	6	-2	4378		275
2866	2.81		-18.54	1.0000	
12	6	-2	4542		245
3005	2.81		-18.54	1.0000	
10	6	-2	1796		106
1112	15.19		6.84	1.0000	
10	6	-2	1685		149
1015	15.19		6.84	1.0000	
10	6	-2	2090		200
1371	15.19		6.84	1.0000	
8	6	-2	573		174
999	3.12		6.28	1.0000	
8	6	-2	338		125
677	3.12		6.28	1.0000	
8	6	-2	429		77
805	3.12		6.28	1.0000	
6	6	-2	2520		102
475	23.08		-14.66	1.0000	
6	6	-2	2566		122

```
497    23.08  -14.66  1.0000
  -6   -6    2     2527      123
515    23.08   14.66  1.0000
  -6   -6    2     2596      118
550    23.08   14.66  1.0000
   4    6   -2    13080     1176
30464   -18.13   60.59  1.0000
   4    6   -2    11188     1177
27401   -18.13   60.59  1.0000
  -4   -6    2    11383     1179
27701   -18.13  -60.59  1.0000
  -4   -6    2    12564     1179
29619   -18.13  -60.59  1.0000
   2    6   -2     2158      263
5219   -16.16  -15.54  1.0000
   2    6   -2     2203      268
5289   -16.16  -15.54  1.0000
  -2   -6    2     2357      271
5529   -16.16   15.54  1.0000
  -2   -6    2     2152      271
5212   -16.16   15.54  1.0000
   0    6   -2    18664     1705
42322    19.44   56.69  1.0000
   0   -6    2    19257     1722
43212    19.44  -56.69  1.0000
   0   -6    2    18912     1706
42694    19.44  -56.69  1.0000
   0   -6    2    18451     1709
42000    19.44  -56.69  1.0000
  -2    6   -2    11091      847
19725   -26.51   20.30  1.0000
  -2    6   -2    11539      807
20326   -26.51   20.30  1.0000
  -2    6   -2    11700      828
20542   -26.51   20.30  1.0000
   2   -6    2    11631      808
20475   -26.51  -20.30  1.0000
   2   -6    2    10788      840
19343   -26.51  -20.30  1.0000
   2   -6    2    11587      828
20417   -26.51  -20.30  1.0000
  -4    6   -2    14565     1034
25223    15.05  -30.12  1.0000
  -4    6   -2    14787     1022
```

25515 15.05 -30.12 1.0000
-4 6 -2 14950 1058
25729 15.05 -30.12 1.0000
4 -6 2 14225 1022
24762 15.05 30.12 1.0000
4 -6 2 14920 1055
25677 15.05 30.12 1.0000
4 -6 2 15182 1035
26021 15.05 30.12 1.0000
-6 6 -2 2748 89
613 23.70 14.83 1.0000
-6 6 -2 2806 82
644 23.70 14.83 1.0000
6 -6 2 2777 87
580 23.70 -14.83 1.0000
6 -6 2 3138 127
767 23.70 -14.83 1.0000
6 -6 2 3132 99
764 23.70 -14.83 1.0000
-8 6 -2 3842 396
8879 -37.80 -8.30 1.0000
8 -6 2 3990 439
9061 -37.80 8.30 1.0000
8 -6 2 4088 403
9221 -37.80 8.30 1.0000
-10 6 -2 3934 179
3204 27.47 -12.40 1.0000
10 -6 2 3498 239
2721 27.47 12.40 1.0000
10 -6 2 3984 187
3247 27.47 12.40 1.0000
12 -6 2 3898 232
2327 -11.97 -2.88 1.0000
12 -6 2 3994 178
2401 -11.97 -2.88 1.0000
14 -6 2 3 149
909 -20.75 -17.83 1.0000
14 -6 2 4 204
1115 -20.75 -17.83 1.0000
16 -6 2 236 250
1338 11.30 18.16 1.0000
18 -6 2 2040 323
2307 -12.87 -6.30 1.0000
20 -6 2 59 218

163	1.73	7.50	1.0000	
22	-6	2	100	250
-49	4.76	7.27	1.0000	
24	-6	2	178	295
278	-2.69	1.17	1.0000	
25	5	-2	201	336
226	1.64	-1.28	1.0000	
23	5	-2	249	303
324	-4.37	0.77	1.0000	
21	5	-2	505	210
559	0.20	-1.72	1.0000	
21	5	-2	4	267
-10	0.20	-1.72	1.0000	
19	5	-2	428	263
187	3.34	-5.19	1.0000	
19	5	-2	447	198
200	3.34	-5.19	1.0000	
17	5	-2	2173	404
3922	-4.15	-14.40	1.0000	
17	5	-2	1931	336
3592	-4.15	-14.40	1.0000	
15	5	-2	351	206
203	3.17	2.37	1.0000	
15	5	-2	21	170
-68	3.17	2.37	1.0000	
13	5	-2	475	153
468	-5.29	-10.22	1.0000	
13	5	-2	518	184
520	-5.29	-10.22	1.0000	
11	5	-2	4671	354
6746	-5.70	-10.52	1.0000	
11	5	-2	4302	298
6301	-5.70	-10.52	1.0000	
11	5	-2	4556	377
6608	-5.70	-10.52	1.0000	
9	5	-2	4104	139
579	-20.33	28.18	1.0000	
9	5	-2	4000	127
540	-20.33	28.18	1.0000	
9	5	-2	4399	83
693	-20.33	28.18	1.0000	
-9	-5	2	3592	118
398	-20.33	-28.18	1.0000	
7	5	-2	4085	315

6432 -1.52 -37.58 1.0000
7 5 -2 3889 325
6137 -1.52 -37.58 1.0000
-7 -5 2 3829 324
6010 -1.52 37.58 1.0000
-7 -5 2 3678 309
5780 -1.52 37.58 1.0000
5 5 -2 167 91
95 -13.69 5.87 1.0000
5 5 -2 169 87
86 -13.69 5.87 1.0000
-5 -5 2 186 101
301 -13.69 -5.87 1.0000
-5 -5 2 157 91
162 -13.69 -5.87 1.0000
3 5 -2 20663 1878
44849 -0.19 -60.30 1.0000
3 5 -2 22546 1879
47613 -0.19 -60.30 1.0000
-3 -5 2 22831 1881
48029 -0.19 60.30 1.0000
-3 -5 2 22380 1881
47372 -0.19 60.30 1.0000
1 5 -2 3553 459
10346 38.49 9.43 1.0000
1 5 -2 3868 460
10893 38.49 9.43 1.0000
-1 -5 2 3594 457
10434 38.49 -9.43 1.0000
-1 -5 2 3751 466
10708 38.49 -9.43 1.0000
-1 5 -2 42511 2335
57450 -4.02 -80.44 1.0000
-1 5 -2 43642 2324
58870 -4.02 -80.44 1.0000
-1 5 -2 42551 2304
57500 -4.02 -80.44 1.0000
1 -5 2 44454 2325
59957 -4.02 80.44 1.0000
1 -5 2 42998 2332
58131 -4.02 80.44 1.0000
1 -5 2 43804 2305
59143 -4.02 80.44 1.0000
-3 5 -2 21535 277

6103 13.98 57.91 1.0000
-3 5 -2 21172 292
5910 13.98 57.91 1.0000
-3 5 -2 21438 261
6051 13.98 57.91 1.0000
3 -5 2 21439 277
6053 13.98 -57.91 1.0000
3 -5 2 21845 262
6270 13.98 -57.91 1.0000
3 -5 2 20846 288
5740 13.98 -57.91 1.0000
-5 5 -2 155 85
163 -13.25 -6.28 1.0000
5 -5 2 164 74
88 -13.25 6.28 1.0000
-7 5 -2 4654 148
2524 1.48 25.09 1.0000
7 -5 2 4896 189
2722 1.48 -25.09 1.0000
7 -5 2 4957 153
2771 1.48 -25.09 1.0000
-9 5 -2 2029 141
2279 1.15 24.52 1.0000
9 -5 2 1838 141
2057 1.15 -24.52 1.0000
9 -5 2 1603 187
1740 1.15 -24.52 1.0000
-11 5 -2 234 93
298 0.89 1.71 1.0000
11 -5 2 217 83
276 0.89 -1.71 1.0000
11 -5 2 51 126
81 0.89 -1.71 1.0000
-13 5 -2 782 150
1703 21.46 -2.92 1.0000
13 -5 2 860 224
1826 21.46 2.92 1.0000
15 -5 2 229 185
645 -0.48 -9.21 1.0000
17 -5 2 579 206
543 5.61 7.21 1.0000
19 -5 2 78 230
307 2.45 -7.23 1.0000
21 -5 2 173 243

306	-4.16	-6.97	1.0000	
23	-5	2	10	253
-136	1.06	-2.58	1.0000	
25	-5	2	191	302
190	-0.60	-0.23	1.0000	
26	4	-2	3	375
-15	0.44	-1.45	1.0000	
24	4	-2	84	324
161	-0.48	3.44	1.0000	
22	4	-2	240	228
236	0.50	-0.63	1.0000	
22	4	-2	244	283
240	0.50	-0.63	1.0000	
20	4	-2	186	271
209	-0.82	9.60	1.0000	
20	4	-2	197	213
226	-0.82	9.60	1.0000	
18	4	-2	602	262
527	-2.50	0.48	1.0000	
18	4	-2	353	208
295	-2.50	0.48	1.0000	
16	4	-2	405	246
824	9.23	17.85	1.0000	
16	4	-2	350	197
684	9.23	17.85	1.0000	
14	4	-2	830	199
591	-8.02	3.17	1.0000	
14	4	-2	700	174
476	-8.02	3.17	1.0000	
12	4	-2	2587	300
4575	11.64	8.95	1.0000	
12	4	-2	2904	332
4994	11.64	8.95	1.0000	
10	4	-2	5625	473
9492	19.51	0.09	1.0000	
10	4	-2	5559	407
9406	19.51	0.09	1.0000	
10	4	-2	5754	453
9659	19.51	0.09	1.0000	
-10	-4	2	5554	441
9402	19.51	-0.09	1.0000	
8	4	-2	12349	361
7139	-17.33	-26.92	1.0000	
8	4	-2	12606	354

7342	-17.33	-26.92	1.0000	
-8	-4	2	11921	339
6812	-17.33	26.92	1.0000	
6	4	-2	13155	980
23285	15.00	29.95	1.0000	
6	4	-2	13592	988
23865	15.00	29.95	1.0000	
-6	-4	2	13252	977
23425	15.00	-29.95	1.0000	
-6	-4	2	14168	988
24640	15.00	-29.95	1.0000	
4	4	-2	39912	1720
40495	13.03	-16.24	1.0000	
4	4	-2	42821	1725
43445	13.03	-16.24	1.0000	
-4	-4	2	44735	1725
45435	13.03	16.24	1.0000	
-4	-4	2	41886	1723
42545	13.03	16.24	1.0000	
2	4	-2	71730	3566
86446	-34.67	55.84	1.0000	
2	4	-2	77896	3575
93421	-34.67	55.84	1.0000	
-2	-4	2	74073	3567
88999	-34.67	-55.84	1.0000	
-2	-4	2	77407	3575
92766	-34.67	-55.84	1.0000	
0	4	-2	68617	2964
75151	14.57	-1.22	1.0000	
0	4	-2	67191	2945
73657	14.57	-1.22	1.0000	
0	-4	2	65959	2968
72395	14.57	1.22	1.0000	
0	-4	2	68389	2962
74942	14.57	1.22	1.0000	
0	-4	2	71827	2967
78543	14.57	1.22	1.0000	
-2	4	-2	79536	3803
94803	-36.46	-3.29	1.0000	
-2	4	-2	81216	3784
96648	-36.46	-3.29	1.0000	
-2	4	-2	80550	3794
95917	-36.46	-3.29	1.0000	
2	-4	2	75537	3799

90459 -36.46 3.29 1.0000
2 -4 2 84219 3784
100000 -36.46 3.29 1.0000
2 -4 2 83781 3796
99520 -36.46 3.29 1.0000
-4 4 -2 42190 1737
42686 12.91 16.72 1.0000
-4 4 -2 44692 1759
45220 12.91 16.72 1.0000
-4 4 -2 41981 1740
42475 12.91 16.72 1.0000
4 -4 2 44565 1742
45042 12.91 -16.72 1.0000
4 -4 2 43257 1737
43718 12.91 -16.72 1.0000
4 -4 2 43211 1757
43671 12.91 -16.72 1.0000
-6 4 -2 11529 1111
28315 -18.42 -61.62 1.0000
-6 4 -2 10176 1105
26052 -18.42 -61.62 1.0000
-6 4 -2 11240 1135
27837 -18.42 -61.62 1.0000
6 -4 2 10815 1109
27148 -18.42 61.62 1.0000
6 -4 2 11389 1136
28103 -18.42 61.62 1.0000
6 -4 2 11452 1107
28208 -18.42 61.62 1.0000
-8 4 -2 6330 174
3238 19.53 2.10 1.0000
8 -4 2 6160 209
3116 19.53 -2.10 1.0000
8 -4 2 6426 169
3306 19.53 -2.10 1.0000
-10 4 -2 1178 99
747 -19.36 -12.08 1.0000
10 -4 2 1010 133
552 -19.36 12.08 1.0000
-12 4 -2 8876 381
8105 -12.70 -1.17 1.0000
12 -4 2 9262 445
8487 -12.70 1.17 1.0000
-14 4 -2 1072 140

1005 2.73 -5.80 1.0000
14 -4 2 1193 204
1122 2.73 5.80 1.0000
16 -4 2 892 299
2689 -20.20 -8.89 1.0000
18 -4 2 2 207
185 6.28 10.63 1.0000
20 -4 2 853 247
817 -2.40 1.67 1.0000
22 -4 2 81 254
183 -2.03 3.44 1.0000
24 -4 2 6 267
73 4.95 2.47 1.0000
26 -4 2 7 314
-155 -0.71 2.10 1.0000
25 3 -2 28 337
-60 -3.21 3.24 1.0000
23 3 -2 34 299
-188 -1.20 -4.90 1.0000
21 3 -2 15 274
-107 3.05 -1.40 1.0000
21 3 -2 15 226
-182 3.05 -1.40 1.0000
19 3 -2 559 258
609 -7.45 -0.69 1.0000
19 3 -2 645 229
704 -7.45 -0.69 1.0000
17 3 -2 973 329
1914 7.07 -10.52 1.0000
17 3 -2 832 293
1710 7.07 -10.52 1.0000
15 3 -2 111 210
873 1.46 -16.67 1.0000
15 3 -2 242 242
1200 1.46 -16.67 1.0000
13 3 -2 2893 242
2673 -4.51 -4.60 1.0000
13 3 -2 2695 259
2481 -4.51 -4.60 1.0000
11 3 -2 586 187
1317 -9.49 4.72 1.0000
11 3 -2 347 170
941 -9.49 4.72 1.0000
-11 -3 2 372 151

982 -9.49 -4.72 1.0000
9 3 -2 5568 247
4040 -4.00 -13.08 1.0000
9 3 -2 5608 266
4074 -4.00 -13.08 1.0000
-9 -3 2 5842 233
4284 -4.00 13.08 1.0000
7 3 -2 14553 1460
36371 -13.95 59.34 1.0000
7 3 -2 12625 1444
33281 -13.95 59.34 1.0000
-7 -3 2 14422 1446
36168 -13.95 -59.34 1.0000
-7 -3 2 15258 1461
37485 -13.95 -59.34 1.0000
5 3 -2 21752 297
6216 14.39 -57.79 1.0000
5 3 -2 21819 304
6252 14.39 -57.79 1.0000
-5 -3 2 20812 290
5717 14.39 57.79 1.0000
-5 -3 2 21978 294
6336 14.39 57.79 1.0000
3 3 -2 18416 826
20113 18.05 23.95 1.0000
-3 -3 2 19684 833
21465 18.05 -23.95 1.0000
-3 -3 2 17587 825
19207 18.05 -23.95 1.0000
1 3 -2 59554 1440
36568 4.54 -56.66 1.0000
1 3 -2 57194 1430
34693 4.54 -56.66 1.0000
-1 -3 2 59820 1440
36828 4.54 56.66 1.0000
-1 -3 2 58460 1431
35745 4.54 56.66 1.0000
-1 3 -2 29332 2181
53918 42.67 36.73 1.0000
-1 3 -2 31920 2195
57429 42.67 36.73 1.0000
-1 3 -2 29776 2192
54522 42.67 36.73 1.0000
1 -3 2 30212 2181

55172	42.67	-36.73	1.0000	
1	-3	2	29877	2192
54716	42.67	-36.73	1.0000	
1	-3	2	31165	2194
56465	42.67	-36.73	1.0000	
-3	3	-2	18206	772
19894	18.59	-24.41	1.0000	
-3	3	-2	16844	756
18421	18.59	-24.41	1.0000	
3	-3	2	16505	769
18065	18.59	24.41	1.0000	
-5	3	-2	22916	1908
48015	-0.67	59.94	1.0000	
-5	3	-2	21559	1885
46035	-0.67	59.94	1.0000	
-5	3	-2	22218	1890
46998	-0.67	59.94	1.0000	
5	-3	2	23892	1887
49425	-0.67	-59.94	1.0000	
5	-3	2	22244	1908
47035	-0.67	-59.94	1.0000	
5	-3	2	23180	1890
48395	-0.67	-59.94	1.0000	
-7	3	-2	2870	102
1377	17.93	28.53	1.0000	
-7	3	-2	2628	130
1133	17.93	28.53	1.0000	
7	-3	2	2848	93
1419	17.93	-28.53	1.0000	
7	-3	2	2570	135
1132	17.93	-28.53	1.0000	
7	-3	2	2672	103
1238	17.93	-28.53	1.0000	
-9	3	-2	5428	454
10601	-9.20	23.72	1.0000	
9	-3	2	5302	493
10425	-9.20	-23.72	1.0000	
-11	3	-2	3024	120
1336	16.34	13.39	1.0000	
11	-3	2	2613	180
1049	16.34	-13.39	1.0000	
-13	3	-2	2323	139
1520	5.47	6.31	1.0000	
13	-3	2	2830	223

1936 5.47 -6.31 1.0000
-15 3 -2 976 157
1573 10.62 -13.40 1.0000
15 -3 2 895 239
1430 10.62 13.40 1.0000
17 -3 2 93 189
141 5.14 -14.37 1.0000
19 -3 2 2308 354
2973 -1.98 5.26 1.0000
21 -3 2 287 218
139 1.84 -4.33 1.0000
23 -3 2 17 256
-25 -0.43 -3.59 1.0000
25 -3 2 29 306
-187 -2.70 -3.80 1.0000
26 2 -2 27 379
64 -1.55 2.25 1.0000
24 2 -2 16 300
33 2.41 -1.49 1.0000
22 2 -2 200 278
445 -1.18 6.76 1.0000
22 2 -2 63 254
-390 -1.18 6.76 1.0000
20 2 -2 626 241
338 1.23 -5.62 1.0000
20 2 -2 44 215
-225 1.23 -5.62 1.0000
18 2 -2 99 211
692 5.98 13.40 1.0000
18 2 -2 80 242
639 5.98 13.40 1.0000
16 2 -2 1274 173
293 -11.34 11.43 1.0000
16 2 -2 1232 191
273 -11.34 11.43 1.0000
14 2 -2 913 209
1517 10.85 3.24 1.0000
14 2 -2 736 224
1275 10.85 3.24 1.0000
12 2 -2 1191 248
2727 -1.16 18.62 1.0000
12 2 -2 1346 223
2969 -1.16 18.62 1.0000
10 2 -2 5696 421

8846	-14.07	-19.46	1.0000	
10	2	-2	6291	451
9606	-14.07	-19.46	1.0000	
-10	-2	2	5692	417
8877	-14.07	19.46	1.0000	
8	2	-2	4368	163
1593	39.07	-12.20	1.0000	
8	2	-2	4723	157
1898	39.07	-12.20	1.0000	
-8	-2	2	4477	141
1685	39.07	12.20	1.0000	
-8	-2	2	4133	158
1392	39.07	12.20	1.0000	
6	2	-2	12517	877
21577	-26.28	-20.26	1.0000	
6	2	-2	10930	867
19460	-26.28	-20.26	1.0000	
-6	-2	2	12135	868
21044	-26.28	20.26	1.0000	
-6	-2	2	13220	877
22476	-26.28	20.26	1.0000	
4	2	-2	74928	3580
89758	-36.59	3.48	1.0000	
4	2	-2	69483	3580
83746	-36.59	3.48	1.0000	
-4	-2	2	82168	3583
97661	-36.59	-3.48	1.0000	
-4	-2	2	76682	3583
91633	-36.59	-3.48	1.0000	
2	2	-2	49951	1299
31480	59.42	2.65	1.0000	
2	2	-2	50214	1310
31696	59.42	2.65	1.0000	
-2	-2	2	52481	1311
33582	59.42	-2.65	1.0000	
-2	-2	2	53200	1302
34176	59.42	-2.65	1.0000	
0	2	-2	5729	1357
33537	-107.78	12.88	1.0000	
0	-2	2	6319	1350
35330	-107.78	-12.88	1.0000	
0	-2	2	5572	1356
33243	-107.78	-12.88	1.0000	
-2	2	-2	55771	1454

36391 58.94 -2.53 1.0000
-2 2 -2 55275 1457
35979 58.94 -2.53 1.0000
-2 2 -2 57023 1461
37433 58.94 -2.53 1.0000
2 -2 2 55532 1460
36172 58.94 2.53 1.0000
2 -2 2 56233 1454
36755 58.94 2.53 1.0000
2 -2 2 57224 1460
37580 58.94 2.53 1.0000
-4 2 -2 76734 3655
92172 -34.69 -56.13 1.0000
-4 2 -2 76761 3660
92203 -34.69 -56.13 1.0000
-4 2 -2 77418 3672
92945 -34.69 -56.13 1.0000
4 -2 2 78928 3657
94755 -34.69 56.13 1.0000
4 -2 2 77652 3660
93313 -34.69 56.13 1.0000
4 -2 2 76828 3673
92382 -34.69 56.13 1.0000
-6 2 -2 2153 242
5307 -16.31 16.17 1.0000
-6 2 -2 2289 264
5520 -16.31 16.17 1.0000
6 -2 2 2101 243
5224 -16.31 -16.17 1.0000
6 -2 2 2146 267
5294 -16.31 -16.17 1.0000
-8 2 -2 6987 75
388 3.99 -57.60 1.0000
8 -2 2 7209 102
437 3.99 57.60 1.0000
-10 2 -2 8666 273
5667 -12.28 -10.47 1.0000
10 -2 2 8800 320
5775 -12.28 10.47 1.0000
-12 2 -2 186 85
216 -5.26 5.90 1.0000
12 -2 2 111 134
104 -5.26 -5.90 1.0000
-14 2 -2 63 105

```
448   -10.45    -4.71  1.0000
  14   -2    2       101       176
541   -10.45     4.71  1.0000
  16   -2    2       680       211
920    -3.04    -2.45  1.0000
  18   -2    2        73       186
-88    -5.95    -4.37  1.0000
  20   -2    2       252       235
572     2.87     6.39  1.0000
  22   -2    2        20       242
29    -0.12    -0.99  1.0000
  24   -2    2        31       274
61      0.09     2.02  1.0000
  26   -2    2       239       334
379     3.54     1.24  1.0000
  25    1   -2       348       329
513     1.60    -3.09  1.0000
  23    1   -2        54       261
-458    -0.90     6.32  1.0000
  23    1   -2       350       293
475    -0.90     6.32  1.0000
  21    1   -2        29       229
-266    -4.17     2.15  1.0000
  21    1   -2       171       264
310    -4.17     2.15  1.0000
  19    1   -2       696       235
219     6.12   -10.68  1.0000
  19    1   -2       815       208
294     6.12   -10.68  1.0000
  17    1   -2      2863       274
2484    -3.02     1.54  1.0000
  17    1   -2      2720       324
2351    -3.02     1.54  1.0000
  15    1   -2       247       207
898     1.38   -14.60  1.0000
  15    1   -2       354       204
1110     1.38   -14.60  1.0000
  13    1   -2       466       159
463    10.93    -6.39  1.0000
  13    1   -2       468       125
466    10.93    -6.39  1.0000
  11    1   -2      2186       185
2299    -4.17     3.79  1.0000
  11    1   -2      2212       217
```

2326	-4.17	3.79	1.0000	
-11	-1	2	1953	177
2055	-4.17	-3.79	1.0000	
9	1	-2	9893	192
3035	-6.95	39.86	1.0000	
9	1	-2	9481	214
2804	-6.95	39.86	1.0000	
-9	-1	2	9491	185
2802	-6.95	-39.86	1.0000	
7	1	-2	6158	459
10298	7.75	-19.39	1.0000	
7	1	-2	5833	446
9876	7.75	-19.39	1.0000	
-7	-1	2	5951	446
10030	7.75	19.39	1.0000	
-7	-1	2	6389	456
10598	7.75	19.39	1.0000	
5	1	-2	41513	2362
56076	-4.37	80.34	1.0000	
5	1	-2	44864	2366
60281	-4.37	80.34	1.0000	
-5	-1	2	44550	2364
59819	-4.37	-80.34	1.0000	
-5	-1	2	46223	2365
61905	-4.37	-80.34	1.0000	
3	1	-2	28510	2183
52841	42.57	-36.78	1.0000	
3	1	-2	30074	2176
54976	42.57	-36.78	1.0000	
-3	-1	2	30985	2176
56157	42.57	36.78	1.0000	
-3	-1	2	31097	2185
56309	42.57	36.78	1.0000	
1	1	-2	48941	1275
30890	37.67	11.89	1.0000	
-1	-1	2	51679	1276
33068	37.67	-11.89	1.0000	
-1	1	-2	51134	1244
32602	37.77	-11.83	1.0000	
-1	1	-2	47906	1249
30035	37.77	-11.83	1.0000	
-1	1	-2	50180	1244
31841	37.77	-11.83	1.0000	
1	-1	2	49948	1250

31662	37.77	11.83	1.0000	
1	-1	2	49172	1243
31044	37.77	11.83	1.0000	
-3	1	-2	64265	1572
40530	4.65	56.37	1.0000	
-3	1	-2	61422	1564
38244	4.65	56.37	1.0000	
-3	1	-2	63216	1562
39685	4.65	56.37	1.0000	
3	-1	2	61127	1561
37958	4.65	-56.37	1.0000	
3	-1	2	64819	1565
40925	4.65	-56.37	1.0000	
3	-1	2	63229	1572
39645	4.65	-56.37	1.0000	
-5	1	-2	2913	420
9395	39.53	-9.31	1.0000	
-5	1	-2	3208	407
9937	39.53	-9.31	1.0000	
5	-1	2	3274	407
10039	39.53	9.31	1.0000	
5	-1	2	2848	422
9256	39.53	9.31	1.0000	
-7	1	-2	7934	835
20552	-15.49	63.47	1.0000	
-7	1	-2	8063	860
20784	-15.49	63.47	1.0000	
7	-1	2	7829	862
20485	-15.49	-63.47	1.0000	
7	-1	2	7752	835
20345	-15.49	-63.47	1.0000	
-9	1	-2	7308	522
12231	14.22	17.02	1.0000	
9	-1	2	7447	561
12413	14.22	-17.02	1.0000	
-11	1	-2	2807	167
2832	7.83	13.78	1.0000	
11	-1	2	2561	224
2546	7.83	-13.78	1.0000	
-13	1	-2	2271	156
2267	0.79	-3.07	1.0000	
13	-1	2	1876	232
1867	0.79	3.07	1.0000	
-15	1	-2	1995	187

2530	9.29	5.20	1.0000	
15	-1	2	2115	279
2667	9.29	-5.20	1.0000	
17	-1	2	455	286
1766	4.70	18.57	1.0000	
19	-1	2	279	234
585	-1.27	-9.93	1.0000	
21	-1	2	298	233
276	0.57	0.15	1.0000	
23	-1	2	274	282
247	-0.43	-0.87	1.0000	
25	-1	2	56	323
30	-0.37	-2.76	1.0000	
26	0	-2	4	325
-67	-0.41	1.71	1.0000	
24	0	-2	380	309
337	0.36	0.96	1.0000	
22	0	-2	57	258
-30	2.82	-5.93	1.0000	
20	0	-2	434	264
902	-1.51	7.85	1.0000	
20	0	-2	406	223
861	-1.51	7.85	1.0000	
18	0	-2	280	221
255	-2.28	-0.02	1.0000	
18	0	-2	38	168
25	-2.28	-0.02	1.0000	
16	0	-2	883	192
554	3.94	3.62	1.0000	
16	0	-2	809	156
496	3.94	3.62	1.0000	
14	0	-2	63	134
594	-11.05	21.64	1.0000	
14	0	-2	76	159
509	-11.05	21.64	1.0000	
12	0	-2	329	152
839	-4.42	-19.68	1.0000	
12	0	-2	229	146
495	-4.42	-19.68	1.0000	
10	0	-2	504	110
208	7.55	-7.13	1.0000	
10	0	-2	660	99
326	7.55	-7.13	1.0000	
-10	0	2	432	87

160	7.55	7.13	1.0000	
8	0	-2	3723	434
9531	-31.67	-5.67	1.0000	
8	0	-2	3689	420
9476	-31.67	-5.67	1.0000	
-8	0	2	3779	431
9632	-31.67	5.67	1.0000	
-8	0	2	3307	417
8867	-31.67	5.67	1.0000	
6	0	-2	18479	1707
42046	19.50	-56.68	1.0000	
6	0	-2	18546	1703
42148	19.50	-56.68	1.0000	
-6	0	2	19176	1705
43095	19.50	56.68	1.0000	
-6	0	2	19015	1703
42853	19.50	56.68	1.0000	
4	0	-2	69379	2972
76011	14.83	1.48	1.0000	
4	0	-2	65917	2976
72382	14.83	1.48	1.0000	
-4	0	2	68586	2977
75149	14.83	-1.48	1.0000	
-4	0	2	70756	2971
77422	14.83	-1.48	1.0000	
2	0	-2	6232	1432
35021	-107.62	-12.83	1.0000	
-2	0	2	6972	1433
36846	-107.62	12.83	1.0000	
0	0	-2	2	487
11656	94.70	0.21	1.0000	
0	0	2	1	488
12132	94.70	-0.21	1.0000	
-2	0	-2	7334	1451
37883	-107.45	-12.97	1.0000	
-2	0	-2	6236	1446
34986	-107.45	-12.97	1.0000	
-2	0	-2	6617	1448
36012	-107.45	-12.97	1.0000	
2	0	2	7017	1451
36916	-107.45	12.97	1.0000	
2	0	2	7124	1448
37194	-107.45	12.97	1.0000	
-4	0	-2	68520	3068

74674 13.84 1.33 1.0000
-4 0 -2 73366 3081
79734 13.84 1.33 1.0000
4 0 2 71367 3081
77618 13.84 -1.33 1.0000
4 0 2 73133 3070
79461 13.84 -1.33 1.0000
-6 0 -2 18010 1679
41755 19.31 -57.67 1.0000
-6 0 -2 18093 1659
41882 19.31 -57.67 1.0000
6 0 2 18824 1660
42991 19.31 57.67 1.0000
6 0 2 16981 1679
40181 19.31 57.67 1.0000
-8 0 -2 3237 404
8597 -31.11 -5.18 1.0000
-8 0 -2 3667 373
9293 -31.11 -5.18 1.0000
8 0 2 2544 368
7448 -31.11 5.18 1.0000
8 0 2 3344 409
8784 -31.11 5.18 1.0000
-10 0 -2 586 75
228 7.38 -8.33 1.0000
10 0 2 433 111
129 7.38 8.33 1.0000
-12 0 -2 319 108
792 -4.25 -19.43 1.0000
12 0 2 218 152
427 -4.25 19.43 1.0000
-14 0 -2 58 109
648 -11.29 21.32 1.0000
14 0 2 65 174
585 -11.29 -21.32 1.0000
-16 0 -2 824 117
502 3.94 3.79 1.0000
16 0 2 720 183
422 3.94 -3.79 1.0000
18 0 2 8 189
-59 -2.52 0.09 1.0000
20 0 2 295 258
699 -1.11 -7.97 1.0000
22 0 2 62 245

```
-10      2.82      6.21  1.0000
  24    0    2       306       281
265      0.73    -1.02  1.0000
  26    0    2         3       308
-132    -0.29    -1.58  1.0000
  25   -1   -2        42       314
19    -0.37     2.72  1.0000
  23   -1   -2       330       279
296     -0.52     0.92  1.0000
  21   -1   -2       143       246
125      0.64    -0.20  1.0000
  21   -1   -2       172       215
153      0.64    -0.20  1.0000
  19   -1   -2       285       238
601     -1.20    10.15  1.0000
  19   -1   -2       323       201
662     -1.20    10.15  1.0000
  17   -1   -2       523       243
1882      4.53   -18.50  1.0000
  17   -1   -2       325       250
1470      4.53   -18.50  1.0000
  15   -1   -2      2343       288
2976      9.38      5.56  1.0000
  15   -1   -2      2462       246
3113      9.38      5.56  1.0000
  13   -1   -2      2204       231
2260      0.21     -3.05  1.0000
  13   -1   -2      2221       200
2277      0.21     -3.05  1.0000
  11   -1   -2      3387       224
3486      8.31     14.37  1.0000
  11   -1   -2      3515       252
3623      8.31     14.37  1.0000
   9   -1   -2      6315       474
10881     13.55     17.47  1.0000
   9   -1   -2      5874       488
10298     13.55     17.47  1.0000
   7   -1   -2      8066       872
20770   -14.67     64.22  1.0000
   7   -1   -2      8310       867
21207   -14.67     64.22  1.0000
  -7    1    2      8282       871
21281   -14.67    -64.22  1.0000
  -7    1    2      7955       864
```

20694	-14.67	-64.22	1.0000	
5	-1	-2	4036	484
11207	38.57	-9.38	1.0000	
5	-1	-2	4201	488
11488	38.57	-9.38	1.0000	
-5	1	2	4189	486
11450	38.57	9.38	1.0000	
-5	1	2	4173	482
11423	38.57	9.38	1.0000	
3	-1	-2	56591	1408
34185	4.38	56.77	1.0000	
3	-1	-2	57381	1398
34810	4.38	56.77	1.0000	
-3	1	2	59584	1409
36512	4.38	-56.77	1.0000	
-3	1	2	58000	1398
35254	4.38	-56.77	1.0000	
1	-1	-2	50643	1309
32227	37.74	-11.80	1.0000	
-1	1	2	52115	1309
33409	37.74	11.80	1.0000	
-1	-1	-2	49412	1253
31247	37.81	11.66	1.0000	
-1	-1	-2	49087	1248
30989	37.81	11.66	1.0000	
1	1	2	51902	1254
33228	37.81	-11.66	1.0000	
1	1	2	48690	1248
30668	37.81	-11.66	1.0000	
-3	-1	-2	30423	2145
55546	43.09	-36.65	1.0000	
-3	-1	-2	28199	2136
52507	43.09	-36.65	1.0000	
3	1	2	28748	2145
53203	43.09	36.65	1.0000	
3	1	2	30217	2136
55209	43.09	36.65	1.0000	
-5	-1	-2	43107	2401
58057	-4.13	79.95	1.0000	
-5	-1	-2	47054	2421
62979	-4.13	79.95	1.0000	
5	1	2	44990	2421
60342	-4.13	-79.95	1.0000	
5	1	2	46105	2403

61730 -4.13 -79.95 1.0000
-7 -1 -2 5429 411
9477 8.34 -19.92 1.0000
-7 -1 -2 5773 442
9931 8.34 -19.92 1.0000
7 1 2 5539 445
9624 8.34 19.92 1.0000
7 1 2 5520 411
9599 8.34 19.92 1.0000
-9 -1 -2 9003 149
2490 -7.95 39.76 1.0000
9 1 2 9149 208
2562 -7.95 -39.76 1.0000
-11 -1 -2 2514 160
2586 -4.20 3.30 1.0000
11 1 2 2258 222
2321 -4.20 -3.30 1.0000
-13 -1 -2 712 110
741 10.41 -6.36 1.0000
13 1 2 659 176
661 10.41 6.36 1.0000
-15 -1 -2 200 130
800 1.72 -14.47 1.0000
15 1 2 210 198
818 1.72 14.47 1.0000
-17 -1 -2 2708 193
2363 -2.88 0.93 1.0000
17 1 2 2472 313
2142 -2.88 -0.93 1.0000
19 1 2 481 225
100 5.89 10.45 1.0000
21 1 2 194 246
333 -4.12 -1.90 1.0000
23 1 2 135 274
188 -1.03 -6.17 1.0000
25 1 2 298 327
444 1.55 2.92 1.0000
26 -2 -2 57 337
127 3.38 -1.17 1.0000
24 -2 -2 46 291
81 -0.12 -2.00 1.0000
22 -2 -2 1 216
-97 -0.24 0.92 1.0000
22 -2 -2 390 283

429	-0.24	0.92	1.0000	
20	-2	-2	64	196
253	3.13	-6.21	1.0000	
20	-2	-2	209	237
501	3.13	-6.21	1.0000	
18	-2	-2	786	216
476	-6.52	4.39	1.0000	
18	-2	-2	344	162
139	-6.52	4.39	1.0000	
16	-2	-2	462	191
651	-2.77	2.37	1.0000	
16	-2	-2	509	163
706	-2.77	2.37	1.0000	
12	-2	-2	696	156
859	-5.23	6.23	1.0000	
10	-2	-2	8847	300
5784	-12.05	-10.81	1.0000	
10	-2	-2	8879	319
5810	-12.05	-10.81	1.0000	
8	-2	-2	6140	88
231	4.13	-56.37	1.0000	
-8	2	2	5973	80
191	4.13	56.37	1.0000	
6	-2	-2	2050	255
5082	-16.23	15.72	1.0000	
6	-2	-2	2219	256
5345	-16.23	15.72	1.0000	
-6	2	2	2022	250
5035	-16.23	-15.72	1.0000	
4	-2	-2	80549	3825
96352	-34.54	-55.84	1.0000	
4	-2	-2	79651	3831
95341	-34.54	-55.84	1.0000	
-4	2	2	83008	3826
99224	-34.54	55.84	1.0000	
2	-2	-2	51143	1324
32544	59.03	-2.55	1.0000	
2	-2	-2	52311	1315
33507	59.03	-2.55	1.0000	
-2	2	2	51950	1316
33190	59.03	2.55	1.0000	
0	-2	-2	6480	1421
35413	-107.30	12.83	1.0000	
0	-2	-2	6724	1414

36065 -107.30 12.83 1.0000
0 2 2 6519 1414
35668 -107.30 -12.83 1.0000
-2 -2 -2 53095 1349
34352 58.60 2.85 1.0000
2 2 2 51781 1348
33283 58.60 -2.85 1.0000
-4 -2 -2 81513 3775
97179 -36.47 3.00 1.0000
-4 -2 -2 77705 3758
92991 -36.47 3.00 1.0000
4 2 2 82555 3758
98266 -36.47 -3.00 1.0000
4 2 2 78219 3775
93501 -36.47 -3.00 1.0000
-6 -2 -2 11883 844
20704 -26.29 -19.89 1.0000
-6 -2 -2 11108 816
19670 -26.29 -19.89 1.0000
6 2 2 11728 817
20473 -26.29 19.89 1.0000
6 2 2 11281 845
19876 -26.29 19.89 1.0000
-8 -2 -2 5003 131
2198 38.64 -12.61 1.0000
8 2 2 4640 179
1879 38.64 12.61 1.0000
8 2 2 4855 136
2067 38.64 12.61 1.0000
-10 -2 -2 6671 429
9895 -13.18 -19.61 1.0000
10 2 2 6537 485
9765 -13.18 19.61 1.0000
-12 -2 -2 2053 202
3976 -1.36 18.17 1.0000
12 2 2 1391 279
3008 -1.36 -18.17 1.0000
-14 -2 -2 758 135
1315 11.24 3.12 1.0000
14 2 2 801 221
1366 11.24 -3.12 1.0000
-16 -2 -2 583 110
25 -12.04 11.44 1.0000
16 2 2 367 167

-42	-12.04	-11.44	1.0000	
-18	-2	-2	94	155
674	6.23	13.25	1.0000	
18	2	2	93	236
672	6.23	-13.25	1.0000	
20	2	2	695	246
386	1.21	5.70	1.0000	
22	2	2	212	271
467	-0.92	-6.81	1.0000	
24	2	2	242	300
317	2.37	1.62	1.0000	
26	2	2	9	355
-117	-1.31	-2.17	1.0000	
25	-3	-2	19	312
30	-2.75	3.83	1.0000	
23	-3	-2	206	276
166	-0.43	3.41	1.0000	
21	-3	-2	388	194
207	1.54	4.48	1.0000	
21	-3	-2	833	253
556	1.54	4.48	1.0000	
19	-3	-2	2660	310
3359	-2.09	-5.14	1.0000	
19	-3	-2	2610	365
3303	-2.09	-5.14	1.0000	
17	-3	-2	94	153
119	5.35	14.34	1.0000	
17	-3	-2	81	197
327	5.35	14.34	1.0000	
15	-3	-2	1066	209
1662	10.19	-13.47	1.0000	
15	-3	-2	1080	255
1682	10.19	-13.47	1.0000	
13	-3	-2	3174	228
2139	5.60	7.09	1.0000	
13	-3	-2	3184	203
2147	5.60	7.09	1.0000	
11	-3	-2	2497	179
992	15.45	13.19	1.0000	
11	-3	-2	3347	163
1588	15.45	13.19	1.0000	
9	-3	-2	5952	520
11364	-8.47	24.20	1.0000	
9	-3	-2	5951	508

11363	-8.47	24.20	1.0000		
7	-3	-2	2868	134	
1437	17.23	28.79	1.0000		
7	-3	-2	2907	132	
1477	17.23	28.79	1.0000		
-7	3	2	2821	133	
1453	17.23	-28.79	1.0000		
5	-3	-2	21795	1884	
46520	-0.29	60.29	1.0000		
5	-3	-2	22203	1884	
47117	-0.29	60.29	1.0000		
-5	3	2	22576	1883	
47660	-0.29	-60.29	1.0000		
3	-3	-2	17235	780	
18764	18.28	-23.87	1.0000		
-3	3	2	17894	782	
19486	18.28	23.87	1.0000		
1	-3	-2	31489	2234	
56967	42.96	36.80	1.0000		
1	-3	-2	30616	2229	
55784	42.96	36.80	1.0000		
-1	-3	-2	58147	1436	
35576	4.55	-56.31	1.0000		
1	3	2	59329	1439	
36566	4.55	56.31	1.0000		
-3	-3	-2	16168	751	
17598	18.88	24.21	1.0000		
3	3	2	17369	754	
18885	18.88	-24.21	1.0000		
-5	-3	-2	19954	243	
5272	14.16	-57.87	1.0000		
-5	-3	-2	20045	271	
5319	14.16	-57.87	1.0000		
5	3	2	20424	277	
5514	14.16	57.87	1.0000		
5	3	2	20532	242	
5570	14.16	57.87	1.0000		
-7	-3	-2	14551	1481	
36184	-14.39	58.77	1.0000		
-7	-3	-2	14194	1446	
35619	-14.39	58.77	1.0000		
7	3	2	14913	1447	
36758	-14.39	-58.77	1.0000		
7	3	2	14664	1484	

36365	-14.39	-58.77	1.0000	
-9	-3	-2	5287	192
3773	-4.03	-13.30	1.0000	
9	3	2	4993	252
3528	-4.03	13.30	1.0000	
-11	-3	-2	804	130
1675	-10.04	4.39	1.0000	
11	3	2	730	210
1566	-10.04	-4.39	1.0000	
-13	-3	-2	3123	178
2922	-4.83	-4.56	1.0000	
13	3	2	2643	260
2449	-4.83	4.56	1.0000	
-15	-3	-2	168	135
1036	1.51	-16.83	1.0000	
15	3	2	172	216
1047	1.51	16.83	1.0000	
-17	-3	-2	681	175
1468	7.09	-10.09	1.0000	
17	3	2	1075	313
2048	7.09	10.09	1.0000	
19	3	2	758	260
802	-7.44	1.14	1.0000	
21	3	2	479	259
330	2.95	1.36	1.0000	
23	3	2	20	308
105	-1.17	4.89	1.0000	
25	3	2	27	334
-122	-3.21	-3.16	1.0000	
26	-4	-2	123	344
101	-0.86	-2.18	1.0000	
24	-4	-2	1	282
31	5.09	-2.40	1.0000	
24	-4	-2	42	260
-123	5.09	-2.40	1.0000	
22	-4	-2	1	235
27	-2.13	-3.32	1.0000	
22	-4	-2	158	248
292	-2.13	-3.32	1.0000	
20	-4	-2	642	197
597	-2.69	-1.65	1.0000	
20	-4	-2	542	245
500	-2.69	-1.65	1.0000	
18	-4	-2	34	220

396 6.50 -10.61 1.0000
18 -4 -2 126 189
645 6.50 -10.61 1.0000
16 -4 -2 1376 320
3547 -20.37 9.11 1.0000
16 -4 -2 1204 276
3257 -20.37 9.11 1.0000
14 -4 -2 2294 215
2229 2.72 -5.93 1.0000
14 -4 -2 2158 237
2093 2.72 -5.93 1.0000
12 -4 -2 9139 445
8428 -12.09 -1.31 1.0000
12 -4 -2 9445 420
8725 -12.09 -1.31 1.0000
10 -4 -2 1335 145
966 -19.36 -11.28 1.0000
10 -4 -2 998 127
557 -19.36 -11.28 1.0000
8 -4 -2 7022 224
3794 19.20 1.59 1.0000
8 -4 -2 7346 236
4033 19.20 1.59 1.0000
6 -4 -2 10717 1084
26656 -18.15 -60.76 1.0000
-6 4 2 10385 1083
26121 -18.15 60.76 1.0000
4 -4 -2 41157 1683
41801 13.10 16.38 1.0000
-4 4 2 41519 1684
42121 13.10 -16.38 1.0000
2 -4 -2 73576 3591
88374 -36.50 -3.05 1.0000
-2 4 2 78106 3597
93422 -36.50 3.05 1.0000
0 -4 -2 69160 2994
75443 14.15 -1.40 1.0000
0 4 2 69741 2996
76081 14.15 1.40 1.0000
-2 -4 -2 73666 3488
88856 -34.57 56.15 1.0000
2 4 2 72652 3488
87605 -34.57 -56.15 1.0000
-4 -4 -2 42051 1639

42537	12.94	-16.61	1.0000	
-4	-4	-2	40932	1662
41403	12.94	-16.61	1.0000	
4	4	2	39499	1661
39999	12.94	16.61	1.0000	
-6	-4	-2	13578	992
23912	15.00	30.18	1.0000	
-6	-4	-2	14641	1029
25318	15.00	30.18	1.0000	
6	4	2	14362	993
24962	15.00	-30.18	1.0000	
6	4	2	14141	1029
24670	15.00	-30.18	1.0000	
-8	-4	-2	11762	307
6817	-16.59	-27.25	1.0000	
8	4	2	11941	310
6968	-16.59	27.25	1.0000	
8	4	2	11810	362
6864	-16.59	27.25	1.0000	
-10	-4	-2	5956	416
9805	18.98	0.45	1.0000	
10	4	2	5495	480
9214	18.98	-0.45	1.0000	
-12	-4	-2	2089	218
3944	12.05	8.79	1.0000	
12	4	2	2469	325
4462	12.05	-8.79	1.0000	
-14	-4	-2	1219	128
951	-8.83	3.14	1.0000	
14	4	2	1230	228
962	-8.83	-3.14	1.0000	
-16	-4	-2	427	140
894	9.62	17.77	1.0000	
16	4	2	278	240
524	9.62	-17.77	1.0000	
-18	-4	-2	216	137
162	-3.01	0.61	1.0000	
18	4	2	13	228
-84	-3.01	-0.61	1.0000	
20	4	2	216	275
249	-0.85	-9.40	1.0000	
22	4	2	301	284
303	0.48	0.84	1.0000	
24	4	2	15	289

-494		-0.21	-3.36	1.0000
26	4	2	3	350
-239		0.47	1.41	1.0000
25	-5	-2	1	289
-310		-0.83	0.03	1.0000
25	-5	-2	122	299
114		-0.83	0.03	1.0000
23	-5	-2	135	273
211		0.64	2.44	1.0000
23	-5	-2	8	239
-55		0.64	2.44	1.0000
21	-5	-2	84	234
-60		-4.05	6.83	1.0000
21	-5	-2	84	209
-33		-4.05	6.83	1.0000
19	-5	-2	12	221
147		2.55	7.16	1.0000
19	-5	-2	1	167
78		2.55	7.16	1.0000
17	-5	-2	563	205
514		5.23	-7.01	1.0000
17	-5	-2	426	178
369		5.23	-7.01	1.0000
15	-5	-2	100	143
403		-0.34	9.17	1.0000
15	-5	-2	65	171
325		-0.34	9.17	1.0000
13	-5	-2	978	227
2021		20.99	-2.99	1.0000
13	-5	-2	1053	209
2146		20.99	-2.99	1.0000
9	-5	-2	1797	187
2030		0.66	24.73	1.0000
7	-5	-2	4345	170
2191		2.33	25.19	1.0000
-7	5	2	4354	173
2202		2.33	-25.19	1.0000
5	-5	-2	166	78
79		-13.82	-5.88	1.0000
-5	5	2	165	87
138		-13.82	5.88	1.0000
3	-5	-2	20501	278
5549		14.55	57.78	1.0000
-3	5	2	21299	289

5969   14.55   -57.78   1.0000
    1   -5   -2   42997   2316
57667   -4.45   -79.83   1.0000
    1   -5   -2   43556   2308
58366   -4.45   -79.83   1.0000
   -1   -5   -2   2809   406
9189   39.57   9.31   1.0000
    1   5   2   2767   406
9128   39.57   -9.31   1.0000
   -3   -5   -2   20681   1779
44716   -0.68   -59.89   1.0000
    3   5   2   20125   1779
43896   -0.68   59.89   1.0000
   -5   -5   -2   195   60
290   -13.29   6.29   1.0000
   -5   -5   -2   288   91
-56   -13.29   6.29   1.0000
    5   5   2   257   104
2   -13.29   -6.29   1.0000
   -7   -5   -2   3993   273
6167   -2.33   -37.43   1.0000
    7   5   2   3757   327
5775   -2.33   37.43   1.0000
    7   5   2   4121   276
6318   -2.33   37.43   1.0000
   -9   -5   -2   4413   95
703   -20.78   27.86   1.0000
    9   5   2   4207   94
624   -20.78   -27.86   1.0000
    9   5   2   3769   146
462   -20.78   -27.86   1.0000
  -11   -5   -2   4196   289
6157   -6.01   -10.23   1.0000
   11   5   2   4088   377
6026   -6.01   10.23   1.0000
  -13   -5   -2   495   98
485   -5.14   -10.23   1.0000
   13   5   2   383   183
328   -5.14   10.23   1.0000
  -15   -5   -2   383   101
230   3.14   2.31   1.0000
   15   5   2   628   210
426   3.14   -2.31   1.0000
  -17   -5   -2   2335   258

4171	-4.33	-14.53	1.0000	
17	5	2	2240	402
4038	-4.33	14.53	1.0000	
19	5	2	443	255
189	3.65	5.22	1.0000	
21	5	2	135	268
165	0.30	1.77	1.0000	
23	5	2	27	298
33	-4.49	-0.81	1.0000	
25	5	2	5	326
-143	1.62	1.12	1.0000	
24	-6	-2	36	271
89	-2.82	-1.11	1.0000	
24	-6	-2	12	271
-337	-2.82	-1.11	1.0000	
22	-6	-2	100	242
-149	4.95	-7.10	1.0000	
22	-6	-2	100	215
-20	4.95	-7.10	1.0000	
20	-6	-2	79	206
-44	1.41	-7.55	1.0000	
20	-6	-2	33	224
24	1.41	-7.55	1.0000	
18	-6	-2	1932	273
2193	-12.79	6.36	1.0000	
18	-6	-2	2082	319
2363	-12.79	6.36	1.0000	
16	-6	-2	142	185
1035	11.36	-18.07	1.0000	
16	-6	-2	158	231
1089	11.36	-18.07	1.0000	
14	-6	-2	44	204
1440	-20.67	17.70	1.0000	
14	-6	-2	26	240
1326	-20.67	17.70	1.0000	
12	-6	-2	4940	263
3140	-11.96	3.11	1.0000	
12	-6	-2	5584	245
3657	-11.96	3.11	1.0000	
10	-6	-2	4428	255
3677	27.24	-12.75	1.0000	
8	-6	-2	3793	423
8732	-37.46	-8.24	1.0000	
6	-6	-2	2533	105

```
496    23.33    14.44  1.0000
  -6    6    2     2388      113
395    23.33  -14.44  1.0000
   4   -6   -2    13587     1005
23869    14.98   -29.96  1.0000
  -4    6    2    14238     1013
24719    14.98    29.96  1.0000
   2   -6   -2    11546      844
20154   -26.05    19.88  1.0000
   0   -6   -2    16248     1592
39118    19.50    57.78  1.0000
   0    6    2    16843     1595
40039    19.50   -57.78  1.0000
  -2   -6   -2     1714      239
4615   -16.45   -16.16  1.0000
   2    6    2     1470      239
4210   -16.45    16.16  1.0000
  -4   -6   -2    10347     1020
26394   -18.37    61.78  1.0000
  -4   -6   -2     9783     1058
25434   -18.37    61.78  1.0000
  -4   -6   -2     9807     1026
25475   -18.37    61.78  1.0000
   4    6    2     9391     1055
24739   -18.37   -61.78  1.0000
  -6   -6   -2     2651       72
491    23.94   -14.52  1.0000
  -6   -6   -2     2471       79
409    23.94   -14.52  1.0000
   6    6    2     2226      129
333    23.94    14.52  1.0000
  -8   -6   -2      632      102
1061      2.85      6.25  1.0000
   8    6    2      614      186
1037      2.85     -6.25  1.0000
   8    6    2      685      110
1128      2.85     -6.25  1.0000
 -10   -6   -2     1858      115
1169     15.31      6.82  1.0000
 -12   -6   -2     4177      172
2732      2.40   -18.81  1.0000
  12    6    2     4002      264
2605      2.40    18.81  1.0000
 -14   -6   -2      366      151
```

```
1477     2.04    17.84  1.0000
  14   6    2      619      278
1967     2.04   -17.84  1.0000
 -16  -6   -2      389      122
401    1.56    -0.73  1.0000
  16   6    2      613      239
628    1.56     0.73  1.0000
 -18  -6   -2      169      139
363   -1.39     7.78  1.0000
  18   6    2       49      223
137   -1.39    -7.78  1.0000
  20   6    2      298      261
574   -3.34    -5.02  1.0000
  22   6    2      530      299
606    1.76     1.43  1.0000
  24   6    2      255      313
252   -2.54     0.55  1.0000
  25  -7   -2       11      292
-415     2.68     1.02  1.0000
  25  -7   -2       12      309
9     2.68     1.02  1.0000
  23  -7   -2      139      263
306   -2.96     4.09  1.0000
  23  -7   -2      278      290
502   -2.96     4.09  1.0000
  21  -7   -2      341      230
414   -0.23     2.05  1.0000
  21  -7   -2      277      253
343   -0.23     2.05  1.0000
  19  -7   -2      512      254
856    0.43     5.79  1.0000
  19  -7   -2      703      234
1098     0.43     5.79  1.0000
  17  -7   -2      162      171
262   -1.90     5.23  1.0000
  17  -7   -2      245      203
372   -1.90     5.23  1.0000
  15  -7   -2     2742      223
1577    16.17    -6.14  1.0000
  15  -7   -2     2447      226
1341    16.17    -6.14  1.0000
  13  -7   -2     1914      221
1472    -1.22    -6.91  1.0000
  11  -7   -2     1563      208
```

1723 -0.06 1.71 1.0000
9 -7 -2 2156 218
2617 7.23 9.00 1.0000
7 -7 -2 1699 110
438 -17.72 -3.67 1.0000
5 -7 -2 3779 295
5908 -1.72 37.59 1.0000
-5 7 2 3143 294
4959 -1.72 -37.59 1.0000
3 -7 -2 13016 1402
33754 -14.43 -58.79 1.0000
-3 7 2 13702 1414
34850 -14.43 58.79 1.0000
1 -7 -2 5921 448
10095 8.23 19.79 1.0000
1 -7 -2 5556 459
9614 8.23 19.79 1.0000
-1 -7 -2 7903 858
20584 -15.34 -63.47 1.0000
1 7 2 7823 857
20317 -15.34 63.47 1.0000
-3 -7 -2 2596 160
1141 18.08 -28.35 1.0000
-3 -7 -2 3102 110
1658 18.08 -28.35 1.0000
3 7 2 2801 169
1289 18.08 28.35 1.0000
-5 -7 -2 4664 141
2534 1.40 -25.31 1.0000
-5 -7 -2 4475 131
2384 1.40 -25.31 1.0000
5 7 2 3804 190
1854 1.40 25.31 1.0000
-7 -7 -2 1593 74
382 -17.60 3.98 1.0000
-7 -7 -2 1647 72
409 -17.60 3.98 1.0000
7 7 2 1194 130
199 -17.60 -3.98 1.0000
-9 -7 -2 11251 402
9706 -4.70 -24.69 1.0000
9 7 2 10502 403
8993 -4.70 24.69 1.0000
9 7 2 10415 469

8909	-4.70	24.69	1.0000	
-11	-7	-2	1402	230
4580	-18.09	19.76	1.0000	
11	7	2	1249	327
4303	-18.09	-19.76	1.0000	
-13	-7	-2	595	122
849	5.22	4.28	1.0000	
13	7	2	711	215
991	5.22	-4.28	1.0000	
-15	-7	-2	283	123
536	-0.01	-15.81	1.0000	
15	7	2	210	208
366	-0.01	15.81	1.0000	
-17	-7	-2	298	141
231	-0.13	4.99	1.0000	
17	7	2	66	208
22	-0.13	-4.99	1.0000	
19	7	2	106	249
-115	6.52	6.08	1.0000	
21	7	2	87	264
-12	-7.21	3.69	1.0000	
23	7	2	250	279
134	1.68	-4.10	1.0000	
25	7	2	238	318
227	0.78	-1.14	1.0000	
24	-8	-2	435	322
494	0.01	-2.44	1.0000	
24	-8	-2	34	275
46	0.01	-2.44	1.0000	
22	-8	-2	1091	271
1029	0.34	-0.74	1.0000	
22	-8	-2	1	249
-113	0.34	-0.74	1.0000	
20	-8	-2	123	219
101	1.97	-4.03	1.0000	
20	-8	-2	27	205
-5	1.97	-4.03	1.0000	
18	-8	-2	1052	228
1129	2.49	-12.95	1.0000	
18	-8	-2	791	251
823	2.49	-12.95	1.0000	
16	-8	-2	118	243
1125	-15.02	16.22	1.0000	
16	-8	-2	124	233

1148 -15.02 16.22 1.0000

14 -8 -2 7 151

80 4.59 -6.92 1.0000

12 -8 -2 440 177

1041 3.88 10.72 1.0000

10 -8 -2 708 270

3693 -21.72 20.15 1.0000

8 -8 -2 1797 252

3630 32.10 -12.26 1.0000

6 -8 -2 606 153

1051 3.31 -6.29 1.0000

4 -8 -2 11365 335

6465 -16.86 26.94 1.0000

2 -8 -2 4455 174

1707 38.83 12.56 1.0000

2 -8 -2 4324 167

1594 38.83 12.56 1.0000

0 -8 -2 3740 438

9440 -31.20 5.18 1.0000

-2 -8 -2 6436 66

247 4.09 57.61 1.0000

-2 -8 -2 6468 106

254 4.09 57.61 1.0000

2 8 2 6596 106

291 4.09 -57.61 1.0000

-4 -8 -2 6687 190

3497 19.53 -1.94 1.0000

-6 -8 -2 3271 357

7825 -37.75 8.57 1.0000

-6 -8 -2 3823 374

8756 -37.75 8.57 1.0000

6 8 2 3570 421

8395 -37.75 -8.57 1.0000

-8 -8 -2 2015 205

4018 32.61 12.32 1.0000

-8 -8 -2 1790 191

3598 32.61 12.32 1.0000

8 8 2 1710 283

3524 32.61 -12.32 1.0000

-10 -8 -2 3 102

725 -2.10 -23.95 1.0000

10 8 2 19 180

557 -2.10 23.95 1.0000

-12 -8 -2 292 100

197	0.66	3.00	1.0000	
12	8	2	327	185
225	0.66	-3.00	1.0000	
-14	-8	-2	258	134
957	11.69	-6.06	1.0000	
14	8	2	239	250
918	11.69	6.06	1.0000	
-16	-8	-2	756	132
530	-11.01	0.87	1.0000	
16	8	2	600	223
383	-11.01	-0.87	1.0000	
-18	-8	-2	306	160
676	-1.82	7.14	1.0000	
18	8	2	247	244
587	-1.82	-7.14	1.0000	
20	8	2	641	266
400	-0.06	4.94	1.0000	
22	8	2	22	263
-152	-3.59	-1.95	1.0000	
24	8	2	26	302
-64	-1.74	4.08	1.0000	
25	-9	-2	5	287
-232	0.47	1.95	1.0000	
25	-9	-2	393	339
477	0.47	1.95	1.0000	
23	-9	-2	2	273
-6	0.58	1.02	1.0000	
23	-9	-2	2	279
-2	0.58	1.02	1.0000	
21	-9	-2	17	236
21	-2.72	4.60	1.0000	
21	-9	-2	65	245
134	-2.72	4.60	1.0000	
19	-9	-2	300	233
214	-2.08	1.28	1.0000	
19	-9	-2	597	209
473	-2.08	1.28	1.0000	
17	-9	-2	226	200
157	8.23	2.11	1.0000	
17	-9	-2	338	183
278	8.23	2.11	1.0000	
15	-9	-2	352	185
295	-6.50	-3.26	1.0000	
13	-9	-2	3265	350

4801 7.87 -10.63 1.0000
9 -9 -2 6 155
707 -14.69 -19.37 1.0000
7 -9 -2 11627 494
10134 -4.46 24.82 1.0000
5 -9 -2 3762 127
451 -20.75 -28.07 1.0000
3 -9 -2 4746 238
3291 -3.95 13.48 1.0000
1 -9 -2 7506 194
1721 -7.87 -39.76 1.0000
1 9 2 6411 516
11067 14.28 17.00 1.0000
3 9 2 4920 473
9852 -9.16 23.58 1.0000
-5 -9 -2 1742 126
1924 1.16 -24.42 1.0000
-5 -9 -2 1874 151
2099 1.16 -24.42 1.0000
5 9 2 1837 232
2022 1.16 24.42 1.0000
-7 -9 -2 2604 163
3120 6.68 -8.62 1.0000
-7 -9 -2 2456 184
2954 6.68 -8.62 1.0000
7 9 2 1981 248
2409 6.68 8.62 1.0000
-9 -9 -2 16 108
906 -15.02 19.30 1.0000
-9 -9 -2 16 124
912 -15.02 19.30 1.0000
9 9 2 33 176
523 -15.02 -19.30 1.0000
-11 -9 -2 3778 192
2692 11.24 -3.41 1.0000
-11 -9 -2 3242 163
2236 11.24 -3.41 1.0000
11 9 2 3075 281
2088 11.24 3.41 1.0000
-13 -9 -2 618 104
132 -6.82 9.53 1.0000
13 9 2 517 202
88 -6.82 -9.53 1.0000
-15 -9 -2 1875 153

1146	-2.16	7.91	1.0000	
15	9	2	1940	283
1197	-2.16	-7.91	1.0000	
-17	-9	-2	389	188
1333	11.92	-8.34	1.0000	
17	9	2	518	305
1564	11.92	8.34	1.0000	
19	9	2	330	289
514	-8.02	-0.97	1.0000	
21	9	2	42	298
207	4.63	-5.06	1.0000	
23	9	2	18	318
-282	2.87	2.28	1.0000	
25	9	2	21	359
-69	-3.02	-2.57	1.0000	
24	-10	-2	125	307
182	0.64	-2.83	1.0000	
24	-10	-2	294	303
381	0.64	-2.83	1.0000	
22	-10	-2	53	267
88	1.16	-2.41	1.0000	
22	-10	-2	10	258
-173	1.16	-2.41	1.0000	
20	-10	-2	562	273
609	-1.47	-1.52	1.0000	
20	-10	-2	333	229
368	-1.47	-1.52	1.0000	
18	-10	-2	438	223
312	3.08	0.49	1.0000	
18	-10	-2	285	186
185	3.08	0.49	1.0000	
16	-10	-2	943	239
1109	3.21	-6.03	1.0000	
14	-10	-2	901	181
175	-7.02	19.83	1.0000	
12	-10	-2	951	186
760	-0.01	2.86	1.0000	
10	-10	-2	1938	210
1591	5.09	6.59	1.0000	
8	-10	-2	2	157
767	-1.99	23.84	1.0000	
6	-10	-2	1858	180
1153	15.17	-6.98	1.0000	
4	-10	-2	5564	464

9351	19.21	-0.26	1.0000	
2	-10	-2	6282	473
9436	-13.18	19.43	1.0000	
2	10	2	8339	325
5424	-12.18	-10.39	1.0000	
4	10	2	985	155
498	-19.34	-12.29	1.0000	
-6	-10	-2	3990	171
3232	27.31	12.49	1.0000	
-8	-10	-2	707	195
3707	-21.85	-20.20	1.0000	
8	10	2	773	293
3857	-21.85	20.20	1.0000	
-10	-10	-2	1217	117
920	5.38	-6.75	1.0000	
10	10	2	1301	206
989	5.38	6.75	1.0000	
-12	-10	-2	1102	186
1660	6.91	-6.03	1.0000	
-12	-10	-2	726	141
1179	6.91	-6.03	1.0000	
12	10	2	862	261
1357	6.91	6.03	1.0000	
-14	-10	-2	334	167
1814	-14.18	-16.14	1.0000	
14	10	2	392	337
1951	-14.18	16.14	1.0000	
-16	-10	-2	26	131
154	8.32	6.31	1.0000	
16	10	2	26	238
166	8.32	-6.31	1.0000	
-18	-10	-2	35	163
95	-5.56	-6.86	1.0000	
18	10	2	67	269
200	-5.56	6.86	1.0000	
20	10	2	190	305
213	-1.17	-1.54	1.0000	
22	10	2	58	295
-266	-5.16	4.08	1.0000	
24	10	2	79	339
62	-2.31	1.33	1.0000	
23	-11	-2	7	285
-33	2.00	1.20	1.0000	
23	-11	-2	7	274

-57 2.00 1.20 1.0000
21 -11 -2 0 235
-245 -0.36 0.28 1.0000
21 -11 -2 0 250
-336 -0.36 0.28 1.0000
19 -11 -2 427 213
279 1.18 4.79 1.0000
19 -11 -2 58 226
7 1.18 4.79 1.0000
17 -11 -2 1546 328
2193 -7.30 -0.74 1.0000
15 -11 -2 75 206
272 5.62 -11.20 1.0000
13 -11 -2 2204 273
2015 1.63 1.94 1.0000
11 -11 -2 4138 369
5505 -4.18 -21.50 1.0000
9 -11 -2 3976 253
2842 11.38 3.45 1.0000
7 -11 -2 1267 299
4343 -18.04 -19.84 1.0000
5 -11 -2 4227 356
6202 -5.64 10.49 1.0000
3 -11 -2 686 200
1515 -10.12 -4.64 1.0000
1 -11 -2 2016 229
2079 -3.98 -3.24 1.0000
1 11 2 2976 241
3007 7.78 13.73 1.0000
3 11 2 2887 197
1244 16.32 13.34 1.0000
5 11 2 71 137
112 1.11 1.70 1.0000
-7 -11 -2 1598 129
1783 0.11 -1.98 1.0000
7 11 2 1447 239
1623 0.11 1.98 1.0000
-9 -11 -2 880 117
1048 8.35 4.25 1.0000
9 11 2 729 206
872 8.35 -4.25 1.0000
-11 -11 -2 2154 183
3022 -4.46 21.52 1.0000
11 11 2 1696 282

2405	-4.46	-21.52	1.0000	
-13	-11	-2	365	124
709	6.56	-1.38	1.0000	
13	11	2	135	234
362	6.56	1.38	1.0000	
-15	-11	-2	2	129
233	6.97	10.46	1.0000	
15	11	2	103	238
19	6.97	-10.46	1.0000	
-17	-11	-2	736	169
957	-6.93	3.40	1.0000	
17	11	2	403	296
558	-6.93	-3.40	1.0000	
19	11	2	22	262
-61	4.06	0.13	1.0000	
21	11	2	409	334
489	1.55	-0.62	1.0000	
23	11	2	50	349
128	0.23	-4.49	1.0000	
22	-12	-2	267	284
226	0.46	-1.42	1.0000	
22	-12	-2	3	268
-27	0.46	-1.42	1.0000	
20	-12	-2	89	231
194	2.02	-3.86	1.0000	
20	-12	-2	303	262
483	2.02	-3.86	1.0000	
18	-12	-2	719	259
1053	-2.89	4.04	1.0000	
16	-12	-2	225	232
553	6.15	4.28	1.0000	
14	-12	-2	226	207
369	1.86	-3.32	1.0000	
12	-12	-2	1745	172
226	-8.02	22.55	1.0000	
10	-12	-2	940	233
1450	6.94	5.93	1.0000	
8	-12	-2	304	144
199	0.94	-2.98	1.0000	
6	-12	-2	4134	244
2719	2.46	18.61	1.0000	
4	-12	-2	2252	292
4168	11.92	-9.00	1.0000	
2	-12	-2	1623	277

3361	-1.37	-18.22	1.0000	
0	-12	-2	378	196
937	-4.20	19.29	1.0000	
2	12	2	82	136
-39	-5.19	5.87	1.0000	
4	12	2	8124	415
7384	-12.70	-1.21	1.0000	
6	12	2	3152	236
1768	-11.88	2.84	1.0000	
8	12	2	310	231
832	4.02	10.72	1.0000	
-10	-12	-2	1203	122
980	-0.02	-2.95	1.0000	
10	12	2	819	221
637	-0.02	2.95	1.0000	
-12	-12	-2	1575	106
161	-8.09	-22.61	1.0000	
12	12	2	768	169
-168	-8.09	22.61	1.0000	
-14	-12	-2	276	127
381	6.50	5.07	1.0000	
14	12	2	208	234
287	6.50	-5.07	1.0000	
-16	-12	-2	399	181
1377	-9.13	-13.62	1.0000	
16	12	2	318	304
1217	-9.13	13.62	1.0000	
-18	-12	-2	6	161
-24	1.97	0.93	1.0000	
18	12	2	6	267
-229	1.97	-0.93	1.0000	
20	12	2	386	323
347	-4.13	2.22	1.0000	
22	12	2	202	350
334	-0.72	3.67	1.0000	
23	-13	-2	98	317
55	-2.11	0.46	1.0000	
23	-13	-2	517	306
411	-2.11	0.46	1.0000	
21	-13	-2	312	283
521	4.30	2.56	1.0000	
21	-13	-2	344	269
563	4.30	2.56	1.0000	
19	-13	-2	165	244

191 -0.84 -3.26 1.0000
19 -13 -2 353 235
398 -0.84 -3.26 1.0000
17 -13 -2 212 208
94 -3.37 -2.59 1.0000
15 -13 -2 74 204
262 1.74 7.40 1.0000
13 -13 -2 1613 363
4224 -1.03 -22.17 1.0000
11 -13 -2 196 182
453 6.47 1.27 1.0000
9 -13 -2 312 160
17 -6.91 -9.58 1.0000
7 -13 -2 520 185
754 5.36 -4.21 1.0000
5 -13 -2 537 158
522 -5.37 10.28 1.0000
3 -13 -2 3144 269
2911 -4.61 4.67 1.0000
1 -13 -2 457 171
426 10.64 6.34 1.0000
3 13 2 2295 231
1478 5.55 6.51 1.0000
5 13 2 602 228
1371 21.54 -3.04 1.0000
7 13 2 1414 224
1028 -1.25 -7.01 1.0000
9 13 2 2418 356
3706 7.62 -10.43 1.0000
11 13 2 2126 313
1922 1.69 2.13 1.0000
-13 -13 -2 2118 264
5008 -0.97 22.06 1.0000
13 13 2 1955 415
4754 -0.97 -22.06 1.0000
-15 -13 -2 236 130
162 0.70 -5.21 1.0000
15 13 2 57 201
11 0.70 5.21 1.0000
-17 -13 -2 182 151
341 0.24 5.09 1.0000
17 13 2 15 257
6 0.24 -5.09 1.0000
19 13 2 44 305

```
43      3.73    -5.16  1.0000
  21  13    2       241       346
329      2.29    -0.01  1.0000
  23  13    2        11       365
-18    -0.35    -2.83  1.0000
  22 -14   -2       218       281
195    -2.82     0.44  1.0000
  22 -14   -2       207       305
184    -2.82     0.44  1.0000
  20 -14   -2        40       241
-145    -1.90     5.16  1.0000
  20 -14   -2       400       254
235    -1.90     5.16  1.0000
  18 -14   -2       541       228
289      8.14    -4.86  1.0000
  16 -14   -2       740       240
789    -6.22     2.79  1.0000
  14 -14   -2       187       188
-39      0.64    11.84  1.0000
  12 -14   -2        98       189
111      6.66    -5.01  1.0000
  10 -14   -2       398       278
1951   -13.89    16.16  1.0000
   8 -14   -2       378       214
1180    11.75     6.02  1.0000
   6 -14   -2       620       264
1965      2.12   -17.81  1.0000
   4 -14   -2       724       175
493    -8.33    -3.24  1.0000
   2 -14   -2       749       221
1288    10.99    -3.19  1.0000
   0 -14   -2        92       182
446   -11.26   -21.47  1.0000
   2  14    2        98       171
532   -10.46    -4.54  1.0000
   4  14    2      1371       234
1285      2.98    -5.82  1.0000
   6  14    2         9       209
831   -20.84    17.93  1.0000
   8  14    2        91       184
-198      4.73    -6.78  1.0000
  10  14    2       861       222
147    -7.03    19.82  1.0000
  12  14    2       274       235
```

427 1.73 -3.35 1.0000
14 14 2 188 245
-42 0.70 11.87 1.0000
-16 -14 -2 9 146
-29 2.53 -0.16 1.0000
16 14 2 9 220
-71 2.53 0.16 1.0000
18 14 2 11 235
-61 -1.22 2.59 1.0000
20 14 2 39 280
-245 -4.63 2.81 1.0000
22 14 2 48 338
46 0.86 0.35 1.0000
21 -15 -2 34 285
63 0.47 -2.30 1.0000
21 -15 -2 114 281
166 0.47 -2.30 1.0000
19 -15 -2 352 257
396 -2.10 0.68 1.0000
17 -15 -2 127 236
361 6.34 2.33 1.0000
15 -15 -2 1008 231
450 -6.51 -11.45 1.0000
13 -15 -2 315 207
237 0.74 5.25 1.0000
11 -15 -2 80 201
538 6.96 -10.38 1.0000
9 -15 -2 1725 222
1018 -2.26 -8.04 1.0000
7 -15 -2 269 187
513 -0.13 15.84 1.0000
5 -15 -2 281 161
153 3.16 -2.26 1.0000
3 -15 -2 122 221
909 1.51 16.73 1.0000
1 -15 -2 207 223
814 1.58 14.56 1.0000
-1 -15 -2 2276 345
2876 9.60 -5.37 1.0000
3 15 2 861 248
1401 10.61 -13.48 1.0000
5 15 2 112 212
430 -0.50 9.20 1.0000
7 15 2 2321 246

1235 16.18 -6.23 1.0000
9 15 2 68 207
-36 -6.38 -3.26 1.0000
11 15 2 107 255
369 5.68 -11.06 1.0000
13 15 2 122 240
347 1.59 7.39 1.0000
15 15 2 637 264
193 -6.54 -11.43 1.0000
17 15 2 21 281
55 3.21 -1.84 1.0000
19 15 2 8 256
-9 -1.36 1.95 1.0000
21 15 2 82 284
21 0.57 -3.91 1.0000
20 -16 -2 3 279
-41 0.18 -1.43 1.0000
18 -16 -2 56 251
99 -4.91 3.03 1.0000
16 -16 -2 13 203
-82 2.99 0.77 1.0000
14 -16 -2 294 216
272 2.56 0.15 1.0000
12 -16 -2 251 247
1067 -9.07 13.56 1.0000
10 -16 -2 32 202
202 8.31 -6.30 1.0000
8 -16 -2 974 212
740 -10.84 -0.79 1.0000
6 -16 -2 804 223
820 1.82 0.74 1.0000
4 -16 -2 284 203
536 9.37 -17.86 1.0000
2 -16 -2 955 188
147 -11.61 -11.42 1.0000
0 -16 -2 521 198
275 3.87 -3.80 1.0000
4 16 2 1066 331
2997 -20.21 9.06 1.0000
6 16 2 138 259
1030 11.61 -18.12 1.0000
8 16 2 106 257
1076 -14.93 16.28 1.0000
10 16 2 819 275

963	3.13	-5.82	1.0000	
12	16	2	467	295
907	6.18	4.23	1.0000	
14	16	2	505	274
530	-6.22	2.71	1.0000	
16	16	2	13	245
-303	3.03	0.76	1.0000	
18	16	2	24	286
-59	-2.24	3.63	1.0000	
20	16	2	84	338
242	5.58	-0.38	1.0000	
19	-17	-2	115	276
126	2.10	1.50	1.0000	
17	-17	-2	295	246
217	-0.48	-2.09	1.0000	
15	-17	-2	183	239
283	3.29	-1.80	1.0000	
13	-17	-2	258	234
441	0.24	-5.03	1.0000	
11	-17	-2	550	234
743	-6.97	-3.37	1.0000	
9	-17	-2	243	254
1054	11.98	8.41	1.0000	
7	-17	-2	252	194
185	-0.19	-4.98	1.0000	
5	-17	-2	2725	390
4657	-4.28	14.35	1.0000	
3	-17	-2	735	276
1564	7.09	10.24	1.0000	
1	-17	-2	2951	343
2605	-2.71	-1.17	1.0000	
-1	-17	-2	531	327
1909	4.67	18.50	1.0000	
5	17	2	500	236
459	5.58	-7.10	1.0000	
7	17	2	44	231
-88	-1.99	5.38	1.0000	
9	17	2	248	232
181	8.22	2.11	1.0000	
11	17	2	1648	363
2323	-7.38	-0.74	1.0000	
13	17	2	313	274
163	-3.44	-2.55	1.0000	
15	17	2	153	290

402 6.29 2.33 1.0000
17 17 2 282 273
207 -0.40 -2.05 1.0000
19 17 2 48 323
123 0.61 -3.66 1.0000
18 -18 -2 354 288
324 -1.23 -0.92 1.0000
16 -18 -2 25 239
-28 -2.24 3.68 1.0000
14 -18 -2 108 227
60 -1.24 2.71 1.0000
12 -18 -2 120 201
174 1.91 -1.02 1.0000
10 -18 -2 79 221
227 -5.47 6.94 1.0000
8 -18 -2 321 235
702 -1.74 -7.24 1.0000
6 -18 -2 316 228
592 -1.46 -7.87 1.0000
4 -18 -2 10 203
-113 -2.71 -0.30 1.0000
2 -18 -2 107 253
718 6.02 -13.45 1.0000
0 -18 -2 7 213
-59 -2.32 0.03 1.0000
-2 -18 -2 259 247
85 -6.13 -4.36 1.0000
4 18 2 16 222
325 6.55 -10.59 1.0000
6 18 2 1466 299
1651 -12.75 6.29 1.0000
8 18 2 935 285
992 2.50 -12.84 1.0000
10 18 2 979 268
786 3.25 0.34 1.0000
12 18 2 788 279
1139 -2.87 4.09 1.0000
14 18 2 295 276
97 8.19 -4.85 1.0000
16 18 2 30 288
44 -4.97 2.95 1.0000
18 18 2 4 328
-188 -1.31 -0.99 1.0000
17 -19 -2 18 261

-228 0.64 -3.63 1.0000
15 -19 -2 139 250
91 -1.41 1.82 1.0000
13 -19 -2 122 226
175 3.83 -5.16 1.0000
11 -19 -2 226 211
123 4.14 0.15 1.0000
9 -19 -2 260 226
422 -8.19 -1.00 1.0000
7 -19 -2 110 206
-78 6.69 6.16 1.0000
5 -19 -2 488 221
226 3.50 5.10 1.0000
3 -19 -2 685 272
740 -7.37 0.81 1.0000
1 -19 -2 409 237
62 6.02 10.61 1.0000
-1 -19 -2 164 257
392 -1.21 -10.12 1.0000
5 19 2 75 203
-96 2.37 7.15 1.0000
7 19 2 514 280
865 0.56 5.87 1.0000
9 19 2 205 252
135 -1.98 1.37 1.0000
11 19 2 665 267
487 1.08 4.73 1.0000
13 19 2 181 262
209 -0.84 -3.26 1.0000
15 19 2 6 293
-68 -2.15 0.49 1.0000
17 19 2 270 335
296 2.03 1.60 1.0000
16 -20 -2 49 275
181 5.61 -0.42 1.0000
14 -20 -2 40 237
-127 -4.63 2.88 1.0000
12 -20 -2 362 252
320 -4.27 2.22 1.0000
10 -20 -2 72 237
87 -1.03 -1.58 1.0000
8 -20 -2 581 242
356 -0.15 4.90 1.0000
6 -20 -2 292 242

553 -3.10 -4.93 1.0000
4 -20 -2 102 220
53 -0.86 -9.56 1.0000
2 -20 -2 666 245
361 1.30 5.76 1.0000
0 -20 -2 554 303
1075 -1.27 -7.92 1.0000
-2 -20 -2 118 277
358 3.17 6.31 1.0000
6 20 2 54 238
4 1.78 -7.50 1.0000
8 20 2 30 244
-199 2.01 -4.30 1.0000
10 20 2 340 270
371 -1.58 -1.47 1.0000
12 20 2 100 266
212 2.05 -3.93 1.0000
14 20 2 729 296
502 -1.93 5.25 1.0000
16 20 2 15 301
28 0.15 -1.42 1.0000
15 -21 -2 20 282
-11 0.54 -3.83 1.0000
13 -21 -2 31 258
67 2.32 0.00 1.0000
11 -21 -2 185 252
240 1.54 -0.67 1.0000
9 -21 -2 408 275
790 4.71 -4.98 1.0000
7 -21 -2 103 231
263 -7.33 3.57 1.0000
5 -21 -2 309 251
352 0.26 1.76 1.0000
3 -21 -2 15 240
-161 3.02 1.36 1.0000
1 -21 -2 386 274
588 -4.12 -2.13 1.0000
-1 -21 -2 268 257
244 0.61 0.21 1.0000
-3 -21 -2 745 317
487 1.66 -4.42 1.0000
7 21 2 6 243
-138 -0.28 2.10 1.0000
9 21 2 144 288

255 -2.86 4.63 1.0000
11 21 2 25 303
30 -0.20 0.35 1.0000
13 21 2 114 288
245 4.27 2.40 1.0000
15 21 2 190 297
255 0.49 -2.27 1.0000
14 -22 -2 229 303
220 0.83 0.13 1.0000
12 -22 -2 103 269
204 -0.63 3.73 1.0000
10 -22 -2 58 238
-266 -5.21 4.06 1.0000
8 -22 -2 402 263
528 -3.50 -2.03 1.0000
6 -22 -2 422 266
487 1.79 1.37 1.0000
4 -22 -2 1 262
-31 0.56 0.77 1.0000
2 -22 -2 56 262
201 -1.02 -6.78 1.0000
0 -22 -2 61 263
-66 2.87 6.12 1.0000
-2 -22 -2 96 265
115 -0.20 -0.92 1.0000
-4 -22 -2 27 316
94 -2.00 3.38 1.0000
8 22 2 11 262
5 0.60 -0.69 1.0000
10 22 2 133 302
193 1.26 -2.51 1.0000
12 22 2 475 341
423 0.48 -1.36 1.0000
14 22 2 493 324
463 -2.89 0.45 1.0000
13 -23 -2 305 302
427 -0.41 -2.78 1.0000
11 -23 -2 212 300
365 0.17 -4.53 1.0000
9 -23 -2 215 272
282 2.89 2.26 1.0000
7 -23 -2 294 255
168 1.61 -4.05 1.0000
5 -23 -2 320 278

412 -4.47 -0.80 1.0000
3 -23 -2 153 275
328 -1.18 4.83 1.0000
1 -23 -2 53 276
-28 -0.98 -6.22 1.0000
-1 -23 -2 42 288
31 -0.42 -1.00 1.0000
-3 -23 -2 161 296
125 -0.39 -3.44 1.0000
-5 -23 -2 408 359
548 0.88 -2.64 1.0000
7 23 2 377 298
628 -2.94 4.00 1.0000
9 23 2 2 273
-115 0.65 1.01 1.0000
11 23 2 93 318
129 1.94 1.15 1.0000
13 23 2 24 328
6 -2.11 0.46 1.0000
10 -24 -2 10 284
-33 -2.29 1.38 1.0000
8 -24 -2 29 267
21 -1.73 4.02 1.0000
6 -24 -2 209 286
202 -2.48 0.43 1.0000
4 -24 -2 218 292
334 -0.32 -3.38 1.0000
2 -24 -2 70 292
110 2.38 1.55 1.0000
0 -24 -2 314 313
276 0.53 -0.92 1.0000
-2 -24 -2 84 312
132 -0.05 2.05 1.0000
-4 -24 -2 23 331
121 4.94 2.46 1.0000
-6 -24 -2 355 373
495 -2.70 1.27 1.0000
8 24 2 381 317
435 -0.07 -2.30 1.0000
10 24 2 24 310
45 0.70 -2.74 1.0000
9 -25 -2 21 272
-115 -2.98 -2.57 1.0000
7 -25 -2 151 300

141 0.75 -1.15 1.0000
5 -25 -2 144 293
160 1.60 1.13 1.0000
3 -25 -2 82 303
56 -3.22 -3.17 1.0000
1 -25 -2 15 296
-52 1.66 2.93 1.0000
-1 -25 -2 344 320
286 -0.37 -2.77 1.0000
-3 -25 -2 245 348
353 -2.56 -3.87 1.0000
-5 -25 -2 1 349
-209 -0.76 -0.08 1.0000
9 25 2 350 317
424 0.56 1.85 1.0000
4 -26 -2 215 321
209 0.46 1.37 1.0000
2 -26 -2 116 329
183 -1.44 -2.21 1.0000
0 -26 -2 143 336
109 -0.37 -1.61 1.0000
-2 -26 -2 16 365
-139 3.32 1.18 1.0000
-4 -26 -2 180 388
157 -0.77 2.21 1.0000
-3 -26 3 4 354
-90 1.32 1.05 1.0000
-1 -26 3 396 355
572 2.57 2.36 1.0000
1 -26 3 124 319
216 -4.34 -2.68 1.0000
3 -26 3 141 323
223 2.06 1.67 1.0000
8 25 -3 236 363
199 -1.51 -0.57 1.0000
-8 -25 3 71 396
51 -1.51 0.57 1.0000
6 25 -3 257 329
235 -0.42 -0.98 1.0000
-6 -25 3 30 351
22 -0.42 0.98 1.0000
4 25 -3 84 306
72 -0.87 0.40 1.0000
-4 -25 3 1 336

-148 -0.87 -0.40 1.0000
-2 -25 3 85 327
33 -2.73 -1.43 1.0000
0 -25 3 73 321
125 2.64 0.16 1.0000
2 -25 3 57 323
106 0.18 2.56 1.0000
4 -25 3 150 304
168 -0.39 1.40 1.0000
6 -25 3 2 307
-113 -1.05 0.84 1.0000
8 -25 3 133 299
216 -2.71 -0.58 1.0000
9 24 -3 348 374
311 0.87 1.34 1.0000
7 24 -3 12 299
-142 2.78 -1.16 1.0000
-7 -24 3 449 373
586 2.78 1.16 1.0000
5 24 -3 39 276
139 4.78 1.88 1.0000
-5 -24 3 31 325
125 4.78 -1.88 1.0000
3 24 -3 277 257
385 2.77 -0.42 1.0000
-3 -24 3 10 305
-167 2.77 0.42 1.0000
1 24 -3 78 241
167 -1.00 -3.70 1.0000
-1 -24 3 90 309
184 -1.00 3.70 1.0000
1 -24 3 106 285
167 3.38 0.40 1.0000
3 -24 3 22 276
-87 -3.84 -1.44 1.0000
5 -24 3 183 284
133 -2.49 1.00 1.0000
7 -24 3 112 288
154 4.05 -2.55 1.0000
9 -24 3 1 280
-151 -0.06 -0.92 1.0000
12 23 -3 233 421
254 0.02 -3.85 1.0000
12 23 -3 20 331

-91 0.02 -3.85 1.0000
10 23 -3 344 292
396 0.11 1.47 1.0000
10 23 -3 685 374
758 0.11 1.47 1.0000
8 23 -3 842 323
906 -0.33 0.89 1.0000
6 23 -3 156 273
68 -6.57 0.47 1.0000
-6 -23 3 58 351
-28 -6.57 -0.47 1.0000
4 23 -3 17 221
-3 -3.52 -0.16 1.0000
-4 -23 3 17 295
-254 -3.52 0.16 1.0000
2 23 -3 23 205
-69 1.52 -3.83 1.0000
-2 -23 3 23 295
-322 1.52 3.83 1.0000
0 23 -3 11 187
59 -3.61 1.91 1.0000
0 -23 3 176 288
317 -3.61 -1.91 1.0000
2 -23 3 125 281
199 0.56 2.68 1.0000
4 -23 3 100 275
204 3.65 -1.06 1.0000
6 -23 3 97 254
164 -1.76 1.92 1.0000
8 -23 3 12 246
-181 -2.63 1.35 1.0000
10 -23 3 106 270
82 -1.26 -1.20 1.0000
12 -23 3 219 294
334 -2.96 0.73 1.0000
13 22 -3 1169 376
1199 -0.05 -1.17 1.0000
13 22 -3 1512 429
1546 -0.05 -1.17 1.0000
11 22 -3 630 351
705 1.39 4.15 1.0000
11 22 -3 375 319
428 1.39 4.15 1.0000
9 22 -3 504 306

589	2.28	-3.54	1.0000	
9	22	-3	107	267
136	2.28	-3.54	1.0000	
7	22	-3	41	245
-163	5.52	-0.17	1.0000	
5	22	-3	314	248
476	4.82	0.40	1.0000	
-5	-22	3	418	345
606	4.82	-0.40	1.0000	
3	22	-3	71	204
166	3.72	-2.99	1.0000	
-3	-22	3	211	308
367	3.72	2.99	1.0000	
1	22	-3	237	209
519	1.39	6.92	1.0000	
-1	-22	3	143	282
373	1.39	-6.92	1.0000	
-1	22	-3	191	196
426	0.93	-5.95	1.0000	
1	-22	3	271	288
543	0.93	5.95	1.0000	
3	-22	3	90	270
101	-1.77	-0.16	1.0000	
5	-22	3	206	266
328	-2.95	-3.80	1.0000	
7	-22	3	348	267
447	-3.79	3.89	1.0000	
9	-22	3	781	299
851	1.70	-1.23	1.0000	
11	-22	3	146	282
389	3.49	-6.09	1.0000	
13	-22	3	48	272
-143	0.18	6.03	1.0000	
14	21	-3	211	383
310	-0.88	-5.50	1.0000	
14	21	-3	207	308
305	-0.88	-5.50	1.0000	
12	21	-3	376	313
522	2.34	2.23	1.0000	
12	21	-3	522	353
692	2.34	2.23	1.0000	
10	21	-3	354	298
458	-2.96	-0.74	1.0000	
10	21	-3	528	307

```
654    -2.96   -0.74  1.0000
   8  21  -3      504      271
900    -6.50   -0.90  1.0000
   8  21  -3      123      266
348    -6.50   -0.90  1.0000
   6  21  -3       33      216
-34    -2.14    4.48  1.0000
   4  21  -3      855      259
1295    -4.66   -3.78  1.0000
  -4 -21   3      979      338
1446    -4.66    3.78  1.0000
   2  21  -3      139      197
234    -4.21    2.74  1.0000
  -2 -21   3      132      270
230    -4.21   -2.74  1.0000
   0  21  -3       45      186
181     2.79   -5.28  1.0000
   0 -21   3       16      269
9     2.79    5.28  1.0000
  -2  21  -3      316      178
270    -0.07    1.17  1.0000
   2 -21   3      256      266
215    -0.07   -1.17  1.0000
   4 -21   3      819      277
814     0.61    4.87  1.0000
   6 -21   3      399      240
243     4.61   -1.97  1.0000
   8 -21   3      380      253
426    -0.18   -4.86  1.0000
  10 -21   3       99      237
-127    -3.27    8.00  1.0000
  12 -21   3      140      266
346    -2.60   -5.26  1.0000
  14 -21   3        1      249
-236    -0.47    0.72  1.0000
  17  20  -3      364      336
403     0.08    1.03  1.0000
  17  20  -3      606      456
657     0.08    1.03  1.0000
  15  20  -3      131      300
298    -4.38    2.92  1.0000
  15  20  -3      842      408
1217    -4.38    2.92  1.0000
  13  20  -3      593      292
```

621	1.77	-0.55	1.0000	
13	20	-3	405	345
428	1.77	-0.55	1.0000	
11	20	-3	244	299
269	1.40	-1.95	1.0000	
11	20	-3	8	279
-76	1.40	-1.95	1.0000	
9	20	-3	308	242
315	4.24	0.77	1.0000	
9	20	-3	262	274
266	4.24	0.77	1.0000	
7	20	-3	140	234
123	6.65	1.90	1.0000	
7	20	-3	64	225
-42	6.65	1.90	1.0000	
5	20	-3	327	197
165	3.51	-2.89	1.0000	
3	20	-3	922	235
1100	0.86	5.21	1.0000	
-3	-20	3	853	316
1025	0.86	-5.21	1.0000	
1	20	-3	798	184
289	5.77	-8.09	1.0000	
-1	-20	3	428	261
88	5.77	8.09	1.0000	
-1	20	-3	213	167
62	-6.64	6.63	1.0000	
1	-20	3	587	260
388	-6.64	-6.63	1.0000	
-3	20	-3	473	201
569	-1.93	0.21	1.0000	
3	-20	3	305	255
383	-1.93	-0.21	1.0000	
5	-20	3	2247	362
2425	-1.46	3.10	1.0000	
7	-20	3	108	227
105	-8.27	-3.88	1.0000	
9	-20	3	595	267
606	-0.13	0.50	1.0000	
11	-20	3	41	228
52	-0.12	4.82	1.0000	
13	-20	3	54	239
-226	3.74	-5.13	1.0000	
15	-20	3	24	250

45 -0.14 1.64 1.0000
17 -20 3 125 284
376 3.92 5.95 1.0000
18 19 -3 68 333
182 1.35 4.66 1.0000
18 19 -3 1014 464
1384 1.35 4.66 1.0000
16 19 -3 529 308
609 -0.02 -5.38 1.0000
16 19 -3 1068 425
1196 -0.02 -5.38 1.0000
14 19 -3 129 272
257 4.04 -0.20 1.0000
14 19 -3 247 348
416 4.04 -0.20 1.0000
12 19 -3 11 260
-194 -2.20 1.92 1.0000
12 19 -3 374 304
274 -2.20 1.92 1.0000
10 19 -3 107 261
164 -2.79 -1.31 1.0000
10 19 -3 13 226
-88 -2.79 -1.31 1.0000
8 19 -3 68 236
-326 -4.87 5.24 1.0000
8 19 -3 68 195
-224 -4.87 5.24 1.0000
6 19 -3 811 252
880 -5.93 -8.35 1.0000
6 19 -3 956 220
1043 -5.93 -8.35 1.0000
4 19 -3 1023 206
740 -0.23 7.13 1.0000
-4 -19 3 885 310
619 -0.23 -7.13 1.0000
2 19 -3 21 162
-11 -3.15 -2.46 1.0000
-2 -19 3 70 252
32 -3.15 2.46 1.0000
0 19 -3 225 170
246 1.09 -2.29 1.0000
0 -19 3 65 258
73 1.09 2.29 1.0000
-2 19 -3 175 180

```
404      9.37      1.44  1.0000
   2 -19    3        80       248
216      9.37    -1.44  1.0000
  -4  19   -3       872       189
705      2.34    -1.16  1.0000
   4 -19    3       791       254
632      2.34     1.16  1.0000
   6 -19    3      1044       258
799      2.83     2.08  1.0000
   8 -19    3        22       210
10     5.59     2.65  1.0000
  10 -19    3        80       234
253    -3.36    -8.97  1.0000
  12 -19    3       206       232
275      0.04     2.92  1.0000
  14 -19    3        18       233
44    -4.10     1.68  1.0000
  16 -19    3        49       250
128    -2.59    -2.69  1.0000
  18 -19    3         8       277
-14      0.04     2.45  1.0000
  19  18   -3       607       453
704      0.04     2.35  1.0000
  19  18   -3         7       352
-54      0.04     2.35  1.0000
  17  18   -3       861       416
1079     -2.60     1.72  1.0000
  17  18   -3       451       345
612    -2.60     1.72  1.0000
  15  18   -3       615       355
622      2.80    -1.76  1.0000
  15  18   -3       161       284
157      2.80    -1.76  1.0000
  13  18   -3        95       234
-247     -5.31     6.59  1.0000
  13  18   -3        63       305
310    -5.31     6.59  1.0000
  11  18   -3       335       276
619      1.39    -8.43  1.0000
  11  18   -3       300       261
568      1.39    -8.43  1.0000
   9  18   -3       110       227
464      8.94     3.54  1.0000
   9  18   -3         5       249
```

172 8.94 3.54 1.0000
7 18 -3 500 199
394 -2.26 0.04 1.0000
7 18 -3 395 233
302 -2.26 0.04 1.0000
5 18 -3 289 177
380 1.59 1.46 1.0000
3 18 -3 1263 261
1383 6.62 -1.16 1.0000
-3 -18 3 1381 319
1509 6.62 1.16 1.0000
1 18 -3 172 146
-4 -10.09 5.25 1.0000
-1 -18 3 172 232
-77 -10.09 -5.25 1.0000
-1 18 -3 1078 204
1142 1.30 -4.14 1.0000
1 -18 3 877 265
937 1.30 4.14 1.0000
-3 18 -3 410 150
106 -8.57 -1.23 1.0000
3 -18 3 278 213
45 -8.57 1.23 1.0000
5 -18 3 1064 284
1479 -10.54 -4.14 1.0000
7 -18 3 367 228
513 2.52 2.21 1.0000
9 -18 3 195 245
523 -7.59 -2.32 1.0000
11 -18 3 510 223
446 0.22 1.65 1.0000
13 -18 3 54 216
-201 3.31 5.47 1.0000
15 -18 3 352 231
216 2.62 -3.27 1.0000
17 -18 3 15 258
41 0.60 -3.02 1.0000
19 -18 3 62 290
171 1.36 4.65 1.0000
20 17 -3 511 484
946 3.85 5.94 1.0000
20 17 -3 126 343
376 3.85 5.94 1.0000
18 17 -3 501 423

644 0.74 -2.99 1.0000
18 17 -3 112 340
182 0.74 -2.99 1.0000
16 17 -3 218 365
233 -1.50 -2.98 1.0000
16 17 -3 29 295
24 -1.50 -2.98 1.0000
14 17 -3 1268 336
1279 3.99 4.78 1.0000
14 17 -3 493 283
480 3.99 4.78 1.0000
12 17 -3 65 236
-39 0.87 -6.96 1.0000
12 17 -3 5 260
55 0.87 -6.96 1.0000
10 17 -3 34 228
206 -4.43 7.09 1.0000
10 17 -3 58 222
266 -4.43 7.09 1.0000
8 17 -3 54 226
-190 0.04 -6.39 1.0000
8 17 -3 295 198
210 0.04 -6.39 1.0000
6 17 -3 1184 284
1754 -0.83 7.97 1.0000
6 17 -3 1252 284
1837 -0.83 7.97 1.0000
4 17 -3 446 167
471 -6.35 -3.92 1.0000
2 17 -3 1183 237
1174 8.93 -3.46 1.0000
-2 -17 3 1031 270
1014 8.93 3.46 1.0000
0 17 -3 464 178
735 4.61 6.24 1.0000
0 -17 3 369 249
607 4.61 -6.24 1.0000
-2 17 -3 1385 244
1595 5.79 -7.18 1.0000
2 -17 3 1574 320
1817 5.79 7.18 1.0000
-4 17 -3 1339 284
2908 17.73 4.66 1.0000
4 -17 3 1067 333

2481	17.73	-4.66	1.0000	
6	-17	3	1492	267
1533	3.24	-1.24	1.0000	
8	-17	3	766	243
833	-0.90	4.80	1.0000	
10	-17	3	456	214
194	6.15	-2.63	1.0000	
12	-17	3	334	210
209	-8.88	-2.65	1.0000	
14	-17	3	694	255
600	-1.65	-1.09	1.0000	
16	-17	3	147	232
151	-1.12	2.50	1.0000	
18	-17	3	135	285
227	-2.51	1.74	1.0000	
20	-17	3	57	271
74	0.04	1.09	1.0000	
19	16	-3	961	433
1244	-2.59	-2.64	1.0000	
19	16	-3	45	311
120	-2.59	-2.64	1.0000	
17	16	-3	10	287
-118	-1.07	2.47	1.0000	
17	16	-3	414	368
426	-1.07	2.47	1.0000	
15	16	-3	95	274
121	-3.42	2.85	1.0000	
15	16	-3	338	313
408	-3.42	2.85	1.0000	
13	16	-3	238	276
492	-0.48	-10.43	1.0000	
13	16	-3	147	263
328	-0.48	-10.43	1.0000	
11	16	-3	1885	343
2413	-1.05	14.22	1.0000	
11	16	-3	1540	328
1996	-1.05	14.22	1.0000	
9	16	-3	561	242
1295	-8.53	-7.15	1.0000	
9	16	-3	533	257
1252	-8.53	-7.15	1.0000	
7	16	-3	339	234
568	5.14	-1.14	1.0000	
7	16	-3	110	175

249 5.14 -1.14 1.0000
5 16 -3 1805 275
2283 1.69 7.37 1.0000
5 16 -3 1708 298
2173 1.69 7.37 1.0000
3 16 -3 1539 272
2745 -9.64 -6.12 1.0000
1 16 -3 554 151
416 3.77 6.86 1.0000
-1 -16 3 1086 239
920 3.77 -6.86 1.0000
-1 16 -3 246 127
-46 -13.45 1.91 1.0000
1 -16 3 564 205
214 -13.45 -1.91 1.0000
-3 16 -3 354 135
47 -10.28 -4.31 1.0000
3 -16 3 165 186
-222 -10.28 4.31 1.0000
-5 16 -3 2183 230
1544 -6.55 1.27 1.0000
5 -16 3 1898 252
1305 -6.55 -1.27 1.0000
7 -16 3 3094 310
2377 -7.90 -2.19 1.0000
9 -16 3 258 205
328 3.96 3.49 1.0000
11 -16 3 709 215
700 2.82 -0.26 1.0000
13 -16 3 928 228
675 -2.32 -4.43 1.0000
15 -16 3 109 235
541 7.89 8.11 1.0000
17 -16 3 284 249
299 -1.45 -2.94 1.0000
19 -16 3 338 275
395 -0.08 -5.24 1.0000
20 15 -3 739 433
842 -0.08 1.62 1.0000
20 15 -3 115 335
158 -0.08 1.62 1.0000
18 15 -3 1205 381
951 2.56 -3.39 1.0000
18 15 -3 179 293

86	2.56	-3.39	1.0000	
16	15	-3	335	331
970	7.94	8.04	1.0000	
16	15	-3	63	285
427	7.94	8.04	1.0000	
14	15	-3	146	280
377	-6.02	-5.97	1.0000	
14	15	-3	244	282
540	-6.02	-5.97	1.0000	
12	15	-3	70	242
-1	4.14	5.95	1.0000	
12	15	-3	27	238
180	4.14	5.95	1.0000	
10	15	-3	1549	252
1133	12.86	-6.15	1.0000	
10	15	-3	963	222
588	12.86	-6.15	1.0000	
8	15	-3	308	170
249	-1.29	2.13	1.0000	
8	15	-3	8	187
-68	-1.29	2.13	1.0000	
6	15	-3	456	202
476	0.21	0.58	1.0000	
6	15	-3	776	180
802	0.21	0.58	1.0000	
4	15	-3	6190	346
5331	5.12	-0.73	1.0000	
4	15	-3	5973	374
5129	5.12	-0.73	1.0000	
2	15	-3	976	161
812	-3.92	3.64	1.0000	
-2	-15	3	991	250
829	-3.92	-3.64	1.0000	
0	15	-3	801	199
1116	15.09	-10.63	1.0000	
0	-15	3	958	252
1361	15.09	10.63	1.0000	
-2	15	-3	466	169
1026	12.89	9.35	1.0000	
2	-15	3	393	224
897	12.89	-9.35	1.0000	
-4	15	-3	517	198
1160	8.08	-7.23	1.0000	
4	-15	3	509	228

1146 8.08 7.23 1.0000
-6 15 -3 21 131
253 17.31 -2.34 1.0000
6 -15 3 75 178
134 17.31 2.34 1.0000
8 -15 3 1247 291
2054 -3.45 -10.99 1.0000
10 -15 3 1611 297
2086 -9.86 8.83 1.0000
12 -15 3 1238 265
1422 2.75 -1.09 1.0000
14 -15 3 6 200
118 -7.41 -2.94 1.0000
16 -15 3 77 219
96 -3.37 2.90 1.0000
18 -15 3 167 227
165 2.69 -1.77 1.0000
20 -15 3 137 291
309 -4.44 2.90 1.0000
21 14 -3 128 307
112 -0.49 0.67 1.0000
21 14 -3 1028 449
982 -0.49 0.67 1.0000
19 14 -3 472 378
645 -4.16 1.61 1.0000
19 14 -3 52 289
107 -4.16 1.61 1.0000
17 14 -3 1194 347
1069 -1.67 -1.11 1.0000
17 14 -3 256 289
199 -1.67 -1.11 1.0000
15 14 -3 24 259
183 -7.35 -3.01 1.0000
15 14 -3 84 270
-62 -7.35 -3.01 1.0000
13 14 -3 1601 330
1758 0.10 6.29 1.0000
13 14 -3 1432 308
1578 0.10 6.29 1.0000
11 14 -3 442 235
1155 -9.47 -11.31 1.0000
11 14 -3 433 269
1140 -9.47 -11.31 1.0000
9 14 -3 171 184

289 -12.07 9.59 1.0000
9 14 -3 300 210
621 -12.07 9.59 1.0000
7 14 -3 1567 230
1118 -4.12 4.16 1.0000
7 14 -3 1268 187
867 -4.12 4.16 1.0000
5 14 -3 1539 154
541 -3.11 -13.52 1.0000
5 14 -3 1272 197
388 -3.11 -13.52 1.0000
3 14 -3 3130 213
1255 7.13 17.54 1.0000
1 14 -3 37 128
212 -10.42 -9.35 1.0000
-1 -14 3 42 165
177 -10.42 9.35 1.0000
-1 14 -3 152 119
169 -4.50 -3.24 1.0000
1 -14 3 292 175
331 -4.50 3.24 1.0000
-3 14 -3 1695 237
2901 -10.57 18.62 1.0000
3 -14 3 1414 175
2527 -10.57 -18.62 1.0000
3 -14 3 1749 278
3023 -10.57 -18.62 1.0000
-5 14 -3 1090 228
2444 -9.59 -11.73 1.0000
5 -14 3 934 278
2200 -9.59 11.73 1.0000
-7 14 -3 372 131
313 -3.64 0.94 1.0000
7 -14 3 232 174
182 -3.64 -0.94 1.0000
9 -14 3 794 199
466 9.90 -4.90 1.0000
11 -14 3 917 220
822 3.64 3.12 1.0000
13 -14 3 534 247
1241 10.85 0.87 1.0000
15 -14 3 230 226
517 -6.08 -5.93 1.0000
17 -14 3 659 249

658 3.96 4.89 1.0000
19 -14 3 31 241
107 4.13 -0.07 1.0000
21 -14 3 123 291
191 -0.91 -5.44 1.0000
22 13 -3 1164 455
1676 0.08 6.02 1.0000
22 13 -3 48 344
-80 0.08 6.02 1.0000
20 13 -3 705 399
983 3.76 -5.12 1.0000
20 13 -3 165 305
292 3.76 -5.12 1.0000
18 13 -3 56 251
-44 3.46 5.46 1.0000
18 13 -3 1141 352
968 3.46 5.46 1.0000
16 13 -3 1838 317
1479 -2.30 -4.47 1.0000
16 13 -3 1045 266
776 -2.30 -4.47 1.0000
14 13 -3 1074 345
2021 10.90 1.00 1.0000
14 13 -3 1009 319
1932 10.90 1.00 1.0000
12 13 -3 626 245
850 5.59 1.90 1.0000
12 13 -3 388 220
561 5.59 1.90 1.0000
10 13 -3 182 184
491 7.28 -1.90 1.0000
10 13 -3 173 219
476 7.28 -1.90 1.0000
8 13 -3 2083 316
4118 16.57 1.53 1.0000
8 13 -3 2284 340
4399 16.57 1.53 1.0000
6 13 -3 1307 217
1303 -0.78 -5.18 1.0000
6 13 -3 1760 241
1761 -0.78 -5.18 1.0000
4 13 -3 3114 261
3461 -2.61 0.60 1.0000
4 13 -3 2911 280

3247	-2.61	0.60	1.0000	
2	13	-3	4936	330
5654	3.86	-3.68	1.0000	
0	13	-3	422	132
637	-5.55	8.83	1.0000	
0	-13	3	424	185
637	-5.55	-8.83	1.0000	
-2	13	-3	2537	133
632	16.24	-15.28	1.0000	
2	-13	3	3241	192
1011	16.24	15.28	1.0000	
-4	13	-3	1521	173
1378	12.14	3.51	1.0000	
4	-13	3	1425	214
1289	12.14	-3.51	1.0000	
-6	13	-3	2407	207
2357	-3.53	5.59	1.0000	
6	-13	3	2165	253
2119	-3.53	-5.59	1.0000	
8	-13	3	556	162
246	7.16	14.45	1.0000	
10	-13	3	3397	331
4041	-12.84	-12.86	1.0000	
12	-13	3	1484	248
1298	-14.50	5.31	1.0000	
14	-13	3	1832	303
2012	0.17	6.33	1.0000	
16	-13	3	221	230
458	-0.46	-10.33	1.0000	
18	-13	3	24	236
211	-5.33	6.56	1.0000	
20	-13	3	34	251
37	1.70	-0.55	1.0000	
22	-13	3	431	307
446	-0.10	-1.17	1.0000	
21	12	-3	773	426
1195	-2.54	-5.29	1.0000	
21	12	-3	22	347
132	-2.54	-5.29	1.0000	
19	12	-3	870	363
1020	0.00	3.03	1.0000	
19	12	-3	69	289
110	0.00	3.03	1.0000	
17	12	-3	404	287

271	-8.82	-2.78	1.0000	
17	12	-3	114	246
-10	-8.82	-2.78	1.0000	
15	12	-3	1645	310
1875	2.76	-1.29	1.0000	
15	12	-3	994	264
1173	2.76	-1.29	1.0000	
13	12	-3	756	247
520	-14.42	5.23	1.0000	
13	12	-3	967	233
747	-14.42	5.23	1.0000	
11	12	-3	918	229
1173	-16.98	1.46	1.0000	
11	12	-3	627	199
755	-16.98	1.46	1.0000	
9	12	-3	2176	275
2807	2.28	-6.05	1.0000	
9	12	-3	2316	314
2966	2.28	-6.05	1.0000	
7	12	-3	2265	162
956	-17.12	1.22	1.0000	
7	12	-3	2149	185
878	-17.12	1.22	1.0000	
5	12	-3	532	178
578	7.09	8.75	1.0000	
5	12	-3	651	140
723	7.09	8.75	1.0000	
3	12	-3	1471	232
2340	2.15	-8.57	1.0000	
3	12	-3	1189	204
1980	2.15	-8.57	1.0000	
1	12	-3	864	184
1595	9.15	1.24	1.0000	
-1	-12	3	671	231
1325	9.15	-1.24	1.0000	
-1	12	-3	2412	245
3735	-0.40	11.88	1.0000	
1	-12	3	2185	285
3448	-0.40	-11.88	1.0000	
-3	12	-3	200	103
271	-6.78	-16.64	1.0000	
3	-12	3	214	88
226	-6.78	16.64	1.0000	
3	-12	3	223	138

314	-6.78	16.64	1.0000	
-5	12	-3	1951	246
3802	-6.46	14.36	1.0000	
5	-12	3	1872	281
3691	-6.46	-14.36	1.0000	
-7	12	-3	587	108
296	12.07	1.26	1.0000	
7	-12	3	524	144
242	12.07	-1.26	1.0000	
9	-12	3	1224	232
1413	-3.23	8.20	1.0000	
11	-12	3	1127	155
38	20.94	-11.49	1.0000	
13	-12	3	729	222
973	5.63	1.93	1.0000	
15	-12	3	3	198
47	4.03	5.93	1.0000	
17	-12	3	66	209
240	0.75	-6.93	1.0000	
19	-12	3	11	236
-83	-2.13	1.94	1.0000	
21	-12	3	150	284
246	2.31	2.30	1.0000	
23	-12	3	18	295
-131	0.00	-3.70	1.0000	
22	11	-3	352	375
704	3.63	-6.04	1.0000	
22	11	-3	1636	428
2330	3.63	-6.04	1.0000	
20	11	-3	30	308
-444	-0.05	4.72	1.0000	
20	11	-3	781	359
915	-0.05	4.72	1.0000	
18	11	-3	512	302
454	0.19	1.47	1.0000	
16	11	-3	478	281
460	2.69	-0.07	1.0000	
16	11	-3	484	269
466	2.69	-0.07	1.0000	
14	11	-3	939	251
833	3.81	3.08	1.0000	
14	11	-3	1244	256
1126	3.81	3.08	1.0000	
12	11	-3	923	170

8	21.09	-11.30	1.0000	
12	11	-3	1260	185
64	21.09	-11.30	1.0000	
10	11	-3	439	157
406	6.63	5.37	1.0000	
10	11	-3	680	209
665	6.63	5.37	1.0000	
8	11	-3	164	136
342	4.14	-2.76	1.0000	
8	11	-3	123	174
281	4.14	-2.76	1.0000	
6	11	-3	1620	213
2034	6.20	-0.72	1.0000	
6	11	-3	1666	225
2086	6.20	-0.72	1.0000	
4	11	-3	1533	246
3379	-16.38	-3.62	1.0000	
4	11	-3	1821	272
3802	-16.38	-3.62	1.0000	
2	11	-3	6231	317
5708	-11.08	-8.36	1.0000	
0	11	-3	1223	177
1723	-5.27	-2.92	1.0000	
0	-11	3	878	208
1311	-5.27	2.92	1.0000	
-2	11	-3	281	126
787	-11.56	0.15	1.0000	
2	-11	3	303	97
825	-11.56	-0.15	1.0000	
2	-11	3	381	168
956	-11.56	-0.15	1.0000	
-4	11	-3	1577	171
1894	7.49	-0.37	1.0000	
4	-11	3	1613	205
1936	7.49	0.37	1.0000	
4	-11	3	1079	129
1334	7.49	0.37	1.0000	
-6	11	-3	269	92
97	2.45	-5.38	1.0000	
6	-11	3	344	129
145	2.45	5.38	1.0000	
8	-11	3	929	227
2255	-17.25	-10.00	1.0000	
10	-11	3	6128	448

```
7711     0.15    10.95  1.0000
  12 -11    3      688      197
848  -16.98     1.41  1.0000
  14 -11    3      700      283
1588    -9.50   -11.33  1.0000
  16 -11    3     1642      316
2119    -1.03    14.24  1.0000
  18 -11    3      279      243
535     1.37    -8.40  1.0000
  20 -11    3        7      236
-309     1.41    -1.79  1.0000
  22 -11    3      332      282
380     1.39     4.06  1.0000
  23  10   -3     1026      391
956   -1.28    -1.37  1.0000
  19  10   -3       37      293
69   -3.29    -8.95  1.0000
  19  10   -3      690      325
1209    -3.29    -8.95  1.0000
  17  10   -3       62      277
-59     6.31    -2.67  1.0000
  17  10   -3      180      261
33     6.31    -2.67  1.0000
  15  10   -3     1211      319
1600    -9.85     8.72  1.0000
  15  10   -3     1275      320
1679    -9.85     8.72  1.0000
  13  10   -3     3358      331
3995   -12.91   -13.04  1.0000
  13  10   -3     3446      369
4097   -12.91   -13.04  1.0000
  11  10   -3     6073      418
7642     0.24    10.97  1.0000
  11  10   -3     5494      425
6987     0.24    10.97  1.0000
   9  10   -3     1895      272
3579   -25.13     7.30  1.0000
   9  10   -3     1839      292
3492   -25.13     7.30  1.0000
   7  10   -3      718      112
207    12.28   -21.66  1.0000
   7  10   -3      744      152
290    12.28   -21.66  1.0000
   5  10   -3     7676      517
```

10502 6.07 17.04 1.0000
5 10 -3 8305 511
11244 6.07 17.04 1.0000
3 10 -3 10809 502
11175 15.97 -12.78 1.0000
3 10 -3 9962 515
10291 15.97 -12.78 1.0000
1 10 -3 1013 116
490 21.43 8.92 1.0000
-1 -10 3 1118 157
571 21.43 -8.92 1.0000
-1 10 -3 1629 146
997 11.67 -0.82 1.0000
1 -10 3 1587 165
959 11.67 0.82 1.0000
-3 10 -3 402 72
385 -0.06 4.16 1.0000
-3 10 -3 378 98
361 -0.06 4.16 1.0000
3 -10 3 258 128
239 -0.06 -4.16 1.0000
3 -10 3 455 85
437 -0.06 -4.16 1.0000
-5 10 -3 6008 313
7068 9.66 -0.17 1.0000
-5 10 -3 5781 343
6820 9.66 -0.17 1.0000
5 -10 3 5734 374
6782 9.66 0.17 1.0000
-7 10 -3 821 128
1567 -10.10 -3.22 1.0000
-7 10 -3 726 156
1434 -10.10 -3.22 1.0000
7 -10 3 840 200
1589 -10.10 3.22 1.0000
-9 10 -3 308 154
1997 21.81 10.21 1.0000
9 -10 3 293 217
1952 21.81 -10.21 1.0000
11 -10 3 568 173
539 6.76 5.32 1.0000
13 -10 3 162 184
444 6.97 -1.93 1.0000
15 -10 3 814 205

453	12.96	-6.27	1.0000		
17	-10	3	65	213	
284	-4.39	7.17	1.0000		
19	-10	3	53	237	
94	-2.88	-1.39	1.0000		
21	-10	3	267	274	
357	-2.98	-0.71	1.0000		
23	-10	3	127	289	
159	0.08	1.46	1.0000		
20	9	-3	1354	331	
1375	-0.06	0.48	1.0000		
20	9	-3	10	297	
12	-0.06	0.48	1.0000		
18	9	-3	483	297	
958	-7.67	-2.22	1.0000		
18	9	-3	154	278	
455	-7.67	-2.22	1.0000		
16	9	-3	154	260	
207	4.10	3.29	1.0000		
16	9	-3	37	236	
-1	4.10	3.29	1.0000		
14	9	-3	727	201	
404	9.95	-4.79	1.0000		
14	9	-3	849	242	
507	9.95	-4.79	1.0000		
12	9	-3	1291	216	
1481	-3.15	8.34	1.0000		
12	9	-3	1395	280	
1597	-3.15	8.34	1.0000		
10	9	-3	91	225	
1327	21.98	-10.28	1.0000		
10	9	-3	276	227	
1922	21.98	-10.28	1.0000		
8	9	-3	883	169	
799	-1.35	-1.64	1.0000		
8	9	-3	655	140	
582	-1.35	-1.64	1.0000		
6	9	-3	765	164	
666	-16.79	0.75	1.0000		
6	9	-3	897	140	
831	-16.79	0.75	1.0000		
4	9	-3	8514	474	
10407	-8.68	-16.18	1.0000		
4	9	-3	7938	481	

9757 -8.68 -16.18 1.0000
2 9 -3 2116 269
4405 -41.39 16.46 1.0000
2 9 -3 2106 258
4381 -41.39 16.46 1.0000
0 9 -3 11479 648
14936 -24.02 -32.34 1.0000
0 -9 3 11335 671
14787 -24.02 32.34 1.0000
-2 9 -3 2134 94
1282 -7.07 10.99 1.0000
-2 9 -3 1813 141
1027 -7.07 10.99 1.0000
2 -9 3 1857 97
1064 -7.07 -10.99 1.0000
2 -9 3 1815 159
1031 -7.07 -10.99 1.0000
-4 9 -3 2504 95
1017 -17.03 -1.44 1.0000
-4 9 -3 2265 131
864 -17.03 -1.44 1.0000
4 -9 3 2538 155
1046 -17.03 1.44 1.0000
4 -9 3 2305 109
895 -17.03 1.44 1.0000
-6 9 -3 1761 146
1367 0.05 -9.32 1.0000
-6 9 -3 1748 116
1355 0.05 -9.32 1.0000
6 -9 3 1624 181
1260 0.05 9.32 1.0000
-8 9 -3 2418 236
4068 5.45 13.84 1.0000
-8 9 -3 2574 221
4271 5.45 13.84 1.0000
8 -9 3 2555 283
4242 5.45 -13.84 1.0000
-10 9 -3 1739 198
3334 -25.43 -7.12 1.0000
10 -9 3 1715 263
3292 -25.43 7.12 1.0000
12 -9 3 1972 274
2595 2.35 -6.25 1.0000
14 -9 3 195 180

370	-12.00	9.45	1.0000	
16	-9	3	313	227
905	-8.62	-7.28	1.0000	
18	-9	3	13	219
217	8.99	3.62	1.0000	
20	-9	3	239	251
245	4.13	0.88	1.0000	
22	-9	3	24	245
-33	2.40	-3.53	1.0000	
24	-9	3	4	308
-140	0.87	1.39	1.0000	
19	8	-3	439	277
723	5.64	2.50	1.0000	
19	8	-3	125	301
284	5.64	2.50	1.0000	
17	8	-3	519	281
566	-0.92	4.74	1.0000	
17	8	-3	576	232
628	-0.92	4.74	1.0000	
15	8	-3	927	274
1626	-3.43	-10.99	1.0000	
15	8	-3	1091	300
1848	-3.43	-10.99	1.0000	
13	8	-3	346	150
-87	7.25	14.41	1.0000	
13	8	-3	477	208
162	7.25	14.41	1.0000	
11	8	-3	881	271
2180	-17.19	-10.10	1.0000	
11	8	-3	916	233
2241	-17.19	-10.10	1.0000	
9	8	-3	2276	271
3887	5.69	-13.69	1.0000	
9	8	-3	2385	285
4031	5.69	-13.69	1.0000	
7	8	-3	7395	372
7205	-0.32	36.48	1.0000	
7	8	-3	7215	381
7003	-0.32	36.48	1.0000	
5	8	-3	841	189
1230	12.41	-43.53	1.0000	
5	8	-3	753	183
1955	12.41	-43.53	1.0000	
3	8	-3	552	181

2466 34.01 22.12 1.0000
3 8 -3 482 185
1712 34.01 22.12 1.0000
-3 -8 3 467 187
1837 34.01 -22.12 1.0000
1 8 -3 3710 139
1091 33.80 -1.79 1.0000
-1 -8 3 3657 171
1054 33.80 1.79 1.0000
-1 8 -3 2546 232
4246 11.91 -12.32 1.0000
-1 8 -3 2425 184
4085 11.91 -12.32 1.0000
1 -8 3 2228 250
3824 11.91 12.32 1.0000
1 -8 3 2331 185
3962 11.91 12.32 1.0000
1 -8 3 2018 249
3538 11.91 12.32 1.0000
-3 8 -3 3373 124
681 22.03 17.99 1.0000
-3 8 -3 4068 88
1017 22.03 17.99 1.0000
3 -8 3 3752 146
858 22.03 -17.99 1.0000
3 -8 3 3889 93
925 22.03 -17.99 1.0000
-5 8 -3 2208 138
1449 -11.80 -1.59 1.0000
-5 8 -3 1993 111
1270 -11.80 -1.59 1.0000
5 -8 3 2171 164
1411 -11.80 1.59 1.0000
-7 8 -3 5492 248
5252 3.53 3.14 1.0000
-7 8 -3 5336 263
5099 3.53 3.14 1.0000
7 -8 3 5020 297
4788 3.53 -3.14 1.0000
-9 8 -3 697 101
637 -1.11 1.67 1.0000
9 -8 3 468 134
418 -1.11 -1.67 1.0000
-11 8 -3 387 114

629 3.92 2.59 1.0000
11 -8 3 190 155
367 3.92 -2.59 1.0000
-13 8 -3 1799 231
3719 16.58 -1.47 1.0000
13 -8 3 1892 307
3845 16.58 1.47 1.0000
15 -8 3 257 177
201 -1.32 2.22 1.0000
17 -8 3 55 195
-21 0.11 -6.43 1.0000
19 -8 3 203 236
70 -4.89 5.17 1.0000
21 -8 3 436 281
812 -6.55 -0.88 1.0000
23 -8 3 438 298
484 -0.34 0.87 1.0000
25 -8 3 26 314
14 -1.54 -0.64 1.0000
20 7 -3 161 264
195 -8.19 -3.91 1.0000
20 7 -3 350 242
461 -8.19 -3.91 1.0000
18 7 -3 195 206
303 2.53 2.17 1.0000
18 7 -3 104 240
185 2.53 2.17 1.0000
16 7 -3 2918 311
2226 -7.86 -2.17 1.0000
16 7 -3 3096 361
2383 -7.86 -2.17 1.0000
14 7 -3 19 172
-14 -3.63 -1.03 1.0000
14 7 -3 406 217
341 -3.63 -1.03 1.0000
12 7 -3 404 80
140 12.29 -1.28 1.0000
12 7 -3 404 152
140 12.29 -1.28 1.0000
12 7 -3 589 180
298 12.29 -1.28 1.0000
10 7 -3 666 115
1353 -10.29 3.20 1.0000
10 7 -3 816 220

1567	-10.29	3.20	1.0000	
10	7	-3	682	201
1376	-10.29	3.20	1.0000	
8	7	-3	4787	297
4579	3.76	-3.11	1.0000	
8	7	-3	4927	286
4717	3.76	-3.11	1.0000	
6	7	-3	5019	194
2363	-13.07	-14.27	1.0000	
6	7	-3	4748	202
2178	-13.07	-14.27	1.0000	
4	7	-3	14218	1187
27304	-36.98	15.35	1.0000	
4	7	-3	16571	1197
30531	-36.98	15.35	1.0000	
-4	-7	3	15551	1198
29146	-36.98	-15.35	1.0000	
2	7	-3	979	283
5800	-25.11	-44.68	1.0000	
2	7	-3	865	284
5375	-25.11	-44.68	1.0000	
-2	-7	3	797	283
5110	-25.11	44.68	1.0000	
0	7	-3	13387	766
19135	-43.22	32.44	1.0000	
0	7	-3	13630	802
19460	-43.22	32.44	1.0000	
0	-7	3	13286	814
19004	-43.22	-32.44	1.0000	
0	-7	3	13162	803
18837	-43.22	-32.44	1.0000	
-2	7	-3	81	132
695	-18.46	-20.92	1.0000	
-2	7	-3	74	80
736	-18.46	-20.92	1.0000	
-2	7	-3	61	121
855	-18.46	-20.92	1.0000	
2	-7	3	63	81
761	-18.46	20.92	1.0000	
2	-7	3	62	132
770	-18.46	20.92	1.0000	
2	-7	3	63	110
763	-18.46	20.92	1.0000	
-4	7	-3	2287	170

1375 -3.11 -11.09 1.0000
-4 7 -3 2192 106
1300 -3.11 -11.09 1.0000
-4 7 -3 2259 133
1353 -3.11 -11.09 1.0000
4 -7 3 2480 134
1528 -3.11 11.09 1.0000
4 -7 3 2374 154
1444 -3.11 11.09 1.0000
4 -7 3 2398 111
1463 -3.11 11.09 1.0000
-6 7 -3 1650 303
6158 2.34 35.75 1.0000
-6 7 -3 1834 292
6524 2.34 35.75 1.0000
6 -7 3 1764 331
6380 2.34 -35.75 1.0000
6 -7 3 1774 307
6400 2.34 -35.75 1.0000
-8 7 -3 7327 346
7141 -0.41 -36.59 1.0000
-8 7 -3 7904 346
7788 -0.41 -36.59 1.0000
8 -7 3 7695 352
7530 -0.41 36.59 1.0000
8 -7 3 7955 395
7820 -0.41 36.59 1.0000
-10 7 -3 702 81
155 12.11 21.94 1.0000
10 -7 3 750 126
23 12.11 -21.94 1.0000
10 -7 3 737 77
287 12.11 -21.94 1.0000
-12 7 -3 2509 137
1151 -17.02 -0.97 1.0000
12 -7 3 2470 200
1115 -17.02 0.97 1.0000
-14 7 -3 1545 154
1091 -4.23 -4.09 1.0000
14 -7 3 1520 207
1072 -4.23 4.09 1.0000
16 -7 3 40 180
-9 5.28 -1.39 1.0000
18 -7 3 405 219

308	-2.31	-0.03	1.0000	
20	-7	3	77	226
39	6.78	1.94	1.0000	
22	-7	3	8	258
13	5.35	-0.08	1.0000	
24	-7	3	353	310
479	2.82	-1.13	1.0000	
21	6	-3	1223	253
951	4.58	-2.02	1.0000	
21	6	-3	700	287
495	4.58	-2.02	1.0000	
19	6	-3	1600	302
1291	2.84	2.10	1.0000	
19	6	-3	1246	235
975	2.84	2.10	1.0000	
17	6	-3	2174	365
2228	3.30	-1.27	1.0000	
17	6	-3	1883	333
1932	3.30	-1.27	1.0000	
15	6	-3	3	200
381	17.28	2.15	1.0000	
15	6	-3	3	218
431	17.28	2.15	1.0000	
13	6	-3	2116	264
2073	-3.63	-5.69	1.0000	
13	6	-3	1954	261
1911	-3.63	-5.69	1.0000	
11	6	-3	399	155
165	2.85	5.79	1.0000	
11	6	-3	305	143
106	2.85	5.79	1.0000	
11	6	-3	312	88
110	2.85	5.79	1.0000	
9	6	-3	1814	199
1433	0.05	9.32	1.0000	
9	6	-3	1460	200
1111	0.05	9.32	1.0000	
7	6	-3	1576	330
6036	2.47	-35.96	1.0000	
7	6	-3	1575	328
6033	2.47	-35.96	1.0000	
5	6	-3	2735	320
6113	20.41	34.95	1.0000	
5	6	-3	2693	316

6036	20.41	34.95	1.0000	
-5	-6	3	2946	324
6503	20.41	-34.95	1.0000	
3	6	-3	219	201
3253	33.43	-20.64	1.0000	
3	6	-3	321	212
3675	33.43	-20.64	1.0000	
-3	-6	3	220	222
3229	33.43	20.64	1.0000	
-3	-6	3	277	207
3475	33.43	20.64	1.0000	
1	6	-3	3864	161
1893	15.56	-4.58	1.0000	
-1	-6	3	4214	156
2140	15.56	4.58	1.0000	
-1	-6	3	4125	175
2077	15.56	4.58	1.0000	
-1	6	-3	5480	217
4764	44.93	27.53	1.0000	
-1	6	-3	5693	252
5071	44.93	27.53	1.0000	
1	-6	3	5322	254
4590	44.93	-27.53	1.0000	
1	-6	3	5451	216
4779	44.93	-27.53	1.0000	
1	-6	3	5651	261
5070	44.93	-27.53	1.0000	
-3	6	-3	3130	297
5384	-12.45	-19.41	1.0000	
-3	6	-3	3540	257
5945	-12.45	-19.41	1.0000	
-3	6	-3	3367	277
5710	-12.45	-19.41	1.0000	
3	-6	3	3578	257
5991	-12.45	19.41	1.0000	
3	-6	3	3289	289
5598	-12.45	19.41	1.0000	
3	-6	3	3427	278
5786	-12.45	19.41	1.0000	
-5	6	-3	2074	150
2318	-4.78	5.02	1.0000	
-5	6	-3	1925	133
2158	-4.78	5.02	1.0000	
-5	6	-3	1813	181

2038	-4.78	5.02	1.0000	
5	-6	3	1997	141
2235	-4.78	-5.02	1.0000	
5	-6	3	2049	153
2291	-4.78	-5.02	1.0000	
5	-6	3	1898	168
2129	-4.78	-5.02	1.0000	
-7	6	-3	5187	156
2464	-13.19	14.31	1.0000	
-7	6	-3	5128	152
2423	-13.19	14.31	1.0000	
7	-6	3	5371	164
2592	-13.19	-14.31	1.0000	
7	-6	3	5070	191
2384	-13.19	-14.31	1.0000	
-9	6	-3	1017	113
985	-17.08	-0.88	1.0000	
9	-6	3	912	158
846	-17.08	0.88	1.0000	
9	-6	3	1037	123
999	-17.08	0.88	1.0000	
-11	6	-3	1351	145
1726	6.27	0.65	1.0000	
11	-6	3	1693	156
2115	6.27	-0.65	1.0000	
11	-6	3	1184	207
1536	6.27	-0.65	1.0000	
-13	6	-3	1469	155
1487	-0.63	5.30	1.0000	
13	-6	3	1268	227
1277	-0.63	-5.30	1.0000	
13	-6	3	1736	159
1753	-0.63	-5.30	1.0000	
-15	6	-3	935	147
965	0.22	-0.62	1.0000	
15	-6	3	949	208
980	0.22	0.62	1.0000	
17	-6	3	1193	299
1765	-0.94	8.02	1.0000	
19	-6	3	503	241
530	-5.81	-8.38	1.0000	
21	-6	3	139	255
128	-2.19	4.48	1.0000	
23	-6	3	448	271

323 -6.60 0.45 1.0000
25 -6 3 417 330
387 -0.48 -0.92 1.0000
24 5 -3 1137 283
1017 -2.51 0.97 1.0000
24 5 -3 10 315
-31 -2.51 0.97 1.0000
22 5 -3 504 268
690 -2.86 -3.79 1.0000
22 5 -3 221 297
344 -2.86 -3.79 1.0000
20 5 -3 2547 415
2747 -1.54 3.14 1.0000
20 5 -3 2440 360
2636 -1.54 3.14 1.0000
18 5 -3 1266 329
1719 -10.42 -4.08 1.0000
18 5 -3 1115 306
1534 -10.42 -4.08 1.0000
16 5 -3 1517 254
991 -6.56 -1.27 1.0000
16 5 -3 1926 244
1327 -6.56 -1.27 1.0000
14 5 -3 1219 288
2625 -9.41 11.87 1.0000
14 5 -3 1100 295
2447 -9.41 11.87 1.0000
12 5 -3 2177 305
4162 -6.67 -14.64 1.0000
12 5 -3 2017 306
3939 -6.67 -14.64 1.0000
10 5 -3 5450 370
6503 9.86 0.11 1.0000
10 5 -3 5809 377
6897 9.86 0.11 1.0000
8 5 -3 2115 203
1354 -11.60 1.83 1.0000
8 5 -3 2521 190
1692 -11.60 1.83 1.0000
6 5 -3 1754 180
1972 -4.94 -5.08 1.0000
6 5 -3 1946 198
2179 -4.94 -5.08 1.0000
-6 -5 3 1927 183

2159	-4.94	5.08	1.0000	
4	5	-3	1772	163
1233	-30.16	-10.65	1.0000	
4	5	-3	1663	135
1066	-30.16	-10.65	1.0000	
-4	-5	3	1726	141
1165	-30.16	10.65	1.0000	
-4	-5	3	1831	167
1321	-30.16	10.65	1.0000	
2	5	-3	22978	506
11664	-37.16	12.78	1.0000	
2	5	-3	22543	494
11353	-37.16	12.78	1.0000	
-2	-5	3	22584	511
11360	-37.16	-12.78	1.0000	
-2	-5	3	22606	493
11375	-37.16	-12.78	1.0000	
0	5	-3	4910	219
4936	-6.39	-34.31	1.0000	
0	5	-3	4915	257
4942	-6.39	-34.31	1.0000	
0	-5	3	4853	252
4847	-6.39	34.31	1.0000	
0	-5	3	5139	270
5189	-6.39	34.31	1.0000	
0	-5	3	5045	248
5077	-6.39	34.31	1.0000	
-2	5	-3	10999	851
21222	-34.33	8.74	1.0000	
-2	5	-3	10314	832
20266	-34.33	8.74	1.0000	
-2	5	-3	10273	858
20208	-34.33	8.74	1.0000	
2	-5	3	10512	832
20538	-34.33	-8.74	1.0000	
2	-5	3	11367	856
21727	-34.33	-8.74	1.0000	
2	-5	3	10136	852
20011	-34.33	-8.74	1.0000	
-4	5	-3	722	129
2141	18.92	18.27	1.0000	
-4	5	-3	769	145
2243	18.92	18.27	1.0000	
-4	5	-3	816	163

2344 18.92 18.27 1.0000
4 -5 3 803 133
2318 18.92 -18.27 1.0000
4 -5 3 765 150
2237 18.92 -18.27 1.0000
4 -5 3 737 154
2177 18.92 -18.27 1.0000
-6 5 -3 2786 287
6214 20.47 -34.94 1.0000
-6 5 -3 2806 289
6251 20.47 -34.94 1.0000
-6 5 -3 2743 321
6135 20.47 -34.94 1.0000
6 -5 3 2916 317
6441 20.47 34.94 1.0000
6 -5 3 2908 291
6427 20.47 34.94 1.0000
-8 5 -3 771 141
2035 12.55 43.66 1.0000
-8 5 -3 773 132
1846 12.55 43.66 1.0000
8 -5 3 758 143
2183 12.55 -43.66 1.0000
8 -5 3 763 189
2277 12.55 -43.66 1.0000
-10 5 -3 7209 454
9949 6.24 -17.09 1.0000
10 -5 3 7577 502
10382 6.24 17.09 1.0000
10 -5 3 7642 445
10460 6.24 17.09 1.0000
-12 5 -3 665 115
753 6.84 -8.88 1.0000
12 -5 3 728 173
831 6.84 8.88 1.0000
-14 5 -3 1391 112
440 -3.02 13.87 1.0000
14 -5 3 1098 166
283 -3.02 -13.87 1.0000
-16 5 -3 1692 195
2144 1.76 -7.30 1.0000
16 -5 3 1960 297
2447 1.76 7.30 1.0000
18 -5 3 59 204

104	1.49	1.56	1.0000	
20	-5	3	401	231
214	3.55	-3.05	1.0000	
22	-5	3	376	274
555	4.93	0.51	1.0000	
24	-5	3	2	280
29	4.73	1.91	1.0000	
25	4	-3	3	338
-2	-0.33	1.48	1.0000	
25	4	-3	1117	307
1177	-0.33	1.48	1.0000	
23	4	-3	17	306
-254	3.47	-0.99	1.0000	
23	4	-3	225	295
365	3.47	-0.99	1.0000	
21	4	-3	837	273
822	0.76	4.83	1.0000	
21	4	-3	946	307
932	0.76	4.83	1.0000	
19	4	-3	1255	264
1057	2.29	1.11	1.0000	
19	4	-3	891	289
725	2.29	1.11	1.0000	
17	4	-3	1731	371
3467	17.56	-4.76	1.0000	
17	4	-3	1721	396
3453	17.56	-4.76	1.0000	
15	4	-3	277	243
769	8.07	7.16	1.0000	
15	4	-3	671	282
1384	8.07	7.16	1.0000	
13	4	-3	1515	246
1390	12.33	-3.47	1.0000	
13	4	-3	1536	243
1412	12.33	-3.47	1.0000	
11	4	-3	1747	224
2062	7.39	0.18	1.0000	
11	4	-3	1678	230
1986	7.39	0.18	1.0000	
9	4	-3	2580	192
1066	-17.15	1.47	1.0000	
9	4	-3	2484	159
1003	-17.15	1.47	1.0000	
7	4	-3	1971	182

1130 -2.91 11.38 1.0000
7 4 -3 2093 156
1225 -2.91 11.38 1.0000
-7 -4 3 2100 152
1230 -2.91 -11.38 1.0000
5 4 -3 793 201
2301 18.99 -18.30 1.0000
5 4 -3 711 164
2124 18.99 -18.30 1.0000
-5 -4 3 720 180
2140 18.99 18.30 1.0000
-5 -4 3 760 163
2227 18.99 18.30 1.0000
3 4 -3 41281 1706
42305 1.37 2.55 1.0000
3 4 -3 41451 1692
42477 1.37 2.55 1.0000
-3 -4 3 40027 1702
41029 1.37 -2.55 1.0000
-3 -4 3 42613 1693
43647 1.37 -2.55 1.0000
1 4 -3 30182 1419
35699 51.30 13.77 1.0000
-1 -4 3 29458 1417
34817 51.30 -13.77 1.0000
-1 -4 3 29378 1401
34725 51.30 -13.77 1.0000
-1 4 -3 25129 1419
35456 -12.85 -27.10 1.0000
-1 4 -3 24870 1419
35146 -12.85 -27.10 1.0000
-1 4 -3 24379 1401
34559 -12.85 -27.10 1.0000
1 -4 3 25282 1423
35625 -12.85 27.10 1.0000
1 -4 3 25306 1414
35654 -12.85 27.10 1.0000
-3 4 -3 1836 112
1941 7.80 29.15 1.0000
-3 4 -3 1710 133
1748 7.80 29.15 1.0000
-3 4 -3 1639 132
1637 7.80 29.15 1.0000
3 -4 3 1760 147

1771 7.80 -29.15 1.0000
3 -4 3 1590 126
1507 7.80 -29.15 1.0000
3 -4 3 1920 112
2012 7.80 -29.15 1.0000
-5 4 -3 1671 94
1064 -30.26 10.51 1.0000
-5 4 -3 1701 118
1111 -30.26 10.51 1.0000
-5 4 -3 1635 100
1009 -30.26 10.51 1.0000
5 -4 3 1706 111
1118 -30.26 -10.51 1.0000
5 -4 3 1652 99
1035 -30.26 -10.51 1.0000
5 -4 3 1627 114
995 -30.26 -10.51 1.0000
-7 4 -3 15934 1246
29813 -37.37 -15.33 1.0000
-7 4 -3 16374 1255
30414 -37.37 -15.33 1.0000
7 -4 3 17860 1251
32422 -37.37 15.33 1.0000
7 -4 3 17495 1284
31929 -37.37 15.33 1.0000
-9 4 -3 9363 490
11374 -8.89 15.81 1.0000
9 -4 3 8869 528
10852 -8.89 -15.81 1.0000
9 -4 3 9599 479
11671 -8.89 -15.81 1.0000
-11 4 -3 1674 208
3614 -16.54 3.73 1.0000
11 -4 3 1654 264
3584 -16.54 -3.73 1.0000
-13 4 -3 2965 210
3309 -2.63 -0.65 1.0000
13 -4 3 3341 282
3706 -2.63 0.65 1.0000
-15 4 -3 5932 280
5082 5.17 0.80 1.0000
15 -4 3 5777 365
4937 5.17 -0.80 1.0000
-17 4 -3 439 138

446	-6.06	4.12	1.0000	
17	-4	3	544	222
561	-6.06	-4.12	1.0000	
19	-4	3	876	242
615	-0.32	7.19	1.0000	
21	-4	3	839	291
1279	-4.74	-3.74	1.0000	
23	-4	3	17	248
-55	-3.56	-0.24	1.0000	
25	-4	3	1	302
-91	-0.84	0.29	1.0000	
26	3	-3	11	367
39	2.08	1.55	1.0000	
26	3	-3	252	330
355	2.08	1.55	1.0000	
24	3	-3	23	307
-28	-3.86	-1.53	1.0000	
24	3	-3	23	343
-14	-3.86	-1.53	1.0000	
22	3	-3	4	291
-171	-1.76	-0.15	1.0000	
22	3	-3	158	308
173	-1.76	-0.15	1.0000	
20	3	-3	271	246
337	-1.80	-0.07	1.0000	
20	3	-3	564	296
658	-1.80	-0.07	1.0000	
18	3	-3	904	232
408	-8.53	1.11	1.0000	
18	3	-3	1517	269
846	-8.53	1.11	1.0000	
16	3	-3	1057	201
452	-10.26	4.25	1.0000	
16	3	-3	905	209
352	-10.26	4.25	1.0000	
14	3	-3	1587	286
2787	-10.55	-18.71	1.0000	
14	3	-3	1987	289
3367	-10.55	-18.71	1.0000	
12	3	-3	226	149
131	-6.88	16.80	1.0000	
12	3	-3	218	136
167	-6.88	16.80	1.0000	
10	3	-3	294	143

287	0.24	-3.93	1.0000	
10	3	-3	507	134
505	0.24	-3.93	1.0000	
8	3	-3	4508	173
1243	21.89	-18.09	1.0000	
8	3	-3	4482	167
1229	21.89	-18.09	1.0000	
-8	-3	3	3786	156
876	21.89	18.09	1.0000	
6	3	-3	2765	271
4878	-12.29	19.42	1.0000	
6	3	-3	2601	254
4646	-12.29	19.42	1.0000	
-6	-3	3	2579	256
4619	-12.29	-19.42	1.0000	
-6	-3	3	2933	255
5117	-12.29	-19.42	1.0000	
4	3	-3	1638	134
1582	7.81	-28.81	1.0000	
-4	-3	3	1752	155
1809	7.81	28.81	1.0000	
-4	-3	3	1520	129
1445	7.81	28.81	1.0000	
2	3	-3	45572	1600
38473	-19.83	5.84	1.0000	
2	3	-3	47218	1621
39992	-19.83	5.84	1.0000	
-2	-3	3	49067	1620
41710	-19.83	-5.84	1.0000	
-2	-3	3	48387	1603
41081	-19.83	-5.84	1.0000	
0	3	-3	13932	868
20801	-32.98	18.41	1.0000	
0	-3	3	13552	865
20324	-32.98	-18.41	1.0000	
0	-3	3	14369	848
21360	-32.98	-18.41	1.0000	
0	-3	3	14669	851
21738	-32.98	-18.41	1.0000	
-2	3	-3	44403	2666
66272	35.83	-19.17	1.0000	
-2	3	-3	46422	2674
68734	35.83	-19.17	1.0000	
-2	3	-3	45282	2677

```
67345    35.83   -19.17  1.0000
   2  -3    3   45257     2678
67319    35.83    19.17  1.0000
   2  -3    3   44426     2672
66305    35.83    19.17  1.0000
   2  -3    3   46943     2665
69372    35.83    19.17  1.0000
  -4   3   -3   44336     1841
45467     1.48    -2.69  1.0000
  -4   3   -3   44842     1830
45979     1.48    -2.69  1.0000
  -4   3   -3   44654     1830
45789     1.48    -2.69  1.0000
   4  -3    3   46119     1835
47280     1.48     2.69  1.0000
   4  -3    3   45595     1830
46749     1.48     2.69  1.0000
   4  -3    3   43929     1841
45062     1.48     2.69  1.0000
  -6   3   -3     507      227
4283    33.63    20.34  1.0000
  -6   3   -3     475      199
4180    33.63    20.34  1.0000
  -6   3   -3     463      206
4142    33.63    20.34  1.0000
   6  -3    3     401      208
3961    33.63   -20.34  1.0000
   6  -3    3     520      208
4346    33.63   -20.34  1.0000
   6  -3    3     530      230
4378    33.63   -20.34  1.0000
  -8   3   -3     519      152
2411    34.11   -22.26  1.0000
   8  -3    3     575      194
2615    34.11    22.26  1.0000
 -10   3   -3   10079      477
10483    16.21    12.68  1.0000
  10  -3    3   11038      528
11474    16.21   -12.68  1.0000
 -12   3   -3    1259      159
2074     2.34     8.54  1.0000
  12  -3    3    1274      226
2093     2.34    -8.54  1.0000
 -14   3   -3    2945      145
```

1144	7.14	-17.56	1.0000	
14	-3	3	3141	210
1261	7.14	17.56	1.0000	
-16	3	-3	1280	202
2407	-9.74	6.17	1.0000	
16	-3	3	1710	308
2986	-9.74	-6.17	1.0000	
-18	3	-3	1088	175
1205	6.71	1.27	1.0000	
18	-3	3	1205	256
1331	6.71	-1.27	1.0000	
20	-3	3	829	257
1007	0.82	5.35	1.0000	
22	-3	3	7	241
21	3.71	-3.01	1.0000	
24	-3	3	11	285
-164	2.89	-0.25	1.0000	
26	-3	3	237	345
271	1.26	-1.01	1.0000	
25	2	-3	10	340
-315	0.00	2.69	1.0000	
25	2	-3	10	329
-461	0.00	2.69	1.0000	
23	2	-3	10	302
-352	0.76	2.63	1.0000	
23	2	-3	201	301
295	0.76	2.63	1.0000	
21	2	-3	2	254
-72	-0.25	-1.19	1.0000	
21	2	-3	226	264
186	-0.25	-1.19	1.0000	
19	2	-3	491	267
878	9.19	-1.37	1.0000	
19	2	-3	382	247
722	9.19	-1.37	1.0000	
17	2	-3	1749	324
2017	5.88	7.26	1.0000	
17	2	-3	2117	316
2422	5.88	7.26	1.0000	
15	2	-3	761	264
1502	12.70	-9.39	1.0000	
15	2	-3	697	246
1401	12.70	-9.39	1.0000	
13	2	-3	2992	183

873	16.25	15.28	1.0000	
13	2	-3	3438	199
1123	16.25	15.28	1.0000	
11	2	-3	268	160
767	-11.66	-0.10	1.0000	
11	2	-3	210	139
659	-11.66	-0.10	1.0000	
9	2	-3	2022	170
1185	-6.96	-11.08	1.0000	
9	2	-3	1728	162
954	-6.96	-11.08	1.0000	
7	2	-3	54	136
855	-18.60	21.09	1.0000	
7	2	-3	48	148
1006	-18.60	21.09	1.0000	
-7	-2	3	80	128
715	-18.60	-21.09	1.0000	
-7	-2	3	77	121
731	-18.60	-21.09	1.0000	
5	2	-3	8512	778
17758	-34.48	-8.89	1.0000	
5	2	-3	9023	770
18493	-34.48	-8.89	1.0000	
-5	-2	3	9568	770
19274	-34.48	8.89	1.0000	
-5	-2	3	9414	781
19055	-34.48	8.89	1.0000	
3	2	-3	42390	2647
63987	36.17	19.16	1.0000	
3	2	-3	42520	2629
64146	36.17	19.16	1.0000	
-3	-2	3	47267	2632
69944	36.17	-19.16	1.0000	
-3	-2	3	46042	2649
68452	36.17	-19.16	1.0000	
1	2	-3	17264	1143
27248	-14.88	-27.59	1.0000	
-1	-2	3	18849	1143
29240	-14.88	27.59	1.0000	
-1	2	-3	14675	804
20118	17.92	20.17	1.0000	
-1	2	-3	14496	824
19904	17.92	20.17	1.0000	
-1	2	-3	14667	804

20108	17.92	20.17	1.0000	
1	-2	3	14636	819
20071	17.92	-20.17	1.0000	
1	-2	3	14488	802
19895	17.92	-20.17	1.0000	
-3	2	-3	49376	1651
41995	-19.70	-5.91	1.0000	
-3	2	-3	49043	1650
41687	-19.70	-5.91	1.0000	
-3	2	-3	47707	1645
40453	-19.70	-5.91	1.0000	
3	-2	3	47560	1649
40307	-19.70	5.91	1.0000	
3	-2	3	49148	1651
41775	-19.70	5.91	1.0000	
3	-2	3	49743	1645
42325	-19.70	5.91	1.0000	
-5	2	-3	24199	520
12458	-37.40	-12.58	1.0000	
-5	2	-3	24737	514
12846	-37.40	-12.58	1.0000	
-5	2	-3	23852	532
12208	-37.40	-12.58	1.0000	
5	-2	3	24314	521
12564	-37.40	12.58	1.0000	
5	-2	3	23523	533
11995	-37.40	12.58	1.0000	
5	-2	3	24492	521
12693	-37.40	12.58	1.0000	
-7	2	-3	899	271
5512	-25.26	44.45	1.0000	
-7	2	-3	1043	295
6024	-25.26	44.45	1.0000	
7	-2	3	1070	300
6103	-25.26	-44.45	1.0000	
-9	2	-3	1912	205
3901	-41.51	-16.56	1.0000	
9	-2	3	1837	245
3745	-41.51	16.56	1.0000	
-11	2	-3	6429	288
5892	-11.43	8.49	1.0000	
11	-2	3	6353	341
5803	-11.43	-8.49	1.0000	
-13	2	-3	4720	276

5444	4.01	3.61	1.0000	
13	-2	3	4484	341
5190	4.01	-3.61	1.0000	
-15	2	-3	928	131
773	-3.83	-3.56	1.0000	
15	-2	3	802	192
654	-3.83	3.56	1.0000	
-17	2	-3	1263	174
1259	8.83	3.46	1.0000	
17	-2	3	1657	285
1671	8.83	-3.46	1.0000	
19	-2	3	21	186
-139	-3.08	-2.50	1.0000	
21	-2	3	294	253
432	-4.13	2.72	1.0000	
23	-2	3	255	275
189	1.44	-3.91	1.0000	
25	-2	3	70	321
23	-2.82	1.45	1.0000	
26	1	-3	37	341
-319	-4.43	-2.87	1.0000	
24	1	-3	16	286
-246	3.47	0.30	1.0000	
22	1	-3	86	277
261	0.85	6.01	1.0000	
20	1	-3	837	263
612	-6.57	-6.67	1.0000	
20	1	-3	389	233
209	-6.57	-6.67	1.0000	
18	1	-3	2700	324
2829	1.35	4.10	1.0000	
16	1	-3	857	205
454	-13.33	-1.92	1.0000	
16	1	-3	756	174
369	-13.33	-1.92	1.0000	
14	1	-3	232	137
259	-4.60	3.16	1.0000	
14	1	-3	218	155
243	-4.60	3.16	1.0000	
12	1	-3	2306	250
3603	-0.30	-11.95	1.0000	
12	1	-3	2025	262
3247	-0.30	-11.95	1.0000	
10	1	-3	1408	157

825 11.44 0.79 1.0000
10 1 -3 1345 143
774 11.44 0.79 1.0000
8 1 -3 2039 227
3595 12.01 12.52 1.0000
8 1 -3 2170 223
3775 12.01 12.52 1.0000
-8 -1 3 1915 214
3421 12.01 -12.52 1.0000
-8 -1 3 1886 215
3380 12.01 -12.52 1.0000
6 1 -3 5326 242
4578 45.10 -27.36 1.0000
6 1 -3 5280 232
4510 45.10 -27.36 1.0000
-6 -1 3 5169 238
4289 45.10 27.36 1.0000
-6 -1 3 5222 226
4368 45.10 27.36 1.0000
4 1 -3 23491 1363
33599 -13.10 27.23 1.0000
4 1 -3 22740 1378
32694 -13.10 27.23 1.0000
-4 -1 3 24323 1380
34611 -13.10 -27.23 1.0000
-4 -1 3 25193 1363
35653 -13.10 -27.23 1.0000
2 1 -3 14988 851
20403 17.78 -19.90 1.0000
-2 -1 3 15553 851
21071 17.78 19.90 1.0000
0 1 -3 14574 549
12880 9.52 -7.82 1.0000
0 -1 3 15045 547
13280 9.52 7.82 1.0000
-2 1 -3 17908 1138
28028 -14.66 27.54 1.0000
-2 1 -3 17605 1145
27648 -14.66 27.54 1.0000
-2 1 -3 18746 1136
29078 -14.66 27.54 1.0000
2 -1 3 18251 1136
28457 -14.66 -27.54 1.0000
2 -1 3 18972 1144

29359 -14.66 -27.54 1.0000
-4 1 -3 29844 1403
35220 51.39 -13.96 1.0000
-4 1 -3 29357 1393
34660 51.39 -13.96 1.0000
-4 1 -3 29742 1396
35103 51.39 -13.96 1.0000
4 -1 3 29700 1405
35104 51.39 13.96 1.0000
4 -1 3 30148 1396
35620 51.39 13.96 1.0000
4 -1 3 28901 1391
34184 51.39 13.96 1.0000
-6 1 -3 3709 119
1764 15.81 4.50 1.0000
-6 1 -3 3800 135
1827 15.81 4.50 1.0000
6 -1 3 3624 119
1706 15.81 -4.50 1.0000
6 -1 3 3628 140
1709 15.81 -4.50 1.0000
-8 1 -3 3564 105
976 34.00 1.62 1.0000
8 -1 3 3037 124
646 34.00 -1.62 1.0000
-10 1 -3 1052 95
488 21.53 -9.33 1.0000
10 -1 3 992 134
461 21.53 9.33 1.0000
-12 1 -3 778 141
1482 9.23 -1.36 1.0000
12 -1 3 895 213
1645 9.23 1.36 1.0000
-14 1 -3 73 115
441 -10.68 9.39 1.0000
14 -1 3 104 151
50 -10.68 -9.39 1.0000
-16 1 -3 744 133
600 3.84 -6.80 1.0000
16 -1 3 475 193
346 3.84 6.80 1.0000
-18 1 -3 173 127
-54 -10.16 -5.16 1.0000
18 -1 3 173 196

-37	-10.16	5.16	1.0000	
20	-1	3	878	232
336	5.72	-8.15	1.0000	
22	-1	3	284	277
601	1.42	7.16	1.0000	
24	-1	3	20	292
-39	-1.04	-3.71	1.0000	
26	-1	3	17	320
-195	2.73	-2.28	1.0000	
25	0	-3	10	305
-124	2.72	0.19	1.0000	
23	0	-3	8	273
54	-3.83	-1.79	1.0000	
21	0	-3	47	238
-94	2.75	5.29	1.0000	
21	0	-3	27	256
3	2.75	5.29	1.0000	
19	0	-3	262	233
289	1.14	2.26	1.0000	
19	0	-3	172	197
193	1.14	2.26	1.0000	
17	0	-3	1863	281
2401	4.43	-6.09	1.0000	
15	0	-3	1369	257
1925	14.97	10.63	1.0000	
15	0	-3	1125	223
1589	14.97	10.63	1.0000	
13	0	-3	443	161
660	-5.64	-8.81	1.0000	
11	0	-3	824	185
1242	-5.21	2.99	1.0000	
11	0	-3	999	177
1455	-5.21	2.99	1.0000	
9	0	-3	12491	691
16204	-24.02	32.47	1.0000	
9	0	-3	12137	685
15771	-24.02	32.47	1.0000	
-9	0	3	11925	685
15481	-24.02	-32.47	1.0000	
7	0	-3	13126	795
18799	-43.38	-32.48	1.0000	
7	0	-3	13133	791
18808	-43.38	-32.48	1.0000	
-7	0	3	12750	793

18290	-43.38	32.48	1.0000	
-7	0	3	13791	790
19684	-43.38	32.48	1.0000	
5	0	-3	4637	240
4585	-6.37	34.37	1.0000	
5	0	-3	4468	221
4381	-6.37	34.37	1.0000	
-5	0	3	4639	218
4608	-6.37	-34.37	1.0000	
-5	0	3	4455	235
4385	-6.37	-34.37	1.0000	
3	0	-3	14014	876
20880	-32.95	-18.67	1.0000	
-3	0	3	14744	877
21796	-32.95	18.67	1.0000	
1	0	-3	13279	494
11547	9.88	7.95	1.0000	
-1	0	3	13509	494
11807	9.88	-7.95	1.0000	
-1	0	-3	16191	601
14371	9.43	7.69	1.0000	
1	0	3	16610	601
14816	9.43	-7.69	1.0000	
-3	0	-3	14724	873
21750	-32.83	-18.52	1.0000	
-3	0	-3	14511	869
21482	-32.83	-18.52	1.0000	
-3	0	-3	14085	870
20944	-32.83	-18.52	1.0000	
3	0	3	15241	870
22394	-32.83	18.52	1.0000	
3	0	3	14484	874
21442	-32.83	18.52	1.0000	
-5	0	-3	5113	245
5174	-6.46	34.48	1.0000	
-5	0	-3	4697	230
4674	-6.46	34.48	1.0000	
5	0	3	4674	230
4667	-6.46	-34.48	1.0000	
5	0	3	5274	252
5388	-6.46	-34.48	1.0000	
-7	0	-3	12240	763
17704	-43.43	-32.81	1.0000	
-7	0	-3	13237	787

19053	-43.43	-32.81	1.0000	
7	0	3	13263	792
19085	-43.43	32.81	1.0000	
-9	0	-3	11650	644
15288	-24.41	32.28	1.0000	
9	0	3	11730	681
15358	-24.41	-32.28	1.0000	
-11	0	-3	1143	136
1640	-5.39	2.80	1.0000	
11	0	3	1182	200
1683	-5.39	-2.80	1.0000	
-13	0	-3	462	117
676	-5.55	-8.59	1.0000	
13	0	3	532	165
772	-5.55	8.59	1.0000	
-15	0	-3	991	154
1411	15.14	10.70	1.0000	
15	0	3	1027	259
1452	15.14	-10.70	1.0000	
-17	0	-3	571	149
874	4.75	-6.27	1.0000	
17	0	3	637	237
959	4.75	6.27	1.0000	
-19	0	-3	320	150
349	1.11	2.20	1.0000	
19	0	3	376	233
407	1.11	-2.20	1.0000	
21	0	3	320	264
605	2.74	-5.23	1.0000	
23	0	3	144	268
273	-3.52	2.01	1.0000	
25	0	3	2	318
7	2.71	-0.19	1.0000	
26	-1	-3	16	345
-109	2.53	2.42	1.0000	
24	-1	-3	199	325
332	-1.12	3.68	1.0000	
22	-1	-3	8	253
77	1.50	-6.99	1.0000	
22	-1	-3	70	275
246	1.50	-6.99	1.0000	
20	-1	-3	574	237
159	5.84	8.13	1.0000	
20	-1	-3	852	218

```
319     5.84     8.13  1.0000
  18   -1   -3      246       188
360   -10.23    -5.30  1.0000
  18   -1   -3      254       224
374   -10.23    -5.30  1.0000
  16   -1   -3      871       205
719     3.87    -6.88  1.0000
  16   -1   -3      926       174
772     3.87    -6.88  1.0000
  12   -1   -3     1281       202
2129     9.01    -1.37  1.0000
  12   -1   -3     1324       215
2185     9.01    -1.37  1.0000
  10   -1   -3      924       112
340    21.43    -8.84  1.0000
  10   -1   -3      902       117
313    21.43    -8.84  1.0000
   8   -1   -3     2775       103
506    33.57     1.66  1.0000
  -8    1    3     2722        97
477    33.57    -1.66  1.0000
  -8    1    3     2695        99
462    33.57    -1.66  1.0000
   6   -1   -3     4279       160
2165    15.75     4.65  1.0000
   6   -1   -3     4216       143
2120    15.75     4.65  1.0000
  -6    1    3     4199       159
2108    15.75    -4.65  1.0000
  -6    1    3     4417       140
2263    15.75    -4.65  1.0000
   4   -1   -3    31373      1489
37072    51.27   -13.58  1.0000
   4   -1   -3    31036      1507
36686    51.27   -13.58  1.0000
  -4    1    3    32267      1488
38147    51.27    13.58  1.0000
  -4    1    3    31740      1506
37544    51.27    13.58  1.0000
   2   -1   -3    18978      1198
29400   -14.87    27.59  1.0000
  -2    1    3    19359      1197
29874   -14.87   -27.59  1.0000
   0   -1   -3    14737       541
```

```
13004     9.62   -7.75  1.0000
    0    1    3   14637      539
12864     9.62    7.75  1.0000
   -2   -1   -3   13965      768
19172    17.86  -19.85  1.0000
   -2   -1   -3   13989      764
19201    17.86  -19.85  1.0000
    2    1    3   13900      767
19094    17.86   19.85  1.0000
    2    1    3   13184      764
18240    17.86   19.85  1.0000
   -4   -1   -3   25856     1414
36332   -13.01   26.98  1.0000
   -4   -1   -3   23829     1403
33909   -13.01   26.98  1.0000
    4    1    3   24928     1402
35238   -13.01  -26.98  1.0000
    4    1    3   24944     1415
35257   -13.01  -26.98  1.0000
   -6   -1   -3    6312      282
6066    45.07  -27.76  1.0000
   -6   -1   -3    6637      306
6518    45.07  -27.76  1.0000
    6    1    3    6501      284
6268    45.07   27.76  1.0000
    6    1    3    6482      311
6242    45.07   27.76  1.0000
   -8   -1   -3    2451      218
4143    12.05   12.44  1.0000
    8    1    3    2815      268
4622    12.05  -12.44  1.0000
  -10   -1   -3    1795      120
1123    11.89    0.80  1.0000
   10    1    3    1828      183
1154    11.89   -0.80  1.0000
  -12   -1   -3    2313      209
3626    -0.61  -11.90  1.0000
   12    1    3    2384      284
3716    -0.61   11.90  1.0000
  -14   -1   -3     236       99
259    -4.21    3.07  1.0000
   14    1    3     279      169
309    -4.21   -3.07  1.0000
  -16   -1   -3     352      103
```

53 -13.80 -1.85 1.0000
16 1 3 357 193
56 -13.80 1.85 1.0000
-18 -1 -3 1242 183
1317 1.29 4.18 1.0000
18 1 3 1320 275
1393 1.29 -4.18 1.0000
-20 -1 -3 388 164
212 -6.67 -6.60 1.0000
20 1 3 307 225
140 -6.67 6.60 1.0000
22 1 3 112 262
308 0.86 -6.06 1.0000
24 1 3 15 290
-18 3.36 -0.27 1.0000
26 1 3 38 347
79 -4.25 2.74 1.0000
25 -2 -3 13 319
-201 -2.80 -1.40 1.0000
23 -2 -3 21 259
-57 1.46 3.74 1.0000
23 -2 -3 21 258
-335 1.46 3.74 1.0000
21 -2 -3 142 250
247 -4.30 -2.78 1.0000
21 -2 -3 35 224
-70 -4.30 -2.78 1.0000
19 -2 -3 235 214
180 -3.23 2.67 1.0000
19 -2 -3 60 183
25 -3.23 2.67 1.0000
17 -2 -3 1755 262
1765 8.86 3.39 1.0000
17 -2 -3 1818 291
1830 8.86 3.39 1.0000
15 -2 -3 1933 248
1726 -3.92 -3.53 1.0000
15 -2 -3 1672 219
1477 -3.92 -3.53 1.0000
13 -2 -3 7650 466
8548 3.88 3.77 1.0000
13 -2 -3 8302 446
9237 3.88 3.77 1.0000
11 -2 -3 6949 362

6402	-11.24	8.33	1.0000	
11	-2	-3	7445	351
6887	-11.24	8.33	1.0000	
9	-2	-3	2405	276
5008	-41.39	-16.34	1.0000	
9	-2	-3	2294	274
4772	-41.39	-16.34	1.0000	
7	-2	-3	937	291
5646	-25.08	44.58	1.0000	
7	-2	-3	991	284
5837	-25.08	44.58	1.0000	
-7	2	3	960	279
5717	-25.08	-44.58	1.0000	
5	-2	-3	23990	531
12375	-37.14	-12.73	1.0000	
5	-2	-3	23310	514
11886	-37.14	-12.73	1.0000	
-5	2	3	23668	527
12167	-37.14	12.73	1.0000	
-5	2	3	23702	514
12191	-37.14	12.73	1.0000	
3	-2	-3	47853	1688
40662	-19.81	-5.67	1.0000	
3	-2	-3	49884	1671
42541	-19.81	-5.67	1.0000	
-3	2	3	49589	1688
42258	-19.81	5.67	1.0000	
-3	2	3	50331	1673
42945	-19.81	5.67	1.0000	
1	-2	-3	13709	784
18844	17.75	19.77	1.0000	
-1	2	3	14100	783
19310	17.75	-19.77	1.0000	
-1	-2	-3	18518	1118
28830	-14.53	-27.66	1.0000	
-1	-2	-3	17912	1127
28070	-14.53	-27.66	1.0000	
1	2	3	17216	1117
27196	-14.53	27.66	1.0000	
-3	-2	-3	46372	2612
68768	35.95	19.27	1.0000	
-3	-2	-3	43305	2603
65021	35.95	19.27	1.0000	
-3	-2	-3	42750	2602

64341	35.95	19.27	1.0000	
3	2	3	43658	2611
65449	35.95	-19.27	1.0000	
-5	-2	-3	11331	873
21616	-34.11	-8.85	1.0000	
-5	-2	-3	10462	854
20408	-34.11	-8.85	1.0000	
5	2	3	11228	876
21477	-34.11	8.85	1.0000	
5	2	3	10720	853
20773	-34.11	8.85	1.0000	
-7	-2	-3	57	134
801	-18.43	20.75	1.0000	
-7	-2	-3	66	93
731	-18.43	20.75	1.0000	
7	2	3	57	143
993	-18.43	-20.75	1.0000	
7	2	3	93	89
630	-18.43	-20.75	1.0000	
-9	-2	-3	1956	114
1147	-7.25	-10.99	1.0000	
9	2	3	1977	175
1161	-7.25	10.99	1.0000	
-11	-2	-3	303	114
827	-11.47	-0.39	1.0000	
11	2	3	456	177
1081	-11.47	0.39	1.0000	
-13	-2	-3	2340	106
546	15.93	15.26	1.0000	
13	2	3	2222	162
488	15.93	-15.26	1.0000	
-15	-2	-3	507	154
1096	12.79	-9.19	1.0000	
15	2	3	462	215
1012	12.79	9.19	1.0000	
-17	-2	-3	1556	187
1798	5.84	7.31	1.0000	
17	2	3	1657	311
1897	5.84	-7.31	1.0000	
-19	-2	-3	289	164
593	9.33	-1.49	1.0000	
19	2	3	426	260
796	9.33	1.49	1.0000	
21	2	3	278	246

228	-0.01	1.38	1.0000	
23	2	3	174	277
255	0.52	-2.55	1.0000	
25	2	3	8	366
-91	0.12	-2.52	1.0000	
26	-3	-3	3	345
-87	1.27	0.99	1.0000	
24	-3	-3	10	299
-28	2.71	0.37	1.0000	
22	-3	-3	89	232
199	3.64	3.18	1.0000	
22	-3	-3	7	263
37	3.64	3.18	1.0000	
20	-3	-3	755	261
921	0.81	-5.28	1.0000	
20	-3	-3	933	253
1120	0.81	-5.28	1.0000	
18	-3	-3	1748	261
1908	6.79	1.22	1.0000	
18	-3	-3	1163	259
1283	6.79	1.22	1.0000	
16	-3	-3	2183	293
3589	-9.66	6.14	1.0000	
16	-3	-3	1888	320
3208	-9.66	6.14	1.0000	
14	-3	-3	3938	211
1817	6.88	-17.61	1.0000	
14	-3	-3	3889	227
1783	6.88	-17.61	1.0000	
12	-3	-3	2170	265
3216	2.20	8.65	1.0000	
12	-3	-3	3194	267
4442	2.20	8.65	1.0000	
10	-3	-3	10656	514
11058	15.89	12.50	1.0000	
10	-3	-3	10825	523
11234	15.89	12.50	1.0000	
8	-3	-3	471	164
1960	34.27	-22.13	1.0000	
8	-3	-3	469	171
1929	34.27	-22.13	1.0000	
-8	3	3	482	160
1695	34.27	22.13	1.0000	
6	-3	-3	436	212

4007	32.96	20.69	1.0000	
6	-3	-3	373	223
3792	32.96	20.69	1.0000	
-6	3	3	400	210
3910	32.96	-20.69	1.0000	
4	-3	-3	44236	1841
45382	1.49	-2.77	1.0000	
4	-3	-3	45538	1828
46701	1.49	-2.77	1.0000	
-4	3	3	44210	1828
45364	1.49	2.77	1.0000	
2	-3	-3	43030	2576
64780	36.19	-19.15	1.0000	
2	-3	-3	42964	2590
64699	36.19	-19.15	1.0000	
-2	3	3	43998	2581
65971	36.19	19.15	1.0000	
0	-3	-3	14038	852
20843	-32.79	18.79	1.0000	
0	-3	-3	13752	840
20482	-32.79	18.79	1.0000	
0	3	3	14315	841
21198	-32.79	-18.79	1.0000	
-2	-3	-3	47031	1567
39858	-19.72	5.80	1.0000	
-2	-3	-3	47566	1564
40352	-19.72	5.80	1.0000	
2	3	3	44317	1563
37365	-19.72	-5.80	1.0000	
-4	-3	-3	1660	111
1635	7.98	-29.21	1.0000	
-4	-3	-3	1599	137
1537	7.98	-29.21	1.0000	
-4	-3	-3	1851	115
1928	7.98	-29.21	1.0000	
4	3	3	1546	144
1504	7.98	29.21	1.0000	
-6	-3	-3	2904	251
5126	-12.61	19.80	1.0000	
-6	-3	-3	3575	289
6053	-12.61	19.80	1.0000	
6	3	3	2821	251
5013	-12.61	-19.80	1.0000	
6	3	3	3340	294

5736 -12.61 -19.80 1.0000
-8 -3 -3 3865 102
910 21.92 -18.12 1.0000
8 3 3 3594 150
781 21.92 18.12 1.0000
-10 -3 -3 729 102
702 -0.24 -4.23 1.0000
10 3 3 398 140
373 -0.24 4.23 1.0000
-12 -3 -3 240 102
403 -6.89 16.75 1.0000
12 3 3 198 149
232 -6.89 -16.75 1.0000
-14 -3 -3 1442 184
2580 -10.71 -18.44 1.0000
14 3 3 1686 299
2900 -10.71 18.44 1.0000
-16 -3 -3 468 122
99 -10.18 4.26 1.0000
16 3 3 162 190
-81 -10.18 -4.26 1.0000
-18 -3 -3 545 134
176 -8.69 1.16 1.0000
18 3 3 530 224
168 -8.69 -1.16 1.0000
-20 -3 -3 404 159
490 -1.88 -0.16 1.0000
20 3 3 616 267
721 -1.88 0.16 1.0000
22 3 3 36 281
42 -1.76 0.21 1.0000
24 3 3 133 334
87 -3.68 1.39 1.0000
26 3 3 9 373
-42 2.03 -1.63 1.0000
25 -4 -3 133 315
117 -0.93 -0.46 1.0000
23 -4 -3 16 264
-15 -3.43 0.10 1.0000
23 -4 -3 16 252
-312 -3.43 0.10 1.0000
21 -4 -3 847 293
1294 -4.75 3.87 1.0000
21 -4 -3 741 263

1162 -4.75 3.87 1.0000
19 -4 -3 1017 250
737 -0.28 -7.13 1.0000
19 -4 -3 1412 227
1086 -0.28 -7.13 1.0000
17 -4 -3 626 178
670 -6.45 4.05 1.0000
17 -4 -3 413 213
433 -6.45 4.05 1.0000
15 -4 -3 6457 384
5567 5.22 0.72 1.0000
15 -4 -3 6754 358
5843 5.22 0.72 1.0000
13 -4 -3 4017 287
4401 -2.55 -0.59 1.0000
13 -4 -3 4159 310
4549 -2.55 -0.59 1.0000
11 -4 -3 2745 290
5118 -16.46 3.72 1.0000
11 -4 -3 2285 291
4481 -16.46 3.72 1.0000
9 -4 -3 8765 488
10702 -8.88 16.11 1.0000
9 -4 -3 8288 485
10164 -8.88 16.11 1.0000
7 -4 -3 18093 1292
32608 -37.04 -15.30 1.0000
7 -4 -3 17005 1294
31141 -37.04 -15.30 1.0000
-7 4 3 16771 1288
30819 -37.04 15.30 1.0000
5 -4 -3 1706 114
1125 -29.98 10.44 1.0000
5 -4 -3 1759 130
1204 -29.98 10.44 1.0000
-5 4 3 1717 128
1140 -29.98 -10.44 1.0000
3 -4 -3 1767 133
1851 7.98 28.97 1.0000
-3 4 3 1817 143
1874 7.98 -28.97 1.0000
1 -4 -3 22511 1318
32415 -13.10 -27.17 1.0000
1 -4 -3 23031 1312

33042	-13.10	-27.17	1.0000	
-1	-4	-3	26568	1260
31525	51.38	13.83	1.0000	
1	4	3	26323	1261
31193	51.38	-13.83	1.0000	
-3	-4	-3	44857	1806
46119	1.67	2.86	1.0000	
3	4	3	42788	1805
44014	1.67	-2.86	1.0000	
-5	-4	-3	812	131
2355	18.70	-18.45	1.0000	
-5	-4	-3	645	162
1988	18.70	-18.45	1.0000	
5	4	3	652	172
2001	18.70	18.45	1.0000	
-7	-4	-3	2052	107
1204	-2.89	11.14	1.0000	
7	4	3	1636	106
884	-2.89	-11.14	1.0000	
7	4	3	2351	168
1440	-2.89	-11.14	1.0000	
-9	-4	-3	2164	105
799	-17.09	1.29	1.0000	
9	4	3	2536	171
1029	-17.09	-1.29	1.0000	
-11	-4	-3	1433	142
1754	7.45	0.62	1.0000	
11	4	3	1553	241
1888	7.45	-0.62	1.0000	
-13	-4	-3	1170	134
1029	12.17	-3.48	1.0000	
13	4	3	1049	203
896	12.17	3.48	1.0000	
-15	-4	-3	435	152
1036	8.10	7.36	1.0000	
15	4	3	555	241
1223	8.10	-7.36	1.0000	
-17	-4	-3	1462	224
3082	17.64	-4.64	1.0000	
17	4	3	1477	386
3107	17.64	4.64	1.0000	
-19	-4	-3	876	163
712	2.31	1.04	1.0000	
19	4	3	929	286

760 2.31 -1.04 1.0000
-21 -4 -3 635 183
630 0.57 4.88 1.0000
21 4 3 863 312
865 0.57 -4.88 1.0000
23 4 3 213 317
359 3.64 1.18 1.0000
25 4 3 3 347
-80 -0.43 -1.33 1.0000
24 -5 -3 33 275
-395 4.66 -1.65 1.0000
24 -5 -3 14 282
83 4.66 -1.65 1.0000
22 -5 -3 400 276
577 4.73 -0.46 1.0000
22 -5 -3 310 252
467 4.73 -0.46 1.0000
20 -5 -3 635 217
400 3.41 2.94 1.0000
20 -5 -3 517 235
308 3.41 2.94 1.0000
18 -5 -3 210 194
286 1.50 -1.48 1.0000
18 -5 -3 8 202
27 1.50 -1.48 1.0000
16 -5 -3 2002 265
2501 1.64 -7.26 1.0000
16 -5 -3 2108 294
2621 1.64 -7.26 1.0000
10 -5 -3 7148 488
9889 6.15 -17.12 1.0000
10 -5 -3 7616 487
10446 6.15 -17.12 1.0000
8 -5 -3 804 157
1465 12.22 43.41 1.0000
8 -5 -3 809 162
1430 12.22 43.41 1.0000
6 -5 -3 2944 311
6536 20.72 -35.13 1.0000
-6 5 3 2608 313
5912 20.72 35.13 1.0000
4 -5 -3 853 163
2446 18.72 18.52 1.0000
-4 5 3 854 175

2451	18.72	-18.52	1.0000	
2	-5	-3	10102	828
19968	-34.26	8.98	1.0000	
0	-5	-3	3655	193
3405	-6.42	-34.42	1.0000	
0	5	3	3654	198
3385	-6.42	34.42	1.0000	
-2	-5	-3	23894	540
12274	-37.35	12.61	1.0000	
2	5	3	24588	546
12751	-37.35	-12.61	1.0000	
-4	-5	-3	1582	128
913	-30.08	-10.22	1.0000	
4	5	3	1472	120
733	-30.08	10.22	1.0000	
-6	-5	-3	1663	181
1871	-4.83	-4.99	1.0000	
-6	-5	-3	1998	135
2231	-4.83	-4.99	1.0000	
6	5	3	2072	193
2311	-4.83	4.99	1.0000	
-8	-5	-3	2053	113
1298	-11.88	1.76	1.0000	
8	5	3	1864	181
1150	-11.88	-1.76	1.0000	
-10	-5	-3	5558	315
6542	9.55	0.40	1.0000	
10	5	3	5997	387
7008	9.55	-0.40	1.0000	
-12	-5	-3	2036	220
3935	-6.52	-14.45	1.0000	
12	5	3	1987	308
3867	-6.52	14.45	1.0000	
-14	-5	-3	997	176
2296	-9.74	11.48	1.0000	
14	5	3	1110	314
2473	-9.74	-11.48	1.0000	
-16	-5	-3	2020	173
1396	-6.67	-1.26	1.0000	
16	5	3	1356	267
855	-6.67	1.26	1.0000	
-18	-5	-3	1207	195
1637	-10.48	-3.95	1.0000	
18	5	3	946	305

1316	-10.48	3.95	1.0000	
-20	-5	-3	2285	240
2471	-1.52	3.03	1.0000	
20	5	3	2381	403
2575	-1.52	-3.03	1.0000	
22	5	3	143	298
238	-2.81	3.82	1.0000	
24	5	3	199	330
149	-2.50	-0.90	1.0000	
25	-6	-3	604	338
569	-0.46	0.96	1.0000	
25	-6	-3	1	307
-118	-0.46	0.96	1.0000	
23	-6	-3	60	264
-452	-6.72	-0.53	1.0000	
23	-6	-3	60	257
-221	-6.72	-0.53	1.0000	
21	-6	-3	312	259
309	-2.09	-4.58	1.0000	
21	-6	-3	152	223
139	-2.09	-4.58	1.0000	
19	-6	-3	984	258
1087	-5.88	8.41	1.0000	
19	-6	-3	757	222
829	-5.88	8.41	1.0000	
17	-6	-3	1368	315
1977	-0.67	-7.91	1.0000	
17	-6	-3	1723	292
2404	-0.67	-7.91	1.0000	
15	-6	-3	967	201
994	0.16	-0.63	1.0000	
15	-6	-3	742	168
766	0.16	-0.63	1.0000	
13	-6	-3	1686	226
1685	-0.86	5.22	1.0000	
13	-6	-3	1773	218
1773	-0.86	5.22	1.0000	
11	-6	-3	1968	204
2429	6.32	0.69	1.0000	
11	-6	-3	1578	210
1990	6.32	0.69	1.0000	
9	-6	-3	910	163
859	-16.78	-0.71	1.0000	
9	-6	-3	895	142

840	-16.78	-0.71	1.0000	
7	-6	-3	5785	202
2897	-13.16	14.16	1.0000	
5	-6	-3	2013	171
2244	-5.06	5.07	1.0000	
-5	6	3	2181	188
2423	-5.06	-5.07	1.0000	
3	-6	-3	2165	231
4073	-12.38	-19.79	1.0000	
-3	6	3	2312	246
4284	-12.38	19.79	1.0000	
1	-6	-3	6382	299
6087	45.25	27.59	1.0000	
1	-6	-3	6570	314
6348	45.25	27.59	1.0000	
-1	-6	-3	3083	144
1321	16.05	-4.55	1.0000	
1	6	3	2814	145
1147	16.05	4.55	1.0000	
-3	-6	-3	267	218
3430	33.32	-20.39	1.0000	
3	6	3	279	217
3451	33.32	20.39	1.0000	
-5	-6	-3	2345	303
5400	20.82	35.09	1.0000	
-5	-6	-3	2408	258
5524	20.82	35.09	1.0000	
5	6	3	2596	306
5890	20.82	-35.09	1.0000	
-7	-6	-3	1737	287
6327	2.21	-35.73	1.0000	
7	6	3	1616	341
6089	2.21	35.73	1.0000	
-9	-6	-3	1715	123
1332	0.19	9.30	1.0000	
9	6	3	1662	195
1267	0.19	-9.30	1.0000	
-11	-6	-3	150	83
34	2.29	5.33	1.0000	
11	6	3	88	138
8	2.29	-5.33	1.0000	
-13	-6	-3	2120	168
2101	-3.63	-5.27	1.0000	
13	6	3	2207	284

2185 -3.63 5.27 1.0000
-15 -6 -3 3 118
368 17.38 2.60 1.0000
15 6 3 98 200
110 17.38 -2.60 1.0000
-17 -6 -3 1667 191
1706 3.16 -1.26 1.0000
17 6 3 2114 347
2162 3.16 1.26 1.0000
-19 -6 -3 1332 179
1051 2.90 2.05 1.0000
19 6 3 763 258
554 2.90 -2.05 1.0000
-21 -6 -3 313 173
179 4.52 -2.08 1.0000
21 6 3 599 275
411 4.52 2.08 1.0000
23 6 3 84 300
147 -1.80 -1.88 1.0000
25 6 3 2 308
-570 -1.02 -0.86 1.0000
24 -7 -3 12 299
-131 2.77 1.05 1.0000
24 -7 -3 12 280
-100 2.77 1.05 1.0000
22 -7 -3 141 248
318 5.30 0.32 1.0000
22 -7 -3 52 263
172 5.30 0.32 1.0000
20 -7 -3 125 230
102 6.52 -1.78 1.0000
20 -7 -3 202 220
193 6.52 -1.78 1.0000
18 -7 -3 751 214
615 -2.31 0.05 1.0000
18 -7 -3 515 225
404 -2.31 0.05 1.0000
16 -7 -3 52 190
153 5.11 1.26 1.0000
16 -7 -3 20 171
88 5.11 1.26 1.0000
14 -7 -3 1519 207
1075 -4.15 -4.10 1.0000
14 -7 -3 1462 181

1027 -4.15 -4.10 1.0000
10 -7 -3 715 114
209 12.09 21.52 1.0000
8 -7 -3 7559 383
7428 -0.30 -36.69 1.0000
6 -7 -3 1654 326
6198 2.29 35.92 1.0000
4 -7 -3 1939 143
1119 -2.69 -11.41 1.0000
-4 7 3 1747 167
971 -2.69 11.41 1.0000
2 -7 -3 133 113
509 -18.54 -20.87 1.0000
2 -7 -3 81 114
696 -18.54 -20.87 1.0000
0 -7 -3 10701 687
15597 -43.46 32.87 1.0000
0 7 3 11586 694
16826 -43.46 -32.87 1.0000
-2 -7 -3 729 262
4769 -25.14 -44.37 1.0000
2 7 3 786 269
5036 -25.14 44.37 1.0000
-4 -7 -3 11340 1084
23377 -37.36 15.35 1.0000
4 7 3 15295 1096
28935 -37.36 -15.35 1.0000
-6 -7 -3 5393 152
2612 -13.33 -14.10 1.0000
-6 -7 -3 5172 145
2459 -13.33 -14.10 1.0000
6 7 3 5051 215
2375 -13.33 14.10 1.0000
-8 -7 -3 5131 244
4907 3.59 -3.10 1.0000
8 7 3 5258 323
5033 3.59 3.10 1.0000
-10 -7 -3 790 135
1554 -10.39 3.48 1.0000
10 7 3 690 228
1415 -10.39 -3.48 1.0000
-12 -7 -3 558 100
272 12.18 -1.24 1.0000
12 7 3 200 148

-136 12.18 1.24 1.0000
-14 -7 -3 426 117
362 -3.75 -0.97 1.0000
14 7 3 173 183
127 -3.75 0.97 1.0000
-16 -7 -3 3590 222
2814 -7.92 -2.21 1.0000
16 7 3 3066 353
2345 -7.92 2.21 1.0000
-18 -7 -3 97 148
175 2.49 2.11 1.0000
18 7 3 14 228
-69 2.49 -2.11 1.0000
-20 -7 -3 188 169
236 -8.17 -3.85 1.0000
20 7 3 109 253
-8 -8.17 3.85 1.0000
22 7 3 126 274
173 -3.81 -3.91 1.0000
24 7 3 32 308
-162 4.19 2.52 1.0000
25 -8 -3 278 342
242 -1.63 0.73 1.0000
25 -8 -3 4 296
-51 -1.63 0.73 1.0000
23 -8 -3 504 309
555 -0.39 -0.90 1.0000
23 -8 -3 370 267
414 -0.39 -0.90 1.0000
21 -8 -3 456 275
831 -6.43 0.80 1.0000
21 -8 -3 353 257
689 -6.43 0.80 1.0000
19 -8 -3 149 202
35 -4.80 -5.21 1.0000
19 -8 -3 195 217
64 -4.80 -5.21 1.0000
17 -8 -3 78 187
17 -0.14 6.31 1.0000
17 -8 -3 158 204
87 -0.14 6.31 1.0000
15 -8 -3 110 173
74 -1.14 -2.08 1.0000
15 -8 -3 245 163

192	-1.14	-2.08	1.0000	
13	-8	-3	1983	290
4008	16.70	-1.66	1.0000	
13	-8	-3	2000	316
4033	16.70	-1.66	1.0000	
11	-8	-3	289	144
511	4.06	2.72	1.0000	
9	-8	-3	1269	176
1173	-1.29	1.50	1.0000	
7	-8	-3	4711	277
4509	3.71	3.07	1.0000	
5	-8	-3	2292	166
1493	-11.68	-1.98	1.0000	
3	-8	-3	3706	148
834	21.80	18.22	1.0000	
1	-8	-3	2154	247
3768	12.17	-12.63	1.0000	
1	-8	-3	2116	234
3716	12.17	-12.63	1.0000	
-1	-8	-3	2450	121
316	33.85	-1.50	1.0000	
1	8	3	2596	124
392	33.85	1.50	1.0000	
3	8	3	465	189
1754	34.39	-22.30	1.0000	
-5	-8	-3	760	119
1757	12.50	-43.54	1.0000	
-5	-8	-3	836	121
1251	12.50	-43.54	1.0000	
5	8	3	824	198
1367	12.50	43.54	1.0000	
-7	-8	-3	7369	324
7191	-0.31	36.79	1.0000	
-7	-8	-3	7418	327
7247	-0.31	36.79	1.0000	
7	8	3	7146	385
6964	-0.31	-36.79	1.0000	
-9	-8	-3	2532	211
4186	5.36	-13.69	1.0000	
-9	-8	-3	2302	214
3885	5.36	-13.69	1.0000	
9	8	3	2570	310
4241	5.36	13.69	1.0000	
-11	-8	-3	894	158

2201 -17.09 -10.13 1.0000
11 8 3 608 251
1695 -17.09 10.13 1.0000
-13 -8 -3 395 108
92 7.17 14.71 1.0000
13 8 3 336 193
15 7.17 -14.71 1.0000
-15 -8 -3 1221 183
2028 -3.46 -11.08 1.0000
15 8 3 1179 341
1976 -3.46 11.08 1.0000
-17 -8 -3 478 157
530 -0.82 4.85 1.0000
17 8 3 638 258
699 -0.82 -4.85 1.0000
-19 -8 -3 16 162
64 5.60 2.64 1.0000
19 8 3 89 240
224 5.60 -2.64 1.0000
-21 -8 -3 511 193
573 -0.13 -4.90 1.0000
21 8 3 368 279
418 -0.13 4.90 1.0000
23 8 3 12 273
-341 -2.66 -1.38 1.0000
25 8 3 121 331
202 -2.74 0.61 1.0000
24 -9 -3 537 333
498 0.77 -1.37 1.0000
24 -9 -3 272 295
244 0.77 -1.37 1.0000
22 -9 -3 209 264
255 2.30 3.53 1.0000
22 -9 -3 24 268
-59 2.30 3.53 1.0000
20 -9 -3 232 225
234 4.06 -0.77 1.0000
20 -9 -3 239 243
241 4.06 -0.77 1.0000
18 -9 -3 12 213
205 8.80 -3.48 1.0000
18 -9 -3 54 226
333 8.80 -3.48 1.0000
16 -9 -3 457 209

1120	-8.37	7.24	1.0000	
16	-9	-3	391	239
1014	-8.37	7.24	1.0000	
14	-9	-3	202	173
395	-12.06	-9.51	1.0000	
14	-9	-3	347	196
715	-12.06	-9.51	1.0000	
12	-9	-3	2410	280
3066	2.17	6.05	1.0000	
10	-9	-3	2371	294
4299	-25.13	-7.20	1.0000	
8	-9	-3	2363	267
3970	5.49	13.52	1.0000	
6	-9	-3	1867	180
1453	0.19	-9.30	1.0000	
4	-9	-3	2145	139
778	-17.18	-1.35	1.0000	
2	-9	-3	1717	168
944	-7.07	11.10	1.0000	
2	9	3	1961	266
4006	-41.39	-16.51	1.0000	
4	9	3	8085	486
9961	-9.01	15.73	1.0000	
-6	-9	-3	874	108
804	-16.99	0.78	1.0000	
-6	-9	-3	816	124
731	-16.99	0.78	1.0000	
6	9	3	881	175
822	-16.99	-0.78	1.0000	
-8	-9	-3	1042	115
967	-1.11	-1.44	1.0000	
-8	-9	-3	1081	124
1004	-1.11	-1.44	1.0000	
-10	-9	-3	252	150
1850	21.95	-10.22	1.0000	
10	9	3	193	249
1685	21.95	10.22	1.0000	
-12	-9	-3	937	147
1101	-3.36	8.09	1.0000	
12	9	3	1256	254
1477	-3.36	-8.09	1.0000	
-14	-9	-3	1023	138
661	9.90	-4.85	1.0000	
14	9	3	1180	248

797	9.90	4.85	1.0000	
-16	-9	-3	348	157
434	3.97	3.62	1.0000	
16	9	3	141	240
180	3.97	-3.62	1.0000	
-18	-9	-3	53	154
257	-7.58	-2.22	1.0000	
18	9	3	221	273
564	-7.58	2.22	1.0000	
-20	-9	-3	611	190
616	-0.24	0.46	1.0000	
20	9	3	0	281
-139	-0.24	-0.46	1.0000	
22	9	3	308	320
353	1.76	1.32	1.0000	
24	9	3	1	348
-148	0.02	0.91	1.0000	
23	-10	-3	31	292
47	0.09	-1.48	1.0000	
23	-10	-3	517	293
582	0.09	-1.48	1.0000	
21	-10	-3	260	271
356	-3.19	0.75	1.0000	
21	-10	-3	16	246
37	-3.19	0.75	1.0000	
19	-10	-3	12	210
-80	-2.79	1.25	1.0000	
19	-10	-3	223	247
303	-2.79	1.25	1.0000	
17	-10	-3	8	205
92	-4.25	-7.06	1.0000	
17	-10	-3	58	189
264	-4.25	-7.06	1.0000	
15	-10	-3	1448	204
1030	12.79	6.11	1.0000	
15	-10	-3	937	207
559	12.79	6.11	1.0000	
13	-10	-3	152	168
443	7.30	1.94	1.0000	
9	-10	-3	268	213
1923	22.15	10.30	1.0000	
7	-10	-3	925	199
1761	-10.55	-3.49	1.0000	
5	-10	-3	5570	363

6580 9.72 -0.28 1.0000
3 -10 -3 638 144
627 0.02 3.98 1.0000
1 -10 -3 1814 177
1151 11.71 -0.77 1.0000
1 10 3 817 124
193 21.62 -9.24 1.0000
3 10 3 9612 495
10028 16.19 12.43 1.0000
5 10 3 6249 463
8817 6.39 -17.24 1.0000
-7 -10 -3 706 86
131 12.02 -21.82 1.0000
7 10 3 703 151
167 12.02 21.82 1.0000
-9 -10 -3 1704 183
3250 -25.28 7.00 1.0000
-9 -10 -3 1349 186
2660 -25.28 7.00 1.0000
9 10 3 1626 265
3128 -25.28 -7.00 1.0000
-11 -10 -3 5709 348
7326 -0.10 11.32 1.0000
-11 -10 -3 5338 341
6903 -0.10 11.32 1.0000
11 10 3 5846 454
7488 -0.10 -11.32 1.0000
-13 -10 -3 3506 242
4150 -12.71 -12.83 1.0000
13 10 3 3297 382
3894 -12.71 12.83 1.0000
-15 -10 -3 1188 177
1562 -9.78 8.81 1.0000
15 10 3 1460 315
1900 -9.78 -8.81 1.0000
-17 -10 -3 417 154
164 6.22 -2.79 1.0000
17 10 3 62 254
-47 6.22 2.79 1.0000
-19 -10 -3 137 176
372 -3.34 -8.97 1.0000
19 10 3 80 285
246 -3.34 8.97 1.0000
-21 -10 -3 792 208

572	-3.13	8.10	1.0000	
21	10	3	275	301
117	-3.13	-8.10	1.0000	
23	10	3	4	332
-156	-1.33	1.23	1.0000	
22	-11	-3	66	277
72	1.38	-4.13	1.0000	
22	-11	-3	362	306
412	1.38	-4.13	1.0000	
20	-11	-3	8	231
-28	1.53	1.92	1.0000	
20	-11	-3	440	262
486	1.53	1.92	1.0000	
18	-11	-3	90	241
221	1.44	8.42	1.0000	
18	-11	-3	87	200
213	1.44	8.42	1.0000	
16	-11	-3	1465	288
1927	-1.18	-14.15	1.0000	
16	-11	-3	1499	306
1970	-1.18	-14.15	1.0000	
14	-11	-3	445	233
1166	-9.45	11.37	1.0000	
12	-11	-3	1050	242
1346	-16.84	-1.52	1.0000	
10	-11	-3	6247	451
7947	-0.08	-11.35	1.0000	
8	-11	-3	1138	243
2628	-17.17	10.21	1.0000	
6	-11	-3	239	121
71	2.65	-5.75	1.0000	
4	-11	-3	1440	203
1742	7.33	-0.47	1.0000	
2	-11	-3	269	163
767	-11.49	0.31	1.0000	
0	-11	-3	1240	232
1738	-5.23	-2.85	1.0000	
2	11	3	6113	343
5575	-11.48	8.47	1.0000	
4	11	3	1650	277
3590	-16.56	3.87	1.0000	
-6	-11	-3	1536	142
1940	6.44	-0.54	1.0000	
6	11	3	1159	227

1507 6.44 0.54 1.0000
-8 -11 -3 380 108
614 3.86 -2.57 1.0000
8 11 3 321 175
540 3.86 2.57 1.0000
-10 -11 -3 927 128
913 6.94 5.32 1.0000
10 11 3 877 216
862 6.94 -5.32 1.0000
-12 -11 -3 1428 111
105 21.12 -11.54 1.0000
12 11 3 1421 205
101 21.12 11.54 1.0000
-14 -11 -3 1456 175
1342 3.67 3.12 1.0000
14 11 3 1260 300
1152 3.67 -3.12 1.0000
-16 -11 -3 577 164
567 2.90 -0.24 1.0000
16 11 3 225 276
214 2.90 0.24 1.0000
-18 -11 -3 747 178
671 0.18 1.64 1.0000
18 11 3 694 312
622 0.18 -1.64 1.0000
-20 -11 -3 30 194
-90 -0.08 4.78 1.0000
20 11 3 98 297
135 -0.08 -4.78 1.0000
22 11 3 131 340
363 3.56 5.98 1.0000
23 -12 -3 54 286
51 -0.16 3.84 1.0000
23 -12 -3 298 327
320 -0.16 3.84 1.0000
21 -12 -3 356 288
491 2.18 -2.28 1.0000
21 -12 -3 188 263
288 2.18 -2.28 1.0000
19 -12 -3 374 250
272 -2.30 -1.96 1.0000
19 -12 -3 319 221
225 -2.30 -1.96 1.0000
17 -12 -3 64 189

```
-87      0.72     6.88  1.0000
  17 -12   -3       64       213
-70      0.72     6.88  1.0000
  15 -12   -3       91       204
321      4.35    -5.94  1.0000
  13 -12   -3      440       205
620      5.52    -1.93  1.0000
  11 -12   -3     1955       165
272     21.27    11.35  1.0000
   9 -12   -3     1366       244
1596    -3.31    -8.24  1.0000
   7 -12   -3      437       135
167     12.38     1.30  1.0000
   5 -12   -3     1912       283
3798    -6.71    14.68  1.0000
   3 -12   -3      210       136
149     -6.92   -16.88  1.0000
   1 -12   -3     2160       283
3430    -0.47    11.94  1.0000
   3  12    3     1245       240
2063     2.48     8.54  1.0000
   5  12    3      850       189
984      6.83    -9.05  1.0000
  -7 -12   -3     2544       132
1181   -16.88     0.85  1.0000
   7  12    3     2438       227
1115   -16.88    -0.85  1.0000
  -9 -12   -3     1780       170
2366     2.29    -6.23  1.0000
   9  12    3     1539       292
2089     2.29     6.23  1.0000
 -11 -12   -3      858       152
1085   -16.82     1.43  1.0000
  11  12    3      862       248
1096   -16.82    -1.43  1.0000
 -13 -12   -3     1442       161
1243   -14.53     5.18  1.0000
  13  12    3      976       236
748    -14.53    -5.18  1.0000
 -15 -12   -3     1488       193
1681     2.71    -1.01  1.0000
  15  12    3     1392       313
1579     2.71     1.01  1.0000
 -17 -12   -3      114       169
```

-172	-8.90	-2.58	1.0000	
17	12	3	227	283
106	-8.90	2.58	1.0000	
-19	-12	-3	18	187
35	0.14	3.02	1.0000	
19	12	3	166	310
229	0.14	-3.02	1.0000	
21	12	3	56	341
207	-2.73	5.34	1.0000	
23	12	3	12	363
-126	-2.90	-0.70	1.0000	
22	-13	-3	747	307
775	0.07	1.06	1.0000	
22	-13	-3	423	313
444	0.07	1.06	1.0000	
20	-13	-3	314	259
332	1.67	0.55	1.0000	
20	-13	-3	368	269
387	1.67	0.55	1.0000	
18	-13	-3	4	221
59	-5.17	-6.56	1.0000	
18	-13	-3	4	224
132	-5.17	-6.56	1.0000	
16	-13	-3	268	234
535	-0.55	10.26	1.0000	
14	-13	-3	1140	242
1265	0.19	-6.24	1.0000	
12	-13	-3	843	214
603	-14.56	-5.14	1.0000	
10	-13	-3	3528	336
4167	-12.85	13.06	1.0000	
8	-13	-3	618	169
449	7.22	-14.67	1.0000	
6	-13	-3	2388	254
2381	-3.81	5.33	1.0000	
4	-13	-3	1139	185
994	12.33	3.45	1.0000	
2	-13	-3	2506	178
623	15.98	-15.30	1.0000	
0	-13	-3	363	195
539	-5.53	8.53	1.0000	
2	13	3	5028	356
5797	4.11	3.76	1.0000	
4	13	3	3020	294

3350 -2.48 -0.68 1.0000
-6 -13 -3 1177 140
1184 -0.66 -5.37 1.0000
6 13 3 1203 220
1215 -0.66 5.37 1.0000
-8 -13 -3 1908 223
3897 16.76 1.59 1.0000
8 13 3 1952 364
3968 16.76 -1.59 1.0000
-10 -13 -3 203 123
513 7.03 -1.93 1.0000
10 13 3 125 203
384 7.03 1.93 1.0000
-12 -13 -3 858 155
1122 5.62 1.98 1.0000
12 13 3 416 256
594 5.62 -1.98 1.0000
-14 -13 -3 676 191
1466 11.01 0.94 1.0000
14 13 3 546 276
1267 11.01 -0.94 1.0000
-16 -13 -3 1388 174
1084 -2.23 -4.39 1.0000
16 13 3 912 262
668 -2.23 4.39 1.0000
-18 -13 -3 428 179
305 3.31 5.33 1.0000
18 13 3 333 293
223 3.31 -5.33 1.0000
-20 -13 -3 79 214
159 3.75 -5.16 1.0000
20 13 3 85 309
170 3.75 5.16 1.0000
22 13 3 57 385
208 0.24 -6.15 1.0000
21 -14 -3 345 303
477 -0.89 5.41 1.0000
21 -14 -3 266 293
379 -0.89 5.41 1.0000
19 -14 -3 20 234
-31 3.88 0.15 1.0000
19 -14 -3 179 246
318 3.88 0.15 1.0000
17 -14 -3 593 246

576 4.04 -4.61 1.0000
15 -14 -3 89 217
265 -6.07 5.86 1.0000
13 -14 -3 820 298
1674 11.04 -1.07 1.0000
11 -14 -3 835 217
734 3.80 -3.09 1.0000
9 -14 -3 992 196
625 9.92 4.68 1.0000
7 -14 -3 453 175
382 -3.76 1.10 1.0000
5 -14 -3 956 274
2230 -9.52 -11.69 1.0000
3 -14 -3 1395 276
2465 -10.67 18.51 1.0000
1 -14 -3 67 160
57 -4.26 -2.94 1.0000
3 14 3 3093 224
1246 6.97 -17.60 1.0000
5 14 3 1306 204
389 -2.95 13.98 1.0000
-7 -14 -3 1322 132
909 -4.19 4.10 1.0000
-9 -14 -3 232 125
467 -11.99 9.30 1.0000
9 14 3 224 226
451 -11.99 -9.30 1.0000
-11 -14 -3 495 163
1252 -9.45 -11.37 1.0000
11 14 3 300 282
895 -9.45 11.37 1.0000
-13 -14 -3 1541 188
1707 0.26 6.25 1.0000
13 14 3 1719 329
1890 0.26 -6.25 1.0000
-15 -14 -3 87 150
-95 -7.50 -3.03 1.0000
15 14 3 87 274
-78 -7.50 3.03 1.0000
-17 -14 -3 694 189
608 -1.54 -0.98 1.0000
17 14 3 498 258
425 -1.54 0.98 1.0000
-19 -14 -3 339 197

486 -4.10 1.70 1.0000
19 14 3 11 245
20 -4.10 -1.70 1.0000
21 14 3 237 328
213 -0.55 -0.65 1.0000
20 -15 -3 324 297
569 -4.38 -2.91 1.0000
20 -15 -3 213 276
418 -4.38 -2.91 1.0000
18 -15 -3 289 248
283 2.73 1.58 1.0000
16 -15 -3 25 208
20 -3.36 -2.85 1.0000
14 -15 -3 86 208
-54 -7.46 3.04 1.0000
12 -15 -3 1688 309
1912 2.70 1.23 1.0000
10 -15 -3 1056 259
1409 -9.84 -8.70 1.0000
8 -15 -3 1134 290
1912 -3.53 11.07 1.0000
6 -15 -3 21 204
581 17.28 -2.38 1.0000
4 -15 -3 544 224
1203 8.08 -7.27 1.0000
2 -15 -3 459 234
993 12.60 9.16 1.0000
0 -15 -3 976 254
1371 15.08 -10.78 1.0000
4 15 3 5179 359
4363 5.34 0.80 1.0000
-6 -15 -3 919 139
949 0.21 0.64 1.0000
6 15 3 722 223
748 0.21 -0.64 1.0000
8 15 3 283 205
223 -1.12 -2.19 1.0000
-10 -15 -3 1213 145
821 12.95 -6.25 1.0000
10 15 3 1252 266
849 12.95 6.25 1.0000
-12 -15 -3 101 135
336 4.22 5.93 1.0000
12 15 3 9 252

30	4.22	-5.93	1.0000	
-14	-15	-3	142	154
363	-6.07	-5.80	1.0000	
14	15	3	252	267
542	-6.07	5.80	1.0000	
-16	-15	-3	126	173
572	7.92	7.98	1.0000	
16	15	3	179	309
683	7.92	-7.98	1.0000	
-18	-15	-3	22	195
-60	2.46	-3.22	1.0000	
18	15	3	201	304
105	2.46	3.22	1.0000	
20	15	3	4	265
-193	-0.05	-1.78	1.0000	
19	-16	-3	442	282
509	-0.04	5.35	1.0000	
17	-16	-3	114	244
119	-1.66	2.89	1.0000	
15	-16	-3	156	240
634	7.95	-7.89	1.0000	
13	-16	-3	756	227
530	-2.29	4.47	1.0000	
11	-16	-3	157	207
141	2.71	0.03	1.0000	
9	-16	-3	115	201
151	4.05	-3.37	1.0000	
7	-16	-3	3917	337
3113	-7.89	2.12	1.0000	
5	-16	-3	1607	240
1056	-6.69	1.27	1.0000	
3	-16	-3	350	191
49	-10.08	-4.18	1.0000	
1	-16	-3	535	209
192	-13.59	1.88	1.0000	
-1	-16	-3	545	232
416	4.02	6.87	1.0000	
3	16	3	1520	307
2729	-9.71	6.17	1.0000	
5	16	3	2099	326
2597	1.80	-7.23	1.0000	
7	16	3	40	201
-25	5.29	1.53	1.0000	
9	16	3	237	250

759   -8.48   7.31 1.0000
  11   16   3   1543   334
2016   -1.12   -14.10 1.0000
  13   16   3   178   281
378   -0.53   10.15 1.0000
 -15 -16   -3   296   173
355   -3.30   2.92 1.0000
  15   16   3   271   264
327   -3.30   -2.92 1.0000
 -17 -16   -3   11   173
-45   -1.21   2.54 1.0000
  17   16   3   178   303
188   -1.21   -2.54 1.0000
 -19 -16   -3   9   212
54   -2.63   -2.80 1.0000
  19   16   3   228   326
381   -2.63   2.80 1.0000
  20 -17   -3   1   268
-192   0.05   -0.95 1.0000
  20 -17   -3   1   279
-59   0.05   -0.95 1.0000
  18 -17   -3   8   277
37   -2.59   -1.65 1.0000
  16 -17   -3   29   221
26   -1.18   -2.54 1.0000
  14 -17   -3   448   223
378   -1.59   0.98 1.0000
  12 -17   -3   375   215
245   -8.87   2.72 1.0000
  10 -17   -3   246   212
63   6.31   2.82 1.0000
   8 -17   -3   474   223
518   -0.87   -4.78 1.0000
   6 -17   -3   1536   278
1571   3.19   1.31 1.0000
   4 -17   -3   1346   345
2886   17.45   4.67 1.0000
   2 -17   -3   1278   282
1482   5.95   -7.42 1.0000
   0 -17   -3   733   264
1067   4.63   6.09 1.0000
  -2 -17   -3   1647   324
1658   8.76   -3.43 1.0000
   4   17   3   578   218

594 -6.11 4.26 1.0000
6 17 3 1062 298
1604 -0.73 -7.96 1.0000
8 17 3 100 215
34 -0.03 6.38 1.0000
10 17 3 249 257
614 -4.22 -7.17 1.0000
12 17 3 64 241
-133 0.63 6.88 1.0000
14 17 3 501 298
484 4.01 -4.68 1.0000
16 17 3 14 290
-173 -1.59 2.81 1.0000
18 17 3 24 288
58 0.76 3.03 1.0000
20 17 3 37 347
200 3.80 -5.88 1.0000
19 -18 -3 30 276
-175 1.19 -4.64 1.0000
17 -18 -3 13 254
-93 0.93 3.01 1.0000
15 -18 -3 258 242
150 2.36 3.35 1.0000
13 -18 -3 448 236
329 3.38 -5.28 1.0000
11 -18 -3 700 241
639 0.08 -1.45 1.0000
9 -18 -3 142 226
435 -7.71 2.13 1.0000
7 -18 -3 14 202
-199 2.45 -2.07 1.0000
5 -18 -3 1313 295
1760 -10.36 3.89 1.0000
3 -18 -3 390 231
96 -8.64 -0.98 1.0000
1 -18 -3 1362 296
1445 1.40 -4.13 1.0000
-1 -18 -3 174 230
-209 -10.17 5.23 1.0000
5 18 3 6 205
-91 1.48 -1.55 1.0000
7 18 3 588 262
466 -2.35 0.12 1.0000
9 18 3 19 244

235 8.90 -3.55 1.0000
11 18 3 104 253
246 1.37 8.38 1.0000
13 18 3 16 283
183 -5.14 -6.56 1.0000
15 18 3 184 307
178 2.63 1.61 1.0000
17 18 3 125 326
211 -2.47 -1.68 1.0000
19 18 3 78 348
114 -0.09 -2.53 1.0000
18 -19 -3 8 279
-59 -0.13 -2.42 1.0000
16 -19 -3 20 248
-82 -2.71 2.74 1.0000
14 -19 -3 30 236
69 -4.19 -1.62 1.0000
12 -19 -3 176 232
244 0.12 -3.15 1.0000
10 -19 -3 139 241
369 -3.30 8.95 1.0000
8 -19 -3 50 218
-57 5.58 -2.49 1.0000
6 -19 -3 1335 273
1051 2.94 -2.05 1.0000
4 -19 -3 1060 266
883 2.25 -1.05 1.0000
2 -19 -3 560 273
972 9.20 1.38 1.0000
0 -19 -3 400 264
435 1.17 -2.22 1.0000
-2 -19 -3 23 259
-198 -3.13 -2.73 1.0000
6 19 3 467 259
496 -5.74 8.44 1.0000
8 19 3 66 246
-233 -4.77 -5.18 1.0000
10 19 3 23 249
48 -2.82 1.35 1.0000
12 19 3 12 253
-121 -2.23 -1.96 1.0000
14 19 3 115 277
234 3.98 -0.01 1.0000
16 19 3 463 335

534	-0.12	5.24	1.0000	
18	19	3	30	346
-162	1.18	-4.62	1.0000	
17	-20	-3	1	281
83	3.71	-5.87	1.0000	
15	-20	-3	4	257
-200	0.01	-1.73	1.0000	
13	-20	-3	47	246
98	3.71	5.10	1.0000	
11	-20	-3	48	249
63	-0.10	-4.64	1.0000	
9	-20	-3	728	261
733	-0.22	-0.42	1.0000	
7	-20	-3	122	234
128	-8.17	3.88	1.0000	
5	-20	-3	1959	365
2146	-1.62	-3.08	1.0000	
3	-20	-3	606	270
699	-1.72	0.03	1.0000	
1	-20	-3	589	280
383	-6.57	6.68	1.0000	
-1	-20	-3	743	263
252	5.85	-8.18	1.0000	
-3	-20	-3	922	332
1108	0.89	5.39	1.0000	
5	20	3	277	237
127	3.51	3.06	1.0000	
7	20	3	64	247
-201	6.68	-1.79	1.0000	
9	20	3	81	279
74	3.95	-0.83	1.0000	
11	20	3	169	261
197	1.57	1.74	1.0000	
13	20	3	417	294
435	1.54	0.55	1.0000	
15	20	3	150	307
329	-4.43	-2.91	1.0000	
17	20	3	166	344
193	0.11	-1.05	1.0000	
14	-21	-3	1	263
-163	-0.58	-0.60	1.0000	
12	-21	-3	49	261
-102	-2.72	5.40	1.0000	
10	-21	-3	476	253

283 -3.22 -8.12 1.0000
8 -21 -3 533 260
600 -0.07 4.80 1.0000
6 -21 -3 32 244
-20 4.46 2.11 1.0000
4 -21 -3 361 265
343 0.74 -4.83 1.0000
2 -21 -3 477 277
410 -0.17 1.37 1.0000
0 -21 -3 79 277
244 2.70 -5.29 1.0000
-2 -21 -3 16 274
13 -4.20 2.76 1.0000
-4 -21 -3 618 351
1009 -4.80 -3.85 1.0000
6 21 3 133 254
119 -2.11 -4.56 1.0000
8 21 3 97 281
301 -6.45 0.78 1.0000
10 21 3 14 277
-200 -3.19 0.69 1.0000
12 21 3 328 298
461 2.23 -2.33 1.0000
14 21 3 195 310
287 -0.88 5.37 1.0000
13 -22 -3 50 276
-51 0.15 -6.11 1.0000
11 -22 -3 110 277
327 3.67 5.90 1.0000
9 -22 -3 655 282
720 1.73 1.34 1.0000
7 -22 -3 94 238
128 -3.86 -3.89 1.0000
5 -22 -3 18 247
38 -2.75 3.86 1.0000
3 -22 -3 232 270
252 -1.80 0.16 1.0000
1 -22 -3 44 281
185 0.86 -6.12 1.0000
-1 -22 -3 189 305
464 1.62 7.21 1.0000
-3 -22 -3 31 298
-43 3.66 -3.18 1.0000
7 22 3 96 267

```
244     5.20     0.20  1.0000
   9  22   3        25       285
-182     2.42     3.57  1.0000
  11  22   3       418       321
472     1.37    -4.04  1.0000
  13  22   3       921       363
949     0.03     1.05  1.0000
  12 -23  -3        63       295
129    -2.92    -0.71  1.0000
  10 -23  -3       242       274
206    -1.38     1.42  1.0000
   8 -23  -3       318       293
271    -2.59    -1.48  1.0000
   6 -23  -3       130       266
205    -1.73    -1.87  1.0000
   4 -23  -3       291       291
449     3.49     1.05  1.0000
   2 -23  -3       329       314
441     0.72    -2.52  1.0000
   0 -23  -3       128       303
253    -3.70     1.87  1.0000
  -2 -23  -3        22       301
-34     1.37    -3.84  1.0000
  -4 -23  -3        16       313
-407    -3.44    -0.16  1.0000
   8  23   3       251       280
286    -0.34    -0.89  1.0000
  10  23   3       353       340
407     0.13    -1.50  1.0000
  12  23   3       489       358
514    -0.15     3.69  1.0000
   9 -24  -3       229       293
207     0.05     0.93  1.0000
   7 -24  -3        32       258
-237     4.19     2.53  1.0000
   5 -24  -3        10       273
-175    -2.57    -0.88  1.0000
   3 -24  -3       159       283
112    -3.70     1.49  1.0000
   1 -24  -3       147       290
220     3.44    -0.22  1.0000
  -1 -24  -3        56       312
134    -1.10    -3.72  1.0000
  -3 -24  -3       336       331
```

456 2.86 -0.23 1.0000
-5 -24 -3 33 341
-416 4.67 1.70 1.0000
9 24 3 170 297
147 0.78 -1.40 1.0000
8 -25 -3 322 335
448 -2.76 0.65 1.0000
6 -25 -3 2 300
-74 -1.00 -0.90 1.0000
4 -25 -3 148 321
167 -0.34 -1.41 1.0000
2 -25 -3 133 327
208 -0.01 -2.70 1.0000
0 -25 -3 34 342
73 2.77 -0.20 1.0000
-2 -25 -3 13 334
-266 -2.83 1.39 1.0000
-4 -25 -3 245 350
224 -0.86 0.36 1.0000
-6 -25 -3 1 378
-359 -0.50 -0.88 1.0000
3 -26 -3 256 348
358 2.02 -1.56 1.0000
1 -26 -3 36 338
-313 -4.29 2.90 1.0000
-1 -26 -3 45 366
117 2.65 -2.36 1.0000
-3 -26 -3 3 347
-329 1.25 -0.98 1.0000
5 25 -4 86 313
82 0.54 1.24 1.0000
-5 -25 4 2 352
-148 0.54 -1.24 1.0000
3 25 -4 65 287
65 -1.56 0.50 1.0000
-3 -25 4 246 342
250 -1.56 -0.50 1.0000
1 25 -4 130 289
265 3.53 -3.39 1.0000
-1 -25 4 33 323
107 3.53 3.39 1.0000
1 -25 4 119 303
52 -3.05 -1.40 1.0000
3 -25 4 8 296

-101	-1.11	2.16	1.0000	
5	-25	4	1	287
-244	-0.90	0.50	1.0000	
8	24	-4	208	356
260	-0.18	-1.59	1.0000	
-8	-24	4	382	402
452	-0.18	1.59	1.0000	
6	24	-4	137	302
237	2.48	-2.79	1.0000	
-6	-24	4	530	381
720	2.48	2.79	1.0000	
4	24	-4	14	283
-7	2.11	-2.44	1.0000	
-4	-24	4	68	320
114	2.11	2.44	1.0000	
2	24	-4	191	245
191	0.30	-1.21	1.0000	
-2	-24	4	2	314
-113	0.30	1.21	1.0000	
0	24	-4	54	234
62	-0.45	-0.31	1.0000	
0	-24	4	0	296
-180	-0.45	0.31	1.0000	
2	-24	4	444	308
534	2.64	-1.17	1.0000	
4	-24	4	176	296
143	0.66	-1.27	1.0000	
6	-24	4	415	309
549	-0.33	2.65	1.0000	
8	-24	4	40	288
37	-0.81	-1.19	1.0000	
11	23	-4	106	306
123	-0.65	0.17	1.0000	
11	23	-4	148	386
167	-0.65	0.17	1.0000	
9	23	-4	456	329
375	1.72	-0.16	1.0000	
7	23	-4	137	291
158	0.34	2.27	1.0000	
-7	-23	4	18	346
21	0.34	-2.27	1.0000	
5	23	-4	127	275
338	-4.93	5.02	1.0000	
-5	-23	4	41	317

175 -4.93 -5.02 1.0000
3 23 -4 41 231
74 5.30 -0.80 1.0000
-3 -23 4 38 293
-269 5.30 0.80 1.0000
1 23 -4 222 244
569 -6.73 3.91 1.0000
-1 -23 4 138 305
427 -6.73 -3.91 1.0000
-1 23 -4 47 206
-51 3.14 -5.04 1.0000
1 -23 4 153 291
368 3.14 5.04 1.0000
-3 23 -4 6 201
-183 -1.51 -1.43 1.0000
3 -23 4 6 254
-138 -1.51 1.43 1.0000
5 -23 4 28 255
-282 -4.43 -1.18 1.0000
7 -23 4 2 268
4 0.94 2.25 1.0000
9 -23 4 418 308
692 2.71 -4.83 1.0000
11 -23 4 294 285
332 0.39 0.85 1.0000
12 22 -4 806 409
1173 5.20 -0.62 1.0000
12 22 -4 295 327
530 5.20 -0.62 1.0000
10 22 -4 245 321
264 -2.73 -1.47 1.0000
10 22 -4 237 285
255 -2.73 -1.47 1.0000
8 22 -4 150 283
305 3.80 -2.54 1.0000
6 22 -4 169 249
204 1.70 -1.69 1.0000
-6 -22 4 190 337
229 1.70 1.69 1.0000
4 22 -4 8 220
-57 0.19 -2.50 1.0000
-4 -22 4 8 294
-44 0.19 2.50 1.0000
2 22 -4 32 197

-29	2.62	-4.14	1.0000		
-2	-22	4	233	292	
436	2.62	4.14	1.0000		
0	22	-4	2	202	
23	4.44	0.51	1.0000		
0	-22	4	153	269	
298	4.44	-0.51	1.0000		
-2	22	-4	18	197	
-122	-3.46	-1.33	1.0000		
2	-22	4	18	262	
-194	-3.46	1.33	1.0000		
-4	22	-4	364	211	
378	2.84	1.68	1.0000		
4	-22	4	472	267	
491	2.84	-1.68	1.0000		
6	-22	4	159	261	
238	2.70	-5.12	1.0000		
8	-22	4	346	262	
102	-7.34	4.39	1.0000		
10	-22	4	122	286	
224	2.69	-2.11	1.0000		
12	-22	4	36	257	
-330	-5.12	0.75	1.0000		
15	21	-4	95	328	
147	-1.20	-1.97	1.0000		
15	21	-4	373	433	
472	-1.20	-1.97	1.0000		
13	21	-4	170	322	
399	1.43	-6.06	1.0000		
13	21	-4	582	400	
967	1.43	-6.06	1.0000		
11	21	-4	3	315	
60	-0.19	6.26	1.0000		
11	21	-4	198	308	
440	-0.19	6.26	1.0000		
9	21	-4	510	276	
463	-0.95	1.15	1.0000		
9	21	-4	192	258	
163	-0.95	1.15	1.0000		
7	21	-4	152	229	
186	-4.35	5.74	1.0000		
5	21	-4	11	219	
70	6.20	0.74	1.0000		
-5	-21	4	105	310	

```
264      6.20    -0.74  1.0000
   3  21  -4        85       206
270    -7.49     2.49  1.0000
  -3 -21   4        17       275
80    -7.49    -2.49  1.0000
   1  21  -4       155       197
138      3.73     0.65  1.0000
  -1 -21   4        19       253
-235      3.73    -0.65  1.0000
  -1  21  -4       331       204
376    -4.25    -1.31  1.0000
   1 -21   4       656       291
730    -4.25     1.31  1.0000
  -3  21  -4       254       204
198    -1.36    -1.38  1.0000
   3 -21   4         5       253
-64    -1.36     1.38  1.0000
   5 -21   4        26       242
16      0.69     4.43  1.0000
   7 -21   4       187       241
228    -2.73    -1.69  1.0000
   9 -21   4        16       251
-110      0.13     3.47  1.0000
  11 -21   4       270       257
270      2.86    -1.25  1.0000
  13 -21   4        96       282
336      4.15    -6.19  1.0000
  15 -21   4        92       294
246      0.32     5.89  1.0000
  16  20  -4      1009       444
904      0.76     2.35  1.0000
  16  20  -4       701       340
613      0.76     2.35  1.0000
  14  20  -4       532       378
453      2.52     0.10  1.0000
  14  20  -4       105       281
69      2.52     0.10  1.0000
  12  20  -4       437       314
779    -5.18     3.42  1.0000
  12  20  -4       328       339
630    -5.18     3.42  1.0000
  10  20  -4       690       285
307      5.62    -5.11  1.0000
  10  20  -4       881       284
```

438 5.62 -5.11 1.0000
8 20 -4 179 230
134 -0.42 -2.37 1.0000
8 20 -4 233 247
182 -0.42 -2.37 1.0000
6 20 -4 147 195
123 2.77 -3.77 1.0000
4 20 -4 305 201
331 -0.03 -1.99 1.0000
-4 -20 4 683 295
723 -0.03 1.99 1.0000
2 20 -4 284 190
242 0.64 0.93 1.0000
-2 -20 4 48 266
32 0.64 -0.93 1.0000
0 20 -4 626 207
440 0.94 -3.68 1.0000
0 -20 4 370 264
230 0.94 3.68 1.0000
-2 20 -4 1431 316
2149 4.26 6.06 1.0000
2 -20 4 1134 323
1781 4.26 -6.06 1.0000
-4 20 -4 376 211
422 -1.53 -0.14 1.0000
4 -20 4 459 259
509 -1.53 0.14 1.0000
6 -20 4 740 267
912 -2.71 0.04 1.0000
8 -20 4 98 247
466 4.59 -9.03 1.0000
10 -20 4 220 255
561 -7.69 0.98 1.0000
12 -20 4 11 255
88 -3.59 4.18 1.0000
14 -20 4 26 256
25 0.59 -0.76 1.0000
16 -20 4 55 260
-216 -4.91 4.18 1.0000
17 19 -4 750 461
835 0.34 -1.54 1.0000
17 19 -4 391 315
453 0.34 -1.54 1.0000
15 19 -4 548 393

842	-3.22	-3.83	1.0000		
15	19	-4	94	295	
232	-3.22	-3.83	1.0000		
13	19	-4	647	345	
743	-0.53	7.26	1.0000		
13	19	-4	791	307	
904	-0.53	7.26	1.0000		
11	19	-4	104	261	
-166	2.91	-8.33	1.0000		
11	19	-4	361	285	
332	2.91	-8.33	1.0000		
9	19	-4	53	259	
16	-3.34	8.83	1.0000		
9	19	-4	126	251	
428	-3.34	8.83	1.0000		
7	19	-4	933	255	
1150	1.81	2.60	1.0000		
7	19	-4	782	274	
982	1.81	2.60	1.0000		
5	19	-4	976	222	
758	-3.69	0.30	1.0000		
-5	-19	4	1068	313	
839	-3.69	-0.30	1.0000		
3	19	-4	762	187	
277	4.38	8.44	1.0000		
-3	-19	4	697	266	
238	4.38	-8.44	1.0000		
1	19	-4	26	176	
223	-3.62	-7.75	1.0000		
-1	-19	4	91	251	
377	-3.62	7.75	1.0000		
-1	19	-4	1489	287	
1611	2.16	-1.25	1.0000		
1	-19	4	1635	313	
1765	2.16	1.25	1.0000		
-3	19	-4	680	203	
476	-2.17	-3.72	1.0000		
3	-19	4	172	237	
76	-2.17	3.72	1.0000		
-5	19	-4	747	218	
954	-0.72	-3.75	1.0000		
5	-19	4	589	253	
772	-0.72	3.75	1.0000		
7	-19	4	178	237	

```
210      0.20      3.12  1.0000
   9 -19    4       850       265
780    -1.17      1.66  1.0000
  11 -19    4       363       243
260      3.79    -1.91  1.0000
  13 -19    4       232       240
141      2.46      3.78  1.0000
  15 -19    4       112       272
378    -0.84    -7.78  1.0000
  17 -19    4        49       286
77      1.87      1.55  1.0000
  18   18   -4       596       466
910    -3.65      3.52  1.0000
  18   18   -4       122       352
281    -3.65      3.52  1.0000
  16   18   -4       590       401
603      2.11      0.51  1.0000
  16   18   -4        74       298
75      2.11      0.51  1.0000
  14   18   -4       664       365
825      1.95      2.20  1.0000
  14   18   -4       215       270
309      1.95      2.20  1.0000
  12   18   -4        60       254
12      1.49    -4.61  1.0000
  12   18   -4       593       287
460      1.49    -4.61  1.0000
  10   18   -4       120       257
44    -2.92      2.61  1.0000
  10   18   -4       369       241
223    -2.92      2.61  1.0000
   8   18   -4       428       231
561      3.53    -0.90  1.0000
   8   18   -4       205       256
297      3.53    -0.90  1.0000
   6   18   -4        71       219
-21    -7.09    -1.79  1.0000
   6   18   -4       217       192
83    -7.09    -1.79  1.0000
   4   18   -4       155       186
186      2.79    -0.94  1.0000
  -4 -18    4       256       277
298      2.79      0.94  1.0000
   2   18   -4       890       200
```

977 0.75 -1.19 1.0000
-2 -18 4 988 288
1079 0.75 1.19 1.0000
0 18 -4 816 197
753 -4.00 7.60 1.0000
0 -18 4 504 260
432 -4.00 -7.60 1.0000
-2 18 -4 24 167
50 2.87 0.66 1.0000
2 -18 4 20 219
45 2.87 -0.66 1.0000
-4 18 -4 309 172
158 3.20 8.73 1.0000
4 -18 4 115 218
-13 3.20 -8.73 1.0000
-6 18 -4 1408 276
1437 -3.57 4.48 1.0000
6 -18 4 1041 259
1062 -3.57 -4.48 1.0000
8 -18 4 152 217
154 0.19 0.28 1.0000
10 -18 4 274 242
422 -3.73 -2.39 1.0000
12 -18 4 14 215
-15 -2.97 -1.35 1.0000
14 -18 4 75 237
92 -5.41 4.26 1.0000
16 -18 4 107 260
208 4.59 1.64 1.0000
18 -18 4 73 307
201 -3.55 3.55 1.0000
19 17 -4 135 360
182 1.92 1.50 1.0000
19 17 -4 998 459
1125 1.92 1.50 1.0000
17 17 -4 33 320
-2 -3.28 -3.72 1.0000
17 17 -4 1095 415
749 -3.28 -3.72 1.0000
15 17 -4 1058 370
1059 2.92 1.94 1.0000
15 17 -4 555 303
551 2.92 1.94 1.0000
13 17 -4 505 298

421	-2.50	-2.55	1.0000		
13	17	-4	253	253	
191	-2.50	-2.55	1.0000		
11	17	-4	286	271	
779	-8.48	6.32	1.0000		
11	17	-4	124	254	
478	-8.48	6.32	1.0000		
9	17	-4	224	214	
270	5.50	-6.29	1.0000		
9	17	-4	323	255	
399	5.50	-6.29	1.0000		
7	17	-4	52	227	
106	2.44	1.37	1.0000		
7	17	-4	10	180	
-160	2.44	1.37	1.0000		
5	17	-4	55	174	
7	1.04	8.64	1.0000		
3	17	-4	522	213	
1019	0.44	-10.49	1.0000		
-3	-17	4	771	310	
1365	0.44	10.49	1.0000		
1	17	-4	679	165	
317	5.74	5.91	1.0000		
-1	-17	4	553	244	
231	5.74	-5.91	1.0000		
-1	17	-4	2251	304	
3219	-2.90	-7.97	1.0000		
1	-17	4	2466	369	
3476	-2.90	7.97	1.0000		
-3	17	-4	707	199	
891	4.71	-12.60	1.0000		
3	-17	4	564	252	
691	4.71	12.60	1.0000		
-5	17	-4	523	183	
458	-3.56	-0.72	1.0000		
5	-17	4	540	226	
473	-3.56	0.72	1.0000		
-7	17	-4	425	200	
890	3.20	-8.38	1.0000		
7	-17	4	356	246	
789	3.20	8.38	1.0000		
9	-17	4	593	243	
760	0.66	-2.86	1.0000		
11	-17	4	503	226	

```
496     0.33   -0.48  1.0000
  13 -17   4     135     226
157     0.24   -0.90  1.0000
  15 -17   4     299     247
359     3.36    1.68  1.0000
  17 -17   4      32     264
-15    -3.34   -3.63  1.0000
  19 -17   4       3     274
-42     0.23   -1.53  1.0000
  20  16  -4     691     467
1138    -5.01     4.10  1.0000
  20  16  -4       2     342
78    -5.01     4.10  1.0000
  18  16  -4      12     331
11      4.62     1.66  1.0000
  18  16  -4    1046     423
1355      4.62     1.66  1.0000
  16  16  -4      39     292
74    -4.18     2.40  1.0000
  16  16  -4     584     352
766    -4.18     2.40  1.0000
  14  16  -4    1556     332
1596    -4.82   -0.32  1.0000
  12  16  -4     648     241
321     8.30    0.77  1.0000
  12  16  -4     555     253
254     8.30    0.77  1.0000
  10  16  -4     467     218
276     1.74    6.30  1.0000
  10  16  -4      57     216
-66     1.74    6.30  1.0000
   8  16  -4     465     249
431   -11.58   -1.97  1.0000
   8  16  -4     595     205
586   -11.58   -1.97  1.0000
   6  16  -4    1337     275
1883      4.62     3.85  1.0000
   6  16  -4     931     251
1395      4.62     3.85  1.0000
   4  16  -4     319     201
780   -10.68    1.72  1.0000
   2  16  -4     777     198
948    -2.10    2.74  1.0000
  -2 -16   4    1213     272
```

1427 -2.10 -2.74 1.0000
0 16 -4 2327 289
3167 6.69 7.44 1.0000
0 -16 4 2655 355
3544 6.69 -7.44 1.0000
-2 16 -4 318 162
470 -5.68 7.18 1.0000
2 -16 4 368 221
542 -5.68 -7.18 1.0000
-4 16 -4 348 148
162 2.64 7.25 1.0000
4 -16 4 261 199
101 2.64 -7.25 1.0000
-6 16 -4 2241 305
3561 7.06 11.49 1.0000
6 -16 4 2055 362
3319 7.06 -11.49 1.0000
8 -16 4 3898 329
2349 -12.02 1.83 1.0000
10 -16 4 295 205
178 5.24 3.01 1.0000
12 -16 4 279 213
133 -4.48 0.78 1.0000
14 -16 4 163 224
141 0.01 0.77 1.0000
16 -16 4 31 228
-55 -4.23 2.40 1.0000
18 -16 4 7 240
-79 2.16 0.51 1.0000
20 -16 4 64 273
35 0.84 2.43 1.0000
21 15 -4 1131 482
1593 0.35 5.88 1.0000
21 15 -4 134 377
313 0.35 5.88 1.0000
19 15 -4 316 365
710 -0.84 -7.77 1.0000
19 15 -4 783 436
1360 -0.84 -7.77 1.0000
17 15 -4 18 274
-22 3.27 1.65 1.0000
17 15 -4 958 376
1071 3.27 1.65 1.0000
15 15 -4 345 289

471   -0.03   2.85 1.0000
  15 15 -4   572   313
731   -0.03   2.85 1.0000
  13 15 -4   484   274
654   -4.30   -7.38 1.0000
  13 15 -4   502   275
677   -4.30   -7.38 1.0000
  11 15 -4   649   241
781   2.88   -3.98 1.0000
  11 15 -4   452   236
559   2.88   -3.98 1.0000
   9 15 -4   2   200
20   -4.91   -1.10 1.0000
   9 15 -4   7   185
10   -4.91   -1.10 1.0000
   7 15 -4   322   173
123   5.10   -3.88 1.0000
   7 15 -4   662   214
360   5.10   -3.88 1.0000
   5 15 -4   81   154
-170   7.58   -1.81 1.0000
   5 15 -4   81   182
-100   7.58   -1.81 1.0000
   3 15 -4   367   179
808   5.78   5.75 1.0000
  -3 -15   4   843   288
1469   5.78   -5.75 1.0000
   1 15 -4   882   147
209   -3.28   -12.81 1.0000
  -1 -15   4   563   209
72   -3.28   12.81 1.0000
  -1 15 -4   1687   166
743   11.53   -3.25 1.0000
   1 -15   4   1893   236
882   11.53   3.25 1.0000
  -3 15 -4   379   171
915   -10.38   -3.13 1.0000
   3 -15   4   251   216
692   -10.38   3.13 1.0000
  -5 15 -4   7   165
727   6.24   -22.69 1.0000
   5 -15   4   8   199
680   6.24   22.69 1.0000
  -7 15 -4   717   151

```
355    -1.30     6.75  1.0000
   7 -15    4       189       191
34    -1.30    -6.75  1.0000
   9 -15    4       309       210
294     2.20     0.11  1.0000
  11 -15    4       513       223
533     1.48    -0.60  1.0000
  13 -15    4        53       205
231    -4.69    -7.96  1.0000
  15 -15    4       327       241
452    -0.13     2.86  1.0000
  17 -15    4       278       240
271     3.00     1.99  1.0000
  19 -15    4       187       280
372    -3.28    -3.80  1.0000
  21 -15    4       147       289
211    -1.31    -1.97  1.0000
  20  14   -4        72       331
73     0.71    -0.74  1.0000
  20  14   -4      1525       446
1536     0.71    -0.74  1.0000
  18  14   -4       911       392
1135    -5.49     4.19  1.0000
  18  14   -4        64       283
-343    -5.49     4.19  1.0000
  16  14   -4       189       307
172     0.01     0.55  1.0000
  16  14   -4       275       275
254     0.01     0.55  1.0000
  14  14   -4       313       279
359     2.77    -0.90  1.0000
  14  14   -4       156       258
186     2.77    -0.90  1.0000
  12  14   -4       696       234
57    -6.39    15.06  1.0000
  12  14   -4       356       236
-106    -6.39    15.06  1.0000
  10  14   -4       272       198
372    -2.09    -1.80  1.0000
  10  14   -4       253       208
350    -2.09    -1.80  1.0000
   8  14   -4       384       243
1286     5.28    13.45  1.0000
   8  14   -4       361       214
```

1244	5.28	13.45	1.0000	
6	14	-4	90	173
570	-18.33	3.56	1.0000	
6	14	-4	202	231
968	-18.33	3.56	1.0000	
4	14	-4	266	156
489	3.85	-5.64	1.0000	
4	14	-4	371	181
633	3.85	-5.64	1.0000	
2	14	-4	806	164
723	-6.87	14.40	1.0000	
-2	-14	4	1243	230
1231	-6.87	-14.40	1.0000	
0	14	-4	394	125
89	-6.90	-6.91	1.0000	
0	-14	4	576	166
196	-6.90	6.91	1.0000	
-2	14	-4	1923	201
1157	13.29	13.18	1.0000	
2	-14	4	2301	241
1498	13.29	-13.18	1.0000	
-4	14	-4	512	137
370	5.30	16.12	1.0000	
4	-14	4	441	186
276	5.30	-16.12	1.0000	
-6	14	-4	657	194
1057	-5.56	-6.19	1.0000	
6	-14	4	531	210
895	-5.56	6.19	1.0000	
-8	14	-4	664	176
1040	14.30	7.31	1.0000	
8	-14	4	580	213
906	14.30	-7.31	1.0000	
10	-14	4	312	238
1158	-14.84	5.94	1.0000	
12	-14	4	713	254
1134	5.16	10.65	1.0000	
14	-14	4	628	232
696	2.82	-0.98	1.0000	
16	-14	4	869	266
900	-4.74	-0.15	1.0000	
18	-14	4	79	236
137	1.87	2.34	1.0000	
20	-14	4	8	260

-59	2.52	0.07	1.0000	
21	13	-4	1121	473
1768	4.16	-6.19	1.0000	
19	13	-4	1208	383
1008	2.41	3.73	1.0000	
19	13	-4	26	267
-75	2.41	3.73	1.0000	
17	13	-4	1	271
-141	0.36	-0.98	1.0000	
17	13	-4	186	313
216	0.36	-0.98	1.0000	
15	13	-4	113	267
-5	-4.71	-7.92	1.0000	
15	13	-4	22	259
95	-4.71	-7.92	1.0000	
13	13	-4	1977	302
1831	1.20	4.06	1.0000	
13	13	-4	2462	346
2302	1.20	4.06	1.0000	
11	13	-4	350	249
1428	0.01	-16.54	1.0000	
11	13	-4	574	287
1852	0.01	-16.54	1.0000	
9	13	-4	36	182
163	5.33	-7.21	1.0000	
9	13	-4	38	174
172	5.33	-7.21	1.0000	
7	13	-4	15	160
-34	-3.22	0.75	1.0000	
7	13	-4	15	147
-70	-3.22	0.75	1.0000	
5	13	-4	1862	263
2943	10.53	-9.39	1.0000	
5	13	-4	2066	282
3206	10.53	-9.39	1.0000	
3	13	-4	857	152
563	0.41	5.05	1.0000	
1	13	-4	2300	299
4340	14.75	6.56	1.0000	
-1	-13	4	2284	337
4325	14.75	-6.56	1.0000	
-1	13	-4	1658	229
2436	-11.09	-8.60	1.0000	
1	-13	4	1379	258

2086	-11.09	8.60	1.0000	
-3	13	-4	141	107
850	3.24	-14.67	1.0000	
-3	13	-4	105	151
757	3.24	-14.67	1.0000	
3	-13	4	124	180
806	3.24	14.67	1.0000	
-5	13	-4	1705	212
1805	-7.55	6.73	1.0000	
5	-13	4	1713	235
1809	-7.55	-6.73	1.0000	
-7	13	-4	633	207
1510	-5.66	-10.66	1.0000	
7	-13	4	569	249
1412	-5.66	10.66	1.0000	
-9	13	-4	1022	161
883	3.66	4.68	1.0000	
9	-13	4	990	198
856	3.66	-4.68	1.0000	
11	-13	4	1440	280
1674	-2.00	-15.27	1.0000	
13	-13	4	2325	315
2159	1.14	4.12	1.0000	
15	-13	4	474	239
637	-4.22	-7.32	1.0000	
17	-13	4	599	240
512	-2.68	-2.55	1.0000	
19	-13	4	518	259
598	-0.49	7.23	1.0000	
21	-13	4	101	286
287	1.43	-6.03	1.0000	
22	12	-4	1776	492
2296	-5.03	0.62	1.0000	
20	12	-4	1193	420
1664	-3.68	4.15	1.0000	
20	12	-4	215	337
436	-3.68	4.15	1.0000	
18	12	-4	396	339
543	-2.91	-1.39	1.0000	
18	12	-4	48	265
104	-2.91	-1.39	1.0000	
16	12	-4	218	234
89	-4.61	0.79	1.0000	
16	12	-4	29	273

-45 -4.61 0.79 1.0000
14 12 -4 432 270
730 5.11 10.54 1.0000
14 12 -4 498 272
827 5.11 10.54 1.0000
12 12 -4 410 219
367 -2.81 0.06 1.0000
12 12 -4 474 222
428 -2.81 0.06 1.0000
10 12 -4 270 208
168 -3.95 18.56 1.0000
10 12 -4 273 170
148 -3.95 18.56 1.0000
8 12 -4 59 147
-60 -4.32 5.05 1.0000
8 12 -4 90 154
118 -4.32 5.05 1.0000
6 12 -4 142 139
229 6.17 -3.97 1.0000
6 12 -4 64 153
94 6.17 -3.97 1.0000
4 12 -4 1966 200
1206 -18.50 16.13 1.0000
4 12 -4 2057 214
1299 -18.50 16.13 1.0000
2 12 -4 2669 381
7246 6.37 -29.38 1.0000
-2 -12 4 2416 420
6820 6.37 29.38 1.0000
0 12 -4 2174 253
3722 -3.02 12.12 1.0000
0 -12 4 1935 288
3407 -3.02 -12.12 1.0000
-2 12 -4 206 131
537 6.66 3.73 1.0000
-2 12 -4 292 89
671 6.66 3.73 1.0000
2 -12 4 313 101
702 6.66 -3.73 1.0000
2 -12 4 211 161
544 6.66 -3.73 1.0000
-4 12 -4 132 81
180 5.43 -7.10 1.0000
-4 12 -4 136 105

187	5.43	-7.10	1.0000	
4	-12	4	148	144
201	5.43	7.10	1.0000	
-6	12	-4	1764	176
2747	18.46	17.84	1.0000	
-6	12	-4	1824	222
2837	18.46	17.84	1.0000	
6	-12	4	1685	249
2620	18.46	-17.84	1.0000	
-8	12	-4	946	108
479	-3.90	-15.00	1.0000	
-8	12	-4	704	117
273	-3.90	-15.00	1.0000	
8	-12	4	657	166
240	-3.90	15.00	1.0000	
10	-12	4	2587	266
1960	7.45	7.01	1.0000	
12	-12	4	526	196
484	-2.79	-0.06	1.0000	
14	-12	4	784	195
83	-6.37	15.14	1.0000	
16	-12	4	828	224
453	8.34	0.83	1.0000	
18	-12	4	241	235
149	1.56	-4.54	1.0000	
20	-12	4	81	257
260	-5.29	3.53	1.0000	
22	-12	4	281	312
510	5.19	-0.63	1.0000	
21	11	-4	2562	416
2593	2.90	-1.23	1.0000	
21	11	-4	777	378
788	2.90	-1.23	1.0000	
19	11	-4	1038	360
869	3.75	-1.91	1.0000	
19	11	-4	200	316
122	3.75	-1.91	1.0000	
17	11	-4	497	289
492	0.28	-0.38	1.0000	
17	11	-4	1035	304
1028	0.28	-0.38	1.0000	
15	11	-4	764	249
783	1.49	-0.73	1.0000	
15	11	-4	845	268

865	1.49	-0.73	1.0000	
13	11	-4	1348	265
1580	-1.84	-15.34	1.0000	
13	11	-4	1278	272
1494	-1.84	-15.34	1.0000	
11	11	-4	175	186
163	0.25	0.37	1.0000	
11	11	-4	417	209
398	0.25	0.37	1.0000	
9	11	-4	225	150
-130	-5.61	-11.72	1.0000	
9	11	-4	519	168
112	-5.61	-11.72	1.0000	
7	11	-4	1955	242
2571	11.47	-11.35	1.0000	
7	11	-4	1696	250
2250	11.47	-11.35	1.0000	
5	11	-4	4012	232
2613	-9.52	19.63	1.0000	
5	11	-4	4193	247
2771	-9.52	19.63	1.0000	
3	11	-4	3675	284
4443	13.02	-6.68	1.0000	
3	11	-4	3644	297
4408	13.02	-6.68	1.0000	
1	11	-4	2311	287
4697	-6.97	16.39	1.0000	
-1	-11	4	2229	319
4581	-6.97	-16.39	1.0000	
-1	11	-4	2241	130
2199	-1.84	5.72	1.0000	
-1	11	-4	2069	200
2026	-1.84	5.72	1.0000	
1	-11	4	2128	229
2088	-1.84	-5.72	1.0000	
-3	11	-4	1518	167
970	-7.68	-2.62	1.0000	
-3	11	-4	2005	112
1370	-7.68	-2.62	1.0000	
3	-11	4	2006	127
1366	-7.68	2.62	1.0000	
3	-11	4	1630	182
1057	-7.68	2.62	1.0000	
-5	11	-4	2075	121

1394	-11.32	-3.22	1.0000	
-5	11	-4	1987	167
1320	-11.32	-3.22	1.0000	
5	-11	4	1964	191
1298	-11.32	3.22	1.0000	
-7	11	-4	1134	135
1605	-5.08	4.85	1.0000	
-7	11	-4	1027	177
1475	-5.08	4.85	1.0000	
7	-11	4	1006	207
1446	-5.08	-4.85	1.0000	
-9	11	-4	1355	177
2452	-8.84	8.77	1.0000	
-9	11	-4	1384	209
2491	-8.84	8.77	1.0000	
9	-11	4	1297	263
2379	-8.84	-8.77	1.0000	
-11	11	-4	970	149
947	0.21	-0.28	1.0000	
11	-11	4	868	207
846	0.21	0.28	1.0000	
13	-11	4	427	283
1581	0.08	-16.57	1.0000	
15	-11	4	556	228
682	2.68	-3.97	1.0000	
17	-11	4	252	245
720	-8.47	6.31	1.0000	
19	-11	4	103	229
-53	2.87	-8.31	1.0000	
21	-11	4	187	285
423	-0.20	6.25	1.0000	
23	-11	4	1	273
-149	-0.77	0.16	1.0000	
20	10	-4	9	328
36	-7.61	0.93	1.0000	
20	10	-4	720	379
1268	-7.61	0.93	1.0000	
18	10	-4	61	301
131	-3.70	-2.46	1.0000	
18	10	-4	621	316
838	-3.70	-2.46	1.0000	
16	10	-4	265	271
152	5.17	2.80	1.0000	
16	10	-4	754	274

577 5.17 2.80 1.0000
14 10 -4 345 270
1238 -14.97 6.05 1.0000
14 10 -4 240 264
1018 -14.97 6.05 1.0000
12 10 -4 2375 282
1779 7.37 7.02 1.0000
12 10 -4 2426 253
1824 7.37 7.02 1.0000
10 10 -4 2467 273
2164 8.19 8.57 1.0000
10 10 -4 2593 272
2287 8.19 8.57 1.0000
8 10 -4 150 141
162 -4.25 2.71 1.0000
8 10 -4 377 167
416 -4.25 2.71 1.0000
6 10 -4 131 136
34 -5.50 4.17 1.0000
6 10 -4 213 126
94 -5.50 4.17 1.0000
4 10 -4 2184 236
2531 13.28 -31.97 1.0000
4 10 -4 2382 230
2846 13.28 -31.97 1.0000
2 10 -4 7871 622
13774 -24.76 1.88 1.0000
0 10 -4 82 168
1208 26.77 -24.76 1.0000
0 -10 4 38 210
1563 26.77 24.76 1.0000
-2 10 -4 1096 181
1791 -1.23 -8.47 1.0000
-2 10 -4 1189 124
1910 -1.23 -8.47 1.0000
2 -10 4 1164 130
1876 -1.23 8.47 1.0000
2 -10 4 895 195
1529 -1.23 8.47 1.0000
-4 10 -4 2252 235
3639 13.00 9.51 1.0000
-4 10 -4 2225 191
3604 13.00 9.51 1.0000
4 -10 4 2197 254

3571 13.00 -9.51 1.0000
-6 10 -4 1176 84
171 11.27 -14.84 1.0000
-6 10 -4 977 91
100 11.27 -14.84 1.0000
6 -10 4 462 108
-4 11.27 14.84 1.0000
-8 10 -4 399 94
148 5.17 7.38 1.0000
-8 10 -4 311 95
91 5.17 7.38 1.0000
8 -10 4 108 128
-24 5.17 -7.38 1.0000
-10 10 -4 3354 198
3040 7.89 -8.36 1.0000
10 -10 4 3305 278
2994 7.89 8.36 1.0000
-12 10 -4 318 125
515 -3.82 -18.67 1.0000
12 -10 4 312 188
455 -3.82 18.67 1.0000
-14 10 -4 615 151
771 -2.05 1.95 1.0000
14 -10 4 323 195
437 -2.05 -1.95 1.0000
16 -10 4 208 201
79 1.76 6.23 1.0000
18 -10 4 22 205
-101 -2.99 2.69 1.0000
20 -10 4 723 254
335 5.63 -4.90 1.0000
22 -10 4 13 258
-83 -2.74 -1.53 1.0000
19 9 -4 2023 380
1913 -1.11 1.66 1.0000
19 9 -4 1091 328
1010 -1.11 1.66 1.0000
17 9 -4 608 295
776 0.59 -2.84 1.0000
17 9 -4 600 259
767 0.59 -2.84 1.0000
15 9 -4 154 227
142 2.36 0.07 1.0000
15 9 -4 291 258

```
277      2.36     0.07  1.0000
  13    9   -4      802      207
680      3.57    -4.58  1.0000
  13    9   -4      906      247
779      3.57    -4.58  1.0000
  11    9   -4      875      257
1803    -8.99    -8.93  1.0000
  11    9   -4      982      251
1958    -8.99    -8.93  1.0000
   9    9   -4     1779      231
1612    -0.96    -1.94  1.0000
   9    9   -4     2110      232
1928    -0.96    -1.94  1.0000
   7    9   -4     2139      220
1585   -13.24     2.46  1.0000
   7    9   -4     2445      220
1865   -13.24     2.46  1.0000
   5    9   -4      746      188
1006     7.37    -1.37  1.0000
   5    9   -4      757      179
1019     7.37    -1.37  1.0000
   3    9   -4     1324      365
6876    -5.32    40.31  1.0000
   3    9   -4     1405      368
7061    -5.32    40.31  1.0000
   1    9   -4    12469      611
13991    -2.00     5.38  1.0000
  -1   -9    4    11734      627
13212    -2.00    -5.38  1.0000
  -1    9   -4     1004       62
130   -12.66    12.28  1.0000
  -1    9   -4      566       91
12   -12.66    12.28  1.0000
   1   -9    4      414      116
-20   -12.66   -12.28  1.0000
  -3    9   -4     3956      182
2010   -12.35    19.95  1.0000
  -3    9   -4     4251      136
2238   -12.35    19.95  1.0000
   3   -9    4     3888      195
1974   -12.35   -19.95  1.0000
   3   -9    4     4111      144
2147   -12.35   -19.95  1.0000
  -5    9   -4     1180      102
```

289 -14.26 -9.59 1.0000
-5 9 -4 1458 88
446 -14.26 -9.59 1.0000
5 -9 4 1196 120
296 -14.26 9.59 1.0000
-7 9 -4 2840 227
3718 -12.33 5.37 1.0000
-7 9 -4 2772 203
3638 -12.33 5.37 1.0000
7 -9 4 2655 252
3502 -12.33 -5.37 1.0000
-9 9 -4 1915 146
1747 -1.07 1.92 1.0000
-9 9 -4 1823 165
1659 -1.07 1.92 1.0000
9 -9 4 1583 212
1430 -1.07 -1.92 1.0000
-11 9 -4 721 106
246 -5.48 11.85 1.0000
11 -9 4 227 151
-90 -5.48 -11.85 1.0000
-13 9 -4 232 134
553 5.13 7.36 1.0000
13 -9 4 76 179
267 5.13 -7.36 1.0000
-15 9 -4 0 127
29 -4.80 1.05 1.0000
15 -9 4 22 189
108 -4.80 -1.05 1.0000
17 -9 4 115 214
109 5.53 -6.22 1.0000
19 -9 4 111 246
396 -3.47 8.73 1.0000
21 -9 4 33 236
20 -1.01 1.15 1.0000
23 -9 4 313 291
249 1.65 -0.07 1.0000
20 8 -4 486 267
1132 4.68 -8.90 1.0000
20 8 -4 179 332
624 4.68 -8.90 1.0000
18 8 -4 237 217
237 0.05 0.14 1.0000
18 8 -4 474 287

474	0.05	0.14	1.0000	
16	8	-4	2689	306
1435	-12.01	1.88	1.0000	
16	8	-4	3024	322
1683	-12.01	1.88	1.0000	
14	8	-4	642	221
997	14.28	-7.43	1.0000	
14	8	-4	738	261
1143	14.28	-7.43	1.0000	
12	8	-4	436	93
68	-3.77	15.02	1.0000	
12	8	-4	534	163
144	-3.77	15.02	1.0000	
12	8	-4	687	191
270	-3.77	15.02	1.0000	
10	8	-4	266	148
67	5.11	-7.24	1.0000	
10	8	-4	281	144
76	5.11	-7.24	1.0000	
8	8	-4	915	208
1543	14.09	2.38	1.0000	
8	8	-4	887	210
1504	14.09	2.38	1.0000	
6	8	-4	190	138
470	3.51	-8.60	1.0000	
6	8	-4	118	145
337	3.51	-8.60	1.0000	
4	8	-4	3114	127
426	-14.02	-27.14	1.0000	
-4	-8	4	2697	113
281	-14.02	27.14	1.0000	
2	8	-4	13396	278
4916	27.11	-30.20	1.0000	
-2	-8	4	12548	280
4398	27.11	30.20	1.0000	
0	8	-4	1931	286
5224	-26.20	-22.02	1.0000	
0	8	-4	1846	230
5064	-26.20	-22.02	1.0000	
0	-8	4	1681	288
4746	-26.20	22.02	1.0000	
0	-8	4	1954	296
5266	-26.20	22.02	1.0000	
-2	8	-4	1844	273

4839	20.79	-17.34	1.0000	
-2	8	-4	1989	231
5083	20.79	-17.34	1.0000	
2	-8	4	1843	232
4854	20.79	17.34	1.0000	
2	-8	4	1827	279
4827	20.79	17.34	1.0000	
-4	8	-4	1410	174
3243	16.82	-1.59	1.0000	
-4	8	-4	1229	205
2965	16.82	-1.59	1.0000	
4	-8	4	1292	215
3065	16.82	1.59	1.0000	
-6	8	-4	2085	175
2815	2.55	8.25	1.0000	
-6	8	-4	2177	201
2923	2.55	8.25	1.0000	
6	-8	4	2626	228
3441	2.55	-8.25	1.0000	
-8	8	-4	1145	158
1840	13.87	-2.05	1.0000	
-8	8	-4	1112	144
1795	13.87	-2.05	1.0000	
8	-8	4	1146	199
1823	13.87	2.05	1.0000	
-10	8	-4	270	100
295	-4.17	-2.66	1.0000	
10	-8	4	280	148
308	-4.17	2.66	1.0000	
-12	8	-4	352	124
455	-4.40	-4.88	1.0000	
12	-8	4	341	171
444	-4.40	4.88	1.0000	
-14	8	-4	580	174
1624	5.27	-13.39	1.0000	
14	-8	4	405	238
1319	5.27	13.39	1.0000	
-16	8	-4	516	145
494	-11.62	2.06	1.0000	
16	-8	4	428	212
383	-11.62	-2.06	1.0000	
18	-8	4	308	229
420	3.51	-0.90	1.0000	
20	-8	4	7	221

-129	-0.46	-2.33	1.0000	
22	-8	4	246	278
433	3.74	-2.47	1.0000	
24	-8	4	101	311
139	-0.22	-1.63	1.0000	
21	7	-4	453	251
513	-2.58	-1.72	1.0000	
21	7	-4	455	304
516	-2.58	-1.72	1.0000	
19	7	-4	261	273
304	0.06	3.03	1.0000	
19	7	-4	343	242
393	0.06	3.03	1.0000	
17	7	-4	388	264
838	3.28	8.35	1.0000	
17	7	-4	394	272
848	3.28	8.35	1.0000	
15	7	-4	445	214
173	-1.48	-6.72	1.0000	
15	7	-4	637	234
299	-1.48	-6.72	1.0000	
13	7	-4	567	286
1401	-5.76	10.48	1.0000	
13	7	-4	629	265
1498	-5.76	10.48	1.0000	
11	7	-4	1271	265
1747	-4.92	-4.76	1.0000	
11	7	-4	1238	240
1708	-4.92	-4.76	1.0000	
9	7	-4	3098	306
4035	-12.47	-5.45	1.0000	
9	7	-4	3699	304
4735	-12.47	-5.45	1.0000	
7	7	-4	8358	547
11612	-5.96	16.16	1.0000	
7	7	-4	8879	564
12228	-5.96	16.16	1.0000	
5	7	-4	197	128
407	-10.63	9.17	1.0000	
5	7	-4	178	128
361	-10.63	9.17	1.0000	
-5	-7	4	190	133
417	-10.63	-9.17	1.0000	
3	7	-4	1279	213

```
2860    -6.91    15.81  1.0000
   3    7   -4     1240      206
2799    -6.91    15.81  1.0000
  -3   -7    4     1199      201
2736    -6.91   -15.81  1.0000
   1    7   -4     1275      163
3376    -6.41    35.58  1.0000
   1    7   -4     1264      222
3351    -6.41    35.58  1.0000
  -1   -7    4     1274      228
3290    -6.41   -35.58  1.0000
  -1   -7    4     1228      210
3182    -6.41   -35.58  1.0000
  -1    7   -4    20976      655
15927     5.52    25.89  1.0000
  -1    7   -4    20133      619
15182     5.52    25.89  1.0000
   1   -7    4    19370      650
14543     5.52   -25.89  1.0000
   1   -7    4    19549      654
14701     5.52   -25.89  1.0000
  -3    7   -4     3204      251
4686   -22.11   -10.83  1.0000
  -3    7   -4     2677      270
3979   -22.11   -10.83  1.0000
  -3    7   -4     3171      216
4642   -22.11   -10.83  1.0000
   3   -7    4     2915      252
4303   -22.11    10.83  1.0000
   3   -7    4     3090      220
4536   -22.11    10.83  1.0000
  -5    7   -4      706       94
608   -14.25    15.92  1.0000
  -5    7   -4      639      104
489   -14.25    15.92  1.0000
   5   -7    4      629      109
445   -14.25   -15.92  1.0000
  -7    7   -4     9445      550
12896    -6.04   -16.08  1.0000
  -7    7   -4     9429      557
12878    -6.04   -16.08  1.0000
   7   -7    4     9221      584
12634    -6.04    16.08  1.0000
  -9    7   -4     3006      165
```

2366	-13.35	-2.44	1.0000	
-9	7	-4	3012	168
2371	-13.35	-2.44	1.0000	
9	-7	4	2777	213
2164	-13.35	2.44	1.0000	
-11	7	-4	2350	194
3065	11.44	11.49	1.0000	
11	-7	4	2165	250
2821	11.44	-11.49	1.0000	
-13	7	-4	245	108
266	-3.09	-0.77	1.0000	
13	-7	4	113	167
123	-3.09	0.77	1.0000	
-15	7	-4	528	126
259	5.12	3.96	1.0000	
15	-7	4	701	198
386	5.12	-3.96	1.0000	
-17	7	-4	75	133
139	2.47	-1.30	1.0000	
17	-7	4	53	203
107	2.47	1.30	1.0000	
19	-7	4	518	240
681	1.80	2.58	1.0000	
21	-7	4	252	258
324	-4.44	5.85	1.0000	
23	-7	4	7	283
-32	0.36	2.21	1.0000	
22	6	-4	71	299
111	2.60	-5.11	1.0000	
22	6	-4	340	272
470	2.60	-5.11	1.0000	
20	6	-4	675	298
847	-2.81	-0.02	1.0000	
20	6	-4	500	283
650	-2.81	-0.02	1.0000	
18	6	-4	1384	301
1408	-3.45	-4.42	1.0000	
18	6	-4	1100	273
1117	-3.45	-4.42	1.0000	
16	6	-4	1860	364
3063	6.98	-11.59	1.0000	
16	6	-4	1692	361
2841	6.98	-11.59	1.0000	
14	6	-4	534	218

894	-5.50	6.15	1.0000	
14	6	-4	561	214
931	-5.50	6.15	1.0000	
12	6	-4	1841	293
2834	18.47	-17.66	1.0000	
12	6	-4	1765	288
2720	18.47	-17.66	1.0000	
10	6	-4	1378	146
250	11.48	14.88	1.0000	
10	6	-4	863	143
63	11.48	14.88	1.0000	
8	6	-4	2373	246
3145	2.47	-8.20	1.0000	
8	6	-4	2515	268
3310	2.47	-8.20	1.0000	
6	6	-4	1413	308
5006	9.30	-34.17	1.0000	
6	6	-4	1264	278
4686	9.30	-34.17	1.0000	
-6	-6	4	1469	291
5112	9.30	34.17	1.0000	
4	6	-4	9679	545
12464	8.31	8.26	1.0000	
4	6	-4	8225	514
10806	8.31	8.26	1.0000	
-4	-6	4	9342	526
12081	8.31	-8.26	1.0000	
2	6	-4	14281	429
9378	-18.00	-55.33	1.0000	
2	6	-4	13875	409
8980	-18.00	-55.33	1.0000	
-2	-6	4	13548	413
8788	-18.00	55.33	1.0000	
0	6	-4	19512	1151
28285	18.35	-20.62	1.0000	
0	6	-4	19231	1119
27945	18.35	-20.62	1.0000	
0	-6	4	18738	1139
27352	18.35	20.62	1.0000	
0	-6	4	19488	1148
28261	18.35	20.62	1.0000	
-2	6	-4	565	153
1189	-13.80	0.00	1.0000	
-2	6	-4	602	100

1247	-13.80	0.00	1.0000	
-2	6	-4	522	143
1120	-13.80	0.00	1.0000	
2	-6	4	590	133
1215	-13.80	0.00	1.0000	
2	-6	4	764	105
1480	-13.80	0.00	1.0000	
-4	6	-4	5074	208
2922	7.38	-16.92	1.0000	
-4	6	-4	5142	189
2975	7.38	-16.92	1.0000	
-4	6	-4	5042	162
2897	7.38	-16.92	1.0000	
4	-6	4	4871	205
2772	7.38	16.92	1.0000	
4	-6	4	4723	163
2658	7.38	16.92	1.0000	
4	-6	4	5052	192
2912	7.38	16.92	1.0000	
-6	6	-4	1797	284
5789	9.12	34.22	1.0000	
-6	6	-4	1966	297
6114	9.12	34.22	1.0000	
6	-6	4	1956	309
6106	9.12	-34.22	1.0000	
6	-6	4	1926	314
6049	9.12	-34.22	1.0000	
-8	6	-4	342	109
708	3.66	8.56	1.0000	
-8	6	-4	315	100
667	3.66	8.56	1.0000	
8	-6	4	177	133
443	3.66	-8.56	1.0000	
8	-6	4	415	134
817	3.66	-8.56	1.0000	
-10	6	-4	302	94
165	-5.48	-4.18	1.0000	
-10	6	-4	306	74
168	-5.48	-4.18	1.0000	
10	-6	4	322	127
181	-5.48	4.18	1.0000	
-12	6	-4	278	117
435	6.17	4.25	1.0000	
12	-6	4	141	143

237 6.17 -4.25 1.0000
-14 6 -4 208 151
995 -18.35 -3.39 1.0000
14 -6 4 165 200
858 -18.35 3.39 1.0000
-16 6 -4 1104 182
1591 4.49 -3.74 1.0000
16 -6 4 1111 281
1598 4.49 3.74 1.0000
-18 6 -4 258 138
112 -7.11 1.63 1.0000
18 -6 4 71 192
-69 -7.11 -1.63 1.0000
20 -6 4 259 240
237 2.75 -3.89 1.0000
22 -6 4 22 260
30 1.64 -1.69 1.0000
24 -6 4 64 295
131 2.33 -2.67 1.0000
25 5 -4 1183 330
1207 -0.84 0.62 1.0000
25 5 -4 111 354
117 -0.84 0.62 1.0000
23 5 -4 29 300
-171 -4.47 -1.26 1.0000
23 5 -4 29 299
-254 -4.47 -1.26 1.0000
21 5 -4 387 300
445 0.78 4.45 1.0000
21 5 -4 239 277
280 0.78 4.45 1.0000
19 5 -4 547 278
725 -0.60 3.89 1.0000
19 5 -4 784 297
996 -0.60 3.89 1.0000
17 5 -4 560 266
486 -3.69 0.65 1.0000
17 5 -4 461 249
393 -3.69 0.65 1.0000
15 5 -4 16 234
887 6.19 22.54 1.0000
15 5 -4 8 230
696 6.19 22.54 1.0000
13 5 -4 2243 284

2380	-7.61	-6.83	1.0000	
13	5	-4	2224	308
2360	-7.61	-6.83	1.0000	
11	5	-4	2018	209
1333	-11.52	3.29	1.0000	
11	5	-4	2290	251
1564	-11.52	3.29	1.0000	
9	5	-4	2153	163
885	-14.14	9.60	1.0000	
9	5	-4	1719	164
604	-14.14	9.60	1.0000	
7	5	-4	673	129
529	-14.13	-15.89	1.0000	
7	5	-4	723	159
615	-14.13	-15.89	1.0000	
-7	-5	4	708	127
615	-14.13	15.89	1.0000	
5	5	-4	7199	167
1837	-8.96	35.51	1.0000	
5	5	-4	7436	212
1958	-8.96	35.51	1.0000	
-5	-5	4	7192	164
1833	-8.96	-35.51	1.0000	
3	5	-4	1188	169
933	4.39	6.91	1.0000	
3	5	-4	1194	111
938	4.39	6.91	1.0000	
-3	-5	4	1246	123
975	4.39	-6.91	1.0000	
1	5	-4	6939	234
3872	-0.76	25.55	1.0000	
-1	-5	4	6427	210
3473	-0.76	-25.55	1.0000	
-1	-5	4	6648	199
3644	-0.76	-25.55	1.0000	
-1	5	-4	3268	124
1992	-10.71	4.92	1.0000	
-1	5	-4	3324	167
2036	-10.71	4.92	1.0000	
1	-5	4	3594	151
2246	-10.71	-4.92	1.0000	
1	-5	4	3539	197
2202	-10.71	-4.92	1.0000	
-3	5	-4	2854	243

5365	4.03	22.38	1.0000	
-3	5	-4	2592	267
4990	4.03	22.38	1.0000	
-3	5	-4	2823	265
5322	4.03	22.38	1.0000	
3	-5	4	2913	245
5446	4.03	-22.38	1.0000	
3	-5	4	2719	261
5169	4.03	-22.38	1.0000	
3	-5	4	2647	280
5065	4.03	-22.38	1.0000	
-5	5	-4	7251	131
1856	-8.80	-35.60	1.0000	
-5	5	-4	7342	150
1902	-8.80	-35.60	1.0000	
-5	5	-4	7075	167
1767	-8.80	-35.60	1.0000	
5	-5	4	7095	153
1778	-8.80	35.60	1.0000	
5	-5	4	7311	167
1887	-8.80	35.60	1.0000	
-7	5	-4	317	99
687	-10.77	-9.25	1.0000	
-7	5	-4	295	101
646	-10.77	-9.25	1.0000	
7	-5	4	359	141
737	-10.77	9.25	1.0000	
7	-5	4	276	117
583	-10.77	9.25	1.0000	
-9	5	-4	923	121
1236	7.34	1.61	1.0000	
-9	5	-4	989	128
1314	7.34	1.61	1.0000	
9	-5	4	1275	152
1644	7.34	-1.61	1.0000	
9	-5	4	872	175
1172	7.34	-1.61	1.0000	
-11	5	-4	4709	198
3286	-9.80	-19.62	1.0000	
11	-5	4	4675	260
3223	-9.80	19.62	1.0000	
-13	5	-4	2503	225
3761	10.62	9.38	1.0000	
13	-5	4	2051	297

3186 10.62 -9.38 1.0000
-15 5 -4 86 119
-49 7.82 1.95 1.0000
15 -5 4 86 170
-2 7.82 -1.95 1.0000
-17 5 -4 10 123
171 0.94 -8.62 1.0000
17 -5 4 44 210
275 0.94 8.62 1.0000
-19 5 -4 1224 189
977 -3.68 -0.27 1.0000
19 -5 4 1179 268
938 -3.68 0.27 1.0000
21 -5 4 35 250
138 6.14 0.77 1.0000
23 -5 4 80 276
257 -5.09 5.06 1.0000
25 -5 4 2 299
-23 0.62 1.14 1.0000
24 4 -4 100 339
75 0.58 -1.27 1.0000
24 4 -4 859 343
785 0.58 -1.27 1.0000
22 4 -4 328 316
346 2.92 -1.61 1.0000
22 4 -4 15 295
-170 2.92 -1.61 1.0000
20 4 -4 419 289
470 -1.52 0.04 1.0000
18 4 -4 1841 309
1660 3.02 -8.87 1.0000
16 4 -4 533 224
316 2.73 -7.20 1.0000
16 4 -4 768 221
513 2.73 -7.20 1.0000
14 4 -4 591 198
496 5.24 -15.92 1.0000
14 4 -4 627 203
545 5.24 -15.92 1.0000
12 4 -4 214 169
296 5.50 6.86 1.0000
12 4 -4 156 155
208 5.50 6.86 1.0000
10 4 -4 2295 285

3685	12.89	-9.44	1.0000	
10	4	-4	2134	264
3474	12.89	-9.44	1.0000	
8	4	-4	1460	260
3333	16.91	1.72	1.0000	
8	4	-4	1390	233
3227	16.91	1.72	1.0000	
-8	-4	4	1376	230
3203	16.91	-1.72	1.0000	
6	4	-4	5304	235
3067	7.44	17.28	1.0000	
6	4	-4	4824	198
2697	7.44	17.28	1.0000	
-6	-4	4	5055	193
2867	7.44	-17.28	1.0000	
4	4	-4	829	474
9987	-12.74	-61.83	1.0000	
4	4	-4	748	440
9687	-12.74	-61.83	1.0000	
-4	-4	4	1035	443
10701	-12.74	61.83	1.0000	
2	4	-4	2559	110
1031	-2.07	16.04	1.0000	
2	4	-4	2988	175
1310	-2.07	16.04	1.0000	
-2	-4	4	2903	156
1253	-2.07	-16.04	1.0000	
-2	-4	4	2516	112
1003	-2.07	-16.04	1.0000	
0	4	-4	6508	410
8718	-21.69	-25.27	1.0000	
0	4	-4	6368	368
8541	-21.69	-25.27	1.0000	
0	-4	4	6761	379
9008	-21.69	25.27	1.0000	
0	-4	4	6933	381
9224	-21.69	25.27	1.0000	
0	-4	4	6117	408
8191	-21.69	25.27	1.0000	
-2	4	-4	3766	114
1170	9.19	-25.54	1.0000	
-2	4	-4	3652	94
1103	9.19	-25.54	1.0000	
-2	4	-4	3335	134

921 9.19 -25.54 1.0000
2 -4 4 3601 102
1068 9.19 25.54 1.0000
2 -4 4 3613 152
1075 9.19 25.54 1.0000
-4 4 -4 1156 473
11094 -12.83 61.82 1.0000
-4 4 -4 1211 489
11269 -12.83 61.82 1.0000
-4 4 -4 1204 483
11247 -12.83 61.82 1.0000
4 -4 4 1165 495
11121 -12.83 -61.82 1.0000
4 -4 4 1288 487
11508 -12.83 -61.82 1.0000
4 -4 4 1066 473
10803 -12.83 -61.82 1.0000
-6 4 -4 10040 531
12846 8.43 -8.05 1.0000
-6 4 -4 9718 532
12481 8.43 -8.05 1.0000
-6 4 -4 9480 552
12211 8.43 -8.05 1.0000
6 -4 4 9622 552
12373 8.43 8.05 1.0000
6 -4 4 9878 541
12663 8.43 8.05 1.0000
-8 4 -4 3351 97
507 -14.19 27.25 1.0000
-8 4 -4 3971 110
765 -14.19 27.25 1.0000
8 -4 4 3829 135
703 -14.19 -27.25 1.0000
8 -4 4 3944 132
753 -14.19 -27.25 1.0000
-10 4 -4 2539 185
3036 13.38 32.01 1.0000
10 -4 4 2315 234
2734 13.38 -32.01 1.0000
-12 4 -4 1969 142
1248 -18.32 -15.95 1.0000
12 -4 4 2166 211
1410 -18.32 15.95 1.0000
-14 4 -4 563 143

895 3.73 5.81 1.0000
14 -4 4 344 189
606 3.73 -5.81 1.0000
-16 4 -4 211 141
583 -10.56 -1.67 1.0000
16 -4 4 324 217
780 -10.56 1.67 1.0000
-18 4 -4 213 136
254 2.68 1.05 1.0000
18 -4 4 203 224
243 2.68 -1.05 1.0000
-20 4 -4 794 178
841 -0.11 1.95 1.0000
20 -4 4 663 249
706 -0.11 -1.95 1.0000
22 -4 4 8 246
-149 0.13 -2.44 1.0000
24 -4 4 201 303
284 2.25 -2.47 1.0000
25 3 -4 8 356
-238 -1.21 2.11 1.0000
25 3 -4 111 354
68 -1.21 2.11 1.0000
23 3 -4 5 296
-453 -1.34 1.45 1.0000
23 3 -4 5 328
-463 -1.34 1.45 1.0000
21 3 -4 408 302
335 -1.43 1.35 1.0000
21 3 -4 5 287
-105 -1.43 1.35 1.0000
19 3 -4 1084 293
822 -2.18 3.76 1.0000
19 3 -4 25 259
-10 -2.18 3.76 1.0000
17 3 -4 1507 345
1876 4.66 12.62 1.0000
15 3 -4 505 221
1113 -10.49 2.88 1.0000
15 3 -4 378 227
914 -10.49 2.88 1.0000
13 3 -4 37 191
543 3.19 14.64 1.0000
13 3 -4 119 184

792	3.19	14.64	1.0000	
11	3	-4	2024	219
1381	-7.70	2.67	1.0000	
11	3	-4	1864	216
1249	-7.70	2.67	1.0000	
9	3	-4	4074	217
2117	-12.36	-19.90	1.0000	
9	3	-4	3705	209
1833	-12.36	-19.90	1.0000	
7	3	-4	2537	238
3802	-22.20	10.90	1.0000	
7	3	-4	2681	260
4000	-22.20	10.90	1.0000	
-7	-3	4	2664	231
3974	-22.20	-10.90	1.0000	
5	3	-4	2596	260
5022	4.04	-22.60	1.0000	
5	3	-4	2701	295
5174	4.04	-22.60	1.0000	
-5	-3	4	3044	263
5668	4.04	22.60	1.0000	
3	3	-4	16283	352
7999	-5.04	33.08	1.0000	
3	3	-4	16565	393
8197	-5.04	33.08	1.0000	
-3	-3	4	16638	384
8247	-5.04	-33.08	1.0000	
1	3	-4	2233	261
4415	21.44	-0.53	1.0000	
-1	-3	4	2249	247
4455	21.44	0.53	1.0000	
-1	3	-4	6109	474
10511	12.13	17.34	1.0000	
-1	3	-4	6241	449
10684	12.13	17.34	1.0000	
-1	3	-4	6073	443
10464	12.13	17.34	1.0000	
1	-3	4	6055	468
10442	12.13	-17.34	1.0000	
1	-3	4	6374	442
10859	12.13	-17.34	1.0000	
-3	3	-4	17349	393
8764	-5.13	-32.99	1.0000	
-3	3	-4	17303	376

8731	-5.13	-32.99	1.0000	
-3	3	-4	17823	382
9102	-5.13	-32.99	1.0000	
3	-3	4	17042	377
8548	-5.13	32.99	1.0000	
3	-3	4	16767	393
8353	-5.13	32.99	1.0000	
-5	3	-4	1407	119
1125	4.36	-6.83	1.0000	
-5	3	-4	1627	106
1329	4.36	-6.83	1.0000	
-5	3	-4	1415	116
1133	4.36	-6.83	1.0000	
5	-3	4	1439	138
1166	4.36	6.83	1.0000	
5	-3	4	1394	120
1124	4.36	6.83	1.0000	
-7	3	-4	1786	192
3630	-7.02	-15.95	1.0000	
-7	3	-4	1818	182
3676	-7.02	-15.95	1.0000	
-7	3	-4	1501	213
3212	-7.02	-15.95	1.0000	
7	-3	4	1508	206
3221	-7.02	15.95	1.0000	
7	-3	4	1656	217
3441	-7.02	15.95	1.0000	
-9	3	-4	1456	330
7176	-5.31	-40.31	1.0000	
9	-3	4	1239	364
6679	-5.31	40.31	1.0000	
-11	3	-4	3480	236
4197	13.10	6.51	1.0000	
11	-3	4	3710	291
4457	13.10	-6.51	1.0000	
-13	3	-4	1070	129
746	0.35	-4.91	1.0000	
13	-3	4	826	169
544	0.35	4.91	1.0000	
-15	3	-4	535	154
1039	5.52	-5.81	1.0000	
15	-3	4	445	213
911	5.52	5.81	1.0000	
-17	3	-4	646	169

1194	0.69	10.68	1.0000	
17	-3	4	622	249
1167	0.69	-10.68	1.0000	
-19	3	-4	436	134
96	4.31	-8.58	1.0000	
19	-3	4	898	221
357	4.31	8.58	1.0000	
21	-3	4	32	239
148	-7.57	2.48	1.0000	
23	-3	4	36	271
62	5.22	-0.91	1.0000	
25	-3	4	134	309
142	-1.89	0.65	1.0000	
24	2	-4	12	348
-603	2.70	-1.21	1.0000	
24	2	-4	305	349
381	2.70	-1.21	1.0000	
22	2	-4	381	309
286	-3.49	1.33	1.0000	
20	2	-4	1207	321
1866	4.24	-5.99	1.0000	
16	2	-4	624	213
883	-5.79	-7.31	1.0000	
16	2	-4	265	214
402	-5.79	-7.31	1.0000	
14	2	-4	2516	250
1689	13.26	-13.10	1.0000	
14	2	-4	1863	213
1106	13.26	-13.10	1.0000	
12	2	-4	236	158
581	6.58	-3.75	1.0000	
12	2	-4	219	153
554	6.58	-3.75	1.0000	
10	2	-4	1551	223
2374	-1.18	8.57	1.0000	
10	2	-4	1492	211
2301	-1.18	8.57	1.0000	
8	2	-4	2058	285
5214	20.78	17.34	1.0000	
8	2	-4	1800	292
4780	20.78	17.34	1.0000	
-8	-2	4	1982	273
5071	20.78	-17.34	1.0000	
6	2	-4	674	169

1341	-13.80	0.10	1.0000	
6	2	-4	572	140
1183	-13.80	0.10	1.0000	
-6	-2	4	497	122
1075	-13.80	-0.10	1.0000	
4	2	-4	3840	152
1203	9.15	25.60	1.0000	
4	2	-4	3332	105
910	9.15	25.60	1.0000	
-4	-2	4	3254	136
871	9.15	-25.60	1.0000	
-4	-2	4	3463	95
989	9.15	-25.60	1.0000	
2	2	-4	5059	485
10783	-18.52	-36.62	1.0000	
-2	-2	4	5149	481
10971	-18.52	36.62	1.0000	
0	2	-4	3559	325
6599	-24.65	-1.42	1.0000	
0	-2	4	3501	313
6558	-24.65	1.42	1.0000	
-2	2	-4	5454	472
11467	-18.44	36.92	1.0000	
-2	2	-4	5198	493
11065	-18.44	36.92	1.0000	
-2	2	-4	5106	469
10919	-18.44	36.92	1.0000	
2	-2	4	5540	488
11556	-18.44	-36.92	1.0000	
2	-2	4	5403	468
11342	-18.44	-36.92	1.0000	
-4	2	-4	2872	105
1227	-2.00	-16.14	1.0000	
-4	2	-4	2912	107
1253	-2.00	-16.14	1.0000	
-4	2	-4	2757	131
1152	-2.00	-16.14	1.0000	
4	-2	4	3328	130
1532	-2.00	16.14	1.0000	
4	-2	4	3125	111
1395	-2.00	16.14	1.0000	
4	-2	4	3080	113
1365	-2.00	16.14	1.0000	
-6	2	-4	14590	439

9826	-17.96	55.39	1.0000	
-6	2	-4	14562	425
9798	-17.96	55.39	1.0000	
-6	2	-4	14806	417
10039	-17.96	55.39	1.0000	
6	-2	4	14591	442
9691	-17.96	-55.39	1.0000	
6	-2	4	14441	421
9544	-17.96	-55.39	1.0000	
-8	2	-4	13003	241
4681	27.21	30.08	1.0000	
8	-2	4	13292	275
4863	27.21	-30.08	1.0000	
-10	2	-4	7938	600
13876	-24.82	-1.67	1.0000	
10	-2	4	8002	641
13961	-24.82	1.67	1.0000	
-12	2	-4	2507	345
6980	6.25	29.46	1.0000	
12	-2	4	2737	410
7364	6.25	-29.46	1.0000	
-14	2	-4	1027	145
989	-6.58	-14.23	1.0000	
14	-2	4	1062	207
1037	-6.58	14.23	1.0000	
-16	2	-4	620	150
765	-2.25	-2.63	1.0000	
16	-2	4	546	213
682	-2.25	2.63	1.0000	
-18	2	-4	989	169
1085	0.78	1.25	1.0000	
18	-2	4	1085	246
1186	0.78	-1.25	1.0000	
-20	2	-4	239	143
202	0.55	-0.96	1.0000	
20	-2	4	482	242
429	0.55	0.96	1.0000	
22	-2	4	33	268
127	2.67	-4.08	1.0000	
24	-2	4	2	281
-254	0.29	-1.22	1.0000	
25	1	-4	81	364
27	-3.19	-1.36	1.0000	
23	1	-4	47	274

-165 3.07 5.07 1.0000
21 1 -4 101 278
113 -4.20 1.34 1.0000
21 1 -4 532 281
593 -4.20 1.34 1.0000
19 1 -4 1891 368
2036 2.12 1.05 1.0000
19 1 -4 2543 373
2712 2.12 1.05 1.0000
17 1 -4 3149 375
4276 -2.95 7.94 1.0000
17 1 -4 3968 407
5223 -2.95 7.94 1.0000
15 1 -4 2507 227
1321 11.46 3.21 1.0000
15 1 -4 2645 250
1422 11.46 3.21 1.0000
13 1 -4 1888 250
2749 -11.22 8.70 1.0000
13 1 -4 1705 258
2517 -11.22 8.70 1.0000
11 1 -4 2141 214
2104 -1.88 -5.89 1.0000
11 1 -4 1897 216
1859 -1.88 -5.89 1.0000
9 1 -4 415 105
-47 -12.73 -12.22 1.0000
9 1 -4 904 106
95 -12.73 -12.22 1.0000
-9 -1 4 415 104
-3 -12.73 12.22 1.0000
7 1 -4 18483 629
13798 5.38 -25.89 1.0000
7 1 -4 19083 616
14327 5.38 -25.89 1.0000
-7 -1 4 19709 613
14845 5.38 25.89 1.0000
5 1 -4 4193 190
2754 -10.64 -4.68 1.0000
5 1 -4 3838 153
2466 -10.64 -4.68 1.0000
-5 -1 4 3715 145
2372 -10.64 4.68 1.0000
3 1 -4 6112 473

10509	11.99	-17.41	1.0000	
-3	-1	4	6241	471
10676	11.99	17.41	1.0000	
1	1	-4	14278	485
10915	14.77	19.41	1.0000	
-1	-1	4	14611	484
11230	14.77	-19.41	1.0000	
-1	1	-4	13849	468
10586	14.63	-19.56	1.0000	
1	-1	4	14169	465
10856	14.63	19.56	1.0000	
-3	1	-4	2351	240
4607	21.52	0.44	1.0000	
-3	1	-4	2478	216
4790	21.52	0.44	1.0000	
-3	1	-4	2268	219
4486	21.52	0.44	1.0000	
3	-1	4	2178	226
4337	21.52	-0.44	1.0000	
3	-1	4	2535	219
4855	21.52	-0.44	1.0000	
-5	1	-4	6876	205
3808	-0.80	-25.64	1.0000	
-5	1	-4	7099	199
3981	-0.80	-25.64	1.0000	
-5	1	-4	6911	205
3835	-0.80	-25.64	1.0000	
5	-1	4	7147	212
4021	-0.80	25.64	1.0000	
5	-1	4	6918	197
3843	-0.80	25.64	1.0000	
-7	1	-4	1279	211
3305	-6.37	-35.65	1.0000	
-7	1	-4	1352	189
3472	-6.37	-35.65	1.0000	
7	-1	4	1322	218
3487	-6.37	35.65	1.0000	
-9	1	-4	12121	601
13667	-2.05	-5.54	1.0000	
9	-1	4	13001	639
14601	-2.05	5.54	1.0000	
-11	1	-4	2205	248
4534	-7.10	-16.26	1.0000	
11	-1	4	2274	302

4632	-7.10	16.26	1.0000	
-13	1	-4	2321	248
4379	14.83	-6.49	1.0000	
13	-1	4	2182	308
4179	14.83	6.49	1.0000	
-15	1	-4	1209	122
380	-3.13	12.87	1.0000	
15	-1	4	427	171
29	-3.13	-12.87	1.0000	
-17	1	-4	661	129
306	5.53	-5.90	1.0000	
17	-1	4	539	209
223	5.53	5.90	1.0000	
-19	1	-4	0	138
97	-3.65	7.85	1.0000	
19	-1	4	28	224
234	-3.65	-7.85	1.0000	
-21	1	-4	49	141
31	3.76	-0.64	1.0000	
21	-1	4	135	255
117	3.76	0.64	1.0000	
23	-1	4	12	279
159	-6.85	4.07	1.0000	
25	-1	4	33	297
-32	3.59	-3.48	1.0000	
24	0	-4	0	307
-32	-0.34	0.34	1.0000	
22	0	-4	28	275
105	4.53	-0.65	1.0000	
20	0	-4	661	248
467	0.90	3.73	1.0000	
20	0	-4	1095	272
841	0.90	3.73	1.0000	
18	0	-4	1122	263
1069	-4.01	-7.53	1.0000	
18	0	-4	905	241
847	-4.01	-7.53	1.0000	
16	0	-4	2971	327
3917	6.76	-7.25	1.0000	
16	0	-4	3422	364
4438	6.76	-7.25	1.0000	
14	0	-4	968	154
453	-6.96	6.61	1.0000	
14	0	-4	764	163

314 -6.96 6.61 1.0000
12 0 -4 2113 261
3652 -3.20 -12.14 1.0000
12 0 -4 1968 265
3461 -3.20 -12.14 1.0000
10 0 -4 82 182
1206 26.71 24.91 1.0000
10 0 -4 84 170
1196 26.71 24.91 1.0000
8 0 -4 1930 293
5227 -26.12 22.09 1.0000
8 0 -4 1854 276
5085 -26.12 22.09 1.0000
-8 0 4 1996 271
5353 -26.12 -22.09 1.0000
6 0 -4 19273 1193
27982 18.42 20.55 1.0000
6 0 -4 20123 1174
29008 18.42 20.55 1.0000
-6 0 4 20865 1172
29897 18.42 -20.55 1.0000
4 0 -4 6670 407
8883 -21.76 25.24 1.0000
-4 0 4 6488 405
8683 -21.76 -25.24 1.0000
2 0 -4 3819 333
7005 -24.68 1.38 1.0000
-2 0 4 3888 333
7059 -24.68 -1.38 1.0000
0 0 -4 24519 1067
25762 -3.40 0.01 1.0000
0 0 4 25354 1068
26618 -3.40 -0.01 1.0000
-2 0 -4 4677 367
8183 -24.67 1.36 1.0000
2 0 4 4764 364
8257 -24.67 -1.36 1.0000
-4 0 -4 5614 351
7580 -21.51 25.40 1.0000
-4 0 -4 5876 353
7918 -21.51 25.40 1.0000
-4 0 -4 6141 354
8257 -21.51 25.40 1.0000
4 0 4 6089 351

8221	-21.51	-25.40	1.0000	
4	0	4	5984	358
8086	-21.51	-25.40	1.0000	
-6	0	-4	19748	1167
28642	18.39	20.79	1.0000	
-6	0	-4	20365	1180
29388	18.39	20.79	1.0000	
6	0	4	19608	1182
28469	18.39	-20.79	1.0000	
-8	0	-4	1646	246
4699	-26.36	22.12	1.0000	
8	0	4	1913	285
5215	-26.36	-22.12	1.0000	
-10	0	-4	77	127
1246	26.89	24.91	1.0000	
10	0	4	100	184
1140	26.89	-24.91	1.0000	
-12	0	-4	2077	215
3600	-3.08	-12.14	1.0000	
12	0	4	2086	286
3612	-3.08	12.14	1.0000	
-14	0	-4	639	109
228	-7.10	7.06	1.0000	
14	0	4	133	158
-40	-7.10	-7.06	1.0000	
-16	0	-4	2366	233
3224	6.85	-7.37	1.0000	
16	0	4	2556	343
3456	6.85	7.37	1.0000	
-18	0	-4	791	159
725	-4.01	-7.70	1.0000	
18	0	4	890	249
827	-4.01	7.70	1.0000	
-20	0	-4	553	150
377	0.84	3.72	1.0000	
20	0	4	224	243
117	0.84	-3.72	1.0000	
-22	0	-4	124	163
256	4.40	-0.55	1.0000	
22	0	4	26	256
-187	4.40	0.55	1.0000	
24	0	4	104	296
113	-0.53	-0.21	1.0000	
25	-1	-4	139	342

282 3.62 3.44 1.0000
23 -1 -4 279 315
664 -6.86 -3.91 1.0000
23 -1 -4 83 289
-311 -6.86 -3.91 1.0000
21 -1 -4 19 244
-121 3.75 -0.65 1.0000
21 -1 -4 19 255
-64 3.75 -0.65 1.0000
19 -1 -4 9 222
162 -3.62 7.74 1.0000
19 -1 -4 270 256
690 -3.62 7.74 1.0000
17 -1 -4 1152 195
662 5.58 -6.01 1.0000
15 -1 -4 1688 165
673 -3.32 12.73 1.0000
15 -1 -4 1640 174
643 -3.32 12.73 1.0000
13 -1 -4 2289 302
4335 14.77 -6.58 1.0000
13 -1 -4 2346 295
4413 14.77 -6.58 1.0000
11 -1 -4 2291 284
4662 -7.08 -16.30 1.0000
11 -1 -4 2045 284
4308 -7.08 -16.30 1.0000
9 -1 -4 11908 584
13444 -2.20 -5.50 1.0000
9 -1 -4 11373 591
12875 -2.20 -5.50 1.0000
-9 1 4 11422 578
12927 -2.20 5.50 1.0000
7 -1 -4 1367 201
3501 -6.51 -35.50 1.0000
7 -1 -4 1317 226
3389 -6.51 -35.50 1.0000
-7 1 4 1265 194
3352 -6.51 35.50 1.0000
5 -1 -4 7546 248
4350 -0.77 -25.48 1.0000
5 -1 -4 7737 216
4501 -0.77 -25.48 1.0000
-5 1 4 7770 213

4530	-0.77	25.48	1.0000	
3	-1	-4	2116	233
4275	21.61	0.43	1.0000	
-3	1	4	1993	226
4075	21.61	-0.43	1.0000	
1	-1	-4	13486	452
10241	14.69	-19.38	1.0000	
-1	1	4	13540	450
10273	14.69	19.38	1.0000	
-1	-1	-4	13438	446
10211	14.58	19.45	1.0000	
1	1	4	13616	445
10386	14.58	-19.45	1.0000	
-3	-1	-4	5655	419
9924	12.30	-17.26	1.0000	
-3	-1	-4	5652	423
9920	12.30	-17.26	1.0000	
-3	-1	-4	5379	417
9558	12.30	-17.26	1.0000	
3	1	4	5632	423
9892	12.30	17.26	1.0000	
3	1	4	5587	421
9832	12.30	17.26	1.0000	
-5	-1	-4	3339	135
2022	-10.88	-5.05	1.0000	
-5	-1	-4	3155	146
1879	-10.88	-5.05	1.0000	
5	1	4	3426	156
2094	-10.88	5.05	1.0000	
5	1	4	3554	130
2195	-10.88	5.05	1.0000	
-7	-1	-4	19073	640
14246	5.67	-25.86	1.0000	
-7	-1	-4	20774	667
15747	5.67	-25.86	1.0000	
7	1	4	21232	674
16116	5.67	25.86	1.0000	
-9	-1	-4	1103	85
163	-12.75	-12.36	1.0000	
9	1	4	525	115
6	-12.75	12.36	1.0000	
-11	-1	-4	2191	160
2175	-1.64	-5.82	1.0000	
11	1	4	1832	220

1811 -1.64 5.82 1.0000
-13 -1 -4 1790 180
2614 -11.12 8.71 1.0000
13 1 4 1463 253
2187 -11.12 -8.71 1.0000
-15 -1 -4 2174 151
1086 11.38 3.35 1.0000
15 1 4 1457 210
600 11.38 -3.35 1.0000
-17 -1 -4 2182 243
3153 -2.89 8.10 1.0000
17 1 4 2536 372
3576 -2.89 -8.10 1.0000
-19 -1 -4 1716 209
1850 2.17 1.26 1.0000
19 1 4 1965 348
2106 2.17 -1.26 1.0000
-21 -1 -4 468 164
532 -4.28 1.21 1.0000
21 1 4 488 273
552 -4.28 -1.21 1.0000
23 1 4 1 282
56 3.15 -5.10 1.0000
25 1 4 223 331
127 -3.04 1.39 1.0000
24 -2 -4 2 309
-142 0.25 1.23 1.0000
22 -2 -4 31 270
-27 2.49 4.17 1.0000
22 -2 -4 31 259
-326 2.49 4.17 1.0000
20 -2 -4 63 216
44 0.69 -0.91 1.0000
20 -2 -4 221 242
184 0.69 -0.91 1.0000
18 -2 -4 1068 245
1160 0.87 1.15 1.0000
18 -2 -4 1803 312
1922 0.87 1.15 1.0000
16 -2 -4 1424 250
1652 -2.26 -2.72 1.0000
16 -2 -4 1604 279
1846 -2.26 -2.72 1.0000
14 -2 -4 1957 234

2029	-7.01	-14.38	1.0000	
14	-2	-4	1807	237
1861	-7.01	-14.38	1.0000	
12	-2	-4	2708	392
7326	6.39	29.45	1.0000	
12	-2	-4	2587	388
7124	6.39	29.45	1.0000	
10	-2	-4	8958	663
15200	-24.75	-1.98	1.0000	
10	-2	-4	8477	665
14571	-24.75	-1.98	1.0000	
8	-2	-4	13782	272
5147	27.10	30.20	1.0000	
8	-2	-4	13719	286
5108	27.10	30.20	1.0000	
-8	2	4	13425	266
4935	27.10	-30.20	1.0000	
6	-2	-4	14560	430
9784	-17.99	55.27	1.0000	
6	-2	-4	14556	455
9780	-17.99	55.27	1.0000	
-6	2	4	14839	430
9922	-17.99	-55.27	1.0000	
4	-2	-4	2609	93
1053	-2.01	-16.19	1.0000	
4	-2	-4	2704	135
1114	-2.01	-16.19	1.0000	
-4	2	4	2935	100
1265	-2.01	16.19	1.0000	
-4	2	4	2650	129
1080	-2.01	16.19	1.0000	
2	-2	-4	5604	511
11668	-18.45	36.62	1.0000	
-2	2	4	5597	507
11612	-18.45	-36.62	1.0000	
0	-2	-4	4805	377
8325	-24.70	-1.41	1.0000	
0	2	4	4805	375
8368	-24.70	1.41	1.0000	
-2	-2	-4	5594	490
11617	-18.35	-36.90	1.0000	
-2	-2	-4	5686	480
11759	-18.35	-36.90	1.0000	
2	2	4	5300	488

11202	-18.35	36.90	1.0000	
2	2	4	5376	483
11321	-18.35	36.90	1.0000	
-4	-2	-4	4172	106
1394	9.23	25.75	1.0000	
-4	-2	-4	3663	105
1091	9.23	25.75	1.0000	
-4	-2	-4	3955	118
1263	9.23	25.75	1.0000	
4	2	4	3815	130
1186	9.23	-25.75	1.0000	
-6	-2	-4	620	114
1267	-13.79	-0.11	1.0000	
-6	-2	-4	554	142
1164	-13.79	-0.11	1.0000	
6	2	4	688	160
1385	-13.79	0.11	1.0000	
-8	-2	-4	1521	233
4306	20.90	17.36	1.0000	
8	2	4	1946	285
5023	20.90	-17.36	1.0000	
-10	-2	-4	1212	147
1942	-1.55	8.57	1.0000	
10	2	4	1071	214
1763	-1.55	-8.57	1.0000	
-12	-2	-4	491	126
946	6.54	-3.56	1.0000	
12	2	4	229	174
563	6.54	3.56	1.0000	
-14	-2	-4	2040	159
1258	13.33	-13.10	1.0000	
14	2	4	1776	222
1022	13.33	13.10	1.0000	
-16	-2	-4	457	149
661	-5.70	-7.20	1.0000	
16	2	4	212	209
317	-5.70	7.20	1.0000	
-18	-2	-4	11	134
-152	2.88	-0.49	1.0000	
18	2	4	11	205
-161	2.88	0.49	1.0000	
-20	-2	-4	1310	222
2012	4.26	-6.20	1.0000	
20	2	4	1748	380

2548 4.26 6.20 1.0000
-22 -2 -4 20 154
-37 -3.60 1.32 1.0000
22 2 4 287 281
196 -3.60 -1.32 1.0000
24 2 4 12 333
-83 2.72 1.19 1.0000
25 -3 -4 160 334
168 -1.81 -0.57 1.0000
23 -3 -4 40 292
-3 5.42 0.76 1.0000
21 -3 -4 82 235
-207 -7.41 -2.58 1.0000
21 -3 -4 82 238
-71 -7.41 -2.58 1.0000
19 -3 -4 988 210
428 4.23 -8.31 1.0000
19 -3 -4 927 231
388 4.23 -8.31 1.0000
17 -3 -4 1103 283
1798 0.48 10.38 1.0000
17 -3 -4 1112 291
1810 0.48 10.38 1.0000
15 -3 -4 848 233
1474 5.80 -5.70 1.0000
15 -3 -4 1098 262
1799 5.80 -5.70 1.0000
11 -3 -4 4246 305
5053 12.82 6.48 1.0000
11 -3 -4 4221 304
5026 12.82 6.48 1.0000
9 -3 -4 1449 375
7134 -5.41 -40.18 1.0000
9 -3 -4 1569 366
7399 -5.41 -40.18 1.0000
7 -3 -4 1786 242
3631 -6.95 -15.95 1.0000
7 -3 -4 2155 225
4158 -6.95 -15.95 1.0000
-7 3 4 1989 223
3923 -6.95 15.95 1.0000
5 -3 -4 1238 104
959 4.19 -6.87 1.0000
5 -3 -4 1057 133

795	4.19	-6.87	1.0000	
-5	3	4	1408	115
1124	4.19	6.87	1.0000	
3	-3	-4	17478	412
8863	-5.06	-32.97	1.0000	
3	-3	-4	17730	383
9043	-5.06	-32.97	1.0000	
-3	3	4	17703	391
9025	-5.06	32.97	1.0000	
1	-3	-4	5060	379
9103	12.15	17.23	1.0000	
1	-3	-4	4501	373
8348	12.15	17.23	1.0000	
1	-3	-4	5136	404
9205	12.15	17.23	1.0000	
-1	-3	-4	1791	198
3789	21.77	-0.43	1.0000	
-1	-3	-4	2229	211
4447	21.77	-0.43	1.0000	
1	3	4	1420	193
3224	21.77	0.43	1.0000	
-3	-3	-4	17744	379
9069	-5.18	32.87	1.0000	
-3	-3	-4	16796	380
8394	-5.18	32.87	1.0000	
3	3	4	16666	385
8301	-5.18	-32.87	1.0000	
-5	-3	-4	2993	277
5565	4.13	-22.47	1.0000	
-5	-3	-4	2760	245
5233	4.13	-22.47	1.0000	
-5	-3	-4	2731	253
5191	4.13	-22.47	1.0000	
5	3	4	2774	277
5258	4.13	22.47	1.0000	
-7	-3	-4	3157	232
4659	-22.15	11.04	1.0000	
-7	-3	-4	2914	261
4334	-22.15	11.04	1.0000	
7	3	4	3088	275
4566	-22.15	-11.04	1.0000	
-9	-3	-4	4016	149
2075	-12.33	-19.95	1.0000	
9	3	4	3820	214

1908 -12.33 19.95 1.0000
-11 -3 -4 1656 130
1087 -7.59 2.77 1.0000
11 3 4 1975 204
1356 -7.59 -2.77 1.0000
-13 -3 -4 174 141
930 3.15 14.71 1.0000
13 3 4 72 180
664 3.15 -14.71 1.0000
-15 -3 -4 310 145
786 -10.28 3.39 1.0000
15 3 4 126 209
455 -10.28 -3.39 1.0000
-17 -3 -4 964 186
1215 4.55 12.56 1.0000
17 3 4 829 265
1055 4.55 -12.56 1.0000
-19 -3 -4 687 154
486 -2.15 3.61 1.0000
19 3 4 690 281
488 -2.15 -3.61 1.0000
-21 -3 -4 102 154
67 -1.37 1.45 1.0000
21 3 4 5 274
-119 -1.37 -1.45 1.0000
-23 -3 -4 46 180
82 -1.45 1.38 1.0000
23 3 4 155 313
217 -1.45 -1.38 1.0000
25 3 4 8 352
-56 -1.13 -2.10 1.0000
24 -4 -4 13 295
-344 2.11 2.39 1.0000
24 -4 -4 13 285
-214 2.11 2.39 1.0000
22 -4 -4 10 249
-282 0.21 2.71 1.0000
22 -4 -4 10 259
-46 0.21 2.71 1.0000
20 -4 -4 480 232
516 -0.11 1.96 1.0000
20 -4 -4 576 256
616 -0.11 1.96 1.0000
18 -4 -4 228 198

267 2.69 0.94 1.0000
18 -4 -4 446 229
503 2.69 0.94 1.0000
16 -4 -4 697 257
1339 -10.58 -1.64 1.0000
14 -4 -4 798 209
1178 3.65 5.65 1.0000
14 -4 -4 422 171
701 3.65 5.65 1.0000
12 -4 -4 2536 215
1837 -18.59 -16.15 1.0000
12 -4 -4 2673 217
1978 -18.59 -16.15 1.0000
10 -4 -4 2337 223
2727 13.29 31.96 1.0000
10 -4 -4 2360 215
2762 13.29 31.96 1.0000
8 -4 -4 3688 117
658 -14.04 27.06 1.0000
8 -4 -4 3690 126
659 -14.04 27.06 1.0000
-8 4 4 3193 123
459 -14.04 -27.06 1.0000
6 -4 -4 10180 557
13062 8.43 -8.32 1.0000
6 -4 -4 9851 575
12689 8.43 -8.32 1.0000
-6 4 4 9917 560
12763 8.43 8.32 1.0000
4 -4 -4 1004 489
10566 -12.67 61.71 1.0000
4 -4 -4 1157 468
11065 -12.67 61.71 1.0000
-4 4 4 1086 474
10836 -12.67 -61.71 1.0000
2 -4 -4 3659 131
1086 9.23 -25.86 1.0000
2 -4 -4 3853 108
1200 9.23 -25.86 1.0000
2 -4 -4 3483 107
985 9.23 -25.86 1.0000
0 -4 -4 5558 340
7534 -21.57 -25.40 1.0000
0 -4 -4 5888 358

7960	-21.57	-25.40	1.0000	
0	4	4	5617	341
7582	-21.57	25.40	1.0000	
-2	-4	-4	2210	126
802	-2.01	16.24	1.0000	
-2	-4	-4	2764	116
1149	-2.01	16.24	1.0000	
2	4	4	2262	129
833	-2.01	-16.24	1.0000	
-4	-4	-4	1315	499
11569	-12.76	-61.74	1.0000	
-4	-4	-4	1099	480
10888	-12.76	-61.74	1.0000	
4	4	4	1234	500
11320	-12.76	61.74	1.0000	
-6	-4	-4	3963	187
2072	7.45	17.05	1.0000	
-6	-4	-4	4146	144
2208	7.45	17.05	1.0000	
6	4	4	4111	189
2176	7.45	-17.05	1.0000	
-8	-4	-4	1088	172
2721	16.61	1.83	1.0000	
8	4	4	1186	227
2874	16.61	-1.83	1.0000	
-10	-4	-4	2147	202
3495	12.92	-9.43	1.0000	
10	4	4	2192	276
3550	12.92	9.43	1.0000	
-12	-4	-4	106	90
-16	5.39	7.12	1.0000	
12	4	4	147	158
206	5.39	-7.12	1.0000	
-14	-4	-4	501	130
368	5.39	-16.12	1.0000	
14	4	4	422	211
225	5.39	16.12	1.0000	
-16	-4	-4	723	131
471	2.58	-7.14	1.0000	
16	4	4	77	229
-177	2.58	7.14	1.0000	
-18	-4	-4	485	162
330	3.18	-8.69	1.0000	
18	4	4	147	244

20 3.18 8.69 1.0000
-20 -4 -4 425 171
472 -1.55 0.20 1.0000
20 4 4 614 310
671 -1.55 -0.20 1.0000
-22 -4 -4 245 183
252 2.89 -1.84 1.0000
22 4 4 38 288
30 2.89 1.84 1.0000
24 4 4 223 332
185 0.53 1.27 1.0000
25 -5 -4 2 326
-133 0.64 -1.19 1.0000
25 -5 -4 43 329
41 0.64 -1.19 1.0000
23 -5 -4 22 281
124 -5.03 -4.97 1.0000
23 -5 -4 343 306
667 -5.03 -4.97 1.0000
21 -5 -4 49 239
-84 6.01 -0.80 1.0000
21 -5 -4 37 225
1 6.01 -0.80 1.0000
19 -5 -4 1224 226
981 -3.58 -0.20 1.0000
19 -5 -4 1034 256
812 -3.58 -0.20 1.0000
17 -5 -4 324 220
778 0.95 -8.56 1.0000
17 -5 -4 86 187
368 0.95 -8.56 1.0000
15 -5 -4 290 180
239 7.62 1.81 1.0000
15 -5 -4 225 160
169 7.62 1.81 1.0000
13 -5 -4 2598 278
3861 10.45 9.27 1.0000
13 -5 -4 2052 281
3173 10.45 9.27 1.0000
11 -5 -4 4675 246
3235 -9.51 -19.52 1.0000
11 -5 -4 4742 254
3295 -9.51 -19.52 1.0000
9 -5 -4 1329 185

1682		7.24	1.31	1.0000
9	-5	-4	1663	197
2061		7.24	1.31	1.0000
7	-5	-4	271	127
599		-10.77	-9.12	1.0000
7	-5	-4	221	111
494		-10.77	-9.12	1.0000
-7	5	4	245	128
521		-10.77	9.12	1.0000
5	-5	-4	8187	163
2321		-8.84	-35.81	1.0000
5	-5	-4	7755	181
2093		-8.84	-35.81	1.0000
-5	5	4	7996	180
2220		-8.84	35.81	1.0000
3	-5	-4	2512	260
4912		4.02	22.65	1.0000
3	-5	-4	2375	239
4711		4.02	22.65	1.0000
-3	5	4	2165	256
4394		4.02	-22.65	1.0000
1	-5	-4	3708	163
2347		-10.73	4.82	1.0000
1	-5	-4	3488	150
2172		-10.73	4.82	1.0000
-1	-5	-4	6661	205
3657		-0.77	25.52	1.0000
1	5	4	6421	205
3470		-0.77	-25.52	1.0000
-3	-5	-4	1466	144
1193		4.37	6.80	1.0000
3	5	4	1370	145
1094		4.37	-6.80	1.0000
-5	-5	-4	8139	145
2291		-8.69	35.86	1.0000
-5	-5	-4	7424	190
1919		-8.69	35.86	1.0000
5	5	4	8063	195
2250		-8.69	-35.86	1.0000
-7	-5	-4	806	107
755		-14.26	-16.06	1.0000
7	5	4	722	148
643		-14.26	16.06	1.0000
-9	-5	-4	1225	88

319	-13.96	9.51	1.0000	
9	5	4	1293	144
358	-13.96	-9.51	1.0000	
-11	-5	-4	2109	140
1398	-11.51	3.44	1.0000	
11	5	4	1969	225
1282	-11.51	-3.44	1.0000	
-13	-5	-4	1475	157
1547	-7.59	-6.67	1.0000	
13	5	4	1515	280
1594	-7.59	6.67	1.0000	
-15	-5	-4	14	141
594	6.03	22.78	1.0000	
15	5	4	48	220
427	6.03	-22.78	1.0000	
-17	-5	-4	342	148
287	-3.35	0.64	1.0000	
17	5	4	438	260
379	-3.35	-0.64	1.0000	
-19	-5	-4	496	167
671	-0.78	3.85	1.0000	
19	5	4	550	285
735	-0.78	-3.85	1.0000	
-21	-5	-4	65	174
73	0.73	4.39	1.0000	
21	5	4	26	277
-121	0.73	-4.39	1.0000	
-23	-5	-4	364	206
406	-4.45	-1.20	1.0000	
23	5	4	28	289
-382	-4.45	1.20	1.0000	
25	5	4	42	323
44	-0.87	-0.55	1.0000	
24	-6	-4	5	293
23	2.43	2.89	1.0000	
24	-6	-4	19	284
-419	2.43	2.89	1.0000	
22	-6	-4	7	255
-244	1.63	1.62	1.0000	
22	-6	-4	7	244
-13	1.63	1.62	1.0000	
20	-6	-4	30	210
-38	2.83	3.79	1.0000	
20	-6	-4	180	241

```
166      2.83      3.79  1.0000
  18   -6   -4       193       208
67    -7.09      1.79  1.0000
  18   -6   -4       403       196
231    -7.09      1.79  1.0000
  16   -6   -4       834       261
1262      4.34    -3.87  1.0000
  16   -6   -4      1168       280
1667      4.34    -3.87  1.0000
  14   -6   -4       257       209
1115   -18.23    -3.44  1.0000
  14   -6   -4       325       228
1272   -18.23    -3.44  1.0000
  12   -6   -4       206       147
326      6.18      4.06  1.0000
  10   -6   -4       399       126
244    -5.57    -4.26  1.0000
   8   -6   -4       145       117
384      3.49      8.48  1.0000
   8   -6   -4       138       119
370      3.49      8.48  1.0000
   6   -6   -4      1835       296
5834      9.13     34.06  1.0000
   6   -6   -4      1506       308
5178      9.13     34.06  1.0000
  -6    6    4      1774       312
5725      9.13    -34.06  1.0000
   4   -6   -4      3930       160
2005      7.52    -17.44  1.0000
  -4    6    4      3836       184
1942      7.52     17.44  1.0000
   2   -6   -4       672       144
1348   -13.72    -0.04  1.0000
   2   -6   -4       689       145
1375   -13.72    -0.04  1.0000
   0   -6   -4     19292      1147
28080     18.47   -20.75  1.0000
  -2   -6   -4     14320       427
9432   -17.94   -55.40  1.0000
   2    6    4     13800       423
9052   -17.94    55.40  1.0000
  -4   -6   -4      9081       545
11783      8.51      8.12  1.0000
   4    6    4      9403       542
```

12149 8.51 -8.12 1.0000
-6 -6 -4 1928 290
6039 8.98 -34.12 1.0000
6 6 4 2015 340
6192 8.98 34.12 1.0000
-8 -6 -4 1879 169
2560 2.41 -8.11 1.0000
8 6 4 1875 234
2558 2.41 8.11 1.0000
-10 -6 -4 846 83
63 11.14 14.86 1.0000
10 6 4 459 132
-26 11.14 -14.86 1.0000
-12 -6 -4 1566 183
2414 18.52 -17.65 1.0000
12 6 4 1712 271
2644 18.52 17.65 1.0000
-14 -6 -4 644 160
1047 -5.56 6.33 1.0000
14 6 4 659 233
1062 -5.56 -6.33 1.0000
-16 -6 -4 1772 226
2957 7.17 -11.39 1.0000
16 6 4 1746 373
2925 7.17 11.39 1.0000
-18 -6 -4 1007 186
1030 -3.59 -4.42 1.0000
18 6 4 1472 309
1507 -3.59 4.42 1.0000
-20 -6 -4 776 195
955 -2.73 0.01 1.0000
20 6 4 405 282
537 -2.73 -0.01 1.0000
-22 -6 -4 120 183
182 2.68 -5.05 1.0000
22 6 4 130 282
197 2.68 5.05 1.0000
24 6 4 220 324
320 -0.26 -2.67 1.0000
23 -7 -4 8 291
-180 0.30 -2.37 1.0000
23 -7 -4 8 265
-16 0.30 -2.37 1.0000
21 -7 -4 66 235

-62 -4.35 -5.56 1.0000
21 -7 -4 86 262
84 -4.35 -5.56 1.0000
19 -7 -4 772 243
957 1.60 -2.60 1.0000
19 -7 -4 843 264
1037 1.60 -2.60 1.0000
17 -7 -4 180 216
276 2.65 -1.20 1.0000
17 -7 -4 12 183
43 2.65 -1.20 1.0000
15 -7 -4 977 189
597 5.06 3.99 1.0000
15 -7 -4 909 177
544 5.06 3.99 1.0000
13 -7 -4 230 152
248 -3.22 -0.69 1.0000
13 -7 -4 118 146
127 -3.22 -0.69 1.0000
11 -7 -4 2602 270
3364 11.37 11.32 1.0000
11 -7 -4 2840 266
3648 11.37 11.32 1.0000
9 -7 -4 3392 222
2739 -13.27 -2.55 1.0000
9 -7 -4 3612 236
2944 -13.27 -2.55 1.0000
7 -7 -4 9484 583
12917 -5.85 -16.17 1.0000
5 -7 -4 621 114
460 -14.21 16.02 1.0000
-5 7 4 567 132
320 -14.21 -16.02 1.0000
3 -7 -4 2889 252
4305 -22.19 -11.07 1.0000
3 -7 -4 3104 256
4593 -22.19 -11.07 1.0000
1 -7 -4 17737 583
13073 5.55 25.83 1.0000
1 -7 -4 18514 599
13754 5.55 25.83 1.0000
-1 -7 -4 1497 231
3868 -6.37 35.61 1.0000
1 7 4 1258 228

3252 -6.37 -35.61 1.0000
-3 -7 -4 2839 281
5112 -7.06 16.02 1.0000
3 7 4 1888 267
3788 -7.06 -16.02 1.0000
-7 -7 -4 9197 539
12582 -5.89 16.11 1.0000
-7 -7 -4 8667 527
11957 -5.89 16.11 1.0000
7 7 4 9816 596
13307 -5.89 -16.11 1.0000
-9 -7 -4 2877 208
3795 -12.40 -5.06 1.0000
9 7 4 2418 277
3246 -12.40 5.06 1.0000
-11 -7 -4 1077 148
1518 -4.92 -4.84 1.0000
11 7 4 970 233
1393 -4.92 4.84 1.0000
-13 -7 -4 641 161
1539 -5.71 10.81 1.0000
13 7 4 635 245
1527 -5.71 -10.81 1.0000
-15 -7 -4 556 133
241 -1.23 -6.88 1.0000
15 7 4 65 205
-121 -1.23 6.88 1.0000
-17 -7 -4 841 197
1479 3.11 8.57 1.0000
-19 -7 -4 319 167
357 0.37 3.05 1.0000
19 7 4 75 259
90 0.37 -3.05 1.0000
-21 -7 -4 16 173
17 -2.75 -1.73 1.0000
21 7 4 33 268
43 -2.75 1.73 1.0000
-23 -7 -4 27 198
55 0.98 2.29 1.0000
23 7 4 8 294
-6 0.98 -2.29 1.0000
24 -8 -4 213 344
267 -0.23 1.62 1.0000
24 -8 -4 4 292

```
-199    -0.23     1.62  1.0000
  22   -8  -4       89       261
216     3.77     2.61  1.0000
  22   -8  -4        1       273
34     3.77     2.61  1.0000
  20   -8  -4        8       229
-30    -0.51     2.37  1.0000
  20   -8  -4      416       249
351    -0.51     2.37  1.0000
  18   -8  -4      423       218
558     3.50     0.99  1.0000
  18   -8  -4      337       236
458     3.50     0.99  1.0000
  16   -8  -4      654       218
661   -11.55     1.94  1.0000
  16   -8  -4      625       198
627   -11.55     1.94  1.0000
  14   -8  -4      719       260
1857     5.18   -13.50  1.0000
  14   -8  -4      736       264
1885     5.18   -13.50  1.0000
   8   -8  -4      930       186
1585    14.05    -2.47  1.0000
   6   -8  -4     2296       218
3046     2.37     8.06  1.0000
   4   -8  -4     1167       211
2856    16.71    -1.91  1.0000
   2   -8  -4     1278       251
3858    20.86   -17.46  1.0000
   2   -8  -4     1526       262
4310    20.86   -17.46  1.0000
   0   -8  -4     1449       270
4315   -26.22   -22.23  1.0000
   2    8   4    12359       267
4286    27.31    30.10  1.0000
   4    8   4     3933       164
752   -14.14    27.21  1.0000
  -6   -8  -4      391       110
774     3.65    -8.42  1.0000
  -6   -8  -4      160        82
406     3.65    -8.42  1.0000
  -8   -8  -4      938       143
1549    13.86     2.18  1.0000
  -8   -8  -4      870       128
```

1453	13.86	2.18	1.0000	
8	8	4	1130	228
1829	13.86	-2.18	1.0000	
-10	-8	-4	299	90
85	5.11	-7.34	1.0000	
10	8	4	721	176
383	5.11	7.34	1.0000	
-12	-8	-4	774	118
339	-3.91	14.91	1.0000	
12	8	4	839	200
386	-3.91	-14.91	1.0000	
-14	-8	-4	712	162
1118	14.38	-7.25	1.0000	
14	8	4	495	247
778	14.38	7.25	1.0000	
-16	-8	-4	3737	213
2226	-12.00	1.94	1.0000	
16	8	4	3775	375
2254	-12.00	-1.94	1.0000	
-18	-8	-4	253	166
255	0.16	0.23	1.0000	
18	8	4	277	254
280	0.16	-0.23	1.0000	
-20	-8	-4	178	188
626	4.59	-9.01	1.0000	
20	8	4	142	283
555	4.59	9.01	1.0000	
-22	-8	-4	379	200
123	-7.32	4.48	1.0000	
22	8	4	98	268
-138	-7.32	-4.48	1.0000	
24	8	4	20	297
16	-0.88	1.16	1.0000	
23	-9	-4	4	277
-12	1.66	0.22	1.0000	
23	-9	-4	4	292
-94	1.66	0.22	1.0000	
21	-9	-4	3	247
-164	-0.92	-1.20	1.0000	
21	-9	-4	3	252
-90	-0.92	-1.20	1.0000	
19	-9	-4	70	228
304	-3.32	-8.70	1.0000	
19	-9	-4	32	249

199 -3.32 -8.70 1.0000
17 -9 -4 415 220
511 5.25 6.26 1.0000
17 -9 -4 260 198
317 5.25 6.26 1.0000
15 -9 -4 6 171
68 -4.85 1.30 1.0000
15 -9 -4 11 181
82 -4.85 1.30 1.0000
13 -9 -4 166 183
450 5.39 7.29 1.0000
13 -9 -4 168 169
454 5.39 7.29 1.0000
11 -9 -4 653 137
199 -5.56 11.69 1.0000
9 -9 -4 2037 209
1847 -0.90 2.05 1.0000
7 -9 -4 3722 293
4803 -12.64 5.17 1.0000
5 -9 -4 1443 124
444 -13.88 -9.55 1.0000
3 -9 -4 4456 206
2403 -12.31 19.87 1.0000
1 -9 -4 842 113
74 -12.75 12.35 1.0000
1 9 4 12570 636
14182 -2.23 -5.62 1.0000
3 9 4 1399 372
7028 -5.32 -40.21 1.0000
5 9 4 1289 221
1651 7.22 1.51 1.0000
-7 -9 -4 3295 172
2655 -13.28 2.52 1.0000
-7 -9 -4 2990 169
2372 -13.28 2.52 1.0000
7 9 4 3647 254
2973 -13.28 -2.52 1.0000
-9 -9 -4 1856 148
1682 -1.06 -2.00 1.0000
-9 -9 -4 1943 152
1765 -1.06 -2.00 1.0000
9 9 4 1621 236
1459 -1.06 2.00 1.0000
-11 -9 -4 1054 177

2041	-8.85	-8.77	1.0000	
11	9	4	1466	306
2603	-8.85	8.77	1.0000	
-13	-9	-4	895	141
765	3.63	-4.57	1.0000	
13	9	4	1108	244
964	3.63	4.57	1.0000	
-15	-9	-4	574	161
544	2.30	0.32	1.0000	
15	9	4	7	231
-150	2.30	-0.32	1.0000	
-17	-9	-4	399	163
537	0.80	-2.87	1.0000	
17	9	4	259	272
372	0.80	2.87	1.0000	
-19	-9	-4	829	192
751	-1.17	1.81	1.0000	
19	9	4	566	297
502	-1.17	-1.81	1.0000	
-21	-9	-4	16	184
-205	0.19	3.49	1.0000	
21	9	4	44	292
25	0.19	-3.49	1.0000	
-23	-9	-4	235	231
449	2.76	-4.86	1.0000	
23	9	4	294	353
530	2.76	4.86	1.0000	
22	-10	-4	20	281
14	-2.83	1.54	1.0000	
22	-10	-4	14	264
-229	-2.83	1.54	1.0000	
20	-10	-4	812	268
391	5.50	5.19	1.0000	
20	-10	-4	919	247
466	5.50	5.19	1.0000	
18	-10	-4	130	207
51	-2.87	-2.57	1.0000	
18	-10	-4	458	233
295	-2.87	-2.57	1.0000	
16	-10	-4	293	189
142	1.83	-6.21	1.0000	
16	-10	-4	251	203
111	1.83	-6.21	1.0000	
14	-10	-4	326	176

435 -2.05 1.80 1.0000
14 -10 -4 277 197
378 -2.05 1.80 1.0000
12 -10 -4 259 169
231 -3.93 -18.48 1.0000
10 -10 -4 2600 237
2298 8.18 -8.65 1.0000
8 -10 -4 286 124
79 5.00 7.20 1.0000
6 -10 -4 1165 120
165 11.34 -14.85 1.0000
4 -10 -4 2335 261
3723 12.81 9.33 1.0000
2 -10 -4 1273 214
2029 -1.45 -8.62 1.0000
0 -10 -4 40 232
1975 26.92 -25.14 1.0000
2 10 4 8251 653
14271 -24.75 -1.73 1.0000
4 10 4 2405 240
2823 13.44 32.00 1.0000
-6 -10 -4 502 100
331 -5.47 4.21 1.0000
-8 -10 -4 398 109
436 -4.15 2.60 1.0000
8 10 4 689 179
745 -4.15 -2.60 1.0000
-10 -10 -4 2621 186
2332 7.96 8.55 1.0000
-10 -10 -4 2643 177
2354 7.96 8.55 1.0000
10 10 4 2959 281
2662 7.96 -8.55 1.0000
-12 -10 -4 2434 182
1818 7.59 7.24 1.0000
12 10 4 2745 320
2103 7.59 -7.24 1.0000
-14 -10 -4 420 185
1364 -14.82 5.87 1.0000
14 10 4 351 308
1238 -14.82 -5.87 1.0000
-16 -10 -4 520 161
369 5.25 2.90 1.0000
16 10 4 511 263

359	5.25	-2.90	1.0000	
-18	-10	-4	363	187
526	-3.62	-2.39	1.0000	
18	10	4	106	288
194	-3.62	2.39	1.0000	
-20	-10	-4	6	190
44	-7.66	1.05	1.0000	
20	10	4	48	287
247	-7.66	-1.05	1.0000	
-22	-10	-4	333	241
489	2.63	-2.08	1.0000	
22	10	4	280	332
424	2.63	2.08	1.0000	
23	-11	-4	130	321
148	-0.64	-0.12	1.0000	
23	-11	-4	222	300
245	-0.64	-0.12	1.0000	
21	-11	-4	53	290
200	-0.22	-6.29	1.0000	
21	-11	-4	77	261
245	-0.22	-6.29	1.0000	
19	-11	-4	102	244
14	2.94	8.23	1.0000	
19	-11	-4	102	218
-89	2.94	8.23	1.0000	
17	-11	-4	252	220
719	-8.47	-6.25	1.0000	
17	-11	-4	122	235
473	-8.47	-6.25	1.0000	
15	-11	-4	643	212
776	2.77	3.98	1.0000	
15	-11	-4	566	228
689	2.77	3.98	1.0000	
13	-11	-4	578	273
1863	0.02	16.58	1.0000	
11	-11	-4	712	193
692	0.17	-0.34	1.0000	
9	-11	-4	1080	229
2101	-9.08	8.95	1.0000	
7	-11	-4	1296	215
1764	-4.75	4.73	1.0000	
5	-11	-4	2138	194
1419	-11.71	-3.45	1.0000	
3	-11	-4	1641	190

1078	-7.61	-2.79	1.0000	
1	-11	-4	1855	224
1831	-1.71	5.93	1.0000	
1	11	4	1949	287
4155	-7.15	-16.19	1.0000	
3	11	4	3912	318
4656	12.95	6.33	1.0000	
5	11	4	4623	268
3207	-9.73	-19.56	1.0000	
-7	-11	-4	2105	190
2740	11.35	-11.41	1.0000	
7	11	4	2308	285
3005	11.35	11.41	1.0000	
-9	-11	-4	987	124
424	-5.40	-11.86	1.0000	
9	11	4	817	199
315	-5.40	11.86	1.0000	
-11	-11	-4	816	134
802	0.13	0.20	1.0000	
11	11	4	560	204
548	0.13	-0.20	1.0000	
-13	-11	-4	1339	178
1568	-1.87	-15.37	1.0000	
13	11	4	1068	281
1239	-1.87	15.37	1.0000	
-15	-11	-4	915	187
951	1.52	-0.49	1.0000	
15	11	4	792	294
826	1.52	0.49	1.0000	
-17	-11	-4	396	166
391	0.31	-0.42	1.0000	
17	11	4	268	292
264	0.31	0.42	1.0000	
-19	-11	-4	49	188
10	3.80	-1.85	1.0000	
19	11	4	110	305
52	3.80	1.85	1.0000	
-21	-11	-4	853	235
866	2.91	-1.23	1.0000	
21	11	4	540	333
549	2.91	1.23	1.0000	
-23	-11	-4	427	251
477	0.39	0.96	1.0000	
23	11	4	80	334

103	0.39	-0.96	1.0000	
22	-12	-4	271	296
492	5.09	0.66	1.0000	
22	-12	-4	207	307
404	5.09	0.66	1.0000	
20	-12	-4	22	262
131	-5.19	-3.32	1.0000	
20	-12	-4	56	253
206	-5.19	-3.32	1.0000	
18	-12	-4	113	224
50	1.48	4.66	1.0000	
18	-12	-4	32	219
-71	1.48	4.66	1.0000	
16	-12	-4	677	206
346	8.33	-0.65	1.0000	
16	-12	-4	263	206
66	8.33	-0.65	1.0000	
14	-12	-4	794	195
90	-6.38	-14.94	1.0000	
12	-12	-4	638	200
591	-2.72	0.01	1.0000	
10	-12	-4	2331	256
1743	7.49	-7.21	1.0000	
8	-12	-4	889	157
433	-3.82	-14.84	1.0000	
6	-12	-4	1797	254
2755	18.49	17.50	1.0000	
4	-12	-4	248	153
352	5.44	-6.91	1.0000	
2	-12	-4	424	185
849	6.54	3.51	1.0000	
0	-12	-4	1938	298
3419	-3.18	12.13	1.0000	
2	12	4	2508	398
6999	6.34	29.52	1.0000	
4	12	4	2386	236
1676	-18.28	-15.89	1.0000	
-6	-12	-4	427	119
635	6.22	-4.31	1.0000	
-8	-12	-4	368	116
478	-4.39	4.90	1.0000	
8	12	4	113	201
148	-4.39	-4.90	1.0000	
-10	-12	-4	327	127

501 -3.82 18.66 1.0000
10 12 4 330 229
549 -3.82 -18.66 1.0000
-12 -12 -4 759 151
714 -2.68 -0.09 1.0000
12 12 4 798 245
752 -2.68 0.09 1.0000
-14 -12 -4 705 171
1128 5.21 10.67 1.0000
14 12 4 653 261
1045 5.21 -10.67 1.0000
-16 -12 -4 26 164
-6 -4.37 0.88 1.0000
16 12 4 26 245
-395 -4.37 -0.88 1.0000
-18 -12 -4 18 186
57 -3.03 -1.29 1.0000
18 12 4 41 299
96 -3.03 1.29 1.0000
-20 -12 -4 32 211
137 -3.48 4.16 1.0000
20 12 4 39 316
-204 -3.48 -4.16 1.0000
-22 -12 -4 12 255
86 -5.16 0.76 1.0000
22 12 4 36 343
-120 -5.16 -0.76 1.0000
21 -13 -4 203 282
451 1.52 6.04 1.0000
21 -13 -4 218 299
473 1.52 6.04 1.0000
19 -13 -4 215 248
230 -0.65 -7.13 1.0000
19 -13 -4 435 267
488 -0.65 -7.13 1.0000
17 -13 -4 353 223
285 -2.51 2.49 1.0000
17 -13 -4 347 236
279 -2.51 2.49 1.0000
15 -13 -4 420 221
573 -4.25 7.35 1.0000
13 -13 -4 2039 304
1879 1.14 -4.20 1.0000
11 -13 -4 1331 278

1574	-1.79	15.51	1.0000	
9	-13	-4	425	172
324	3.56	4.48	1.0000	
7	-13	-4	578	247
1432	-5.86	-10.62	1.0000	
5	-13	-4	2169	243
2297	-7.66	6.76	1.0000	
3	-13	-4	54	173
603	3.12	-14.68	1.0000	
1	-13	-4	1733	274
2541	-11.20	-8.73	1.0000	
3	13	4	589	172
357	0.32	-4.87	1.0000	
5	13	4	2355	310
3565	10.59	9.32	1.0000	
-7	-13	-4	356	128
380	-3.12	0.69	1.0000	
7	13	4	217	189
233	-3.12	-0.69	1.0000	
-9	-13	-4	204	129
515	5.24	-7.43	1.0000	
9	13	4	115	236
352	5.24	7.43	1.0000	
-11	-13	-4	422	178
1574	0.17	-16.60	1.0000	
11	13	4	441	279
1612	0.17	16.60	1.0000	
-13	-13	-4	2130	200
1959	1.10	4.25	1.0000	
13	13	4	2486	363
2303	1.10	-4.25	1.0000	
-15	-13	-4	112	183
366	-4.68	-7.98	1.0000	
15	13	4	53	236
238	-4.68	7.98	1.0000	
-17	-13	-4	1	170
-136	0.42	-0.89	1.0000	
17	13	4	107	245
130	0.42	0.89	1.0000	
-19	-13	-4	26	205
-144	2.43	3.74	1.0000	
19	13	4	26	283
-86	2.43	-3.74	1.0000	
-21	-13	-4	16	235

153	4.13	-6.08	1.0000	
21	13	4	72	334
-151	4.13	6.08	1.0000	
20	-14	-4	8	256
-71	2.49	-0.15	1.0000	
20	-14	-4	336	277
271	2.49	-0.15	1.0000	
18	-14	-4	529	263
671	1.94	-2.13	1.0000	
18	-14	-4	109	239
176	1.94	-2.13	1.0000	
16	-14	-4	1013	275
1050	-4.76	0.16	1.0000	
14	-14	-4	336	215
384	2.87	1.05	1.0000	
12	-14	-4	582	237
938	5.12	-10.54	1.0000	
10	-14	-4	285	234
1119	-15.02	-5.96	1.0000	
8	-14	-4	947	249
1455	14.33	7.38	1.0000	
6	-14	-4	405	200
718	-5.54	-6.24	1.0000	
4	-14	-4	507	175
355	5.34	15.93	1.0000	
2	-14	-4	2112	233
1314	13.29	12.95	1.0000	
0	-14	-4	651	187
229	-7.10	-6.78	1.0000	
2	14	4	1356	222
1362	-6.61	-14.12	1.0000	
4	14	4	300	213
546	3.56	5.81	1.0000	
6	14	4	128	211
739	-18.16	-3.33	1.0000	
-8	-14	-4	541	172
1559	5.21	13.43	1.0000	
8	14	4	596	269
1650	5.21	-13.43	1.0000	
-10	-14	-4	611	150
763	-1.99	-1.93	1.0000	
10	14	4	700	269
863	-1.99	1.93	1.0000	
-12	-14	-4	1239	152

271	-6.32	15.03	1.0000	
12	14	4	354	228
-106	-6.32	-15.03	1.0000	
-14	-14	-4	623	173
690	2.90	-1.08	1.0000	
14	14	4	401	273
454	2.90	1.08	1.0000	
-16	-14	-4	277	189
247	0.01	0.81	1.0000	
16	14	4	1	264
-4	0.01	-0.81	1.0000	
-18	-14	-4	278	206
377	-5.40	4.22	1.0000	
18	14	4	63	239
-46	-5.40	-4.22	1.0000	
-20	-14	-4	197	238
195	0.55	-0.77	1.0000	
20	14	4	160	282
158	0.55	0.77	1.0000	
21	-15	-4	66	304
109	-1.20	1.95	1.0000	
21	-15	-4	39	286
73	-1.20	1.95	1.0000	
19	-15	-4	253	263
457	-3.17	3.76	1.0000	
19	-15	-4	347	278
582	-3.17	3.76	1.0000	
17	-15	-4	156	246
144	2.85	-1.80	1.0000	
17	-15	-4	437	241
427	2.85	-1.80	1.0000	
15	-15	-4	114	220
187	-0.04	-2.74	1.0000	
13	-15	-4	38	215
192	-4.73	7.92	1.0000	
11	-15	-4	546	216
567	1.50	0.64	1.0000	
9	-15	-4	179	204
162	2.38	-0.23	1.0000	
7	-15	-4	66	175
-107	-1.44	6.88	1.0000	
5	-15	-4	8	198
660	6.01	-22.62	1.0000	
3	-15	-4	226	211

656	-10.36	-3.10	1.0000	
1	-15	-4	2015	243
978	11.33	-3.34	1.0000	
-1	-15	-4	1344	238
461	-3.14	-12.80	1.0000	
3	15	4	622	230
1160	5.60	-5.76	1.0000	
5	15	4	132	214
72	7.86	1.98	1.0000	
-7	-15	-4	693	136
375	5.12	-4.07	1.0000	
7	15	4	805	211
458	5.12	4.07	1.0000	
-9	-15	-4	0	131
36	-4.73	-1.30	1.0000	
9	15	4	92	221
232	-4.73	1.30	1.0000	
-11	-15	-4	823	164
978	2.57	-3.89	1.0000	
11	15	4	358	275
453	2.57	3.89	1.0000	
-13	-15	-4	647	169
845	-4.15	-7.32	1.0000	
13	15	4	271	239
372	-4.15	7.32	1.0000	
-15	-15	-4	290	191
404	-0.07	2.79	1.0000	
15	15	4	329	288
450	-0.07	-2.79	1.0000	
-17	-15	-4	322	196
382	3.28	1.69	1.0000	
17	15	4	100	295
129	3.28	-1.69	1.0000	
-19	-15	-4	313	232
698	-0.76	-7.66	1.0000	
19	15	4	124	328
394	-0.76	7.66	1.0000	
-21	-15	-4	28	261
124	0.41	5.94	1.0000	
21	15	4	47	276
-277	0.41	-5.94	1.0000	
20	-16	-4	35	259
15	0.75	-2.28	1.0000	
20	-16	-4	299	302

```
243     0.75   -2.28  1.0000
  18 -16  -4      149      237
155     2.14   -0.47  1.0000
  18 -16  -4        6      275
-20     2.14   -0.47  1.0000
  16 -16  -4       28      225
53    -4.21   -2.45  1.0000
  14 -16  -4      101      223
87     0.00   -0.63  1.0000
  12 -16  -4      128      214
36    -4.56   -0.81  1.0000
  10 -16  -4      539      210
380     5.17   -2.67  1.0000
   8 -16  -4     3495      308
2036  -12.02   -1.95  1.0000
   6 -16  -4     1451      326
2526     7.07   11.51  1.0000
   4 -16  -4      230      197
80     2.60    7.09  1.0000
   2 -16  -4      490      244
706   -5.76    7.32  1.0000
   0 -16  -4     2189      347
3030     6.94    7.14  1.0000
   4  16   4      227      222
600  -10.38   -1.59  1.0000
  -6 -16  -4     1347      183
1862     4.29    3.69  1.0000
   6  16   4      823      287
1235     4.29   -3.69  1.0000
  -8 -16  -4      488      146
458  -11.59   -1.99  1.0000
   8  16   4      570      247
561  -11.59    1.99  1.0000
 -10 -16  -4      549      147
336     1.88    6.11  1.0000
  10  16   4      453      237
264     1.88   -6.11  1.0000
 -12 -16  -4      920      166
529     8.37    0.68  1.0000
  12  16   4      886      281
503     8.37   -0.68  1.0000
 -14 -16  -4      953      190
995    -4.67   -0.03  1.0000
  14  16   4     1212      326
```

1264	-4.67	0.03	1.0000	
-16	-16	-4	83	208
145	-4.29	2.41	1.0000	
16	16	4	122	279
201	-4.29	-2.41	1.0000	
-18	-16	-4	122	212
234	4.70	1.59	1.0000	
18	16	4	19	321
3	4.70	-1.59	1.0000	
-20	-16	-4	11	246
18	-4.99	4.12	1.0000	
20	16	4	56	338
-154	-4.99	-4.12	1.0000	
19	-17	-4	91	276
120	0.31	1.48	1.0000	
19	-17	-4	3	279
-86	0.31	1.48	1.0000	
17	-17	-4	589	272
345	-3.25	3.71	1.0000	
15	-17	-4	106	233
134	3.15	-1.66	1.0000	
13	-17	-4	24	227
36	0.44	1.00	1.0000	
11	-17	-4	334	229
330	0.25	0.34	1.0000	
9	-17	-4	241	223
352	0.64	2.88	1.0000	
7	-17	-4	237	239
601	3.13	-8.48	1.0000	
5	-17	-4	481	226
413	-3.48	-0.56	1.0000	
3	-17	-4	890	276
1136	4.51	-12.59	1.0000	
1	-17	-4	1597	357
2437	-2.88	-8.08	1.0000	
-1	-17	-4	488	252
188	5.39	6.01	1.0000	
5	17	4	64	236
319	0.84	-8.54	1.0000	
-7	-17	-4	11	140
-100	2.68	1.12	1.0000	
7	17	4	11	250
-291	2.68	-1.12	1.0000	
-9	-17	-4	456	157

```
556      5.30    -6.13  1.0000
   9   17    4       343       248
416      5.30     6.13  1.0000
 -11  -17   -4       278       175
762     -8.46     6.21  1.0000
  11   17    4        38       225
262     -8.46    -6.21  1.0000
 -13  -17   -4       534       180
457     -2.67    -2.42  1.0000
  13   17    4       883       301
789     -2.67     2.42  1.0000
 -15  -17   -4       700       208
692      2.97     1.86  1.0000
  15   17    4       519       320
509      2.97    -1.86  1.0000
 -17  -17   -4       202       215
73     -3.30    -3.63  1.0000
  17   17    4        32       271
-55     -3.30     3.63  1.0000
 -19  -17   -4         8       238
-10      1.83     1.52  1.0000
  19   17    4       342       329
417      1.83    -1.52  1.0000
  18  -18   -4         6       298
65     -3.71    -3.49  1.0000
  16  -18   -4        82       254
175      4.68    -1.60  1.0000
  14  -18   -4       259       255
364     -5.50    -4.13  1.0000
  12  -18   -4       143       236
235     -2.95     1.29  1.0000
  10  -18   -4       426       254
602     -3.64     2.53  1.0000
   8  -18   -4       359       232
359      0.02    -0.08  1.0000
   6  -18   -4       801       263
813     -3.52     4.37  1.0000
   4  -18   -4       384       235
234      3.01     8.88  1.0000
   2  -18   -4        12       238
-34      3.01     0.55  1.0000
   0  -18   -4       595       272
525     -3.98     7.62  1.0000
  -2  -18   -4      1027       318
```

1122	0.91	-1.20	1.0000	
4	18	4	285	229
334	2.63	1.06	1.0000	
-6	-18	-4	311	157
152	-7.04	-1.64	1.0000	
6	18	4	70	244
-45	-7.04	1.64	1.0000	
-8	-18	-4	404	163
540	3.52	-1.05	1.0000	
8	18	4	223	266
324	3.52	1.05	1.0000	
-10	-18	-4	21	151
-56	-2.93	2.65	1.0000	
10	18	4	860	268
625	-2.93	-2.65	1.0000	
-12	-18	-4	235	179
145	1.55	-4.65	1.0000	
12	18	4	330	263
228	1.55	4.65	1.0000	
14	18	4	243	297
339	1.87	-2.21	1.0000	
16	18	4	7	320
-6	2.20	-0.46	1.0000	
-18	-18	-4	53	221
166	-3.60	3.47	1.0000	
18	18	4	403	372
663	-3.60	-3.47	1.0000	
17	-19	-4	82	282
117	1.85	-1.47	1.0000	
15	-19	-4	2	250
90	-0.79	7.61	1.0000	
13	-19	-4	25	234
-274	2.35	-3.66	1.0000	
11	-19	-4	90	230
37	3.66	1.88	1.0000	
9	-19	-4	964	256
886	-1.15	-1.72	1.0000	
7	-19	-4	11	215
-225	0.18	-2.92	1.0000	
5	-19	-4	332	256
477	-0.67	-3.93	1.0000	
3	-19	-4	265	249
143	-2.18	-3.68	1.0000	
1	-19	-4	1985	381

2132	2.13	-1.07	1.0000	
-1	-19	-4	149	267
492	-3.62	-7.88	1.0000	
-3	-19	-4	969	298
411	4.20	8.43	1.0000	
5	19	4	917	250
707	-3.57	-0.14	1.0000	
-7	-19	-4	846	185
1039	1.60	2.57	1.0000	
7	19	4	844	305
1037	1.60	-2.57	1.0000	
9	19	4	114	259
-207	-3.42	-8.60	1.0000	
11	19	4	283	265
231	2.90	8.20	1.0000	
13	19	4	464	281
524	-0.59	-7.13	1.0000	
15	19	4	299	332
519	-3.21	3.71	1.0000	
17	19	4	714	353
793	0.24	1.45	1.0000	
16	-20	-4	19	269
10	-5.10	-4.05	1.0000	
14	-20	-4	46	270
46	0.63	0.75	1.0000	
12	-20	-4	164	262
358	-3.61	-4.07	1.0000	
10	-20	-4	149	258
439	-7.58	-1.04	1.0000	
8	-20	-4	160	263
588	4.64	8.91	1.0000	
6	-20	-4	494	276
647	-2.84	0.11	1.0000	
4	-20	-4	202	265
236	-1.52	-0.09	1.0000	
2	-20	-4	1812	396
2619	4.25	6.15	1.0000	
0	-20	-4	482	271
317	0.82	-3.74	1.0000	
-2	-20	-4	55	267
37	0.65	0.94	1.0000	
-6	-20	-4	130	174
107	2.80	-3.93	1.0000	
6	20	4	31	238

-258	2.80	3.93	1.0000	
8	20	4	8	268
-18	-0.53	2.36	1.0000	
10	20	4	74	281
-222	5.52	5.02	1.0000	
12	20	4	287	294
577	-5.26	-3.45	1.0000	
14	20	4	233	293
180	2.48	-0.10	1.0000	
16	20	4	322	321
260	0.85	-2.31	1.0000	
15	-21	-4	6	288
37	0.43	-5.92	1.0000	
13	-21	-4	44	277
226	4.12	6.08	1.0000	
11	-21	-4	1117	301
1134	2.94	1.27	1.0000	
9	-21	-4	282	252
262	-0.01	-3.36	1.0000	
7	-21	-4	14	234
-171	-2.66	1.81	1.0000	
5	-21	-4	26	240
-46	0.74	-4.40	1.0000	
3	-21	-4	270	262
210	-1.44	-1.40	1.0000	
1	-21	-4	427	282
482	-4.22	-1.25	1.0000	
-1	-21	-4	25	267
6	3.77	0.61	1.0000	
-3	-21	-4	22	321
108	-7.48	2.56	1.0000	
7	21	4	189	256
234	-4.40	-5.70	1.0000	
9	21	4	3	286
-6	-0.98	-1.18	1.0000	
11	21	4	8	319
23	-0.17	-6.30	1.0000	
13	21	4	304	314
596	1.51	6.03	1.0000	
15	21	4	321	321
412	-1.32	1.92	1.0000	
12	-22	-4	35	282
-152	-5.09	-0.66	1.0000	
10	-22	-4	0	278

11	2.57	2.02	1.0000	
8	-22	-4	97	246
-275	-7.29	-4.48	1.0000	
6	-22	-4	178	271
265	2.62	5.09	1.0000	
4	-22	-4	124	259
125	2.97	1.82	1.0000	
2	-22	-4	20	268
-60	-3.63	-1.31	1.0000	
0	-22	-4	28	267
-29	4.54	0.72	1.0000	
-2	-22	-4	31	286
-77	2.54	-4.12	1.0000	
-4	-22	-4	264	352
266	0.16	-2.64	1.0000	
8	22	4	81	278
202	3.78	2.54	1.0000	
10	22	4	14	297
-89	-2.88	1.61	1.0000	
12	22	4	92	334
234	5.09	0.71	1.0000	
11	-23	-4	226	292
261	0.41	-0.90	1.0000	
9	-23	-4	363	300
630	2.87	4.97	1.0000	
5	-23	-4	93	274
102	-4.56	1.31	1.0000	
3	-23	-4	85	283
129	-1.31	-1.40	1.0000	
1	-23	-4	75	296
242	3.03	-5.16	1.0000	
-1	-23	-4	151	314
463	-6.92	4.06	1.0000	
-3	-23	-4	26	300
39	5.34	-0.87	1.0000	
-5	-23	-4	355	370
688	-5.08	5.03	1.0000	
9	23	4	500	321
418	1.65	0.13	1.0000	
11	23	4	1	315
-159	-0.75	-0.16	1.0000	
8	-24	-4	76	307
73	-0.81	1.19	1.0000	
6	-24	-4	234	298

336	-0.20	-2.64	1.0000	
4	-24	-4	3	294
-246	0.51	1.32	1.0000	
2	-24	-4	171	316
232	2.83	1.28	1.0000	
0	-24	-4	0	311
-235	-0.41	-0.22	1.0000	
-2	-24	-4	175	329
173	0.26	-1.23	1.0000	
-4	-24	-4	102	351
159	2.19	-2.44	1.0000	
-6	-24	-4	4	377
15	2.33	-2.77	1.0000	
5	-25	-4	25	302
27	-0.82	-0.61	1.0000	
3	-25	-4	192	342
132	-1.29	-2.07	1.0000	
1	-25	-4	16	316
-376	-3.15	1.38	1.0000	
-1	-25	-4	83	346
200	3.67	-3.52	1.0000	
-3	-25	-4	63	363
68	-1.99	0.72	1.0000	
-5	-25	-4	172	385
174	0.71	1.09	1.0000	
4	25	-5	50	326
48	-1.72	-0.05	1.0000	
-4	-25	5	863	397
866	-1.72	0.05	1.0000	
2	25	-5	1	287
-47	0.87	-0.55	1.0000	
-2	-25	5	197	351
223	0.87	0.55	1.0000	
0	25	-5	5	288
-31	-0.82	1.74	1.0000	
0	-25	5	126	321
169	-0.82	-1.74	1.0000	
-2	25	-5	496	310
452	0.52	1.04	1.0000	
2	-25	5	189	320
162	0.52	-1.04	1.0000	
4	-25	5	49	316
49	-1.37	1.17	1.0000	
7	24	-5	460	346

446 -0.10 -0.38 1.0000
-7 -24 5 632 388
616 -0.10 0.38 1.0000
5 24 -5 286 312
451 -1.21 -3.56 1.0000
-5 -24 5 7 370
49 -1.21 3.56 1.0000
3 24 -5 24 295
-238 3.57 2.29 1.0000
-3 -24 5 331 343
248 3.57 -2.29 1.0000
1 24 -5 76 270
198 -2.52 -4.01 1.0000
-1 -24 5 72 322
192 -2.52 4.01 1.0000
-1 24 -5 187 280
223 2.25 -1.91 1.0000
1 -24 5 340 324
390 2.25 1.91 1.0000
-3 24 -5 281 294
310 0.43 1.10 1.0000
3 -24 5 219 311
245 0.43 -1.10 1.0000
5 -24 5 77 300
178 -3.10 2.53 1.0000
7 -24 5 141 310
262 2.61 -3.66 1.0000
10 23 -5 1043 391
780 -2.92 2.43 1.0000
8 23 -5 50 309
69 3.86 0.88 1.0000
-8 -23 5 63 388
85 3.86 -0.88 1.0000
6 23 -5 14 287
34 -0.82 2.88 1.0000
-6 -23 5 436 348
553 -0.82 -2.88 1.0000
4 23 -5 369 277
524 2.11 2.55 1.0000
-4 -23 5 21 328
67 2.11 -2.55 1.0000
2 23 -5 274 260
351 0.75 -1.87 1.0000
-2 -23 5 18 318

41 0.75 1.87 1.0000
0 23 -5 28 231
-88 -0.73 4.50 1.0000
0 -23 5 113 291
131 -0.73 -4.50 1.0000
-2 23 -5 34 228
63 -1.34 -2.81 1.0000
2 -23 5 135 289
196 -1.34 2.81 1.0000
-4 23 -5 217 259
77 2.04 4.88 1.0000
4 -23 5 661 297
393 2.04 -4.88 1.0000
6 -23 5 347 283
305 -0.76 -0.74 1.0000
8 -23 5 431 318
645 -0.92 5.22 1.0000
10 -23 5 322 304
280 1.47 -1.15 1.0000
11 22 -5 576 394
593 -1.25 1.57 1.0000
11 22 -5 171 310
178 -1.25 1.57 1.0000
9 22 -5 228 314
336 0.56 -3.04 1.0000
7 22 -5 164 274
262 1.42 -2.65 1.0000
-7 -22 5 267 360
390 1.42 2.65 1.0000
5 22 -5 105 223
122 -0.40 0.85 1.0000
-5 -22 5 290 324
318 -0.40 -0.85 1.0000
3 22 -5 155 228
82 -1.83 -6.55 1.0000
-3 -22 5 200 299
136 -1.83 6.55 1.0000
1 22 -5 201 228
275 2.46 1.33 1.0000
-1 -22 5 387 303
490 2.46 -1.33 1.0000
-1 22 -5 172 227
142 -0.19 -1.05 1.0000
1 -22 5 273 287

234	-0.19	1.05	1.0000	
-3	22	-5	53	226
-10	-1.74	-6.10	1.0000	
3	-22	5	53	263
-16	-1.74	6.10	1.0000	
-5	22	-5	286	249
230	2.84	2.65	1.0000	
5	-22	5	20	244
-117	2.84	-2.65	1.0000	
7	-22	5	127	275
332	-6.01	-0.45	1.0000	
9	-22	5	163	270
169	2.43	-1.48	1.0000	
11	-22	5	491	281
175	2.05	-7.63	1.0000	
14	21	-5	295	450
537	-4.14	-3.98	1.0000	
14	21	-5	120	339
284	-4.14	-3.98	1.0000	
12	21	-5	613	375
822	1.01	4.15	1.0000	
12	21	-5	702	350
926	1.01	4.15	1.0000	
10	21	-5	69	278
194	5.34	1.27	1.0000	
10	21	-5	42	303
144	5.34	1.27	1.0000	
8	21	-5	559	275
544	-3.98	3.06	1.0000	
6	21	-5	69	228
17	4.56	3.53	1.0000	
-6	-21	5	232	335
159	4.56	-3.53	1.0000	
4	21	-5	5	204
-73	1.28	-1.47	1.0000	
-4	-21	5	316	308
244	1.28	1.47	1.0000	
2	21	-5	87	207
-36	-1.72	7.92	1.0000	
-2	-21	5	68	291
105	-1.72	-7.92	1.0000	
0	21	-5	82	207
151	2.20	-6.61	1.0000	
0	-21	5	198	268

338	2.20	6.61	1.0000	
-2	21	-5	61	198
-81	-0.81	6.72	1.0000	
2	-21	5	337	278
482	-0.81	-6.72	1.0000	
-4	21	-5	268	217
162	-3.94	-0.92	1.0000	
4	-21	5	171	258
88	-3.94	0.92	1.0000	
-6	21	-5	194	241
331	3.81	-0.18	1.0000	
6	-21	5	52	253
130	3.81	0.18	1.0000	
8	-21	5	235	272
357	-1.67	-3.37	1.0000	
10	-21	5	49	258
232	-4.15	7.03	1.0000	
12	-21	5	251	281
392	2.42	3.18	1.0000	
14	-21	5	278	300
333	0.37	2.23	1.0000	
15	20	-5	307	327
256	0.10	1.44	1.0000	
15	20	-5	1284	451
1177	0.10	1.44	1.0000	
13	20	-5	619	332
515	2.09	1.62	1.0000	
13	20	-5	1395	397
1239	2.09	1.62	1.0000	
11	20	-5	452	315
738	-0.97	-5.43	1.0000	
11	20	-5	381	320
645	-0.97	-5.43	1.0000	
9	20	-5	217	265
193	-0.08	0.73	1.0000	
9	20	-5	578	280
539	-0.08	0.73	1.0000	
7	20	-5	385	250
559	-2.67	-2.65	1.0000	
5	20	-5	10	211
-19	1.25	-2.41	1.0000	
-5	-20	5	153	288
221	1.25	2.41	1.0000	
3	20	-5	63	181

18 -1.93 2.59 1.0000
-3 -20 5 270 276
163 -1.93 -2.59 1.0000
1 20 -5 424 195
209 1.05 -5.53 1.0000
-1 -20 5 582 290
325 1.05 5.53 1.0000
-1 20 -5 365 204
278 1.74 -1.59 1.0000
1 -20 5 300 258
221 1.74 1.59 1.0000
-3 20 -5 590 215
709 1.34 1.72 1.0000
3 -20 5 483 277
591 1.34 -1.72 1.0000
-5 20 -5 559 223
533 -1.52 -3.21 1.0000
5 -20 5 529 254
505 -1.52 3.21 1.0000
-7 20 -5 66 206
58 1.21 1.17 1.0000
7 -20 5 67 241
59 1.21 -1.17 1.0000
9 -20 5 149 243
391 0.70 -6.56 1.0000
11 -20 5 28 232
14 -3.87 1.26 1.0000
13 -20 5 40 256
204 4.11 -6.20 1.0000
15 -20 5 24 272
110 -4.49 -1.78 1.0000
16 19 -5 698 432
826 -0.85 -2.58 1.0000
16 19 -5 10 300
-198 -0.85 -2.58 1.0000
14 19 -5 540 306
431 3.12 1.71 1.0000
14 19 -5 714 381
590 3.12 1.71 1.0000
12 19 -5 513 306
740 -2.66 2.98 1.0000
12 19 -5 478 327
698 -2.66 2.98 1.0000
10 19 -5 206 267

210	-1.72	0.14	1.0000	
10	19	-5	4	268
-78	-1.72	0.14	1.0000	
8	19	-5	267	261
583	4.91	4.76	1.0000	
8	19	-5	22	246
153	4.91	4.76	1.0000	
6	19	-5	737	243
664	-0.72	-2.02	1.0000	
4	19	-5	132	197
257	2.29	4.06	1.0000	
-4	-19	5	121	275
241	2.29	-4.06	1.0000	
2	19	-5	256	191
338	2.13	-4.61	1.0000	
-2	-19	5	565	287
696	2.13	4.61	1.0000	
0	19	-5	2308	329
3005	-3.98	5.21	1.0000	
0	-19	5	2502	399
3227	-3.98	-5.21	1.0000	
-2	19	-5	2156	305
1962	-0.90	-2.48	1.0000	
2	-19	5	1621	319
1453	-0.90	2.48	1.0000	
-4	19	-5	2224	323
2504	0.85	2.37	1.0000	
4	-19	5	1795	366
2047	0.85	-2.37	1.0000	
-6	19	-5	575	209
467	-7.66	-0.14	1.0000	
6	-19	5	388	239
286	-7.66	0.14	1.0000	
-8	19	-5	190	201
110	0.92	-3.57	1.0000	
8	-19	5	18	230
-63	0.92	3.57	1.0000	
10	-19	5	443	239
407	0.61	0.48	1.0000	
12	-19	5	177	244
71	-3.33	2.82	1.0000	
14	-19	5	14	231
17	0.44	4.95	1.0000	
16	-19	5	84	267

```
143      1.68      1.93  1.0000
  17   18   -5       171       308
250      1.15      2.42  1.0000
  17   18   -5       680       445
833      1.15      2.42  1.0000
  15   18   -5       335       301
370      0.81     -0.05  1.0000
  15   18   -5      1127       398
1190      0.81     -0.05  1.0000
  13   18   -5       237       331
459     -5.06     -1.37  1.0000
  13   18   -5        37       260
-26     -5.06     -1.37  1.0000
  11   18   -5       106       269
230      3.03     -3.12  1.0000
  11   18   -5        14       277
74      3.03     -3.12  1.0000
   9   18   -5       635       278
751      2.12     -1.01  1.0000
   9   18   -5       844       265
978      2.12     -1.01  1.0000
   7   18   -5       286       214
441     -2.16      4.24  1.0000
   7   18   -5        99       236
191     -2.16      4.24  1.0000
   5   18   -5       272       197
333     -3.18      0.17  1.0000
  -5  -18    5        14       286
-88     -3.18     -0.17  1.0000
   3   18   -5       813       213
768      2.58      1.34  1.0000
  -3  -18    5       820       287
775      2.58     -1.34  1.0000
   1   18   -5      1553       288
1571      0.01      4.43  1.0000
  -1  -18    5      1365       307
1383      0.01     -4.43  1.0000
  -1   18   -5      1227       275
1414      0.97     -2.67  1.0000
   1  -18    5      1424       304
1625      0.97      2.67  1.0000
  -3   18   -5        10       180
89      7.55     -1.31  1.0000
   3  -18    5        78       230
```

-59 7.55 1.31 1.0000
-5 18 -5 137 184
144 -0.16 -0.19 1.0000
5 -18 5 0 214
-193 -0.16 0.19 1.0000
-7 18 -5 654 210
578 4.99 3.92 1.0000
7 -18 5 617 238
540 4.99 -3.92 1.0000
9 -18 5 54 223
136 -4.15 -0.76 1.0000
11 -18 5 162 235
134 0.56 -6.18 1.0000
13 -18 5 346 241
304 -1.22 1.13 1.0000
15 -18 5 17 235
23 -0.03 -2.18 1.0000
17 -18 5 153 274
129 -1.19 -0.90 1.0000
18 17 -5 320 337
286 -1.16 -0.90 1.0000
18 17 -5 1431 459
1360 -1.16 -0.90 1.0000
16 17 -5 597 381
480 -2.93 -1.17 1.0000
16 17 -5 600 319
483 -2.93 -1.17 1.0000
14 17 -5 4 266
18 1.64 4.24 1.0000
14 17 -5 277 325
462 1.64 4.24 1.0000
12 17 -5 572 280
657 2.01 0.37 1.0000
12 17 -5 693 278
787 2.01 0.37 1.0000
10 17 -5 374 237
165 -6.24 6.83 1.0000
10 17 -5 114 232
-451 -6.24 6.83 1.0000
8 17 -5 46 199
-16 -0.09 -5.88 1.0000
8 17 -5 283 220
135 -0.09 -5.88 1.0000
6 17 -5 443 203

346 2.65 0.93 1.0000
4 17 -5 862 223
1190 -4.38 -4.32 1.0000
-4 -17 5 970 319
1318 -4.38 4.32 1.0000
2 17 -5 1136 271
1575 4.58 -3.79 1.0000
-2 -17 5 838 304
1220 4.58 3.79 1.0000
0 17 -5 312 187
436 -2.70 -0.93 1.0000
0 -17 5 244 241
355 -2.70 0.93 1.0000
-2 17 -5 1940 280
1917 -3.76 -1.11 1.0000
2 -17 5 2033 334
2008 -3.76 1.11 1.0000
-4 17 -5 1644 282
1779 -4.32 -0.02 1.0000
4 -17 5 1465 295
1593 -4.32 0.02 1.0000
-6 17 -5 894 215
803 -2.31 2.14 1.0000
6 -17 5 743 242
660 -2.31 -2.14 1.0000
-8 17 -5 1902 313
2155 -4.05 -4.76 1.0000
8 -17 5 1949 332
2204 -4.05 4.76 1.0000
10 -17 5 426 221
480 1.40 1.13 1.0000
12 -17 5 252 224
131 -0.12 3.83 1.0000
14 -17 5 188 233
135 -2.98 0.54 1.0000
16 -17 5 114 256
181 1.05 2.27 1.0000
18 -17 5 30 276
67 1.29 2.34 1.0000
19 16 -5 1276 470
1479 1.61 1.91 1.0000
19 16 -5 8 335
-156 1.61 1.91 1.0000
17 16 -5 515 394

```
651      1.10      2.28  1.0000
  17   16  -5         9       310
-164      1.10      2.28  1.0000
  15   16  -5        47       289
120    -3.10      2.85  1.0000
  15   16  -5       101       324
203    -3.10      2.85  1.0000
  13   16  -5       681       263
512      1.67    -7.14  1.0000
  13   16  -5      1143       299
939      1.67    -7.14  1.0000
  11   16  -5        37       216
-440      2.42      4.69  1.0000
  11   16  -5        20       223
18     2.42     4.69  1.0000
   9   16  -5       341       244
306    -3.05    -0.79  1.0000
   9   16  -5       403       219
366    -3.05    -0.79  1.0000
   7   16  -5      2111       322
1894      5.32    -0.45  1.0000
   7   16  -5      2061       298
1846      5.32    -0.45  1.0000
   5   16  -5       443       233
1261     -0.05    15.44  1.0000
   3   16  -5       837       193
625    -2.04      2.87  1.0000
  -3  -16   5       901       258
681    -2.04    -2.87  1.0000
   1   16  -5      1210       266
1581      4.97      2.21  1.0000
  -1  -16   5      1315       289
1702      4.97     -2.21  1.0000
  -1   16  -5      2066       267
1748      7.50      7.96  1.0000
   1  -16   5      2071       316
1757      7.50     -7.96  1.0000
  -3   16  -5       822       253
1232     -1.30     -7.35  1.0000
   3  -16   5       967       270
1404     -1.30      7.35  1.0000
  -5   16  -5       754       170
178    13.67      3.61  1.0000
   5  -16   5       579       206
```

89 13.67 -3.61 1.0000
-7 16 -5 213 178
209 -1.22 -0.24 1.0000
7 -16 5 266 219
262 -1.22 0.24 1.0000
-9 16 -5 553 192
508 -0.95 0.76 1.0000
9 -16 5 401 216
363 -0.95 -0.76 1.0000
11 -16 5 2273 317
1824 0.66 -4.27 1.0000
13 -16 5 396 242
543 -5.84 -1.38 1.0000
15 -16 5 23 223
-231 3.30 2.60 1.0000
17 -16 5 188 249
123 -2.96 -1.13 1.0000
19 -16 5 251 297
324 -0.85 -2.49 1.0000
20 15 -5 1514 497
1973 -4.46 -1.72 1.0000
20 15 -5 2 341
46 -4.46 -1.72 1.0000
18 15 -5 7 300
0 -0.07 -2.24 1.0000
18 15 -5 1194 431
1282 -0.07 -2.24 1.0000
16 15 -5 24 289
-62 3.33 2.65 1.0000
16 15 -5 386 358
525 3.33 2.65 1.0000
14 15 -5 453 288
736 -7.00 -1.21 1.0000
14 15 -5 432 303
709 -7.00 -1.21 1.0000
12 15 -5 49 233
-145 -5.33 2.89 1.0000
12 15 -5 49 226
-43 -5.33 2.89 1.0000
10 15 -5 10 220
18 1.52 -4.52 1.0000
10 15 -5 18 198
54 1.52 -4.52 1.0000
8 15 -5 59 221

221 -5.40 3.30 1.0000
8 15 -5 119 192
329 -5.40 3.30 1.0000
6 15 -5 173 184
-40 -7.83 -8.27 1.0000
6 15 -5 173 172
-77 -7.83 -8.27 1.0000
4 15 -5 911 271
1797 12.06 -10.24 1.0000
2 15 -5 2510 203
1137 -14.14 -1.29 1.0000
-2 -15 5 2645 270
1229 -14.14 1.29 1.0000
0 15 -5 1288 183
817 4.87 -7.00 1.0000
0 -15 5 1545 256
1034 4.87 7.00 1.0000
-2 15 -5 63 158
135 -1.02 3.17 1.0000
2 -15 5 92 207
177 -1.02 -3.17 1.0000
-4 15 -5 587 194
876 -10.63 4.54 1.0000
4 -15 5 382 220
592 -10.63 -4.54 1.0000
-6 15 -5 248 169
321 1.50 -7.41 1.0000
6 -15 5 181 201
226 1.50 7.41 1.0000
-8 15 -5 195 188
594 -1.54 9.15 1.0000
8 -15 5 169 214
549 -1.54 -9.15 1.0000
10 -15 5 612 242
1060 -1.87 8.15 1.0000
12 -15 5 327 213
232 2.42 0.57 1.0000
14 -15 5 529 228
491 1.03 -1.80 1.0000
16 -15 5 75 239
166 -3.09 2.90 1.0000
18 -15 5 427 257
466 0.80 -0.12 1.0000
20 -15 5 3 278

-358 0.10 1.43 1.0000

21 14 -5 1919 505

2072 0.30 2.25 1.0000

21 14 -5 169 372

214 0.30 2.25 1.0000

19 14 -5 990 431

1271 0.41 4.94 1.0000

19 14 -5 33 327

-32 0.41 4.94 1.0000

17 14 -5 1026 359

914 -3.04 0.44 1.0000

17 14 -5 13 281

-80 -3.04 0.44 1.0000

15 14 -5 521 288

489 0.93 -1.86 1.0000

15 14 -5 1112 316

1068 0.93 -1.86 1.0000

13 14 -5 694 270

768 4.38 6.06 1.0000

13 14 -5 451 269

496 4.38 6.06 1.0000

11 14 -5 375 229

539 0.69 -3.27 1.0000

11 14 -5 463 223

644 0.69 -3.27 1.0000

9 14 -5 502 196

209 6.82 4.19 1.0000

9 14 -5 520 206

221 6.82 4.19 1.0000

7 14 -5 1010 277

1865 -1.07 13.63 1.0000

7 14 -5 1127 291

2030 -1.07 13.63 1.0000

5 14 -5 67 160

171 4.29 -0.17 1.0000

5 14 -5 221 185

391 4.29 -0.17 1.0000

3 14 -5 1283 176

769 1.59 20.05 1.0000

-3 -14 5 1393 233

865 1.59 -20.05 1.0000

1 14 -5 823 187

1042 1.73 6.89 1.0000

-1 -14 5 722 216

916 1.73 -6.89 1.0000
-1 14 -5 1011 157
418 2.33 -9.63 1.0000
1 -14 5 776 170
272 2.33 9.63 1.0000
-3 14 -5 68 92
188 15.43 6.07 1.0000
-3 14 -5 105 143
109 15.43 6.07 1.0000
3 -14 5 116 169
128 15.43 -6.07 1.0000
-5 14 -5 203 126
705 -1.47 -10.76 1.0000
-5 14 -5 42 148
347 -1.47 -10.76 1.0000
5 -14 5 48 188
365 -1.47 10.76 1.0000
-7 14 -5 1147 149
1014 1.73 -4.35 1.0000
-7 14 -5 1564 202
1412 1.73 -4.35 1.0000
7 -14 5 1356 230
1215 1.73 4.35 1.0000
-9 14 -5 1578 204
2329 5.77 5.58 1.0000
-9 14 -5 1556 267
2302 5.77 5.58 1.0000
9 -14 5 1509 295
2246 5.77 -5.58 1.0000
11 -14 5 2203 351
3511 -10.56 3.92 1.0000
13 -14 5 379 213
356 2.24 -1.47 1.0000
15 -14 5 262 244
476 -6.96 -1.25 1.0000
17 -14 5 29 226
-241 1.64 4.38 1.0000
19 -14 5 183 261
118 3.12 1.70 1.0000
21 -14 5 143 308
325 -4.28 -4.05 1.0000
20 13 -5 35 307
8 4.11 -6.20 1.0000
20 13 -5 1012 449

1606	4.11	-6.20	1.0000		
18	13	-5	4	292	
-85	-1.19	1.12	1.0000		
18	13	-5	1304	378	
1224	-1.19	1.12	1.0000		
16	13	-5	292	295	
415	-5.91	-1.37	1.0000		
16	13	-5	691	320	
894	-5.91	-1.37	1.0000		
14	13	-5	735	280	
710	2.29	-1.58	1.0000		
14	13	-5	500	256	
478	2.29	-1.58	1.0000		
12	13	-5	63	229	
14	-9.02	1.31	1.0000		
12	13	-5	142	225	
332	-9.02	1.31	1.0000		
10	13	-5	184	200	
144	-5.70	-1.38	1.0000		
10	13	-5	46	185	
-22	-5.70	-1.38	1.0000		
8	13	-5	172	209	
513	-7.30	-6.49	1.0000		
8	13	-5	156	181	
482	-7.30	-6.49	1.0000		
6	13	-5	867	204	
1035	3.68	-5.31	1.0000		
6	13	-5	490	180	
606	3.68	-5.31	1.0000		
4	13	-5	318	184	
968	-14.50	-8.93	1.0000		
2	13	-5	383	215	
1031	17.66	-9.62	1.0000		
-2	-13	5	737	259	
1736	17.66	9.62	1.0000		
0	13	-5	1081	221	
1567	-5.90	0.44	1.0000		
0	-13	5	1214	257	
1728	-5.90	-0.44	1.0000		
-2	13	-5	363	114	
892	-9.00	5.47	1.0000		
-2	13	-5	221	158	
652	-9.00	5.47	1.0000		
2	-13	5	179	181	

567 -9.00 -5.47 1.0000
-4 13 -5 747 217
1268 8.57 3.34 1.0000
-4 13 -5 1112 141
1743 8.57 3.34 1.0000
4 -13 5 873 222
1440 8.57 -3.34 1.0000
-6 13 -5 1597 301
4160 -8.90 19.47 1.0000
-6 13 -5 1829 238
4530 -8.90 19.47 1.0000
6 -13 5 1386 327
3811 -8.90 -19.47 1.0000
-8 13 -5 808 148
1170 4.02 -9.09 1.0000
-8 13 -5 822 190
1188 4.02 -9.09 1.0000
8 -13 5 471 201
736 4.02 9.09 1.0000
-10 13 -5 300 135
303 1.35 7.31 1.0000
-10 13 -5 224 153
215 1.35 7.31 1.0000
10 -13 5 270 186
268 1.35 -7.31 1.0000
-12 13 -5 953 166
812 0.45 -2.16 1.0000
12 -13 5 819 217
688 0.45 2.16 1.0000
14 -13 5 802 247
886 4.33 6.01 1.0000
16 -13 5 644 249
483 1.78 -7.11 1.0000
18 -13 5 39 231
-67 -5.20 -1.39 1.0000
20 -13 5 511 292
411 2.23 1.60 1.0000
19 12 -5 239 275
112 -3.42 2.77 1.0000
19 12 -5 2064 406
1651 -3.42 2.77 1.0000
17 12 -5 681 308
467 -0.12 3.88 1.0000
17 12 -5 20 246

-216 -0.12 3.88 1.0000
15 12 -5 525 278
405 2.36 0.52 1.0000
15 12 -5 216 273
142 2.36 0.52 1.0000
13 12 -5 526 235
424 0.23 2.09 1.0000
13 12 -5 709 243
590 0.23 2.09 1.0000
11 12 -5 2297 332
2661 3.92 0.00 1.0000
11 12 -5 2019 334
2360 3.92 0.00 1.0000
9 12 -5 941 182
592 4.71 4.68 1.0000
9 12 -5 553 190
291 4.71 4.68 1.0000
7 12 -5 544 163
240 7.44 3.44 1.0000
7 12 -5 543 172
239 7.44 3.44 1.0000
5 12 -5 1310 156
294 -3.25 17.99 1.0000
5 12 -5 1511 171
400 -3.25 17.99 1.0000
3 12 -5 1788 228
1791 1.99 -0.54 1.0000
1 12 -5 50 126
18 -5.42 6.55 1.0000
-1 -12 5 160 167
351 -5.42 -6.55 1.0000
-1 12 -5 879 196
1033 5.59 5.42 1.0000
-1 12 -5 907 114
1064 5.59 5.42 1.0000
1 -12 5 1159 205
1346 5.59 -5.42 1.0000
-3 12 -5 1423 227
2286 2.19 -21.80 1.0000
-3 12 -5 1709 164
2703 2.19 -21.80 1.0000
3 -12 5 1674 251
2663 2.19 21.80 1.0000
-5 12 -5 459 126

203 3.47 -9.26 1.0000
-5 12 -5 506 94
239 3.47 -9.26 1.0000
5 -12 5 433 148
192 3.47 9.26 1.0000
-7 12 -5 2790 152
1640 5.23 -9.49 1.0000
-7 12 -5 2536 202
1447 5.23 -9.49 1.0000
7 -12 5 2360 210
1316 5.23 9.49 1.0000
-9 12 -5 1058 121
333 -11.15 -11.46 1.0000
-9 12 -5 1049 137
327 -11.15 -11.46 1.0000
9 -12 5 841 174
208 -11.15 11.46 1.0000
-11 12 -5 671 157
834 4.99 7.01 1.0000
11 -12 5 528 206
656 4.99 -7.01 1.0000
-13 12 -5 305 155
614 -9.10 -1.22 1.0000
13 -12 5 147 216
347 -9.10 1.22 1.0000
15 -12 5 50 200
-24 -5.32 3.09 1.0000
17 -12 5 523 254
608 2.01 0.46 1.0000
19 -12 5 517 278
743 -2.69 2.92 1.0000
21 -12 5 303 300
454 0.92 4.17 1.0000
20 11 -5 288 350
302 -3.82 1.26 1.0000
20 11 -5 1275 411
1329 -3.82 1.26 1.0000
18 11 -5 881 352
887 0.50 -6.11 1.0000
18 11 -5 91 279
55 0.50 -6.11 1.0000
16 11 -5 2832 399
2311 0.63 -4.44 1.0000
16 11 -5 1723 287

1322	0.63	-4.44	1.0000	
14	11	-5	2207	400
3522	-10.57	3.98	1.0000	
14	11	-5	1982	376
3235	-10.57	3.98	1.0000	
12	11	-5	784	254
971	5.15	-6.96	1.0000	
12	11	-5	197	224
240	5.15	-6.96	1.0000	
10	11	-5	248	187
-27	-11.37	7.57	1.0000	
10	11	-5	191	189
447	-11.37	7.57	1.0000	
8	11	-5	3728	316
3802	-2.32	-0.77	1.0000	
8	11	-5	3176	289
3243	-2.32	-0.77	1.0000	
6	11	-5	755	240
1807	-4.31	-12.42	1.0000	
6	11	-5	620	236
1593	-4.31	-12.42	1.0000	
4	11	-5	1314	169
953	8.14	4.85	1.0000	
4	11	-5	1484	178
1104	8.14	4.85	1.0000	
2	11	-5	384	138
435	-1.70	-8.65	1.0000	
-2	-11	5	746	182
847	-1.70	8.65	1.0000	
0	11	-5	969	206
1859	10.08	3.27	1.0000	
0	-11	5	1234	237
2218	10.08	-3.27	1.0000	
-2	11	-5	1019	113
1033	5.44	10.56	1.0000	
-2	11	-5	1016	161
1030	5.44	10.56	1.0000	
2	-11	5	1152	192
1193	5.44	-10.56	1.0000	
-4	11	-5	1432	218
2519	-11.39	19.00	1.0000	
-4	11	-5	1402	167
2473	-11.39	19.00	1.0000	
4	-11	5	1715	236

2942 -11.39 -19.00 1.0000
-6 11 -5 474 153
878 10.48 9.13 1.0000
-6 11 -5 526 125
961 10.48 9.13 1.0000
6 -11 5 379 171
721 10.48 -9.13 1.0000
-8 11 -5 62 147
798 -3.75 19.72 1.0000
-8 11 -5 95 132
939 -3.75 19.72 1.0000
8 -11 5 55 177
763 -3.75 -19.72 1.0000
-10 11 -5 29 128
188 4.87 -6.21 1.0000
-10 11 -5 36 120
206 4.87 -6.21 1.0000
10 -11 5 77 166
302 4.87 6.21 1.0000
-12 11 -5 2794 234
3219 4.13 0.00 1.0000
12 -11 5 2962 326
3397 4.13 0.00 1.0000
-14 11 -5 372 150
543 0.83 3.38 1.0000
14 -11 5 120 200
224 0.83 -3.38 1.0000
16 -11 5 27 211
47 2.30 4.56 1.0000
18 -11 5 27 242
100 3.10 -3.04 1.0000
20 -11 5 31 263
120 -0.96 -5.25 1.0000
22 -11 5 5 288
-85 -1.31 1.45 1.0000
21 10 -5 88 341
-89 -4.16 7.01 1.0000
21 10 -5 1040 417
1682 -4.16 7.01 1.0000
19 10 -5 246 319
219 0.59 0.50 1.0000
19 10 -5 1186 348
1125 0.59 0.50 1.0000
17 10 -5 460 280

513 1.24 1.07 1.0000
17 10 -5 433 306
484 1.24 1.07 1.0000
15 10 -5 751 275
1254 -2.02 8.18 1.0000
15 10 -5 566 284
1007 -2.02 8.18 1.0000
13 10 -5 135 204
102 1.13 -7.31 1.0000
13 10 -5 73 206
-203 1.13 -7.31 1.0000
11 10 -5 48 188
238 5.04 6.01 1.0000
11 10 -5 82 188
-16 5.04 6.01 1.0000
9 10 -5 257 175
54 1.97 -7.30 1.0000
9 10 -5 1405 196
830 1.97 -7.30 1.0000
7 10 -5 1964 243
2051 -1.04 0.86 1.0000
7 10 -5 2519 269
2618 -1.04 0.86 1.0000
5 10 -5 153 152
328 -1.00 4.90 1.0000
5 10 -5 110 145
262 -1.00 4.90 1.0000
3 10 -5 749 199
1572 -11.63 2.09 1.0000
1 10 -5 4780 284
4355 -4.83 0.19 1.0000
-1 -10 5 5098 300
4661 -4.83 -0.19 1.0000
-1 10 -5 990 197
1746 -5.74 -15.71 1.0000
-1 10 -5 1091 129
1893 -5.74 -15.71 1.0000
1 -10 5 950 201
1673 -5.74 15.71 1.0000
-3 10 -5 269 112
179 -3.13 -12.10 1.0000
-3 10 -5 375 88
326 -3.13 -12.10 1.0000
3 -10 5 285 115

183	-3.13	12.10	1.0000	
-5	10	-5	694	101
-16	3.69	-22.53	1.0000	
-5	10	-5	507	87
174	3.69	-22.53	1.0000	
5	-10	5	527	122
218	3.69	22.53	1.0000	
-7	10	-5	1631	183
1401	-4.28	-19.22	1.0000	
-7	10	-5	1730	143
1511	-4.28	-19.22	1.0000	
7	-10	5	1548	193
1299	-4.28	19.22	1.0000	
-9	10	-5	86	114
204	4.45	-0.21	1.0000	
-9	10	-5	108	105
238	4.45	-0.21	1.0000	
9	-10	5	41	133
129	4.45	0.21	1.0000	
-11	10	-5	169	122
383	-11.24	-7.34	1.0000	
-11	10	-5	150	121
335	-11.24	-7.34	1.0000	
11	-10	5	246	179
551	-11.24	7.34	1.0000	
-13	10	-5	325	130
278	-5.69	1.19	1.0000	
13	-10	5	221	183
176	-5.69	-1.19	1.0000	
-15	10	-5	195	153
328	1.54	4.43	1.0000	
15	-10	5	215	207
353	1.54	-4.43	1.0000	
-17	10	-5	112	136
-124	-6.21	-6.76	1.0000	
17	-10	5	299	223
109	-6.21	6.76	1.0000	
19	-10	5	5	233
-3	-1.86	0.21	1.0000	
21	-10	5	190	263
382	5.36	1.22	1.0000	
23	-10	5	20	290
-182	-3.00	2.45	1.0000	
20	9	-5	462	343

```
846     0.69    -6.58  1.0000
  20    9   -5      798      295
1285     0.69    -6.58  1.0000
  18    9   -5      254      324
419    -4.18    -0.87  1.0000
  18    9   -5      250      243
413    -4.18    -0.87  1.0000
  16    9   -5      529      283
487    -0.92    -0.76  1.0000
  16    9   -5      694      231
646    -0.92    -0.76  1.0000
  14    9   -5     1586      348
2346     5.80    -5.66  1.0000
  14    9   -5     1078      316
1716     5.80    -5.66  1.0000
  12    9   -5      688      189
117   -11.34    11.60  1.0000
  12    9   -5     1242      181
456   -11.34    11.60  1.0000
  10    9   -5       93      169
218     4.57     0.35  1.0000
  10    9   -5        2      166
38     4.57     0.35  1.0000
   8    9   -5     3519      323
4517    -3.22     7.29  1.0000
   8    9   -5     3904      325
4953    -3.22     7.29  1.0000
   6    9   -5     3874      302
4757    -0.50    15.34  1.0000
   6    9   -5     3308      285
4106    -0.50    15.34  1.0000
   4    9   -5       85      154
787    16.53   -18.22  1.0000
   4    9   -5       86      152
648    16.53   -18.22  1.0000
   2    9   -5     1887      250
3224     8.23    12.15  1.0000
  -2   -9    5     2203      269
3642     8.23   -12.15  1.0000
   0    9   -5     1395      152
1001     4.94     3.58  1.0000
   0    9   -5     1448      104
1046     4.94     3.58  1.0000
   0   -9    5     1510      186
```

1101 4.94 -3.58 1.0000
-2 9 -5 149 92
484 7.98 16.43 1.0000
-2 9 -5 138 123
420 7.98 16.43 1.0000
2 -9 5 125 128
393 7.98 -16.43 1.0000
-4 9 -5 404 138
1843 8.97 24.70 1.0000
-4 9 -5 350 179
1686 8.97 24.70 1.0000
4 -9 5 363 182
1728 8.97 -24.70 1.0000
-6 9 -5 1004 193
2161 -12.83 20.44 1.0000
-6 9 -5 1037 156
2219 -12.83 20.44 1.0000
6 -9 5 990 201
2175 -12.83 -20.44 1.0000
-8 9 -5 303 97
286 4.31 4.77 1.0000
-8 9 -5 174 102
146 4.31 4.77 1.0000
8 -9 5 53 130
5 4.31 -4.77 1.0000
-10 9 -5 1208 127
677 2.28 7.31 1.0000
-10 9 -5 1406 140
827 2.28 7.31 1.0000
10 -9 5 1322 168
763 2.28 -7.31 1.0000
-12 9 -5 829 142
513 4.70 -4.47 1.0000
12 -9 5 647 176
371 4.70 4.47 1.0000
-14 9 -5 517 121
226 6.67 -4.12 1.0000
14 -9 5 608 192
286 6.67 4.12 1.0000
-16 9 -5 273 139
239 -3.02 0.81 1.0000
16 -9 5 445 218
405 -3.02 -0.81 1.0000
-18 9 -5 630 176

744 2.08 0.97 1.0000
18 -9 5 469 249
568 2.08 -0.97 1.0000
20 -9 5 93 239
78 -0.11 0.72 1.0000
22 -9 5 213 299
313 0.47 -2.94 1.0000
23 8 -5 1854 434
2280 -0.80 5.18 1.0000
23 8 -5 175 382
308 -0.80 5.18 1.0000
21 8 -5 201 325
314 -1.72 -3.29 1.0000
21 8 -5 329 271
472 -1.72 -3.29 1.0000
19 8 -5 757 263
606 0.86 3.54 1.0000
19 8 -5 184 308
110 0.86 3.54 1.0000
17 8 -5 1788 353
2027 -4.18 4.77 1.0000
17 8 -5 2155 364
2421 -4.18 4.77 1.0000
15 8 -5 316 269
797 -1.63 -9.14 1.0000
15 8 -5 8 228
158 -1.63 -9.14 1.0000
13 8 -5 693 243
1029 4.01 9.03 1.0000
13 8 -5 420 229
665 4.01 9.03 1.0000
11 8 -5 32 206
592 -4.00 -19.66 1.0000
11 8 -5 29 187
524 -4.00 -19.66 1.0000
9 8 -5 57 158
-102 4.52 -4.77 1.0000
9 8 -5 149 144
133 4.52 -4.77 1.0000
7 8 -5 3279 257
2329 -6.57 -6.15 1.0000
7 8 -5 3391 229
2424 -6.57 -6.15 1.0000
5 8 -5 1633 162

1119	-11.52	-20.41	1.0000	
5	8	-5	1671	198
1161	-11.52	-20.41	1.0000	
3	8	-5	2074	146
771	-15.68	13.52	1.0000	
3	8	-5	2372	97
977	-15.68	13.52	1.0000	
3	8	-5	2347	142
960	-15.68	13.52	1.0000	
-3	-8	5	1984	135
689	-15.68	-13.52	1.0000	
1	8	-5	1437	232
2755	-12.45	-9.76	1.0000	
1	8	-5	1597	161
2981	-12.45	-9.76	1.0000	
-1	-8	5	1412	224
2713	-12.45	9.76	1.0000	
-1	-8	5	1655	227
3056	-12.45	9.76	1.0000	
-1	8	-5	2091	252
3891	-12.92	-13.44	1.0000	
-1	8	-5	2182	194
4019	-12.92	-13.44	1.0000	
1	-8	5	1700	252
3330	-12.92	13.44	1.0000	
1	-8	5	1939	241
3674	-12.92	13.44	1.0000	
-3	8	-5	2026	203
3756	-8.31	-16.00	1.0000	
-3	8	-5	2095	245
3853	-8.31	-16.00	1.0000	
3	-8	5	2156	241
3935	-8.31	16.00	1.0000	
-5	8	-5	2199	311
6338	3.37	-28.33	1.0000	
-5	8	-5	2462	341
6780	3.37	-28.33	1.0000	
5	-8	5	2404	347
6683	3.37	28.33	1.0000	
-7	8	-5	401	126
1118	4.19	-11.08	1.0000	
-7	8	-5	385	157
1091	4.19	-11.08	1.0000	
7	-8	5	459	171

1216	4.19	11.08	1.0000	
-9	8	-5	4652	285
5758	-3.05	-7.07	1.0000	
-9	8	-5	4204	293
5257	-3.05	-7.07	1.0000	
9	-8	5	4326	336
5394	-3.05	7.07	1.0000	
-11	8	-5	4116	255
4231	-2.46	0.57	1.0000	
-11	8	-5	4450	262
4570	-2.46	0.57	1.0000	
11	-8	5	4461	316
4581	-2.46	-0.57	1.0000	
-13	8	-5	367	149
824	-7.14	6.54	1.0000	
13	-8	5	314	198
746	-7.14	-6.54	1.0000	
-15	8	-5	148	137
372	-5.33	-3.22	1.0000	
15	-8	5	159	205
388	-5.33	3.22	1.0000	
-17	8	-5	85	134
12	-0.08	5.75	1.0000	
17	-8	5	165	202
56	-0.08	-5.75	1.0000	
-19	8	-5	168	170
425	4.84	-4.66	1.0000	
19	-8	5	11	223
21	4.84	4.66	1.0000	
21	-8	5	75	268
45	-3.99	3.15	1.0000	
23	-8	5	20	287
-361	3.79	0.78	1.0000	
24	7	-5	803	329
1073	2.59	-3.62	1.0000	
24	7	-5	26	396
-39	2.59	-3.62	1.0000	
22	7	-5	261	294
533	-5.98	-0.53	1.0000	
22	7	-5	173	366
403	-5.98	-0.53	1.0000	
20	7	-5	287	276
271	1.14	-1.20	1.0000	
20	7	-5	248	318

233	1.14	-1.20	1.0000	
18	7	-5	743	270
663	5.03	-3.96	1.0000	
18	7	-5	407	294
334	5.03	-3.96	1.0000	
16	7	-5	441	273
437	-1.26	0.24	1.0000	
16	7	-5	178	243
175	-1.26	0.24	1.0000	
14	7	-5	998	247
861	1.52	4.41	1.0000	
14	7	-5	1378	269
1221	1.52	4.41	1.0000	
12	7	-5	2768	247
1637	5.19	9.35	1.0000	
12	7	-5	2285	242
1271	5.19	9.35	1.0000	
10	7	-5	1864	233
1647	-4.26	19.29	1.0000	
10	7	-5	1741	267
1512	-4.26	19.29	1.0000	
8	7	-5	306	190
973	4.14	11.34	1.0000	
8	7	-5	284	160
932	4.14	11.34	1.0000	
6	7	-5	2053	253
2347	13.89	7.74	1.0000	
6	7	-5	1661	205
1890	13.89	7.74	1.0000	
-6	-7	5	1541	194
1750	13.89	-7.74	1.0000	
4	7	-5	366	152
631	-2.93	18.93	1.0000	
4	7	-5	415	143
747	-2.93	18.93	1.0000	
-4	-7	5	383	140
655	-2.93	-18.93	1.0000	
2	7	-5	4831	494
10500	24.83	-17.03	1.0000	
2	7	-5	4999	437
10749	24.83	-17.03	1.0000	
-2	-7	5	4603	488
10184	24.83	17.03	1.0000	
-2	-7	5	4479	472

9998	24.83	17.03	1.0000	
0	7	-5	4729	200
4129	13.50	15.96	1.0000	
0	7	-5	4556	260
3957	13.50	15.96	1.0000	
0	-7	5	4347	240
3725	13.50	-15.96	1.0000	
0	-7	5	4491	240
3868	13.50	-15.96	1.0000	
-2	7	-5	410	100
809	2.98	6.50	1.0000	
-2	7	-5	334	126
700	2.98	6.50	1.0000	
2	-7	5	456	140
872	2.98	-6.50	1.0000	
-4	7	-5	8118	550
12629	-2.38	19.35	1.0000	
-4	7	-5	8692	577
13342	-2.38	19.35	1.0000	
4	-7	5	8357	578
12926	-2.38	-19.35	1.0000	
-6	7	-5	1536	117
809	-5.37	22.92	1.0000	
-6	7	-5	1361	121
633	-5.37	22.92	1.0000	
6	-7	5	1500	130
757	-5.37	-22.92	1.0000	
-8	7	-5	3511	191
2551	-6.45	5.94	1.0000	
-8	7	-5	3701	181
2714	-6.45	5.94	1.0000	
8	-7	5	3537	218
2567	-6.45	-5.94	1.0000	
-10	7	-5	2821	196
2896	-1.16	-0.61	1.0000	
-10	7	-5	2797	194
2871	-1.16	-0.61	1.0000	
10	-7	5	2642	246
2714	-1.16	0.61	1.0000	
-12	7	-5	665	111
337	7.41	-3.11	1.0000	
12	-7	5	530	160
241	7.41	3.11	1.0000	
-14	7	-5	1077	196

1952	-1.00	-13.64	1.0000	
14	-7	5	1220	287
2154	-1.00	13.64	1.0000	
-16	7	-5	1601	183
1403	5.18	0.54	1.0000	
16	-7	5	1885	294
1674	5.18	-0.54	1.0000	
-18	7	-5	168	156
291	-2.16	-4.34	1.0000	
18	-7	5	94	220
186	-2.16	4.34	1.0000	
-20	7	-5	474	189
669	-2.74	2.70	1.0000	
20	-7	5	410	265
593	-2.74	-2.70	1.0000	
22	-7	5	11	267
-273	1.42	-2.53	1.0000	
24	-7	5	316	318
307	-0.15	-0.36	1.0000	
23	6	-5	159	316
132	-0.78	-0.66	1.0000	
23	6	-5	1	325
-58	-0.78	-0.66	1.0000	
21	6	-5	215	306
359	3.84	0.17	1.0000	
21	6	-5	1346	358
1680	3.84	0.17	1.0000	
19	6	-5	2040	338
1902	-7.72	0.15	1.0000	
19	6	-5	596	282
487	-7.72	0.15	1.0000	
17	6	-5	381	269
320	-2.50	-2.18	1.0000	
17	6	-5	762	272
680	-2.50	-2.18	1.0000	
15	6	-5	223	231
284	1.37	7.35	1.0000	
15	6	-5	257	257
328	1.37	7.35	1.0000	
13	6	-5	1669	380
4269	-8.84	-19.48	1.0000	
13	6	-5	1527	351
4040	-8.84	-19.48	1.0000	
11	6	-5	326	207

623	10.49	-9.03	1.0000	
11	6	-5	415	189
776	10.49	-9.03	1.0000	
9	6	-5	753	215
1727	-12.86	-20.47	1.0000	
9	6	-5	939	275
2087	-12.86	-20.47	1.0000	
7	6	-5	1191	129
452	-5.34	-23.01	1.0000	
7	6	-5	1049	169
310	-5.34	-23.01	1.0000	
-7	-6	5	890	138
157	-5.34	23.01	1.0000	
5	6	-5	4164	342
5767	-9.31	3.86	1.0000	
5	6	-5	3728	290
5252	-9.31	3.86	1.0000	
-5	-6	5	3787	296
5320	-9.31	-3.86	1.0000	
3	6	-5	14033	463
10174	-16.33	-16.28	1.0000	
3	6	-5	14052	502
10190	-16.33	-16.28	1.0000	
-3	-6	5	14480	466
10564	-16.33	16.28	1.0000	
1	6	-5	4559	347
6302	-24.25	-1.50	1.0000	
1	6	-5	4588	286
6338	-24.25	-1.50	1.0000	
-1	-6	5	4592	316
6368	-24.25	1.50	1.0000	
-1	-6	5	4625	318
6410	-24.25	1.50	1.0000	
-1	6	-5	1257	98
821	-11.80	8.57	1.0000	
-1	6	-5	1133	129
708	-11.80	8.57	1.0000	
1	-6	5	1302	127
853	-11.80	-8.57	1.0000	
1	-6	5	1301	139
852	-11.80	-8.57	1.0000	
-3	6	-5	5048	190
2245	0.12	-23.37	1.0000	
-3	6	-5	4772	193

2057 0.12 -23.37 1.0000
-3 6 -5 5440 148
2515 0.12 -23.37 1.0000
3 -6 5 5166 171
2335 0.12 23.37 1.0000
-5 6 -5 2880 185
2466 -0.29 -5.23 1.0000
-5 6 -5 2529 152
2141 -0.29 -5.23 1.0000
-5 6 -5 2028 188
1680 -0.29 -5.23 1.0000
5 -6 5 2946 183
2534 -0.29 5.23 1.0000
-7 6 -5 2706 190
3104 13.83 -7.55 1.0000
-7 6 -5 2854 208
3271 13.83 -7.55 1.0000
7 -6 5 2728 223
3128 13.83 7.55 1.0000
-9 6 -5 4308 274
5212 -0.60 -15.28 1.0000
-9 6 -5 4214 274
5105 -0.60 -15.28 1.0000
9 -6 5 4449 313
5392 -0.60 15.28 1.0000
-11 6 -5 1284 190
2559 -4.16 12.24 1.0000
-11 6 -5 1074 171
2259 -4.16 12.24 1.0000
11 -6 5 1307 229
2594 -4.16 -12.24 1.0000
11 -6 5 1020 236
2181 -4.16 -12.24 1.0000
-13 6 -5 768 146
924 3.76 5.40 1.0000
13 -6 5 592 204
726 3.76 -5.40 1.0000
-15 6 -5 551 135
169 -7.72 8.23 1.0000
15 -6 5 490 177
130 -7.72 -8.23 1.0000
-17 6 -5 189 128
126 2.60 -0.83 1.0000
17 -6 5 43 194

15 2.60 0.83 1.0000
-19 6 -5 614 161
553 -0.84 1.96 1.0000
19 -6 5 805 266
736 -0.84 -1.96 1.0000
-21 6 -5 43 149
-7 4.49 -3.46 1.0000
21 -6 5 104 255
46 4.49 3.46 1.0000
23 -6 5 211 277
294 -0.96 2.95 1.0000
24 5 -5 25 367
90 -3.07 2.44 1.0000
24 5 -5 217 365
370 -3.07 2.44 1.0000
22 5 -5 414 329
348 2.84 -2.73 1.0000
22 5 -5 465 312
396 2.84 -2.73 1.0000
20 5 -5 1436 338
1402 -1.57 3.18 1.0000
20 5 -5 384 304
359 -1.57 3.18 1.0000
18 5 -5 9 270
11 -0.12 0.23 1.0000
18 5 -5 374 273
386 -0.12 0.23 1.0000
16 5 -5 947 264
283 13.58 -3.63 1.0000
16 5 -5 730 238
163 13.58 -3.63 1.0000
14 5 -5 98 202
483 -1.48 10.62 1.0000
14 5 -5 136 238
564 -1.48 10.62 1.0000
12 5 -5 465 167
212 3.39 9.44 1.0000
12 5 -5 499 196
239 3.39 9.44 1.0000
10 5 -5 527 149
185 3.57 22.50 1.0000
10 5 -5 538 173
265 3.57 22.50 1.0000
8 5 -5 2274 339

6499	3.31	28.51	1.0000	
8	5	-5	2175	385
6331	3.31	28.51	1.0000	
-8	-5	5	1818	331
5710	3.31	-28.51	1.0000	
6	5	-5	2542	249
2160	-0.27	5.18	1.0000	
6	5	-5	2038	177
1695	-0.27	5.18	1.0000	
-6	-5	5	2040	176
1692	-0.27	-5.18	1.0000	
4	5	-5	3854	170
2299	19.83	-4.56	1.0000	
4	5	-5	4466	250
2806	19.83	-4.56	1.0000	
-4	-5	5	3477	174
1995	19.83	4.56	1.0000	
2	5	-5	2074	223
2472	2.58	12.84	1.0000	
0	5	-5	1253	170
3095	13.70	-23.96	1.0000	
0	5	-5	1256	234
3101	13.70	-23.96	1.0000	
0	-5	5	1311	185
3186	13.70	23.96	1.0000	
0	-5	5	1370	185
3294	13.70	23.96	1.0000	
-2	5	-5	3016	196
2577	8.05	15.66	1.0000	
-2	5	-5	2975	154
2536	8.05	15.66	1.0000	
-2	5	-5	3024	184
2585	8.05	15.66	1.0000	
2	-5	5	3016	162
2577	8.05	-15.66	1.0000	
-4	5	-5	11988	760
17528	-19.66	3.84	1.0000	
-4	5	-5	12757	781
18455	-19.66	3.84	1.0000	
-4	5	-5	13071	780
18833	-19.66	3.84	1.0000	
4	-5	5	13111	775
18885	-19.66	-3.84	1.0000	
-6	5	-5	4575	290

6221	-9.22	-3.73	1.0000	
-6	5	-5	4724	302
6395	-9.22	-3.73	1.0000	
6	-5	5	3639	326
5121	-9.22	3.73	1.0000	
6	-5	5	4326	311
5932	-9.22	3.73	1.0000	
-8	5	-5	2105	145
1662	-11.72	20.55	1.0000	
-8	5	-5	2102	150
1658	-11.72	20.55	1.0000	
8	-5	5	1826	200
1325	-11.72	-20.55	1.0000	
8	-5	5	2006	178
1522	-11.72	-20.55	1.0000	
-10	5	-5	357	95
581	-0.72	-4.47	1.0000	
-10	5	-5	422	118
663	-0.72	-4.47	1.0000	
10	-5	5	331	136
548	-0.72	4.47	1.0000	
10	-5	5	237	142
424	-0.72	4.47	1.0000	
-12	5	-5	1606	121
463	-3.33	-17.96	1.0000	
12	-5	5	1146	154
216	-3.33	17.96	1.0000	
-14	5	-5	270	135
456	4.26	0.39	1.0000	
14	-5	5	193	182
354	4.26	-0.39	1.0000	
-16	5	-5	506	183
1371	-0.12	-15.43	1.0000	
16	-5	5	465	251
1303	-0.12	15.43	1.0000	
-18	5	-5	212	139
268	-3.15	-0.11	1.0000	
18	-5	5	409	231
487	-3.15	0.11	1.0000	
-20	5	-5	9	145
-50	1.16	2.28	1.0000	
20	-5	5	9	221
-162	1.16	-2.28	1.0000	
22	-5	5	140	267

159	-0.40	0.82	1.0000	
24	-5	5	19	298
-175	-1.19	-3.54	1.0000	
25	4	-5	517	379
526	-1.33	1.10	1.0000	
25	4	-5	262	375
267	-1.33	1.10	1.0000	
23	4	-5	36	330
-495	1.96	-4.83	1.0000	
23	4	-5	916	344
600	1.96	-4.83	1.0000	
21	4	-5	21	310
-169	-3.90	0.96	1.0000	
21	4	-5	248	293
149	-3.90	0.96	1.0000	
19	4	-5	2782	417
3089	0.76	-2.38	1.0000	
19	4	-5	1809	347
2058	0.76	-2.38	1.0000	
17	4	-5	2174	373
2329	-4.30	-0.02	1.0000	
17	4	-5	2011	393
2159	-4.30	-0.02	1.0000	
15	4	-5	800	262
1164	-10.65	-4.64	1.0000	
15	4	-5	713	254
1050	-10.65	-4.64	1.0000	
13	4	-5	1514	311
2244	8.39	-3.40	1.0000	
13	4	-5	1215	265
1873	8.39	-3.40	1.0000	
11	4	-5	1584	259
2736	-11.49	-18.92	1.0000	
11	4	-5	1889	305
3183	-11.49	-18.92	1.0000	
9	4	-5	319	208
1599	9.09	-24.80	1.0000	
9	4	-5	410	255
1871	9.09	-24.80	1.0000	
7	4	-5	6943	547
11091	-2.42	-19.11	1.0000	
7	4	-5	6623	505
10685	-2.42	-19.11	1.0000	
-7	-4	5	7693	511

12035 -2.42 19.11 1.0000
5 4 -5 10716 708
15968 -19.51 -4.09 1.0000
5 4 -5 11644 752
17097 -19.51 -4.09 1.0000
-5 -4 5 12365 715
17965 -19.51 4.09 1.0000
3 4 -5 1411 235
2462 -10.32 1.73 1.0000
-3 -4 5 1484 159
2559 -10.32 -1.73 1.0000
1 4 -5 6391 229
2775 -9.29 22.23 1.0000
-1 4 -5 15617 803
19125 -15.49 -6.36 1.0000
-1 4 -5 15502 770
18996 -15.49 -6.36 1.0000
1 -4 5 14859 768
18295 -15.49 6.36 1.0000
-3 4 -5 7752 280
5571 6.16 12.40 1.0000
-3 4 -5 8167 271
5925 6.16 12.40 1.0000
-3 4 -5 8387 302
6114 6.16 12.40 1.0000
3 -4 5 8179 274
5943 6.16 -12.40 1.0000
3 -4 5 7233 302
5136 6.16 -12.40 1.0000
-5 4 -5 4505 167
2804 19.69 4.93 1.0000
-5 4 -5 4339 192
2667 19.69 4.93 1.0000
-5 4 -5 4489 194
2791 19.69 4.93 1.0000
5 -4 5 4674 185
2940 19.69 -4.93 1.0000
5 -4 5 4025 214
2405 19.69 -4.93 1.0000
-7 4 -5 528 122
943 -2.70 -18.84 1.0000
-7 4 -5 682 140
1219 -2.70 -18.84 1.0000
7 -4 5 572 154

1042	-2.70	18.84	1.0000	
7	-4	5	581	148
1058	-2.70	18.84	1.0000	
-9	4	-5	111	129
988	16.55	17.82	1.0000	
-9	4	-5	122	122
1054	16.55	17.82	1.0000	
9	-4	5	108	145
987	16.55	-17.82	1.0000	
9	-4	5	111	156
1005	16.55	-17.82	1.0000	
-11	4	-5	1586	153
1196	8.06	-4.87	1.0000	
11	-4	5	1313	179
950	8.06	4.87	1.0000	
-13	4	-5	325	149
985	-14.38	8.90	1.0000	
13	-4	5	388	210
1108	-14.38	-8.90	1.0000	
-15	4	-5	1286	206
2363	12.06	10.26	1.0000	
15	-4	5	1120	292
2109	12.06	-10.26	1.0000	
-17	4	-5	867	181
1195	-4.38	4.30	1.0000	
17	-4	5	763	254
1070	-4.38	-4.30	1.0000	
-19	4	-5	56	133
142	2.28	-4.08	1.0000	
19	-4	5	281	242
459	2.28	4.08	1.0000	
-21	4	-5	5	144
-70	1.16	1.48	1.0000	
21	-4	5	5	247
-19	1.16	-1.48	1.0000	
23	-4	5	356	308
504	1.96	2.52	1.0000	
25	-4	5	4	336
-119	-1.78	0.06	1.0000	
24	3	-5	2	330
-509	0.54	-1.08	1.0000	
24	3	-5	2	376
-357	0.54	-1.08	1.0000	
22	3	-5	405	308

278	-1.78	6.08	1.0000	
22	3	-5	53	322
-512	-1.78	6.08	1.0000	
20	3	-5	258	301
343	1.46	-1.78	1.0000	
20	3	-5	36	290
71	1.46	-1.78	1.0000	
18	3	-5	9	263
51	7.46	1.19	1.0000	
18	3	-5	175	285
449	7.46	1.19	1.0000	
16	3	-5	2245	383
2907	-1.34	7.32	1.0000	
14	3	-5	351	187
-34	15.14	-5.86	1.0000	
14	3	-5	192	202
537	15.14	-5.86	1.0000	
12	3	-5	1619	276
2589	2.33	21.65	1.0000	
12	3	-5	1512	264
2432	2.33	21.65	1.0000	
10	3	-5	427	152
370	-3.23	12.26	1.0000	
10	3	-5	393	138
327	-3.23	12.26	1.0000	
8	3	-5	2158	278
3926	-8.19	16.04	1.0000	
8	3	-5	1821	240
3452	-8.19	16.04	1.0000	
-8	-3	5	1899	234
3566	-8.19	-16.04	1.0000	
6	3	-5	5160	178
2316	0.00	23.60	1.0000	
6	3	-5	5577	236
2605	0.00	23.60	1.0000	
-6	-3	5	5445	170
2503	0.00	-23.60	1.0000	
4	3	-5	7897	322
5726	6.05	-12.31	1.0000	
2	3	-5	232	198
1719	8.73	-24.23	1.0000	
0	3	-5	8646	493
10669	4.47	-7.74	1.0000	
-2	3	-5	1935	320

6478 -15.42 -27.67 1.0000
-2 3 -5 1790 280
6211 -15.42 -27.67 1.0000
-2 3 -5 1572 278
5799 -15.42 -27.67 1.0000
2 -3 5 1569 271
5795 -15.42 27.67 1.0000
2 -3 5 1661 310
5970 -15.42 27.67 1.0000
-4 3 -5 1133 150
2080 -10.21 -1.76 1.0000
-4 3 -5 1276 179
2272 -10.21 -1.76 1.0000
-4 3 -5 1248 145
2235 -10.21 -1.76 1.0000
4 -3 5 1188 181
2154 -10.21 1.76 1.0000
4 -3 5 1103 147
2038 -10.21 1.76 1.0000
-6 3 -5 15771 498
11768 -16.41 15.97 1.0000
-6 3 -5 15284 507
11341 -16.41 15.97 1.0000
-6 3 -5 15134 495
11210 -16.41 15.97 1.0000
6 -3 5 15799 511
11789 -16.41 -15.97 1.0000
6 -3 5 14798 501
10914 -16.41 -15.97 1.0000
-8 3 -5 2286 119
897 -15.55 -13.34 1.0000
-8 3 -5 1977 117
690 -15.55 -13.34 1.0000
8 -3 5 2481 150
1059 -15.55 13.34 1.0000
8 -3 5 2057 122
765 -15.55 13.34 1.0000
-10 3 -5 703 144
1528 -11.79 -1.84 1.0000
10 -3 5 616 190
1391 -11.79 1.84 1.0000
-12 3 -5 2289 187
2308 2.12 0.72 1.0000
12 -3 5 2120 240

2139 2.12 -0.72 1.0000
-14 3 -5 1207 139
677 1.54 -19.73 1.0000
14 -3 5 1286 212
770 1.54 19.73 1.0000
-16 3 -5 852 155
642 -1.97 -2.83 1.0000
16 -3 5 923 223
704 -1.97 2.83 1.0000
-18 3 -5 556 148
518 2.50 -1.32 1.0000
18 -3 5 814 237
768 2.50 1.32 1.0000
-20 3 -5 15 131
-109 -2.05 -2.61 1.0000
20 -3 5 216 233
119 -2.05 2.61 1.0000
-22 3 -5 61 146
-80 -1.88 6.50 1.0000
22 -3 5 110 277
39 -1.88 -6.50 1.0000
24 -3 5 23 298
-222 3.54 2.22 1.0000
25 2 -5 2 362
-309 0.41 -1.12 1.0000
23 2 -5 12 338
-556 -1.21 2.75 1.0000
23 2 -5 71 332
113 -1.21 2.75 1.0000
21 2 -5 62 288
-463 -0.80 -6.78 1.0000
21 2 -5 66 295
99 -0.80 -6.78 1.0000
19 2 -5 1116 292
980 -0.98 2.47 1.0000
17 2 -5 2356 351
2336 -3.86 1.19 1.0000
17 2 -5 2090 362
2070 -3.86 1.19 1.0000
15 2 -5 426 207
599 -0.95 -3.25 1.0000
15 2 -5 302 199
449 -0.95 -3.25 1.0000
13 2 -5 154 180

518 -8.98 -5.51 1.0000
13 2 -5 228 178
657 -8.98 -5.51 1.0000
11 2 -5 1158 204
1191 5.40 -10.78 1.0000
11 2 -5 1097 220
1124 5.40 -10.78 1.0000
9 2 -5 152 147
540 7.81 -16.31 1.0000
9 2 -5 166 146
595 7.81 -16.31 1.0000
-9 -2 5 132 123
258 7.81 16.31 1.0000
7 2 -5 230 115
547 3.00 -6.54 1.0000
7 2 -5 297 139
648 3.00 -6.54 1.0000
-7 -2 5 248 106
576 3.00 6.54 1.0000
5 2 -5 2897 216
2482 7.90 -15.83 1.0000
3 2 -5 1915 337
6489 -15.37 27.97 1.0000
-1 2 -5 1528 195
2226 12.42 33.17 1.0000
1 -2 5 1279 166
1779 12.42 -33.17 1.0000
-3 2 -5 272 178
1805 8.72 23.97 1.0000
-3 2 -5 208 123
1602 8.72 23.97 1.0000
-3 2 -5 200 115
1575 8.72 23.97 1.0000
3 -2 5 151 146
1408 8.72 -23.97 1.0000
3 -2 5 182 115
1526 8.72 -23.97 1.0000
-5 2 -5 1997 163
2417 2.82 -12.78 1.0000
-5 2 -5 2191 161
2640 2.82 -12.78 1.0000
-5 2 -5 2109 185
2546 2.82 -12.78 1.0000
5 -2 5 2202 170

2645	2.82	12.78	1.0000	
5	-2	5	2137	174
2570	2.82	12.78	1.0000	
-7	2	-5	4605	475
10171	24.72	17.12	1.0000	
-7	2	-5	5070	452
10861	24.72	17.12	1.0000	
-7	2	-5	4530	457
10058	24.72	17.12	1.0000	
7	-2	5	4689	454
10271	24.72	-17.12	1.0000	
7	-2	5	4864	479
10531	24.72	-17.12	1.0000	
-9	2	-5	2169	209
3576	8.07	-12.01	1.0000	
9	-2	5	2036	245
3402	8.07	12.01	1.0000	
-11	2	-5	716	129
797	-1.42	8.78	1.0000	
11	-2	5	545	158
613	-1.42	-8.78	1.0000	
-13	2	-5	548	156
1359	17.49	9.42	1.0000	
13	-2	5	606	234
1473	17.49	-9.42	1.0000	
-15	2	-5	2350	156
1040	-14.03	1.21	1.0000	
15	-2	5	1734	208
645	-14.03	-1.21	1.0000	
-17	2	-5	1058	197
1483	4.34	3.89	1.0000	
17	-2	5	1339	310
1813	4.34	-3.89	1.0000	
-19	2	-5	121	152
170	2.21	4.58	1.0000	
19	-2	5	696	258
851	2.21	-4.58	1.0000	
-21	2	-5	89	134
-128	-1.78	-7.99	1.0000	
21	-2	5	89	254
-26	-1.78	7.99	1.0000	
-23	2	-5	80	180
123	0.77	1.86	1.0000	
23	-2	5	62	293

100	0.77	-1.86	1.0000	
25	-2	5	150	341
172	0.81	-0.56	1.0000	
24	1	-5	12	351
10	2.25	1.93	1.0000	
22	1	-5	2	309
-488	-0.13	1.14	1.0000	
22	1	-5	458	306
403	-0.13	1.14	1.0000	
20	1	-5	390	270
298	1.69	1.65	1.0000	
20	1	-5	253	269
180	1.69	1.65	1.0000	
18	1	-5	1351	277
1531	1.06	2.52	1.0000	
16	1	-5	3049	333
2713	7.43	-8.08	1.0000	
16	1	-5	2915	340
2583	7.43	-8.08	1.0000	
14	1	-5	1237	175
574	2.25	9.52	1.0000	
14	1	-5	902	180
354	2.25	9.52	1.0000	
12	1	-5	911	183
1062	5.43	-5.29	1.0000	
12	1	-5	1208	217
1392	5.43	-5.29	1.0000	
10	1	-5	1018	199
1787	-5.72	15.85	1.0000	
10	1	-5	1103	218
1912	-5.72	15.85	1.0000	
8	1	-5	1558	247
3129	-12.94	13.50	1.0000	
8	1	-5	1712	219
3356	-12.94	13.50	1.0000	
-8	-1	5	1805	211
3491	-12.94	-13.50	1.0000	
6	1	-5	1405	168
940	-11.76	-8.38	1.0000	
6	1	-5	1245	127
795	-11.76	-8.38	1.0000	
-6	-1	5	1391	107
937	-11.76	8.38	1.0000	
4	1	-5	14651	777

18046	-15.26	6.40	1.0000	
2	1	-5	1418	180
2109	12.23	-33.30	1.0000	
0	1	-5	2196	323
6395	-3.77	-35.34	1.0000	
0	-1	5	2186	317
6382	-3.77	35.34	1.0000	
-2	1	-5	1462	142
763	-14.51	-16.21	1.0000	
2	-1	5	1136	97
466	-14.51	16.21	1.0000	
-4	1	-5	6258	157
2729	-9.25	-21.87	1.0000	
-4	1	-5	6119	161
2637	-9.25	-21.87	1.0000	
-4	1	-5	6352	193
2791	-9.25	-21.87	1.0000	
4	-1	5	6220	164
2700	-9.25	21.87	1.0000	
4	-1	5	6190	174
2680	-9.25	21.87	1.0000	
-6	1	-5	4648	306
6453	-24.51	1.62	1.0000	
-6	1	-5	4275	297
5983	-24.51	1.62	1.0000	
-6	1	-5	4433	302
6183	-24.51	1.62	1.0000	
6	-1	5	4531	312
6280	-24.51	-1.62	1.0000	
6	-1	5	4422	294
6143	-24.51	-1.62	1.0000	
-8	1	-5	1507	182
2850	-12.26	9.85	1.0000	
8	-1	5	1696	223
3120	-12.26	-9.85	1.0000	
-10	1	-5	5135	250
4746	-4.83	-0.56	1.0000	
10	-1	5	4766	292
4390	-4.83	0.56	1.0000	
-12	1	-5	158	121
346	-5.41	-6.40	1.0000	
12	-1	5	110	149
256	-5.41	6.40	1.0000	
-14	1	-5	670	146

839	1.82	-6.66	1.0000	
14	-1	5	820	213
1021	1.82	6.66	1.0000	
-16	1	-5	1157	188
1519	5.16	-2.08	1.0000	
16	-1	5	1443	303
1845	5.16	2.08	1.0000	
-18	1	-5	1216	193
1224	-0.08	-4.40	1.0000	
18	-1	5	1213	278
1218	-0.08	4.40	1.0000	
-20	1	-5	677	154
400	1.00	5.49	1.0000	
20	-1	5	473	259
246	1.00	-5.49	1.0000	
-22	1	-5	119	182
176	2.42	-1.30	1.0000	
22	-1	5	129	287
188	2.42	1.30	1.0000	
24	-1	5	26	306
109	-2.67	-3.91	1.0000	
25	0	-5	230	373
288	-0.76	-1.77	1.0000	
23	0	-5	315	324
365	-0.75	-4.57	1.0000	
21	0	-5	39	272
62	2.13	6.48	1.0000	
19	0	-5	2897	406
3670	-3.85	-5.21	1.0000	
19	0	-5	2552	387
3280	-3.85	-5.21	1.0000	
17	0	-5	537	232
697	-2.75	0.81	1.0000	
17	0	-5	694	247
874	-2.75	0.81	1.0000	
15	0	-5	1521	216
1009	4.92	7.11	1.0000	
15	0	-5	2013	269
1424	4.92	7.11	1.0000	
13	0	-5	1280	236
1825	-6.10	-0.41	1.0000	
13	0	-5	1220	233
1753	-6.10	-0.41	1.0000	
11	0	-5	859	203

1698	10.05	-3.24	1.0000	
11	0	-5	933	222
1801	10.05	-3.24	1.0000	
9	0	-5	1131	148
774	5.03	-3.70	1.0000	
9	0	-5	1397	161
998	5.03	-3.70	1.0000	
-9	0	5	1096	130
743	5.03	3.70	1.0000	
7	0	-5	4737	226
4066	13.26	-16.18	1.0000	
7	0	-5	4554	258
3885	13.26	-16.18	1.0000	
-7	0	5	4696	217
4050	13.26	16.18	1.0000	
5	0	-5	1174	214
2944	13.78	24.04	1.0000	
3	0	-5	8174	466
10102	4.29	7.63	1.0000	
1	0	-5	2420	333
6797	-3.92	35.33	1.0000	
-1	0	5	2310	331
6592	-3.92	-35.33	1.0000	
-1	0	-5	2403	330
6757	-3.76	35.22	1.0000	
1	0	5	2455	328
6846	-3.76	-35.22	1.0000	
-3	0	-5	8314	476
10304	4.63	7.67	1.0000	
-3	0	-5	8895	467
10949	4.63	7.67	1.0000	
-3	0	-5	9083	458
11158	4.63	7.67	1.0000	
3	0	5	8809	475
10853	4.63	-7.67	1.0000	
3	0	5	9061	461
11133	4.63	-7.67	1.0000	
-5	0	-5	1388	204
3337	13.80	23.90	1.0000	
-5	0	-5	1487	194
3512	13.80	23.90	1.0000	
-5	0	-5	1366	188
3296	13.80	23.90	1.0000	
5	0	5	1387	203

3349	13.80	-23.90	1.0000	
5	0	5	1466	193
3490	13.80	-23.90	1.0000	
-7	0	-5	4392	234
3757	13.64	-16.27	1.0000	
-7	0	-5	4589	215
3953	13.64	-16.27	1.0000	
7	0	5	4642	243
4030	13.64	16.27	1.0000	
-9	0	-5	1546	129
1116	5.13	-3.34	1.0000	
9	0	5	1436	175
1021	5.13	3.34	1.0000	
-11	0	-5	1043	156
1920	9.89	-2.84	1.0000	
11	0	5	838	222
1641	9.89	2.84	1.0000	
-13	0	-5	1258	173
1765	-5.78	-0.23	1.0000	
13	0	5	1096	244
1571	-5.78	0.23	1.0000	
-15	0	-5	1194	147
764	4.59	6.86	1.0000	
15	0	5	1018	224
617	4.59	-6.86	1.0000	
-17	0	-5	313	149
434	-2.66	0.83	1.0000	
17	0	5	194	234
290	-2.66	-0.83	1.0000	
-19	0	-5	2006	245
2661	-4.16	-5.19	1.0000	
19	0	5	2101	382
2770	-4.16	5.19	1.0000	
-21	0	-5	65	145
-69	2.17	6.66	1.0000	
21	0	5	163	257
283	2.17	-6.66	1.0000	
-23	0	-5	275	183
320	-0.76	-4.55	1.0000	
23	0	5	50	283
50	-0.76	4.55	1.0000	
25	0	5	5	314
-26	-0.81	1.70	1.0000	
24	-1	-5	30	313

-273	-2.47	4.09	1.0000	
22	-1	-5	144	287
211	2.55	-1.41	1.0000	
22	-1	-5	9	294
24	2.55	-1.41	1.0000	
20	-1	-5	821	283
515	1.12	5.37	1.0000	
20	-1	-5	100	243
15	1.12	5.37	1.0000	
18	-1	-5	2141	330
2172	0.05	-4.23	1.0000	
16	-1	-5	1969	313
2422	4.72	-2.18	1.0000	
16	-1	-5	2380	327
2877	4.72	-2.18	1.0000	
14	-1	-5	984	233
1228	1.82	-7.15	1.0000	
14	-1	-5	570	190
744	1.82	-7.15	1.0000	
12	-1	-5	121	138
282	-5.42	-6.61	1.0000	
12	-1	-5	147	139
329	-5.42	-6.61	1.0000	
10	-1	-5	5010	281
4548	-5.03	-0.11	1.0000	
10	-1	-5	5409	301
4930	-5.03	-0.11	1.0000	
8	-1	-5	1309	216
2587	-12.36	10.00	1.0000	
8	-1	-5	1284	185
2551	-12.36	10.00	1.0000	
-8	1	5	1224	178
2469	-12.36	-10.00	1.0000	
6	-1	-5	4705	302
6493	-24.13	1.52	1.0000	
6	-1	-5	4414	339
6128	-24.13	1.52	1.0000	
-6	1	5	4962	299
6786	-24.13	-1.52	1.0000	
4	-1	-5	5948	189
2465	-9.37	-22.42	1.0000	
2	-1	-5	1310	114
631	-14.05	-16.14	1.0000	
0	-1	-5	2825	351

7499	-3.96	-35.29	1.0000	
0	1	5	2638	349
7177	-3.96	35.29	1.0000	
-2	-1	-5	1375	153
2002	12.47	-32.98	1.0000	
2	1	5	1347	148
1806	12.47	32.98	1.0000	
-4	-1	-5	15528	802
19028	-15.56	6.28	1.0000	
-4	-1	-5	16328	803
19917	-15.56	6.28	1.0000	
-4	-1	-5	15669	804
19185	-15.56	6.28	1.0000	
4	1	5	15781	804
19294	-15.56	-6.28	1.0000	
4	1	5	16365	808
19943	-15.56	-6.28	1.0000	
-6	-1	-5	1252	110
801	-11.59	-8.18	1.0000	
-6	-1	-5	1285	119
831	-11.59	-8.18	1.0000	
6	1	5	1005	108
589	-11.59	8.18	1.0000	
6	1	5	1266	126
823	-11.59	8.18	1.0000	
-8	-1	-5	2205	219
4063	-13.25	13.46	1.0000	
8	1	5	2136	263
3967	-13.25	-13.46	1.0000	
-10	-1	-5	1088	155
1867	-5.72	15.59	1.0000	
10	1	5	1080	215
1868	-5.72	-15.59	1.0000	
-12	-1	-5	982	146
1147	5.65	-5.62	1.0000	
12	1	5	854	197
1002	5.65	5.62	1.0000	
-14	-1	-5	1142	131
507	2.40	9.56	1.0000	
14	1	5	923	198
365	2.40	-9.56	1.0000	
-16	-1	-5	2119	203
1785	7.49	-7.63	1.0000	
16	1	5	2564	331

2205	7.49	7.63	1.0000	
-18	-1	-5	1107	196
1280	1.11	2.65	1.0000	
18	1	5	977	276
1140	1.11	-2.65	1.0000	
-20	-1	-5	390	151
300	1.74	1.58	1.0000	
20	1	5	490	263
389	1.74	-1.58	1.0000	
-22	-1	-5	294	179
258	-0.32	0.92	1.0000	
22	1	5	1	268
-19	-0.32	-0.92	1.0000	
-24	-1	-5	61	201
78	2.29	1.99	1.0000	
24	1	5	81	317
103	2.29	-1.99	1.0000	
25	-2	-5	1	339
-223	0.84	0.50	1.0000	
23	-2	-5	5	293
-281	0.75	1.79	1.0000	
23	-2	-5	5	295
-211	0.75	1.79	1.0000	
21	-2	-5	84	253
-70	-1.68	-7.77	1.0000	
21	-2	-5	84	248
-40	-1.68	-7.77	1.0000	
19	-2	-5	226	229
295	1.99	4.58	1.0000	
19	-2	-5	500	248
614	1.99	4.58	1.0000	
17	-2	-5	1075	294
1501	4.65	3.71	1.0000	
17	-2	-5	1322	297
1792	4.65	3.71	1.0000	
15	-2	-5	2712	259
1269	-14.22	1.27	1.0000	
15	-2	-5	2613	212
1202	-14.22	1.27	1.0000	
13	-2	-5	704	224
1683	17.59	9.74	1.0000	
13	-2	-5	705	226
1685	17.59	9.74	1.0000	
11	-2	-5	583	145

655	-1.60	8.66	1.0000	
11	-2	-5	740	151
834	-1.60	8.66	1.0000	
9	-2	-5	2094	253
3517	8.04	-12.29	1.0000	
9	-2	-5	2176	227
3624	8.04	-12.29	1.0000	
-9	2	5	1820	223
3151	8.04	12.29	1.0000	
7	-2	-5	4638	451
10236	24.89	16.87	1.0000	
7	-2	-5	4382	481
9852	24.89	16.87	1.0000	
-7	2	5	4639	449
10213	24.89	-16.87	1.0000	
5	-2	-5	1940	141
2300	2.34	-12.86	1.0000	
5	-2	-5	1940	190
2300	2.34	-12.86	1.0000	
-5	2	5	1884	144
2229	2.34	12.86	1.0000	
3	-2	-5	128	152
1311	8.82	24.18	1.0000	
1	-2	-5	1290	154
1656	12.32	33.01	1.0000	
-1	2	5	1175	146
1477	12.32	-33.01	1.0000	
-1	-2	-5	1232	100
560	-14.19	15.99	1.0000	
1	2	5	1191	103
508	-14.19	-15.99	1.0000	
-3	-2	-5	1635	282
5936	-15.45	27.76	1.0000	
-3	-2	-5	1810	292
6267	-15.45	27.76	1.0000	
3	2	5	1639	292
5942	-15.45	-27.76	1.0000	
-5	-2	-5	2983	162
2547	8.11	-15.91	1.0000	
-5	-2	-5	3134	176
2698	8.11	-15.91	1.0000	
-5	-2	-5	2958	162
2522	8.11	-15.91	1.0000	
5	2	5	2996	188

2560 8.11 15.91 1.0000
-7 -2 -5 378 113
753 2.92 -6.36 1.0000
-7 -2 -5 343 131
703 2.92 -6.36 1.0000
7 2 5 434 145
832 2.92 6.36 1.0000
-9 -2 -5 151 104
542 8.04 -16.11 1.0000
9 2 5 139 140
430 8.04 16.11 1.0000
-11 -2 -5 1151 135
1196 5.48 -10.50 1.0000
11 2 5 965 184
977 5.48 10.50 1.0000
-13 -2 -5 301 142
783 -9.05 -5.22 1.0000
13 2 5 276 197
749 -9.05 5.22 1.0000
-15 -2 -5 67 123
133 -1.03 -2.88 1.0000
15 2 5 12 199
-60 -1.03 2.88 1.0000
-17 -2 -5 1679 203
1641 -3.81 0.98 1.0000
17 2 5 1703 295
1667 -3.81 -0.98 1.0000
-19 -2 -5 1619 210
1459 -0.98 2.42 1.0000
19 2 5 1439 297
1288 -0.98 -2.42 1.0000
-21 -2 -5 126 161
197 -0.82 -6.75 1.0000
21 2 5 61 269
-41 -0.82 6.75 1.0000
-23 -2 -5 75 188
120 -1.34 2.84 1.0000
23 2 5 13 308
-229 -1.34 -2.84 1.0000
25 2 5 2 337
-483 0.48 1.02 1.0000
24 -3 -5 144 314
84 3.56 -2.35 1.0000
22 -3 -5 62 274

```
-84    -1.75     6.59  1.0000
  22   -3   -5        62       266
-103    -1.75     6.59  1.0000
  20   -3   -5       326       243
206    -1.92    -2.66  1.0000
  20   -3   -5        14       221
-212    -1.92    -2.66  1.0000
  18   -3   -5       847       228
793     2.59    -1.48  1.0000
  18   -3   -5      1016       254
958     2.59    -1.48  1.0000
  16   -3   -5       958       200
729    -1.95    -2.92  1.0000
  16   -3   -5       815       209
605    -1.95    -2.92  1.0000
  14   -3   -5      1657       202
1125     1.54   -19.96  1.0000
  14   -3   -5      1353       187
821     1.54   -19.96  1.0000
  12   -3   -5      2347       235
2346     1.79     0.45  1.0000
  12   -3   -5      2019       224
2017     1.79     0.45  1.0000
  10   -3   -5       905       202
1801   -11.59    -2.08  1.0000
  10   -3   -5       798       182
1647   -11.59    -2.08  1.0000
   8   -3   -5      2307       139
898   -15.79   -13.49  1.0000
   8   -3   -5      2306       171
897   -15.79   -13.49  1.0000
  -8    3    5      2335       134
943   -15.79    13.49  1.0000
   6   -3   -5     14328       450
10392   -16.30    16.45  1.0000
   6   -3   -5     13699       484
9848   -16.30    16.45  1.0000
  -6    3    5     14365       454
10421   -16.30   -16.45  1.0000
   4   -3   -5      1665       203
2771   -10.10    -1.83  1.0000
  -4    3    5      1677       172
2786   -10.10     1.83  1.0000
   2   -3   -5      1823       323
```

6341	-15.38	-28.11	1.0000	
0	-3	-5	8201	459
10151	4.36	-7.68	1.0000	
-2	-3	-5	248	135
1753	8.81	-24.00	1.0000	
-2	-3	-5	316	150
1953	8.81	-24.00	1.0000	
-4	-3	-5	8227	295
6017	6.08	-12.15	1.0000	
-4	-3	-5	8381	284
6149	6.08	-12.15	1.0000	
-4	-3	-5	8138	288
5940	6.08	-12.15	1.0000	
4	3	5	7999	296
5814	6.08	12.15	1.0000	
-6	-3	-5	5196	184
2350	-0.03	23.52	1.0000	
-6	-3	-5	4770	154
2059	-0.03	23.52	1.0000	
-6	-3	-5	4866	147
2124	-0.03	23.52	1.0000	
6	3	5	5248	195
2377	-0.03	-23.52	1.0000	
-8	-3	-5	2455	230
4300	-8.07	15.81	1.0000	
8	3	5	2269	276
4050	-8.07	-15.81	1.0000	
-10	-3	-5	316	95
221	-3.25	12.05	1.0000	
10	3	5	263	140
166	-3.25	-12.05	1.0000	
-12	-3	-5	1558	189
2500	2.19	21.89	1.0000	
12	3	5	1490	276
2389	2.19	-21.89	1.0000	
-14	-3	-5	94	118
188	15.53	-6.09	1.0000	
14	3	5	73	209
173	15.53	6.09	1.0000	
-16	-3	-5	1003	191
1434	-1.27	7.16	1.0000	
16	3	5	774	296
1159	-1.27	-7.16	1.0000	
-18	-3	-5	8	144

63	7.52	1.44	1.0000	
18	3	5	168	270
439	7.52	-1.44	1.0000	
-20	-3	-5	601	181
722	1.34	-1.76	1.0000	
20	3	5	857	313
1001	1.34	1.76	1.0000	
-22	-3	-5	106	163
28	-1.71	6.14	1.0000	
22	3	5	54	273
-368	-1.71	-6.14	1.0000	
-24	-3	-5	2	186
-165	0.38	-1.10	1.0000	
24	3	5	197	335
222	0.38	1.10	1.0000	
25	-4	-5	59	338
55	-1.71	-0.03	1.0000	
23	-4	-5	62	292
132	2.05	-2.53	1.0000	
23	-4	-5	14	283
-28	2.05	-2.53	1.0000	
21	-4	-5	181	261
126	1.36	1.42	1.0000	
21	-4	-5	81	239
46	1.36	1.42	1.0000	
19	-4	-5	126	215
248	2.25	-4.03	1.0000	
19	-4	-5	23	230
80	2.25	-4.03	1.0000	
17	-4	-5	1000	256
1356	-4.40	4.36	1.0000	
17	-4	-5	1046	283
1411	-4.40	4.36	1.0000	
15	-4	-5	1396	289
2507	11.96	10.38	1.0000	
15	-4	-5	1580	303
2764	11.96	10.38	1.0000	
13	-4	-5	302	180
942	-14.49	8.95	1.0000	
13	-4	-5	370	189
1086	-14.49	8.95	1.0000	
11	-4	-5	1469	195
1091	8.09	-4.88	1.0000	
11	-4	-5	1474	172

1096	8.09	-4.88	1.0000	
9	-4	-5	94	149
884	16.57	18.21	1.0000	
9	-4	-5	90	153
841	16.57	18.21	1.0000	
7	-4	-5	619	142
1124	-2.84	-19.00	1.0000	
7	-4	-5	658	178
1194	-2.84	-19.00	1.0000	
-7	4	5	685	164
1259	-2.84	19.00	1.0000	
5	-4	-5	3505	148
2044	19.73	4.33	1.0000	
5	-4	-5	3358	190
1924	19.73	4.33	1.0000	
-5	4	5	3455	170
2000	19.73	-4.33	1.0000	
3	-4	-5	8354	280
6142	5.99	12.05	1.0000	
3	-4	-5	8122	315
5942	5.99	12.05	1.0000	
1	-4	-5	15814	835
19337	-15.30	-6.42	1.0000	
1	-4	-5	17504	807
21211	-15.30	-6.42	1.0000	
1	-4	-5	15183	807
18635	-15.30	-6.42	1.0000	
-1	-4	-5	7031	189
3224	-9.33	22.01	1.0000	
-1	-4	-5	6810	200
3074	-9.33	22.01	1.0000	
1	4	5	6956	184
3177	-9.33	-22.01	1.0000	
-3	-4	-5	1755	188
2871	-10.00	1.73	1.0000	
-5	-4	-5	11634	708
17103	-19.60	-4.00	1.0000	
-5	-4	-5	11499	726
16939	-19.60	-4.00	1.0000	
-5	-4	-5	11515	708
16958	-19.60	-4.00	1.0000	
5	4	5	11312	725
16708	-19.60	4.00	1.0000	
-7	-4	-5	6772	491

10863	-2.32	-19.06	1.0000	
7	4	5	7341	525
11581	-2.32	19.06	1.0000	
-9	-4	-5	327	148
1597	8.93	-24.45	1.0000	
9	4	5	347	208
1657	8.93	24.45	1.0000	
-11	-4	-5	1345	174
2362	-11.33	-18.78	1.0000	
11	4	5	1337	259
2350	-11.33	18.78	1.0000	
-13	-4	-5	917	173
1496	8.56	-3.32	1.0000	
13	4	5	1122	261
1753	8.56	3.32	1.0000	
-15	-4	-5	499	161
762	-10.58	-4.44	1.0000	
15	4	5	574	258
859	-10.58	4.44	1.0000	
-17	-4	-5	1237	201
1372	-4.37	0.24	1.0000	
17	4	5	1422	311
1566	-4.37	-0.24	1.0000	
-19	-4	-5	2077	241
2352	0.92	-2.38	1.0000	
19	4	5	1778	369
2033	0.92	2.38	1.0000	
-21	-4	-5	85	165
27	-4.04	0.87	1.0000	
21	4	5	23	283
-13	-4.04	-0.87	1.0000	
-23	-4	-5	66	185
4	2.02	-4.96	1.0000	
23	4	5	38	293
-211	2.02	4.96	1.0000	
-25	-4	-5	5	219
-37	-1.40	1.23	1.0000	
25	4	5	25	362
23	-1.40	-1.23	1.0000	
24	-5	-5	19	310
75	-1.09	3.62	1.0000	
24	-5	-5	19	296
-446	-1.09	3.62	1.0000	
22	-5	-5	264	277

291	-0.48	-0.88	1.0000	
22	-5	-5	162	279
184	-0.48	-0.88	1.0000	
20	-5	-5	45	247
86	1.31	2.50	1.0000	
20	-5	-5	11	210
-178	1.31	2.50	1.0000	
18	-5	-5	141	220
185	-3.19	-0.16	1.0000	
18	-5	-5	339	219
411	-3.19	-0.16	1.0000	
16	-5	-5	605	245
1545	-0.10	-15.47	1.0000	
16	-5	-5	272	210
930	-0.10	-15.47	1.0000	
14	-5	-5	137	162
275	4.26	0.10	1.0000	
14	-5	-5	231	175
402	4.26	0.10	1.0000	
12	-5	-5	1506	155
411	-3.35	-17.87	1.0000	
12	-5	-5	1505	156
410	-3.35	-17.87	1.0000	
10	-5	-5	328	148
568	-1.12	-4.88	1.0000	
10	-5	-5	366	138
617	-1.12	-4.88	1.0000	
8	-5	-5	1825	162
1351	-11.60	20.37	1.0000	
8	-5	-5	1705	184
1218	-11.60	20.37	1.0000	
6	-5	-5	4403	304
6081	-9.30	-4.23	1.0000	
6	-5	-5	4456	338
6143	-9.30	-4.23	1.0000	
-6	5	5	4413	322
6094	-9.30	4.23	1.0000	
4	-5	-5	11134	732
16474	-19.47	4.21	1.0000	
4	-5	-5	11610	702
17052	-19.47	4.21	1.0000	
-4	5	5	11210	716
16570	-19.47	-4.21	1.0000	
2	-5	-5	3523	174

3112	7.92	15.98	1.0000	
2	-5	-5	3135	201
2723	7.92	15.98	1.0000	
2	-5	-5	2820	170
2408	7.92	15.98	1.0000	
0	-5	-5	1460	235
3507	13.95	-24.06	1.0000	
0	-5	-5	2117	220
4617	13.95	-24.06	1.0000	
-2	-5	-5	3437	232
4001	2.56	12.77	1.0000	
2	5	5	3326	224
3886	2.56	-12.77	1.0000	
-6	-5	-5	2232	153
1856	-0.08	5.24	1.0000	
-8	-5	-5	2625	333
7012	3.18	28.14	1.0000	
8	5	5	2543	382
6878	3.18	-28.14	1.0000	
-10	-5	-5	557	91
53	3.71	22.54	1.0000	
10	5	5	594	136
19	3.71	-22.54	1.0000	
-12	-5	-5	414	110
175	3.39	9.29	1.0000	
12	5	5	535	166
259	3.39	-9.29	1.0000	
-14	-5	-5	92	146
483	-1.53	10.89	1.0000	
14	5	5	22	206
285	-1.53	-10.89	1.0000	
-16	-5	-5	1033	153
328	13.69	-3.74	1.0000	
16	5	5	268	230
-38	13.69	3.74	1.0000	
-18	-5	-5	0	144
-1	-0.16	0.14	1.0000	
18	5	5	209	272
216	-0.16	-0.14	1.0000	
-20	-5	-5	789	196
765	-1.50	3.23	1.0000	
20	5	5	497	298
473	-1.50	-3.23	1.0000	
-22	-5	-5	241	190

191	2.83	-2.60	1.0000	
22	5	5	50	282
20	2.83	2.60	1.0000	
-24	-5	-5	430	217
640	-3.08	2.53	1.0000	
24	5	5	21	313
-219	-3.08	-2.53	1.0000	
23	-6	-5	338	316
444	-0.82	-2.95	1.0000	
23	-6	-5	12	285
-72	-0.82	-2.95	1.0000	
21	-6	-5	44	240
-32	4.50	-3.59	1.0000	
21	-6	-5	44	255
-9	4.50	-3.59	1.0000	
19	-6	-5	745	270
674	-0.80	2.01	1.0000	
19	-6	-5	876	232
799	-0.80	2.01	1.0000	
17	-6	-5	444	214
343	2.60	-0.69	1.0000	
17	-6	-5	507	200
399	2.60	-0.69	1.0000	
15	-6	-5	619	177
221	-7.91	8.22	1.0000	
15	-6	-5	338	186
54	-7.91	8.22	1.0000	
13	-6	-5	915	193
1080	3.75	5.26	1.0000	
13	-6	-5	813	185
966	3.75	5.26	1.0000	
11	-6	-5	1015	223
2193	-4.24	12.40	1.0000	
11	-6	-5	1236	247
2513	-4.24	12.40	1.0000	
9	-6	-5	5057	326
6090	-0.42	-15.35	1.0000	
9	-6	-5	4559	339
5529	-0.42	-15.35	1.0000	
7	-6	-5	2504	207
2871	13.73	-7.47	1.0000	
7	-6	-5	2487	233
2852	13.73	-7.47	1.0000	
5	-6	-5	1980	155

1620	-0.03	-5.20	1.0000	
5	-6	-5	1923	182
1568	-0.03	-5.20	1.0000	
-5	6	5	1936	190
1585	-0.03	5.20	1.0000	
3	-6	-5	4470	153
1834	-0.16	-23.78	1.0000	
3	-6	-5	4056	160
1564	-0.16	-23.78	1.0000	
3	-6	-5	4884	184
2111	-0.16	-23.78	1.0000	
1	-6	-5	1414	140
948	-11.57	7.95	1.0000	
1	-6	-5	1313	144
857	-11.57	7.95	1.0000	
1	-6	-5	1577	144
1096	-11.57	7.95	1.0000	
-1	-6	-5	5046	333
6900	-24.44	-1.72	1.0000	
-1	-6	-5	4629	325
6382	-24.44	-1.72	1.0000	
1	6	5	4589	326
6358	-24.44	1.72	1.0000	
-3	-6	-5	16224	565
12107	-16.35	-16.18	1.0000	
-3	-6	-5	17894	577
13576	-16.35	-16.18	1.0000	
3	6	5	17269	579
13028	-16.35	16.18	1.0000	
-5	-6	-5	4562	316
6237	-9.19	4.07	1.0000	
-5	-6	-5	4633	305
6320	-9.19	4.07	1.0000	
5	6	5	4899	341
6629	-9.19	-4.07	1.0000	
-7	-6	-5	1369	116
614	-5.54	-22.78	1.0000	
7	6	5	1616	165
874	-5.54	22.78	1.0000	
-9	-6	-5	907	166
2018	-12.82	-20.31	1.0000	
9	6	5	1011	232
2168	-12.82	20.31	1.0000	
-11	-6	-5	467	132

860	10.54	-9.02	1.0000	
11	6	5	540	196
975	10.54	9.02	1.0000	
-13	-6	-5	1554	258
4057	-8.72	-19.34	1.0000	
13	6	5	1589	353
4117	-8.72	19.34	1.0000	
-15	-6	-5	122	140
144	1.53	7.55	1.0000	
15	6	5	79	217
-81	1.53	-7.55	1.0000	
-17	-6	-5	965	176
870	-2.12	-2.09	1.0000	
17	6	5	879	278
787	-2.12	2.09	1.0000	
-19	-6	-5	161	156
70	-7.73	0.18	1.0000	
19	6	5	758	288
644	-7.73	-0.18	1.0000	
-21	-6	-5	103	182
208	3.86	0.16	1.0000	
21	6	5	146	294
268	3.86	-0.16	1.0000	
-23	-6	-5	366	204
325	-0.78	-0.66	1.0000	
23	6	5	351	330
311	-0.78	0.66	1.0000	
24	-7	-5	0	309
-322	-0.25	0.47	1.0000	
24	-7	-5	0	302
-272	-0.25	0.47	1.0000	
22	-7	-5	50	254
112	1.45	2.73	1.0000	
22	-7	-5	44	293
102	1.45	2.73	1.0000	
20	-7	-5	73	225
157	-2.68	2.70	1.0000	
20	-7	-5	367	273
538	-2.68	2.70	1.0000	
18	-7	-5	302	216
465	-2.08	-4.31	1.0000	
18	-7	-5	158	232
277	-2.08	-4.31	1.0000	
16	-7	-5	2017	290

1804 5.34 0.43 1.0000
16 -7 -5 1933 270
1724 5.34 0.43 1.0000
14 -7 -5 1195 278
2110 -1.18 -13.41 1.0000
14 -7 -5 1577 292
2626 -1.18 -13.41 1.0000
12 -7 -5 995 161
579 7.36 -3.33 1.0000
12 -7 -5 734 159
380 7.36 -3.33 1.0000
10 -7 -5 2825 260
2922 -1.12 -0.79 1.0000
10 -7 -5 2716 238
2811 -1.12 -0.79 1.0000
8 -7 -5 3887 234
2863 -6.54 5.94 1.0000
8 -7 -5 3895 217
2870 -6.54 5.94 1.0000
6 -7 -5 1230 136
495 -5.49 22.88 1.0000
6 -7 -5 1350 128
615 -5.49 22.88 1.0000
4 -7 -5 6170 468
10036 -2.37 18.80 1.0000
4 -7 -5 6432 489
10370 -2.37 18.80 1.0000
2 -7 -5 243 121
558 3.01 6.31 1.0000
2 -7 -5 248 124
565 3.01 6.31 1.0000
0 -7 -5 5755 291
5092 13.43 16.48 1.0000
-2 -7 -5 6588 574
13035 24.81 -16.99 1.0000
2 7 5 6108 567
12379 24.81 16.99 1.0000
-6 -7 -5 2642 186
3039 13.73 7.32 1.0000
6 7 5 2507 232
2886 13.73 -7.32 1.0000
-8 -7 -5 398 139
1129 4.34 11.24 1.0000
8 7 5 481 207

1265 4.34 -11.24 1.0000
-10 -7 -5 1645 155
1403 -4.23 19.25 1.0000
10 7 5 1634 240
1400 -4.23 -19.25 1.0000
-12 -7 -5 2218 160
1211 5.23 9.48 1.0000
12 7 5 2758 260
1616 5.23 -9.48 1.0000
-14 -7 -5 1298 172
1165 1.70 4.21 1.0000
14 7 5 1109 241
983 1.70 -4.21 1.0000
-16 -7 -5 161 148
154 -1.24 0.37 1.0000
16 7 5 104 232
98 -1.24 -0.37 1.0000
-18 -7 -5 695 179
615 4.99 -3.96 1.0000
18 7 5 364 270
293 4.99 3.96 1.0000
-20 -7 -5 245 181
230 1.13 -1.19 1.0000
20 7 5 4 260
-125 1.13 1.19 1.0000
-22 -7 -5 66 204
228 -6.04 -0.42 1.0000
22 7 5 49 266
-468 -6.04 0.42 1.0000
-24 -7 -5 162 233
293 2.66 -3.68 1.0000
24 7 5 28 318
-69 2.66 3.68 1.0000
23 -8 -5 110 308
148 3.98 -0.90 1.0000
23 -8 -5 22 279
-138 3.98 -0.90 1.0000
21 -8 -5 171 254
148 -4.05 -3.10 1.0000
21 -8 -5 303 280
284 -4.05 -3.10 1.0000
19 -8 -5 165 245
423 4.84 -4.72 1.0000
19 -8 -5 82 234

281 4.84 -4.72 1.0000

17 -8 -5 46 190

0 -0.20 5.91 1.0000

17 -8 -5 46 212

-14 -0.20 5.91 1.0000

15 -8 -5 127 175

338 -5.33 -3.19 1.0000

15 -8 -5 33 188

164 -5.33 -3.19 1.0000

13 -8 -5 450 203

960 -7.28 6.58 1.0000

13 -8 -5 326 189

768 -7.28 6.58 1.0000

11 -8 -5 4408 309

4501 -2.39 0.72 1.0000

11 -8 -5 4192 321

4282 -2.39 0.72 1.0000

9 -8 -5 4107 329

5162 -3.18 -7.17 1.0000

9 -8 -5 4131 312

5189 -3.18 -7.17 1.0000

7 -8 -5 218 169

818 4.32 -11.45 1.0000

7 -8 -5 380 160

1113 4.32 -11.45 1.0000

5 -8 -5 2871 374

7448 3.13 -28.33 1.0000

3 -8 -5 1350 231

2736 -8.00 -15.85 1.0000

3 -8 -5 2217 237

3973 -8.00 -15.85 1.0000

1 -8 -5 1984 254

3761 -13.34 -13.51 1.0000

1 -8 -5 2136 261

3977 -13.34 -13.51 1.0000

1 8 5 2152 252

3775 -12.18 10.16 1.0000

3 8 5 3464 197

1735 -15.64 -13.25 1.0000

-7 -8 -5 3295 181

2369 -6.41 -5.77 1.0000

7 8 5 4049 240

3024 -6.41 5.77 1.0000

-9 -8 -5 200 109

186	4.31	-4.82	1.0000	
9	8	5	274	174
254	4.31	4.82	1.0000	
-11	-8	-5	80	135
875	-3.72	-19.67	1.0000	
11	8	5	66	225
813	-3.72	19.67	1.0000	
-13	-8	-5	863	172
1241	3.92	9.17	1.0000	
13	8	5	638	250
946	3.92	-9.17	1.0000	
-15	-8	-5	189	160
583	-1.33	-9.13	1.0000	
15	8	5	287	273
748	-1.33	9.13	1.0000	
-17	-8	-5	1904	237
2160	-4.13	4.84	1.0000	
17	8	5	2069	397
2338	-4.13	-4.84	1.0000	
-19	-8	-5	19	167
-160	1.05	3.60	1.0000	
19	8	5	422	293
296	1.05	-3.60	1.0000	
-21	-8	-5	7	192
27	-1.63	-3.32	1.0000	
21	8	5	36	281
84	-1.63	3.32	1.0000	
-23	-8	-5	79	221
176	-0.96	5.36	1.0000	
23	8	5	40	304
-141	-0.96	-5.36	1.0000	
22	-9	-5	423	317
568	0.48	3.10	1.0000	
22	-9	-5	199	274
301	0.48	3.10	1.0000	
20	-9	-5	651	276
612	0.01	-0.67	1.0000	
20	-9	-5	514	256
480	0.01	-0.67	1.0000	
18	-9	-5	606	251
715	2.08	1.24	1.0000	
18	-9	-5	466	231
562	2.08	1.24	1.0000	
16	-9	-5	275	202

239	-3.12	0.90	1.0000	
16	-9	-5	199	214
166	-3.12	0.90	1.0000	
14	-9	-5	602	186
283	6.77	-4.28	1.0000	
14	-9	-5	490	182
206	6.77	-4.28	1.0000	
12	-9	-5	853	173
530	4.71	-4.52	1.0000	
12	-9	-5	1138	194
762	4.71	-4.52	1.0000	
10	-9	-5	996	160
525	1.98	7.27	1.0000	
10	-9	-5	1054	156
568	1.98	7.27	1.0000	
8	-9	-5	76	134
34	4.48	4.81	1.0000	
8	-9	-5	146	113
118	4.48	4.81	1.0000	
6	-9	-5	967	201
2097	-12.88	20.38	1.0000	
4	-9	-5	295	177
1493	9.04	24.52	1.0000	
2	-9	-5	133	141
382	7.87	16.00	1.0000	
0	-9	-5	1570	182
1127	5.24	3.39	1.0000	
2	9	5	2141	258
3558	7.88	-12.15	1.0000	
4	9	5	413	216
1990	16.62	17.80	1.0000	
-6	-9	-5	4984	316
6013	-0.50	15.29	1.0000	
6	9	5	6145	388
7288	-0.50	-15.29	1.0000	
-8	-9	-5	4224	263
5274	-3.07	7.04	1.0000	
-8	-9	-5	3053	243
3951	-3.07	7.04	1.0000	
8	9	5	4106	332
5141	-3.07	-7.04	1.0000	
-10	-9	-5	91	115
206	4.30	0.27	1.0000	
10	9	5	79	190

189 4.30 -0.27 1.0000
-12 -9 -5 960 130
282 -11.10 11.50 1.0000
12 9 5 824 223
193 -11.10 -11.50 1.0000
-14 -9 -5 1300 208
1997 5.90 -5.52 1.0000
14 9 5 1372 343
2086 5.90 5.52 1.0000
-16 -9 -5 628 182
582 -0.91 -0.69 1.0000
16 9 5 470 262
430 -0.91 0.69 1.0000
-18 -9 -5 23 172
-37 -4.12 -0.73 1.0000
18 9 5 136 291
257 -4.12 0.73 1.0000
-20 -9 -5 466 215
847 0.71 -6.51 1.0000
20 9 5 140 308
374 0.71 6.51 1.0000
-22 -9 -5 76 210
78 2.37 -1.54 1.0000
22 9 5 353 350
369 2.37 1.54 1.0000
23 -10 -5 19 279
-104 -3.04 -2.30 1.0000
23 -10 -5 235 328
120 -3.04 -2.30 1.0000
21 -10 -5 69 271
199 5.42 -1.19 1.0000
21 -10 -5 18 287
96 5.42 -1.19 1.0000
19 -10 -5 148 231
150 -1.78 -0.20 1.0000
19 -10 -5 226 255
230 -1.78 -0.20 1.0000
17 -10 -5 227 218
53 -6.17 -7.01 1.0000
17 -10 -5 239 202
61 -6.17 -7.01 1.0000
15 -10 -5 201 210
340 1.45 4.74 1.0000
15 -10 -5 240 193

392	1.45	4.74	1.0000	
13	-10	-5	87	179
45	-5.65	1.41	1.0000	
13	-10	-5	92	167
50	-5.65	1.41	1.0000	
11	-10	-5	216	168
495	-11.21	-7.45	1.0000	
11	-10	-5	171	164
392	-11.21	-7.45	1.0000	
9	-10	-5	78	145
191	4.45	-0.30	1.0000	
7	-10	-5	1809	202
1590	-4.18	-19.32	1.0000	
5	-10	-5	511	117
144	3.58	-22.46	1.0000	
3	-10	-5	344	122
281	-3.33	-12.20	1.0000	
1	-10	-5	982	212
1744	-5.69	-15.81	1.0000	
1	10	5	4931	291
4518	-5.03	-0.46	1.0000	
3	10	5	1528	249
2683	-11.79	-1.82	1.0000	
-7	-10	-5	2779	198
2846	-1.22	0.55	1.0000	
-9	-10	-5	979	135
510	2.31	-7.27	1.0000	
9	10	5	1322	212
765	2.31	7.27	1.0000	
-11	-10	-5	12	114
136	4.94	6.14	1.0000	
11	10	5	17	199
148	4.94	-6.14	1.0000	
-13	-10	-5	284	156
292	1.55	-7.27	1.0000	
13	10	5	223	257
220	1.55	7.27	1.0000	
-15	-10	-5	1035	217
1611	-1.96	8.09	1.0000	
-17	-10	-5	557	186
609	1.55	0.96	1.0000	
17	10	5	329	283
369	1.55	-0.96	1.0000	
-19	-10	-5	151	189

130 0.57 0.52 1.0000
19 10 5 521 311
481 0.57 -0.52 1.0000
-21 -10 -5 28 215
22 -4.17 7.16 1.0000
21 10 5 41 309
216 -4.17 -7.16 1.0000
-23 -10 -5 17 246
7 1.47 -1.11 1.0000
23 10 5 5 342
-30 1.47 1.11 1.0000
22 -11 -5 6 281
-42 -1.25 -1.63 1.0000
22 -11 -5 6 301
-118 -1.25 -1.63 1.0000
20 -11 -5 430 293
707 -1.06 5.41 1.0000
20 -11 -5 222 262
429 -1.06 5.41 1.0000
18 -11 -5 54 220
150 2.94 3.31 1.0000
18 -11 -5 247 259
428 2.94 3.31 1.0000
16 -11 -5 35 189
-196 2.45 -4.52 1.0000
16 -11 -5 35 200
-184 2.45 -4.52 1.0000
14 -11 -5 344 196
508 0.72 3.38 1.0000
14 -11 -5 259 210
403 0.72 3.38 1.0000
12 -11 -5 2757 310
3141 3.82 -0.06 1.0000
12 -11 -5 2839 308
3229 3.82 -0.06 1.0000
10 -11 -5 19 163
153 5.06 -5.92 1.0000
8 -11 -5 33 162
604 -4.02 19.67 1.0000
6 -11 -5 342 159
647 10.54 8.97 1.0000
4 -11 -5 1343 224
2349 -11.44 18.71 1.0000
2 -11 -5 1051 177

1062	5.40	10.71	1.0000	
0	-11	-5	1037	240
1908	9.82	2.83	1.0000	
2	11	5	1167	209
1293	-1.33	8.83	1.0000	
4	11	5	1214	181
865	8.09	-4.87	1.0000	
6	11	5	2883	334
4681	-4.14	12.20	1.0000	
-8	-11	-5	4300	257
4434	-2.54	-0.49	1.0000	
8	11	5	4077	353
4207	-2.54	0.49	1.0000	
-10	-11	-5	362	148
753	-11.15	7.23	1.0000	
10	11	5	217	220
485	-11.15	-7.23	1.0000	
-12	-11	-5	643	161
798	5.06	-7.03	1.0000	
12	11	5	400	215
508	5.06	7.03	1.0000	
-14	-11	-5	2063	251
3343	-10.65	3.95	1.0000	
14	11	5	1817	388
3030	-10.65	-3.95	1.0000	
-16	-11	-5	2221	237
1796	0.61	-4.09	1.0000	
16	11	5	1932	341
1538	0.61	4.09	1.0000	
-18	-11	-5	319	194
299	0.55	-6.07	1.0000	
18	11	5	245	314
226	0.55	6.07	1.0000	
-20	-11	-5	22	212
-39	-3.83	1.29	1.0000	
20	11	5	22	333
-20	-3.83	-1.29	1.0000	
-22	-11	-5	581	233
231	2.12	-7.62	1.0000	
22	11	5	83	339
-172	2.12	7.62	1.0000	
21	-12	-5	209	314
327	0.97	-3.98	1.0000	
21	-12	-5	436	285

605 0.97 -3.98 1.0000
19 -12 -5 132 235
255 -2.50 -3.03 1.0000
19 -12 -5 61 252
151 -2.50 -3.03 1.0000
17 -12 -5 358 244
424 2.01 -0.31 1.0000
17 -12 -5 627 237
715 2.01 -0.31 1.0000
15 -12 -5 7 175
61 -5.31 -2.99 1.0000
15 -12 -5 21 207
7 -5.31 -2.99 1.0000
13 -12 -5 124 188
308 -8.98 -1.19 1.0000
13 -12 -5 78 201
212 -8.98 -1.19 1.0000
11 -12 -5 605 195
769 5.22 7.07 1.0000
9 -12 -5 1214 189
431 -11.24 -11.61 1.0000
7 -12 -5 2249 216
1243 5.20 -9.33 1.0000
5 -12 -5 472 152
205 3.30 -9.46 1.0000
3 -12 -5 1289 241
2096 2.36 -21.73 1.0000
1 -12 -5 1090 207
1258 5.46 5.45 1.0000
3 12 5 2369 264
2389 1.83 0.66 1.0000
-7 -12 -5 843 136
480 7.32 3.00 1.0000
-9 -12 -5 848 141
535 4.73 4.35 1.0000
9 12 5 689 219
408 4.73 -4.35 1.0000
-11 -12 -5 2697 220
3093 4.01 0.10 1.0000
11 12 5 2106 350
2459 4.01 -0.10 1.0000
-13 -12 -5 989 176
850 0.40 2.08 1.0000
13 12 5 559 234

455 0.40 -2.08 1.0000
-15 -12 -5 864 185
694 2.60 0.46 1.0000
15 12 5 286 239
192 2.60 -0.46 1.0000
-17 -12 -5 219 190
109 0.05 3.77 1.0000
17 12 5 19 273
-77 0.05 -3.77 1.0000
-19 -12 -5 334 218
179 -3.34 2.86 1.0000
19 12 5 358 319
197 -3.34 -2.86 1.0000
-21 -12 -5 349 247
513 2.42 3.15 1.0000
21 12 5 21 338
-120 2.42 -3.15 1.0000
20 -13 -5 131 281
85 2.06 -1.55 1.0000
20 -13 -5 632 279
529 2.06 -1.55 1.0000
18 -13 -5 37 220
-196 -5.05 1.45 1.0000
18 -13 -5 37 232
-147 -5.05 1.45 1.0000
16 -13 -5 616 245
455 1.69 7.19 1.0000
16 -13 -5 692 213
525 1.69 7.19 1.0000
14 -13 -5 373 205
402 4.34 -5.85 1.0000
14 -13 -5 585 220
641 4.34 -5.85 1.0000
12 -13 -5 727 221
609 0.17 -2.03 1.0000
10 -13 -5 193 187
177 1.33 7.26 1.0000
8 -13 -5 825 223
1181 3.91 -9.00 1.0000
6 -13 -5 1393 304
3799 -8.70 19.37 1.0000
4 -13 -5 1012 236
1609 8.32 3.43 1.0000
2 -13 -5 89 180

388	-9.04	5.24	1.0000	
0	-13	-5	1195	251
1703	-5.96	0.17	1.0000	
2	13	5	678	240
1615	17.46	9.56	1.0000	
4	13	5	310	214
957	-14.39	8.97	1.0000	
6	13	5	884	229
1053	3.80	5.39	1.0000	
-8	-13	-5	277	153
689	-7.15	-6.61	1.0000	
8	13	5	689	254
1295	-7.15	6.61	1.0000	
-10	-13	-5	444	139
400	-5.63	-1.22	1.0000	
10	13	5	624	248
578	-5.63	1.22	1.0000	
-12	-13	-5	387	167
726	-9.06	1.15	1.0000	
12	13	5	69	253
194	-9.06	-1.15	1.0000	
-14	-13	-5	793	178
753	2.40	-1.37	1.0000	
14	13	5	446	265
413	2.40	1.37	1.0000	
-16	-13	-5	472	205
635	-5.90	-1.36	1.0000	
16	13	5	289	246
411	-5.90	1.36	1.0000	
-18	-13	-5	599	224
546	-1.25	1.09	1.0000	
18	13	5	186	279
156	-1.25	-1.09	1.0000	
-20	-13	-5	100	242
328	4.12	-6.18	1.0000	
20	13	5	112	340
350	4.12	6.18	1.0000	
21	-14	-5	43	289
-288	-4.02	4.04	1.0000	
21	-14	-5	158	286
341	-4.02	4.04	1.0000	
19	-14	-5	16	253
-32	3.05	-1.62	1.0000	
19	-14	-5	16	256

-285 3.05 -1.62 1.0000
17 -14 -5 26 238
-70 1.63 -4.14 1.0000
17 -14 -5 95 228
204 1.63 -4.14 1.0000
15 -14 -5 109 217
242 -7.01 1.11 1.0000
15 -14 -5 132 220
280 -7.01 1.11 1.0000
13 -14 -5 547 227
515 2.45 1.51 1.0000
11 -14 -5 2131 349
3433 -10.67 -3.91 1.0000
9 -14 -5 1510 299
2262 5.92 5.62 1.0000
7 -14 -5 1164 224
1024 1.52 -4.22 1.0000
5 -14 -5 31 184
310 -1.46 -10.75 1.0000
3 -14 -5 131 178
91 15.23 5.83 1.0000
1 -14 -5 474 182
112 2.32 -9.47 1.0000
-1 -14 -5 812 250
1027 1.80 6.93 1.0000
3 14 5 1391 208
857 1.49 -19.64 1.0000
5 14 5 60 212
159 4.23 0.30 1.0000
-7 -14 -5 1544 212
2582 -1.11 13.43 1.0000
7 14 5 1277 308
2220 -1.11 -13.43 1.0000
-9 -14 -5 82 135
-8 6.61 4.23 1.0000
9 14 5 578 230
272 6.61 -4.23 1.0000
-11 -14 -5 411 160
598 0.86 -3.52 1.0000
11 14 5 210 269
349 0.86 3.52 1.0000
-13 -14 -5 537 186
586 4.27 5.84 1.0000
13 14 5 394 255

428 4.27 -5.84 1.0000
-15 -14 -5 550 189
510 1.09 -1.92 1.0000
15 14 5 604 286
561 1.09 1.92 1.0000
-17 -14 -5 231 214
168 -3.08 0.67 1.0000
17 14 5 581 283
482 -3.08 -0.67 1.0000
-19 -14 -5 73 236
145 0.47 4.90 1.0000
19 14 5 32 265
-62 0.47 -4.90 1.0000
-21 -14 -5 58 258
83 0.38 2.31 1.0000
21 14 5 206 316
255 0.38 -2.31 1.0000
20 -15 -5 56 269
36 0.16 -1.38 1.0000
20 -15 -5 47 295
29 0.16 -1.38 1.0000
18 -15 -5 279 264
311 0.81 -0.09 1.0000
18 -15 -5 87 230
105 0.81 -0.09 1.0000
16 -15 -5 22 210
-271 -3.00 -2.77 1.0000
16 -15 -5 113 251
217 -3.00 -2.77 1.0000
14 -15 -5 471 217
440 0.94 1.96 1.0000
12 -15 -5 474 220
354 2.54 -0.42 1.0000
10 -15 -5 798 255
1309 -2.10 -8.06 1.0000
8 -15 -5 179 214
564 -1.41 9.11 1.0000
6 -15 -5 229 206
299 1.34 -7.38 1.0000
4 -15 -5 399 204
610 -10.61 4.54 1.0000
2 -15 -5 78 202
153 -0.99 3.00 1.0000
0 -15 -5 1003 227

599	4.68	-7.01	1.0000	
4	15	5	1216	319
2254	11.97	10.46	1.0000	
6	15	5	674	220
253	-7.81	8.22	1.0000	
-8	-15	-5	138	159
350	-5.28	3.05	1.0000	
8	15	5	147	232
365	-5.28	-3.05	1.0000	
-10	-15	-5	211	157
351	1.49	-4.62	1.0000	
10	15	5	53	247
124	1.49	4.62	1.0000	
-12	-15	-5	118	178
301	-5.26	3.22	1.0000	
12	15	5	39	264
157	-5.26	-3.22	1.0000	
-14	-15	-5	299	191
526	-7.02	-1.13	1.0000	
14	15	5	183	265
361	-7.02	1.13	1.0000	
-16	-15	-5	89	208
151	3.31	2.49	1.0000	
16	15	5	218	300
322	3.31	-2.49	1.0000	
-18	-15	-5	48	233
66	0.14	-2.26	1.0000	
18	15	5	7	308
-174	0.14	2.26	1.0000	
-20	-15	-5	129	259
287	-4.51	-1.82	1.0000	
20	15	5	162	345
336	-4.51	1.82	1.0000	
19	-16	-5	10	287
-36	-0.75	2.64	1.0000	
19	-16	-5	138	268
198	-0.75	2.64	1.0000	
17	-16	-5	13	254
-108	-2.91	1.28	1.0000	
17	-16	-5	208	240
136	-2.91	1.28	1.0000	
15	-16	-5	23	230
-131	3.27	-2.51	1.0000	
13	-16	-5	197	234

```
295   -5.93    1.40  1.0000
  11 -16  -5     2042      279
1620     0.60     4.26  1.0000
   9 -16  -5      626      230
580   -0.91    0.78  1.0000
   7 -16  -5       19      207
15   -1.27   -0.37  1.0000
   5 -16  -5     1001      202
315    13.62    3.76  1.0000
   3 -16  -5      940      263
1362    -1.27   -7.13  1.0000
   1 -16  -5     2551      338
2209     7.41     7.77  1.0000
  -1 -16  -5      810      270
1098     4.89     2.01  1.0000
   3  16   5     1373      244
1100    -1.92   -2.89  1.0000
   5  16   5      242      252
870   -0.22  -15.49  1.0000
  -7 -16  -5     1692      204
1492     5.22   -0.52  1.0000
   7  16   5     1671      278
1470     5.22     0.52  1.0000
  -9 -16  -5      330      159
290   -3.12   -0.92  1.0000
   9  16   5      345      249
305   -3.12    0.92  1.0000
 -11 -16  -5       72      162
124     2.27    4.42  1.0000
  11  16   5       33      235
-222     2.27   -4.42  1.0000
 -13 -16  -5      912      216
729     1.81   -7.22  1.0000
  13  16   5     1230      322
1033     1.81     7.22  1.0000
 -15 -16  -5      182      201
311   -3.04    2.79  1.0000
  15  16   5       23      261
-226    -3.04   -2.79  1.0000
 -17 -16  -5       87      239
148     1.08    2.32  1.0000
  17  16   5        9      290
-92     1.08   -2.32  1.0000
 -19 -16  -5      315      268
```

422 1.71 1.87 1.0000
19 16 5 9 321
-302 1.71 -1.87 1.0000
18 -17 -5 9 286
-385 1.15 -2.33 1.0000
18 -17 -5 9 248
-59 1.15 -2.33 1.0000
16 -17 -5 209 261
301 1.15 -2.35 1.0000
14 -17 -5 380 236
301 -3.10 -0.60 1.0000
12 -17 -5 19 221
-124 0.00 -3.81 1.0000
10 -17 -5 568 234
616 1.42 -0.87 1.0000
8 -17 -5 1850 336
2090 -4.25 -4.75 1.0000
6 -17 -5 811 253
722 -2.27 2.16 1.0000
4 -17 -5 1442 292
1577 -4.41 -0.10 1.0000
2 -17 -5 1832 309
1803 -3.92 -1.10 1.0000
0 -17 -5 272 261
386 -2.73 -0.78 1.0000
-2 -17 -5 992 335
1398 4.39 -3.80 1.0000
4 17 5 990 273
1350 -4.41 4.41 1.0000
6 17 5 215 247
145 2.59 -0.56 1.0000
-8 -17 -5 45 153
-112 -0.18 -5.79 1.0000
8 17 5 45 213
-288 -0.18 5.79 1.0000
-10 -17 -5 424 166
199 -6.14 6.95 1.0000
10 17 5 114 230
-213 -6.14 -6.95 1.0000
-12 -17 -5 631 213
722 2.04 0.37 1.0000
12 17 5 694 284
790 2.04 -0.37 1.0000
-14 -17 -5 70 202

174 1.70 4.31 1.0000
14 17 5 27 278
95 1.70 -4.31 1.0000
-16 -17 -5 13 226
-41 -2.91 -1.27 1.0000
16 17 5 78 313
35 -2.91 1.27 1.0000
-18 -17 -5 3 251
-9 -1.28 -0.91 1.0000
18 17 5 3 303
-244 -1.28 0.91 1.0000
17 -18 -5 154 249
130 -1.24 0.91 1.0000
17 -18 -5 272 300
241 -1.24 0.91 1.0000
15 -18 -5 427 263
485 0.09 2.33 1.0000
13 -18 -5 4 233
-14 -1.28 -1.05 1.0000
11 -18 -5 49 215
-173 0.50 6.05 1.0000
9 -18 -5 217 231
370 -4.17 0.83 1.0000
7 -18 -5 195 227
128 4.98 4.03 1.0000
5 -18 -5 138 227
144 -0.14 -0.17 1.0000
3 -18 -5 51 233
3 7.45 -1.34 1.0000
1 -18 -5 981 297
1129 1.20 -2.47 1.0000
-1 -18 -5 1067 293
1076 0.02 4.21 1.0000
5 18 5 258 226
323 -3.17 -0.05 1.0000
-7 -18 -5 557 182
785 -2.11 4.44 1.0000
7 18 5 211 267
354 -2.11 -4.44 1.0000
-9 -18 -5 796 197
917 2.03 -1.24 1.0000
9 18 5 254 258
323 2.03 1.24 1.0000
-11 -18 -5 238 194

416 3.00 -3.25 1.0000
11 18 5 223 261
396 3.00 3.25 1.0000
-13 -18 -5 40 212
-2 -5.27 -1.47 1.0000
13 18 5 40 252
-30 -5.27 1.47 1.0000
-15 -18 -5 549 224
591 0.77 0.00 1.0000
15 18 5 48 312
61 0.77 0.00 1.0000
-17 -18 -5 211 260
297 1.27 2.24 1.0000
17 18 5 112 350
175 1.27 -2.24 1.0000
16 -19 -5 8 268
-61 1.61 -1.91 1.0000
14 -19 -5 181 262
300 0.44 -4.90 1.0000
12 -19 -5 93 232
22 -3.41 -2.80 1.0000
10 -19 -5 629 273
586 0.56 -0.52 1.0000
8 -19 -5 18 218
-124 1.04 -3.49 1.0000
6 -19 -5 527 252
423 -7.73 -0.10 1.0000
4 -19 -5 2297 387
2582 0.82 2.40 1.0000
2 -19 -5 1449 303
1301 -1.04 -2.40 1.0000
0 -19 -5 2317 413
3018 -4.08 5.21 1.0000
-2 -19 -5 186 285
248 1.99 -4.62 1.0000
-6 -19 -5 502 189
448 -0.92 -2.00 1.0000
6 19 5 780 260
713 -0.92 2.00 1.0000
-8 -19 -5 235 190
524 4.80 4.60 1.0000
8 19 5 59 258
-70 4.80 -4.60 1.0000
-10 -19 -5 74 195

74	-1.90	0.24	1.0000	
10	19	5	167	277
170	-1.90	-0.24	1.0000	
-12	-19	-5	343	234
529	-2.53	2.99	1.0000	
12	19	5	121	278
239	-2.53	-2.99	1.0000	
-14	-19	-5	263	229
190	3.11	1.58	1.0000	
14	19	5	58	282
22	3.11	-1.58	1.0000	
-16	-19	-5	174	241
239	-0.71	-2.56	1.0000	
16	19	5	9	313
-277	-0.71	2.56	1.0000	
15	-20	-5	31	283
-67	-4.46	1.78	1.0000	
13	-20	-5	74	264
-20	4.14	6.19	1.0000	
11	-20	-5	281	262
295	-3.81	-1.26	1.0000	
9	-20	-5	213	270
492	0.65	6.58	1.0000	
7	-20	-5	3	231
-108	1.04	1.21	1.0000	
5	-20	-5	673	272
644	-1.54	-3.17	1.0000	
3	-20	-5	625	296
757	1.43	1.86	1.0000	
1	-20	-5	473	289
371	1.69	-1.67	1.0000	
-1	-20	-5	331	284
149	1.11	-5.33	1.0000	
-3	-20	-5	15	288
-353	-2.01	2.66	1.0000	
-7	-20	-5	535	209
750	-2.83	-2.80	1.0000	
7	20	5	325	263
496	-2.83	2.80	1.0000	
-9	-20	-5	629	206
590	-0.05	0.69	1.0000	
9	20	5	651	311
611	-0.05	-0.69	1.0000	
-11	-20	-5	442	231

713	-1.06	-5.25	1.0000	
11	20	5	84	284
216	-1.06	5.25	1.0000	
-13	-20	-5	633	240
522	2.19	1.53	1.0000	
13	20	5	332	295
253	2.19	-1.53	1.0000	
15	20	5	110	311
81	0.10	-1.38	1.0000	
14	-21	-5	166	303
212	0.28	-2.33	1.0000	
12	-21	-5	213	272
339	2.41	-3.05	1.0000	
10	-21	-5	26	270
172	-4.14	-7.07	1.0000	
8	-21	-5	18	248
-112	-1.63	3.29	1.0000	
6	-21	-5	289	270
453	3.86	-0.10	1.0000	
4	-21	-5	303	260
188	-3.96	-0.87	1.0000	
2	-21	-5	213	279
329	-0.84	6.82	1.0000	
0	-21	-5	324	299
496	2.04	-6.52	1.0000	
-2	-21	-5	86	277
-214	-1.75	7.82	1.0000	
-4	-21	-5	71	324
40	1.17	-1.43	1.0000	
-6	-21	-5	393	223
315	4.46	3.54	1.0000	
6	21	5	419	270
332	4.46	-3.54	1.0000	
-8	-21	-5	968	222
954	-4.02	3.19	1.0000	
8	21	5	572	312
554	-4.02	-3.19	1.0000	
10	21	5	98	304
249	5.46	-1.09	1.0000	
12	21	5	16	302
48	0.91	-4.02	1.0000	
14	21	5	13	312
82	-4.15	4.15	1.0000	
11	-22	-5	711	305

315 2.10 7.65 1.0000
9 -22 -5 662 301
689 2.31 1.54 1.0000
7 -22 -5 20 269
131 -6.01 0.57 1.0000
5 -22 -5 207 276
156 2.82 2.71 1.0000
3 -22 -5 501 280
362 -1.77 -6.09 1.0000
1 -22 -5 550 287
499 -0.21 -0.95 1.0000
-1 -22 -5 224 308
306 2.51 1.39 1.0000
-3 -22 -5 141 313
69 -1.81 -6.55 1.0000
-5 -22 -5 23 358
30 -0.50 0.84 1.0000
7 22 5 154 282
250 1.43 2.64 1.0000
9 22 5 33 315
76 0.35 3.02 1.0000
11 22 5 207 340
212 -1.34 -1.52 1.0000
10 -23 -5 502 318
451 1.49 1.13 1.0000
8 -23 -5 94 290
195 -0.87 -5.27 1.0000
6 -23 -5 112 284
90 -0.76 0.64 1.0000
4 -23 -5 561 294
316 1.94 4.96 1.0000
2 -23 -5 12 303
-48 -1.27 -2.78 1.0000
0 -23 -5 29 328
-93 -0.81 4.61 1.0000
-2 -23 -5 215 341
279 0.72 -1.74 1.0000
-4 -23 -5 338 363
480 1.92 2.50 1.0000
8 23 5 127 300
168 3.89 -0.81 1.0000
10 23 5 229 331
114 -3.08 -2.37 1.0000
7 -24 -5 314 327

494 2.66 3.72 1.0000
5 -24 -5 20 293
-88 -3.08 -2.39 1.0000
3 -24 -5 202 325
227 0.50 1.15 1.0000
1 -24 -5 12 317
-7 2.32 -1.99 1.0000
-1 -24 -5 115 350
260 -2.63 -3.95 1.0000
-3 -24 -5 142 358
83 3.51 2.30 1.0000
-5 -24 -5 132 397
250 -1.10 -3.61 1.0000
4 -25 -5 128 333
131 -1.37 -1.14 1.0000
2 -25 -5 318 336
288 0.34 1.16 1.0000
0 -25 -5 5 337
-16 -0.78 1.76 1.0000
-2 -25 -5 414 378
447 0.81 -0.50 1.0000
-4 -25 -5 4 384
-95 -1.77 0.15 1.0000
4 24 -6 247 305
220 -0.88 -0.48 1.0000
-4 -24 6 1 344
-523 -0.88 0.48 1.0000
2 24 -6 215 315
326 0.63 -2.95 1.0000
-2 -24 6 12 341
-198 0.63 2.95 1.0000
0 24 -6 210 289
327 -3.08 1.59 1.0000
0 -24 6 172 341
279 -3.08 -1.59 1.0000
-2 24 -6 167 298
131 1.19 0.77 1.0000
2 -24 6 3 313
-149 1.19 -0.77 1.0000
-4 24 -6 49 308
78 -1.21 1.08 1.0000
4 -24 6 4 299
-248 -1.21 -1.08 1.0000
7 23 -6 410 309

```
552      3.01    -0.52  1.0000
  -7 -23    6       416       382
560      3.01     0.52  1.0000
   5  23   -6       159       286
223    -0.66     1.91  1.0000
  -5 -23    6        90       347
139    -0.66    -1.91  1.0000
   3  23   -6       571       292
856      5.22     0.02  1.0000
  -3 -23    6       205       360
385      5.22    -0.02  1.0000
   1  23   -6       151       266
238    -0.20     2.79  1.0000
  -1 -23    6        10       308
-43    -0.20    -2.79  1.0000
  -1  23   -6        13       279
14      0.30    -2.32  1.0000
   1 -23    6       261       316
289      0.30     2.32  1.0000
  -3  23   -6       248       291
159      2.44    -1.88  1.0000
   3 -23    6       672       323
521      2.44     1.88  1.0000
  -5  23   -6       161       302
181      0.41     1.13  1.0000
   5 -23    6        77       309
90      0.41    -1.13  1.0000
   7 -23    6        61       302
70    -2.43    -0.10  1.0000
  10  22   -6       343       371
623    -5.14    -2.21  1.0000
   8  22   -6       394       312
366    -0.12    -0.70  1.0000
  -8 -22    6       249       390
227    -0.12     0.70  1.0000
   6  22   -6       256       285
266      0.16    -0.22  1.0000
  -6 -22    6       704       346
720      0.16     0.22  1.0000
   4  22   -6       129       248
191    -2.47    -1.02  1.0000
  -4 -22    6        10       319
-101    -2.47     1.02  1.0000
   2  22   -6        10       230
```

60 -3.74 -1.36 1.0000
-2 -22 6 111 311
228 -3.74 1.36 1.0000
0 22 -6 19 238
3 2.41 -1.11 1.0000
0 -22 6 9 288
-209 2.41 1.11 1.0000
-2 22 -6 486 283
578 -5.32 1.64 1.0000
2 -22 6 526 306
625 -5.32 -1.64 1.0000
-4 22 -6 127 262
173 -1.44 0.97 1.0000
4 -22 6 4 287
-184 -1.44 -0.97 1.0000
-6 22 -6 200 278
221 2.95 5.04 1.0000
6 -22 6 45 267
-158 2.95 -5.04 1.0000
8 -22 6 440 292
603 -2.53 2.14 1.0000
10 -22 6 34 289
6 4.26 -1.39 1.0000
11 21 -6 707 365
459 3.95 2.11 1.0000
11 21 -6 27 292
-112 3.95 2.11 1.0000
9 21 -6 272 310
375 3.28 0.75 1.0000
7 21 -6 121 267
129 1.05 1.49 1.0000
-7 -21 6 69 351
74 1.05 -1.49 1.0000
5 21 -6 146 235
217 5.56 1.46 1.0000
-5 -21 6 44 320
-64 5.56 -1.46 1.0000
3 21 -6 363 234
257 -2.45 0.98 1.0000
-3 -21 6 269 301
179 -2.45 -0.98 1.0000
1 21 -6 586 257
859 4.46 -1.05 1.0000
-1 -21 6 528 326

789	4.46	1.05	1.0000	
-1	21	-6	552	244
594	2.96	1.53	1.0000	
1	-21	6	380	291
412	2.96	-1.53	1.0000	
-3	21	-6	183	235
160	-0.21	-1.09	1.0000	
3	-21	6	266	287
238	-0.21	1.09	1.0000	
-5	21	-6	681	276
740	-0.13	-1.73	1.0000	
5	-21	6	318	275
358	-0.13	1.73	1.0000	
-7	21	-6	358	282
368	-0.90	-1.18	1.0000	
7	-21	6	521	292
534	-0.90	1.18	1.0000	
9	-21	6	218	282
373	-3.08	-3.02	1.0000	
11	-21	6	213	304
409	-4.37	2.27	1.0000	
14	20	-6	294	426
434	3.19	-1.04	1.0000	
14	20	-6	15	321
-104	3.19	-1.04	1.0000	
12	20	-6	140	315
368	-6.87	1.96	1.0000	
12	20	-6	49	346
199	-6.87	1.96	1.0000	
10	20	-6	12	267
-69	2.72	-1.28	1.0000	
10	20	-6	244	297
360	2.72	-1.28	1.0000	
8	20	-6	71	262
171	-4.62	1.53	1.0000	
6	20	-6	29	225
-258	-4.27	-1.92	1.0000	
-6	-20	6	29	318
-22	-4.27	1.92	1.0000	
4	20	-6	271	237
277	2.75	-1.34	1.0000	
-4	-20	6	440	300
452	2.75	1.34	1.0000	
2	20	-6	1060	254

1072 -1.13 2.33 1.0000
-2 -20 6 1146 333
1156 -1.13 -2.33 1.0000
0 20 -6 538 206
341 -4.68 -3.20 1.0000
0 -20 6 482 275
297 -4.68 3.20 1.0000
-2 20 -6 338 223
302 -0.13 -0.87 1.0000
2 -20 6 384 269
345 -0.13 0.87 1.0000
-4 20 -6 382 238
543 -0.92 5.18 1.0000
4 -20 6 72 251
130 -0.92 -5.18 1.0000
-6 20 -6 2093 305
1747 -2.30 -3.24 1.0000
6 -20 6 1222 279
962 -2.30 3.24 1.0000
-8 20 -6 107 257
355 6.26 5.22 1.0000
8 -20 6 24 264
166 6.26 -5.22 1.0000
10 -20 6 1 252
2 0.71 -0.53 1.0000
12 -20 6 199 279
265 0.58 1.99 1.0000
14 -20 6 284 291
143 4.24 0.57 1.0000
15 19 -6 8 318
-9 -1.69 -1.88 1.0000
15 19 -6 1263 447
1414 -1.69 -1.88 1.0000
13 19 -6 214 316
315 3.11 5.91 1.0000
13 19 -6 438 365
599 3.11 5.91 1.0000
11 19 -6 145 291
293 2.24 -4.19 1.0000
11 19 -6 655 335
945 2.24 -4.19 1.0000
9 19 -6 183 261
254 0.80 2.57 1.0000
9 19 -6 10 250

-66 0.80 2.57 1.0000
7 19 -6 156 231
74 3.85 0.49 1.0000
5 19 -6 445 230
421 0.49 -0.01 1.0000
-5 -19 6 377 299
355 0.49 0.01 1.0000
3 19 -6 377 217
395 0.56 0.52 1.0000
-3 -19 6 460 291
480 0.56 -0.52 1.0000
1 19 -6 484 201
461 -0.96 0.12 1.0000
-1 -19 6 330 279
311 -0.96 -0.12 1.0000
-1 19 -6 102 179
107 2.47 -1.20 1.0000
1 -19 6 524 280
546 2.47 1.20 1.0000
-3 19 -6 548 209
401 2.52 2.01 1.0000
3 -19 6 14 237
-119 2.52 -2.01 1.0000
-5 19 -6 784 236
616 -0.92 -2.62 1.0000
5 -19 6 368 241
256 -0.92 2.62 1.0000
-7 19 -6 39 220
-3 -4.38 3.15 1.0000
7 -19 6 10 241
21 -4.38 -3.15 1.0000
-9 19 -6 40 221
-184 -4.92 -2.44 1.0000
9 -19 6 40 227
-54 -4.92 2.44 1.0000
11 -19 6 340 253
299 -2.39 -1.43 1.0000
13 -19 6 199 277
220 -1.91 0.78 1.0000
15 -19 6 180 288
195 -3.25 0.34 1.0000
16 18 -6 96 306
144 2.22 0.79 1.0000
16 18 -6 855 430

994	2.22	0.79	1.0000		
14	18	-6	303	349	
350	-0.99	2.05	1.0000		
14	18	-6	7	277	
-317	-0.99	2.05	1.0000		
12	18	-6	260	295	
266	-3.95	-3.25	1.0000		
12	18	-6	525	310	
548	-3.95	-3.25	1.0000		
10	18	-6	177	262	
116	-3.40	2.35	1.0000		
10	18	-6	23	260	
-72	-3.40	2.35	1.0000		
8	18	-6	172	243	
130	1.52	-2.05	1.0000		
8	18	-6	261	230	
210	1.52	-2.05	1.0000		
6	18	-6	707	241	
598	1.48	2.69	1.0000		
-6	-18	6	665	303	
562	1.48	-2.69	1.0000		
4	18	-6	103	186	
150	-2.61	-0.70	1.0000		
-4	-18	6	173	269	
234	-2.61	0.70	1.0000		
2	18	-6	165	212	
399	7.10	-0.42	1.0000		
-2	-18	6	59	264	
211	7.10	0.42	1.0000		
0	18	-6	1016	235	
1047	-1.34	1.53	1.0000		
0	-18	6	793	285	
823	-1.34	-1.53	1.0000		
-2	18	-6	825	213	
894	-0.84	-0.66	1.0000		
2	-18	6	698	270	
761	-0.84	0.66	1.0000		
-4	18	-6	401	215	
638	-0.07	-4.93	1.0000		
4	-18	6	28	248	
105	-0.07	4.93	1.0000		
-6	18	-6	1129	269	
1598	0.84	6.54	1.0000		
6	-18	6	1108	297	

1572 0.84 -6.54 1.0000
-8 18 -6 27 214
68 3.02 -2.53 1.0000
8 -18 6 21 229
-26 3.02 2.53 1.0000
10 -18 6 201 253
322 3.32 0.41 1.0000
12 -18 6 741 274
856 2.72 -2.32 1.0000
14 -18 6 215 271
234 -0.78 1.38 1.0000
16 -18 6 15 286
2 3.21 2.06 1.0000
17 17 -6 1811 441
1709 -1.32 -0.29 1.0000
17 17 -6 719 332
655 -1.32 -0.29 1.0000
15 17 -6 365 361
546 -0.93 3.61 1.0000
15 17 -6 18 271
-354 -0.93 3.61 1.0000
13 17 -6 129 277
270 2.09 -4.65 1.0000
13 17 -6 288 305
489 2.09 -4.65 1.0000
11 17 -6 919 292
1382 2.37 6.75 1.0000
11 17 -6 807 313
1242 2.37 6.75 1.0000
9 17 -6 272 249
358 1.42 -2.73 1.0000
9 17 -6 292 238
381 1.42 -2.73 1.0000
7 17 -6 101 204
97 0.32 2.04 1.0000
5 17 -6 10 198
87 -8.96 1.29 1.0000
-5 -17 6 109 282
-36 -8.96 -1.29 1.0000
3 17 -6 31 196
57 -0.16 -1.89 1.0000
-3 -17 6 5 255
-71 -0.16 1.89 1.0000
1 17 -6 3222 338

3368	-0.80	-0.83	1.0000	
-1	-17	6	3085	404
3229	-0.80	0.83	1.0000	
-1	17	-6	1249	291
1644	-4.65	1.88	1.0000	
1	-17	6	989	285
1347	-4.65	-1.88	1.0000	
-3	17	-6	333	190
216	2.91	-2.40	1.0000	
3	-17	6	377	243
251	2.91	2.40	1.0000	
-5	17	-6	136	199
250	-0.85	4.69	1.0000	
5	-17	6	189	230
323	-0.85	-4.69	1.0000	
-7	17	-6	904	240
1125	-1.60	-3.16	1.0000	
7	-17	6	716	268
911	-1.60	3.16	1.0000	
-9	17	-6	849	242
1077	-4.11	2.62	1.0000	
9	-17	6	497	250
670	-4.11	-2.62	1.0000	
11	-17	6	200	235
114	-4.27	-1.36	1.0000	
13	-17	6	34	230
-136	-4.61	2.12	1.0000	
15	-17	6	7	258
7	0.27	0.99	1.0000	
17	-17	6	218	285
182	-1.36	-0.29	1.0000	
18	16	-6	182	343
233	3.18	2.05	1.0000	
18	16	-6	1434	472
1597	3.18	2.05	1.0000	
16	16	-6	1188	411
1131	-0.43	0.72	1.0000	
16	16	-6	179	289
157	-0.43	0.72	1.0000	
14	16	-6	78	269
180	-4.37	-0.84	1.0000	
14	16	-6	438	335
657	-4.37	-0.84	1.0000	
12	16	-6	135	252

240 4.45 1.39 1.0000
12 16 -6 209 275
339 4.45 1.39 1.0000
10 16 -6 34 238
-197 -4.96 -0.76 1.0000
10 16 -6 394 237
228 -4.96 -0.76 1.0000
8 16 -6 34 188
68 -2.05 0.50 1.0000
8 16 -6 6 218
-148 -2.05 0.50 1.0000
6 16 -6 3 183
99 7.40 -4.37 1.0000
4 16 -6 401 279
1354 12.04 8.16 1.0000
-4 -16 6 431 310
1409 12.04 -8.16 1.0000
2 16 -6 482 193
422 -1.84 -4.64 1.0000
-2 -16 6 729 271
666 -1.84 4.64 1.0000
0 16 -6 981 189
546 9.61 1.89 1.0000
0 -16 6 706 237
340 9.61 -1.89 1.0000
-2 16 -6 82 173
66 1.82 -0.35 1.0000
2 -16 6 5 209
-147 1.82 0.35 1.0000
-4 16 -6 794 134
698 -2.79 -0.98 1.0000
-4 16 -6 676 198
587 -2.79 -0.98 1.0000
4 -16 6 364 231
297 -2.79 0.98 1.0000
-6 16 -6 334 122
112 5.83 -3.35 1.0000
-6 16 -6 183 187
34 5.83 -3.35 1.0000
6 -16 6 188 213
36 5.83 3.35 1.0000
-8 16 -6 2703 326
3176 -3.85 1.16 1.0000
8 -16 6 2388 352

2835 -3.85 -1.16 1.0000
-10 16 -6 686 209
578 3.08 0.46 1.0000
10 -16 6 437 233
350 3.08 -0.46 1.0000
12 -16 6 83 227
205 4.73 2.95 1.0000
14 -16 6 116 257
142 -1.41 -1.04 1.0000
16 -16 6 1 253
-142 -0.38 0.67 1.0000
18 -16 6 152 284
211 2.21 0.75 1.0000
19 15 -6 1344 465
1404 -3.18 0.33 1.0000
19 15 -6 628 355
665 -3.18 0.33 1.0000
17 15 -6 1161 408
1184 0.27 1.02 1.0000
17 15 -6 129 316
136 0.27 1.02 1.0000
15 15 -6 7 329
13 0.07 0.91 1.0000
15 15 -6 194 279
223 0.07 0.91 1.0000
13 15 -6 168 244
297 -3.52 2.44 1.0000
13 15 -6 352 278
532 -3.52 2.44 1.0000
11 15 -6 549 231
408 -1.23 -2.59 1.0000
11 15 -6 129 233
66 -1.23 -2.59 1.0000
9 15 -6 239 216
351 2.21 4.79 1.0000
9 15 -6 476 240
640 2.21 4.79 1.0000
7 15 -6 8 197
158 -9.77 -0.08 1.0000
7 15 -6 10 200
168 -9.77 -0.08 1.0000
5 15 -6 721 209
644 -1.88 1.19 1.0000
3 15 -6 1351 188

595 -13.56 -0.54 1.0000

-3 -15 6 1193 243

479 -13.56 0.54 1.0000

1 15 -6 852 223

1296 -5.81 2.69 1.0000

-1 -15 6 882 289

1334 -5.81 -2.69 1.0000

-1 15 -6 403 195

626 6.59 -3.94 1.0000

1 -15 6 463 235

703 6.59 3.94 1.0000

-3 15 -6 2719 303

2580 -4.04 3.34 1.0000

-3 15 -6 2510 183

2375 -4.04 3.34 1.0000

3 -15 6 2849 326

2704 -4.04 -3.34 1.0000

-5 15 -6 949 223

1151 4.00 -1.62 1.0000

-5 15 -6 868 152

1061 4.00 -1.62 1.0000

5 -15 6 753 248

933 4.00 1.62 1.0000

-7 15 -6 915 152

657 0.83 4.02 1.0000

-7 15 -6 664 197

447 0.83 4.02 1.0000

7 -15 6 589 213

386 0.83 -4.02 1.0000

-9 15 -6 237 188

221 0.18 -1.56 1.0000

-9 15 -6 337 148

318 0.18 -1.56 1.0000

9 -15 6 111 207

98 0.18 1.56 1.0000

-11 15 -6 10 155

60 -5.14 0.50 1.0000

-11 15 -6 68 182

180 -5.14 0.50 1.0000

11 -15 6 36 201

-93 -5.14 -0.50 1.0000

13 -15 6 21 209

8 -1.98 -2.01 1.0000

15 -15 6 1 250

-75 0.12 0.84 1.0000
17 -15 6 52 255
130 -0.90 3.57 1.0000
19 -15 6 217 299
286 -1.80 -1.98 1.0000
18 14 -6 1700 422
1758 -0.78 1.44 1.0000
18 14 -6 157 313
173 -0.78 1.44 1.0000
16 14 -6 822 358
894 -1.43 -1.11 1.0000
16 14 -6 334 307
379 -1.43 -1.11 1.0000
14 14 -6 492 276
321 4.43 0.31 1.0000
14 14 -6 543 268
363 4.43 0.31 1.0000
12 14 -6 24 224
125 -5.09 1.88 1.0000
12 14 -6 296 244
550 -5.09 1.88 1.0000
10 14 -6 29 196
105 4.69 -4.11 1.0000
10 14 -6 165 221
344 4.69 -4.11 1.0000
8 14 -6 194 218
81 4.73 -0.59 1.0000
8 14 -6 153 190
55 4.73 -0.59 1.0000
6 14 -6 161 193
354 5.48 6.71 1.0000
6 14 -6 420 227
744 5.48 6.71 1.0000
4 14 -6 2054 332
3589 11.19 -9.95 1.0000
-4 -14 6 2137 368
3713 11.19 9.95 1.0000
2 14 -6 15 173
396 15.88 11.30 1.0000
-2 -14 6 40 227
752 15.88 -11.30 1.0000
0 14 -6 393 172
433 -0.15 -4.81 1.0000
0 -14 6 667 200

```
729    -0.15     4.81  1.0000
  -2  14  -6     1329      259
1406    -0.06    -2.94  1.0000
  -2  14  -6     1581      150
1666    -0.06    -2.94  1.0000
   2 -14   6     1482      247
1567    -0.06     2.94  1.0000
  -4  14  -6     2158      266
1714     1.42     4.64  1.0000
  -4  14  -6     2756      170
2252     1.42     4.64  1.0000
   4 -14   6     1987      285
1562     1.42    -4.64  1.0000
  -6  14  -6      687      146
1076   -10.76    -7.73  1.0000
  -6  14  -6      630      196
995   -10.76    -7.73  1.0000
   6 -14   6      348      222
567   -10.76     7.73  1.0000
  -8  14  -6      531      183
355      3.41     1.37  1.0000
  -8  14  -6      351      136
211      3.41     1.37  1.0000
   8 -14   6       66      184
15      3.41    -1.37  1.0000
 -10  14  -6       87      144
99      0.07    -1.39  1.0000
 -10  14  -6      152      173
169      0.07    -1.39  1.0000
  10 -14   6      215      207
235      0.07     1.39  1.0000
 -12  14  -6      293      159
320     -3.01    -1.42  1.0000
  12 -14   6      365      220
397     -3.01     1.42  1.0000
  14 -14   6      293      220
163      4.44     0.31  1.0000
  16 -14   6      220      245
383     -4.44    -0.82  1.0000
  18 -14   6      178      265
216     -0.96     2.11  1.0000
  20 -14   6       16      300
-41      3.30    -1.21  1.0000
  19  13  -6     1767      434
```

1836	-1.89	0.82	1.0000	
19	13	-6	6	337
-33	-1.89	0.82	1.0000	
17	13	-6	222	357
365	-4.67	2.13	1.0000	
17	13	-6	35	295
-226	-4.67	2.13	1.0000	
15	13	-6	11	245
-157	-1.98	-2.11	1.0000	
15	13	-6	246	286
221	-1.98	-2.11	1.0000	
13	13	-6	422	259
423	0.19	1.13	1.0000	
13	13	-6	358	243
359	0.19	1.13	1.0000	
11	13	-6	653	235
635	-2.14	2.16	1.0000	
11	13	-6	634	220
616	-2.14	2.16	1.0000	
9	13	-6	1137	264
1657	-6.07	0.02	1.0000	
9	13	-6	662	242
1070	-6.07	0.02	1.0000	
7	13	-6	1584	285
1775	-3.04	6.68	1.0000	
7	13	-6	1994	292
2215	-3.04	6.68	1.0000	
5	13	-6	166	172
423	-15.17	-5.02	1.0000	
3	13	-6	9	161
97	-5.88	6.86	1.0000	
-3	-13	6	219	212
600	-5.88	-6.86	1.0000	
1	13	-6	1428	206
1317	-3.86	-4.77	1.0000	
-1	-13	6	1855	269
1735	-3.86	4.77	1.0000	
-1	13	-6	502	98
240	-4.97	4.56	1.0000	
-1	13	-6	248	144
75	-4.97	4.56	1.0000	
1	-13	6	545	174
269	-4.97	-4.56	1.0000	
-3	13	-6	209	131

995 16.50 -1.66 1.0000
-3 13 -6 157 194
865 16.50 -1.66 1.0000
3 -13 6 142 188
799 16.50 1.66 1.0000
-5 13 -6 534 129
594 8.53 1.17 1.0000
-5 13 -6 620 181
693 8.53 1.17 1.0000
5 -13 6 432 186
473 8.53 -1.17 1.0000
-7 13 -6 1671 266
2188 7.22 -0.82 1.0000
-7 13 -6 1963 194
2523 7.22 -0.82 1.0000
7 -13 6 1952 290
2512 7.22 0.82 1.0000
-9 13 -6 229 166
355 1.21 3.17 1.0000
-9 13 -6 206 136
326 1.21 3.17 1.0000
9 -13 6 4 182
29 1.21 -3.17 1.0000
-11 13 -6 86 142
98 -0.55 -0.13 1.0000
-11 13 -6 31 156
39 -0.55 -0.13 1.0000
11 -13 6 27 194
34 -0.55 0.13 1.0000
-13 13 -6 335 154
335 0.23 -1.15 1.0000
13 -13 6 466 226
465 0.23 1.15 1.0000
-15 13 -6 28 154
89 -3.59 -2.41 1.0000
15 -13 6 288 225
455 -3.59 2.41 1.0000
17 -13 6 88 249
209 2.21 -4.62 1.0000
19 -13 6 64 271
91 3.12 5.87 1.0000
20 12 -6 1448 458
1620 0.60 1.98 1.0000
18 12 -6 881 335

1002 2.68 -2.41 1.0000
16 12 -6 41 272
-117 4.67 2.97 1.0000
16 12 -6 212 314
395 4.67 2.97 1.0000
14 12 -6 246 249
274 -3.06 1.55 1.0000
14 12 -6 608 275
660 -3.06 1.55 1.0000
12 12 -6 30 227
7 2.36 -3.87 1.0000
12 12 -6 205 227
198 2.36 -3.87 1.0000
10 12 -6 36 199
-7 -1.92 4.82 1.0000
10 12 -6 36 212
-89 -1.92 4.82 1.0000
8 12 -6 2552 309
2370 2.76 -2.28 1.0000
8 12 -6 2327 285
2153 2.76 -2.28 1.0000
6 12 -6 1122 172
295 13.27 -5.99 1.0000
6 12 -6 1674 194
609 13.27 -5.99 1.0000
4 12 -6 381 158
203 5.82 14.13 1.0000
2 12 -6 2118 190
1048 7.95 -12.41 1.0000
-2 -12 6 2724 226
1494 7.95 12.41 1.0000
0 12 -6 670 150
238 8.85 7.54 1.0000
0 -12 6 899 168
403 8.85 -7.54 1.0000
-2 12 -6 1582 235
1840 -9.46 1.90 1.0000
-2 12 -6 1259 146
1477 -9.46 1.90 1.0000
2 -12 6 1536 225
1791 -9.46 -1.90 1.0000
-4 12 -6 214 238
1364 -14.26 -13.09 1.0000
-4 12 -6 322 150

1620 -14.26 -13.09 1.0000
4 -12 6 293 227
1551 -14.26 13.09 1.0000
-6 12 -6 273 185
894 -8.01 10.08 1.0000
-6 12 -6 399 149
1120 -8.01 10.08 1.0000
6 -12 6 204 176
759 -8.01 -10.08 1.0000
-8 12 -6 604 128
89 -10.92 -8.86 1.0000
-8 12 -6 663 156
114 -10.92 -8.86 1.0000
8 -12 6 953 177
273 -10.92 8.86 1.0000
-10 12 -6 2638 267
2284 -2.12 -2.89 1.0000
-10 12 -6 2501 200
2156 -2.12 -2.89 1.0000
10 -12 6 2190 279
1871 -2.12 2.89 1.0000
-12 12 -6 358 160
363 2.45 4.06 1.0000
-12 12 -6 293 141
295 2.45 4.06 1.0000
12 -12 6 161 198
152 2.45 -4.06 1.0000
-14 12 -6 190 156
400 -5.04 -1.92 1.0000
14 -12 6 172 216
373 -5.04 1.92 1.0000
-16 12 -6 215 174
344 4.43 -1.43 1.0000
16 -12 6 117 232
214 4.43 1.43 1.0000
18 -12 6 139 248
133 -4.07 -3.30 1.0000
20 -12 6 353 313
688 -6.84 1.91 1.0000
19 11 -6 30 297
13 -2.53 -1.45 1.0000
19 11 -6 1059 385
995 -2.53 -1.45 1.0000
17 11 -6 741 327

588	-4.26	-1.23	1.0000	
17	11	-6	290	257
191	-4.26	-1.23	1.0000	
15	11	-6	104	254
238	-5.10	-0.62	1.0000	
15	11	-6	35	248
-58	-5.10	-0.62	1.0000	
13	11	-6	1	210
-223	-0.61	-0.12	1.0000	
13	11	-6	66	232
78	-0.61	-0.12	1.0000	
11	11	-6	349	230
424	1.55	-0.54	1.0000	
11	11	-6	231	224
292	1.55	-0.54	1.0000	
9	11	-6	647	181
577	-0.32	3.14	1.0000	
9	11	-6	430	194
371	-0.32	3.14	1.0000	
7	11	-6	717	210
1027	-3.75	-3.03	1.0000	
7	11	-6	1037	216
1405	-3.75	-3.03	1.0000	
5	11	-6	545	152
247	-5.22	12.11	1.0000	
5	11	-6	231	156
-78	-5.22	12.11	1.0000	
3	11	-6	127	175
562	-5.92	-9.04	1.0000	
-3	-11	6	249	198
795	-5.92	9.04	1.0000	
1	11	-6	2467	245
2087	-2.80	6.25	1.0000	
1	11	-6	2530	150
2145	-2.80	6.25	1.0000	
-1	-11	6	2621	251
2240	-2.80	-6.25	1.0000	
-1	11	-6	40	152
420	11.94	-3.84	1.0000	
-1	11	-6	96	105
579	11.94	-3.84	1.0000	
1	-11	6	146	172
691	11.94	3.84	1.0000	
-3	11	-6	534	111

599 13.22 3.58 1.0000
-3 11 -6 315 151
281 13.22 3.58 1.0000
3 -11 6 458 149
492 13.22 -3.58 1.0000
-5 11 -6 228 107
315 18.64 -1.08 1.0000
-5 11 -6 250 133
107 18.64 -1.08 1.0000
5 -11 6 254 149
426 18.64 1.08 1.0000
-7 11 -6 120 143
974 17.57 0.79 1.0000
-7 11 -6 147 202
1050 17.57 0.79 1.0000
7 -11 6 139 187
1029 17.57 -0.79 1.0000
-9 11 -6 1325 185
1773 5.04 0.07 1.0000
-9 11 -6 1448 226
1914 5.04 0.07 1.0000
9 -11 6 1373 259
1830 5.04 -0.07 1.0000
-11 11 -6 914 164
1036 1.60 0.56 1.0000
-11 11 -6 863 168
982 1.60 0.56 1.0000
11 -11 6 561 210
657 1.60 -0.56 1.0000
-13 11 -6 666 164
645 -2.17 -2.28 1.0000
13 -11 6 733 212
711 -2.17 2.28 1.0000
-15 11 -6 267 142
176 -1.13 2.48 1.0000
15 -11 6 376 226
266 -1.13 -2.48 1.0000
-17 11 -6 657 198
1044 2.39 -6.62 1.0000
17 -11 6 724 282
1133 2.39 6.62 1.0000
19 -11 6 603 301
874 2.21 -4.07 1.0000
21 -11 6 620 302

393	3.89	2.07	1.0000	
20	10	-6	1	344
-114	0.78	-0.44	1.0000	
20	10	-6	780	364
824	0.78	-0.44	1.0000	
18	10	-6	768	266
989	3.28	0.36	1.0000	
18	10	-6	418	318
584	3.28	0.36	1.0000	
16	10	-6	550	255
457	2.98	-0.48	1.0000	
16	10	-6	537	233
445	2.98	-0.48	1.0000	
14	10	-6	2	223
-167	-0.06	1.35	1.0000	
14	10	-6	103	212
119	-0.06	1.35	1.0000	
12	10	-6	2171	282
1843	-2.15	3.09	1.0000	
12	10	-6	1472	247
1203	-2.15	3.09	1.0000	
10	10	-6	2596	284
2148	-4.14	0.04	1.0000	
10	10	-6	2405	287
1974	-4.14	0.04	1.0000	
8	10	-6	8	158
-259	-0.52	2.40	1.0000	
8	10	-6	56	146
39	-0.52	2.40	1.0000	
6	10	-6	1126	254
1244	-4.70	3.31	1.0000	
6	10	-6	1365	234
1498	-4.70	3.31	1.0000	
4	10	-6	2805	291
3355	9.00	-10.88	1.0000	
2	10	-6	169	156
437	2.52	16.20	1.0000	
2	10	-6	235	101
627	2.52	16.20	1.0000	
-2	-10	6	281	173
753	2.52	-16.20	1.0000	
0	10	-6	530	205
1244	-10.29	-7.12	1.0000	
0	10	-6	661	127

1447	-10.29	-7.12	1.0000	
0	-10	6	629	200
1406	-10.29	7.12	1.0000	
-2	10	-6	506	122
189	-7.00	-3.67	1.0000	
-2	10	-6	527	94
202	-7.00	-3.67	1.0000	
2	-10	6	423	127
139	-7.00	3.67	1.0000	
-4	10	-6	874	117
-75	-24.03	8.92	1.0000	
-4	10	-6	1034	94
60	-24.03	8.92	1.0000	
4	-10	6	874	117
-14	-24.03	-8.92	1.0000	
-6	10	-6	410	229
2229	-19.58	-13.07	1.0000	
-6	10	-6	308	174
1979	-19.58	-13.07	1.0000	
6	-10	6	383	220
2158	-19.58	13.07	1.0000	
-8	10	-6	82	121
465	-10.61	2.37	1.0000	
-8	10	-6	118	146
548	-10.61	2.37	1.0000	
8	-10	6	51	154
387	-10.61	-2.37	1.0000	
-10	10	-6	2450	194
2023	-3.98	0.19	1.0000	
-10	10	-6	2500	225
2069	-3.98	0.19	1.0000	
10	-10	6	2865	273
2403	-3.98	-0.19	1.0000	
-12	10	-6	294	134
247	-1.97	-4.93	1.0000	
-12	10	-6	330	132
283	-1.97	-4.93	1.0000	
12	-10	6	40	170
2	-1.97	4.93	1.0000	
-14	10	-6	58	139
169	4.68	4.20	1.0000	
14	-10	6	34	203
119	4.68	-4.20	1.0000	
-16	10	-6	383	149

222	-5.06	0.78	1.0000	
16	-10	6	35	212
-98	-5.06	-0.78	1.0000	
-18	10	-6	130	149
78	-3.30	-2.42	1.0000	
18	-10	6	29	236
2	-3.30	2.42	1.0000	
20	-10	6	63	267
128	2.77	-1.25	1.0000	
22	-10	6	45	305
173	-5.16	-2.24	1.0000	
21	9	-6	25	358
-89	-3.09	-3.01	1.0000	
21	9	-6	872	318
1165	-3.09	-3.01	1.0000	
19	9	-6	92	325
37	-4.98	2.42	1.0000	
19	9	-6	440	257
356	-4.98	2.42	1.0000	
17	9	-6	898	324
1143	-4.13	-2.75	1.0000	
17	9	-6	1015	271
1275	-4.13	-2.75	1.0000	
15	9	-6	180	225
170	0.00	1.67	1.0000	
15	9	-6	4	256
-50	0.00	1.67	1.0000	
13	9	-6	16	219
-199	1.21	-3.25	1.0000	
13	9	-6	16	202
-54	1.21	-3.25	1.0000	
11	9	-6	1181	316
1604	4.96	-0.35	1.0000	
11	9	-6	1744	285
2251	4.96	-0.35	1.0000	
9	9	-6	77	198
420	10.20	-0.29	1.0000	
7	9	-6	2889	259
3374	1.31	3.53	1.0000	
7	9	-6	2334	294
2772	1.31	3.53	1.0000	
5	9	-6	2890	255
3375	6.71	-4.63	1.0000	
5	9	-6	2526	273

2976 6.71 -4.63 1.0000
3 9 -6 321 95
512 -1.18 9.15 1.0000
3 9 -6 167 141
278 -1.18 9.15 1.0000
-3 -9 6 372 160
580 -1.18 -9.15 1.0000
1 9 -6 607 208
1334 1.97 -10.11 1.0000
1 9 -6 729 128
1511 1.97 -10.11 1.0000
-1 -9 6 862 204
1700 1.97 10.11 1.0000
-1 9 -6 301 190
1007 16.02 8.23 1.0000
-1 9 -6 480 124
1379 16.02 8.23 1.0000
1 -9 6 455 178
1334 16.02 -8.23 1.0000
-3 9 -6 1536 99
405 19.07 -7.29 1.0000
-3 9 -6 1207 129
224 19.07 -7.29 1.0000
3 -9 6 1270 115
257 19.07 7.29 1.0000
-5 9 -6 2769 144
503 29.22 2.96 1.0000
-5 9 -6 3067 114
649 29.22 2.96 1.0000
5 -9 6 2893 136
579 29.22 -2.96 1.0000
-7 9 -6 610 129
209 11.37 0.64 1.0000
-7 9 -6 940 118
437 11.37 0.64 1.0000
7 -9 6 611 134
206 11.37 -0.64 1.0000
-9 9 -6 401 151
994 9.99 0.43 1.0000
-9 9 -6 379 155
960 9.99 0.43 1.0000
9 -9 6 324 168
872 9.99 -0.43 1.0000
-11 9 -6 734 139

651 -0.24 -3.42 1.0000
-11 9 -6 854 147
765 -0.24 -3.42 1.0000
11 -9 6 497 178
425 -0.24 3.42 1.0000
-13 9 -6 810 185
1244 -5.94 0.06 1.0000
13 -9 6 677 230
1079 -5.94 -0.06 1.0000
-15 9 -6 534 160
711 2.32 -4.71 1.0000
15 -9 6 235 224
350 2.32 4.71 1.0000
-17 9 -6 42 149
73 1.32 2.72 1.0000
17 -9 6 191 232
259 1.32 -2.72 1.0000
-19 9 -6 28 152
56 0.78 -2.56 1.0000
19 -9 6 9 241
-21 0.78 2.56 1.0000
21 -9 6 15 270
-93 3.24 0.69 1.0000
22 8 -6 745 318
955 -2.49 2.27 1.0000
22 8 -6 192 374
304 -2.49 2.27 1.0000
20 8 -6 116 286
371 6.15 -5.31 1.0000
20 8 -6 10 335
102 6.15 -5.31 1.0000
18 8 -6 21 287
56 2.99 2.50 1.0000
18 8 -6 39 258
89 2.99 2.50 1.0000
16 8 -6 2189 398
2618 -3.86 -1.16 1.0000
16 8 -6 2453 413
2907 -3.86 -1.16 1.0000
14 8 -6 17 247
-12 3.34 -1.30 1.0000
14 8 -6 17 233
-19 3.34 -1.30 1.0000
12 8 -6 534 174

66 -10.91 8.85 1.0000
12 8 -6 263 209
-146 -10.91 8.85 1.0000
10 8 -6 0 210
173 -10.81 -2.29 1.0000
10 8 -6 44 169
375 -10.81 -2.29 1.0000
8 8 -6 70 156
445 -11.14 2.32 1.0000
8 8 -6 41 197
364 -11.14 2.32 1.0000
6 8 -6 449 159
199 -8.96 4.11 1.0000
4 8 -6 219 150
291 -7.95 2.42 1.0000
4 8 -6 312 131
417 -7.95 2.42 1.0000
-4 -8 6 71 135
46 -7.95 -2.42 1.0000
2 8 -6 1537 140
1906 -1.49 -5.09 1.0000
2 8 -6 1298 214
1637 -1.49 -5.09 1.0000
-2 -8 6 1577 205
1950 -1.49 5.09 1.0000
-2 -8 6 1603 198
1979 -1.49 5.09 1.0000
0 8 -6 1776 191
1398 -2.36 6.91 1.0000
0 8 -6 1594 124
1234 -2.36 6.91 1.0000
0 -8 6 1694 188
1320 -2.36 -6.91 1.0000
0 -8 6 1674 169
1302 -2.36 -6.91 1.0000
-2 8 -6 337 133
376 -26.65 -0.23 1.0000
-2 8 -6 301 98
634 -26.65 -0.23 1.0000
2 -8 6 319 123
580 -26.65 0.23 1.0000
2 -8 6 319 140
599 -26.65 0.23 1.0000
-4 8 -6 1792 261

3871	-17.74	-1.40	1.0000	
-4	8	-6	1893	217
4019	-17.74	-1.40	1.0000	
4	-8	6	1753	244
3808	-17.74	1.40	1.0000	
-6	8	-6	2422	324
5798	-24.98	-1.78	1.0000	
-6	8	-6	2449	295
5841	-24.98	-1.78	1.0000	
6	-8	6	2465	325
5844	-24.98	1.78	1.0000	
-8	8	-6	73	130
449	-11.02	-2.20	1.0000	
-8	8	-6	138	129
597	-11.02	-2.20	1.0000	
8	-8	6	164	149
650	-11.02	2.20	1.0000	
-10	8	-6	103	115
84	-0.38	-2.45	1.0000	
-10	8	-6	8	112
-5	-0.38	-2.45	1.0000	
10	-8	6	74	124
53	-0.38	2.45	1.0000	
-12	8	-6	2974	220
2775	2.86	2.53	1.0000	
-12	8	-6	2970	218
2771	2.86	2.53	1.0000	
12	-8	6	3105	290
2908	2.86	-2.53	1.0000	
-14	8	-6	363	137
202	4.62	0.48	1.0000	
14	-8	6	213	192
95	4.62	-0.48	1.0000	
-16	8	-6	6	138
-41	-2.11	-0.61	1.0000	
16	-8	6	6	203
-2	-2.11	0.61	1.0000	
-18	8	-6	361	153
297	1.63	2.05	1.0000	
18	-8	6	9	218
-6	1.63	-2.05	1.0000	
-20	8	-6	39	161
117	-4.68	-1.57	1.0000	
20	-8	6	201	286

363 -4.68 1.57 1.0000
22 -8 6 1 291
-50 0.00 -0.68 1.0000
23 7 -6 946 337
999 -2.40 -0.16 1.0000
23 7 -6 8 391
-35 -2.40 -0.16 1.0000
21 7 -6 394 360
396 -1.01 1.02 1.0000
21 7 -6 398 320
400 -1.01 1.02 1.0000
19 7 -6 15 304
59 -4.36 -3.10 1.0000
19 7 -6 20 280
73 -4.36 -3.10 1.0000
17 7 -6 388 295
541 -1.79 3.08 1.0000
17 7 -6 585 277
770 -1.79 3.08 1.0000
15 7 -6 312 250
168 0.86 -4.07 1.0000
15 7 -6 1115 269
825 0.86 -4.07 1.0000
13 7 -6 1515 350
1988 7.01 0.76 1.0000
13 7 -6 1507 306
1979 7.01 0.76 1.0000
11 7 -6 166 207
1094 17.51 -0.74 1.0000
11 7 -6 16 230
583 17.51 -0.74 1.0000
9 7 -6 953 204
438 11.35 -0.72 1.0000
9 7 -6 827 150
349 11.35 -0.72 1.0000
7 7 -6 1143 241
3093 20.61 -0.37 1.0000
7 7 -6 1291 303
3340 20.61 -0.37 1.0000
5 7 -6 5203 322
6043 5.94 1.16 1.0000
5 7 -6 5542 379
6408 5.94 1.16 1.0000
-5 -7 6 5103 334

5929	5.94	-1.16	1.0000	
3	7	-6	2739	295
3496	2.86	-6.70	1.0000	
-3	-7	6	3517	250
4373	2.86	6.70	1.0000	
1	7	-6	4731	318
7016	9.58	9.34	1.0000	
1	7	-6	4614	382
6873	9.58	9.34	1.0000	
-1	-7	6	4857	352
7171	9.58	-9.34	1.0000	
-1	-7	6	4501	352
6737	9.58	-9.34	1.0000	
-1	7	-6	978	186
1091	0.95	-14.07	1.0000	
-1	7	-6	1088	122
1223	0.95	-14.07	1.0000	
1	-7	6	959	175
1115	0.95	14.07	1.0000	
1	-7	6	1057	146
1234	0.95	14.07	1.0000	
-3	7	-6	4540	186
1364	26.06	6.39	1.0000	
-3	7	-6	4637	126
1418	26.06	6.39	1.0000	
-3	7	-6	3615	178
878	26.06	6.39	1.0000	
3	-7	6	4449	149
1317	26.06	-6.39	1.0000	
-5	7	-6	1043	93
256	14.09	-1.19	1.0000	
-5	7	-6	959	118
215	14.09	-1.19	1.0000	
5	-7	6	1172	108
322	14.09	1.19	1.0000	
-7	7	-6	1316	239
3376	20.50	0.26	1.0000	
-7	7	-6	1293	211
3338	20.50	0.26	1.0000	
7	-7	6	1440	244
3572	20.50	-0.26	1.0000	
-9	7	-6	2801	223
3301	1.40	-3.67	1.0000	
-9	7	-6	2992	237

3508 1.40 -3.67 1.0000
9 -7 6 2801 258
3301 1.40 3.67 1.0000
-11 7 -6 989 165
1351 -3.72 3.24 1.0000
-11 7 -6 1093 170
1473 -3.72 3.24 1.0000
11 -7 6 1147 222
1538 -3.72 -3.24 1.0000
-13 7 -6 2046 209
2289 -3.09 -6.69 1.0000
13 -7 6 2098 285
2332 -3.09 6.69 1.0000
-15 7 -6 54 151
316 -9.61 0.07 1.0000
15 -7 6 46 208
294 -9.61 -0.07 1.0000
-17 7 -6 182 147
180 0.32 -2.06 1.0000
17 -7 6 228 213
225 0.32 2.06 1.0000
-19 7 -6 19 143
-104 3.71 -0.51 1.0000
19 -7 6 19 236
-95 3.71 0.51 1.0000
-21 7 -6 4 162
-23 1.07 -1.49 1.0000
21 -7 6 4 273
-71 1.07 1.49 1.0000
23 -7 6 15 290
49 3.02 -0.45 1.0000
22 6 -6 202 338
224 2.95 -5.06 1.0000
22 6 -6 41 339
5 2.95 -5.06 1.0000
20 6 -6 1074 336
832 -2.30 3.21 1.0000
20 6 -6 1590 332
1293 -2.30 3.21 1.0000
18 6 -6 799 332
1186 0.79 -6.41 1.0000
18 6 -6 754 314
1131 0.79 -6.41 1.0000
16 6 -6 342 268

118	5.76	3.35	1.0000	
16	6	-6	371	271
135	5.76	3.35	1.0000	
14	6	-6	476	269
769	-10.78	7.73	1.0000	
14	6	-6	475	239
768	-10.78	7.73	1.0000	
12	6	-6	113	238
560	-8.09	-10.10	1.0000	
12	6	-6	139	207
621	-8.09	-10.10	1.0000	
10	6	-6	415	248
2230	-19.49	13.14	1.0000	
10	6	-6	535	329
2498	-19.49	13.14	1.0000	
8	6	-6	1947	305
5024	-25.04	1.83	1.0000	
8	6	-6	2186	376
5411	-25.04	1.83	1.0000	
6	6	-6	692	256
1487	-10.82	-7.77	1.0000	
4	6	-6	3858	172
1928	-8.06	13.59	1.0000	
4	6	-6	3315	239
1550	-8.06	13.59	1.0000	
2	6	-6	5808	380
6289	-5.17	-5.22	1.0000	
-2	-6	6	5851	328
6327	-5.17	5.22	1.0000	
-2	-6	6	6672	324
7184	-5.17	5.22	1.0000	
0	6	-6	889	112
774	-5.67	-0.01	1.0000	
0	6	-6	710	149
604	-5.67	-0.01	1.0000	
0	-6	6	837	113
722	-5.67	0.01	1.0000	
0	-6	6	831	122
716	-5.67	0.01	1.0000	
-2	6	-6	338	116
903	-4.01	12.38	1.0000	
-2	6	-6	221	145
683	-4.01	12.38	1.0000	
2	-6	6	360	120

937 -4.01 -12.38 1.0000
-4 6 -6 1534 255
3642 -22.08 -14.57 1.0000
-4 6 -6 1574 207
3711 -22.08 -14.57 1.0000
-4 6 -6 1562 256
3690 -22.08 -14.57 1.0000
4 -6 6 1780 227
4065 -22.08 14.57 1.0000
-6 6 -6 502 134
1197 -10.68 7.98 1.0000
-6 6 -6 348 167
935 -10.68 7.98 1.0000
6 -6 6 459 152
1127 -10.68 -7.98 1.0000
-8 6 -6 501 111
230 -8.90 -4.02 1.0000
-8 6 -6 400 95
155 -8.90 -4.02 1.0000
8 -6 6 297 116
88 -8.90 4.02 1.0000
-10 6 -6 1446 174
1571 -4.75 -3.18 1.0000
-10 6 -6 1641 179
1776 -4.75 -3.18 1.0000
10 -6 6 1590 221
1724 -4.75 3.18 1.0000
-12 6 -6 1769 138
664 13.29 5.75 1.0000
12 -6 6 1129 164
299 13.29 -5.75 1.0000
-14 6 -6 341 155
636 5.54 -6.60 1.0000
14 -6 6 325 207
610 5.54 6.60 1.0000
-16 6 -6 43 131
12 7.42 4.35 1.0000
16 -6 6 99 198
-92 7.42 -4.35 1.0000
-18 6 -6 578 155
488 1.35 -2.62 1.0000
18 -6 6 748 252
644 1.35 2.62 1.0000
-20 6 -6 52 154

150	-4.26	1.85	1.0000	
20	-6	6	11	253
65	-4.26	-1.85	1.0000	
-22	6	-6	207	187
217	0.11	0.27	1.0000	
22	-6	6	0	268
-57	0.11	-0.27	1.0000	
23	5	-6	2	359
-160	0.33	-1.13	1.0000	
23	5	-6	2	344
-261	0.33	-1.13	1.0000	
21	5	-6	4	326
-34	-0.11	1.81	1.0000	
21	5	-6	385	332
432	-0.11	1.81	1.0000	
19	5	-6	538	296
405	-0.96	2.52	1.0000	
19	5	-6	48	295
15	-0.96	2.52	1.0000	
17	5	-6	190	285
327	-0.90	-4.75	1.0000	
17	5	-6	291	274
460	-0.90	-4.75	1.0000	
15	5	-6	1282	279
1510	3.80	1.39	1.0000	
15	5	-6	881	292
1070	3.80	1.39	1.0000	
13	5	-6	724	246
815	8.55	-1.07	1.0000	
13	5	-6	676	230
761	8.55	-1.07	1.0000	
11	5	-6	227	170
212	18.59	1.08	1.0000	
11	5	-6	256	208
432	18.59	1.08	1.0000	
9	5	-6	2410	140
360	29.04	-3.01	1.0000	
9	5	-6	3154	199
723	29.04	-3.01	1.0000	
7	5	-6	1287	175
380	14.16	1.25	1.0000	
7	5	-6	1001	113
233	14.16	1.25	1.0000	
-7	-5	6	1025	121

244	14.16	-1.25	1.0000	
5	5	-6	3035	236
4247	9.19	-2.98	1.0000	
5	5	-6	3234	324
4482	9.19	-2.98	1.0000	
-5	-5	6	3211	246
4457	9.19	2.98	1.0000	
3	5	-6	5206	336
4989	1.21	2.09	1.0000	
1	5	-6	965	145
434	-8.74	-7.45	1.0000	
-1	5	-6	3858	326
5288	8.93	2.19	1.0000	
-1	5	-6	3988	264
5440	8.93	2.19	1.0000	
1	-5	6	4027	260
5485	8.93	-2.19	1.0000	
1	-5	6	3921	272
5361	8.93	-2.19	1.0000	
-3	5	-6	473	121
715	-3.28	-3.03	1.0000	
-3	5	-6	439	107
673	-3.28	-3.03	1.0000	
-3	5	-6	340	134
548	-3.28	-3.03	1.0000	
3	-5	6	425	113
655	-3.28	3.03	1.0000	
-5	5	-6	2922	261
4109	9.18	2.94	1.0000	
-5	5	-6	2820	222
3987	9.18	2.94	1.0000	
-5	5	-6	2875	249
4053	9.18	2.94	1.0000	
5	-5	6	3139	243
4364	9.18	-2.94	1.0000	
-7	5	-6	5954	345
6843	5.91	-1.11	1.0000	
-7	5	-6	6700	362
7642	5.91	-1.11	1.0000	
7	-5	6	6286	369
7205	5.91	1.11	1.0000	
-9	5	-6	2499	195
2980	6.88	4.66	1.0000	
-9	5	-6	2316	204

2777 6.88 4.66 1.0000
9 -5 6 2166 230
2595 6.88 -4.66 1.0000
-11 5 -6 398 113
125 -5.05 -12.11 1.0000
11 -5 6 314 142
55 -5.05 12.11 1.0000
-13 5 -6 189 137
494 -14.99 5.15 1.0000
13 -5 6 300 198
756 -14.99 -5.15 1.0000
-15 5 -6 1054 163
947 -2.04 -1.13 1.0000
15 -5 6 1153 232
1043 -2.04 1.13 1.0000
-17 5 -6 132 159
444 -8.97 -1.44 1.0000
17 -5 6 72 224
322 -8.97 1.44 1.0000
-19 5 -6 369 152
341 0.64 0.03 1.0000
19 -5 6 146 233
129 0.64 -0.03 1.0000
-21 5 -6 43 149
-51 5.46 -1.55 1.0000
21 -5 6 93 262
138 5.46 1.55 1.0000
-23 5 -6 7 189
25 -0.71 -1.92 1.0000
23 -5 6 208 298
282 -0.71 1.92 1.0000
24 4 -6 3 388
-532 -1.17 -1.04 1.0000
24 4 -6 3 342
-582 -1.17 -1.04 1.0000
22 4 -6 91 331
130 -1.43 -1.11 1.0000
22 4 -6 4 347
-187 -1.43 -1.11 1.0000
20 4 -6 37 306
-420 -0.97 -5.20 1.0000
20 4 -6 47 282
88 -0.97 -5.20 1.0000
18 4 -6 60 282

162	-0.06	4.84	1.0000	
18	4	-6	659	295
950	-0.06	4.84	1.0000	
16	4	-6	1053	264
934	-2.90	1.09	1.0000	
16	4	-6	930	276
818	-2.90	1.09	1.0000	
14	4	-6	1989	259
1559	1.42	-4.68	1.0000	
14	4	-6	2299	351
1835	1.42	-4.68	1.0000	
12	4	-6	833	332
2622	-14.30	13.11	1.0000	
12	4	-6	460	253
1918	-14.30	13.11	1.0000	
10	4	-6	1684	182
526	-24.01	-8.83	1.0000	
10	4	-6	1093	154
98	-24.01	-8.83	1.0000	
8	4	-6	1637	248
3628	-17.70	1.52	1.0000	
8	4	-6	1864	312
3964	-17.70	1.52	1.0000	
-8	-4	6	1893	246
4011	-17.70	-1.52	1.0000	
6	4	-6	1934	231
4314	-22.06	14.47	1.0000	
6	4	-6	1570	312
3709	-22.06	14.47	1.0000	
-6	-4	6	1551	224
3669	-22.06	-14.47	1.0000	
4	4	-6	4937	401
7190	3.53	-16.87	1.0000	
2	4	-6	4835	309
4410	11.15	12.28	1.0000	
0	4	-6	3104	244
2469	2.81	4.42	1.0000	
-2	4	-6	7327	457
10493	4.71	-13.93	1.0000	
-2	4	-6	7254	498
10405	4.71	-13.93	1.0000	
-4	4	-6	4405	344
6551	3.73	16.93	1.0000	
-4	4	-6	4447	312

6603 3.73 16.93 1.0000
-4 4 -6 4073 315
6137 3.73 16.93 1.0000
4 -4 6 4561 314
6763 3.73 -16.93 1.0000
-6 4 -6 2572 130
1056 -7.99 -13.64 1.0000
-6 4 -6 2701 146
1139 -7.99 -13.64 1.0000
-6 4 -6 2547 146
1040 -7.99 -13.64 1.0000
6 -4 6 2228 141
840 -7.99 13.64 1.0000
-8 4 -6 230 106
314 -7.89 -2.51 1.0000
-8 4 -6 234 105
320 -7.89 -2.51 1.0000
8 -4 6 70 132
42 -7.89 2.51 1.0000
8 -4 6 198 126
264 -7.89 2.51 1.0000
-10 4 -6 3223 230
3811 8.76 10.82 1.0000
10 -4 6 2478 246
2954 8.76 -10.82 1.0000
10 -4 6 3195 266
3765 8.76 -10.82 1.0000
-12 4 -6 885 156
845 5.80 -14.07 1.0000
12 -4 6 565 164
453 5.80 14.07 1.0000
-14 4 -6 2223 255
3813 11.08 9.92 1.0000
14 -4 6 1922 330
3396 11.08 -9.92 1.0000
-16 4 -6 413 190
1377 12.12 -8.06 1.0000
16 -4 6 424 266
1396 12.12 8.06 1.0000
-18 4 -6 148 143
204 -2.74 0.59 1.0000
18 -4 6 275 235
350 -2.74 -0.59 1.0000
-20 4 -6 494 165

506 2.70 1.32 1.0000
20 -4 6 396 250
405 2.70 -1.32 1.0000
-22 4 -6 25 164
54 -2.57 1.06 1.0000
22 -4 6 140 282
208 -2.57 -1.06 1.0000
-24 4 -6 1 205
-80 -0.80 0.52 1.0000
24 -4 6 1 318
-83 -0.80 -0.52 1.0000
23 3 -6 192 351
116 2.40 1.82 1.0000
21 3 -6 2 318
-459 -0.20 1.12 1.0000
21 3 -6 42 308
31 -0.20 1.12 1.0000
19 3 -6 611 284
463 2.36 -2.08 1.0000
19 3 -6 123 294
61 2.36 -2.08 1.0000
17 3 -6 464 272
320 2.98 2.39 1.0000
17 3 -6 768 261
581 2.98 2.39 1.0000
15 3 -6 3462 353
3298 -4.01 -3.23 1.0000
15 3 -6 3376 389
3213 -4.01 -3.23 1.0000
13 3 -6 157 209
830 16.39 1.59 1.0000
13 3 -6 106 217
684 16.39 1.59 1.0000
11 3 -6 568 170
639 13.20 -3.63 1.0000
11 3 -6 522 185
577 13.20 -3.63 1.0000
9 3 -6 1523 132
397 19.14 7.30 1.0000
9 3 -6 1589 161
436 19.14 7.30 1.0000
-9 -3 6 1224 129
232 19.14 -7.30 1.0000
7 3 -6 5512 240

1936	25.95	-6.36	1.0000	
7	3	-6	5034	176
1656	25.95	-6.36	1.0000	
-7	-3	6	5246	159
1776	25.95	6.36	1.0000	
5	3	-6	463	162
708	-3.29	3.21	1.0000	
3	3	-6	11395	650
14239	-11.12	-2.95	1.0000	
1	3	-6	4343	385
7088	-17.94	-4.39	1.0000	
-1	3	-6	1391	274
3865	-22.50	3.48	1.0000	
-3	3	-6	10767	586
13509	-11.05	2.81	1.0000	
-3	3	-6	10903	621
13662	-11.05	2.81	1.0000	
-3	3	-6	11335	593
14145	-11.05	2.81	1.0000	
3	-3	6	11477	585
14312	-11.05	-2.81	1.0000	
-5	3	-6	4150	220
3984	1.05	-2.08	1.0000	
-5	3	-6	3909	212
3748	1.05	-2.08	1.0000	
-5	3	-6	3786	244
3627	1.05	-2.08	1.0000	
5	-3	6	4090	220
3920	1.05	2.08	1.0000	
5	-3	6	3355	233
3201	1.05	2.08	1.0000	
-7	3	-6	2482	203
3244	2.77	6.99	1.0000	
-7	3	-6	2594	221
3372	2.77	6.99	1.0000	
7	-3	6	2900	228
3721	2.77	-6.99	1.0000	
7	-3	6	2246	221
2972	2.77	-6.99	1.0000	
-9	3	-6	411	119
635	-0.99	-9.00	1.0000	
9	-3	6	386	140
605	-0.99	9.00	1.0000	
-11	3	-6	234	137

767	-5.80	9.09	1.0000	
11	-3	6	247	178
790	-5.80	-9.09	1.0000	
-13	3	-6	90	131
361	-5.68	-6.97	1.0000	
13	-3	6	42	177
246	-5.68	6.97	1.0000	
-15	3	-6	1477	150
682	-13.46	0.49	1.0000	
15	-3	6	1209	205
503	-13.46	-0.49	1.0000	
-17	3	-6	100	140
149	-0.08	2.01	1.0000	
17	-3	6	230	218
301	-0.08	-2.01	1.0000	
-19	3	-6	213	147
229	0.42	-0.56	1.0000	
19	-3	6	158	231
172	0.42	0.56	1.0000	
-21	3	-6	122	158
65	-2.39	-1.03	1.0000	
21	-3	6	370	266
264	-2.39	1.03	1.0000	
-23	3	-6	125	191
269	5.17	-0.05	1.0000	
23	-3	6	36	298
-10	5.17	0.05	1.0000	
24	2	-6	71	379
49	1.19	-0.78	1.0000	
22	2	-6	41	346
-514	-5.30	-1.67	1.0000	
22	2	-6	277	335
338	-5.30	-1.67	1.0000	
20	2	-6	1	277
-352	-0.04	0.89	1.0000	
20	2	-6	1	282
-286	-0.04	0.89	1.0000	
18	2	-6	629	274
691	-0.88	0.66	1.0000	
18	2	-6	1017	295
1096	-0.88	0.66	1.0000	
16	2	-6	5	245
-111	1.92	0.17	1.0000	
16	2	-6	351	243

328 1.92 0.17 1.0000
14 2 -6 1756 301
1835 -0.19 2.87 1.0000
14 2 -6 1540 258
1614 -0.19 2.87 1.0000
12 2 -6 1967 289
2256 -9.50 -1.77 1.0000
12 2 -6 2136 265
2442 -9.50 -1.77 1.0000
10 2 -6 809 153
388 -7.05 3.56 1.0000
10 2 -6 490 147
178 -7.05 3.56 1.0000
8 2 -6 318 132
646 -26.79 0.33 1.0000
8 2 -6 324 160
764 -26.79 0.33 1.0000
-8 -2 6 304 121
750 -26.79 -0.33 1.0000
6 2 -6 323 162
862 -3.89 -12.23 1.0000
4 2 -6 7573 513
10790 4.79 13.91 1.0000
0 2 -6 744 326
5780 42.40 -1.52 1.0000
-2 2 -6 265 96
503 14.82 2.69 1.0000
-4 2 -6 4402 263
4004 11.03 -12.07 1.0000
-4 2 -6 4158 212
3762 11.03 -12.07 1.0000
-4 2 -6 4136 206
3740 11.03 -12.07 1.0000
4 -2 6 4088 209
3662 11.03 12.07 1.0000
4 -2 6 4294 241
3866 11.03 12.07 1.0000
-6 2 -6 5068 273
5492 -4.99 5.01 1.0000
-6 2 -6 4898 291
5314 -4.99 5.01 1.0000
-6 2 -6 5017 279
5438 -4.99 5.01 1.0000
6 -2 6 4939 284

5363	-4.99	-5.01	1.0000	
6	-2	6	4572	286
4978	-4.99	-5.01	1.0000	
-8	2	-6	1566	169
1929	-1.54	4.86	1.0000	
-8	2	-6	1430	156
1777	-1.54	4.86	1.0000	
8	-2	6	1754	179
2138	-1.54	-4.86	1.0000	
8	-2	6	1421	188
1767	-1.54	-4.86	1.0000	
-10	2	-6	151	118
384	2.72	-16.34	1.0000	
10	-2	6	216	151
579	2.72	16.34	1.0000	
-12	2	-6	2265	158
1151	7.87	12.70	1.0000	
12	-2	6	2034	196
988	7.87	-12.70	1.0000	
-14	2	-6	30	159
687	15.76	-11.18	1.0000	
14	-2	6	9	203
479	15.76	11.18	1.0000	
-16	2	-6	644	156
592	-2.00	4.61	1.0000	
16	-2	6	128	201
86	-2.00	-4.61	1.0000	
-18	2	-6	130	144
340	7.07	0.39	1.0000	
18	-2	6	111	232
307	7.07	-0.39	1.0000	
-20	2	-6	1081	186
1086	-1.21	-2.32	1.0000	
20	-2	6	798	289
803	-1.21	2.32	1.0000	
-22	2	-6	20	157
-10	-3.67	1.29	1.0000	
22	-2	6	118	282
235	-3.67	-1.29	1.0000	
-24	2	-6	85	188
157	0.52	2.95	1.0000	
24	-2	6	12	309
-379	0.52	-2.95	1.0000	
23	1	-6	135	348

151	0.43	2.30	1.0000	
21	1	-6	607	328
646	2.92	-1.64	1.0000	
21	1	-6	15	305
5	2.92	-1.64	1.0000	
19	1	-6	105	259
108	2.59	1.19	1.0000	
19	1	-6	11	268
-21	2.59	1.19	1.0000	
17	1	-6	1414	328
1860	-4.88	-1.98	1.0000	
17	1	-6	1378	349
1819	-4.88	-1.98	1.0000	
15	1	-6	625	231
906	6.58	4.08	1.0000	
15	1	-6	492	216
738	6.58	4.08	1.0000	
13	1	-6	459	161
210	-4.93	-4.61	1.0000	
13	1	-6	555	176
278	-4.93	-4.61	1.0000	
11	1	-6	97	176
576	11.94	3.81	1.0000	
11	1	-6	105	155
597	11.94	3.81	1.0000	
9	1	-6	381	174
1175	16.20	-8.00	1.0000	
9	1	-6	424	208
1263	16.20	-8.00	1.0000	
-9	-1	6	348	159
1102	16.20	8.00	1.0000	
7	1	-6	1147	157
1344	1.02	13.94	1.0000	
7	1	-6	1294	210
1520	1.02	13.94	1.0000	
5	1	-6	4504	339
6047	8.95	-2.30	1.0000	
3	1	-6	658	220
2564	-22.79	-3.46	1.0000	
1	1	-6	17840	416
8481	-36.15	-0.24	1.0000	
-1	1	-6	17012	391
7924	-36.13	0.28	1.0000	
-3	1	-6	4188	348

6879	-17.91		4.25	1.0000
-3	1	-6	4054	316
6705	-17.91		4.25	1.0000
3	-1	6	3955	334
6563	-17.91		-4.25	1.0000
-5	1	-6	736	86
270	-8.51		7.65	1.0000
-5	1	-6	969	98
428	-8.51		7.65	1.0000
-5	1	-6	1065	128
496	-8.51		7.65	1.0000
5	-1	6	907	111
385	-8.51		-7.65	1.0000
5	-1	6	923	100
396	-8.51		-7.65	1.0000
-7	1	-6	4459	336
6665	9.71		-9.12	1.0000
-7	1	-6	4747	343
7016	9.71		-9.12	1.0000
-7	1	-6	4626	347
6869	9.71		-9.12	1.0000
7	-1	6	4986	356
7304	9.71		9.12	1.0000
7	-1	6	4687	335
6941	9.71		9.12	1.0000
-9	1	-6	621	139
1374	1.85		10.37	1.0000
9	-1	6	664	184
1438	1.85		-10.37	1.0000
-11	1	-6	2518	185
2152	-2.91		-6.25	1.0000
11	-1	6	2516	235
2140	-2.91		6.25	1.0000
-13	1	-6	1351	171
1251	-3.83		4.58	1.0000
13	-1	6	1392	217
1292	-3.83		-4.58	1.0000
-15	1	-6	986	183
1447	-5.55		-2.71	1.0000
15	-1	6	663	245
1047	-5.55		2.71	1.0000
-17	1	-6	3444	257
3581	-0.84		0.69	1.0000
17	-1	6	2623	367

2742	-0.84	-0.69	1.0000	
-19	1	-6	450	163
431	-0.93	-0.18	1.0000	
19	-1	6	541	265
520	-0.93	0.18	1.0000	
-21	1	-6	295	175
491	4.37	1.08	1.0000	
21	-1	6	337	287
544	4.37	-1.08	1.0000	
-23	1	-6	110	182
187	-0.23	-2.83	1.0000	
23	-1	6	11	285
-51	-0.23	2.83	1.0000	
24	0	-6	78	355
154	-3.11	-1.67	1.0000	
24	0	-6	17	376
-377	-3.11	-1.67	1.0000	
22	0	-6	10	300
-10	2.39	1.21	1.0000	
20	0	-6	42	264
-290	-4.72	3.09	1.0000	
20	0	-6	42	262
-60	-4.72	3.09	1.0000	
18	0	-6	470	246
487	-1.22	-1.51	1.0000	
18	0	-6	727	265
749	-1.22	-1.51	1.0000	
16	0	-6	1013	242
578	9.59	-1.94	1.0000	
16	0	-6	1099	228
646	9.59	-1.94	1.0000	
14	0	-6	270	190
301	-0.08	4.79	1.0000	
14	0	-6	516	199
570	-0.08	4.79	1.0000	
12	0	-6	922	168
415	8.94	-7.50	1.0000	
12	0	-6	766	152
305	8.94	-7.50	1.0000	
10	0	-6	553	212
1297	-10.45	7.16	1.0000	
10	0	-6	510	184
1229	-10.45	7.16	1.0000	
8	0	-6	1447	153

1096 -2.41 -6.88 1.0000
8 0 -6 1664 203
1289 -2.41 -6.88 1.0000
-8 0 6 1730 147
1352 -2.41 6.88 1.0000
6 0 -6 746 144
636 -5.85 -0.02 1.0000
4 0 -6 1848 187
1376 2.90 -4.21 1.0000
2 0 -6 609 303
5410 42.57 1.37 1.0000
0 0 -6 9983 711
16385 24.35 0.00 1.0000
-2 0 -6 984 328
6423 42.44 1.48 1.0000
-4 0 -6 1987 134
1496 2.73 -4.34 1.0000
-4 0 -6 1759 121
1299 2.73 -4.34 1.0000
-4 0 -6 2106 175
1600 2.73 -4.34 1.0000
4 0 6 1755 132
1296 2.73 4.34 1.0000
4 0 6 1819 156
1351 2.73 4.34 1.0000
-6 0 -6 960 162
843 -5.82 -0.05 1.0000
-6 0 -6 740 115
633 -5.82 -0.05 1.0000
-6 0 -6 731 112
624 -5.82 -0.05 1.0000
6 0 6 749 112
644 -5.82 0.05 1.0000
6 0 6 768 126
662 -5.82 0.05 1.0000
-8 0 -6 1727 141
1338 -2.45 -7.17 1.0000
8 0 6 1582 177
1212 -2.45 7.17 1.0000
-10 0 -6 522 147
1230 -10.01 7.16 1.0000
10 0 6 734 206
1547 -10.01 -7.16 1.0000
-12 0 -6 1227 129

642 8.93 -7.55 1.0000
12 0 6 1094 180
532 8.93 7.55 1.0000
-14 0 -6 520 145
574 -0.12 4.90 1.0000
14 0 6 430 215
476 -0.12 -4.90 1.0000
-16 0 -6 1107 159
650 9.40 -1.69 1.0000
16 0 6 1375 249
861 9.40 1.69 1.0000
-18 0 -6 774 169
804 -1.30 -1.44 1.0000
18 0 6 412 252
431 -1.30 1.44 1.0000
-20 0 -6 371 163
208 -4.71 3.18 1.0000
20 0 6 305 249
157 -4.71 -3.18 1.0000
-22 0 -6 413 175
336 2.37 1.11 1.0000
22 0 6 224 278
166 2.37 -1.11 1.0000
-24 0 -6 145 192
243 -3.05 -1.67 1.0000
24 0 6 51 313
112 -3.05 1.67 1.0000
23 -1 -6 11 310
-364 -0.23 -2.90 1.0000
21 -1 -6 440 306
689 4.61 0.88 1.0000
21 -1 -6 336 293
557 4.61 0.88 1.0000
19 -1 -6 493 269
470 -0.95 -0.12 1.0000
19 -1 -6 240 250
224 -0.95 -0.12 1.0000
17 -1 -6 3496 364
3644 -0.68 0.85 1.0000
17 -1 -6 2976 359
3112 -0.68 0.85 1.0000
15 -1 -6 671 223
1078 -5.90 -2.74 1.0000
15 -1 -6 1083 271

1589	-5.90	-2.74	1.0000	
13	-1	-6	1445	207
1339	-3.90	4.71	1.0000	
13	-1	-6	1503	252
1396	-3.90	4.71	1.0000	
11	-1	-6	2914	225
2525	-2.76	-6.09	1.0000	
11	-1	-6	2760	255
2380	-2.76	-6.09	1.0000	
9	-1	-6	938	174
1794	2.04	9.96	1.0000	
9	-1	-6	865	210
1692	2.04	9.96	1.0000	
-9	1	6	909	174
1754	2.04	-9.96	1.0000	
7	-1	-6	4157	355
6318	9.66	-9.31	1.0000	
7	-1	-6	4111	305
6261	9.66	-9.31	1.0000	
-7	1	6	4204	302
6374	9.66	9.31	1.0000	
5	-1	-6	939	126
417	-8.69	7.42	1.0000	
3	-1	-6	3909	346
6500	-17.82	4.23	1.0000	
1	-1	-6	19138	443
9348	-36.35	0.32	1.0000	
-1	-1	-6	18462	419
8887	-36.28	-0.42	1.0000	
-3	-1	-6	845	197
2867	-22.26	-3.37	1.0000	
3	1	6	638	187
2460	-22.26	3.37	1.0000	
-5	-1	-6	3703	268
5104	8.92	-2.17	1.0000	
-5	-1	-6	4013	267
5466	8.92	-2.17	1.0000	
-5	-1	-6	3923	279
5361	8.92	-2.17	1.0000	
5	1	6	3825	276
5246	8.92	2.17	1.0000	
5	1	6	3861	280
5289	8.92	2.17	1.0000	
-7	-1	-6	1080	139

1285	1.18	14.21	1.0000	
-7	-1	-6	1093	159
1300	1.18	14.21	1.0000	
7	1	6	1146	146
1315	1.18	-14.21	1.0000	
7	1	6	1072	170
1225	1.18	-14.21	1.0000	
-9	-1	-6	345	131
1096	15.83	-8.18	1.0000	
9	1	6	443	186
1294	15.83	8.18	1.0000	
-11	-1	-6	96	132
572	11.91	3.87	1.0000	
11	1	6	113	172
618	11.91	-3.87	1.0000	
-13	-1	-6	471	128
219	-4.98	-4.29	1.0000	
13	1	6	58	149
-183	-4.98	4.29	1.0000	
-15	-1	-6	440	169
678	6.62	3.75	1.0000	
15	1	6	99	220
188	6.62	-3.75	1.0000	
-17	-1	-6	1365	217
1781	-4.65	-1.99	1.0000	
17	1	6	980	287
1332	-4.65	1.99	1.0000	
-19	-1	-6	183	150
188	2.45	1.05	1.0000	
19	1	6	303	253
312	2.45	-1.05	1.0000	
-21	-1	-6	508	195
543	2.89	-1.59	1.0000	
21	1	6	196	264
212	2.89	1.59	1.0000	
-23	-1	-6	9	181
7	0.36	2.40	1.0000	
23	1	6	8	295
-149	0.36	-2.40	1.0000	
24	-2	-6	13	338
-577	0.82	3.02	1.0000	
24	-2	-6	31	345
82	0.82	3.02	1.0000	
22	-2	-6	20	288

-408	-3.68	1.28	1.0000	
22	-2	-6	20	305
-157	-3.68	1.28	1.0000	
20	-2	-6	886	292
889	-1.14	-2.21	1.0000	
20	-2	-6	1169	286
1174	-1.14	-2.21	1.0000	
18	-2	-6	114	224
315	7.14	0.41	1.0000	
18	-2	-6	120	250
326	7.14	0.41	1.0000	
16	-2	-6	536	210
476	-1.80	4.62	1.0000	
16	-2	-6	752	221
687	-1.80	4.62	1.0000	
14	-2	-6	21	191
630	15.88	-11.25	1.0000	
14	-2	-6	22	182
635	15.88	-11.25	1.0000	
12	-2	-6	2702	238
1485	7.86	12.38	1.0000	
12	-2	-6	2390	187
1250	7.86	12.38	1.0000	
10	-2	-6	300	160
791	2.52	-16.14	1.0000	
10	-2	-6	293	143
776	2.52	-16.14	1.0000	
8	-2	-6	1611	167
1989	-1.49	5.11	1.0000	
8	-2	-6	1432	209
1788	-1.49	5.11	1.0000	
-8	2	6	1632	170
2013	-1.49	-5.11	1.0000	
6	-2	-6	5061	328
5497	-5.14	5.20	1.0000	
6	-2	-6	5273	272
5719	-5.14	5.20	1.0000	
4	-2	-6	4349	263
3965	11.10	-12.28	1.0000	
2	-2	-6	504	158
936	14.84	2.43	1.0000	
0	-2	-6	1119	344
6795	42.58	-1.43	1.0000	
2	2	6	195	65

```
33    14.75     2.55  1.0000
  -4   -2   -6     7889       480
11138     4.70    13.83  1.0000
  -4   -2   -6     7536       466
10718     4.70    13.83  1.0000
  -4   -2   -6     7294       468
10428     4.70    13.83  1.0000
   4    2    6     7135       471
10239     4.70   -13.83  1.0000
   4    2    6     7671       480
10879     4.70   -13.83  1.0000
  -6   -2   -6      287       139
797    -4.19   -12.30  1.0000
  -6   -2   -6      306       123
832    -4.19   -12.30  1.0000
  -6   -2   -6      456       140
1091    -4.19   -12.30  1.0000
   6    2    6      304       141
834    -4.19    12.30  1.0000
  -8   -2   -6      318       120
645   -26.44     0.53  1.0000
   8    2    6      304       145
544   -26.44    -0.53  1.0000
 -10   -2   -6      523       108
203    -6.88     3.70  1.0000
  10    2    6      755       152
357    -6.88    -3.70  1.0000
 -12   -2   -6     1346       170
1595    -9.42    -2.09  1.0000
  12    2    6     1474       251
1737    -9.42     2.09  1.0000
 -14   -2   -6     1193       185
1270    -0.09     3.04  1.0000
  14    2    6     1398       254
1479    -0.09    -3.04  1.0000
 -16   -2   -6      111       151
91      1.98     0.40  1.0000
  16    2    6      252       235
224      1.98    -0.40  1.0000
 -18   -2   -6      848       175
920    -0.86     0.71  1.0000
  18    2    6      835       281
907    -0.86    -0.71  1.0000
 -20   -2   -6      209       164
```

180	-0.14	0.88	1.0000	
20	2	6	324	283
288	-0.14	-0.88	1.0000	
-22	-2	-6	335	178
408	-5.37	-1.66	1.0000	
22	2	6	356	319
430	-5.37	1.66	1.0000	
-24	-2	-6	3	192
-146	1.19	-0.78	1.0000	
24	2	6	3	339
-61	1.19	0.78	1.0000	
23	-3	-6	17	317
81	5.25	-0.14	1.0000	
23	-3	-6	671	353
984	5.25	-0.14	1.0000	
21	-3	-6	281	275
190	-2.40	-0.99	1.0000	
21	-3	-6	9	246
-179	-2.40	-0.99	1.0000	
19	-3	-6	842	268
882	0.51	-0.69	1.0000	
17	-3	-6	133	210
182	-0.15	1.83	1.0000	
17	-3	-6	4	198
-87	-0.15	1.83	1.0000	
15	-3	-6	1448	202
654	-13.54	0.57	1.0000	
15	-3	-6	1594	201
758	-13.54	0.57	1.0000	
13	-3	-6	21	153
180	-5.88	-6.81	1.0000	
13	-3	-6	63	161
300	-5.88	-6.81	1.0000	
11	-3	-6	70	143
427	-5.80	9.00	1.0000	
11	-3	-6	135	180
571	-5.80	9.00	1.0000	
9	-3	-6	235	131
383	-1.19	-9.16	1.0000	
9	-3	-6	274	118
441	-1.19	-9.16	1.0000	
-9	3	6	230	129
378	-1.19	9.16	1.0000	
7	-3	-6	3039	218

3856 2.98 6.86 1.0000
7 -3 -6 2889 264
3686 2.98 6.86 1.0000
-7 3 6 2892 229
3690 2.98 -6.86 1.0000
5 -3 -6 3465 242
3293 1.20 -2.27 1.0000
3 -3 -6 11937 664
14862 -11.21 2.88 1.0000
1 -3 -6 1015 221
3216 -22.55 3.30 1.0000
-1 -3 -6 4205 345
6857 -17.77 -4.17 1.0000
-3 -3 -6 13678 734
16786 -11.13 -2.78 1.0000
-3 -3 -6 13822 717
16945 -11.13 -2.78 1.0000
3 3 6 14686 716
17891 -11.13 2.78 1.0000
-5 -3 -6 441 116
672 -3.15 3.16 1.0000
-5 -3 -6 403 108
625 -3.15 3.16 1.0000
-5 -3 -6 393 109
612 -3.15 3.16 1.0000
5 3 6 432 125
661 -3.15 -3.16 1.0000
-7 -3 -6 4895 144
1566 25.99 -6.52 1.0000
7 3 6 4123 184
1139 25.99 6.52 1.0000
-9 -3 -6 1560 105
425 19.00 7.18 1.0000
9 3 6 1193 142
221 19.00 -7.18 1.0000
-11 -3 -6 560 133
635 13.11 -3.40 1.0000
11 3 6 619 192
716 13.11 3.40 1.0000
-13 -3 -6 135 152
786 16.64 1.61 1.0000
13 3 6 262 233
1127 16.64 -1.61 1.0000
-15 -3 -6 3101 240

2938 -4.18 -3.36 1.0000
15 3 6 2524 361
2377 -4.18 3.36 1.0000
-17 -3 -6 136 151
64 2.87 2.36 1.0000
17 3 6 18 248
-158 2.87 -2.36 1.0000
-19 -3 -6 342 162
230 2.45 -2.04 1.0000
19 3 6 339 269
228 2.45 2.04 1.0000
-21 -3 -6 108 177
92 -0.15 1.04 1.0000
21 3 6 264 305
239 -0.15 -1.04 1.0000
-23 -3 -6 329 197
225 2.38 1.92 1.0000
23 3 6 108 321
52 2.38 -1.92 1.0000
24 -4 -6 1 319
-373 -0.90 0.43 1.0000
24 -4 -6 1 326
-288 -0.90 0.43 1.0000
22 -4 -6 127 299
191 -2.45 1.16 1.0000
22 -4 -6 10 267
-243 -2.45 1.16 1.0000
20 -4 -6 237 247
249 2.99 1.27 1.0000
20 -4 -6 296 243
311 2.99 1.27 1.0000
18 -4 -6 216 220
286 -2.70 0.69 1.0000
18 -4 -6 225 241
297 -2.70 0.69 1.0000
16 -4 -6 413 254
1376 12.06 -8.12 1.0000
16 -4 -6 467 286
1472 12.06 -8.12 1.0000
14 -4 -6 2130 331
3718 11.27 10.03 1.0000
14 -4 -6 2020 305
3570 11.27 10.03 1.0000
12 -4 -6 772 184

711	5.81	-14.09	1.0000	
12	-4	-6	718	158
645	5.81	-14.09	1.0000	
10	-4	-6	3301	259
3918	8.89	10.80	1.0000	
10	-4	-6	3034	289
3617	8.89	10.80	1.0000	
8	-4	-6	333	110
453	-7.86	-2.52	1.0000	
8	-4	-6	292	133
398	-7.86	-2.52	1.0000	
-8	4	6	199	134
267	-7.86	2.52	1.0000	
6	-4	-6	2754	132
1167	-8.14	-13.65	1.0000	
6	-4	-6	2793	185
1193	-8.14	-13.65	1.0000	
-6	4	6	2539	162
1029	-8.14	13.65	1.0000	
4	-4	-6	4913	375
7139	3.60	16.77	1.0000	
2	-4	-6	7519	502
10717	4.77	-13.88	1.0000	
0	-4	-6	2825	187
2248	2.81	4.11	1.0000	
-4	-4	-6	4930	342
7212	3.75	-16.85	1.0000	
-4	-4	-6	4640	345
6854	3.75	-16.85	1.0000	
4	4	6	4688	340
6895	3.75	16.85	1.0000	
-6	-4	-6	1560	213
3684	-22.01	14.67	1.0000	
-6	-4	-6	1411	233
3427	-22.01	14.67	1.0000	
-6	-4	-6	1488	214
3561	-22.01	14.67	1.0000	
6	4	6	1515	243
3599	-22.01	-14.67	1.0000	
-8	-4	-6	1662	221
3655	-17.63	1.58	1.0000	
8	4	6	1719	263
3745	-17.63	-1.58	1.0000	
-10	-4	-6	1060	107

```
76   -24.06    -8.95  1.0000
  10    4    6     1051       141
69   -24.06     8.95  1.0000
 -12   -4   -6      329       174
1633   -14.17    13.17  1.0000
  12    4    6      299       251
1567   -14.17   -13.17  1.0000
 -14   -4   -6     2167       201
1727     1.50    -4.60  1.0000
  14    4    6     2434       292
1967     1.50     4.60  1.0000
 -16   -4   -6      769       179
676    -2.58     0.98  1.0000
  16    4    6      576       268
495    -2.58    -0.98  1.0000
 -18   -4   -6      267       169
463    -0.09     4.91  1.0000
  18    4    6      449       283
697    -0.09    -4.91  1.0000
 -20   -4   -6       41       162
77    -0.90    -5.15  1.0000
  20    4    6       76       268
139    -0.90     5.15  1.0000
 -22   -4   -6      285       191
352    -1.44    -1.04  1.0000
  22    4    6      116       307
160    -1.44     1.04  1.0000
 -24   -4   -6      257       221
320    -1.24    -1.05  1.0000
  24    4    6        3       328
-150    -1.24     1.05  1.0000
  23   -5   -6        6       300
-323    -0.58    -2.05  1.0000
  23   -5   -6        6       291
-241    -0.58    -2.05  1.0000
  21   -5   -6       54       258
80      5.50    -1.45  1.0000
  21   -5   -6      303       285
417      5.50    -1.45  1.0000
  19   -5   -6      139       238
126      0.50    -0.06  1.0000
  19   -5   -6      396       229
374      0.50    -0.06  1.0000
  17   -5   -6      189       229
```

543 -8.95 -1.21 1.0000
17 -5 -6 105 214
387 -8.95 -1.21 1.0000
15 -5 -6 641 210
568 -1.86 -1.21 1.0000
15 -5 -6 795 205
714 -1.86 -1.21 1.0000
13 -5 -6 130 169
225 -15.21 4.97 1.0000
13 -5 -6 154 167
376 -15.21 4.97 1.0000
11 -5 -6 342 141
74 -5.19 -11.92 1.0000
11 -5 -6 334 129
68 -5.19 -11.92 1.0000
9 -5 -6 2422 250
2868 6.71 4.80 1.0000
9 -5 -6 2241 213
2668 6.71 4.80 1.0000
7 -5 -6 5967 330
6835 5.85 -1.25 1.0000
7 -5 -6 5651 366
6496 5.85 -1.25 1.0000
-7 5 6 5470 350
6307 5.85 1.25 1.0000
5 -5 -6 3309 296
4586 9.28 3.01 1.0000
5 -5 -6 3671 251
5012 9.28 3.01 1.0000
-5 5 6 3419 279
4714 9.28 -3.01 1.0000
3 -5 -6 442 102
679 -3.18 -3.32 1.0000
3 -5 -6 368 124
587 -3.18 -3.32 1.0000
3 -5 -6 360 97
576 -3.18 -3.32 1.0000
1 -5 -6 4370 316
5888 8.93 2.24 1.0000
1 -5 -6 4089 280
5561 8.93 2.24 1.0000
1 -5 -6 3969 276
5420 8.93 2.24 1.0000
-1 -5 -6 2064 166

1260	-8.48	-7.65	1.0000	
-3	-5	-6	4966	276
4778	1.06	2.23	1.0000	
-5	-5	-6	3384	259
4665	9.24	-2.98	1.0000	
-5	-5	-6	3312	248
4580	9.24	-2.98	1.0000	
-5	-5	-6	3327	275
4598	9.24	-2.98	1.0000	
5	5	6	3547	276
4858	9.24	2.98	1.0000	
-7	-5	-6	1118	93
306	13.79	1.16	1.0000	
-7	-5	-6	1054	106
273	13.79	1.16	1.0000	
7	5	6	1179	141
338	13.79	-1.16	1.0000	
-9	-5	-6	3094	131
683	29.19	-2.98	1.0000	
9	5	6	2958	168
596	29.19	2.98	1.0000	
-11	-5	-6	234	118
178	18.91	1.16	1.0000	
11	5	6	227	163
200	18.91	-1.16	1.0000	
-13	-5	-6	473	148
519	8.41	-1.18	1.0000	
13	5	6	416	216
454	8.41	1.18	1.0000	
-15	-5	-6	906	192
1093	3.89	1.71	1.0000	
15	5	6	695	259
857	3.89	-1.71	1.0000	
-17	-5	-6	171	165
303	-0.89	-4.83	1.0000	
17	5	6	132	247
248	-0.89	4.83	1.0000	
-19	-5	-6	692	175
534	-0.88	2.65	1.0000	
19	5	6	267	261
173	-0.88	-2.65	1.0000	
-21	-5	-6	405	197
450	-0.18	1.77	1.0000	
21	5	6	430	293

476 -0.18 -1.77 1.0000
-23 -5 -6 192 197
213 0.41 -1.10 1.0000
23 5 6 2 293
-183 0.41 1.10 1.0000
22 -6 -6 7 274
8 0.18 0.10 1.0000
22 -6 -6 0 283
-264 0.18 0.10 1.0000
20 -6 -6 29 226
-290 -4.26 1.85 1.0000
20 -6 -6 2 255
21 -4.26 1.85 1.0000
18 -6 -6 632 246
522 1.68 -2.53 1.0000
18 -6 -6 583 227
477 1.68 -2.53 1.0000
16 -6 -6 96 182
-39 7.30 4.30 1.0000
16 -6 -6 24 189
200 7.30 4.30 1.0000
14 -6 -6 349 196
636 5.35 -6.59 1.0000
14 -6 -6 264 190
511 5.35 -6.59 1.0000
12 -6 -6 1410 167
452 13.24 5.91 1.0000
12 -6 -6 1838 166
709 13.24 5.91 1.0000
10 -6 -6 1644 201
1796 -4.53 -3.29 1.0000
10 -6 -6 1661 243
1814 -4.53 -3.29 1.0000
8 -6 -6 315 130
96 -8.96 -4.21 1.0000
8 -6 -6 474 112
212 -8.96 -4.21 1.0000
6 -6 -6 629 189
1371 -10.62 7.63 1.0000
6 -6 -6 811 157
1645 -10.62 7.63 1.0000
-6 6 6 681 202
1451 -10.62 -7.63 1.0000
4 -6 -6 1407 220

3410 -22.00 -14.60 1.0000
4 -6 -6 1473 252
3525 -22.00 -14.60 1.0000
4 -6 -6 1352 207
3313 -22.00 -14.60 1.0000
2 -6 -6 303 140
821 -4.10 12.11 1.0000
2 -6 -6 359 148
920 -4.10 12.11 1.0000
2 -6 -6 457 132
1085 -4.10 12.11 1.0000
0 -6 -6 745 133
639 -6.07 0.06 1.0000
0 -6 -6 746 120
640 -6.07 0.06 1.0000
-2 -6 -6 5633 342
6088 -5.00 -5.04 1.0000
-2 -6 -6 6647 342
7146 -5.00 -5.04 1.0000
2 6 6 5948 330
6411 -5.00 5.04 1.0000
-4 -6 -6 3820 179
1892 -8.10 13.66 1.0000
-6 -6 -6 619 164
1367 -10.56 -7.80 1.0000
-6 -6 -6 732 149
1541 -10.56 -7.80 1.0000
6 6 6 473 182
1134 -10.56 7.80 1.0000
-8 -6 -6 2269 292
5503 -24.80 1.83 1.0000
8 6 6 2455 351
5817 -24.80 -1.83 1.0000
-10 -6 -6 269 185
1879 -19.65 13.12 1.0000
10 6 6 396 260
2206 -19.65 -13.12 1.0000
-12 -6 -6 135 139
610 -8.08 -10.05 1.0000
12 6 6 174 212
695 -8.08 10.05 1.0000
-14 -6 -6 544 172
864 -10.51 7.63 1.0000
14 6 6 292 229

468 -10.51 -7.63 1.0000

-16 -6 -6 440 165

174 5.91 3.32 1.0000

16 6 6 61 233

-63 5.91 -3.32 1.0000

-18 -6 -6 579 190

918 0.86 -6.48 1.0000

18 6 6 663 291

1025 0.86 6.48 1.0000

-20 -6 -6 1226 208

971 -2.28 3.10 1.0000

20 6 6 1069 295

830 -2.28 -3.10 1.0000

-22 -6 -6 149 200

158 2.85 -5.04 1.0000

22 6 6 45 272

-89 2.85 5.04 1.0000

23 -7 -6 218 331

330 3.13 0.46 1.0000

23 -7 -6 13 289

-97 3.13 0.46 1.0000

21 -7 -6 5 234

-216 1.06 -1.52 1.0000

21 -7 -6 5 277

-26 1.06 -1.52 1.0000

19 -7 -6 149 226

70 3.80 -0.42 1.0000

19 -7 -6 242 244

139 3.80 -0.42 1.0000

17 -7 -6 5 215

-15 0.22 -2.02 1.0000

17 -7 -6 5 195

-8 0.22 -2.02 1.0000

15 -7 -6 156 207

539 -9.75 0.08 1.0000

15 -7 -6 62 194

341 -9.75 0.08 1.0000

13 -7 -6 2107 288

2344 -2.98 -6.45 1.0000

13 -7 -6 2224 264

2469 -2.98 -6.45 1.0000

11 -7 -6 1077 211

1448 -3.74 3.00 1.0000

11 -7 -6 1054 232

1421 -3.74 3.00 1.0000
9 -7 -6 2690 279
3155 1.32 -3.50 1.0000
9 -7 -6 2797 245
3271 1.32 -3.50 1.0000
7 -7 -6 1176 223
3146 20.60 0.50 1.0000
7 -7 -6 1224 261
3226 20.60 0.50 1.0000
5 -7 -6 1087 120
287 13.84 -1.24 1.0000
5 -7 -6 1144 109
317 13.84 -1.24 1.0000
3 -7 -6 4519 175
1362 25.90 6.46 1.0000
3 -7 -6 5202 193
1750 25.90 6.46 1.0000
3 -7 -6 4548 159
1378 25.90 6.46 1.0000
1 -7 -6 1188 169
1367 1.26 -14.09 1.0000
1 -7 -6 900 179
1018 1.26 -14.09 1.0000
1 -7 -6 1196 177
1376 1.26 -14.09 1.0000
-1 -7 -6 4196 337
6333 9.71 9.08 1.0000
-1 -7 -6 4158 342
6286 9.71 9.08 1.0000
1 7 6 4275 329
6432 9.71 -9.08 1.0000
-3 -7 -6 2194 244
2926 2.84 -7.12 1.0000
-3 -7 -6 2725 230
3537 2.84 -7.12 1.0000
3 7 6 2467 233
3242 2.84 7.12 1.0000
-5 -7 -6 6128 363
7009 5.79 1.17 1.0000
5 7 6 6477 382
7376 5.79 -1.17 1.0000
-7 -7 -6 1051 212
2924 20.51 -0.40 1.0000
-7 -7 -6 1370 228

3454	20.51	-0.40	1.0000	
7	7	6	1449	258
3586	20.51	0.40	1.0000	
-9	-7	-6	815	118
346	11.38	-0.55	1.0000	
9	7	6	1018	183
498	11.38	0.55	1.0000	
-11	-7	-6	179	167
1132	17.54	-0.89	1.0000	
11	7	6	198	226
1177	17.54	0.89	1.0000	
-13	-7	-6	1883	212
2432	7.25	0.80	1.0000	
13	7	6	1368	329
1835	7.25	-0.80	1.0000	
-15	-7	-6	836	175
588	0.88	-4.07	1.0000	
15	7	6	655	273
438	0.88	4.07	1.0000	
-17	-7	-6	899	202
1124	-1.63	3.26	1.0000	
17	7	6	328	261
468	-1.63	-3.26	1.0000	
-19	-7	-6	55	179
150	-4.47	-3.19	1.0000	
19	7	6	40	247
-132	-4.47	3.19	1.0000	
-21	-7	-6	457	210
474	-0.87	1.29	1.0000	
21	7	6	3	272
-179	-0.87	-1.29	1.0000	
-23	-7	-6	513	224
555	-2.42	-0.07	1.0000	
23	7	6	8	306
-184	-2.42	0.07	1.0000	
22	-8	-6	1	264
-86	-0.13	0.73	1.0000	
22	-8	-6	1	293
-153	-0.13	0.73	1.0000	
20	-8	-6	81	247
186	-4.69	-1.41	1.0000	
20	-8	-6	61	259
153	-4.69	-1.41	1.0000	
18	-8	-6	96	230

65 1.46 1.88 1.0000
18 -8 -6 166 217
126 1.46 1.88 1.0000
16 -8 -6 24 209
51 -1.93 -0.44 1.0000
16 -8 -6 112 190
165 -1.93 -0.44 1.0000
14 -8 -6 258 186
122 4.81 0.58 1.0000
14 -8 -6 136 178
43 4.81 0.58 1.0000
12 -8 -6 2820 295
2638 2.69 2.40 1.0000
12 -8 -6 2848 263
2665 2.69 2.40 1.0000
10 -8 -6 8 147
-68 -0.55 -2.34 1.0000
10 -8 -6 8 134
-50 -0.55 -2.34 1.0000
8 -8 -6 57 137
412 -11.13 -2.28 1.0000
8 -8 -6 71 153
450 -11.13 -2.28 1.0000
6 -8 -6 2471 314
5849 -24.84 -1.86 1.0000
6 -8 -6 2129 337
5307 -24.84 -1.86 1.0000
4 -8 -6 1666 263
3658 -17.59 -1.68 1.0000
4 -8 -6 1754 240
3788 -17.59 -1.68 1.0000
2 -8 -6 303 141
559 -26.62 -0.64 1.0000
2 -8 -6 306 146
772 -26.62 -0.64 1.0000
2 -8 -6 304 142
741 -26.62 -0.64 1.0000
0 -8 -6 1498 193
1131 -2.55 7.08 1.0000
0 -8 -6 1782 182
1384 -2.55 7.08 1.0000
0 -8 -6 1869 202
1462 -2.55 7.08 1.0000
-2 -8 -6 1783 205

2180 -1.60 -4.93 1.0000
2 8 6 2162 219
2599 -1.60 4.93 1.0000
-6 -8 -6 611 113
329 -8.92 4.14 1.0000
-8 -8 -6 130 127
584 -11.11 2.19 1.0000
-8 -8 -6 138 128
602 -11.11 2.19 1.0000
8 8 6 63 153
427 -11.11 -2.19 1.0000
-10 -8 -6 4 137
212 -10.56 -2.45 1.0000
10 8 6 108 199
524 -10.56 2.45 1.0000
-12 -8 -6 826 125
208 -10.80 8.89 1.0000
12 8 6 948 227
262 -10.80 -8.89 1.0000
-14 -8 -6 172 148
81 3.34 -1.33 1.0000
14 8 6 17 224
-127 3.34 1.33 1.0000
-16 -8 -6 2737 265
3224 -3.93 -1.11 1.0000
16 8 6 2024 397
2444 -3.93 1.11 1.0000
-18 -8 -6 21 180
-85 3.16 2.40 1.0000
18 8 6 21 264
-194 3.16 -2.40 1.0000
-20 -8 -6 35 187
198 6.26 -5.19 1.0000
20 8 6 293 311
664 6.26 5.19 1.0000
-22 -8 -6 650 237
845 -2.54 2.10 1.0000
22 8 6 72 324
144 -2.54 -2.10 1.0000
21 -9 -6 16 243
-447 3.40 -0.88 1.0000
21 -9 -6 16 269
-264 3.40 -0.88 1.0000
19 -9 -6 10 243

-178 0.93 -2.62 1.0000
19 -9 -6 10 229
-15 0.93 -2.62 1.0000
17 -9 -6 107 206
164 1.42 2.91 1.0000
17 -9 -6 40 222
73 1.42 2.91 1.0000
15 -9 -6 312 201
427 1.99 -4.60 1.0000
15 -9 -6 508 222
659 1.99 -4.60 1.0000
13 -9 -6 972 237
1455 -6.07 0.09 1.0000
13 -9 -6 734 226
1160 -6.07 0.09 1.0000
11 -9 -6 588 181
523 -0.32 -3.11 1.0000
11 -9 -6 647 170
579 -0.32 -3.11 1.0000
9 -9 -6 305 175
855 10.23 0.29 1.0000
9 -9 -6 186 167
646 10.23 0.29 1.0000
7 -9 -6 644 129
231 11.42 0.62 1.0000
7 -9 -6 833 150
360 11.42 0.62 1.0000
5 -9 -6 2251 138
284 29.02 3.03 1.0000
5 -9 -6 2745 136
502 29.02 3.03 1.0000
3 -9 -6 998 122
127 19.03 -7.19 1.0000
3 -9 -6 837 110
62 19.03 -7.19 1.0000
1 -9 -6 422 179
1245 15.92 8.00 1.0000
3 9 6 519 157
775 -1.06 -9.00 1.0000
5 9 6 2387 256
2839 6.82 4.86 1.0000
-7 -9 -6 2521 214
2988 1.33 3.64 1.0000
7 9 6 2898 291

3397 1.33 -3.64 1.0000
-9 -9 -6 409 153
1012 10.05 -0.42 1.0000
9 9 6 302 188
840 10.05 0.42 1.0000
-11 -9 -6 1601 207
2087 5.00 -0.03 1.0000
11 9 6 1449 317
1911 5.00 0.03 1.0000
-13 -9 -6 174 150
288 1.25 -3.24 1.0000
13 9 6 155 251
263 1.25 3.24 1.0000
-15 -9 -6 156 161
137 0.36 1.52 1.0000
15 9 6 72 247
59 0.36 -1.52 1.0000
-17 -9 -6 741 214
953 -4.07 -2.62 1.0000
17 9 6 653 303
852 -4.07 2.62 1.0000
-19 -9 -6 41 194
-22 -4.94 2.46 1.0000
19 9 6 268 291
192 -4.94 -2.46 1.0000
-21 -9 -6 359 228
553 -3.14 -2.97 1.0000
21 9 6 8 308
6 -3.14 2.97 1.0000
22 -10 -6 1 277
58 -5.16 2.17 1.0000
22 -10 -6 140 323
334 -5.16 2.17 1.0000
20 -10 -6 70 258
137 2.70 1.39 1.0000
20 -10 -6 70 280
137 2.70 1.39 1.0000
18 -10 -6 293 246
212 -3.53 -2.31 1.0000
18 -10 -6 24 221
-3 -3.53 -2.31 1.0000
16 -10 -6 34 209
-189 -4.96 0.78 1.0000
16 -10 -6 240 199

117	-4.96	0.78	1.0000	
14	-10	-6	85	192
215	4.72	4.04	1.0000	
14	-10	-6	45	199
139	4.72	4.04	1.0000	
12	-10	-6	79	171
40	-1.84	-4.75	1.0000	
12	-10	-6	100	181
60	-1.84	-4.75	1.0000	
10	-10	-6	2624	256
2181	-4.03	0.06	1.0000	
10	-10	-6	2729	266
2277	-4.03	0.06	1.0000	
8	-10	-6	32	150
335	-10.74	2.39	1.0000	
8	-10	-6	22	157
302	-10.74	2.39	1.0000	
6	-10	-6	249	211
1829	-19.58	-13.14	1.0000	
6	-10	-6	352	239
2094	-19.58	-13.14	1.0000	
4	-10	-6	1251	133
199	-24.05	8.85	1.0000	
2	-10	-6	741	144
345	-6.95	-3.66	1.0000	
0	-10	-6	613	220
1383	-10.22	-7.29	1.0000	
2	10	6	234	163
640	2.66	-16.22	1.0000	
4	10	6	3869	307
4509	8.58	10.74	1.0000	
-6	-10	-6	1804	179
1953	-4.67	3.20	1.0000	
-8	-10	-6	8	111
-179	-0.43	2.38	1.0000	
8	10	6	50	165
35	-0.43	-2.38	1.0000	
-10	-10	-6	2701	212
2253	-3.97	-0.24	1.0000	
10	10	6	2189	291
1787	-3.97	0.24	1.0000	
-12	-10	-6	2029	197
1724	-2.12	2.83	1.0000	
12	10	6	2287	329

1960	-2.12	-2.83	1.0000	
-14 -10	-6	15		158
19	0.05	1.35	1.0000	
14 10	6	2		254
-78	0.05	-1.35	1.0000	
-16 -10	-6	523		189
438	2.95	-0.60	1.0000	
16 10	6	759		301
657	2.95	0.60	1.0000	
-18 -10	-6	586		218
776	3.20	0.40	1.0000	
18 10	6	450		315
619	3.20	-0.40	1.0000	
-20 -10	-6	1		202
-82	0.68	-0.52	1.0000	
20 10	6	272		312
299	0.68	0.52	1.0000	
-22 -10	-6	50		233
21	4.36	-1.38	1.0000	
22 10	6	408		360
373	4.36	1.38	1.0000	
21 -11	-6	563		278
347	3.94	-2.00	1.0000	
21 -11	-6	498		315
297	3.94	-2.00	1.0000	
19 -11	-6	241		256
425	2.30	4.17	1.0000	
19 -11	-6	184		270
348	2.30	4.17	1.0000	
17 -11	-6	337		256
624	2.41	-6.71	1.0000	
17 -11	-6	527		245
880	2.41	-6.71	1.0000	
15 -11	-6	336		212
224	-1.23	2.70	1.0000	
15 -11	-6	319		207
210	-1.23	2.70	1.0000	
13 -11	-6	550		200
537	-2.16	-2.09	1.0000	
13 -11	-6	566		213
553	-2.16	-2.09	1.0000	
11 -11	-6	497		196
587	1.51	0.73	1.0000	
11 -11	-6	468		206

555	1.51	0.73	1.0000	
9	-11	-6	1796	278
2306	4.93	0.30	1.0000	
9	-11	-6	1587	260
2068	4.93	0.30	1.0000	
7	-11	-6	221	219
1230	17.51	0.84	1.0000	
5	-11	-6	229	141
332	18.88	-1.19	1.0000	
3	-11	-6	558	158
628	13.06	3.53	1.0000	
1	-11	-6	3	151
193	11.84	-3.82	1.0000	
3	11	6	559	213
1294	-5.77	9.05	1.0000	
5	11	6	591	163
284	-5.08	-11.90	1.0000	
-7	-11	-6	1238	173
1643	-3.73	-3.23	1.0000	
7	11	6	854	235
1193	-3.73	3.23	1.0000	
-9	-11	-6	503	156
435	-0.32	3.39	1.0000	
9	11	6	472	217
407	-0.32	-3.39	1.0000	
-11	-11	-6	566	172
663	1.51	-0.79	1.0000	
11	11	6	483	253
573	1.51	0.79	1.0000	
-13	-11	-6	245	156
262	-0.44	0.14	1.0000	
13	11	6	70	207
79	-0.44	-0.14	1.0000	
-15	-11	-6	35	176
119	-5.16	-0.43	1.0000	
15	11	6	36	241
-312	-5.16	0.43	1.0000	
-17	-11	-6	296	199
196	-4.08	-1.29	1.0000	
17	11	6	24	279
-164	-4.08	1.29	1.0000	
-19	-11	-6	650	228
601	-2.38	-1.34	1.0000	
19	11	6	427	320

386 -2.38 1.34 1.0000
-21 -11 -6 59 237
177 -4.41 2.26 1.0000
21 11 6 155 353
328 -4.41 -2.26 1.0000
20 -12 -6 69 273
-251 -6.87 -2.07 1.0000
20 -12 -6 69 255
-65 -6.87 -2.07 1.0000
18 -12 -6 151 223
147 -3.98 3.30 1.0000
18 -12 -6 66 247
48 -3.98 3.30 1.0000
16 -12 -6 11 202
24 4.44 -1.31 1.0000
16 -12 -6 29 218
-133 4.44 -1.31 1.0000
14 -12 -6 239 226
472 -5.13 -1.76 1.0000
14 -12 -6 36 198
151 -5.13 -1.76 1.0000
12 -12 -6 250 201
251 2.44 3.85 1.0000
12 -12 -6 222 182
221 2.44 3.85 1.0000
10 -12 -6 2185 285
1862 -2.09 -2.98 1.0000
10 -12 -6 2293 282
1962 -2.09 -2.98 1.0000
8 -12 -6 572 163
79 -10.76 -8.81 1.0000
6 -12 -6 167 184
683 -8.15 10.09 1.0000
4 -12 -6 441 241
1883 -14.27 -13.20 1.0000
2 -12 -6 1820 259
2104 -9.50 1.89 1.0000
0 -12 -6 1392 192
751 9.00 7.47 1.0000
2 12 6 2435 208
1290 7.70 12.71 1.0000
4 12 6 609 185
508 5.77 -14.02 1.0000
6 12 6 1555 186

539   13.21   5.73 1.0000
  -8 -12 -6   2889   231
2720   2.69   -2.59 1.0000
   8 12   6   2927   332
2751   2.69   2.59 1.0000
 -10 -12 -6   385   167
339   -1.89   4.91 1.0000
  10 12   6   195   224
152   -1.89   -4.91 1.0000
 -12 -12 -6   312   157
315   2.51   -4.01 1.0000
  12 12   6   54   232
36   2.51   4.01 1.0000
 -14 -12 -6   330   186
356   -3.09   1.35 1.0000
  14 12   6   29   231
25   -3.09   -1.35 1.0000
 -16 -12 -6   170   194
333   4.62   2.82 1.0000
  16 12   6   39   245
-25   4.62   -2.82 1.0000
 -18 -12 -6   793   238
908   2.69   -2.43 1.0000
  18 12   6   519   302
613   2.69   2.43 1.0000
 -20 -12 -6   37   232
66   0.52   1.95 1.0000
  20 12   6   11   345
28   0.52   -1.95 1.0000
  19 -13 -6   36   276
21   3.14   -5.70 1.0000
  19 -13 -6   197   250
294   3.14   -5.70 1.0000
  17 -13 -6   43   242
131   2.14   4.62 1.0000
  17 -13 -6   35   216
116   2.14   4.62 1.0000
  15 -13 -6   85   208
178   -3.45   -2.40 1.0000
  15 -13 -6   248   236
400   -3.45   -2.40 1.0000
  13 -13 -6   184   206
187   0.10   -1.00 1.0000
  13 -13 -6   286   192

290 0.10 -1.00 1.0000
11 -13 -6 143 201
158 -0.52 0.13 1.0000
11 -13 -6 51 165
60 -0.52 0.13 1.0000
9 -13 -6 17 183
-60 1.33 3.30 1.0000
7 -13 -6 1440 277
1902 7.03 -0.76 1.0000
5 -13 -6 675 189
756 8.44 1.11 1.0000
3 -13 -6 77 183
620 16.53 -1.51 1.0000
1 -13 -6 497 181
237 -4.99 4.34 1.0000
-1 -13 -6 1509 254
1416 -3.94 -4.53 1.0000
3 13 6 98 173
376 -5.76 -6.87 1.0000
5 13 6 131 204
172 -15.14 5.06 1.0000
-7 -13 -6 2359 235
2611 -3.08 6.49 1.0000
7 13 6 2894 325
3194 -3.08 -6.49 1.0000
-9 -13 -6 753 194
1174 -5.95 -0.23 1.0000
9 13 6 537 271
899 -5.95 0.23 1.0000
-11 -13 -6 904 182
886 -2.22 2.22 1.0000
11 13 6 706 275
689 -2.22 -2.22 1.0000
-13 -13 -6 235 179
237 0.13 1.00 1.0000
13 13 6 177 266
179 0.13 -1.00 1.0000
-15 -13 -6 238 195
210 -1.80 -2.06 1.0000
15 13 6 10 246
-270 -1.80 2.06 1.0000
-17 -13 -6 220 222
361 -4.65 2.16 1.0000
17 13 6 35 235

-121	-4.65	-2.16	1.0000	
-19 -13	-6	110		251
125	-1.94	0.79	1.0000	
19 13	6	6		280
-184	-1.94	-0.79	1.0000	
20 -14	-6	15		292
-51	3.22	0.95	1.0000	
20 -14	-6	123		284
217	3.22	0.95	1.0000	
18 -14	-6	101		244
125	-0.94	-1.99	1.0000	
18 -14	-6	6		257
-38	-0.94	-1.99	1.0000	
16 -14	-6	141		252
277	-4.45	0.91	1.0000	
16 -14	-6	102		217
221	-4.45	0.91	1.0000	
14 -14	-6	340		206
200	4.43	-0.20	1.0000	
14 -14	-6	454		225
290	4.43	-0.20	1.0000	
12 -14	-6	258		208
284	-3.07	-1.45	1.0000	
12 -14	-6	403		204
439	-3.07	-1.45	1.0000	
10 -14	-6	256		201
280	-0.04	-1.29	1.0000	
8 -14	-6	338		202
205	3.31	1.28	1.0000	
6 -14	-6	504		226
802	-10.50	-7.60	1.0000	
4 -14	-6	1988		258
1566	1.50	4.61	1.0000	
2 -14	-6	1508		256
1577	-0.26	-3.01	1.0000	
0 -14	-6	446		213
493	-0.11	-4.87	1.0000	
4 14	6	2476		372
4159	11.14	10.05	1.0000	
6 14	6	404		226
714	5.38	-6.49	1.0000	
-8 -14	-6	167		168
63	4.71	-0.48	1.0000	
8 14	6	221		219

98 4.71 0.48 1.0000
-10 -14 -6 207 175
406 4.66 -4.11 1.0000
10 14 6 51 244
-28 4.66 4.11 1.0000
-12 -14 -6 194 172
408 -5.11 1.81 1.0000
12 14 6 71 271
216 -5.11 -1.81 1.0000
-14 -14 -6 281 195
157 4.39 0.22 1.0000
14 14 6 166 265
72 4.39 -0.22 1.0000
-16 -14 -6 251 225
290 -1.42 -1.08 1.0000
16 14 6 778 310
847 -1.42 1.08 1.0000
-18 -14 -6 320 250
347 -0.85 1.42 1.0000
18 14 6 4 268
-200 -0.85 -1.42 1.0000
-20 -14 -6 353 261
191 4.32 0.48 1.0000
20 14 6 512 312
312 4.32 -0.48 1.0000
19 -15 -6 9 278
-304 -1.65 1.92 1.0000
19 -15 -6 9 265
20 -1.65 1.92 1.0000
17 -15 -6 39 262
108 -1.02 -3.46 1.0000
17 -15 -6 17 241
-13 -1.02 -3.46 1.0000
15 -15 -6 1 211
-60 0.12 -0.99 1.0000
15 -15 -6 10 229
18 0.12 -0.99 1.0000
13 -15 -6 46 183
27 -1.79 2.20 1.0000
13 -15 -6 315 228
278 -1.79 2.20 1.0000
11 -15 -6 11 215
63 -5.09 0.53 1.0000
9 -15 -6 175 209

161 0.16 -1.61 1.0000
7 -15 -6 734 216
500 0.96 4.13 1.0000
5 -15 -6 1004 237
1196 3.71 -1.46 1.0000
3 -15 -6 2728 322
2573 -4.15 3.27 1.0000
1 -15 -6 405 247
629 6.58 -3.87 1.0000
-1 -15 -6 896 279
1341 -5.68 2.65 1.0000
3 15 6 1203 218
487 -13.53 0.54 1.0000
5 15 6 859 250
760 -2.08 -1.15 1.0000
-7 -15 -6 115 181
455 -9.66 -0.11 1.0000
7 15 6 9 218
157 -9.66 0.11 1.0000
-9 -15 -6 629 189
805 2.04 4.55 1.0000
9 15 6 362 251
490 2.04 -4.55 1.0000
-11 -15 -6 319 177
214 -1.13 -2.61 1.0000
11 15 6 263 268
169 -1.13 2.61 1.0000
-13 -15 -6 134 193
249 -3.51 2.38 1.0000
13 15 6 28 277
87 -3.51 -2.38 1.0000
-15 -15 -6 1 209
-10 0.14 0.90 1.0000
15 15 6 367 282
408 0.14 -0.90 1.0000
-17 -15 -6 249 250
255 0.17 0.86 1.0000
17 15 6 408 311
416 0.17 -0.86 1.0000
-19 -15 -6 180 275
191 -3.18 0.23 1.0000
19 15 6 30 318
25 -3.18 -0.23 1.0000
18 -16 -6 8 280

-8 2.30 -0.90 1.0000
18 -16 -6 8 249
-270 2.30 -0.90 1.0000
16 -16 -6 1 248
-1 -0.33 -0.59 1.0000
16 -16 -6 226 227
206 -0.33 -0.59 1.0000
14 -16 -6 346 246
392 -1.45 1.11 1.0000
14 -16 -6 384 244
433 -1.45 1.11 1.0000
12 -16 -6 52 231
147 4.60 -2.84 1.0000
10 -16 -6 671 237
577 2.95 0.65 1.0000
8 -16 -6 2563 349
3025 -3.87 1.16 1.0000
6 -16 -6 534 214
237 5.85 -3.35 1.0000
4 -16 -6 1274 255
1143 -2.70 -1.12 1.0000
2 -16 -6 6 214
-8 2.05 -0.22 1.0000
0 -16 -6 1040 245
597 9.36 1.76 1.0000
-2 -16 -6 459 286
408 -1.98 -4.55 1.0000
4 16 6 397 259
1346 12.12 -8.05 1.0000
6 16 6 74 238
326 7.33 4.31 1.0000
-8 -16 -6 210 191
286 -2.03 0.56 1.0000
8 16 6 38 227
74 -2.03 -0.56 1.0000
-10 -16 -6 35 177
-217 -5.07 -0.82 1.0000
10 16 6 35 237
-105 -5.07 0.82 1.0000
-12 -16 -6 29 193
74 4.40 1.35 1.0000
12 16 6 28 246
-47 4.40 -1.35 1.0000
-14 -16 -6 70 222

172 -4.51 -0.92 1.0000
14 16 6 28 283
-107 -4.51 0.92 1.0000
-16 -16 -6 162 241
145 -0.34 0.60 1.0000
16 16 6 1 307
-36 -0.34 -0.60 1.0000
-18 -16 -6 66 264
92 3.31 2.07 1.0000
18 16 6 357 317
438 3.31 -2.07 1.0000
17 -17 -6 241 241
205 -1.34 0.35 1.0000
17 -17 -6 79 282
59 -1.34 0.35 1.0000
15 -17 -6 1 256
-1 0.17 -0.90 1.0000
15 -17 -6 1 229
-112 0.17 -0.90 1.0000
13 -17 -6 29 240
73 -4.66 -2.19 1.0000
11 -17 -6 353 227
246 -4.05 1.23 1.0000
9 -17 -6 651 265
857 -4.05 2.75 1.0000
7 -17 -6 621 256
818 -1.81 -3.18 1.0000
5 -17 -6 255 231
419 -0.90 4.92 1.0000
3 -17 -6 239 225
139 2.97 -2.33 1.0000
1 -17 -6 1072 297
1459 -4.87 2.03 1.0000
-1 -17 -6 3469 411
3604 -0.78 -0.71 1.0000
5 17 6 50 231
267 -9.02 -1.31 1.0000
-7 -17 -6 177 191
178 0.17 2.04 1.0000
7 17 6 6 235
-279 0.17 -2.04 1.0000
-9 -17 -6 328 192
422 1.33 -2.90 1.0000
9 17 6 149 247

213	1.33	2.90	1.0000	
-11	-17	-6	876	229
1322	2.40	6.60	1.0000	
11	17	6	646	296
1029	2.40	-6.60	1.0000	
-13	-17	-6	274	225
474	2.23	-4.60	1.0000	
13	17	6	23	281
91	2.23	4.60	1.0000	
-15	-17	-6	116	243
220	-0.98	3.42	1.0000	
15	17	6	136	330
248	-0.98	-3.42	1.0000	
-17	-17	-6	319	273
277	-1.38	-0.41	1.0000	
17	17	6	135	335
108	-1.38	0.41	1.0000	
16	-18	-6	255	262
321	3.31	-2.08	1.0000	
16	-18	-6	20	274
-68	3.31	-2.08	1.0000	
14	-18	-6	72	254
83	-0.81	-1.43	1.0000	
12	-18	-6	908	289
1033	2.68	2.49	1.0000	
10	-18	-6	754	282
967	3.20	-0.35	1.0000	
8	-18	-6	7	234
13	3.17	-2.37	1.0000	
6	-18	-6	762	282
1144	0.86	6.39	1.0000	
4	-18	-6	364	253
585	-0.10	-4.83	1.0000	
2	-18	-6	824	268
899	-0.92	-0.70	1.0000	
0	-18	-6	754	272
777	-1.23	1.43	1.0000	
-2	-18	-6	150	278
375	7.12	-0.42	1.0000	
6	18	6	742	271
632	1.52	-2.50	1.0000	
-8	-18	-6	485	207
415	1.55	-1.93	1.0000	
8	18	6	131	266

93	1.55	1.93	1.0000	
-10	-18	-6	575	207
472	-3.48	2.36	1.0000	
10	18	6	82	260
36	-3.48	-2.36	1.0000	
-12	-18	-6	627	225
661	-4.13	-3.36	1.0000	
12	18	6	258	278
265	-4.13	3.36	1.0000	
-14	-18	-6	371	253
426	-0.93	2.09	1.0000	
14	18	6	7	281
-145	-0.93	-2.09	1.0000	
-16	-18	-6	237	275
315	2.28	0.87	1.0000	
16	18	6	158	345
223	2.28	-0.87	1.0000	
15	-19	-6	120	283
126	-3.11	-0.24	1.0000	
13	-19	-6	6	257
-69	-1.92	-0.81	1.0000	
11	-19	-6	390	255
350	-2.46	1.39	1.0000	
9	-19	-6	41	242
-41	-4.99	-2.40	1.0000	
7	-19	-6	39	233
-93	-4.40	3.18	1.0000	
5	-19	-6	368	243
259	-0.88	-2.56	1.0000	
3	-19	-6	413	244
295	2.29	2.09	1.0000	
1	-19	-6	67	261
66	2.54	-1.06	1.0000	
-1	-19	-6	375	281
358	-0.93	0.19	1.0000	
-3	-19	-6	322	319
349	0.29	0.71	1.0000	
-7	-19	-6	377	209
250	3.62	0.41	1.0000	
7	19	6	114	264
49	3.62	-0.41	1.0000	
-9	-19	-6	49	207
88	0.86	2.65	1.0000	
9	19	6	11	279

29 0.86 -2.65 1.0000

-11 -19 -6 730 243

1025 2.27 -4.05 1.0000

11 19 6 380 303

599 2.27 4.05 1.0000

-13 -19 -6 177 257

264 3.17 5.69 1.0000

13 19 6 67 281

101 3.17 -5.69 1.0000

-15 -19 -6 264 271

341 -1.75 -2.10 1.0000

15 19 6 109 292

160 -1.75 2.10 1.0000

14 -20 -6 349 291

189 4.28 -0.55 1.0000

12 -20 -6 204 286

269 0.57 -1.94 1.0000

10 -20 -6 233 268

256 0.79 0.41 1.0000

8 -20 -6 88 252

-207 6.18 5.27 1.0000

6 -20 -6 1506 301

1223 -2.26 -3.07 1.0000

4 -20 -6 193 269

305 -0.94 5.20 1.0000

2 -20 -6 91 266

74 -0.05 -0.84 1.0000

0 -20 -6 190 276

73 -4.78 -3.10 1.0000

-2 -20 -6 520 311

518 -1.26 2.27 1.0000

-4 -20 -6 363 339

379 2.90 -1.26 1.0000

-6 -20 -6 28 212

-176 -4.28 -1.74 1.0000

6 20 6 28 244

-37 -4.28 1.74 1.0000

-8 -20 -6 66 219

165 -4.80 1.45 1.0000

8 20 6 235 292

410 -4.80 -1.45 1.0000

-10 -20 -6 123 225

211 2.77 -1.37 1.0000

10 20 6 101 308

181 2.77 1.37 1.0000
-12 -20 -6 89 254
278 -6.86 1.95 1.0000
12 20 6 168 299
409 -6.86 -1.95 1.0000
-14 -20 -6 12 270
51 3.27 -1.09 1.0000
14 20 6 138 309
240 3.27 1.09 1.0000
11 -21 -6 102 287
247 -4.40 -2.23 1.0000
9 -21 -6 348 290
541 -3.10 3.03 1.0000
7 -21 -6 313 280
317 -0.98 -1.09 1.0000
5 -21 -6 157 272
185 -0.16 -1.81 1.0000
3 -21 -6 2 263
-259 -0.14 -1.09 1.0000
1 -21 -6 229 294
243 2.82 1.72 1.0000
-1 -21 -6 357 318
573 4.43 -0.95 1.0000
-3 -21 -6 519 322
394 -2.34 0.99 1.0000
-7 -21 -6 345 236
361 1.06 1.50 1.0000
7 21 6 45 280
48 1.06 -1.50 1.0000
-9 -21 -6 238 243
337 3.32 0.76 1.0000
9 21 6 15 296
-78 3.32 -0.76 1.0000
-11 -21 -6 1070 255
767 3.87 1.99 1.0000
11 21 6 713 336
470 3.87 -1.99 1.0000
10 -22 -6 209 309
180 4.29 1.48 1.0000
8 -22 -6 107 305
192 -2.46 -2.23 1.0000
6 -22 -6 119 287
121 2.89 5.05 1.0000
4 -22 -6 181 291

235 -1.40 1.20 1.0000
2 -22 -6 302 311
366 -5.34 1.69 1.0000
0 -22 -6 219 317
163 2.35 -1.14 1.0000
-2 -22 -6 79 325
176 -3.66 -1.21 1.0000
-4 -22 -6 11 337
-248 -2.62 -1.14 1.0000
-6 -22 -6 0 251
-185 0.08 -0.17 1.0000
-8 -22 -6 697 261
664 -0.05 -0.67 1.0000
8 22 6 11 301
7 -0.05 0.67 1.0000
-10 -22 -6 515 274
854 -5.22 -2.22 1.0000
10 22 6 234 335
477 -5.22 2.22 1.0000
7 -23 -6 301 319
328 -2.38 0.16 1.0000
5 -23 -6 6 299
9 0.32 1.15 1.0000
3 -23 -6 479 320
356 2.30 -1.85 1.0000
1 -23 -6 266 320
295 0.44 -2.38 1.0000
-1 -23 -6 105 326
183 -0.28 2.91 1.0000
-3 -23 -6 170 348
334 5.14 0.18 1.0000
-5 -23 -6 6 380
-14 -0.64 2.03 1.0000
-7 -23 -6 800 289
1005 3.13 -0.40 1.0000
4 -24 -6 200 337
255 -1.17 1.06 1.0000
2 -24 -6 3 318
-58 1.18 0.79 1.0000
0 -24 -6 188 346
300 -3.09 1.76 1.0000
-2 -24 -6 98 371
177 0.68 -2.95 1.0000
-4 -24 -6 1 342

-365	-0.88	-0.48	1.0000	
6	23	-7	74	328
72	-0.39	-2.22	1.0000	
-6	-23	7	7	380
-181	-0.39	2.22	1.0000	
4	23	-7	29	278
13	1.56	-0.38	1.0000	
-4	-23	7	3	347
-109	1.56	0.38	1.0000	
2	23	-7	357	298
249	1.34	-2.41	1.0000	
-2	-23	7	462	343
338	1.34	2.41	1.0000	
0	23	-7	561	323
579	-0.78	0.49	1.0000	
0	-23	7	16	328
18	-0.78	-0.49	1.0000	
-2	23	-7	303	308
406	-2.34	-0.79	1.0000	
2	-23	7	430	336
552	-2.34	0.79	1.0000	
-4	23	-7	29	333
22	0.91	-1.13	1.0000	
4	-23	7	3	316
-23	0.91	1.13	1.0000	
-6	23	-7	34	347
75	-1.52	1.94	1.0000	
6	-23	7	88	319
149	-1.52	-1.94	1.0000	
9	22	-7	256	379
385	-3.46	-0.33	1.0000	
-9	-22	7	237	421
364	-3.46	0.33	1.0000	
7	22	-7	319	322
311	0.21	1.15	1.0000	
-7	-22	7	2	352
-379	0.21	-1.15	1.0000	
5	22	-7	7	288
-61	-1.08	2.05	1.0000	
-5	-22	7	730	371
650	-1.08	-2.05	1.0000	
3	22	-7	201	267
186	-4.58	0.84	1.0000	
-3	-22	7	370	328

361	-4.58	-0.84	1.0000	
1	22	-7	77	271
166	3.46	1.98	1.0000	
-1	-22	7	216	305
357	3.46	-1.98	1.0000	
-1	22	-7	340	279
300	-1.41	2.19	1.0000	
1	-22	7	380	305
340	-1.41	-2.19	1.0000	
-3	22	-7	572	297
579	0.32	-0.09	1.0000	
3	-22	7	0	284
-374	0.32	0.09	1.0000	
-5	22	-7	472	324
612	2.44	3.68	1.0000	
5	-22	7	296	314
404	2.44	-3.68	1.0000	
-7	22	-7	414	337
460	0.55	-1.82	1.0000	
7	-22	7	146	305
173	0.55	1.82	1.0000	
9	-22	7	2	296
-130	-1.25	-0.38	1.0000	
10	21	-7	29	349
123	2.12	-4.63	1.0000	
8	21	-7	406	310
378	-0.04	-0.63	1.0000	
-8	-21	7	1	378
-206	-0.04	0.63	1.0000	
6	21	-7	29	261
-83	3.75	-2.76	1.0000	
-6	-21	7	261	352
373	3.75	2.76	1.0000	
4	21	-7	348	260
431	3.07	0.38	1.0000	
-4	-21	7	382	341
469	3.07	-0.38	1.0000	
2	21	-7	279	248
268	-1.55	-0.88	1.0000	
-2	-21	7	60	311
53	-1.55	0.88	1.0000	
0	21	-7	663	273
959	0.63	-4.93	1.0000	
0	-21	7	886	348

1225 0.63 4.93 1.0000
-2 21 -7 694 277
718 -0.37 -0.51 1.0000
2 -21 7 387 307
405 -0.37 0.51 1.0000
-4 21 -7 182 265
358 -4.49 1.43 1.0000
4 -21 7 173 294
345 -4.49 -1.43 1.0000
-6 21 -7 512 308
498 -0.30 0.20 1.0000
6 -21 7 0 279
-54 -0.30 -0.20 1.0000
-8 21 -7 7 300
-36 -0.44 2.22 1.0000
8 -21 7 56 290
83 -0.44 -2.22 1.0000
10 -21 7 1 295
14 -1.52 -3.00 1.0000
11 20 -7 160 297
193 -1.28 -2.66 1.0000
11 20 -7 597 375
668 -1.28 -2.66 1.0000
9 20 -7 521 321
871 1.67 6.58 1.0000
7 20 -7 19 248
-75 -3.26 1.85 1.0000
-7 -20 7 139 343
256 -3.26 -1.85 1.0000
5 20 -7 231 234
166 -3.47 1.24 1.0000
-5 -20 7 18 306
-106 -3.47 -1.24 1.0000
3 20 -7 103 234
150 2.09 0.12 1.0000
-3 -20 7 110 314
159 2.09 -0.12 1.0000
1 20 -7 13 230
79 -5.85 2.48 1.0000
-1 -20 7 13 282
79 -5.85 -2.48 1.0000
-1 20 -7 250 225
321 4.28 0.97 1.0000
1 -20 7 56 283

80 4.28 -0.97 1.0000
-3 20 -7 360 244
300 1.88 4.16 1.0000
3 -20 7 232 282
180 1.88 -4.16 1.0000
-5 20 -7 123 249
230 1.50 -3.65 1.0000
5 -20 7 26 275
83 1.50 3.65 1.0000
-7 20 -7 218 287
195 -0.41 0.94 1.0000
7 -20 7 449 277
417 -0.41 -0.94 1.0000
-9 20 -7 219 291
289 2.44 -0.10 1.0000
9 -20 7 123 268
177 2.44 0.10 1.0000
11 -20 7 26 289
-126 -0.22 4.38 1.0000
14 19 -7 647 414
891 4.05 0.68 1.0000
14 19 -7 22 329
-88 4.05 0.68 1.0000
12 19 -7 389 344
520 -2.13 -2.98 1.0000
12 19 -7 390 300
521 -2.13 -2.98 1.0000
10 19 -7 308 299
354 0.96 1.65 1.0000
10 19 -7 182 281
217 0.96 1.65 1.0000
8 19 -7 134 261
40 1.35 -5.21 1.0000
6 19 -7 11 238
-16 1.33 2.58 1.0000
-6 -19 7 194 307
282 1.33 -2.58 1.0000
4 19 -7 388 234
212 0.74 -5.13 1.0000
-4 -19 7 310 294
155 0.74 5.13 1.0000
2 19 -7 281 219
222 2.32 2.40 1.0000
-2 -19 7 82 290

46 2.32 -2.40 1.0000
0 19 -7 404 225
384 0.22 -1.31 1.0000
0 -19 7 476 276
454 0.22 1.31 1.0000
-2 19 -7 452 233
419 -4.53 -4.43 1.0000
2 -19 7 438 275
402 -4.53 4.43 1.0000
-4 19 -7 399 232
383 -1.06 2.07 1.0000
4 -19 7 271 268
257 -1.06 -2.07 1.0000
-6 19 -7 377 246
177 -4.56 2.96 1.0000
6 -19 7 259 263
99 -4.56 -2.96 1.0000
-8 19 -7 557 293
676 0.63 2.91 1.0000
8 -19 7 230 265
307 0.63 -2.91 1.0000
-10 19 -7 569 283
362 -2.34 3.73 1.0000
10 -19 7 474 266
287 -2.34 -3.73 1.0000
12 -19 7 630 285
548 0.73 -1.30 1.0000
14 -19 7 3 281
-102 -1.19 -1.04 1.0000
15 18 -7 729 415
756 0.81 -0.47 1.0000
15 18 -7 343 318
361 0.81 -0.47 1.0000
13 18 -7 14 293
-369 -2.17 2.42 1.0000
13 18 -7 193 329
196 -2.17 2.42 1.0000
11 18 -7 336 296
611 1.27 5.40 1.0000
11 18 -7 13 269
100 1.27 5.40 1.0000
9 18 -7 98 254
54 -0.83 -3.02 1.0000
9 18 -7 614 254

```
507    -0.83    -3.02  1.0000
   7   18   -7       651       239
496    -0.59     2.92  1.0000
  -7  -18    7       575       330
430    -0.59    -2.92  1.0000
   5   18   -7       102       212
66      0.22    -2.81  1.0000
  -5  -18    7       107       279
70      0.22     2.81  1.0000
   3   18   -7       458       225
473    -2.90     1.10  1.0000
  -3  -18    7       708       301
729    -2.90    -1.10  1.0000
   1   18   -7       440       204
196     5.31    -3.15  1.0000
  -1  -18    7       786       273
445     5.31     3.15  1.0000
  -1   18   -7       273       217
294    -4.28     2.34  1.0000
   1  -18    7       435       269
473    -4.28    -2.34  1.0000
  -3   18   -7        89       216
269     6.24    -1.54  1.0000
   3  -18    7        87       258
264     6.24     1.54  1.0000
  -5   18   -7       161       218
228     3.13     0.19  1.0000
   5  -18    7       125       243
186     3.13    -0.19  1.0000
  -7   18   -7       392       244
320     1.55    -0.66  1.0000
   7  -18    7       159       239
114     1.55     0.66  1.0000
  -9   18   -7        44       245
-15    -0.32    -5.75  1.0000
   9  -18    7       101       251
57     -0.32     5.75  1.0000
 -11   18   -7       117       251
166    -1.68     0.83  1.0000
  11  -18    7         5       241
-9     -1.68    -0.83  1.0000
  13  -18    7       178       261
317     2.73     3.73  1.0000
  15  -18    7        24       307
```

52 -1.58 2.02 1.0000
16 17 -7 12 302
47 0.94 3.78 1.0000
16 17 -7 735 458
967 0.94 3.78 1.0000
14 17 -7 473 376
562 -1.64 -0.45 1.0000
14 17 -7 1 290
10 -1.64 -0.45 1.0000
12 17 -7 231 292
189 1.48 -3.47 1.0000
12 17 -7 19 273
-248 1.48 -3.47 1.0000
10 17 -7 689 268
562 -3.28 -3.27 1.0000
10 17 -7 451 270
345 -3.28 -3.27 1.0000
8 17 -7 372 229
330 2.50 2.34 1.0000
6 17 -7 593 223
654 1.09 -1.40 1.0000
-6 -17 7 582 317
642 1.09 1.40 1.0000
4 17 -7 77 213
374 -1.68 10.87 1.0000
-4 -17 7 51 285
299 -1.68 -10.87 1.0000
2 17 -7 43 182
-142 1.58 -5.46 1.0000
-2 -17 7 68 250
49 1.58 5.46 1.0000
0 17 -7 178 204
382 -4.03 3.61 1.0000
0 -17 7 282 252
530 -4.03 -3.61 1.0000
-2 17 -7 18 121
-54 0.89 3.60 1.0000
-2 17 -7 18 191
-131 0.89 3.60 1.0000
2 -17 7 182 240
103 0.89 -3.60 1.0000
-4 17 -7 38 138
175 -5.44 -5.32 1.0000
-4 17 -7 106 222

318 -5.44 -5.32 1.0000
4 -17 7 59 244
222 -5.44 5.32 1.0000
-6 17 -7 315 169
456 -0.87 4.14 1.0000
-6 17 -7 152 222
250 -0.87 4.14 1.0000
6 -17 7 166 249
268 -0.87 -4.14 1.0000
-8 17 -7 65 173
247 -4.03 5.32 1.0000
-8 17 -7 146 236
390 -4.03 5.32 1.0000
8 -17 7 52 231
220 -4.03 -5.32 1.0000
-10 17 -7 131 239
176 3.67 0.25 1.0000
10 -17 7 13 228
10 3.67 -0.25 1.0000
12 -17 7 26 237
-50 -0.72 -4.35 1.0000
14 -17 7 328 289
410 -2.47 -0.46 1.0000
16 -17 7 7 285
-78 2.25 0.50 1.0000
17 16 -7 43 305
84 2.21 0.52 1.0000
17 16 -7 813 440
969 2.21 0.52 1.0000
15 16 -7 299 284
219 -3.95 0.24 1.0000
15 16 -7 892 358
761 -3.95 0.24 1.0000
13 16 -7 13 250
-413 3.05 -0.52 1.0000
13 16 -7 312 307
433 3.05 -0.52 1.0000
11 16 -7 529 273
717 3.04 1.89 1.0000
11 16 -7 432 290
603 3.04 1.89 1.0000
9 16 -7 483 238
399 -0.70 4.76 1.0000
9 16 -7 513 245

427   -0.70   4.76 1.0000
    7   16   -7   1507   347
2084   0.79   -6.20 1.0000
    5   16   -7   215   198
316   -0.55   -2.83 1.0000
   -5 -16   7   324   264
446   -0.55   2.83 1.0000
    3   16   -7   96   203
445   3.61   -9.09 1.0000
   -3 -16   7   128   257
510   3.61   9.09 1.0000
    1   16   -7   4   189
10   -1.96   -0.67 1.0000
   -1 -16   7   405   244
480   -1.96   0.67 1.0000
   -1   16   -7   910   217
467   11.21   -4.59 1.0000
    1 -16   7   739   241
331   11.21   4.59 1.0000
   -3   16   -7   860   149
963   -1.44   -1.60 1.0000
   -3   16   -7   701   229
794   -1.44   -1.60 1.0000
    3 -16   7   804   255
904   -1.44   1.60 1.0000
   -5   16   -7   2007   198
2283   3.69   3.65 1.0000
   -5   16   -7   2333   331
2632   3.69   3.65 1.0000
    5 -16   7   1816   338
2077   3.69   -3.65 1.0000
   -7   16   -7   545   156
278   3.01   -6.92 1.0000
   -7   16   -7   505   215
249   3.01   -6.92 1.0000
    7 -16   7   310   230
112   3.01   6.92 1.0000
   -9   16   -7   45   157
78   -1.74   1.30 1.0000
   -9   16   -7   43   221
75   -1.74   1.30 1.0000
    9 -16   7   217   229
285   -1.74   -1.30 1.0000
  -11   16   -7   272   224

251   -0.62   -2.05 1.0000
 -11 16 -7   6   159
-311   -0.62   -2.05 1.0000
  11 -16   7   74   219
62   -0.62   2.05 1.0000
  13 -16   7   142   245
175   -2.83   3.57 1.0000
  15 -16   7   349   272
300   0.38   -1.38 1.0000
  17 -16   7   20   275
-210   0.83   3.78 1.0000
  18 15 -7   1406   463
1583   -1.42   1.96 1.0000
  16 15 -7   1446   393
1345   0.38   -1.39 1.0000
  14 15 -7   4   262
-72   -1.09   1.35 1.0000
  14 15 -7   579   318
571   -1.09   1.35 1.0000
  12 15 -7   22   252
6   -3.85   2.23 1.0000
  12 15 -7   110   252
134   -3.85   2.23 1.0000
  10 15 -7   174   252
119   -0.08   -2.12 1.0000
  10 15 -7   769   238
649   -0.08   -2.12 1.0000
   8 15 -7   386   218
295   -3.83   0.21 1.0000
   8 15 -7   403   214
310   -3.83   0.21 1.0000
   6 15 -7   591   190
228   -0.53   8.23 1.0000
   4 15 -7   465   215
744   4.46   2.30 1.0000
  -4 -15   7   771   280
1122   4.46   -2.30 1.0000
   2 15 -7   355   220
990   -4.96   14.87 1.0000
  -2 -15   7   654   299
1516   -4.96   -14.87 1.0000
   0 15 -7   6   177
-108   1.36   -1.74 1.0000
   0 -15   7   97   222

```
146      1.36      1.74  1.0000
  -2  15  -7        50       193
144     -5.30    -1.88  1.0000
  -2  15  -7       259       125
459     -5.30    -1.88  1.0000
   2 -15   7       330       235
554     -5.30     1.88  1.0000
  -4  15  -7      1777       196
2143     -1.93     3.58  1.0000
  -4  15  -7      1789       321
2156     -1.93     3.58  1.0000
   4 -15   7      1904       325
2282     -1.93    -3.58  1.0000
  -6  15  -7      1543       180
1212      0.98    -4.36  1.0000
  -6  15  -7      1636       247
1295      0.98    -4.36  1.0000
   6 -15   7      1555       254
1223      0.98     4.36  1.0000
  -8  15  -7       982       177
1024      0.36     1.31  1.0000
  -8  15  -7       854       239
893      0.36     1.31  1.0000
   8 -15   7       526       236
556      0.36    -1.31  1.0000
 -10  15  -7      1216       306
1501     -0.81     3.53  1.0000
 -10  15  -7       915       191
1164     -0.81     3.53  1.0000
  10 -15   7      1073       270
1341     -0.81    -3.53  1.0000
 -12  15  -7       683       229
772      2.35      0.54  1.0000
 -12  15  -7       386       181
453      2.35      0.54  1.0000
  12 -15   7       490       240
566      2.35     -0.54  1.0000
 -14  15  -7       126       173
64      5.11    -0.56  1.0000
  14 -15   7       182       254
112      5.11      0.56  1.0000
  16 -15   7        21       254
-22     -3.95      0.12  1.0000
  18 -15   7        47       289
```

54 0.89 -0.52 1.0000

19 14 -7 1424 464

1538 -1.16 -1.00 1.0000

19 14 -7 28 344

45 -1.16 -1.00 1.0000

17 14 -7 253 313

326 -2.49 -0.48 1.0000

17 14 -7 1554 432

1732 -2.49 -0.48 1.0000

15 14 -7 628 329

516 5.05 0.58 1.0000

15 14 -7 34 264

-87 5.05 0.58 1.0000

13 14 -7 701 286

802 1.79 1.45 1.0000

13 14 -7 64 238

94 1.79 1.45 1.0000

11 14 -7 837 250

692 -0.96 -2.40 1.0000

11 14 -7 699 252

567 -0.96 -2.40 1.0000

9 14 -7 215 232

41 7.08 -3.89 1.0000

9 14 -7 256 198

62 7.08 -3.89 1.0000

7 14 -7 101 207

91 0.30 0.57 1.0000

7 14 -7 233 189

219 0.30 0.57 1.0000

5 14 -7 2 184

185 3.06 -11.61 1.0000

-5 -14 7 37 232

62 3.06 11.61 1.0000

3 14 -7 622 181

394 -1.38 -6.03 1.0000

-3 -14 7 538 231

329 -1.38 6.03 1.0000

1 14 -7 947 276

1469 7.10 -9.11 1.0000

-1 -14 7 1216 263

1805 7.10 9.11 1.0000

-1 14 -7 197 185

263 -1.00 -1.64 1.0000

-1 14 -7 447 125

545 -1.00 -1.64 1.0000
1 -14 7 287 197
366 -1.00 1.64 1.0000
-3 14 -7 2639 360
4022 10.47 4.74 1.0000
-3 14 -7 2642 246
4025 10.47 4.74 1.0000
3 -14 7 3032 365
4501 10.47 -4.74 1.0000
-5 14 -7 828 218
673 -1.13 -2.60 1.0000
-5 14 -7 1232 155
1042 -1.13 -2.60 1.0000
5 -14 7 827 225
672 -1.13 2.60 1.0000
-7 14 -7 808 210
557 -1.06 6.17 1.0000
-7 14 -7 1243 167
934 -1.06 6.17 1.0000
7 -14 7 887 217
628 -1.06 -6.17 1.0000
-9 14 -7 82 193
103 0.83 -0.53 1.0000
-9 14 -7 227 147
262 0.83 -0.53 1.0000
9 -14 7 1 200
-235 0.83 0.53 1.0000
-11 14 -7 730 167
489 -3.04 -4.94 1.0000
-11 14 -7 1265 232
947 -3.04 -4.94 1.0000
11 -14 7 1072 239
784 -3.04 4.94 1.0000
-13 14 -7 7 157
16 -4.45 3.76 1.0000
13 -14 7 45 218
-176 -4.45 -3.76 1.0000
-15 14 -7 63 169
60 -1.15 -1.29 1.0000
15 -14 7 203 252
199 -1.15 1.29 1.0000
17 -14 7 4 260
-8 -1.60 -0.40 1.0000
19 -14 7 286 315

454 4.05 0.64 1.0000
18 13 -7 29 300
-13 2.69 3.81 1.0000
18 13 -7 1144 422
1483 2.69 3.81 1.0000
16 13 -7 37 292
39 -2.75 3.63 1.0000
16 13 -7 528 348
618 -2.75 3.63 1.0000
14 13 -7 255 298
511 -4.30 -3.83 1.0000
14 13 -7 120 257
310 -4.30 -3.83 1.0000
12 13 -7 1076 272
1101 -1.86 -0.03 1.0000
12 13 -7 512 249
528 -1.86 -0.03 1.0000
10 13 -7 1269 272
1693 -3.60 3.47 1.0000
10 13 -7 1349 342
1785 -3.60 3.47 1.0000
8 13 -7 439 223
576 -2.23 1.69 1.0000
8 13 -7 205 211
301 -2.23 1.69 1.0000
6 13 -7 455 208
572 -0.06 7.55 1.0000
4 13 -7 202 207
783 -2.92 11.56 1.0000
-4 -13 7 304 243
973 -2.92 -11.56 1.0000
2 13 -7 399 185
603 6.32 2.31 1.0000
-2 -13 7 763 233
1054 6.32 -2.31 1.0000
0 13 -7 1028 129
816 -2.49 10.83 1.0000
0 13 -7 714 196
512 -2.49 10.83 1.0000
0 -13 7 1058 212
860 -2.49 -10.83 1.0000
-2 13 -7 984 280
1570 -1.99 -7.10 1.0000
-2 13 -7 1158 170

1788	-1.99	-7.10	1.0000	
2	-13	7	1469	263
2171	-1.99	7.10	1.0000	
-4	13	-7	252	141
750	4.66	-8.82	1.0000	
-4	13	-7	193	213
644	4.66	-8.82	1.0000	
4	-13	7	173	192
607	4.66	8.82	1.0000	
-6	13	-7	1271	282
1495	-4.06	-3.26	1.0000	
-6	13	-7	1405	185
1641	-4.06	-3.26	1.0000	
6	-13	7	1053	239
1255	-4.06	3.26	1.0000	
-8	13	-7	865	169
920	4.54	-8.60	1.0000	
-8	13	-7	956	213
1020	4.54	-8.60	1.0000	
8	-13	7	712	224
744	4.54	8.60	1.0000	
-10	13	-7	6	176
29	3.37	2.89	1.0000	
-10	13	-7	26	141
-111	3.37	2.89	1.0000	
10	-13	7	26	191
-159	3.37	-2.89	1.0000	
-12	13	-7	12	149
46	3.10	-0.63	1.0000	
-12	13	-7	209	182
320	3.10	-0.63	1.0000	
12	-13	7	13	206
-79	3.10	0.63	1.0000	
-14	13	-7	372	171
441	1.89	-1.35	1.0000	
14	-13	7	286	241
346	1.89	1.35	1.0000	
-16	13	-7	12	161
-5	2.96	0.42	1.0000	
16	-13	7	12	234
-195	2.96	-0.42	1.0000	
18	-13	7	14	269
-57	-2.20	2.35	1.0000	
19	12	-7	184	337

142 0.66 -1.27 1.0000
19 12 -7 1158 425
1050 0.66 -1.27 1.0000
17 12 -7 149 292
52 -0.70 -4.34 1.0000
17 12 -7 305 346
156 -0.70 -4.34 1.0000
15 12 -7 720 316
818 2.40 -0.60 1.0000
15 12 -7 518 277
601 2.40 -0.60 1.0000
13 12 -7 211 250
320 3.07 0.57 1.0000
13 12 -7 220 246
332 3.07 0.57 1.0000
11 12 -7 443 232
373 6.29 -0.13 1.0000
11 12 -7 571 225
498 6.29 -0.13 1.0000
9 12 -7 244 222
275 -0.58 3.10 1.0000
9 12 -7 588 208
643 -0.58 3.10 1.0000
7 12 -7 491 213
420 5.37 -7.35 1.0000
7 12 -7 608 193
542 5.37 -7.35 1.0000
5 12 -7 533 187
373 -1.11 -3.97 1.0000
3 12 -7 537 172
157 1.97 -9.02 1.0000
-3 -12 7 1109 195
513 1.97 9.02 1.0000
1 12 -7 558 191
820 -0.86 -4.43 1.0000
1 12 -7 701 137
991 -0.86 -4.43 1.0000
-1 -12 7 824 204
1137 -0.86 4.43 1.0000
-1 12 -7 468 117
486 5.94 0.80 1.0000
-1 12 -7 208 170
202 5.94 0.80 1.0000
1 -12 7 425 169

440 5.94 -0.80 1.0000
-3 12 -7 298 172
235 -5.62 7.07 1.0000
-3 12 -7 555 125
513 -5.62 7.07 1.0000
3 -12 7 459 168
404 -5.62 -7.07 1.0000
-5 12 -7 2092 224
1421 5.82 8.60 1.0000
-5 12 -7 2248 171
1553 5.82 8.60 1.0000
5 -12 7 1651 199
1055 5.82 -8.60 1.0000
-7 12 -7 1741 212
984 -0.93 9.35 1.0000
-7 12 -7 1994 176
1177 -0.93 9.35 1.0000
7 -12 7 1409 210
739 -0.93 -9.35 1.0000
-9 12 -7 312 179
337 -4.98 1.62 1.0000
-9 12 -7 601 159
649 -4.98 1.62 1.0000
9 -12 7 297 194
320 -4.98 -1.62 1.0000
-11 12 -7 187 149
284 -4.51 2.75 1.0000
-11 12 -7 177 182
271 -4.51 2.75 1.0000
11 -12 7 37 195
-72 -4.51 -2.75 1.0000
-13 12 -7 1126 201
1157 -1.83 -0.04 1.0000
-13 12 -7 845 177
871 -1.83 -0.04 1.0000
13 -12 7 861 245
887 -1.83 0.04 1.0000
-15 12 -7 301 161
358 -3.93 -2.30 1.0000
15 -12 7 28 227
-105 -3.93 2.30 1.0000
-17 12 -7 20 163
-82 1.53 3.59 1.0000
17 -12 7 159 263

123	1.53	-3.59	1.0000	
-19	12	-7	18	186
-36	-2.02	3.04	1.0000	
19	-12	7	206	289
299	-2.02	-3.04	1.0000	
20	11	-7	141	328
283	-0.17	4.45	1.0000	
20	11	-7	1235	441
1609	-0.17	4.45	1.0000	
18	11	-7	366	348
445	-1.56	-0.86	1.0000	
18	11	-7	204	304
264	-1.56	-0.86	1.0000	
16	11	-7	689	303
663	-0.59	1.90	1.0000	
16	11	-7	279	281
261	-0.59	1.90	1.0000	
14	11	-7	1000	262
715	-3.13	5.04	1.0000	
14	11	-7	672	247
439	-3.13	5.04	1.0000	
12	11	-7	37	220
-116	-4.55	-2.70	1.0000	
12	11	-7	36	221
60	-4.55	-2.70	1.0000	
10	11	-7	450	192
598	-4.21	-4.09	1.0000	
10	11	-7	375	226
508	-4.21	-4.09	1.0000	
8	11	-7	181	183
117	-0.79	2.29	1.0000	
8	11	-7	8	197
-174	-0.79	2.29	1.0000	
6	11	-7	119	152
125	0.27	0.10	1.0000	
6	11	-7	28	182
31	0.27	0.10	1.0000	
4	11	-7	757	274
1481	5.21	8.53	1.0000	
2	11	-7	386	102
277	2.16	10.32	1.0000	
-2	-11	7	354	170
248	2.16	-10.32	1.0000	
0	11	-7	437	97

72 2.75 -10.52 1.0000
0 11 -7 375 158
48 2.75 -10.52 1.0000
0 -11 7 960 163
346 2.75 10.52 1.0000
-2 11 -7 1183 149
1513 8.40 0.37 1.0000
-2 11 -7 967 205
1260 8.40 0.37 1.0000
2 -11 7 946 210
1241 8.40 -0.37 1.0000
-4 11 -7 79 186
614 -4.50 -17.69 1.0000
-4 11 -7 130 137
813 -4.50 -17.69 1.0000
4 -11 7 66 162
543 -4.50 17.69 1.0000
-6 11 -7 2613 275
4704 6.31 -15.53 1.0000
-6 11 -7 2916 355
5111 6.31 -15.53 1.0000
6 -11 7 2722 332
4843 6.31 15.53 1.0000
-8 11 -7 2053 273
2081 0.61 -6.04 1.0000
-8 11 -7 1702 194
1723 0.61 -6.04 1.0000
8 -11 7 1945 259
1971 0.61 6.04 1.0000
-10 11 -7 865 219
1447 5.45 -8.41 1.0000
-10 11 -7 639 180
1141 5.45 -8.41 1.0000
10 -11 7 564 225
1042 5.45 8.41 1.0000
-12 11 -7 591 162
511 6.29 0.25 1.0000
-12 11 -7 511 174
433 6.29 0.25 1.0000
12 -11 7 510 207
433 6.29 -0.25 1.0000
-14 11 -7 751 162
620 -1.08 2.32 1.0000
14 -11 7 800 245

664 -1.08 -2.32 1.0000
-16 11 -7 288 171
434 3.13 -1.92 1.0000
16 -11 7 471 270
655 3.13 1.92 1.0000
-18 11 -7 41 180
-32 1.19 -5.42 1.0000
18 -11 7 7 252
80 1.19 5.42 1.0000
-20 11 -7 90 202
113 -1.30 2.76 1.0000
20 -11 7 238 290
281 -1.30 -2.76 1.0000
21 10 -7 787 332
1007 -1.51 -2.93 1.0000
21 10 -7 14 359
-82 -1.51 -2.93 1.0000
19 10 -7 1268 282
937 -2.38 -3.90 1.0000
19 10 -7 213 318
91 -2.38 -3.90 1.0000
17 10 -7 341 256
414 3.48 -0.11 1.0000
17 10 -7 260 272
323 3.48 -0.11 1.0000
15 10 -7 1385 335
1692 -0.82 -3.58 1.0000
15 10 -7 1234 302
1524 -0.82 -3.58 1.0000
13 10 -7 28 230
-52 3.49 -2.96 1.0000
13 10 -7 28 200
-44 3.49 -2.96 1.0000
11 10 -7 496 223
947 5.50 8.34 1.0000
11 10 -7 179 232
448 5.50 8.34 1.0000
9 10 -7 133 165
54 3.52 2.58 1.0000
9 10 -7 25 182
-56 3.52 2.58 1.0000
7 10 -7 293 177
117 -2.01 5.24 1.0000
7 10 -7 642 170

363 -2.01 5.24 1.0000
5 10 -7 917 213
1079 -0.47 2.20 1.0000
3 10 -7 397 109
407 -4.76 -5.77 1.0000
3 10 -7 98 162
54 -4.76 -5.77 1.0000
-3 -10 7 408 164
422 -4.76 5.77 1.0000
1 10 -7 764 127
970 -3.15 0.14 1.0000
1 10 -7 516 182
687 -3.15 0.14 1.0000
-1 -10 7 654 175
846 -3.15 -0.14 1.0000
-1 10 -7 390 201
1065 -5.93 9.67 1.0000
-1 10 -7 343 133
986 -5.93 9.67 1.0000
1 -10 7 457 197
1173 -5.93 -9.67 1.0000
-3 10 -7 2045 182
2465 5.36 7.07 1.0000
-3 10 -7 1924 269
2329 5.36 7.07 1.0000
3 -10 7 2244 231
2681 5.36 -7.07 1.0000
-5 10 -7 571 175
1965 -6.09 22.62 1.0000
-5 10 -7 515 252
1841 -6.09 22.62 1.0000
5 -10 7 680 219
2206 -6.09 -22.62 1.0000
-7 10 -7 172 153
74 1.36 6.83 1.0000
-7 10 -7 330 130
210 1.36 6.83 1.0000
7 -10 7 390 152
273 1.36 -6.83 1.0000
-9 10 -7 339 159
432 -3.63 7.97 1.0000
-9 10 -7 436 142
555 -3.63 7.97 1.0000
9 -10 7 232 169

283 -3.63 -7.97 1.0000
-11 10 -7 646 170
818 -4.21 3.93 1.0000
-11 10 -7 786 174
979 -4.21 3.93 1.0000
11 -10 7 493 202
640 -4.21 -3.93 1.0000
-13 10 -7 1024 233
1408 -3.50 -3.52 1.0000
-13 10 -7 1416 222
1863 -3.50 -3.52 1.0000
13 -10 7 1061 265
1451 -3.50 3.52 1.0000
-15 10 -7 286 156
211 -0.02 2.28 1.0000
15 -10 7 713 239
591 -0.02 -2.28 1.0000
-17 10 -7 128 149
65 -3.34 3.25 1.0000
17 -10 7 380 244
279 -3.34 -3.25 1.0000
-19 10 -7 54 174
73 1.10 -1.76 1.0000
19 -10 7 72 263
95 1.10 1.76 1.0000
-21 10 -7 21 192
105 2.11 4.61 1.0000
21 -10 7 151 319
325 2.11 -4.61 1.0000
22 9 -7 741 321
832 -1.35 -0.46 1.0000
22 9 -7 31 413
52 -1.35 -0.46 1.0000
20 9 -7 8 358
-125 2.41 0.10 1.0000
20 9 -7 602 293
717 2.41 0.10 1.0000
18 9 -7 42 300
-347 -0.21 5.63 1.0000
18 9 -7 312 274
270 -0.21 5.63 1.0000
16 9 -7 117 246
166 -1.70 -1.22 1.0000
16 9 -7 7 248

20 -1.70 -1.22 1.0000
14 9 -7 1 233
-102 0.82 0.36 1.0000
14 9 -7 1 225
-71 0.82 0.36 1.0000
12 9 -7 38 224
-35 -5.12 -1.60 1.0000
12 9 -7 321 207
345 -5.12 -1.60 1.0000
10 9 -7 123 223
122 -3.60 -7.92 1.0000
10 9 -7 378 195
473 -3.60 -7.92 1.0000
8 9 -7 879 218
1306 2.22 -5.71 1.0000
8 9 -7 667 234
1043 2.22 -5.71 1.0000
6 9 -7 1193 215
1374 0.68 -2.09 1.0000
6 9 -7 748 205
893 0.68 -2.09 1.0000
4 9 -7 470 190
929 9.20 -5.76 1.0000
2 9 -7 1149 120
844 7.18 0.23 1.0000
2 9 -7 652 174
417 7.18 0.23 1.0000
-2 -9 7 1174 170
861 7.18 -0.23 1.0000
0 9 -7 265 179
473 12.27 4.52 1.0000
0 9 -7 393 117
693 12.27 4.52 1.0000
0 -9 7 312 150
557 12.27 -4.52 1.0000
-2 9 -7 518 198
1325 -0.73 -24.57 1.0000
-2 9 -7 462 139
1177 -0.73 -24.57 1.0000
2 -9 7 555 182
1454 -0.73 24.57 1.0000
-4 9 -7 2032 204
3105 5.04 -12.09 1.0000
-4 9 -7 1759 267

2757	5.04	-12.09	1.0000	
4	-9	7	2091	232
3178	5.04	12.09	1.0000	
-6	9	-7	296	147
1126	-1.10	-21.70	1.0000	
-6	9	-7	271	185
1055	-1.10	-21.70	1.0000	
6	-9	7	247	166
960	-1.10	21.70	1.0000	
-8	9	-7	1630	176
727	3.56	-12.30	1.0000	
-8	9	-7	1593	150
702	3.56	-12.30	1.0000	
8	-9	7	1402	165
576	3.56	12.30	1.0000	
-10	9	-7	26	137
-113	3.63	-2.57	1.0000	
-10	9	-7	26	116
-162	3.63	-2.57	1.0000	
10	-9	7	26	150
-39	3.63	2.57	1.0000	
-12	9	-7	955	168
1034	-0.65	-3.13	1.0000	
-12	9	-7	884	166
960	-0.65	-3.13	1.0000	
12	-9	7	765	223
834	-0.65	3.13	1.0000	
-14	9	-7	309	142
92	7.08	3.85	1.0000	
14	-9	7	243	209
55	7.08	-3.85	1.0000	
-16	9	-7	646	170
550	-0.73	-4.74	1.0000	
16	-9	7	634	238
540	-0.73	4.74	1.0000	
-18	9	-7	128	150
80	-0.81	3.03	1.0000	
18	-9	7	13	233
-188	-0.81	-3.03	1.0000	
-20	9	-7	266	198
521	1.74	-6.56	1.0000	
20	-9	7	205	291
433	1.74	6.56	1.0000	
-22	9	-7	16	204

-164	-3.48	0.41	1.0000	
22	-9	7	65	321
136	-3.48	-0.41	1.0000	
21	8	-7	251	317
313	-0.42	-2.32	1.0000	
21	8	-7	7	357
-341	-0.42	-2.32	1.0000	
19	8	-7	442	338
542	0.54	-2.81	1.0000	
19	8	-7	356	308
446	0.54	-2.81	1.0000	
17	8	-7	61	286
-223	-4.01	-5.46	1.0000	
17	8	-7	1	264
65	-4.01	-5.46	1.0000	
15	8	-7	755	271
791	0.29	-1.22	1.0000	
15	8	-7	510	263
539	0.29	-1.22	1.0000	
13	8	-7	895	215
944	4.53	8.52	1.0000	
13	8	-7	746	260
779	4.53	8.52	1.0000	
11	8	-7	1594	280
1610	0.58	5.90	1.0000	
11	8	-7	1307	272
1317	0.58	5.90	1.0000	
9	8	-7	1180	226
429	3.53	12.41	1.0000	
9	8	-7	1698	178
768	3.53	12.41	1.0000	
7	8	-7	4318	336
2575	-2.05	13.00	1.0000	
7	8	-7	4655	242
2837	-2.05	13.00	1.0000	
5	8	-7	2352	282
1584	-8.22	3.65	1.0000	
5	8	-7	2717	205
1888	-8.22	3.65	1.0000	
-5	-8	7	2133	213
1405	-8.22	-3.65	1.0000	
3	8	-7	2275	203
1080	-6.21	11.60	1.0000	
-3	-8	7	2425	198

1184	-6.21	-11.60	1.0000	
-3	-8	7	2735	199
1404	-6.21	-11.60	1.0000	
1	8	-7	2471	161
2109	-10.36	-0.47	1.0000	
1	8	-7	2396	239
2038	-10.36	-0.47	1.0000	
-1	-8	7	2303	218
1945	-10.36	0.47	1.0000	
-1	-8	7	2329	196
1970	-10.36	0.47	1.0000	
-1	8	-7	418	102
210	-4.30	3.50	1.0000	
-1	8	-7	41	143
-37	-4.30	3.50	1.0000	
1	-8	7	362	109
169	-4.30	-3.50	1.0000	
1	-8	7	436	145
221	-4.30	-3.50	1.0000	
-3	8	-7	2220	176
793	-5.36	26.02	1.0000	
-3	8	-7	2485	129
999	-5.36	26.02	1.0000	
3	-8	7	2272	134
817	-5.36	-26.02	1.0000	
-5	8	-7	1218	159
469	7.78	8.52	1.0000	
-5	8	-7	1539	130
675	7.78	8.52	1.0000	
5	-8	7	1505	141
652	7.78	-8.52	1.0000	
-7	8	-7	1271	266
2941	-5.58	18.67	1.0000	
-7	8	-7	1385	213
3120	-5.58	18.67	1.0000	
7	-8	7	1323	243
3021	-5.58	-18.67	1.0000	
-9	8	-7	986	215
1427	2.20	5.61	1.0000	
-9	8	-7	945	177
1377	2.20	5.61	1.0000	
9	-8	7	927	214
1356	2.20	-5.61	1.0000	
-11	8	-7	186	127

120   -0.69   -2.30 1.0000
 -11   8   -7   36   131
11   -0.69   -2.30 1.0000
  11   -8   7   164   169
103   -0.69   2.30 1.0000
 -13   8   -7   626   145
789   -2.25   -1.70 1.0000
 -13   8   -7   585   167
743   -2.25   -1.70 1.0000
  13   -8   7   530   215
681   -2.25   1.70 1.0000
 -15   8   -7   529   157
420   -3.80   -0.28 1.0000
  15   -8   7   400   219
305   -3.80   0.28 1.0000
 -17   8   -7   427   162
383   2.61   -2.45 1.0000
  17   -8   7   276   231
237   2.61   2.45 1.0000
 -19   8   -7   137   158
42   1.32   5.26 1.0000
 -21   8   -7   43   182
35   0.07   0.61 1.0000
  21   -8   7   222   290
203   0.07   -0.61 1.0000
  22   7   -7   5   351
-23   0.50   1.86 1.0000
  22   7   -7   404   379
453   0.50   1.86 1.0000
  20   7   -7   380   335
346   -0.49   -1.03 1.0000
  20   7   -7   248   318
221   -0.49   -1.03 1.0000
  18   7   -7   4   274
-26   1.45   0.73 1.0000
  18   7   -7   22   308
8   1.45   0.73 1.0000
  16   7   -7   377   265
159   3.01   6.70 1.0000
  16   7   -7   72   255
-40   3.01   6.70 1.0000
  14   7   -7   112   262
22   -1.03   -6.10 1.0000
  14   7   -7   735   261

500	-1.03	-6.10	1.0000	
12	7	-7	1522	210
821	-0.91	-9.35	1.0000	
12	7	-7	1120	276
532	-0.91	-9.35	1.0000	
10	7	-7	238	165
139	1.27	-6.85	1.0000	
10	7	-7	277	231
174	1.27	-6.85	1.0000	
8	7	-7	1655	260
3560	-5.77	-18.76	1.0000	
8	7	-7	1094	333
2678	-5.77	-18.76	1.0000	
6	7	-7	1695	142
725	14.23	-8.47	1.0000	
6	7	-7	1283	201
444	14.23	-8.47	1.0000	
-6	-7	7	1615	157
675	14.23	8.47	1.0000	
4	7	-7	555	196
668	4.37	-2.08	1.0000	
-4	-7	7	475	136
580	4.37	2.08	1.0000	
2	7	-7	3753	384
6102	12.70	-10.29	1.0000	
-2	-7	7	3833	321
6215	12.70	10.29	1.0000	
-2	-7	7	3537	314
5833	12.70	10.29	1.0000	
0	7	-7	1016	221
1124	8.29	-3.71	1.0000	
0	7	-7	1339	142
1479	8.29	-3.71	1.0000	
0	-7	7	1284	170
1420	8.29	3.71	1.0000	
0	-7	7	1385	156
1530	8.29	3.71	1.0000	
-2	7	-7	965	145
1607	6.86	-6.59	1.0000	
-2	7	-7	710	221
1266	6.86	-6.59	1.0000	
2	-7	7	1034	196
1704	6.86	6.59	1.0000	
2	-7	7	916	158

1549 6.86 6.59 1.0000
-4 7 -7 2242 254
4748 -1.62 -23.44 1.0000
-4 7 -7 2047 312
4449 -1.62 -23.44 1.0000
4 -7 7 2364 272
4921 -1.62 23.44 1.0000
-6 7 -7 897 181
1261 -3.16 -12.91 1.0000
-6 7 -7 673 141
961 -3.16 -12.91 1.0000
6 -7 7 798 159
1113 -3.16 12.91 1.0000
-8 7 -7 4774 212
2925 -2.22 -13.05 1.0000
-8 7 -7 4613 248
2799 -2.22 -13.05 1.0000
8 -7 7 4233 234
2505 -2.22 13.05 1.0000
-10 7 -7 960 145
613 -1.99 -5.23 1.0000
-10 7 -7 657 139
376 -1.99 -5.23 1.0000
10 -7 7 684 160
396 -1.99 5.23 1.0000
-12 7 -7 716 154
647 5.45 7.44 1.0000
-12 7 -7 955 148
894 5.45 7.44 1.0000
12 -7 7 708 203
646 5.45 -7.44 1.0000
-14 7 -7 371 144
351 0.35 -0.58 1.0000
14 -7 7 360 207
340 0.35 0.58 1.0000
-16 7 -7 1050 218
1533 0.69 6.19 1.0000
16 -7 7 1190 291
1702 0.69 -6.19 1.0000
-18 7 -7 430 155
308 -0.65 -2.88 1.0000
18 -7 7 44 236
11 -0.65 2.88 1.0000
-20 7 -7 69 163

156 -3.25 -1.87 1.0000
20 -7 7 347 286
522 -3.25 1.87 1.0000
-22 7 -7 2 184
-37 0.32 -1.22 1.0000
22 -7 7 2 303
-196 0.32 1.22 1.0000
23 6 -7 9 364
-159 -1.57 -1.99 1.0000
23 6 -7 101 414
168 -1.57 -1.99 1.0000
21 6 -7 0 326
-316 -0.31 -0.27 1.0000
21 6 -7 367 382
355 -0.31 -0.27 1.0000
19 6 -7 39 316
-81 -4.49 -3.04 1.0000
19 6 -7 323 321
142 -4.49 -3.04 1.0000
17 6 -7 87 281
161 -0.83 -4.16 1.0000
17 6 -7 87 294
161 -0.83 -4.16 1.0000
15 6 -7 1701 302
1361 0.90 4.32 1.0000
15 6 -7 1267 298
975 0.90 4.32 1.0000
13 6 -7 948 288
1137 -4.05 3.25 1.0000
13 6 -7 832 244
1008 -4.05 3.25 1.0000
11 6 -7 2462 334
4491 6.29 15.55 1.0000
11 6 -7 2667 425
4769 6.29 15.55 1.0000
9 6 -7 188 229
716 -0.99 21.59 1.0000
9 6 -7 235 173
907 -0.99 21.59 1.0000
7 6 -7 805 179
1118 -3.08 13.01 1.0000
7 6 -7 868 232
1201 -3.08 13.01 1.0000
-7 -6 7 936 175

1309	-3.08	-13.01	1.0000	
5	6	-7	867	304
2225	-7.57	14.16	1.0000	
-5	-6	7	748	193
2032	-7.57	-14.16	1.0000	
3	6	-7	1720	283
2854	-13.11	-0.61	1.0000	
1	6	-7	674	113
720	-3.76	7.98	1.0000	
1	6	-7	526	178
554	-3.76	7.98	1.0000	
-1	6	-7	1327	227
1186	-9.10	7.39	1.0000	
-1	6	-7	1582	142
1445	-9.10	7.39	1.0000	
1	-6	7	1830	140
1685	-9.10	-7.39	1.0000	
1	-6	7	1490	168
1341	-9.10	-7.39	1.0000	
-3	6	-7	6683	399
7430	3.32	2.14	1.0000	
-3	6	-7	6245	380
6968	3.32	2.14	1.0000	
-3	6	-7	6821	352
7575	3.32	2.14	1.0000	
3	-6	7	6837	355
7592	3.32	-2.14	1.0000	
-5	6	-7	1549	118
562	-2.67	18.79	1.0000	
-5	6	-7	1311	147
402	-2.67	18.79	1.0000	
-5	6	-7	1001	116
211	-2.67	18.79	1.0000	
5	-6	7	1540	123
576	-2.67	-18.79	1.0000	
-7	6	-7	1788	136
804	14.16	8.56	1.0000	
-7	6	-7	1479	153
586	14.16	8.56	1.0000	
7	-6	7	1648	143
697	14.16	-8.56	1.0000	
-9	6	-7	1066	169
1233	0.93	1.98	1.0000	
-9	6	-7	941	200

1098	0.93	1.98	1.0000	
9	-6	7	1069	186
1236	0.93	-1.98	1.0000	
-11	6	-7	349	138
360	0.29	-0.18	1.0000	
-11	6	-7	404	124
415	0.29	-0.18	1.0000	
11	-6	7	112	152
118	0.29	0.18	1.0000	
-13	6	-7	673	164
834	-0.07	-7.60	1.0000	
13	-6	7	644	207
798	-0.07	7.60	1.0000	
-15	6	-7	242	133
38	-0.52	-8.31	1.0000	
15	-6	7	674	206
278	-0.52	8.31	1.0000	
-17	6	-7	508	171
561	1.24	1.38	1.0000	
17	-6	7	639	259
699	1.24	-1.38	1.0000	
-19	6	-7	11	139
-14	1.32	-2.62	1.0000	
19	-6	7	220	262
316	1.32	2.62	1.0000	
-21	6	-7	210	171
311	3.81	2.78	1.0000	
21	-6	7	17	269
6	3.81	-2.78	1.0000	
-23	6	-7	6	180
-44	-0.31	2.17	1.0000	
23	-6	7	210	325
208	-0.31	-2.17	1.0000	
22	5	-7	26	361
-16	2.38	-3.74	1.0000	
22	5	-7	26	368
-317	2.38	-3.74	1.0000	
20	5	-7	21	300
-203	1.56	3.67	1.0000	
20	5	-7	21	322
-466	1.56	3.67	1.0000	
18	5	-7	12	309
-116	3.04	-0.29	1.0000	
18	5	-7	12	290

-44	3.04	-0.29	1.0000	
16	5 -7	1707	338	
1947	3.58	-3.50	1.0000	
16	5 -7	2219	362	
2495	3.58	-3.50	1.0000	
14	5 -7	1507	273	
1299	-1.16	2.50	1.0000	
14	5 -7	930	283	
768	-1.16	2.50	1.0000	
12	5 -7	1906	236	
1263	5.84	-8.66	1.0000	
12	5 -7	1729	268	
1116	5.84	-8.66	1.0000	
10	5 -7	526	242	
1873	-6.05	-22.59	1.0000	
10	5 -7	595	334	
2024	-6.05	-22.59	1.0000	
8	5 -7	1598	215	
722	7.64	-8.45	1.0000	
8	5 -7	1732	152	
813	7.64	-8.45	1.0000	
-8	-5 7	1346	145	
557	7.64	8.45	1.0000	
6	5 -7	1661	203	
666	-2.75	-18.79	1.0000	
4	5 -7	2101	195	
767	14.88	-5.38	1.0000	
2	5 -7	3642	289	
2687	9.89	11.18	1.0000	
0	5 -7	7616	275	
5719	4.42	-13.64	1.0000	
0	5 -7	7212	354	
5366	4.42	-13.64	1.0000	
-2	5 -7	1402	241	
1781	3.78	2.87	1.0000	
-2	5 -7	1698	157	
2114	3.78	2.87	1.0000	
2	-5 7	1563	145	
1964	3.78	-2.87	1.0000	
-4	5 -7	1309	218	
1949	-4.43	-6.11	1.0000	
-4	5 -7	1123	192	
1720	-4.43	-6.11	1.0000	
-4	5 -7	1565	167	

2260	-4.43	-6.11	1.0000	
4	-5	7	1475	165
2153	-4.43	6.11	1.0000	
-6	5	-7	940	172
2363	-7.48	-14.35	1.0000	
-6	5	-7	755	235
2061	-7.48	-14.35	1.0000	
6	-5	7	1080	197
2581	-7.48	14.35	1.0000	
-8	5	-7	2208	205
1477	-8.03	-3.63	1.0000	
-8	5	-7	2436	172
1666	-8.03	-3.63	1.0000	
8	-5	7	2200	192
1469	-8.03	3.63	1.0000	
-10	5	-7	1289	194
1477	-0.60	-2.15	1.0000	
-10	5	-7	1001	166
1168	-0.60	-2.15	1.0000	
10	-5	7	1193	216
1374	-0.60	2.15	1.0000	
-12	5	-7	1180	162
940	-1.01	3.94	1.0000	
12	-5	7	1075	196
845	-1.01	-3.94	1.0000	
-14	5	-7	5	129
144	3.07	11.62	1.0000	
14	-5	7	2	203
192	3.07	-11.62	1.0000	
-16	5	-7	171	148
264	-0.55	2.90	1.0000	
16	-5	7	461	234
609	-0.55	-2.90	1.0000	
-18	5	-7	111	150
74	0.20	2.72	1.0000	
18	-5	7	10	217
-373	0.20	-2.72	1.0000	
-20	5	-7	177	154
121	-3.45	-1.31	1.0000	
20	-5	7	89	256
47	-3.45	1.31	1.0000	
-22	5	-7	58	170
35	-1.13	-1.99	1.0000	
22	-5	7	27	283

11	-1.13	1.99	1.0000	
23	4	-7	3	387
-575	0.89	1.05	1.0000	
23	4	-7	142	403
129	0.89	1.05	1.0000	
21	4	-7	99	353
237	-4.48	-1.45	1.0000	
19	4	-7	24	306
15	-1.19	-2.10	1.0000	
19	4	-7	8	302
-76	-1.19	-2.10	1.0000	
17	4	-7	65	266
236	-5.41	5.36	1.0000	
17	4	-7	77	273
-136	-5.41	5.36	1.0000	
15	4	-7	1824	348
2219	-1.82	-3.81	1.0000	
15	4	-7	1344	329
1685	-1.82	-3.81	1.0000	
13	4	-7	113	245
492	4.64	8.87	1.0000	
13	4	-7	128	223
522	4.64	8.87	1.0000	
11	4	-7	125	228
797	-4.53	17.69	1.0000	
11	4	-7	229	197
1090	-4.53	17.69	1.0000	
9	4	-7	2447	339
3623	5.16	12.12	1.0000	
9	4	-7	1720	241
2704	5.16	12.12	1.0000	
-9	-4	7	1670	237
2641	5.16	-12.12	1.0000	
7	4	-7	2215	359
4678	-1.72	23.35	1.0000	
-7	-4	7	2061	254
4452	-1.72	-23.35	1.0000	
5	4	-7	1642	287
2354	-4.30	6.14	1.0000	
3	4	-7	3871	324
4216	-4.34	-4.75	1.0000	
1	4	-7	3758	330
4345	-16.43	-6.85	1.0000	
-1	4	-7	824	205

```
1428     9.13   -1.98  1.0000
  -1   4  -7      653      132
1196     9.13   -1.98  1.0000
  -3   4  -7      725      161
576   -1.92   -3.03  1.0000
  -3   4  -7      988      122
814   -1.92   -3.03  1.0000
  -3   4  -7     1137      129
950   -1.92   -3.03  1.0000
   3  -4   7      827      114
667   -1.92    3.03  1.0000
  -5   4  -7     2255      134
858   14.91    5.52  1.0000
  -5   4  -7     2265      157
864   14.91    5.52  1.0000
  -5   4  -7     2447      137
978   14.91    5.52  1.0000
   5  -4   7     2327      139
902   14.91   -5.52  1.0000
  -7   4  -7      455      119
556    4.33    2.04  1.0000
  -7   4  -7      527      152
637    4.33    2.04  1.0000
   7  -4   7      692      139
819    4.33   -2.04  1.0000
  -9   4  -7     1011      196
1677     9.04    5.87  1.0000
  -9   4  -7      798      169
1390     9.04    5.87  1.0000
   9  -4   7      821      201
1413     9.04   -5.87  1.0000
 -11   4  -7     1044      194
1896     5.26   -8.70  1.0000
  11  -4   7     1101      248
1971     5.26    8.70  1.0000
 -13   4  -7      359      178
1065    -2.89  -11.51  1.0000
  13  -4   7      488      236
1281    -2.89   11.51  1.0000
 -15   4  -7      762      176
1120     4.57   -2.35  1.0000
  15  -4   7      793      239
1157     4.57    2.35  1.0000
 -17   4  -7      188      169
```

609 -1.82 -10.85 1.0000
17 -4 7 88 231
399 -1.82 10.85 1.0000
-19 4 -7 452 158
263 0.75 5.09 1.0000
19 -4 7 252 235
115 0.75 -5.09 1.0000
-21 4 -7 376 188
462 3.09 -0.43 1.0000
21 -4 7 101 273
143 3.09 0.43 1.0000
-23 4 -7 74 188
46 1.62 0.37 1.0000
23 -4 7 4 305
-237 1.62 -0.37 1.0000
22 3 -7 11 347
12 0.36 0.06 1.0000
22 3 -7 0 357
-488 0.36 0.06 1.0000
20 3 -7 143 337
95 1.76 -4.20 1.0000
20 3 -7 567 320
493 1.76 -4.20 1.0000
18 3 -7 56 280
-184 6.29 1.53 1.0000
18 3 -7 6 273
35 6.29 1.53 1.0000
16 3 -7 620 277
701 -1.32 1.53 1.0000
16 3 -7 708 275
794 -1.32 1.53 1.0000
14 3 -7 3004 425
4456 10.40 -4.59 1.0000
14 3 -7 3044 379
4505 10.40 -4.59 1.0000
12 3 -7 579 207
536 -5.56 -7.09 1.0000
12 3 -7 460 194
408 -5.56 -7.09 1.0000
10 3 -7 2046 310
2470 5.24 -7.12 1.0000
10 3 -7 2000 251
2419 5.24 -7.12 1.0000
8 3 -7 2964 267

```
1372    -5.20  -25.89  1.0000
   8    3   -7     2461      173
974    -5.20  -25.89  1.0000
  -8   -3    7     2347      151
902    -5.20   25.89  1.0000
   6    3   -7     6503      419
7239     3.38   -1.97  1.0000
   4    3   -7     1424      241
1222    -2.04    2.97  1.0000
   2    3   -7     3996      264
2388    13.09    0.86  1.0000
   0    3   -7     5603      579
12117     6.77   30.20  1.0000
  -2    3   -7     2209      189
3046    -7.23   -0.96  1.0000
  -2    3   -7     2127      272
2950    -7.23   -0.96  1.0000
  -4    3   -7     3121      210
3433    -4.40    4.68  1.0000
  -4    3   -7     3146      273
3459    -4.40    4.68  1.0000
  -4    3   -7     3547      218
3882    -4.40    4.68  1.0000
   4   -3    7     3341      208
3671    -4.40   -4.68  1.0000
  -6    3   -7     1041      175
1911   -13.03    0.74  1.0000
  -6    3   -7      728      153
1460   -13.03    0.74  1.0000
  -6    3   -7      590      203
1251   -13.03    0.74  1.0000
   6   -3    7      997      170
1858   -13.03   -0.74  1.0000
  -8    3   -7     2264      151
1068    -6.10  -11.67  1.0000
  -8    3   -7     2513      168
1242    -6.10  -11.67  1.0000
   8   -3    7     2429      180
1183    -6.10   11.67  1.0000
 -10    3   -7      410      125
413    -4.47    5.73  1.0000
  10   -3    7      202      143
178    -4.47   -5.73  1.0000
 -12    3   -7     1118      143
```

518	1.95	9.04	1.0000	
12	-3	7	801	165
311	1.95	-9.04	1.0000	
-14	3	-7	704	167
464	-1.44	5.94	1.0000	
14	-3	7	611	199
385	-1.44	-5.94	1.0000	
-16	3	-7	71	157
385	3.59	9.04	1.0000	
16	-3	7	141	221
534	3.59	-9.04	1.0000	
-18	3	-7	716	174
743	-2.72	-1.16	1.0000	
18	-3	7	768	267
797	-2.72	1.16	1.0000	
-20	3	-7	187	174
248	2.09	-0.05	1.0000	
20	-3	7	85	256
126	2.09	0.05	1.0000	
-22	3	-7	151	179
131	-4.56	-0.94	1.0000	
22	-3	7	246	307
229	-4.56	0.94	1.0000	
23	2	-7	160	376
237	-2.36	0.72	1.0000	
21	2	-7	455	327
473	-0.43	0.49	1.0000	
21	2	-7	1	334
-256	-0.43	0.49	1.0000	
19	2	-7	118	289
74	-4.35	4.39	1.0000	
19	2	-7	253	300
215	-4.35	4.39	1.0000	
17	2	-7	20	248
-16	0.75	-3.76	1.0000	
17	2	-7	171	253
97	0.75	-3.76	1.0000	
15	2	-7	180	231
348	-5.29	2.01	1.0000	
15	2	-7	75	230
187	-5.29	2.01	1.0000	
13	2	-7	1598	296
2315	-2.01	6.96	1.0000	
13	2	-7	1978	331

2768	-2.01	6.96	1.0000	
11	2 -7	1284	280	
1637	8.60	-0.26	1.0000	
11	2 -7	1060	227	
1376	8.60	-0.26	1.0000	
9	2 -7	577	197	
1507	-0.81	24.51	1.0000	
9	2 -7	832	276	
2051	-0.81	24.51	1.0000	
-9	-2 7	620	177	
1572	-0.81	-24.51	1.0000	
7	2 -7	1098	244	
1781	6.79	6.58	1.0000	
5	2 -7	1920	250	
2356	3.71	-2.84	1.0000	
3	2 -7	2324	272	
3180	-7.21	0.95	1.0000	
-1	2 -7	1635	263	
2896	-9.26	-26.83	1.0000	
-3	2 -7	3645	163	
2100	13.29	-0.80	1.0000	
-3	2 -7	3414	239	
1925	13.29	-0.80	1.0000	
-5	2 -7	3625	183	
2685	9.84	-11.13	1.0000	
-5	2 -7	3841	246	
2878	9.84	-11.13	1.0000	
-5	2 -7	3208	176	
2314	9.84	-11.13	1.0000	
5	-2 7	3432	184	
2503	9.84	11.13	1.0000	
-7	2 -7	3597	309	
5894	12.54	10.27	1.0000	
-7	2 -7	3640	330	
5949	12.54	10.27	1.0000	
7	-2 7	3859	326	
6221	12.54	-10.27	1.0000	
-9	2 -7	1047	142	
750	7.13	-0.27	1.0000	
-9	2 -7	1093	143	
790	7.13	-0.27	1.0000	
9	-2 7	988	140	
703	7.13	0.27	1.0000	
9	-2 7	994	160	

708 7.13 0.27 1.0000
-11 2 -7 368 124
260 2.05 -10.33 1.0000
11 -2 7 272 156
150 2.05 10.33 1.0000
-13 2 -7 613 170
883 6.47 -2.28 1.0000
13 -2 7 378 208
585 6.47 2.28 1.0000
-15 2 -7 520 198
1290 -4.99 -14.87 1.0000
15 -2 7 462 252
1188 -4.99 14.87 1.0000
-17 2 -7 43 138
11 1.54 5.39 1.0000
17 -2 7 42 226
-143 1.54 -5.39 1.0000
-19 2 -7 147 157
103 2.25 -2.44 1.0000
19 -2 7 15 249
-7 2.25 2.44 1.0000
-21 2 -7 281 172
271 -1.58 0.93 1.0000
21 -2 7 236 277
227 -1.58 -0.93 1.0000
-23 2 -7 148 187
83 1.41 2.30 1.0000
23 -2 7 393 317
281 1.41 -2.30 1.0000
22 1 -7 134 355
108 -1.44 -2.19 1.0000
20 1 -7 27 293
-378 4.37 -1.05 1.0000
20 1 -7 318 310
406 4.37 -1.05 1.0000
18 1 -7 95 253
95 -4.27 -2.29 1.0000
18 1 -7 426 272
464 -4.27 -2.29 1.0000
16 1 -7 1092 248
615 11.16 4.71 1.0000
16 1 -7 1287 268
775 11.16 4.71 1.0000
14 1 -7 516 220

607 -0.79 1.52 1.0000
14 1 -7 626 223
726 -0.79 1.52 1.0000
12 1 -7 457 197
476 5.82 -0.71 1.0000
12 1 -7 500 186
522 5.82 -0.71 1.0000
10 1 -7 725 256
1575 -5.87 -9.58 1.0000
10 1 -7 755 212
1618 -5.87 -9.58 1.0000
-10 -1 7 479 184
1201 -5.87 9.58 1.0000
8 1 -7 365 154
173 -4.20 -3.57 1.0000
6 1 -7 1902 219
1751 -9.10 -7.32 1.0000
4 1 -7 765 218
1377 9.32 2.10 1.0000
2 1 -7 1525 245
2796 -9.22 26.93 1.0000
0 1 -7 6855 504
10472 2.38 22.35 1.0000
-2 1 -7 5604 221
1774 7.82 27.32 1.0000
-4 1 -7 2930 214
3391 -16.41 7.01 1.0000
-4 1 -7 3185 270
3684 -16.41 7.01 1.0000
-6 1 -7 619 120
675 -3.59 -8.04 1.0000
-6 1 -7 587 117
639 -3.59 -8.04 1.0000
-6 1 -7 486 147
523 -3.59 -8.04 1.0000
6 -1 7 580 125
625 -3.59 8.04 1.0000
-8 1 -7 2650 203
2269 -10.16 0.37 1.0000
-8 1 -7 2602 185
2224 -10.16 0.37 1.0000
8 -1 7 2550 212
2179 -10.16 -0.37 1.0000
8 -1 7 2581 202

2209 -10.16 -0.37 1.0000
-10 1 -7 614 140
801 -3.13 -0.07 1.0000
10 -1 7 819 185
1032 -3.13 0.07 1.0000
-12 1 -7 663 167
936 -0.86 4.30 1.0000
12 -1 7 814 206
1115 -0.86 -4.30 1.0000
-14 1 -7 1246 208
1845 7.11 9.14 1.0000
14 -1 7 892 264
1397 7.11 -9.14 1.0000
-16 1 -7 53 153
80 -1.88 0.59 1.0000
16 -1 7 119 226
159 -1.88 -0.59 1.0000
-18 1 -7 312 148
116 5.20 3.28 1.0000
18 -1 7 411 241
179 5.20 -3.28 1.0000
-20 1 -7 25 168
118 -5.86 -2.69 1.0000
20 -1 7 33 262
137 -5.86 2.69 1.0000
-22 1 -7 65 167
149 3.50 -1.87 1.0000
22 -1 7 21 296
-16 3.50 1.87 1.0000
23 0 -7 1 368
-346 -0.73 -0.53 1.0000
23 0 -7 821 383
846 -0.73 -0.53 1.0000
21 0 -7 60 303
164 0.77 4.89 1.0000
21 0 -7 564 326
841 0.77 4.89 1.0000
19 0 -7 26 260
21 0.02 1.35 1.0000
19 0 -7 192 278
183 0.02 1.35 1.0000
17 0 -7 146 238
331 -3.85 -3.66 1.0000
17 0 -7 152 242

340 -3.85 -3.66 1.0000
15 0 -7 184 221
249 1.33 1.69 1.0000
15 0 -7 170 198
232 1.33 1.69 1.0000
13 0 -7 1035 192
828 -2.33 -10.79 1.0000
13 0 -7 951 212
747 -2.33 -10.79 1.0000
11 0 -7 934 173
337 2.82 10.36 1.0000
11 0 -7 897 149
315 2.82 10.36 1.0000
9 0 -7 313 133
545 12.23 -4.26 1.0000
9 0 -7 270 164
468 12.23 -4.26 1.0000
-9 0 7 296 126
517 12.23 4.26 1.0000
7 0 -7 1607 220
1781 8.46 3.72 1.0000
5 0 -7 7540 340
5652 4.28 13.62 1.0000
3 0 -7 6099 598
12829 6.58 -30.16 1.0000
1 0 -7 7404 528
11152 2.32 -22.50 1.0000
-1 0 -7 6662 491
10254 2.63 -22.40 1.0000
-3 0 -7 6307 565
13116 6.82 -30.08 1.0000
-3 0 -7 6158 588
12901 6.82 -30.08 1.0000
-5 0 -7 7307 289
5470 4.39 13.54 1.0000
-5 0 -7 7514 278
5651 4.39 13.54 1.0000
-5 0 -7 7468 319
5611 4.39 13.54 1.0000
5 0 7 7390 289
5534 4.39 -13.54 1.0000
-7 0 -7 1561 207
1704 8.22 3.88 1.0000
-7 0 -7 1484 177

1621	8.22	3.88	1.0000	
-7	0	-7	1460	157
1595	8.22	3.88	1.0000	
7	0	7	1262	179
1379	8.22	-3.88	1.0000	
7	0	7	1620	181
1767	8.22	-3.88	1.0000	
-9	0	-7	310	126
551	12.06	-4.57	1.0000	
9	0	7	227	151
401	12.06	4.57	1.0000	
-11	0	-7	765	126
226	2.83	10.72	1.0000	
11	0	7	791	162
240	2.83	-10.72	1.0000	
-13	0	-7	759	151
567	-2.53	-10.95	1.0000	
13	0	7	875	216
667	-2.53	10.95	1.0000	
-15	0	-7	134	145
192	1.33	1.76	1.0000	
15	0	7	97	211
146	1.33	-1.76	1.0000	
-17	0	-7	146	163
338	-4.11	-3.66	1.0000	
17	0	7	245	249
483	-4.11	3.66	1.0000	
-19	0	-7	290	166
266	0.40	1.22	1.0000	
19	0	7	175	253
156	0.40	-1.22	1.0000	
-21	0	-7	596	208
873	0.65	4.84	1.0000	
21	0	7	433	298
673	0.65	-4.84	1.0000	
-23	0	-7	450	206
459	-0.86	-0.36	1.0000	
23	0	7	170	317
175	-0.86	0.36	1.0000	
22	-1	-7	71	345
156	3.39	-2.06	1.0000	
22	-1	-7	21	306
-546	3.39	-2.06	1.0000	
20	-1	-7	91	284

250	-5.81	-2.55	1.0000	
20	-1	-7	54	272
-139	-5.81	-2.55	1.0000	
18	-1	-7	445	254
196	5.43	3.16	1.0000	
18	-1	-7	290	239
98	5.43	3.16	1.0000	
16	-1	-7	181	212
229	-1.84	0.52	1.0000	
16	-1	-7	281	242
341	-1.84	0.52	1.0000	
14	-1	-7	1380	304
2000	6.97	9.25	1.0000	
14	-1	-7	996	275
1512	6.97	9.25	1.0000	
12	-1	-7	572	180
841	-0.87	4.50	1.0000	
12	-1	-7	717	210
1015	-0.87	4.50	1.0000	
10	-1	-7	399	170
542	-2.96	-0.14	1.0000	
10	-1	-7	375	143
514	-2.96	-0.14	1.0000	
-10	1	7	188	119
289	-2.96	0.14	1.0000	
8	-1	-7	2593	244
2218	-10.51	0.50	1.0000	
6	-1	-7	471	143
503	-3.64	-8.04	1.0000	
4	-1	-7	2820	256
3274	-16.39	6.88	1.0000	
2	-1	-7	5445	200
1683	8.09	27.24	1.0000	
0	-1	-7	7355	525
11166	2.52	22.59	1.0000	
-2	-1	-7	1911	245
3462	-9.48	26.86	1.0000	
-4	-1	-7	705	148
1278	9.08	1.97	1.0000	
-4	-1	-7	1072	190
1766	9.08	1.97	1.0000	
-6	-1	-7	1605	158
1438	-9.20	-7.20	1.0000	
-6	-1	-7	1410	145

```
1242    -9.20    -7.20  1.0000
  -6   -1   -7     1826       194
1660    -9.20    -7.20  1.0000
   6    1    7     1375       178
1217    -9.20     7.20  1.0000
   6    1    7     1649       175
1494    -9.20     7.20  1.0000
  -8   -1   -7      169       102
49    -4.20    -3.52  1.0000
   8    1    7      232       131
87    -4.20     3.52  1.0000
 -10   -1   -7      244       145
813    -5.86    -9.80  1.0000
  10    1    7      277       181
874    -5.86     9.80  1.0000
 -12   -1   -7      397       145
406     5.95    -0.92  1.0000
  12    1    7      396       177
404     5.95     0.92  1.0000
 -14   -1   -7      228       156
298    -0.94     1.66  1.0000
  14    1    7      120       207
172    -0.94    -1.66  1.0000
 -16   -1   -7     1018       174
543    11.30     4.54  1.0000
  16    1    7      876       244
436    11.30    -4.54  1.0000
 -18   -1   -7      417       163
451    -4.32    -2.45  1.0000
  18    1    7      535       259
575    -4.32     2.45  1.0000
 -20   -1   -7      255       173
331     4.19    -0.82  1.0000
  20    1    7      188       272
248     4.19     0.82  1.0000
 -22   -1   -7       10       182
-105    -1.42    -2.29  1.0000
  22    1    7       10       329
-53    -1.42     2.29  1.0000
  23   -2   -7       10       340
-62     1.44     2.31  1.0000
  21   -2   -7        4       282
-63    -1.57     0.91  1.0000
  21   -2   -7      265       302
```

255	-1.57	0.91	1.0000	
19	-2	-7	193	251
137	2.50	-2.45	1.0000	
19	-2	-7	16	263
-52	2.50	-2.45	1.0000	
17	-2	-7	138	210
144	1.59	5.59	1.0000	
17	-2	-7	45	215
-11	1.59	5.59	1.0000	
15	-2	-7	685	300
1568	-5.01	-14.88	1.0000	
15	-2	-7	593	239
1415	-5.01	-14.88	1.0000	
13	-2	-7	530	184
787	6.57	-2.24	1.0000	
13	-2	-7	513	206
765	6.57	-2.24	1.0000	
11	-2	-7	448	141
351	2.20	-10.23	1.0000	
11	-2	-7	328	162
221	2.20	-10.23	1.0000	
9	-2	-7	910	168
622	7.34	-0.34	1.0000	
9	-2	-7	893	131
608	7.34	-0.34	1.0000	
-9	2	7	820	134
550	7.34	0.34	1.0000	
7	-2	-7	3514	348
5810	12.58	10.39	1.0000	
5	-2	-7	3388	227
2468	9.91	-11.23	1.0000	
3	-2	-7	3472	205
1980	13.19	-0.84	1.0000	
1	-2	-7	1640	229
2926	-9.42	-26.89	1.0000	
-1	-2	-7	5428	189
1677	7.93	-27.27	1.0000	
-3	-2	-7	2255	227
3092	-7.15	0.95	1.0000	
-5	-2	-7	1839	195
2274	3.91	-2.82	1.0000	
-5	-2	-7	1644	166
2057	3.91	-2.82	1.0000	
-5	-2	-7	1628	166

2039	3.91	-2.82	1.0000	
5	2	7	1299	179
1666	3.91	2.82	1.0000	
-7	-2	-7	839	182
1456	6.96	6.60	1.0000	
-7	-2	-7	800	151
1404	6.96	6.60	1.0000	
7	2	7	1003	191
1667	6.96	-6.60	1.0000	
-9	-2	-7	517	178
1356	-0.65	24.61	1.0000	
9	2	7	528	196
1349	-0.65	-24.61	1.0000	
-11	-2	-7	1024	169
1332	8.35	-0.39	1.0000	
11	2	7	1284	231
1630	8.35	0.39	1.0000	
-13	-2	-7	1202	206
1852	-2.00	7.19	1.0000	
13	2	7	1268	314
1934	-2.00	-7.19	1.0000	
-15	-2	-7	210	167
395	-5.36	1.85	1.0000	
15	2	7	268	247
475	-5.36	-1.85	1.0000	
-17	-2	-7	19	164
-43	0.93	-3.66	1.0000	
17	2	7	174	261
95	0.93	3.66	1.0000	
-19	-2	-7	397	174
359	-4.60	4.46	1.0000	
19	2	7	118	267
72	-4.60	-4.46	1.0000	
-21	-2	-7	294	191
305	-0.37	0.38	1.0000	
21	2	7	220	295
229	-0.37	-0.38	1.0000	
-23	-2	-7	8	188
-110	-2.30	0.77	1.0000	
23	2	7	189	351
271	-2.30	-0.77	1.0000	
22	-3	-7	28	288
-344	-4.50	-0.90	1.0000	
22	-3	-7	473	344

463	-4.50	-0.90	1.0000	
20	-3	-7	6	263
-37	2.12	-0.10	1.0000	
20	-3	-7	75	251
116	2.12	-0.10	1.0000	
18	-3	-7	775	273
801	-2.99	-1.18	1.0000	
18	-3	-7	770	258
796	-2.99	-1.18	1.0000	
16	-3	-7	115	212
488	3.70	9.15	1.0000	
16	-3	-7	15	216
232	3.70	9.15	1.0000	
14	-3	-7	492	181
298	-1.25	5.96	1.0000	
14	-3	-7	534	189
332	-1.25	5.96	1.0000	
12	-3	-7	782	172
302	1.87	8.98	1.0000	
12	-3	-7	1045	162
473	1.87	8.98	1.0000	
10	-3	-7	168	148
149	-4.90	5.74	1.0000	
10	-3	-7	481	140
511	-4.90	5.74	1.0000	
8	-3	-7	2435	153
1200	-5.99	-11.53	1.0000	
8	-3	-7	2366	220
1152	-5.99	-11.53	1.0000	
-8	3	7	2658	161
1359	-5.99	11.53	1.0000	
6	-3	-7	959	216
1795	-12.96	0.63	1.0000	
4	-3	-7	3586	277
3930	-4.46	4.72	1.0000	
2	-3	-7	2343	246
3191	-7.13	-1.00	1.0000	
0	-3	-7	6399	605
13237	6.63	30.06	1.0000	
-2	-3	-7	3542	193
2012	13.37	0.89	1.0000	
-4	-3	-7	898	128
719	-2.01	3.26	1.0000	
-4	-3	-7	832	111

660 -2.01 3.26 1.0000
4 3 7 1095 130
898 -2.01 -3.26 1.0000
-6 -3 -7 6278 370
6997 3.22 -2.32 1.0000
-6 -3 -7 6992 367
7749 3.22 -2.32 1.0000
-6 -3 -7 6820 362
7568 3.22 -2.32 1.0000
6 3 7 6607 376
7344 3.22 2.32 1.0000
-8 -3 -7 2657 157
1092 -5.66 -25.85 1.0000
8 3 7 2687 177
1134 -5.66 25.85 1.0000
-10 -3 -7 1727 192
2108 5.37 -7.13 1.0000
10 3 7 1912 252
2322 5.37 7.13 1.0000
-12 -3 -7 441 144
388 -5.51 -7.03 1.0000
12 3 7 562 189
524 -5.51 7.03 1.0000
-14 -3 -7 2134 266
3385 10.47 -4.87 1.0000
14 3 7 2586 380
3956 10.47 4.87 1.0000
-16 -3 -7 841 192
950 -1.52 1.64 1.0000
16 3 7 642 274
736 -1.52 -1.64 1.0000
-18 -3 -7 55 157
-46 6.25 1.50 1.0000
18 3 7 102 275
290 6.25 -1.50 1.0000
-20 -3 -7 335 179
279 1.98 -4.26 1.0000
20 3 7 362 290
303 1.98 4.26 1.0000
-22 -3 -7 0 189
-38 0.39 0.10 1.0000
22 3 7 0 319
-87 0.39 -0.10 1.0000
23 -4 -7 116 347

80	1.62	0.31	1.0000	
23	-4	-7	4	309
-518	1.62	0.31	1.0000	
21	-4	-7	148	274
202	3.10	-0.35	1.0000	
21	-4	-7	338	314
422	3.10	-0.35	1.0000	
19	-4	-7	285	254
141	0.81	5.01	1.0000	
19	-4	-7	309	238
158	0.81	5.01	1.0000	
17	-4	-7	224	238
677	-1.69	-10.83	1.0000	
17	-4	-7	306	243
820	-1.69	-10.83	1.0000	
15	-4	-7	776	234
1136	4.61	-2.27	1.0000	
15	-4	-7	577	218
892	4.61	-2.27	1.0000	
13	-4	-7	281	211
929	-2.91	-11.53	1.0000	
13	-4	-7	510	232
1317	-2.91	-11.53	1.0000	
11	-4	-7	1218	272
2100	5.32	-8.40	1.0000	
11	-4	-7	1454	227
2407	5.32	-8.40	1.0000	
9	-4	-7	901	180
1552	9.27	5.78	1.0000	
9	-4	-7	755	230
1352	9.27	5.78	1.0000	
7	-4	-7	350	145
441	4.38	1.93	1.0000	
7	-4	-7	543	114
662	4.38	1.93	1.0000	
-7	4	7	593	142
716	4.38	-1.93	1.0000	
5	-4	-7	2204	159
829	14.86	5.57	1.0000	
3	-4	-7	715	134
562	-2.12	-3.17	1.0000	
1	-4	-7	914	188
1558	9.24	-1.99	1.0000	
-1	-4	-7	2737	236

3161	-16.42	-7.03	1.0000	
-3	-4	-7	3241	217
3585	-4.53	-4.62	1.0000	
-3	-4	-7	3342	242
3691	-4.53	-4.62	1.0000	
3	4	7	3187	230
3522	-4.53	4.62	1.0000	
-5	-4	-7	1996	189
2790	-4.42	6.23	1.0000	
-5	-4	-7	2080	207
2890	-4.42	6.23	1.0000	
-5	-4	-7	1800	210
2557	-4.42	6.23	1.0000	
5	4	7	1853	215
2619	-4.42	-6.23	1.0000	
-7	-4	-7	2331	273
4869	-1.52	23.36	1.0000	
-7	-4	-7	2432	272
5020	-1.52	23.36	1.0000	
7	4	7	2227	301
4722	-1.52	-23.36	1.0000	
-9	-4	-7	1768	218
2764	5.27	12.15	1.0000	
9	4	7	2093	263
3179	5.27	-12.15	1.0000	
-11	-4	-7	157	152
895	-4.53	17.68	1.0000	
11	4	7	204	208
1025	-4.53	-17.68	1.0000	
-13	-4	-7	169	161
601	4.65	8.85	1.0000	
13	4	7	298	237
829	4.65	-8.85	1.0000	
-15	-4	-7	1709	227
2078	-1.81	-3.69	1.0000	
15	4	7	1301	290
1625	-1.81	3.69	1.0000	
-17	-4	-7	75	159
-24	-5.30	5.28	1.0000	
17	4	7	35	252
166	-5.30	-5.28	1.0000	
-19	-4	-7	164	171
153	-1.17	-2.11	1.0000	
19	4	7	399	276

386	-1.17	2.11	1.0000	
-21	-4	-7	116	191
262	-4.51	-1.37	1.0000	
21	4	7	169	309
340	-4.51	1.37	1.0000	
-23	-4	-7	366	228
350	0.81	1.04	1.0000	
23	4	7	2	311
-49	0.81	-1.04	1.0000	
22	-5	-7	7	289
-109	-1.06	-2.11	1.0000	
22	-5	-7	7	297
-148	-1.06	-2.11	1.0000	
20	-5	-7	175	267
119	-3.35	-1.16	1.0000	
20	-5	-7	17	249
-53	-3.35	-1.16	1.0000	
18	-5	-7	54	218
28	0.28	2.77	1.0000	
18	-5	-7	239	243
188	0.28	2.77	1.0000	
16	-5	-7	153	211
239	-0.48	2.87	1.0000	
16	-5	-7	107	195
181	-0.48	2.87	1.0000	
14	-5	-7	2	184
197	2.87	11.60	1.0000	
14	-5	-7	2	166
199	2.87	11.60	1.0000	
12	-5	-7	1007	195
778	-0.94	4.04	1.0000	
12	-5	-7	1034	173
802	-0.94	4.04	1.0000	
10	-5	-7	1056	193
1227	-0.38	-2.17	1.0000	
10	-5	-7	1123	238
1299	-0.38	-2.17	1.0000	
8	-5	-7	2502	180
1715	-8.27	-3.57	1.0000	
8	-5	-7	2835	238
1994	-8.27	-3.57	1.0000	
-8	5	7	2312	230
1555	-8.27	3.57	1.0000	
6	-5	-7	752	227

2048	-7.54	-14.26	1.0000	
6	-5	-7	754	165
2052	-7.54	-14.26	1.0000	
-6	5	7	608	209
1799	-7.54	14.26	1.0000	
4	-5	-7	1699	226
2435	-4.26	-6.28	1.0000	
2	-5	-7	1607	212
2009	3.82	2.82	1.0000	
0	-5	-7	7561	326
5684	4.27	-13.47	1.0000	
-2	-5	-7	4149	228
3135	9.78	11.17	1.0000	
-2	-5	-7	3494	207
2551	9.78	11.17	1.0000	
2	5	7	4163	207
3159	9.78	-11.17	1.0000	
-4	-5	-7	2405	148
951	14.89	-5.66	1.0000	
-4	-5	-7	2424	163
963	14.89	-5.66	1.0000	
4	5	7	2637	157
1100	14.89	5.66	1.0000	
-6	-5	-7	1631	124
642	-2.54	-18.59	1.0000	
-6	-5	-7	1705	129
695	-2.54	-18.59	1.0000	
6	5	7	1447	137
495	-2.54	18.59	1.0000	
-8	-5	-7	1843	152
886	7.69	-8.48	1.0000	
-8	-5	-7	1432	122
609	7.69	-8.48	1.0000	
8	5	7	1274	167
508	7.69	8.48	1.0000	
-10	-5	-7	612	199
2080	-6.22	-22.70	1.0000	
10	5	7	643	267
2134	-6.22	22.70	1.0000	
-12	-5	-7	1779	175
1157	5.88	-8.68	1.0000	
12	5	7	1857	240
1220	5.88	8.68	1.0000	
-14	-5	-7	832	169

676 -1.16 2.59 1.0000

14 5 7 806 242

652 -1.16 -2.59 1.0000

-16 -5 -7 2054 246

2330 3.65 -3.62 1.0000

16 5 7 1816 330

2075 3.65 3.62 1.0000

-18 -5 -7 131 173

194 3.08 -0.30 1.0000

18 5 7 158 266

225 3.08 0.30 1.0000

-20 -5 -7 319 193

486 1.52 3.63 1.0000

20 5 7 21 275

-255 1.52 -3.63 1.0000

-22 -5 -7 299 210

410 2.46 -3.73 1.0000

22 5 7 261 316

364 2.46 3.73 1.0000

23 -6 -7 7 333

-80 -0.33 2.26 1.0000

23 -6 -7 7 305

-235 -0.33 2.26 1.0000

21 -6 -7 27 265

-36 3.75 2.55 1.0000

21 -6 -7 44 287

76 3.75 2.55 1.0000

19 -6 -7 36 253

77 1.34 -2.57 1.0000

19 -6 -7 96 230

160 1.34 -2.57 1.0000

17 -6 -7 602 236

661 1.02 1.35 1.0000

17 -6 -7 577 251

635 1.02 1.35 1.0000

15 -6 -7 520 203

189 -0.46 -8.07 1.0000

15 -6 -7 734 191

327 -0.46 -8.07 1.0000

13 -6 -7 620 191

764 0.04 -7.56 1.0000

13 -6 -7 532 210

661 0.04 -7.56 1.0000

11 -6 -7 207 144

215	0.28	-0.19	1.0000	
11	-6	-7	179	170
186	0.28	-0.19	1.0000	
9	-6	-7	1056	182
1228	0.74	2.09	1.0000	
9	-6	-7	938	227
1100	0.74	2.09	1.0000	
7	-6	-7	1410	155
541	14.11	8.54	1.0000	
7	-6	-7	1709	134
750	14.11	8.54	1.0000	
-7	6	7	1614	180
676	14.11	-8.54	1.0000	
5	-6	-7	1610	152
607	-2.61	18.64	1.0000	
5	-6	-7	1698	130
669	-2.61	18.64	1.0000	
5	-6	-7	1621	116
615	-2.61	18.64	1.0000	
3	-6	-7	6737	353
7486	3.28	2.23	1.0000	
3	-6	-7	7056	408
7822	3.28	2.23	1.0000	
3	-6	-7	6368	354
7097	3.28	2.23	1.0000	
1	-6	-7	1405	155
1241	-9.27	7.15	1.0000	
1	-6	-7	1349	190
1184	-9.27	7.15	1.0000	
1	-6	-7	1333	165
1168	-9.27	7.15	1.0000	
-1	-6	-7	466	134
500	-3.52	8.12	1.0000	
-1	-6	-7	563	135
611	-3.52	8.12	1.0000	
-3	-6	-7	889	186
1696	-12.94	-0.74	1.0000	
-3	-6	-7	490	195
1095	-12.94	-0.74	1.0000	
3	6	7	616	180
1284	-12.94	0.74	1.0000	
-5	-6	-7	856	197
2234	-7.50	14.43	1.0000	
5	6	7	730	219

2027 -7.50 -14.43 1.0000
-7 -6 -7 653 169
924 -3.27 12.89 1.0000
-7 -6 -7 1076 179
1482 -3.27 12.89 1.0000
7 6 7 835 187
1188 -3.27 -12.89 1.0000
-9 -6 -7 244 160
945 -1.10 21.64 1.0000
9 6 7 267 211
1040 -1.10 -21.64 1.0000
-11 -6 -7 2086 270
3986 6.43 15.55 1.0000
11 6 7 2260 348
4236 6.43 -15.55 1.0000
-13 -6 -7 1307 200
1530 -3.88 3.21 1.0000
13 6 7 1020 273
1215 -3.88 -3.21 1.0000
-15 -6 -7 1461 196
1139 1.03 4.32 1.0000
15 6 7 880 256
633 1.03 -4.32 1.0000
-17 -6 -7 351 188
499 -0.92 -4.18 1.0000
17 6 7 50 244
105 -0.92 4.18 1.0000
-19 -6 -7 522 182
282 -4.50 -3.02 1.0000
19 6 7 174 266
50 -4.50 3.02 1.0000
-21 -6 -7 128 191
118 -0.39 -0.13 1.0000
21 6 7 255 312
241 -0.39 0.13 1.0000
-23 -6 -7 117 225
184 -1.50 -1.87 1.0000
23 6 7 8 318
-158 -1.50 1.87 1.0000
22 -7 -7 277 326
270 0.21 -1.13 1.0000
22 -7 -7 2 271
-28 0.21 -1.13 1.0000
20 -7 -7 30 283

94 -3.20 -1.92 1.0000
20 -7 -7 19 237
-11 -3.20 -1.92 1.0000
18 -7 -7 355 245
249 -0.44 -2.72 1.0000
18 -7 -7 240 223
155 -0.44 -2.72 1.0000
16 -7 -7 1630 323
2218 0.83 6.10 1.0000
16 -7 -7 1195 314
1705 0.83 6.10 1.0000
14 -7 -7 271 208
260 0.21 -0.48 1.0000
14 -7 -7 297 196
285 0.21 -0.48 1.0000
12 -7 -7 540 176
469 5.33 7.43 1.0000
12 -7 -7 554 191
483 5.33 7.43 1.0000
10 -7 -7 929 150
592 -2.09 -5.06 1.0000
10 -7 -7 467 167
239 -2.09 -5.06 1.0000
8 -7 -7 5105 237
3176 -1.82 -13.11 1.0000
8 -7 -7 4956 281
3059 -1.82 -13.11 1.0000
6 -7 -7 862 171
1219 -3.18 -12.99 1.0000
6 -7 -7 844 217
1196 -3.18 -12.99 1.0000
4 -7 -7 2240 315
4728 -1.62 -23.32 1.0000
4 -7 -7 2242 265
4731 -1.62 -23.32 1.0000
4 -7 -7 2061 278
4455 -1.62 -23.32 1.0000
2 -7 -7 606 160
1126 6.88 -6.64 1.0000
2 -7 -7 833 166
1436 6.88 -6.64 1.0000
2 -7 -7 656 200
1195 6.88 -6.64 1.0000
0 -7 -7 1214 193

1332	8.32	-3.88	1.0000	
0	-7	-7	1250	203
1372	8.32	-3.88	1.0000	
0	-7	-7	1364	185
1496	8.32	-3.88	1.0000	
-2	-7	-7	4259	351
6724	12.41	-10.30	1.0000	
-2	-7	-7	3215	344
5384	12.41	-10.30	1.0000	
2	7	7	3802	330
6152	12.41	10.30	1.0000	
-4	-7	-7	780	182
920	4.29	-1.91	1.0000	
-6	-7	-7	1948	152
925	14.00	-8.61	1.0000	
-6	-7	-7	1838	154
844	14.00	-8.61	1.0000	
6	7	7	1707	176
757	14.00	8.61	1.0000	
-8	-7	-7	1538	228
3355	-5.43	-18.81	1.0000	
-8	-7	-7	1326	228
3025	-5.43	-18.81	1.0000	
8	7	7	1123	268
2701	-5.43	18.81	1.0000	
-10	-7	-7	231	131
124	1.54	-6.73	1.0000	
10	7	7	419	186
279	1.54	6.73	1.0000	
-12	-7	-7	1204	160
592	-1.19	-9.41	1.0000	
12	7	7	1784	261
1017	-1.19	9.41	1.0000	
-14	-7	-7	860	171
605	-1.11	-6.22	1.0000	
14	7	7	744	259
503	-1.11	6.22	1.0000	
-16	-7	-7	478	172
231	2.96	6.81	1.0000	
16	7	7	73	252
-30	2.96	-6.81	1.0000	
-18	-7	-7	75	183
45	1.55	0.74	1.0000	
18	7	7	71	280

42 1.55 -0.74 1.0000
-20 -7 -7 447 206
413 -0.42 -1.03 1.0000
20 7 7 37 277
27 -0.42 1.03 1.0000
-22 -7 -7 4 204
-170 0.60 1.66 1.0000
22 7 7 4 302
-40 0.60 -1.66 1.0000
21 -8 -7 144 272
129 0.00 0.57 1.0000
21 -8 -7 105 284
92 0.00 0.57 1.0000
19 -8 -7 39 231
-2 1.22 5.31 1.0000
19 -8 -7 39 260
-83 1.22 5.31 1.0000
17 -8 -7 364 220
319 2.45 -2.43 1.0000
17 -8 -7 237 246
198 2.45 -2.43 1.0000
15 -8 -7 197 210
129 -3.81 -0.23 1.0000
15 -8 -7 263 204
185 -3.81 -0.23 1.0000
13 -8 -7 245 188
350 -2.26 -1.66 1.0000
13 -8 -7 315 193
433 -2.26 -1.66 1.0000
11 -8 -7 547 183
432 -0.60 -2.25 1.0000
11 -8 -7 592 162
472 -0.60 -2.25 1.0000
9 -8 -7 885 201
1308 2.24 5.65 1.0000
9 -8 -7 954 248
1391 2.24 5.65 1.0000
7 -8 -7 1148 276
2758 -5.54 18.88 1.0000
7 -8 -7 1388 234
3141 -5.54 18.88 1.0000
5 -8 -7 1303 159
533 7.56 8.42 1.0000
5 -8 -7 1717 143

808 7.56 8.42 1.0000
3 -8 -7 2524 203
1015 -5.54 25.78 1.0000
3 -8 -7 2248 142
804 -5.54 25.78 1.0000
3 -8 -7 2189 154
759 -5.54 25.78 1.0000
1 -8 -7 291 139
126 -4.11 3.65 1.0000
1 -8 -7 239 126
92 -4.11 3.65 1.0000
1 -8 -7 332 128
154 -4.11 3.65 1.0000
-1 -8 -7 1106 159
821 -10.39 -0.41 1.0000
-3 -8 -7 2323 194
1114 -6.01 11.63 1.0000
3 8 7 2496 204
1235 -6.01 -11.63 1.0000
-5 -8 -7 2212 205
1484 -8.11 3.51 1.0000
5 8 7 2197 223
1473 -8.11 -3.51 1.0000
-7 -8 -7 5005 243
3091 -2.00 13.16 1.0000
-7 -8 -7 4583 225
2761 -2.00 13.16 1.0000
7 8 7 4770 279
2907 -2.00 -13.16 1.0000
-9 -8 -7 1185 141
438 3.35 12.24 1.0000
-9 -8 -7 1575 146
691 3.35 12.24 1.0000
9 8 7 1371 173
555 3.35 -12.24 1.0000
-11 -8 -7 1616 208
1635 0.61 5.97 1.0000
11 8 7 2001 298
2027 0.61 -5.97 1.0000
-13 -8 -7 874 183
914 4.66 8.51 1.0000
13 8 7 810 278
849 4.66 -8.51 1.0000
-15 -8 -7 919 194

950 0.48 -1.25 1.0000

15 8 7 506 272

529 0.48 1.25 1.0000

-17 -8 -7 22 182

153 -3.96 -5.41 1.0000

17 8 7 24 275

157 -3.96 5.41 1.0000

-19 -8 -7 446 214

556 0.62 -2.98 1.0000

19 8 7 322 304

414 0.62 2.98 1.0000

-21 -8 -7 7 201

-119 -0.56 -2.19 1.0000

21 8 7 7 310

-226 -0.56 2.19 1.0000

22 -9 -7 15 276

-19 -3.36 0.30 1.0000

22 -9 -7 114 327

202 -3.36 0.30 1.0000

20 -9 -7 159 269

363 1.60 -6.59 1.0000

20 -9 -7 225 292

464 1.60 -6.59 1.0000

18 -9 -7 11 221

-90 -0.75 2.82 1.0000

18 -9 -7 272 251

206 -0.75 2.82 1.0000

16 -9 -7 429 244

348 -0.73 -4.57 1.0000

16 -9 -7 28 209

-24 -0.73 -4.57 1.0000

14 -9 -7 450 197

177 7.25 4.03 1.0000

14 -9 -7 577 198

264 7.25 4.03 1.0000

12 -9 -7 634 190

697 -0.67 -3.01 1.0000

10 -9 -7 102 155

37 3.51 -2.60 1.0000

10 -9 -7 176 172

90 3.51 -2.60 1.0000

8 -9 -7 1069 149

361 3.35 -12.36 1.0000

8 -9 -7 1178 170

427	3.35	-12.36	1.0000	
6	-9	-7	203	157
808	-0.98	-21.57	1.0000	
6	-9	-7	165	171
605	-0.98	-21.57	1.0000	
4	-9	-7	2151	240
3251	5.35	-12.17	1.0000	
4	-9	-7	2074	281
3154	5.35	-12.17	1.0000	
2	-9	-7	440	187
1112	-0.74	-24.54	1.0000	
2	-9	-7	499	214
1276	-0.74	-24.54	1.0000	
0	-9	-7	442	163
751	11.96	4.33	1.0000	
0	-9	-7	316	144
549	11.96	4.33	1.0000	
-2	-9	-7	1137	161
824	7.25	0.35	1.0000	
2	9	7	991	163
694	7.25	-0.35	1.0000	
-4	-9	-7	1071	212
1750	9.07	-5.85	1.0000	
4	9	7	823	228
1427	9.07	5.85	1.0000	
-6	-9	-7	1059	180
1225	0.89	-1.98	1.0000	
-6	-9	-7	1088	208
1256	0.89	-1.98	1.0000	
6	9	7	1376	255
1565	0.89	1.98	1.0000	
-8	-9	-7	1125	221
1590	2.17	-5.57	1.0000	
-8	-9	-7	1182	195
1658	2.17	-5.57	1.0000	
8	9	7	1159	247
1631	2.17	5.57	1.0000	
-10	-9	-7	358	149
454	-3.60	-8.10	1.0000	
-10	-9	-7	326	154
413	-3.60	-8.10	1.0000	
10	9	7	233	201
296	-3.60	8.10	1.0000	
-12	-9	-7	360	158

391	-4.89	-1.65	1.0000	
12	9	7	111	235
111	-4.89	1.65	1.0000	
-14	-9	-7	1	154
-27	0.82	0.43	1.0000	
14	9	7	86	257
107	0.82	-0.43	1.0000	
-16	-9	-7	39	172
72	-1.87	-1.39	1.0000	
16	9	7	7	267
-149	-1.87	1.39	1.0000	
-18	-9	-7	311	198
262	-0.44	5.74	1.0000	
18	9	7	44	275
-290	-0.44	-5.74	1.0000	
-20	-9	-7	182	215
247	2.44	0.06	1.0000	
20	9	7	118	314
170	2.44	-0.06	1.0000	
-22	-9	-7	2	230
-51	-1.24	-0.30	1.0000	
22	9	7	324	349
379	-1.24	0.30	1.0000	
21	-10	-7	36	267
-123	2.16	4.71	1.0000	
21	-10	-7	56	312
177	2.16	4.71	1.0000	
19	-10	-7	107	243
135	1.13	-1.76	1.0000	
19	-10	-7	499	289
563	1.13	-1.76	1.0000	
17	-10	-7	31	246
-123	-3.36	3.50	1.0000	
17	-10	-7	31	213
-23	-3.36	3.50	1.0000	
15	-10	-7	201	219
142	-0.22	2.05	1.0000	
15	-10	-7	255	216
188	-0.22	2.05	1.0000	
13	-10	-7	1343	263
1777	-3.55	-3.47	1.0000	
13	-10	-7	1139	293
1541	-3.55	-3.47	1.0000	
11	-10	-7	661	196

```
848    -4.18     4.12  1.0000
  11 -10   -7       570       220
742    -4.18     4.12  1.0000
   9 -10   -7       340       179
438    -3.54     8.02  1.0000
   9 -10   -7       453       192
581    -3.54     8.02  1.0000
   7 -10   -7        64       158
-83     1.47     6.77  1.0000
   7 -10   -7       231       143
120     1.47     6.77  1.0000
   5 -10   -7       478       240
1761    -6.12    22.66  1.0000
   5 -10   -7       459       216
1715    -6.12    22.66  1.0000
   3 -10   -7      1910       262
2328     5.22     7.15  1.0000
   3 -10   -7      1958       233
2381     5.22     7.15  1.0000
   1 -10   -7       433       191
1135    -5.91     9.67  1.0000
   1 -10   -7       433       199
1134    -5.91     9.67  1.0000
  -1 -10   -7       278       150
402    -3.03     0.12  1.0000
  -3 -10   -7       335       145
335    -4.69    -5.74  1.0000
   3  10    7       166       152
143    -4.69     5.74  1.0000
  -5 -10   -7      1141       223
1319    -0.56     2.16  1.0000
   5  10    7      1099       204
1274    -0.56    -2.16  1.0000
  -7 -10   -7       846       152
526    -2.13     5.02  1.0000
  -7 -10   -7       659       147
381    -2.13     5.02  1.0000
   7  10    7       809       195
498    -2.13    -5.02  1.0000
  -9 -10   -7        76       140
18      3.63     2.55  1.0000
  -9 -10   -7        26       147
-33     3.63     2.55  1.0000
   9  10    7       190       211
```

97 3.63 -2.55 1.0000
-11 -10 -7 560 186
1037 5.50 8.28 1.0000
11 10 7 239 238
546 5.50 -8.28 1.0000
-13 -10 -7 26 154
-27 3.31 -2.88 1.0000
13 10 7 74 235
164 3.31 2.88 1.0000
-15 -10 -7 947 214
1207 -0.87 -3.64 1.0000
15 10 7 821 309
1064 -0.87 3.64 1.0000
-17 -10 -7 20 197
26 3.66 -0.21 1.0000
17 10 7 178 293
232 3.66 0.21 1.0000
-19 -10 -7 421 217
246 -2.38 -3.72 1.0000
19 10 7 316 328
167 -2.38 3.72 1.0000
-21 -10 -7 16 225
-60 -1.49 -3.09 1.0000
21 10 7 13 342
53 -1.49 3.09 1.0000
20 -11 -7 183 270
217 -1.25 2.64 1.0000
20 -11 -7 300 294
346 -1.25 2.64 1.0000
18 -11 -7 40 261
-190 1.23 -5.32 1.0000
18 -11 -7 21 241
117 1.23 -5.32 1.0000
16 -11 -7 483 235
666 3.09 -1.92 1.0000
16 -11 -7 265 242
404 3.09 -1.92 1.0000
14 -11 -7 916 236
761 -1.03 2.46 1.0000
14 -11 -7 902 236
748 -1.03 2.46 1.0000
12 -11 -7 650 201
574 6.25 0.16 1.0000
12 -11 -7 519 208

446 6.25 0.16 1.0000
10 -11 -7 864 259
1451 5.61 -8.19 1.0000
10 -11 -7 1035 287
1675 5.61 -8.19 1.0000
6 -11 -7 2499 316
4563 6.37 -15.60 1.0000
6 -11 -7 2478 350
4534 6.37 -15.60 1.0000
4 -11 -7 150 193
876 -4.59 -17.69 1.0000
4 -11 -7 122 174
786 -4.59 -17.69 1.0000
2 -11 -7 930 194
1218 8.51 0.33 1.0000
2 -11 -7 908 199
1192 8.51 0.33 1.0000
0 -11 -7 1213 174
503 2.89 -10.54 1.0000
4 11 7 1196 265
2085 5.27 -8.56 1.0000
6 11 7 285 178
293 0.26 -0.21 1.0000
-8 -11 -7 141 154
85 -0.56 2.27 1.0000
-8 -11 -7 7 137
-34 -0.56 2.27 1.0000
8 11 7 7 218
-258 -0.56 -2.27 1.0000
-10 -11 -7 418 172
554 -4.26 -3.99 1.0000
-10 -11 -7 601 176
770 -4.26 -3.99 1.0000
10 11 7 554 255
715 -4.26 3.99 1.0000
-12 -11 -7 169 168
262 -4.54 -2.80 1.0000
12 11 7 100 241
166 -4.54 2.80 1.0000
-14 -11 -7 963 195
683 -3.16 4.90 1.0000
14 11 7 713 239
469 -3.16 -4.90 1.0000
-16 -11 -7 124 192

113 -0.52 2.01 1.0000
16 11 7 128 267
114 -0.52 -2.01 1.0000
-18 -11 -7 1 211
9 -1.64 -0.98 1.0000
18 11 7 87 312
130 -1.64 0.98 1.0000
-20 -11 -7 99 232
223 -0.37 4.45 1.0000
20 11 7 113 327
244 -0.37 -4.45 1.0000
19 -12 -7 19 266
-393 -2.20 3.04 1.0000
19 -12 -7 64 248
117 -2.20 3.04 1.0000
17 -12 -7 18 216
-133 1.50 3.41 1.0000
17 -12 -7 76 260
43 1.50 3.41 1.0000
15 -12 -7 175 213
212 -3.82 -2.14 1.0000
15 -12 -7 271 227
322 -3.82 -2.14 1.0000
13 -12 -7 882 240
897 -1.89 0.15 1.0000
13 -12 -7 469 216
479 -1.89 0.15 1.0000
11 -12 -7 50 193
87 -4.54 2.75 1.0000
11 -12 -7 173 187
265 -4.54 2.75 1.0000
9 -12 -7 375 179
404 -4.97 1.59 1.0000
9 -12 -7 542 211
585 -4.97 1.59 1.0000
7 -12 -7 1570 190
859 -1.11 9.35 1.0000
7 -12 -7 1403 199
736 -1.11 9.35 1.0000
5 -12 -7 1935 208
1281 5.89 8.73 1.0000
5 -12 -7 1936 254
1282 5.89 8.73 1.0000
3 -12 -7 541 175

504	-5.46	7.04	1.0000	
1	-12	-7	317	176
320	5.80	0.80	1.0000	
-1	-12	-7	570	224
831	-1.00	-4.40	1.0000	
3	12	7	969	184
423	1.76	8.98	1.0000	
5	12	7	1262	217
1006	-0.93	4.02	1.0000	
-7	-12	-7	903	174
854	5.37	-7.48	1.0000	
7	12	7	672	231
607	5.37	7.48	1.0000	
-9	-12	-7	885	185
969	-0.78	3.05	1.0000	
9	12	7	763	272
840	-0.78	-3.05	1.0000	
-11	-12	-7	640	174
556	6.28	-0.32	1.0000	
-11	-12	-7	601	187
518	6.28	-0.32	1.0000	
11	12	7	395	243
318	6.28	0.32	1.0000	
-13	-12	-7	87	176
162	3.13	0.51	1.0000	
13	12	7	71	256
139	3.13	-0.51	1.0000	
-15	-12	-7	613	214
700	2.36	-0.57	1.0000	
15	12	7	204	245
253	2.36	0.57	1.0000	
-17	-12	-7	210	221
88	-0.83	-4.41	1.0000	
17	12	7	305	266
153	-0.83	4.41	1.0000	
-19	-12	-7	273	234
220	0.62	-1.34	1.0000	
19	12	7	3	298
-201	0.62	1.34	1.0000	
18	-13	-7	14	239
-97	-2.14	-2.45	1.0000	
18	-13	-7	60	270
56	-2.14	-2.45	1.0000	
16	-13	-7	13	219

-130	3.05	0.63	1.0000	
16	-13	-7	67	250
130	3.05	0.63	1.0000	
14	-13	-7	204	240
259	1.74	-1.48	1.0000	
14	-13	-7	477	229
561	1.74	-1.48	1.0000	
12	-13	-7	72	214
140	3.13	-0.50	1.0000	
12	-13	-7	61	195
125	3.13	-0.50	1.0000	
10	-13	-7	296	208
472	3.45	2.91	1.0000	
10	-13	-7	168	191
304	3.45	2.91	1.0000	
8	-13	-7	1187	245
1257	4.65	-8.43	1.0000	
8	-13	-7	1002	237
1056	4.65	-8.43	1.0000	
6	-13	-7	1354	244
1577	-3.85	-3.16	1.0000	
4	-13	-7	259	192
764	4.61	-8.89	1.0000	
2	-13	-7	1253	270
1908	-2.09	-7.07	1.0000	
0	-13	-7	920	214
703	-2.40	10.95	1.0000	
4	13	7	267	229
901	-2.93	-11.48	1.0000	
6	13	7	848	241
1031	0.00	-7.59	1.0000	
-8	-13	-7	357	181
484	-2.33	1.60	1.0000	
8	13	7	357	233
485	-2.33	-1.60	1.0000	
-10	-13	-7	1594	234
2064	-3.52	3.50	1.0000	
10	13	7	828	290
1176	-3.52	-3.50	1.0000	
-12	-13	-7	938	216
961	-1.91	-0.05	1.0000	
12	13	7	803	268
824	-1.91	0.05	1.0000	
-14	-13	-7	46	192

-217 -4.43 -3.83 1.0000
14 13 7 20 273
127 -4.43 3.83 1.0000
-16 -13 -7 538 218
617 -3.02 3.47 1.0000
16 13 7 176 300
210 -3.02 -3.47 1.0000
-18 -13 -7 628 258
882 2.69 3.80 1.0000
18 13 7 6 268
25 2.69 -3.80 1.0000
19 -14 -7 22 262
-31 4.05 -0.65 1.0000
19 -14 -7 270 306
435 4.05 -0.65 1.0000
17 -14 -7 224 247
286 -1.63 0.46 1.0000
17 -14 -7 49 257
80 -1.63 0.46 1.0000
15 -14 -7 109 255
107 -1.14 -1.22 1.0000
15 -14 -7 324 228
322 -1.14 -1.22 1.0000
13 -14 -7 25 236
136 -4.27 3.94 1.0000
13 -14 -7 45 190
-106 -4.27 3.94 1.0000
11 -14 -7 954 216
667 -3.21 -5.03 1.0000
11 -14 -7 852 232
581 -3.21 -5.03 1.0000
9 -14 -7 364 197
405 0.87 -0.27 1.0000
9 -14 -7 200 200
231 0.87 -0.27 1.0000
7 -14 -7 813 205
565 -1.07 6.09 1.0000
5 -14 -7 1402 251
1200 -1.14 -2.57 1.0000
3 -14 -7 2215 342
3478 10.37 4.70 1.0000
1 -14 -7 280 218
349 -0.77 -1.54 1.0000
-1 -14 -7 1174 300

1748 6.93 -9.27 1.0000
5 14 7 2 218
161 2.79 11.60 1.0000
-7 -14 -7 335 176
321 0.22 0.52 1.0000
7 14 7 0 211
-65 0.22 -0.52 1.0000
-9 -14 -7 571 179
258 7.25 -3.98 1.0000
9 14 7 91 224
-38 7.25 3.98 1.0000
-11 -14 -7 649 208
524 -1.17 -2.39 1.0000
11 14 7 939 286
788 -1.17 2.39 1.0000
-13 -14 -7 287 197
347 1.79 1.34 1.0000
13 14 7 147 273
189 1.79 -1.34 1.0000
-15 -14 -7 472 219
377 5.10 0.47 1.0000
15 14 7 244 293
169 5.10 -0.47 1.0000
-17 -14 -7 327 256
408 -2.46 -0.49 1.0000
17 14 7 416 314
508 -2.46 0.49 1.0000
-19 -14 -7 4 255
-320 -1.18 -1.15 1.0000
19 14 7 4 319
-75 -1.18 1.15 1.0000
18 -15 -7 1 269
-218 0.88 0.59 1.0000
18 -15 -7 1 255
-160 0.88 0.59 1.0000
16 -15 -7 392 241
298 -4.03 -0.30 1.0000
16 -15 -7 22 259
-300 -4.03 -0.30 1.0000
14 -15 -7 41 233
2 5.05 -0.55 1.0000
14 -15 -7 34 205
-22 5.05 -0.55 1.0000
12 -15 -7 452 223

531	2.50	0.60	1.0000	
12	-15	-7	497	247
580	2.50	0.60	1.0000	
10	-15	-7	970	268
1234	-0.89	3.65	1.0000	
10	-15	-7	1211	302
1504	-0.89	3.65	1.0000	
8	-15	-7	904	242
930	0.49	1.16	1.0000	
6	-15	-7	1140	235
864	0.94	-4.27	1.0000	
4	-15	-7	2456	330
2929	-1.72	3.95	1.0000	
2	-15	-7	73	217
185	-5.32	-1.97	1.0000	
0	-15	-7	112	230
164	1.25	-1.74	1.0000	
-2	-15	-7	253	282
775	-5.12	14.89	1.0000	
4	15	7	611	239
940	4.68	-2.30	1.0000	
6	15	7	88	225
-66	-0.49	-8.14	1.0000	
-8	-15	-7	434	196
334	-3.85	0.28	1.0000	
8	15	7	390	240
294	-3.85	-0.28	1.0000	
-10	-15	-7	403	204
312	-0.16	-2.22	1.0000	
10	15	7	364	244
278	-0.16	2.22	1.0000	
-12	-15	-7	300	206
357	-3.89	2.22	1.0000	
12	15	7	27	263
-119	-3.89	-2.22	1.0000	
-14	-15	-7	590	239
593	-1.22	1.18	1.0000	
14	15	7	213	305
214	-1.22	-1.18	1.0000	
-16	-15	-7	346	242
302	0.46	-1.30	1.0000	
16	15	7	326	297
283	0.46	1.30	1.0000	
-18	-15	-7	469	275

590 -1.75 1.98 1.0000
18 15 7 297 337
393 -1.75 -1.98 1.0000
17 -16 -7 19 260
-135 0.81 -3.73 1.0000
17 -16 -7 19 271
-309 0.81 -3.73 1.0000
15 -16 -7 61 231
42 0.51 1.38 1.0000
15 -16 -7 45 267
29 0.51 1.38 1.0000
13 -16 -7 268 260
319 -2.92 -3.51 1.0000
13 -16 -7 28 204
-97 -2.92 -3.51 1.0000
11 -16 -7 29 232
21 -0.45 -1.83 1.0000
11 -16 -7 124 203
111 -0.45 -1.83 1.0000
9 -16 -7 6 216
-247 -1.80 1.24 1.0000
7 -16 -7 1055 241
683 3.03 -6.64 1.0000
5 -16 -7 2206 332
2473 3.54 3.42 1.0000
3 -16 -7 653 244
740 -1.40 -1.62 1.0000
1 -16 -7 1040 250
574 11.21 -4.65 1.0000
-1 -16 -7 93 240
128 -1.79 -0.43 1.0000
5 16 7 189 237
286 -0.54 2.88 1.0000
-7 -16 -7 1445 256
1996 0.73 -6.07 1.0000
7 16 7 1318 321
1845 0.73 6.07 1.0000
-9 -16 -7 508 213
419 -0.79 4.58 1.0000
9 16 7 632 264
535 -0.79 -4.58 1.0000
-11 -16 -7 344 227
503 3.14 1.94 1.0000
11 16 7 247 266

383	3.14	-1.94	1.0000	
-13 -16	-7	25		217
62	2.95	-0.58	1.0000	
13 16	7	12		265
-338	2.95	0.58	1.0000	
-15 -16	-7	386		250
299	-4.05	0.15	1.0000	
15 16	7	52		295
15	-4.05	-0.15	1.0000	
-17 -16	-7	250		277
339	2.25	0.27	1.0000	
17 16	7	335		342
438	2.25	-0.27	1.0000	
16 -17	-7	75		254
127	2.22	-0.32	1.0000	
16 -17	-7	7		287
-49	2.22	-0.32	1.0000	
14 -17	-7	183		274
245	-2.44	0.46	1.0000	
14 -17	-7	194		245
257	-2.44	0.46	1.0000	
12 -17	-7	589		227
371	-0.81	4.35	1.0000	
12 -17	-7	26		243
-259	-0.81	4.35	1.0000	
10 -17	-7	17		217
-275	3.60	0.05	1.0000	
8 -17	-7	12		228
124	-3.92	5.56	1.0000	
6 -17	-7	248		233
377	-0.85	4.23	1.0000	
4 -17	-7	68		229
241	-5.25	-5.33	1.0000	
2 -17	-7	20		241
-27	0.76	3.80	1.0000	
0 -17	-7	159		258
355	-3.95	3.73	1.0000	
-2 -17	-7	99		261
72	1.52	-5.51	1.0000	
6 17	7	472		272
523	1.13	1.35	1.0000	
-8 -17	-7	69		212
42	2.53	2.48	1.0000	
8 17	7	346		272

302 2.53 -2.48 1.0000
-10 -17 -7 469 230
358 -3.45 -3.46 1.0000
10 17 7 363 266
265 -3.45 3.46 1.0000
-12 -17 -7 91 232
59 1.52 -3.53 1.0000
12 17 7 263 272
216 1.52 3.53 1.0000
-14 -17 -7 324 261
396 -1.59 -0.36 1.0000
14 17 7 314 313
384 -1.59 0.36 1.0000
-16 -17 -7 480 279
667 0.69 3.73 1.0000
16 17 7 13 342
50 0.69 -3.73 1.0000
15 -18 -7 245 264
326 -1.61 -1.89 1.0000
15 -18 -7 238 295
318 -1.61 -1.89 1.0000
13 -18 -7 29 231
-48 2.70 -3.85 1.0000
13 -18 -7 26 268
86 2.70 -3.85 1.0000
11 -18 -7 4 248
-35 -1.51 1.01 1.0000
9 -18 -7 256 252
214 -0.31 -5.65 1.0000
7 -18 -7 276 232
214 1.48 -0.89 1.0000
5 -18 -7 343 248
441 3.04 0.33 1.0000
3 -18 -7 55 237
0 6.28 -1.48 1.0000
1 -18 -7 527 262
567 -4.29 2.39 1.0000
-1 -18 -7 51 257
-203 5.28 -3.27 1.0000
-3 -18 -7 852 338
887 -2.86 1.25 1.0000
5 18 7 10 231
-230 0.29 2.69 1.0000
-7 -18 -7 472 226

348 -0.56 2.75 1.0000
7 18 7 165 263
95 -0.56 -2.75 1.0000
-9 -18 -7 381 231
299 -0.77 -2.83 1.0000
-11 -18 -7 40 233
-73 1.14 5.36 1.0000
11 18 7 107 275
277 1.14 -5.36 1.0000
-13 -18 -7 27 258
18 -2.19 2.40 1.0000
13 18 7 373 300
389 -2.19 -2.40 1.0000
-15 -18 -7 451 281
482 0.96 -0.65 1.0000
15 18 7 2 312
-12 0.96 0.65 1.0000
14 -19 -7 130 252
165 -1.11 1.08 1.0000
14 -19 -7 3 296
-13 -1.11 1.08 1.0000
12 -19 -7 397 295
334 0.64 1.29 1.0000
10 -19 -7 150 266
52 -2.38 3.87 1.0000
8 -19 -7 237 271
314 0.63 2.87 1.0000
6 -19 -7 339 254
155 -4.38 3.11 1.0000
4 -19 -7 260 246
251 -1.27 2.15 1.0000
2 -19 -7 216 273
177 -4.45 -4.43 1.0000
0 -19 -7 2 273
-25 0.16 -1.28 1.0000
-2 -19 -7 30 281
7 2.35 2.52 1.0000
-4 -19 -7 33 319
-54 0.77 -4.94 1.0000
6 19 7 194 256
283 1.22 -2.59 1.0000
-8 -19 -7 40 240
-121 1.21 -5.33 1.0000
8 19 7 285 279

145 1.21 5.33 1.0000

-10 -19 -7 32 251

48 1.24 1.85 1.0000

10 19 7 7 279

-508 1.24 -1.85 1.0000

-12 -19 -7 12 261

30 -2.09 -3.13 1.0000

12 19 7 222 296

322 -2.09 3.13 1.0000

-14 -19 -7 575 293

802 3.99 0.57 1.0000

14 19 7 197 319

338 3.99 -0.57 1.0000

11 -20 -7 143 277

290 -0.27 -4.54 1.0000

9 -20 -7 204 278

271 2.40 -0.10 1.0000

7 -20 -7 444 279

407 -0.48 1.07 1.0000

5 -20 -7 151 271

271 1.58 -3.70 1.0000

3 -20 -7 168 269

118 1.88 4.27 1.0000

1 -20 -7 401 300

496 4.25 0.91 1.0000

-1 -20 -7 44 298

160 -5.80 2.78 1.0000

-3 -20 -7 6 299

-28 2.12 0.03 1.0000

-7 -20 -7 127 252

239 -3.17 1.94 1.0000

7 20 7 124 265

235 -3.17 -1.94 1.0000

-9 -20 -7 155 267

357 1.63 6.57 1.0000

9 20 7 185 313

402 1.63 -6.57 1.0000

-11 -20 -7 675 287

753 -1.31 -2.71 1.0000

11 20 7 12 316

-74 -1.31 2.71 1.0000

10 -21 -7 233 314

360 -1.39 3.02 1.0000

8 -21 -7 149 288

194	-0.47	2.28	1.0000	
6	-21	-7	0	274
-80	-0.35	0.19	1.0000	
4	-21	-7	104	294
243	-4.48	1.38	1.0000	
2	-21	-7	882	308
898	-0.45	-0.36	1.0000	
0	-21	-7	749	336
1059	0.76	-4.80	1.0000	
-2	-21	-7	296	336
285	-1.68	-0.95	1.0000	
-4	-21	-7	301	353
376	3.04	0.39	1.0000	
-6	-21	-7	310	269
423	3.78	-2.55	1.0000	
-8	-21	-7	136	281
122	0.01	-0.56	1.0000	
8	21	7	0	290
-78	0.01	0.56	1.0000	
-10	-21	-7	234	289
447	2.13	-4.70	1.0000	
10	21	7	4	312
58	2.13	4.70	1.0000	
9	-22	-7	322	319
381	-1.32	0.36	1.0000	
7	-22	-7	343	310
381	0.56	-1.74	1.0000	
5	-22	-7	341	304
463	2.39	3.76	1.0000	
3	-22	-7	233	307
238	0.43	-0.10	1.0000	
1	-22	-7	311	316
272	-1.44	2.27	1.0000	
-1	-22	-7	159	330
280	3.41	1.96	1.0000	
-3	-22	-7	403	351
386	-4.49	1.01	1.0000	
-5	-22	-7	296	402
243	-1.11	2.07	1.0000	
-7	-22	-7	321	293
312	0.26	1.18	1.0000	
-9	-22	-7	18	292
58	-3.39	-0.35	1.0000	
9	22	7	167	315

272 -3.39 0.35 1.0000
6 -23 -7 8 325
-17 -1.52 1.89 1.0000
4 -23 -7 2 296
-237 0.78 -1.00 1.0000
2 -23 -7 288 331
387 -2.31 -0.73 1.0000
0 -23 -7 797 363
813 -0.87 0.41 1.0000
-2 -23 -7 9 332
-426 1.45 -2.19 1.0000
-4 -23 -7 4 355
-123 1.61 -0.28 1.0000
-6 -23 -7 7 302
-136 -0.29 -2.22 1.0000
-6 -23 -7 7 401
-150 -0.29 -2.22 1.0000
6 22 -8 516 332
541 2.88 -0.02 1.0000
-6 -22 8 453 395
477 2.88 0.02 1.0000
4 22 -8 161 311
231 -2.49 2.43 1.0000
-4 -22 8 372 372
485 -2.49 -2.43 1.0000
2 22 -8 347 310
310 2.24 0.10 1.0000
-2 -22 8 283 339
248 2.24 -0.10 1.0000
0 22 -8 2 294
-46 0.77 -0.81 1.0000
0 -22 8 104 343
118 0.77 0.81 1.0000
-2 22 -8 13 313
4 -1.21 0.62 1.0000
2 -22 8 2 330
-23 -1.21 -0.62 1.0000
-4 22 -8 326 340
301 0.64 0.13 1.0000
4 -22 8 1 315
-413 0.64 -0.13 1.0000
-6 22 -8 310 361
317 1.93 -0.16 1.0000
6 -22 8 102 307

104 1.93 0.16 1.0000
9 21 -8 621 369
783 -2.36 -1.23 1.0000
-9 -21 8 32 417
76 -2.36 1.23 1.0000
7 21 -8 257 315
397 -2.15 -3.46 1.0000
-7 -21 8 302 398
453 -2.15 3.46 1.0000
5 21 -8 2 282
-25 -0.58 -1.08 1.0000
-5 -21 8 2 346
-39 -0.58 1.08 1.0000
3 21 -8 1 275
-150 -0.35 -0.94 1.0000
-3 -21 8 281 337
313 -0.35 0.94 1.0000
1 21 -8 391 286
567 -4.59 -1.35 1.0000
-1 -21 8 282 344
434 -4.59 1.35 1.0000
-1 21 -8 31 279
63 2.05 2.08 1.0000
1 -21 8 60 321
107 2.05 -2.08 1.0000
-3 21 -8 469 298
417 -0.96 1.08 1.0000
3 -21 8 264 300
225 -0.96 -1.08 1.0000
-5 21 -8 99 328
202 -1.28 3.85 1.0000
5 -21 8 481 320
689 -1.28 -3.85 1.0000
-7 21 -8 265 347
262 0.14 0.13 1.0000
7 -21 8 53 299
52 0.14 -0.13 1.0000
-9 21 -8 131 372
185 1.13 1.52 1.0000
9 -21 8 17 287
40 1.13 -1.52 1.0000
10 20 -8 596 367
627 0.66 -0.01 1.0000
8 20 -8 799 327

1099	3.61	2.22	1.0000	
-8	-20	8	5	382
50	3.61	-2.22	1.0000	
6	20	-8	289	288
480	-1.65	4.02	1.0000	
-6	-20	8	53	346
147	-1.65	-4.02	1.0000	
4	20	-8	23	246
4	3.66	-0.96	1.0000	
-4	-20	8	239	346
223	3.66	0.96	1.0000	
2	20	-8	264	263
253	-1.01	4.06	1.0000	
-2	-20	8	44	302
26	-1.01	-4.06	1.0000	
0	20	-8	47	250
42	0.99	-1.17	1.0000	
0	-20	8	3	292
-34	0.99	1.17	1.0000	
-2	20	-8	1	256
17	0.01	-3.79	1.0000	
2	-20	8	121	298
233	0.01	3.79	1.0000	
-4	20	-8	132	269
138	-0.15	-0.15	1.0000	
4	-20	8	311	281
319	-0.15	0.15	1.0000	
-6	20	-8	305	318
460	-1.61	-3.29	1.0000	
6	-20	8	319	278
476	-1.61	3.29	1.0000	
-8	20	-8	284	325
217	1.90	1.41	1.0000	
8	-20	8	7	273
-3	1.90	-1.41	1.0000	
-10	20	-8	51	340
62	-1.72	0.50	1.0000	
10	-20	8	4	280
-152	-1.72	-0.50	1.0000	
11	19	-8	1064	379
1283	-1.59	-3.14	1.0000	
9	19	-8	272	281
272	0.05	-0.05	1.0000	
-9	-19	8	268	367

268 0.05 0.05 1.0000
7 19 -8 630 269
530 -1.47 -1.37 1.0000
-7 -19 8 5 338
-36 -1.47 1.37 1.0000
5 19 -8 310 244
247 -0.99 -3.44 1.0000
-5 -19 8 17 323
-149 -0.99 3.44 1.0000
3 19 -8 15 236
-128 -3.03 -1.54 1.0000
-3 -19 8 210 301
118 -3.03 1.54 1.0000
1 19 -8 269 242
282 2.23 0.21 1.0000
-1 -19 8 596 309
618 2.23 -0.21 1.0000
-1 19 -8 74 256
170 -3.88 0.50 1.0000
1 -19 8 1 284
28 -3.88 -0.50 1.0000
-3 19 -8 48 239
-230 0.62 5.98 1.0000
3 -19 8 228 280
82 0.62 -5.98 1.0000
-5 19 -8 93 277
221 2.35 3.97 1.0000
5 -19 8 6 274
59 2.35 -3.97 1.0000
-7 19 -8 981 314
804 -0.27 3.05 1.0000
7 -19 8 337 274
236 -0.27 -3.05 1.0000
-9 19 -8 495 323
501 -0.18 1.98 1.0000
9 -19 8 236 280
241 -0.18 -1.98 1.0000
-11 19 -8 56 325
92 2.31 -0.13 1.0000
11 -19 8 245 291
319 2.31 0.13 1.0000
14 18 -8 95 325
102 -0.09 -0.31 1.0000
14 18 -8 518 417

535	-0.09	-0.31	1.0000	
12	18	-8	742	364
717	2.28	-1.91	1.0000	
12	18	-8	255	288
235	2.28	-1.91	1.0000	
10	18	-8	374	302
381	1.90	3.07	1.0000	
8	18	-8	240	252
312	-1.30	-1.67	1.0000	
-8	-18	8	6	330
-53	-1.30	1.67	1.0000	
6	18	-8	196	224
167	1.78	1.45	1.0000	
-6	-18	8	7	312
-351	1.78	-1.45	1.0000	
4	18	-8	1	213
21	-2.93	3.98	1.0000	
-4	-18	8	24	294
112	-2.93	-3.98	1.0000	
2	18	-8	368	217
197	1.94	-4.03	1.0000	
-2	-18	8	613	287
384	1.94	4.03	1.0000	
0	18	-8	535	230
482	-1.03	-0.37	1.0000	
0	-18	8	664	280
605	-1.03	0.37	1.0000	
-2	18	-8	144	234
177	0.62	-2.46	1.0000	
2	-18	8	9	248
-17	0.62	2.46	1.0000	
-4	18	-8	117	264
355	-0.26	-10.41	1.0000	
-4	18	-8	48	159
58	-0.26	-10.41	1.0000	
4	-18	8	204	257
527	-0.26	10.41	1.0000	
-6	18	-8	1033	199
872	-1.63	1.83	1.0000	
-6	18	-8	1037	301
875	-1.63	1.83	1.0000	
6	-18	8	808	272
666	-1.63	-1.83	1.0000	
-8	18	-8	934	208

1134 -2.10 -2.33 1.0000
-8 18 -8 1302 335
1537 -2.10 -2.33 1.0000
8 -18 8 755 285
936 -2.10 2.33 1.0000
-10 18 -8 9 295
-130 1.05 -2.33 1.0000
10 -18 8 9 243
-87 1.05 2.33 1.0000
-12 18 -8 480 295
698 -0.36 4.21 1.0000
12 -18 8 45 270
123 -0.36 -4.21 1.0000
14 -18 8 64 297
185 -2.19 4.35 1.0000
15 17 -8 642 329
552 1.77 0.73 1.0000
15 17 -8 733 411
636 1.77 0.73 1.0000
13 17 -8 618 346
694 -2.72 -0.65 1.0000
13 17 -8 377 322
435 -2.72 -0.65 1.0000
11 17 -8 31 291
-73 -1.93 4.39 1.0000
11 17 -8 115 281
238 -1.93 4.39 1.0000
9 17 -8 539 281
539 -1.30 -2.63 1.0000
7 17 -8 494 243
482 1.31 0.20 1.0000
-7 -17 8 164 326
156 1.31 -0.20 1.0000
5 17 -8 47 207
79 0.98 -1.56 1.0000
-5 -17 8 4 282
-190 0.98 1.56 1.0000
3 17 -8 593 230
381 4.58 -1.25 1.0000
-3 -17 8 547 289
343 4.58 1.25 1.0000
1 17 -8 1084 252
1190 -1.25 1.27 1.0000
-1 -17 8 1159 304

1268 -1.25 -1.27 1.0000
-1 17 -8 752 272
1211 2.08 6.08 1.0000
1 -17 8 860 305
1348 2.08 -6.08 1.0000
-3 17 -8 133 143
251 -0.09 4.61 1.0000
-3 17 -8 166 236
297 -0.09 4.61 1.0000
3 -17 8 131 240
246 -0.09 -4.61 1.0000
-5 17 -8 197 166
368 -0.14 4.47 1.0000
-5 17 -8 273 247
470 -0.14 4.47 1.0000
5 -17 8 181 242
347 -0.14 -4.47 1.0000
-7 17 -8 334 262
447 4.59 4.02 1.0000
-7 17 -8 204 174
284 4.59 4.02 1.0000
7 -17 8 92 243
129 4.59 -4.02 1.0000
-9 17 -8 236 185
213 0.98 2.19 1.0000
-9 17 -8 129 256
110 0.98 2.19 1.0000
9 -17 8 255 245
231 0.98 -2.19 1.0000
-11 17 -8 249 274
210 -0.89 -1.34 1.0000
-11 17 -8 132 191
104 -0.89 -1.34 1.0000
11 -17 8 147 258
117 -0.89 1.34 1.0000
13 -17 8 243 280
326 1.51 -1.78 1.0000
15 -17 8 11 287
-30 1.12 2.64 1.0000
16 16 -8 68 304
98 -1.56 0.88 1.0000
16 16 -8 833 434
935 -1.56 0.88 1.0000
14 16 -8 612 323

658	0.69	-0.48	1.0000	
14	16	-8	1069	373
1129	0.69	-0.48	1.0000	
12	16	-8	478	301
623	3.79	-2.61	1.0000	
12	16	-8	245	303
346	3.79	-2.61	1.0000	
10	16	-8	592	257
610	0.85	-5.67	1.0000	
10	16	-8	44	254
-69	0.85	-5.67	1.0000	
8	16	-8	190	211
139	1.83	0.31	1.0000	
6	16	-8	400	226
446	-2.86	-1.92	1.0000	
-6	-16	8	367	306
409	-2.86	1.92	1.0000	
4	16	-8	31	211
35	-1.45	0.33	1.0000	
-4	-16	8	321	279
339	-1.45	-0.33	1.0000	
2	16	-8	435	211
552	-5.49	0.77	1.0000	
-2	-16	8	528	273
660	-5.49	-0.77	1.0000	
0	16	-8	907	225
528	0.12	-8.72	1.0000	
0	-16	8	1056	260
649	0.12	8.72	1.0000	
-2	16	-8	859	243
816	0.78	-8.43	1.0000	
-2	16	-8	965	159
925	0.78	-8.43	1.0000	
2	-16	8	604	245
558	0.78	8.43	1.0000	
-4	16	-8	281	235
318	-1.10	-1.71	1.0000	
-4	16	-8	443	158
490	-1.10	-1.71	1.0000	
4	-16	8	300	239
340	-1.10	1.71	1.0000	
-6	16	-8	1175	185
854	-0.39	-6.49	1.0000	
-6	16	-8	1503	272

1141 -0.39 -6.49 1.0000
6 -16 8 1065 252
764 -0.39 6.49 1.0000
-8 16 -8 5 237
-66 -1.23 -1.55 1.0000
-8 16 -8 201 167
258 -1.23 -1.55 1.0000
8 -16 8 5 214
-121 -1.23 1.55 1.0000
-10 16 -8 73 180
193 -1.72 4.64 1.0000
-10 16 -8 234 269
428 -1.72 4.64 1.0000
10 -16 8 116 241
261 -1.72 -4.64 1.0000
-12 16 -8 15 182
-16 1.58 -2.98 1.0000
-12 16 -8 414 261
272 1.58 -2.98 1.0000
12 -16 8 388 270
251 1.58 2.98 1.0000
-14 16 -8 140 207
283 1.06 4.24 1.0000
14 -16 8 0 259
24 1.06 -4.24 1.0000
16 -16 8 288 311
347 -1.65 0.77 1.0000
17 15 -8 11 296
-79 1.12 2.60 1.0000
17 15 -8 1001 435
1218 1.12 2.60 1.0000
15 15 -8 3 288
-2 -1.35 0.75 1.0000
15 15 -8 827 367
910 -1.35 0.75 1.0000
13 15 -8 234 278
190 -0.27 3.70 1.0000
13 15 -8 527 295
468 -0.27 3.70 1.0000
11 15 -8 216 250
202 -0.04 1.38 1.0000
11 15 -8 529 275
508 -0.04 1.38 1.0000
9 15 -8 327 239

433	-0.89	6.97	1.0000	
9	15	-8	253	247
339	-0.89	6.97	1.0000	
7	15	-8	1088	247
1099	1.15	0.37	1.0000	
5	15	-8	224	204
298	6.08	0.37	1.0000	
-5	-15	8	322	274
425	6.08	-0.37	1.0000	
3	15	-8	392	199
358	3.04	-1.28	1.0000	
-3	-15	8	827	284
782	3.04	1.28	1.0000	
1	15	-8	900	240
953	6.93	2.89	1.0000	
-1	-15	8	1173	282
1251	6.93	-2.89	1.0000	
-1	15	-8	684	147
894	1.30	5.79	1.0000	
-1	15	-8	349	219
493	1.30	5.79	1.0000	
1	-15	8	487	251
663	1.30	-5.79	1.0000	
-3	15	-8	410	218
286	-0.33	6.20	1.0000	
-3	15	-8	51	130
-96	-0.33	6.20	1.0000	
3	-15	8	162	217
73	-0.33	-6.20	1.0000	
-5	15	-8	1430	341
1947	2.78	4.73	1.0000	
-5	15	-8	1368	215
1875	2.78	4.73	1.0000	
5	-15	8	912	266
1333	2.78	-4.73	1.0000	
-7	15	-8	533	239
556	-0.69	1.51	1.0000	
-7	15	-8	216	166
230	-0.69	1.51	1.0000	
7	-15	8	310	233
326	-0.69	-1.51	1.0000	
-9	15	-8	149	166
212	3.62	-2.07	1.0000	
-9	15	-8	23	223

-162	3.62	-2.07	1.0000	
9	-15	8	23	210
-104	3.62	2.07	1.0000	
-11	15	-8	31	167
66	1.49	1.72	1.0000	
-11	15	-8	211	242
293	1.49	1.72	1.0000	
11	-15	8	0	226
9	1.49	-1.72	1.0000	
-13	15	-8	193	245
399	-1.51	-5.61	1.0000	
-13	15	-8	27	187
118	-1.51	-5.61	1.0000	
13	-15	8	389	283
669	-1.51	5.61	1.0000	
-15	15	-8	39	191
56	-1.28	-0.78	1.0000	
15	-15	8	194	275
232	-1.28	0.78	1.0000	
17	-15	8	154	294
109	1.82	0.79	1.0000	
18	14	-8	744	454
1079	-2.07	4.37	1.0000	
18	14	-8	7	317
66	-2.07	4.37	1.0000	
16	14	-8	679	373
963	1.09	-4.21	1.0000	
16	14	-8	101	286
225	1.09	-4.21	1.0000	
14	14	-8	568	327
815	4.12	-4.91	1.0000	
14	14	-8	193	276
330	4.12	-4.91	1.0000	
12	14	-8	430	265
349	-1.51	-1.95	1.0000	
12	14	-8	657	281
557	-1.51	-1.95	1.0000	
10	14	-8	1108	258
753	1.80	-6.91	1.0000	
10	14	-8	1066	253
718	1.80	-6.91	1.0000	
8	14	-8	34	232
187	-0.21	-7.65	1.0000	
8	14	-8	133	233

388 -0.21 -7.65 1.0000
6 14 -8 595 217
496 -1.78 -0.68 1.0000
-6 -14 8 655 285
551 -1.78 0.68 1.0000
4 14 -8 474 206
537 -5.58 -2.05 1.0000
-4 -14 8 645 247
724 -5.58 2.05 1.0000
2 14 -8 1300 290
1452 -0.98 -1.54 1.0000
-2 -14 8 2025 307
2214 -0.98 1.54 1.0000
0 14 -8 3624 368
3695 -4.60 -1.79 1.0000
0 14 -8 3980 238
4055 -4.60 -1.79 1.0000
0 -14 8 3702 343
3772 -4.60 1.79 1.0000
-2 14 -8 1515 329
2386 2.19 -10.24 1.0000
-2 14 -8 1688 210
2605 2.19 -10.24 1.0000
2 -14 8 1340 304
2157 2.19 10.24 1.0000
-4 14 -8 1173 171
1080 1.87 -4.61 1.0000
-4 14 -8 1347 252
1249 1.87 -4.61 1.0000
4 -14 8 1065 247
972 1.87 4.61 1.0000
-6 14 -8 794 154
567 1.80 -3.98 1.0000
-6 14 -8 887 232
647 1.80 -3.98 1.0000
6 -14 8 272 210
145 1.80 3.98 1.0000
-8 14 -8 231 160
187 0.27 2.09 1.0000
-8 14 -8 304 216
253 0.27 2.09 1.0000
8 -14 8 286 206
237 0.27 -2.09 1.0000
-10 14 -8 24 220

27 0.04 1.65 1.0000
-10 14 -8 24 161
27 0.04 1.65 1.0000
10 -14 8 45 206
49 0.04 -1.65 1.0000
-12 14 -8 273 241
431 -1.03 4.40 1.0000
-12 14 -8 12 163
43 -1.03 4.40 1.0000
12 -14 8 27 216
-75 -1.03 -4.40 1.0000
-14 14 -8 134 190
244 3.98 4.93 1.0000
14 -14 8 276 266
437 3.98 -4.93 1.0000
-16 14 -8 211 191
238 0.66 0.55 1.0000
16 -14 8 224 284
252 0.66 -0.55 1.0000
-18 14 -8 131 206
140 -0.12 0.30 1.0000
18 -14 8 340 304
354 -0.12 -0.30 1.0000
17 13 -8 898 413
1061 1.61 -1.81 1.0000
17 13 -8 8 305
-30 1.61 -1.81 1.0000
15 13 -8 170 282
364 -1.42 5.60 1.0000
15 13 -8 684 352
1044 -1.42 5.60 1.0000
13 13 -8 616 304
844 -0.88 3.75 1.0000
13 13 -8 191 258
325 -0.88 3.75 1.0000
11 13 -8 635 261
1007 0.20 5.80 1.0000
11 13 -8 368 270
661 0.20 5.80 1.0000
9 13 -8 1590 362
2040 1.28 4.88 1.0000
9 13 -8 1332 298
1746 1.28 4.88 1.0000
7 13 -8 312 221

585	3.07	5.71	1.0000	
5	13	-8	1135	238
1014	3.96	-0.72	1.0000	
-5	-13	8	1820	283
1670	3.96	0.72	1.0000	
3	13	-8	1072	234
1090	8.53	-0.54	1.0000	
-3	-13	8	1276	257
1306	8.53	0.54	1.0000	
1	13	-8	1002	230
833	3.50	1.34	1.0000	
1	13	-8	1330	162
1136	3.50	1.34	1.0000	
-1	-13	8	1902	280
1670	3.50	-1.34	1.0000	
-1	13	-8	1660	313
1606	3.04	0.17	1.0000	
-1	13	-8	1870	179
1813	3.04	0.17	1.0000	
1	-13	8	1944	274
1886	3.04	-0.17	1.0000	
-3	13	-8	1734	248
1303	-0.43	4.80	1.0000	
-3	13	-8	1897	176
1445	-0.43	4.80	1.0000	
3	-13	8	1556	216
1149	-0.43	-4.80	1.0000	
-5	13	-8	1773	217
1927	-3.12	0.90	1.0000	
-5	13	-8	2127	322
2297	-3.12	0.90	1.0000	
5	-13	8	1656	288
1804	-3.12	-0.90	1.0000	
-7	13	-8	1610	266
1626	-0.96	0.64	1.0000	
-7	13	-8	1327	205
1341	-0.96	0.64	1.0000	
7	-13	8	1010	238
1023	-0.96	-0.64	1.0000	
-9	13	-8	1297	219
1490	-0.62	-2.16	1.0000	
-9	13	-8	1874	315
2105	-0.62	-2.16	1.0000	
9	-13	8	1357	264

1555	-0.62	2.16	1.0000	
-11	13	-8	226	173
404	2.15	-6.42	1.0000	
-11	13	-8	329	227
546	2.15	-6.42	1.0000	
11	-13	8	227	221
406	2.15	6.42	1.0000	
-13	13	-8	493	183
699	-0.81	-3.82	1.0000	
-13	13	-8	448	231
645	-0.81	-3.82	1.0000	
13	-13	8	106	232
210	-0.81	3.82	1.0000	
-15	13	-8	237	172
187	-0.41	-3.76	1.0000	
15	-13	8	405	264
345	-0.41	3.76	1.0000	
-17	13	-8	304	202
357	-2.68	0.66	1.0000	
17	-13	8	301	280
353	-2.68	-0.66	1.0000	
18	12	-8	110	314
221	-0.38	-4.17	1.0000	
18	12	-8	671	419
923	-0.38	-4.17	1.0000	
16	12	-8	1275	368
1015	1.69	2.94	1.0000	
16	12	-8	579	294
409	1.69	2.94	1.0000	
14	12	-8	388	290
574	-1.01	-4.40	1.0000	
14	12	-8	257	262
410	-1.01	-4.40	1.0000	
12	12	-8	118	222
316	3.09	-11.28	1.0000	
12	12	-8	97	252
260	3.09	-11.28	1.0000	
10	12	-8	525	234
386	2.18	-4.06	1.0000	
10	12	-8	223	226
130	2.18	-4.06	1.0000	
8	12	-8	300	236
530	1.07	-5.91	1.0000	
8	12	-8	418	212

687 1.07 -5.91 1.0000
6 12 -8 56 192
8 -2.32 -3.39 1.0000
4 12 -8 923 245
1269 -1.02 4.46 1.0000
4 12 -8 1029 161
1393 -1.02 4.46 1.0000
-4 -12 8 1224 282
1619 -1.02 -4.46 1.0000
2 12 -8 531 144
915 -6.53 -0.56 1.0000
2 12 -8 311 213
616 -6.53 -0.56 1.0000
-2 -12 8 661 207
1083 -6.53 0.56 1.0000
0 12 -8 532 207
491 4.71 -2.76 1.0000
0 12 -8 752 134
711 4.71 -2.76 1.0000
0 -12 8 846 186
804 4.71 2.76 1.0000
-2 12 -8 768 156
1024 -2.80 5.16 1.0000
-2 12 -8 556 223
772 -2.80 5.16 1.0000
2 -12 8 510 189
718 -2.80 -5.16 1.0000
-4 12 -8 3152 183
1598 9.89 -10.01 1.0000
-4 12 -8 3112 254
1570 9.89 -10.01 1.0000
4 -12 8 2996 248
1487 9.89 10.01 1.0000
-6 12 -8 431 159
338 3.61 1.58 1.0000
-6 12 -8 892 228
763 3.61 1.58 1.0000
6 -12 8 621 191
515 3.61 -1.58 1.0000
-8 12 -8 1342 178
902 5.51 1.75 1.0000
-8 12 -8 1409 236
957 5.51 1.75 1.0000
8 -12 8 1352 216

909 5.51 -1.75 1.0000
-10 12 -8 1159 231
1156 0.76 3.07 1.0000
-10 12 -8 925 185
921 0.76 3.07 1.0000
10 -12 8 1030 241
1029 0.76 -3.07 1.0000
-12 12 -8 70 159
158 3.01 11.30 1.0000
-12 12 -8 221 205
538 3.01 11.30 1.0000
12 -12 8 75 211
62 3.01 -11.30 1.0000
-14 12 -8 115 177
73 -1.59 2.02 1.0000
-14 12 -8 34 164
12 -1.59 2.02 1.0000
14 -12 8 237 229
176 -1.59 -2.02 1.0000
-16 12 -8 129 171
196 3.76 2.53 1.0000
16 -12 8 192 267
277 3.76 -2.53 1.0000
-18 12 -8 12 176
-40 2.33 1.90 1.0000
18 -12 8 97 281
80 2.33 -1.90 1.0000
19 11 -8 8 324
-399 2.38 0.12 1.0000
19 11 -8 521 405
631 2.38 0.12 1.0000
17 11 -8 438 255
389 -0.96 1.31 1.0000
17 11 -8 583 316
526 -0.96 1.31 1.0000
15 11 -8 18 219
50 1.65 -1.83 1.0000
15 11 -8 0 268
9 1.65 -1.83 1.0000
13 11 -8 247 226
434 2.22 6.41 1.0000
13 11 -8 278 239
477 2.22 6.41 1.0000
11 11 -8 210 194

231 -1.50 8.24 1.0000
11 11 -8 184 205
196 -1.50 8.24 1.0000
9 11 -8 10 195
18 -3.18 4.89 1.0000
9 11 -8 100 206
255 -3.18 4.89 1.0000
7 11 -8 187 183
182 0.60 1.25 1.0000
7 11 -8 150 208
145 0.60 1.25 1.0000
5 11 -8 1075 150
1080 3.44 -1.38 1.0000
5 11 -8 797 218
799 3.44 -1.38 1.0000
-5 -11 8 1304 239
1315 3.44 1.38 1.0000
3 11 -8 1096 228
983 3.89 -4.39 1.0000
3 11 -8 1285 158
1166 3.89 -4.39 1.0000
-3 -11 8 1673 231
1550 3.89 4.39 1.0000
1 11 -8 774 151
1197 4.33 -7.21 1.0000
1 11 -8 435 210
751 4.33 -7.21 1.0000
-1 -11 8 825 198
1270 4.33 7.21 1.0000
-1 11 -8 633 136
618 0.05 -0.89 1.0000
-1 11 -8 446 204
433 0.05 -0.89 1.0000
1 -11 8 582 160
568 0.05 0.89 1.0000
-3 11 -8 121 121
229 -3.49 -3.73 1.0000
-3 11 -8 35 176
-21 -3.49 -3.73 1.0000
3 -11 8 197 149
334 -3.49 3.73 1.0000
-5 11 -8 699 159
632 -8.72 1.58 1.0000
-5 11 -8 515 206

442 -8.72 1.58 1.0000
5 -11 8 500 177
426 -8.72 -1.58 1.0000
-7 11 -8 474 153
437 -4.28 -3.48 1.0000
-7 11 -8 839 227
804 -4.28 -3.48 1.0000
7 -11 8 338 172
298 -4.28 3.48 1.0000
-9 11 -8 722 204
770 -3.10 -3.63 1.0000
-9 11 -8 366 158
392 -3.10 -3.63 1.0000
9 -11 8 354 200
377 -3.10 3.63 1.0000
-11 11 -8 95 188
-46 -1.51 -8.33 1.0000
-11 11 -8 99 144
77 -1.51 -8.33 1.0000
11 -11 8 95 182
-161 -1.51 8.33 1.0000
-13 11 -8 673 223
1058 0.19 -5.86 1.0000
-13 11 -8 446 184
768 0.19 -5.86 1.0000
13 -11 8 411 244
722 0.19 5.86 1.0000
-15 11 -8 374 177
353 -0.09 -1.42 1.0000
15 -11 8 213 245
197 -0.09 1.42 1.0000
-17 11 -8 93 184
204 -1.94 -4.27 1.0000
17 -11 8 20 266
76 -1.94 4.27 1.0000
-19 11 -8 253 206
363 -1.55 3.18 1.0000
19 -11 8 130 284
210 -1.55 -3.18 1.0000
20 10 -8 4 322
-134 -1.71 -0.59 1.0000
20 10 -8 663 288
715 -1.71 -0.59 1.0000
18 10 -8 414 322

310 1.18 2.33 1.0000
18 10 -8 464 271
353 1.18 2.33 1.0000
16 10 -8 249 255
446 -1.69 -4.59 1.0000
16 10 -8 70 289
186 -1.69 -4.59 1.0000
14 10 -8 3 222
-141 0.15 -1.60 1.0000
14 10 -8 151 235
164 0.15 -1.60 1.0000
12 10 -8 492 234
490 0.73 -3.14 1.0000
12 10 -8 959 243
961 0.73 -3.14 1.0000
10 10 -8 42 217
368 4.99 -10.42 1.0000
10 10 -8 151 200
633 4.99 -10.42 1.0000
8 10 -8 2142 331
2035 4.26 0.97 1.0000
8 10 -8 2763 289
2645 4.26 0.97 1.0000
6 10 -8 331 189
199 5.23 2.12 1.0000
4 10 -8 376 130
529 -2.75 2.98 1.0000
4 10 -8 178 198
284 -2.75 2.98 1.0000
-4 -10 8 460 184
629 -2.75 -2.98 1.0000
2 10 -8 290 157
1089 3.78 13.59 1.0000
2 10 -8 365 234
1230 3.78 13.59 1.0000
-2 -10 8 450 221
1382 3.78 -13.59 1.0000
0 10 -8 597 137
786 -4.81 8.57 1.0000
0 10 -8 352 204
469 -4.81 8.57 1.0000
0 -10 8 845 174
1082 -4.81 -8.57 1.0000
-2 10 -8 2378 227

```
3516    13.04    -4.96  1.0000
  -2  10  -8      2115       326
3186    13.04    -4.96  1.0000
   2 -10   8      2500       268
3694    13.04     4.96  1.0000
  -4  10  -8        80       117
126      3.14     7.05  1.0000
  -4  10  -8        79       175
-2      3.14     7.05  1.0000
   4 -10   8       122       142
194      3.14    -7.05  1.0000
  -6  10  -8      5751       477
8237    14.25    -3.71  1.0000
  -6  10  -8      5404       399
7819    14.25    -3.71  1.0000
   6 -10   8      5364       436
7769    14.25     3.71  1.0000
  -8  10  -8      1607       300
1960     4.42    -0.35  1.0000
  -8  10  -8      1702       216
2065     4.42    -0.35  1.0000
   8 -10   8      1487       255
1827     4.42     0.35  1.0000
 -10  10  -8       390       216
1070     5.02    10.38  1.0000
 -10  10  -8       335       183
977      5.02    10.38  1.0000
  10 -10   8       173       213
676      5.02   -10.38  1.0000
 -12  10  -8       290       155
182      2.04     4.14  1.0000
 -12  10  -8       366       183
245      2.04     4.14  1.0000
  12 -10   8       423       215
292      2.04    -4.14  1.0000
 -14  10  -8      1043       181
704      1.84     6.90  1.0000
 -14  10  -8      1037       176
699      1.84     6.90  1.0000
  14 -10   8       917       238
596      1.84    -6.90  1.0000
 -16  10  -8       217       163
205      0.89     5.68  1.0000
  16 -10   8        81       246
```

```
55      0.89    -5.68  1.0000
  -18   10   -8        19       172
-1      1.90    -3.21  1.0000
   18 -10    8        55       258
47      1.90     3.21  1.0000
  -20   10   -8       104       196
117      0.65     0.01  1.0000
   20 -10    8        59       293
69      0.65    -0.01  1.0000
   21    9   -8       793       334
920      1.17    -1.51  1.0000
   21    9   -8        32       378
61      1.17    -1.51  1.0000
   19    9   -8       288       328
295    -0.19    -2.01  1.0000
   19    9   -8       337       289
345    -0.19    -2.01  1.0000
   17    9   -8         8       264
-199      0.99    -2.31  1.0000
   17    9   -8         8       260
-25      0.99    -2.31  1.0000
   15    9   -8        22       250
-1      3.54     1.99  1.0000
   15    9   -8       190       254
267      3.54     1.99  1.0000
   13    9   -8      1338       298
1517    -0.46     2.01  1.0000
   13    9   -8      1496       273
1685    -0.46     2.01  1.0000
   11    9   -8       448       211
472    -2.97     3.61  1.0000
   11    9   -8       229       243
237    -2.97     3.61  1.0000
    9    9   -8      1633       206
1260    -2.41     5.06  1.0000
    9    9   -8      1347       256
1009    -2.41     5.06  1.0000
    7    9   -8      1987       346
2539    -7.70    -2.68  1.0000
    7    9   -8      2226       272
2812    -7.70    -2.68  1.0000
    5    9   -8        68       192
121    -2.47    -5.37  1.0000
   -5   -9    8        78       174
```

134	-2.47	5.37	1.0000	
3	9	-8	412	148
1025	2.53	-11.43	1.0000	
-3	-9	8	610	196
1333	2.53	11.43	1.0000	
1	9	-8	852	171
2046	0.05	-14.43	1.0000	
1	9	-8	565	275
1581	0.05	-14.43	1.0000	
-1	-9	8	674	268
1763	0.05	14.43	1.0000	
-1	-9	8	857	210
2054	0.05	14.43	1.0000	
-1	9	-8	3182	186
2444	-1.96	-8.79	1.0000	
-1	9	-8	2382	295
1742	-1.96	-8.79	1.0000	
1	-9	8	3393	216
2637	-1.96	8.79	1.0000	
-3	9	-8	210	189
351	-9.51	-3.43	1.0000	
-3	9	-8	428	134
677	-9.51	-3.43	1.0000	
3	-9	8	447	147
718	-9.51	3.43	1.0000	
-5	9	-8	489	129
213	-7.48	-1.71	1.0000	
-5	9	-8	78	173
-314	-7.48	-1.71	1.0000	
5	-9	8	286	132
86	-7.48	1.71	1.0000	
-7	9	-8	21	128
86	-11.98	0.56	1.0000	
-7	9	-8	191	179
-60	-11.98	0.56	1.0000	
7	-9	8	191	146
-54	-11.98	-0.56	1.0000	
-9	9	-8	2153	236
1721	-2.36	-5.09	1.0000	
-9	9	-8	1867	199
1465	-2.36	-5.09	1.0000	
9	-9	8	1805	218
1408	-2.36	5.09	1.0000	
-11	9	-8	188	162

397	-3.30	-4.93	1.0000	
-11	9	-8	91	156
244	-3.30	-4.93	1.0000	
11	-9	8	113	199
281	-3.30	4.93	1.0000	
-13	9	-8	1435	220
1874	1.27	-4.98	1.0000	
-13	9	-8	1344	253
1769	1.27	-4.98	1.0000	
13	-9	8	1342	319
1768	1.27	4.98	1.0000	
-15	9	-8	48	150
18	-0.88	-6.95	1.0000	
15	-9	8	204	226
274	-0.88	6.95	1.0000	
-17	9	-8	700	192
701	-1.33	2.63	1.0000	
17	-9	8	662	271
665	-1.33	-2.63	1.0000	
-19	9	-8	151	184
153	0.16	0.09	1.0000	
19	-9	8	41	282
42	0.16	-0.09	1.0000	
-21	9	-8	9	186
-42	-2.28	1.35	1.0000	
21	-9	8	9	300
-295	-2.28	-1.35	1.0000	
20	8	-8	253	314
191	1.86	-1.39	1.0000	
20	8	-8	7	373
-332	1.86	-1.39	1.0000	
18	8	-8	673	322
841	-2.02	2.30	1.0000	
18	8	-8	241	334
344	-2.02	2.30	1.0000	
16	8	-8	243	255
305	-1.00	1.57	1.0000	
16	8	-8	5	263
-81	-1.00	1.57	1.0000	
14	8	-8	302	250
248	0.22	-2.16	1.0000	
14	8	-8	472	249
405	0.22	-2.16	1.0000	
12	8	-8	967	265

600	5.50	-1.74	1.0000	
12	8	-8	856	221
513	5.50	-1.74	1.0000	
10	8	-8	1329	263
1656	4.43	0.43	1.0000	
10	8	-8	1164	291
1471	4.43	0.43	1.0000	
8	8	-8	923	268
1168	10.41	-2.21	1.0000	
8	8	-8	1144	229
1432	10.41	-2.21	1.0000	
6	8	-8	72	209
489	5.85	10.53	1.0000	
-6	-8	8	66	161
471	5.85	-10.53	1.0000	
4	8	-8	668	215
566	5.90	12.88	1.0000	
-4	-8	8	871	165
821	5.90	-12.88	1.0000	
-4	-8	8	577	159
475	5.90	-12.88	1.0000	
2	8	-8	1724	138
960	-1.41	10.56	1.0000	
-2	-8	8	1449	157
759	-1.41	-10.56	1.0000	
-2	-8	8	1826	155
1043	-1.41	-10.56	1.0000	
0	8	-8	58	131
636	8.31	16.21	1.0000	
0	-8	8	32	127
463	8.31	-16.21	1.0000	
0	-8	8	45	175
585	8.31	-16.21	1.0000	
-2	8	-8	3315	271
4811	2.27	9.99	1.0000	
-2	8	-8	3270	368
4756	2.27	9.99	1.0000	
2	-8	8	3217	313
4693	2.27	-9.99	1.0000	
2	-8	8	3652	283
5215	2.27	-9.99	1.0000	
-4	8	-8	312	163
-58	14.66	-4.43	1.0000	
-4	8	-8	730	110

157	14.66	-4.43	1.0000	
4	-8	8	809	122
202	14.66	4.43	1.0000	
-6	8	-8	586	169
1139	7.50	-3.78	1.0000	
-6	8	-8	516	206
1040	7.50	-3.78	1.0000	
6	-8	8	552	180
1093	7.50	3.78	1.0000	
-8	8	-8	1181	253
1476	10.25	2.20	1.0000	
-8	8	-8	1142	188
1430	10.25	2.20	1.0000	
8	-8	8	1085	227
1358	10.25	-2.20	1.0000	
-10	8	-8	3276	283
3149	4.29	-0.98	1.0000	
-10	8	-8	2533	234
2418	4.29	-0.98	1.0000	
10	-8	8	2516	276
2401	4.29	0.98	1.0000	
-12	8	-8	569	181
879	1.14	5.83	1.0000	
-12	8	-8	501	182
794	1.14	5.83	1.0000	
12	-8	8	227	209
428	1.14	-5.83	1.0000	
-14	8	-8	26	144
169	-0.15	7.75	1.0000	
-14	8	-8	13	154
122	-0.15	7.75	1.0000	
14	-8	8	61	221
254	-0.15	-7.75	1.0000	
-16	8	-8	376	171
304	1.73	-0.36	1.0000	
16	-8	8	4	223
-144	1.73	0.36	1.0000	
-18	8	-8	5	160
-129	-1.21	1.64	1.0000	
18	-8	8	5	250
-181	-1.21	-1.64	1.0000	
-20	8	-8	250	195
430	3.61	-2.31	1.0000	
20	-8	8	156	303

```
304      3.61      2.31  1.0000
  21    7   -8         0       373
-245      0.13    -0.13  1.0000
  21    7   -8       150       340
148      0.13    -0.13  1.0000
  19    7   -8       747       329
592    -0.28    -3.11  1.0000
  19    7   -8       151       349
84    -0.28    -3.11  1.0000
  17    7   -8        50       291
-164      4.57    -4.07  1.0000
  17    7   -8       252       295
342      4.57    -4.07  1.0000
  15    7   -8       121       268
132    -0.68    -1.61  1.0000
  15    7   -8       249       283
266    -0.68    -1.61  1.0000
  13    7   -8      1526       246
1537     -0.87    -0.65  1.0000
  13    7   -8       947       307
955    -0.87    -0.65  1.0000
  11    7   -8       443       275
399    -4.30      3.37  1.0000
  11    7   -8       228       188
185    -4.30      3.37  1.0000
   9    7   -8       192       152
-14   -12.00    -0.67  1.0000
   9    7   -8        11       245
127   -12.00    -0.67  1.0000
   7    7   -8       124       214
308    -6.25    -2.30  1.0000
   7    7   -8       186       150
406    -6.25    -2.30  1.0000
  -7   -7    8       117       166
297    -6.25      2.30  1.0000
   5    7   -8       493       235
957    -5.55   -13.52  1.0000
   3    7   -8      1088       318
2605     -5.30   -14.75  1.0000
   1    7   -8      2810       364
4288      2.66   -11.46  1.0000
   1    7   -8      3252       255
4832      2.66   -11.46  1.0000
  -1    7   -8      4497       313
```

3184	-5.68	-18.49	1.0000	
-1	7	-8	5211	228
3820	-5.68	-18.49	1.0000	
1	-7	8	5250	221
3845	-5.68	18.49	1.0000	
1	-7	8	5001	250
3623	-5.68	18.49	1.0000	
-3	7	-8	737	204
1139	-6.45	-1.67	1.0000	
-3	7	-8	828	155
1252	-6.45	-1.67	1.0000	
3	-7	8	927	165
1378	-6.45	1.67	1.0000	
-5	7	-8	1038	128
406	-10.87	3.29	1.0000	
-5	7	-8	730	172
220	-10.87	3.29	1.0000	
5	-7	8	933	125
338	-10.87	-3.29	1.0000	
-7	7	-8	78	175
228	-6.23	2.28	1.0000	
-7	7	-8	78	132
228	-6.23	2.28	1.0000	
7	-7	8	276	153
536	-6.23	-2.28	1.0000	
-9	7	-8	2900	298
3563	-7.78	2.54	1.0000	
-9	7	-8	2420	242
3024	-7.78	2.54	1.0000	
9	-7	8	2560	283
3176	-7.78	-2.54	1.0000	
-11	7	-8	586	168
590	0.78	-1.19	1.0000	
-11	7	-8	447	162
450	0.78	-1.19	1.0000	
11	-7	8	413	190
416	0.78	1.19	1.0000	
-13	7	-8	406	184
700	2.89	-5.64	1.0000	
-13	7	-8	497	156
820	2.89	-5.64	1.0000	
13	-7	8	333	218
604	2.89	5.64	1.0000	
-15	7	-8	1294	203

1298 1.10 -0.46 1.0000
15 -7 8 1199 273
1203 1.10 0.46 1.0000
-17 7 -8 568 178
557 1.40 -0.16 1.0000
17 -7 8 322 257
313 1.40 0.16 1.0000
-19 7 -8 154 173
107 -1.44 1.43 1.0000
19 -7 8 213 269
156 -1.44 -1.43 1.0000
-21 7 -8 237 200
369 -2.01 3.41 1.0000
21 -7 8 22 297
66 -2.01 -3.41 1.0000
22 6 -8 40 386
40 1.89 0.10 1.0000
22 6 -8 5 357
-616 1.89 0.10 1.0000
20 6 -8 30 349
85 -1.58 3.22 1.0000
20 6 -8 17 342
-309 -1.58 3.22 1.0000
18 6 -8 698 340
565 -1.66 -1.82 1.0000
18 6 -8 806 331
663 -1.66 -1.82 1.0000
16 6 -8 589 277
364 -0.43 6.43 1.0000
16 6 -8 803 301
541 -0.43 6.43 1.0000
14 6 -8 248 231
128 1.93 3.98 1.0000
14 6 -8 774 284
552 1.93 3.98 1.0000
12 6 -8 501 182
400 3.78 -1.63 1.0000
12 6 -8 775 273
652 3.78 -1.63 1.0000
10 6 -8 4419 492
6614 14.32 3.62 1.0000
10 6 -8 4080 382
6195 14.32 3.62 1.0000
8 6 -8 501 252

1015 7.40 3.83 1.0000
8 6 -8 437 152
922 7.40 3.83 1.0000
-8 -6 8 434 172
916 7.40 -3.83 1.0000
6 6 -8 1844 348
2656 8.80 5.01 1.0000
4 6 -8 70 220
445 6.50 18.69 1.0000
2 6 -8 5939 334
3224 5.75 16.82 1.0000
0 6 -8 176 110
98 -2.76 8.77 1.0000
0 6 -8 223 180
154 -2.76 8.77 1.0000
-2 6 -8 78 128
526 10.50 10.41 1.0000
-2 6 -8 32 170
280 10.50 10.41 1.0000
-4 6 -8 1933 243
2292 6.15 -6.06 1.0000
-4 6 -8 1986 188
2351 6.15 -6.06 1.0000
-4 6 -8 2035 276
2406 6.15 -6.06 1.0000
4 -6 8 2352 194
2750 6.15 6.06 1.0000
-6 6 -8 1742 197
2530 8.67 -5.00 1.0000
-6 6 -8 1355 278
2051 8.67 -5.00 1.0000
6 -6 8 1739 213
2521 8.67 5.00 1.0000
-8 6 -8 125 187
623 5.96 -10.61 1.0000
-8 6 -8 168 149
716 5.96 -10.61 1.0000
8 -6 8 172 160
726 5.96 10.61 1.0000
-10 6 -8 254 135
138 5.14 -2.10 1.0000
-10 6 -8 467 160
313 5.14 -2.10 1.0000
10 -6 8 424 169

277	5.14	2.10	1.0000	
-12	6	-8	484	140
303	-2.34	3.38	1.0000	
-12	6	-8	414	158
248	-2.34	3.38	1.0000	
12	-6	8	202	186
91	-2.34	-3.38	1.0000	
-14	6	-8	501	158
418	-1.72	0.51	1.0000	
14	-6	8	849	234
740	-1.72	-0.51	1.0000	
-16	6	-8	127	144
148	-2.76	1.96	1.0000	
16	-6	8	298	248
338	-2.76	-1.96	1.0000	
-18	6	-8	88	171
66	1.69	-1.47	1.0000	
18	-6	8	220	262
188	1.69	1.47	1.0000	
-20	6	-8	149	184
291	-1.56	-4.04	1.0000	
20	-6	8	25	263
-173	-1.56	4.04	1.0000	
-22	6	-8	132	192
139	2.87	-0.04	1.0000	
22	-6	8	56	312
56	2.87	0.04	1.0000	
21	5	-8	22	349
-352	-1.25	-3.83	1.0000	
21	5	-8	114	396
223	-1.25	-3.83	1.0000	
19	5	-8	29	334
-18	2.38	-4.04	1.0000	
19	5	-8	10	335
71	2.38	-4.04	1.0000	
17	5	-8	328	301
546	-0.04	-4.56	1.0000	
17	5	-8	104	315
239	-0.04	-4.56	1.0000	
15	5	-8	639	299
1002	2.90	-4.73	1.0000	
15	5	-8	1337	299
1845	2.90	-4.73	1.0000	
13	5	-8	1300	308

1443	-3.28		-1.00	1.0000
13	5	-8	1576	307
1734	-3.28		-1.00	1.0000
11	5	-8	946	268
889	-8.75		-1.62	1.0000
11	5	-8	870	194
810	-8.75		-1.62	1.0000
9	5	-8	595	232
296	-7.41		1.79	1.0000
-9	-5	8	77	149
-98	-7.41		-1.79	1.0000
7	5	-8	1488	222
707	-10.91		-3.19	1.0000
5	5	-8	2961	333
1954	-5.67		-7.11	1.0000
3	5	-8	1331	225
976	-2.12		-16.76	1.0000
1	5	-8	1815	308
2348	0.16		-17.25	1.0000
-1	5	-8	3989	318
3593	3.23		-2.13	1.0000
-1	5	-8	3938	222
3544	3.23		-2.13	1.0000
-3	5	-8	7325	433
7402	-5.59		-1.77	1.0000
-3	5	-8	7598	367
7677	-5.59		-1.77	1.0000
-5	5	-8	2398	208
1517	-5.61		6.93	1.0000
-5	5	-8	2269	237
1415	-5.61		6.93	1.0000
-5	5	-8	2523	169
1617	-5.61		6.93	1.0000
5	-5	8	2446	171
1557	-5.61		-6.93	1.0000
-7	5	-8	795	211
1421	-5.61		13.58	1.0000
-7	5	-8	547	160
1042	-5.61		13.58	1.0000
7	-5	8	628	184
1177	-5.61		-13.58	1.0000
-9	5	-8	420	146
586	-2.41		5.44	1.0000
-9	5	-8	422	176

589   -2.41   5.44 1.0000
9   -5   8   277   159
413   -2.41   -5.44 1.0000
-11   5   -8   812   170
822   3.41   1.28 1.0000
11   -5   8   801   193
808   3.41   -1.28 1.0000
-13   5   -8   1498   208
1365   3.98   0.66 1.0000
13   -5   8   1376   242
1249   3.98   -0.66 1.0000
-15   5   -8   291   158
393   6.12   -0.23 1.0000
15   -5   8   240   228
323   6.12   0.23 1.0000
-17   5   -8   197   153
258   0.96   1.54 1.0000
17   -5   8   4   229
-144   0.96   -1.54 1.0000
-19   5   -8   287   179
232   -0.94   3.33 1.0000
19   -5   8   133   248
88   -0.94   -3.33 1.0000
-21   5   -8   295   181
344   -0.55   1.09 1.0000
21   -5   8   269   299
316   -0.55   -1.09 1.0000
22   4   -8   1   380
-140   0.61   -0.16 1.0000
20   4   -8   0   338
-271   -0.19   0.11 1.0000
20   4   -8   0   335
-406   -0.19   0.11 1.0000
18   4   -8   41   312
99   -0.25   10.44 1.0000
18   4   -8   71   298
22   -0.25   10.44 1.0000
16   4   -8   336   300
382   -1.17   1.66 1.0000
16   4   -8   150   278
180   -1.17   1.66 1.0000
14   4   -8   1194   278
1095   1.90   4.63 1.0000
14   4   -8   731   278

647	1.90	4.63	1.0000	
12	4	-8	3900	380
2137	9.96	10.00	1.0000	
12	4	-8	3234	291
1652	9.96	10.00	1.0000	
10	4	-8	368	243
539	3.28	-7.02	1.0000	
10	4	-8	180	158
282	3.28	-7.02	1.0000	
8	4	-8	1524	213
660	14.84	4.28	1.0000	
6	4	-8	2006	336
2374	6.08	6.10	1.0000	
4	4	-8	1188	294
1515	4.43	1.78	1.0000	
2	4	-8	33	199
575	-2.86	20.54	1.0000	
0	4	-8	4864	426
6964	4.04	11.26	1.0000	
-2	4	-8	736	135
765	-3.85	-9.46	1.0000	
-2	4	-8	768	190
801	-3.85	-9.46	1.0000	
-4	4	-8	841	147
1116	4.52	-1.65	1.0000	
-4	4	-8	1000	209
1299	4.52	-1.65	1.0000	
-6	4	-8	119	158
202	6.46	-18.68	1.0000	
-6	4	-8	99	141
249	6.46	-18.68	1.0000	
-6	4	-8	70	131
473	6.46	-18.68	1.0000	
6	-4	8	74	127
549	6.46	18.68	1.0000	
-8	4	-8	762	143
692	5.95	-12.89	1.0000	
-8	4	-8	873	186
821	5.95	-12.89	1.0000	
8	-4	8	815	158
734	5.95	12.89	1.0000	
-10	4	-8	505	170
687	-2.80	-3.07	1.0000	
-10	4	-8	280	147

417	-2.80	-3.07	1.0000	
10	-4	8	504	172
686	-2.80	3.07	1.0000	
-12	4	-8	1140	200
1516	-0.93	-4.40	1.0000	
12	-4	8	1220	240
1608	-0.93	4.40	1.0000	
-14	4	-8	619	182
697	-5.66	2.10	1.0000	
14	-4	8	569	224
643	-5.66	-2.10	1.0000	
-16	4	-8	317	149
332	-1.53	-0.47	1.0000	
16	-4	8	250	232
263	-1.53	0.47	1.0000	
-18	4	-8	114	161
264	-2.82	-3.92	1.0000	
18	-4	8	31	228
-238	-2.82	3.92	1.0000	
-20	4	-8	78	166
60	3.65	0.97	1.0000	
20	-4	8	138	279
120	3.65	-0.97	1.0000	
-22	4	-8	16	171
-300	-2.50	-2.46	1.0000	
22	-4	8	16	301
-291	-2.50	2.46	1.0000	
21	3	-8	340	382
294	-1.03	-1.13	1.0000	
21	3	-8	3	358
-149	-1.03	-1.13	1.0000	
19	3	-8	49	317
-339	0.64	-6.03	1.0000	
19	3	-8	764	336
479	0.64	-6.03	1.0000	
17	3	-8	32	283
89	-0.05	-4.54	1.0000	
17	3	-8	465	288
669	-0.05	-4.54	1.0000	
15	3	-8	54	256
-7	-0.22	-6.39	1.0000	
15	3	-8	54	222
-193	-0.22	-6.39	1.0000	
13	3	-8	2361	264

1855	-0.28	-4.80	1.0000	
13	3	-8	1511	278
1112	-0.28	-4.80	1.0000	
11	3	-8	270	225
432	-3.41	3.77	1.0000	
11	3	-8	86	181
178	-3.41	3.77	1.0000	
9	3	-8	385	228
641	-9.69	3.38	1.0000	
7	3	-8	1301	306
1836	-6.46	1.75	1.0000	
5	3	-8	7230	450
7321	-5.48	1.84	1.0000	
3	3	-8	1456	266
1332	8.76	-6.59	1.0000	
1	3	-8	1254	276
1849	5.81	-14.59	1.0000	
-1	3	-8	2519	300
2695	4.25	1.52	1.0000	
-3	3	-8	1105	198
990	8.87	6.53	1.0000	
-3	3	-8	1035	137
918	8.87	6.53	1.0000	
-5	3	-8	1271	189
931	-2.22	16.94	1.0000	
-5	3	-8	1333	147
995	-2.22	16.94	1.0000	
5	-3	8	1219	133
857	-2.22	-16.94	1.0000	
-7	3	-8	1596	288
3355	-5.27	14.67	1.0000	
-7	3	-8	1831	240
3691	-5.27	14.67	1.0000	
7	-3	8	1803	250
3652	-5.27	-14.67	1.0000	
-9	3	-8	493	161
1151	2.49	11.41	1.0000	
-9	3	-8	403	183
1007	2.49	11.41	1.0000	
9	-3	8	298	177
831	2.49	-11.41	1.0000	
-11	3	-8	1415	191
1304	3.91	4.31	1.0000	
11	-3	8	1532	221

1412	3.91	-4.31	1.0000	
-13	3	-8	1121	186
1140	8.51	0.58	1.0000	
13	-3	8	977	228
987	8.51	-0.58	1.0000	
-15	3	-8	599	175
554	3.07	1.20	1.0000	
15	-3	8	587	235
544	3.07	-1.20	1.0000	
-17	3	-8	409	157
237	4.60	1.15	1.0000	
17	-3	8	432	248
256	4.60	-1.15	1.0000	
-19	3	-8	141	154
68	-2.97	1.53	1.0000	
19	-3	8	15	252
-153	-2.97	-1.53	1.0000	
-21	3	-8	148	190
171	-0.27	0.90	1.0000	
21	-3	8	247	304
276	-0.27	-0.90	1.0000	
22	2	-8	3	357
-138	-1.23	-0.66	1.0000	
20	2	-8	20	318
-320	0.00	3.84	1.0000	
20	2	-8	305	336
476	0.00	3.84	1.0000	
18	2	-8	107	294
135	0.61	2.33	1.0000	
18	2	-8	485	298
549	0.61	2.33	1.0000	
16	2	-8	1064	292
1039	0.81	8.57	1.0000	
16	2	-8	653	272
611	0.81	8.57	1.0000	
14	2	-8	1423	323
2260	2.20	10.18	1.0000	
14	2	-8	1474	379
2325	2.20	10.18	1.0000	
12	2	-8	737	250
992	-2.83	-5.20	1.0000	
12	2	-8	697	218
944	-2.83	-5.20	1.0000	
10	2	-8	2471	352

3657	13.04	4.97	1.0000	
10	2	-8	2431	281
3607	13.04	4.97	1.0000	
-10	-2	8	2226	259
3325	13.04	-4.97	1.0000	
8	2	-8	3715	384
5274	2.35	-9.88	1.0000	
6	2	-8	148	200
712	10.58	-10.30	1.0000	
4	2	-8	820	202
874	-3.82	9.52	1.0000	
2	2	-8	753	164
540	-4.23	-2.77	1.0000	
0	2	-8	4518	367
5636	-7.45	3.59	1.0000	
-2	2	-8	733	173
520	-4.13	2.84	1.0000	
-2	2	-8	438	104
274	-4.13	2.84	1.0000	
-4	2	-8	38	172
443	-3.07	-20.56	1.0000	
-4	2	-8	34	119
501	-3.07	-20.56	1.0000	
-6	2	-8	5149	208
2642	5.87	-16.83	1.0000	
-6	2	-8	5034	274
2560	5.87	-16.83	1.0000	
-6	2	-8	5107	207
2612	5.87	-16.83	1.0000	
6	-2	8	5769	216
3090	5.87	16.83	1.0000	
-8	2	-8	1395	168
721	-1.43	-10.53	1.0000	
-8	2	-8	1799	159
1024	-1.43	-10.53	1.0000	
8	-2	8	1597	163
866	-1.43	10.53	1.0000	
-10	2	-8	369	175
1238	3.84	-13.57	1.0000	
-10	2	-8	330	216
1165	3.84	-13.57	1.0000	
10	-2	8	434	226
1356	3.84	13.57	1.0000	
-12	2	-8	385	156

720 -6.61 0.46 1.0000
12 -2 8 410 203
755 -6.61 -0.46 1.0000
-14 2 -8 1275 210
1427 -1.00 1.54 1.0000
14 -2 8 1252 267
1402 -1.00 -1.54 1.0000
-16 2 -8 589 179
723 -5.49 -0.66 1.0000
16 -2 8 450 249
564 -5.49 0.66 1.0000
-18 2 -8 411 174
230 1.83 4.03 1.0000
18 -2 8 26 237
-208 1.83 -4.03 1.0000
-20 2 -8 24 174
-50 -0.99 -4.10 1.0000
20 -2 8 234 292
222 -0.99 4.10 1.0000
-22 2 -8 7 190
-79 2.30 -0.06 1.0000
22 -2 8 7 308
-149 2.30 0.06 1.0000
21 1 -8 12 332
-329 2.11 -2.08 1.0000
21 1 -8 12 336
-445 2.11 -2.08 1.0000
19 1 -8 20 294
-264 -3.87 -0.50 1.0000
19 1 -8 6 314
47 -3.87 -0.50 1.0000
17 1 -8 830 297
1322 2.17 -6.21 1.0000
17 1 -8 435 271
807 2.17 -6.21 1.0000
15 1 -8 518 248
701 1.35 -5.71 1.0000
15 1 -8 924 259
1174 1.35 -5.71 1.0000
13 1 -8 1830 289
1778 3.16 -0.11 1.0000
13 1 -8 2480 342
2421 3.16 -0.11 1.0000
11 1 -8 693 210

675 0.09 0.94 1.0000
11 1 -8 632 179
615 0.09 0.94 1.0000
9 1 -8 3404 306
2626 -1.76 8.85 1.0000
7 1 -8 5113 320
3745 -5.77 18.35 1.0000
5 1 -8 4312 319
3897 3.17 2.16 1.0000
3 1 -8 2120 255
2278 4.31 -1.49 1.0000
1 1 -8 3139 236
1579 16.09 13.37 1.0000
-1 1 -8 2711 233
1277 16.14 -13.39 1.0000
-3 1 -8 776 145
1231 5.85 14.79 1.0000
-3 1 -8 751 234
1193 5.85 14.79 1.0000
-5 1 -8 1706 267
2232 0.24 17.28 1.0000
-5 1 -8 1647 185
2157 0.24 17.28 1.0000
-5 1 -8 1650 171
2161 0.24 17.28 1.0000
-7 1 -8 3003 266
4520 2.50 11.41 1.0000
-7 1 -8 2313 258
3660 2.50 11.41 1.0000
-7 1 -8 3110 313
4652 2.50 11.41 1.0000
7 -1 8 3100 278
4641 2.50 -11.41 1.0000
-9 1 -8 625 228
1675 -0.02 14.37 1.0000
-9 1 -8 473 183
1417 -0.02 14.37 1.0000
9 -1 8 950 231
2190 -0.02 -14.37 1.0000
-11 1 -8 669 177
1063 4.22 7.29 1.0000
11 -1 8 527 205
870 4.22 -7.29 1.0000
-13 1 -8 1379 182

1171 3.60 -1.44 1.0000
13 -1 8 1554 251
1334 3.60 1.44 1.0000
-15 1 -8 711 181
752 6.93 -2.94 1.0000
15 -1 8 801 252
844 6.93 2.94 1.0000
-17 1 -8 1125 204
1234 -1.21 -1.29 1.0000
17 -1 8 737 272
826 -1.21 1.29 1.0000
-19 1 -8 244 173
257 2.32 -0.23 1.0000
19 -1 8 267 271
281 2.32 0.23 1.0000
-21 1 -8 287 200
438 -4.61 1.27 1.0000
21 -1 8 227 310
359 -4.61 -1.27 1.0000
22 0 -8 119 360
134 0.81 0.80 1.0000
20 0 -8 3 316
-9 1.05 1.17 1.0000
20 0 -8 3 289
-1 1.05 1.17 1.0000
18 0 -8 382 281
335 -1.05 0.44 1.0000
18 0 -8 251 255
213 -1.05 0.44 1.0000
16 0 -8 761 242
419 0.21 8.70 1.0000
14 0 -8 3309 368
3370 -4.63 1.77 1.0000
14 0 -8 3018 336
3075 -4.63 1.77 1.0000
12 0 -8 732 204
683 4.73 2.63 1.0000
12 0 -8 878 229
828 4.73 2.63 1.0000
10 0 -8 1117 276
1409 -4.78 -8.61 1.0000
10 0 -8 714 182
927 -4.78 -8.61 1.0000
-10 0 8 677 156

890	-4.78	8.61	1.0000	
8	0	-8	31	179
455	8.34	-16.14	1.0000	
6	0	-8	229	163
167	-2.80	-8.64	1.0000	
4	0	-8	5160	417
7297	4.11	-11.13	1.0000	
2	0	-8	4161	331
5217	-7.45	-3.45	1.0000	
0	0	-8	778	236
1972	-14.31	0.05	1.0000	
-2	0	-8	4460	339
5590	-7.67	-3.56	1.0000	
-4	0	-8	5202	401
7368	4.14	-11.23	1.0000	
-4	0	-8	5033	358
7166	4.14	-11.23	1.0000	
-6	0	-8	331	156
284	-2.84	-8.74	1.0000	
-6	0	-8	218	110
154	-2.84	-8.74	1.0000	
-6	0	-8	117	111
24	-2.84	-8.74	1.0000	
6	0	8	178	119
98	-2.84	8.74	1.0000	
-8	0	-8	31	164
386	8.40	-16.20	1.0000	
-8	0	-8	34	140
494	8.40	-16.20	1.0000	
8	0	8	45	151
562	8.40	16.20	1.0000	
-10	0	-8	522	152
688	-4.77	-8.61	1.0000	
10	0	8	571	184
758	-4.77	8.61	1.0000	
-12	0	-8	944	163
904	4.76	2.83	1.0000	
12	0	8	910	200
871	4.76	-2.83	1.0000	
-14	0	-8	3299	267
3365	-4.62	1.81	1.0000	
14	0	8	3666	361
3739	-4.62	-1.81	1.0000	
-16	0	-8	988	170

593	0.07	8.71	1.0000	
16	0	8	703	238
368	0.07	-8.71	1.0000	
-18	0	-8	558	169
503	-1.12	0.29	1.0000	
18	0	8	223	261
189	-1.12	-0.29	1.0000	
-20	0	-8	174	171
166	1.01	1.18	1.0000	
20	0	8	140	284
134	1.01	-1.18	1.0000	
-22	0	-8	263	207
287	0.81	0.86	1.0000	
22	0	8	2	303
-526	0.81	-0.86	1.0000	
21	-1	-8	32	327
-326	-4.74	1.25	1.0000	
21	-1	-8	32	311
-288	-4.74	1.25	1.0000	
19	-1	-8	7	263
-110	2.29	-0.21	1.0000	
19	-1	-8	7	291
-59	2.29	-0.21	1.0000	
17	-1	-8	747	271
833	-1.22	-1.24	1.0000	
17	-1	-8	622	267
700	-1.22	-1.24	1.0000	
15	-1	-8	820	244
869	6.90	-2.94	1.0000	
15	-1	-8	786	242
832	6.90	-2.94	1.0000	
13	-1	-8	1759	230
1543	3.62	-1.22	1.0000	
13	-1	-8	1433	247
1237	3.62	-1.22	1.0000	
11	-1	-8	585	211
973	4.51	7.30	1.0000	
11	-1	-8	471	178
819	4.51	7.30	1.0000	
9	-1	-8	785	274
1949	0.00	14.52	1.0000	
7	-1	-8	2756	325
4222	2.73	11.49	1.0000	
5	-1	-8	2166	277

2791	0.18	17.16	1.0000	
3	-1	-8	829	210
1288	5.62	14.70	1.0000	
1	-1	-8	2558	208
1168	16.14	-13.56	1.0000	
-1	-1	-8	2857	213
1366	16.21	13.65	1.0000	
-3	-1	-8	2427	237
2591	4.25	-1.44	1.0000	
-5	-1	-8	4211	281
3801	3.14	2.16	1.0000	
-5	-1	-8	4091	240
3687	3.14	2.16	1.0000	
-7	-1	-8	4537	233
3197	-5.66	18.64	1.0000	
-7	-1	-8	5385	284
3951	-5.66	18.64	1.0000	
7	1	8	5005	259
3622	-5.66	-18.64	1.0000	
-9	-1	-8	2846	209
2159	-2.04	8.78	1.0000	
9	1	8	3195	256
2462	-2.04	-8.78	1.0000	
-11	-1	-8	601	157
592	-0.01	0.77	1.0000	
11	1	8	703	207
692	-0.01	-0.77	1.0000	
-13	-1	-8	1599	207
1542	2.94	-0.22	1.0000	
13	1	8	2223	332
2158	2.94	0.22	1.0000	
-15	-1	-8	491	177
669	1.29	-5.84	1.0000	
15	1	8	621	246
822	1.29	5.84	1.0000	
-17	-1	-8	748	210
1213	2.12	-6.15	1.0000	
17	1	8	551	292
958	2.12	6.15	1.0000	
-19	-1	-8	17	173
73	-3.91	-0.51	1.0000	
19	1	8	21	281
-48	-3.91	0.51	1.0000	
-21	-1	-8	41	184

82	2.14	-2.16	1.0000	
21	1	8	12	320
-9	2.14	2.16	1.0000	
22	-2	-8	7	346
-203	2.23	-0.09	1.0000	
22	-2	-8	7	326
-275	2.23	-0.09	1.0000	
20	-2	-8	23	267
-23	-0.95	-4.05	1.0000	
20	-2	-8	23	299
-221	-0.95	-4.05	1.0000	
18	-2	-8	143	252
45	2.04	4.08	1.0000	
18	-2	-8	28	237
-77	2.04	4.08	1.0000	
16	-2	-8	242	219
322	-5.41	-0.82	1.0000	
16	-2	-8	432	258
549	-5.41	-0.82	1.0000	
14	-2	-8	2268	303
2463	-0.86	1.54	1.0000	
14	-2	-8	2089	337
2276	-0.86	1.54	1.0000	
12	-2	-8	685	196
1111	-6.50	0.56	1.0000	
10	-2	-8	406	203
1311	3.78	-13.68	1.0000	
10	-2	-8	482	262
1447	3.78	-13.68	1.0000	
-10	2	8	374	185
1255	3.78	13.68	1.0000	
8	-2	-8	1401	187
726	-1.44	-10.52	1.0000	
6	-2	-8	5188	265
2687	5.76	-16.73	1.0000	
4	-2	-8	40	149
435	-2.82	-20.65	1.0000	
2	-2	-8	481	141
303	-4.04	3.02	1.0000	
0	-2	-8	4209	325
5294	-7.64	3.51	1.0000	
-2	-2	-8	454	129
284	-4.03	-2.93	1.0000	
-4	-2	-8	989	150

1053	-3.94	9.47	1.0000	
-4	-2	-8	1038	199
1107	-3.94	9.47	1.0000	
-6	-2	-8	114	147
627	10.46	-10.42	1.0000	
-6	-2	-8	161	155
750	10.46	-10.42	1.0000	
-6	-2	-8	74	129
505	10.46	-10.42	1.0000	
6	2	8	44	135
388	10.46	10.42	1.0000	
-8	-2	-8	3017	286
4464	2.28	-10.08	1.0000	
-8	-2	-8	3615	319
5186	2.28	-10.08	1.0000	
8	2	8	3821	319
5432	2.28	10.08	1.0000	
-10	-2	-8	2249	250
3379	13.01	4.91	1.0000	
10	2	8	2558	300
3738	13.01	-4.91	1.0000	
-12	-2	-8	588	164
808	-2.76	-5.12	1.0000	
12	2	8	622	216
848	-2.76	5.12	1.0000	
-14	-2	-8	1386	232
2208	2.14	10.16	1.0000	
14	2	8	1734	366
2653	2.14	-10.16	1.0000	
-16	-2	-8	902	191
869	0.79	8.54	1.0000	
16	2	8	702	273
653	0.79	-8.54	1.0000	
-18	-2	-8	347	190
410	0.78	2.44	1.0000	
18	2	8	344	281
404	0.78	-2.44	1.0000	
-20	-2	-8	274	201
435	-0.02	3.80	1.0000	
20	2	8	117	307
229	-0.02	-3.80	1.0000	
-22	-2	-8	3	186
-100	-1.20	-0.68	1.0000	
22	2	8	3	338

-17	-1.20	0.68	1.0000	
21	-3	-8	1	293
-170	-0.29	0.96	1.0000	
21	-3	-8	61	337
77	-0.29	0.96	1.0000	
19	-3	-8	15	281
-4	-3.04	1.49	1.0000	
19	-3	-8	15	245
-27	-3.04	1.49	1.0000	
17	-3	-8	401	239
229	4.56	1.27	1.0000	
17	-3	-8	347	230
188	4.56	1.27	1.0000	
15	-3	-8	722	220
679	3.14	1.35	1.0000	
15	-3	-8	508	216
470	3.14	1.35	1.0000	
13	-3	-8	993	234
1000	8.44	0.62	1.0000	
13	-3	-8	1426	239
1454	8.44	0.62	1.0000	
11	-3	-8	1292	209
1177	3.93	4.45	1.0000	
11	-3	-8	1493	262
1373	3.93	4.45	1.0000	
9	-3	-8	461	156
1101	2.57	11.42	1.0000	
9	-3	-8	447	204
1078	2.57	11.42	1.0000	
-9	3	8	368	168
950	2.57	-11.42	1.0000	
7	-3	-8	1352	279
2998	-5.12	14.74	1.0000	
5	-3	-8	1205	182
864	-2.21	16.90	1.0000	
3	-3	-8	780	151
651	8.63	6.47	1.0000	
1	-3	-8	1934	221
2086	4.26	1.52	1.0000	
-1	-3	-8	703	187
1074	5.68	-14.76	1.0000	
-3	-3	-8	1171	184
1056	8.76	-6.37	1.0000	
-5	-3	-8	7071	369

7175	-5.48	1.92	1.0000	
-5	-3	-8	7453	359
7561	-5.48	1.92	1.0000	
-5	-3	-8	7304	386
7411	-5.48	1.92	1.0000	
5	3	8	7038	370
7143	-5.48	-1.92	1.0000	
-7	-3	-8	1059	208
1548	-6.55	1.70	1.0000	
-7	-3	-8	952	186
1418	-6.55	1.70	1.0000	
7	3	8	963	204
1427	-6.55	-1.70	1.0000	
-9	-3	-8	444	154
712	-9.53	3.51	1.0000	
9	3	8	531	180
816	-9.53	-3.51	1.0000	
-11	-3	-8	86	133
175	-3.46	3.69	1.0000	
11	3	8	104	178
202	-3.46	-3.69	1.0000	
-13	-3	-8	1866	197
1430	-0.37	-4.66	1.0000	
13	3	8	2210	274
1733	-0.37	4.66	1.0000	
-15	-3	-8	281	155
171	-0.30	-6.31	1.0000	
15	3	8	53	227
-34	-0.30	6.31	1.0000	
-17	-3	-8	120	173
232	-0.18	-4.65	1.0000	
17	3	8	16	254
3	-0.18	4.65	1.0000	
-19	-3	-8	49	182
-37	0.48	-6.07	1.0000	
19	3	8	663	303
387	0.48	6.07	1.0000	
-21	-3	-8	179	199
146	-0.88	-1.10	1.0000	
21	3	8	352	322
306	-0.88	1.10	1.0000	
22	-4	-8	16	296
-202	-2.43	-2.42	1.0000	
22	-4	-8	112	349

171	-2.43	-2.42	1.0000	
20	-4	-8	123	302
105	3.66	1.00	1.0000	
20	-4	-8	111	268
93	3.66	1.00	1.0000	
18	-4	-8	32	263
-62	-2.84	-4.00	1.0000	
18	-4	-8	32	226
-54	-2.84	-4.00	1.0000	
16	-4	-8	148	221
161	-1.47	-0.28	1.0000	
16	-4	-8	179	217
194	-1.47	-0.28	1.0000	
14	-4	-8	348	215
393	-5.45	1.98	1.0000	
14	-4	-8	625	207
701	-5.45	1.98	1.0000	
12	-4	-8	1057	231
1430	-0.98	-4.50	1.0000	
12	-4	-8	1039	278
1408	-0.98	-4.50	1.0000	
10	-4	-8	321	146
458	-2.71	-2.89	1.0000	
10	-4	-8	251	178
372	-2.71	-2.89	1.0000	
8	-4	-8	755	192
690	5.89	-12.96	1.0000	
6	-4	-8	81	156
323	6.55	-18.73	1.0000	
4	-4	-8	804	196
1073	4.48	-1.67	1.0000	
2	-4	-8	741	161
769	-3.89	-9.46	1.0000	
0	-4	-8	5098	399
7234	4.18	11.18	1.0000	
-2	-4	-8	47	136
388	-3.01	20.73	1.0000	
-4	-4	-8	961	191
1253	4.55	1.65	1.0000	
4	4	8	1020	165
1324	4.55	-1.65	1.0000	
-6	-4	-8	1949	200
2305	6.21	6.08	1.0000	
-6	-4	-8	2243	225

2630 6.21 6.08 1.0000
6 4 8 2159 235
2543 6.21 -6.08 1.0000
-8 -4 -8 867 126
238 14.50 4.34 1.0000
-8 -4 -8 533 125
62 14.50 4.34 1.0000
8 4 8 751 157
170 14.50 -4.34 1.0000
-10 -4 -8 78 130
-39 3.25 -6.94 1.0000
10 4 8 154 186
246 3.25 6.94 1.0000
-12 -4 -8 3228 203
1673 9.80 9.77 1.0000
12 4 8 3099 262
1581 9.80 -9.77 1.0000
-14 -4 -8 1052 186
960 1.85 4.60 1.0000
14 4 8 881 259
798 1.85 -4.60 1.0000
-16 -4 -8 484 183
531 -1.01 1.62 1.0000
16 4 8 334 260
371 -1.01 -1.62 1.0000
-18 -4 -8 94 196
308 -0.24 10.44 1.0000
18 4 8 129 284
383 -0.24 -10.44 1.0000
-20 -4 -8 55 192
57 -0.09 0.09 1.0000
20 4 8 0 291
-47 -0.09 -0.09 1.0000
-22 -4 -8 1 211
-6 0.72 -0.10 1.0000
22 4 8 1 306
-514 0.72 0.10 1.0000
21 -5 -8 2 315
-197 -0.56 1.05 1.0000
21 -5 -8 2 275
-223 -0.56 1.05 1.0000
19 -5 -8 17 246
-64 -0.92 3.42 1.0000
19 -5 -8 17 270

-98	-0.92		3.42	1.0000
17	-5	-8	1	240
10	0.95		1.60	1.0000
17	-5	-8	5	206
-253	0.95		1.60	1.0000
15	-5	-8	131	205
182	6.10		-0.34	1.0000
15	-5	-8	422	228
547	6.10		-0.34	1.0000
13	-5	-8	1600	230
1454	4.03		0.76	1.0000
13	-5	-8	1720	298
1569	4.03		0.76	1.0000
11	-5	-8	1556	280
1567	3.47		1.43	1.0000
11	-5	-8	1379	222
1388	3.47		1.43	1.0000
9	-5	-8	261	139
382	-2.32		5.36	1.0000
9	-5	-8	201	169
305	-2.32		5.36	1.0000
7	-5	-8	597	229
1124	-5.66		13.58	1.0000
5	-5	-8	3092	250
2056	-5.79		7.09	1.0000
3	-5	-8	6750	399
6857	-5.36		-1.92	1.0000
1	-5	-8	3962	273
3577	3.06		-2.09	1.0000
-1	-5	-8	1850	233
2396	0.27		-17.12	1.0000
-3	-5	-8	1245	158
877	-2.32		-17.00	1.0000
-3	-5	-8	1217	145
849	-2.32		-17.00	1.0000
-5	-5	-8	2599	201
1677	-5.74		-6.90	1.0000
-5	-5	-8	2877	178
1902	-5.74		-6.90	1.0000
-5	-5	-8	1968	197
1178	-5.74		-6.90	1.0000
5	5	8	2125	212
1299	-5.74		6.90	1.0000
-7	-5	-8	1232	136

532	-10.80	-3.30	1.0000	
-7	-5	-8	1131	147
465	-10.80	-3.30	1.0000	
7	5	8	1212	165
522	-10.80	3.30	1.0000	
-9	-5	-8	420	134
171	-7.42	1.68	1.0000	
9	5	8	342	165
118	-7.42	-1.68	1.0000	
-11	-5	-8	882	171
817	-8.69	-1.52	1.0000	
11	5	8	668	208
597	-8.69	1.52	1.0000	
-13	-5	-8	1645	217
1796	-3.04	-0.95	1.0000	
13	5	8	1589	287
1738	-3.04	0.95	1.0000	
-15	-5	-8	1304	228
1788	2.70	-4.64	1.0000	
15	5	8	1236	299
1708	2.70	4.64	1.0000	
-17	-5	-8	313	193
523	-0.23	-4.49	1.0000	
17	5	8	233	274
418	-0.23	4.49	1.0000	
-19	-5	-8	3	191
51	2.44	-4.03	1.0000	
19	5	8	128	289
278	2.44	4.03	1.0000	
-21	-5	-8	451	218
658	-1.44	-3.90	1.0000	
21	5	8	4	306
28	-1.44	3.90	1.0000	
22	-6	-8	11	335
-217	2.90	0.02	1.0000	
22	-6	-8	11	298
-288	2.90	0.02	1.0000	
20	-6	-8	14	258
73	-1.64	-3.99	1.0000	
20	-6	-8	9	296
61	-1.64	-3.99	1.0000	
18	-6	-8	408	270
364	1.79	-1.54	1.0000	
18	-6	-8	7	220

-72	1.79	-1.54	1.0000	
16	-6	-8	325	218
370	-2.72	2.01	1.0000	
16	-6	-8	220	218
254	-2.72	2.01	1.0000	
14	-6	-8	1266	227
1120	-1.82	0.61	1.0000	
14	-6	-8	1123	241
986	-1.82	0.61	1.0000	
12	-6	-8	1100	207
816	-2.38	3.36	1.0000	
12	-6	-8	1132	191
843	-2.38	3.36	1.0000	
10	-6	-8	1529	258
1258	5.24	-2.15	1.0000	
8	-6	-8	166	184
702	5.89	-10.45	1.0000	
8	-6	-8	229	146
826	5.89	-10.45	1.0000	
6	-6	-8	1551	215
2311	8.85	-5.08	1.0000	
6	-6	-8	1310	257
2009	8.85	-5.08	1.0000	
4	-6	-8	2029	263
2399	6.18	-6.07	1.0000	
2	-6	-8	75	154
512	10.50	10.34	1.0000	
0	-6	-8	173	131
91	-2.87	8.68	1.0000	
-2	-6	-8	5066	238
2587	5.84	16.79	1.0000	
-2	-6	-8	4496	206
2184	5.84	16.79	1.0000	
2	6	8	4444	203
2149	5.84	-16.79	1.0000	
-4	-6	-8	83	151
314	6.52	18.77	1.0000	
-4	-6	-8	70	139
426	6.52	18.77	1.0000	
4	6	8	83	138
315	6.52	-18.77	1.0000	
-6	-6	-8	1501	223
2238	8.71	5.08	1.0000	
-6	-6	-8	1539	200

2285	8.71	5.08	1.0000	
6	6	8	1285	241
1972	8.71	-5.08	1.0000	
-8	-6	-8	743	188
1366	7.58	3.87	1.0000	
-8	-6	-8	497	189
1022	7.58	3.87	1.0000	
8	6	8	670	206
1264	7.58	-3.87	1.0000	
-10	-6	-8	4549	386
6739	14.12	3.56	1.0000	
10	6	8	5386	455
7756	14.12	-3.56	1.0000	
-12	-6	-8	568	161
468	3.56	-1.58	1.0000	
12	6	8	243	225
172	3.56	1.58	1.0000	
-14	-6	-8	797	175
574	1.89	3.91	1.0000	
14	6	8	679	259
474	1.89	-3.91	1.0000	
-16	-6	-8	1576	213
1216	-0.38	6.38	1.0000	
16	6	8	1329	306
992	-0.38	-6.38	1.0000	
-18	-6	-8	914	209
762	-1.61	-1.94	1.0000	
18	6	8	656	294
528	-1.61	1.94	1.0000	
-20	-6	-8	107	209
202	-1.55	3.31	1.0000	
20	6	8	42	302
107	-1.55	-3.31	1.0000	
-22	-6	-8	176	223
181	1.99	0.17	1.0000	
22	6	8	131	338
134	1.99	-0.17	1.0000	
21	-7	-8	20	283
-79	-2.03	3.32	1.0000	
21	-7	-8	195	326
312	-2.03	3.32	1.0000	
19	-7	-8	6	272
-147	-1.46	1.43	1.0000	
19	-7	-8	6	230

-108	-1.46	1.43	1.0000	
17	-7	-8	332	228
319	1.38	-0.27	1.0000	
17	-7	-8	153	251
143	1.38	-0.27	1.0000	
15	-7	-8	1509	271
1525	1.00	-0.27	1.0000	
15	-7	-8	1632	320
1649	1.00	-0.27	1.0000	
13	-7	-8	445	229
764	3.20	-5.54	1.0000	
13	-7	-8	598	213
963	3.20	-5.54	1.0000	
11	-7	-8	1713	284
1709	0.69	-1.29	1.0000	
9	-7	-8	2656	254
3304	-7.68	2.70	1.0000	
9	-7	-8	2278	307
2877	-7.68	2.70	1.0000	
7	-7	-8	127	156
314	-6.31	2.27	1.0000	
7	-7	-8	141	131
338	-6.31	2.27	1.0000	
5	-7	-8	1210	128
522	-10.84	3.22	1.0000	
5	-7	-8	997	160
383	-10.84	3.22	1.0000	
5	-7	-8	1207	141
520	-10.84	3.22	1.0000	
3	-7	-8	891	166
1342	-6.58	-1.73	1.0000	
3	-7	-8	826	231
1261	-6.58	-1.73	1.0000	
3	-7	-8	934	170
1394	-6.58	-1.73	1.0000	
1	-7	-8	5568	301
4151	-5.71	-18.43	1.0000	
1	-7	-8	4941	258
3588	-5.71	-18.43	1.0000	
1	-7	-8	5225	245
3843	-5.71	-18.43	1.0000	
-1	-7	-8	3111	306
4650	2.55	-11.39	1.0000	
-1	-7	-8	2605	288

4024	2.55	-11.39	1.0000	
-1	-7	-8	2807	290
4275	2.55	-11.39	1.0000	
-3	-7	-8	1724	268
3527	-5.12	-14.63	1.0000	
3	7	8	1432	254
3104	-5.12	14.63	1.0000	
-5	-7	-8	782	209
1420	-5.72	-13.61	1.0000	
5	7	8	673	199
1248	-5.72	13.61	1.0000	
-7	-7	-8	152	148
353	-6.29	-2.25	1.0000	
-7	-7	-8	84	134
239	-6.29	-2.25	1.0000	
7	7	8	12	168
37	-6.29	2.25	1.0000	
-9	-7	-8	19	131
99	-11.91	-0.68	1.0000	
-9	-7	-8	189	139
-68	-11.91	-0.68	1.0000	
9	7	8	1	182
177	-11.91	0.68	1.0000	
-11	-7	-8	374	156
337	-4.19	3.50	1.0000	
11	7	8	462	231
428	-4.19	-3.50	1.0000	
-13	-7	-8	1435	206
1450	-0.95	-0.65	1.0000	
13	7	8	1131	279
1143	-0.95	0.65	1.0000	
-15	-7	-8	445	178
465	-0.67	-1.51	1.0000	
15	7	8	4	248
-140	-0.67	1.51	1.0000	
-17	-7	-8	260	193
355	4.61	-3.96	1.0000	
17	7	8	367	289
489	4.61	3.96	1.0000	
-19	-7	-8	496	217
375	-0.37	-3.06	1.0000	
19	7	8	714	326
566	-0.37	3.06	1.0000	
-21	-7	-8	156	227

158 0.00 -0.24 1.0000
21 7 8 35 332
36 0.00 0.24 1.0000
20 -8 -8 182 280
336 3.55 -2.22 1.0000
20 -8 -8 184 317
338 3.55 -2.22 1.0000
18 -8 -8 6 226
-191 -1.24 1.78 1.0000
18 -8 -8 6 258
-319 -1.24 1.78 1.0000
16 -8 -8 167 214
117 1.90 -0.38 1.0000
16 -8 -8 149 231
102 1.90 -0.38 1.0000
14 -8 -8 969 311
1551 -0.25 7.58 1.0000
12 -8 -8 347 199
594 1.05 5.90 1.0000
12 -8 -8 396 191
659 1.05 5.90 1.0000
10 -8 -8 2844 257
2720 4.22 -1.01 1.0000
10 -8 -8 2445 303
2328 4.22 -1.01 1.0000
8 -8 -8 1191 209
1494 10.49 2.21 1.0000
8 -8 -8 1099 265
1385 10.49 2.21 1.0000
6 -8 -8 452 200
950 7.48 -3.92 1.0000
6 -8 -8 753 175
1371 7.48 -3.92 1.0000
4 -8 -8 1060 150
347 14.66 -4.21 1.0000
4 -8 -8 742 125
159 14.66 -4.21 1.0000
4 -8 -8 773 155
176 14.66 -4.21 1.0000
2 -8 -8 3426 290
4942 2.37 9.96 1.0000
2 -8 -8 3789 350
5376 2.37 9.96 1.0000
2 -8 -8 2934 312

4348	2.37	9.96	1.0000	
0	-8	-8	39	183
312	8.40	16.14	1.0000	
0	-8	-8	35	153
346	8.40	16.14	1.0000	
0	-8	-8	33	147
417	8.40	16.14	1.0000	
-2	-8	-8	1650	172
906	-1.43	10.54	1.0000	
2	8	8	1280	152
637	-1.43	-10.54	1.0000	
-4	-8	-8	898	175
834	5.92	12.98	1.0000	
4	8	8	549	162
442	5.92	-12.98	1.0000	
-6	-8	-8	180	153
738	6.00	10.52	1.0000	
-6	-8	-8	84	151
523	6.00	10.52	1.0000	
6	8	8	74	187
495	6.00	-10.52	1.0000	
-8	-8	-8	1225	215
1523	10.32	-2.19	1.0000	
-8	-8	-8	1208	202
1503	10.32	-2.19	1.0000	
8	8	8	1377	259
1705	10.32	2.19	1.0000	
-10	-8	-8	1535	221
1881	4.40	0.38	1.0000	
10	8	8	1377	285
1706	4.40	-0.38	1.0000	
-12	-8	-8	1292	188
858	5.54	-1.79	1.0000	
12	8	8	849	257
506	5.54	1.79	1.0000	
-14	-8	-8	202	168
158	0.26	-2.19	1.0000	
14	8	8	210	249
165	0.26	2.19	1.0000	
-16	-8	-8	276	186
338	-1.31	1.43	1.0000	
16	8	8	5	259
-42	-1.31	-1.43	1.0000	
-18	-8	-8	751	224

927	-2.17	2.24	1.0000	
18	8	8	414	314
545	-2.17	-2.24	1.0000	
-20	-8	-8	8	222
-29	1.94	-1.44	1.0000	
20	8	8	8	298
-518	1.94	1.44	1.0000	
21	-9	-8	9	329
-56	-2.29	1.23	1.0000	
21	-9	-8	9	271
-289	-2.29	1.23	1.0000	
19	-9	-8	263	296
263	0.02	0.01	1.0000	
19	-9	-8	0	243
-130	0.02	0.01	1.0000	
17	-9	-8	454	269
456	-1.39	2.64	1.0000	
17	-9	-8	407	235
408	-1.39	2.64	1.0000	
15	-9	-8	66	218
-255	-0.83	-6.97	1.0000	
15	-9	-8	142	212
193	-0.83	-6.97	1.0000	
13	-9	-8	1963	315
2454	1.29	-4.83	1.0000	
13	-9	-8	1577	321
2020	1.29	-4.83	1.0000	
11	-9	-8	6	169
32	-3.19	-4.76	1.0000	
11	-9	-8	10	175
68	-3.19	-4.76	1.0000	
9	-9	-8	2728	300
2247	-2.45	-5.07	1.0000	
9	-9	-8	2453	244
1997	-2.45	-5.07	1.0000	
7	-9	-8	16	169
97	-11.92	0.71	1.0000	
7	-9	-8	24	140
79	-11.92	0.71	1.0000	
5	-9	-8	273	131
79	-7.35	-1.75	1.0000	
5	-9	-8	362	154
133	-7.35	-1.75	1.0000	
3	-9	-8	528	160

824	-9.68	-3.42	1.0000	
3	-9	-8	269	180
452	-9.68	-3.42	1.0000	
1	-9	-8	2861	268
2155	-1.86	-8.78	1.0000	
1	-9	-8	2813	226
2113	-1.86	-8.78	1.0000	
-1	-9	-8	912	259
2135	-0.07	-14.40	1.0000	
-1	-9	-8	771	250
1914	-0.07	-14.40	1.0000	
-3	-9	-8	323	176
873	2.50	-11.39	1.0000	
3	9	8	377	184
962	2.50	11.39	1.0000	
-5	-9	-8	422	169
588	-2.29	-5.43	1.0000	
5	9	8	544	176
728	-2.29	5.43	1.0000	
-7	-9	-8	2450	245
3059	-7.77	-2.60	1.0000	
-7	-9	-8	2466	271
3077	-7.77	-2.60	1.0000	
7	9	8	2061	315
2623	-7.77	2.60	1.0000	
-9	-9	-8	1958	225
1547	-2.39	5.07	1.0000	
-9	-9	-8	1530	199
1168	-2.39	5.07	1.0000	
9	9	8	1830	268
1435	-2.39	-5.07	1.0000	
-11	-9	-8	639	181
678	-3.02	3.51	1.0000	
11	9	8	399	225
427	-3.02	-3.51	1.0000	
-13	-9	-8	1280	213
1470	-0.63	2.13	1.0000	
13	9	8	1031	284
1202	-0.63	-2.13	1.0000	
-15	-9	-8	179	185
254	3.58	2.05	1.0000	
15	9	8	23	252
-78	3.58	-2.05	1.0000	
-17	-9	-8	185	189

163	1.00	-2.16	1.0000	
17	9	8	436	309
405	1.00	2.16	1.0000	
-19	-9	-8	114	220
115	-0.14	-2.00	1.0000	
19	9	8	174	343
174	-0.14	2.00	1.0000	
-21	-9	-8	101	239
148	1.11	-1.49	1.0000	
21	9	8	5	333
-148	1.11	1.49	1.0000	
20	-10	-8	343	318
374	0.79	-0.11	1.0000	
20	-10	-8	1	256
-52	0.79	-0.11	1.0000	
18	-10	-8	17	275
-43	1.96	-3.05	1.0000	
18	-10	-8	93	249
86	1.96	-3.05	1.0000	
16	-10	-8	44	219
-31	0.81	5.72	1.0000	
16	-10	-8	205	261
195	0.81	5.72	1.0000	
14	-10	-8	529	203
287	1.87	6.95	1.0000	
14	-10	-8	524	219
283	1.87	6.95	1.0000	
12	-10	-8	658	217
508	2.16	4.00	1.0000	
12	-10	-8	634	206
486	2.16	4.00	1.0000	
10	-10	-8	295	214
910	5.04	10.42	1.0000	
10	-10	-8	283	197
888	5.04	10.42	1.0000	
8	-10	-8	2103	259
2515	4.42	-0.48	1.0000	
8	-10	-8	2185	316
2605	4.42	-0.48	1.0000	
6	-10	-8	4717	385
6944	14.20	-3.46	1.0000	
6	-10	-8	4610	445
6814	14.20	-3.46	1.0000	
4	-10	-8	53	139

53 3.34 6.84 1.0000
4 -10 -8 65 174
87 3.34 6.84 1.0000
2 -10 -8 2481 274
3641 13.00 -4.91 1.0000
2 -10 -8 2237 315
3336 13.00 -4.91 1.0000
0 -10 -8 452 175
607 -4.73 8.62 1.0000
0 -10 -8 505 187
675 -4.73 8.62 1.0000
-2 -10 -8 447 255
1386 3.81 13.66 1.0000
-4 -10 -8 421 174
581 -2.74 2.97 1.0000
4 10 8 366 178
516 -2.74 -2.97 1.0000
-6 -10 -8 508 166
346 5.14 2.16 1.0000
-6 -10 -8 672 161
487 5.14 2.16 1.0000
6 10 8 191 187
88 5.14 -2.16 1.0000
-8 -10 -8 2793 281
2673 4.23 0.98 1.0000
-8 -10 -8 2674 245
2556 4.23 0.98 1.0000
8 10 8 2267 327
2157 4.23 -0.98 1.0000
-10 -10 -8 160 164
652 5.07 -10.39 1.0000
-10 -10 -8 221 184
773 5.07 -10.39 1.0000
10 10 8 87 255
490 5.07 10.39 1.0000
-12 -10 -8 880 204
884 0.67 -3.08 1.0000
12 10 8 622 253
622 0.67 3.08 1.0000
-14 -10 -8 12 176
12 0.00 -1.71 1.0000
14 10 8 4 229
-204 0.00 1.71 1.0000
-16 -10 -8 95 199

228 -1.73 -4.64 1.0000
16 10 8 34 281
124 -1.73 4.64 1.0000
-18 -10 -8 294 217
216 0.90 2.27 1.0000
18 10 8 139 311
87 0.90 -2.27 1.0000
-20 -10 -8 5 241
-249 -1.82 -0.63 1.0000
20 10 8 64 344
80 -1.82 0.63 1.0000
19 -11 -8 16 295
-87 -1.54 3.12 1.0000
19 -11 -8 89 272
154 -1.54 3.12 1.0000
17 -11 -8 38 246
117 -1.96 -4.47 1.0000
17 -11 -8 121 288
251 -1.96 -4.47 1.0000
15 -11 -8 423 247
401 -0.12 -1.27 1.0000
15 -11 -8 77 212
67 -0.12 -1.27 1.0000
13 -11 -8 316 215
588 0.07 -5.81 1.0000
13 -11 -8 384 226
681 0.07 -5.81 1.0000
11 -11 -8 92 188
60 -1.50 -8.23 1.0000
11 -11 -8 304 208
362 -1.50 -8.23 1.0000
9 -11 -8 606 186
639 -2.90 -3.57 1.0000
9 -11 -8 319 207
335 -2.90 -3.57 1.0000
7 -11 -8 447 191
409 -4.22 -3.41 1.0000
7 -11 -8 437 174
399 -4.22 -3.41 1.0000
5 -11 -8 950 219
888 -8.71 1.55 1.0000
5 -11 -8 818 177
753 -8.71 1.55 1.0000
3 -11 -8 33 174

-39 -3.37 -3.70 1.0000
3 -11 -8 37 149
94 -3.37 -3.70 1.0000
1 -11 -8 236 174
228 0.02 -0.79 1.0000
1 -11 -8 431 174
421 0.02 -0.79 1.0000
-1 -11 -8 593 195
968 4.38 -7.34 1.0000
-1 -11 -8 564 211
930 4.38 -7.34 1.0000
-3 -11 -8 1380 217
1264 3.96 -4.36 1.0000
3 11 8 1293 213
1185 3.96 4.36 1.0000
-5 -11 -8 1032 203
1041 3.40 -1.30 1.0000
5 11 8 1096 223
1108 3.40 1.30 1.0000
-7 -11 -8 613 182
622 0.85 1.16 1.0000
-7 -11 -8 695 189
704 0.85 1.16 1.0000
7 11 8 150 211
153 0.85 -1.16 1.0000
-9 -11 -8 23 172
109 -3.30 4.80 1.0000
-9 -11 -8 15 160
11 -3.30 4.80 1.0000
9 11 8 25 234
114 -3.30 -4.80 1.0000
-11 -11 -8 231 171
260 -1.53 8.23 1.0000
-11 -11 -8 93 162
-65 -1.53 8.23 1.0000
11 11 8 197 241
223 -1.53 -8.23 1.0000
-13 -11 -8 95 177
204 2.14 6.38 1.0000
13 11 8 32 249
11 2.14 -6.38 1.0000
-15 -11 -8 42 194
83 1.52 -1.75 1.0000
15 11 8 7 231

```
-135      1.52      1.75  1.0000
 -17 -11   -8       168       212
139    -0.89     1.23  1.0000
  17  11    8       168       300
139    -0.89    -1.23  1.0000
 -19 -11   -8       411       254
500     2.23     0.18  1.0000
  19  11    8        96       329
140     2.23    -0.18  1.0000
  18 -12   -8        46       247
31     2.30     1.94  1.0000
  18 -12   -8       243       301
222     2.30     1.94  1.0000
  16 -12   -8        58       256
100     3.88     2.67  1.0000
  16 -12   -8        56       235
97     3.88     2.67  1.0000
  14 -12   -8       262       211
202    -1.55     1.87  1.0000
  14 -12   -8         8       230
-52    -1.55     1.87  1.0000
  12 -12   -8       163       213
426     3.19    11.37  1.0000
  12 -12   -8        70       207
155     3.19    11.37  1.0000
  10 -12   -8      1269       247
1276      0.67     3.16  1.0000
  10 -12   -8       992       235
997     0.67     3.16  1.0000
   8 -12   -8      2858       307
2196      5.52     1.75  1.0000
   8 -12   -8      2017       233
1468      5.52     1.75  1.0000
   6 -12   -8       317       180
233     3.71     1.56  1.0000
   6 -12   -8       696       191
576     3.71     1.56  1.0000
   4 -12   -8      3646       262
1973      9.86    -9.78  1.0000
   4 -12   -8      2986       277
1496      9.86    -9.78  1.0000
   2 -12   -8       547       208
761    -2.81     5.09  1.0000
   2 -12   -8       812       207
```

1075	-2.81	5.09	1.0000	
0	-12	-8	661	203
615	4.77	-2.71	1.0000	
0	-12	-8	1147	220
1099	4.77	-2.71	1.0000	
-2	-12	-8	408	196
749	-6.56	-0.42	1.0000	
-4	-12	-8	1016	279
1377	-0.93	4.45	1.0000	
4	12	8	1224	243
1618	-0.93	-4.45	1.0000	
-6	-12	-8	560	185
360	-2.38	-3.40	1.0000	
6	12	8	23	201
-38	-2.38	3.40	1.0000	
-8	-12	-8	462	192
740	1.14	-5.80	1.0000	
-8	-12	-8	331	196
570	1.14	-5.80	1.0000	
8	12	8	359	226
609	1.14	5.80	1.0000	
-10	-12	-8	290	195
182	2.01	-4.09	1.0000	
-10	-12	-8	214	181
121	2.01	-4.09	1.0000	
10	12	8	331	252
217	2.01	4.09	1.0000	
-12	-12	-8	68	192
122	3.11	-11.40	1.0000	
12	12	8	80	257
211	3.11	11.40	1.0000	
-14	-12	-8	143	201
257	-1.15	-4.39	1.0000	
14	12	8	129	272
238	-1.15	4.39	1.0000	
-16	-12	-8	368	225
240	1.39	2.90	1.0000	
16	12	8	14	261
-147	1.39	-2.90	1.0000	
-18	-12	-8	360	260
554	-0.38	-4.27	1.0000	
18	12	8	3	280
18	-0.38	4.27	1.0000	
17	-13	-8	240	283

286 -2.76 0.65 1.0000
17 -13 -8 76 249
99 -2.76 0.65 1.0000
15 -13 -8 130 225
92 -0.32 -3.70 1.0000
15 -13 -8 280 256
230 -0.32 -3.70 1.0000
13 -13 -8 247 223
397 -0.96 -3.73 1.0000
13 -13 -8 251 248
403 -0.96 -3.73 1.0000
11 -13 -8 310 224
521 2.20 -6.42 1.0000
11 -13 -8 299 225
505 2.20 -6.42 1.0000
9 -13 -8 1586 298
1784 -0.47 -2.04 1.0000
9 -13 -8 1592 306
1790 -0.47 -2.04 1.0000
7 -13 -8 1668 256
1682 -0.85 0.58 1.0000
7 -13 -8 1592 292
1606 -0.85 0.58 1.0000
5 -13 -8 2082 308
2266 -3.15 1.04 1.0000
5 -13 -8 1804 278
1975 -3.15 1.04 1.0000
3 -13 -8 1498 227
1109 -0.23 4.70 1.0000
3 -13 -8 1631 215
1224 -0.23 4.70 1.0000
1 -13 -8 1743 293
1684 3.07 0.21 1.0000
1 -13 -8 1829 274
1769 3.07 0.21 1.0000
-1 -13 -8 1415 240
1212 3.72 1.32 1.0000
-1 -13 -8 1336 222
1139 3.72 1.32 1.0000
-3 -13 -8 1077 219
1087 8.41 -0.63 1.0000
-5 -13 -8 1151 219
1028 4.06 -0.73 1.0000
5 13 8 1081 243

960   4.06   0.73 1.0000
  -7 -13 -8   487   224
812   3.03   5.51 1.0000
  -7 -13 -8   477   202
799   3.03   5.51 1.0000
   7 13   8   398   234
692   3.03   -5.51 1.0000
  -9 -13 -8   1533   300
1973   1.22   4.85 1.0000
  -9 -13 -8   1308   242
1716   1.22   4.85 1.0000
   9 13   8   1223   281
1618   1.22   -4.85 1.0000
 -11 -13 -8   446   220
767   0.09   5.87 1.0000
 -11 -13 -8   425   218
739   0.09   5.87 1.0000
  11 13   8   290   280
556   0.09   -5.87 1.0000
 -13 -13 -8   326   213
495   -0.89   3.72 1.0000
  13 13   8   262   273
415   -0.89   -3.72 1.0000
 -15 -13 -8   88   221
236   -1.53   5.60 1.0000
  15 13   8   45   281
-199   -1.53   -5.60 1.0000
 -17 -13 -8   247   236
331   1.53   -1.76 1.0000
  17 13   8   40   294
77   1.53   1.76 1.0000
  18 -14 -8   149   262
159   -0.13   0.34 1.0000
  18 -14 -8   161   314
172   -0.13   0.34 1.0000
  16 -14 -8   148   276
170   0.69   0.40 1.0000
  16 -14 -8   172   249
196   0.69   0.40 1.0000
  14 -14 -8   29   255
16   4.17   5.07 1.0000
  14 -14 -8   39   224
80   4.17   5.07 1.0000
  12 -14 -8   79   212

166 -1.08 4.37 1.0000
12 -14 -8 27 227
-21 -1.08 4.37 1.0000
10 -14 -8 78 198
87 0.10 1.62 1.0000
10 -14 -8 150 215
164 0.10 1.62 1.0000
8 -14 -8 234 203
185 0.22 2.25 1.0000
8 -14 -8 202 179
156 0.22 2.25 1.0000
6 -14 -8 775 206
555 1.98 -3.95 1.0000
6 -14 -8 756 224
539 1.98 -3.95 1.0000
4 -14 -8 808 222
727 1.86 -4.69 1.0000
4 -14 -8 1079 249
990 1.86 -4.69 1.0000
2 -14 -8 1007 279
1716 2.14 -10.13 1.0000
2 -14 -8 1123 309
1870 2.14 -10.13 1.0000
0 -14 -8 3477 379
3540 -4.67 -1.76 1.0000
-2 -14 -8 1483 326
1640 -0.89 -1.52 1.0000
4 14 8 465 217
525 -5.52 2.03 1.0000
6 14 8 326 228
259 -1.80 0.40 1.0000
-8 -14 -8 120 193
368 -0.18 -7.73 1.0000
-8 -14 -8 58 213
248 -0.18 -7.73 1.0000
8 14 8 15 232
133 -0.18 7.73 1.0000
-10 -14 -8 773 222
479 1.91 -6.93 1.0000
-10 -14 -8 1382 210
990 1.91 -6.93 1.0000
10 14 8 1121 254
771 1.91 6.93 1.0000
-12 -14 -8 9 202

-159 -1.65 -1.95 1.0000
-12 -14 -8 9 208
-103 -1.65 -1.95 1.0000
12 14 8 116 271
75 -1.65 1.95 1.0000
-14 -14 -8 398 236
599 4.01 -5.07 1.0000
14 14 8 310 307
490 4.01 5.07 1.0000
-16 -14 -8 128 251
266 1.03 -4.27 1.0000
16 14 8 67 300
174 1.03 4.27 1.0000
-18 -14 -8 179 275
362 -2.35 4.30 1.0000
18 14 8 0 318
36 -2.35 -4.30 1.0000
17 -15 -8 241 295
187 1.71 -0.73 1.0000
17 -15 -8 96 250
63 1.71 -0.73 1.0000
15 -15 -8 3 271
-69 -1.39 -0.75 1.0000
15 -15 -8 3 229
-203 -1.39 -0.75 1.0000
13 -15 -8 6 231
46 -1.47 -5.59 1.0000
13 -15 -8 65 265
194 -1.47 -5.59 1.0000
11 -15 -8 161 208
239 1.68 1.80 1.0000
11 -15 -8 8 231
-35 1.68 1.80 1.0000
9 -15 -8 122 200
175 3.50 -2.05 1.0000
9 -15 -8 63 218
96 3.50 -2.05 1.0000
7 -15 -8 293 219
313 -0.62 1.56 1.0000
7 -15 -8 512 208
539 -0.62 1.56 1.0000
5 -15 -8 1242 273
1720 2.83 4.62 1.0000
5 -15 -8 1442 308

1954 2.83 4.62 1.0000
3 -15 -8 56 212
-77 -0.17 6.49 1.0000
1 -15 -8 311 240
448 1.35 5.76 1.0000
-1 -15 -8 869 279
913 6.90 2.99 1.0000
5 15 8 50 214
-224 6.15 -0.19 1.0000
-7 -15 -8 1124 231
1128 0.94 0.37 1.0000
7 15 8 1196 284
1201 0.94 -0.37 1.0000
-9 -15 -8 66 202
-90 -0.81 6.98 1.0000
9 15 8 288 241
387 -0.81 -6.98 1.0000
-11 -15 -8 222 219
204 -0.18 1.27 1.0000
-11 -15 -8 328 235
306 -0.18 1.27 1.0000
11 15 8 456 257
431 -0.18 -1.27 1.0000
-13 -15 -8 536 225
467 -0.45 3.76 1.0000
13 15 8 19 288
-161 -0.45 -3.76 1.0000
-15 -15 -8 3 240
-288 -1.30 0.75 1.0000
15 15 8 36 317
52 -1.30 -0.75 1.0000
-17 -15 -8 166 268
263 1.03 2.69 1.0000
17 15 8 1 310
20 1.03 -2.69 1.0000
16 -16 -8 256 267
313 -1.65 -0.79 1.0000
16 -16 -8 4 304
-142 -1.65 -0.79 1.0000
14 -16 -8 25 236
-35 1.11 4.21 1.0000
14 -16 -8 25 262
-199 1.11 4.21 1.0000
12 -16 -8 345 247

220 1.51 -2.89 1.0000
12 -16 -8 85 215
30 1.51 -2.89 1.0000
10 -16 -8 66 247
180 -1.65 4.60 1.0000
10 -16 -8 147 207
304 -1.65 4.60 1.0000
8 -16 -8 511 243
596 -1.08 -1.49 1.0000
8 -16 -8 319 217
386 -1.08 -1.49 1.0000
6 -16 -8 1570 249
1202 -0.42 -6.39 1.0000
6 -16 -8 1454 275
1099 -0.42 -6.39 1.0000
4 -16 -8 678 250
739 -1.13 -1.60 1.0000
2 -16 -8 651 249
602 0.83 -8.64 1.0000
0 -16 -8 1216 268
781 0.17 -8.69 1.0000
-2 -16 -8 289 288
377 -5.43 0.74 1.0000
6 16 8 79 240
96 -2.62 2.06 1.0000
-8 -16 -8 5 211
-144 1.81 0.42 1.0000
8 16 8 5 241
-69 1.81 -0.42 1.0000
-10 -16 -8 655 233
682 0.82 -5.77 1.0000
10 16 8 269 256
264 0.82 5.77 1.0000
-12 -16 -8 28 230
-107 3.82 -2.59 1.0000
-12 -16 -8 295 255
406 3.82 -2.59 1.0000
12 16 8 18 267
24 3.82 2.59 1.0000
-14 -16 -8 384 262
417 0.61 -0.50 1.0000
14 16 8 149 302
170 0.61 0.50 1.0000
-16 -16 -8 177 269

224 -1.75 0.72 1.0000
16 16 8 5 327
-171 -1.75 -0.72 1.0000
15 -17 -8 11 289
-94 1.02 -2.65 1.0000
15 -17 -8 278 264
398 1.02 -2.65 1.0000
13 -17 -8 8 224
-12 1.62 1.77 1.0000
13 -17 -8 8 272
-69 1.62 1.77 1.0000
11 -17 -8 3 208
-63 -0.96 -1.24 1.0000
11 -17 -8 3 255
-23 -0.96 -1.24 1.0000
9 -17 -8 8 208
-125 1.03 2.20 1.0000
9 -17 -8 114 243
95 1.03 2.20 1.0000
7 -17 -8 288 233
391 4.60 3.96 1.0000
7 -17 -8 202 248
283 4.60 3.96 1.0000
5 -17 -8 420 260
663 -0.10 4.56 1.0000
3 -17 -8 17 244
61 -0.13 4.54 1.0000
1 -17 -8 765 301
1244 2.18 6.26 1.0000
-1 -17 -8 694 295
779 -1.19 1.28 1.0000
-3 -17 -8 355 309
196 4.60 -1.20 1.0000
-7 -17 -8 475 237
461 1.42 0.25 1.0000
7 17 8 386 274
373 1.42 -0.25 1.0000
-9 -17 -8 683 240
692 -1.42 -2.60 1.0000
9 17 8 222 285
221 -1.42 2.60 1.0000
-11 -17 -8 265 251
446 -1.98 4.40 1.0000
11 17 8 31 257

-165 -1.98 -4.40 1.0000
-13 -17 -8 251 263
298 -2.70 -0.66 1.0000
-13 -17 -8 414 262
475 -2.70 -0.66 1.0000
13 17 8 254 297
301 -2.70 0.66 1.0000
-15 -17 -8 621 288
529 1.77 0.80 1.0000
15 17 8 120 312
81 1.77 -0.80 1.0000
14 -18 -8 31 258
-60 -2.20 -4.32 1.0000
14 -18 -8 4 303
56 -2.20 -4.32 1.0000
12 -18 -8 62 245
150 -0.37 4.22 1.0000
12 -18 -8 363 287
555 -0.37 4.22 1.0000
10 -18 -8 149 268
91 1.06 -2.33 1.0000
10 -18 -8 68 217
31 1.06 -2.33 1.0000
8 -18 -8 701 260
869 -2.08 -2.24 1.0000
8 -18 -8 708 282
877 -2.08 -2.24 1.0000
6 -18 -8 1131 288
960 -1.65 1.87 1.0000
4 -18 -8 268 282
641 -0.23 -10.49 1.0000
2 -18 -8 59 256
80 0.77 -2.34 1.0000
0 -18 -8 373 286
328 -1.13 -0.31 1.0000
-2 -18 -8 27 275
-253 1.93 -4.06 1.0000
-4 -18 -8 31 323
-46 -2.78 3.99 1.0000
6 18 8 126 250
99 1.74 -1.56 1.0000
-8 -18 -8 6 233
-222 -1.16 -1.74 1.0000
8 18 8 6 255

-401	-1.16	1.74	1.0000	
-10	-18	-8	492	265
504	1.94	3.15	1.0000	
10	18	8	18	287
-36	1.94	-3.15	1.0000	
-12	-18	-8	491	279
470	2.37	-1.98	1.0000	
12	18	8	205	285
185	2.37	1.98	1.0000	
-14	-18	-8	172	280
183	-0.15	-0.32	1.0000	
14	18	8	319	324
334	-0.15	0.32	1.0000	
11	-19	-8	66	248
105	2.31	-0.19	1.0000	
11	-19	-8	10	292
25	2.31	-0.19	1.0000	
9	-19	-8	187	275
188	-0.14	1.98	1.0000	
9	-19	-8	22	238
19	-0.14	1.98	1.0000	
7	-19	-8	256	270
169	-0.35	3.08	1.0000	
5	-19	-8	459	286
720	2.51	4.02	1.0000	
3	-19	-8	507	273
268	0.48	6.11	1.0000	
1	-19	-8	30	263
98	-3.91	0.47	1.0000	
-1	-19	-8	263	291
277	2.36	0.23	1.0000	
-3	-19	-8	355	325
236	-2.99	-1.46	1.0000	
-7	-19	-8	6	252
-50	-1.46	-1.43	1.0000	
7	19	8	383	266
306	-1.46	1.43	1.0000	
-9	-19	-8	209	267
211	0.10	-0.03	1.0000	
9	19	8	0	278
-125	0.10	0.03	1.0000	
-11	-19	-8	687	292
864	-1.51	-3.14	1.0000	
11	19	8	16	311

-28 -1.51 3.14 1.0000
10 -20 -8 5 230
-14 -1.79 0.66 1.0000
10 -20 -8 14 279
20 -1.79 0.66 1.0000
8 -20 -8 358 285
285 1.92 1.36 1.0000
6 -20 -8 424 303
602 -1.55 -3.31 1.0000
4 -20 -8 0 257
-180 -0.13 -0.06 1.0000
2 -20 -8 41 284
115 -0.04 -3.85 1.0000
0 -20 -8 3 294
-14 1.07 -1.18 1.0000
-2 -20 -8 24 301
-205 -0.94 4.13 1.0000
-4 -20 -8 97 338
79 3.67 -0.99 1.0000
-6 -20 -8 25 262
-45 -1.57 4.02 1.0000
-8 -20 -8 362 289
572 3.56 2.29 1.0000
8 20 8 236 296
410 3.56 -2.29 1.0000
-10 -20 -8 352 299
383 0.79 0.08 1.0000
10 20 8 1 305
-131 0.79 -0.08 1.0000
9 -21 -8 156 300
214 1.17 1.45 1.0000
7 -21 -8 4 297
4 0.03 0.21 1.0000
5 -21 -8 299 316
467 -1.40 3.84 1.0000
3 -21 -8 216 309
181 -0.93 1.06 1.0000
1 -21 -8 303 329
408 2.20 2.15 1.0000
-1 -21 -8 335 344
497 -4.77 -1.19 1.0000
-3 -21 -8 29 337
40 -0.24 -0.92 1.0000
-5 -21 -8 2 384

-150	-0.55	-1.06	1.0000	
-7	-21	-8	86	282
163	-1.92	-3.26	1.0000	
-9	-21	-8	130	307
208	-2.27	-1.30	1.0000	
9	21	8	9	295
-215	-2.27	1.30	1.0000	
6	-22	-8	397	323
406	1.97	-0.16	1.0000	
4	-22	-8	1	287
-254	0.68	0.08	1.0000	
2	-22	-8	3	330
-134	-1.23	0.72	1.0000	
0	-22	-8	194	347
214	0.83	-0.82	1.0000	
-2	-22	-8	276	352
243	2.29	0.06	1.0000	
-4	-22	-8	136	384
199	-2.45	2.46	1.0000	
-6	-22	-8	11	312
-87	2.88	0.06	1.0000	
-6	-22	-8	11	414
-215	2.88	0.06	1.0000	
4	21	-9	512	316
598	-0.90	1.82	1.0000	
-4	-21	9	436	379
516	-0.90	-1.82	1.0000	
2	21	-9	238	338
361	-3.06	-0.70	1.0000	
-2	-21	9	346	362
492	-3.06	0.70	1.0000	
0	21	-9	131	324
127	0.13	0.60	1.0000	
0	-21	9	1	321
-18	0.13	-0.60	1.0000	
-2	21	-9	19	338
5	1.95	-1.13	1.0000	
2	-21	9	7	292
-326	1.95	1.13	1.0000	
-4	21	-9	48	350
52	0.04	0.76	1.0000	
4	-21	9	1	308
-148	0.04	-0.76	1.0000	
7	20	-9	528	337

608 1.56 -0.41 1.0000
-7 -20 9 3 388
-24 1.56 0.41 1.0000
5 20 -9 415 299
461 0.03 -1.18 1.0000
-5 -20 9 2 347
-105 0.03 1.18 1.0000
3 20 -9 399 301
371 1.84 -0.47 1.0000
-3 -20 9 5 342
-12 1.84 0.47 1.0000
1 20 -9 211 290
244 -0.29 1.02 1.0000
-1 -20 9 21 338
32 -0.29 -1.02 1.0000
-1 20 -9 180 296
228 -3.17 -1.34 1.0000
1 -20 9 16 294
-118 -3.17 1.34 1.0000
-3 20 -9 229 322
299 -0.66 2.73 1.0000
3 -20 9 179 305
244 -0.66 -2.73 1.0000
-5 20 -9 122 335
197 -2.67 -0.18 1.0000
5 -20 9 10 297
-118 -2.67 0.18 1.0000
-7 20 -9 66 363
97 -1.70 0.40 1.0000
7 -20 9 65 290
95 -1.70 -0.40 1.0000
10 19 -9 369 326
396 -0.74 1.35 1.0000
-10 -19 9 330 412
355 -0.74 -1.35 1.0000
8 19 -9 262 287
381 -2.14 -2.28 1.0000
-8 -19 9 6 375
30 -2.14 2.28 1.0000
6 19 -9 84 265
113 -2.39 3.47 1.0000
-6 -19 9 256 345
318 -2.39 -3.47 1.0000
4 19 -9 352 287

508 -2.83 -2.03 1.0000

-4 -19 9 317 322

466 -2.83 2.03 1.0000

2 19 -9 221 269

282 -0.34 2.03 1.0000

-2 -19 9 143 312

193 -0.34 -2.03 1.0000

0 19 -9 100 271

149 2.77 -1.12 1.0000

0 -19 9 18 296

37 2.77 1.12 1.0000

-2 19 -9 470 305

470 -0.26 0.11 1.0000

2 -19 9 0 276

-125 -0.26 -0.11 1.0000

-4 19 -9 606 314

352 5.02 0.75 1.0000

-4 19 -9 34 183

-77 5.02 0.75 1.0000

4 -19 9 740 293

457 5.02 -0.75 1.0000

-6 19 -9 451 334

289 3.83 0.41 1.0000

-6 19 -9 152 207

65 3.83 0.41 1.0000

6 -19 9 382 281

235 3.83 -0.41 1.0000

-8 19 -9 10 209

24 0.10 1.50 1.0000

-8 19 -9 72 341

104 0.10 1.50 1.0000

8 -19 9 54 286

83 0.10 -1.50 1.0000

-10 19 -9 249 361

210 2.22 1.73 1.0000

10 -19 9 217 288

181 2.22 -1.73 1.0000

11 18 -9 242 349

346 1.32 -2.53 1.0000

9 18 -9 330 311

362 0.64 0.94 1.0000

-9 -18 9 2 380

-13 0.64 -0.94 1.0000

7 18 -9 327 280

365	0.46	-1.61	1.0000	
-7	-18	9	4	345
-115	0.46	1.61	1.0000	
5	18	-9	203	244
248	2.09	-0.89	1.0000	
-5	-18	9	7	330
-67	2.09	0.89	1.0000	
3	18	-9	175	245
194	0.59	0.26	1.0000	
-3	-18	9	1	288
-104	0.59	-0.26	1.0000	
1	18	-9	616	261
402	-3.88	-1.60	1.0000	
-1	-18	9	580	294
374	-3.88	1.60	1.0000	
-1	18	-9	64	267
78	-2.34	3.43	1.0000	
1	-18	9	23	276
-343	-2.34	-3.43	1.0000	
-3	18	-9	229	183
298	-4.87	-3.51	1.0000	
-3	18	-9	632	300
772	-4.87	-3.51	1.0000	
3	-18	9	442	286
553	-4.87	3.51	1.0000	
-5	18	-9	746	322
1047	-4.44	1.00	1.0000	
-5	18	-9	750	209
1052	-4.44	1.00	1.0000	
5	-18	9	292	278
490	-4.44	-1.00	1.0000	
-7	18	-9	316	206
383	-1.03	2.27	1.0000	
-7	18	-9	168	316
217	-1.03	2.27	1.0000	
7	-18	9	200	277
253	-1.03	-2.27	1.0000	
-9	18	-9	251	330
247	-1.50	0.00	1.0000	
-9	18	-9	111	211
108	-1.50	0.00	1.0000	
9	-18	9	3	267
-14	-1.50	0.00	1.0000	
-11	18	-9	184	210

226 0.13 -2.98 1.0000
-11 18 -9 12 335
-339 0.13 -2.98 1.0000
11 -18 9 259 304
308 0.13 2.98 1.0000
12 17 -9 504 339
523 1.88 2.54 1.0000
12 17 -9 409 299
425 1.88 2.54 1.0000
10 17 -9 785 294
776 0.27 -1.48 1.0000
8 17 -9 681 276
648 -0.30 1.39 1.0000
-8 -17 9 471 357
444 -0.30 -1.39 1.0000
6 17 -9 278 252
255 -0.16 -1.55 1.0000
-6 -17 9 59 323
49 -0.16 1.55 1.0000
4 17 -9 102 232
170 -1.16 2.97 1.0000
-4 -17 9 59 286
112 -1.16 -2.97 1.0000
2 17 -9 166 242
263 0.90 -3.98 1.0000
-2 -17 9 53 269
107 0.90 3.98 1.0000
0 17 -9 404 257
625 4.49 0.21 1.0000
0 -17 9 313 281
507 4.49 -0.21 1.0000
-2 17 -9 978 196
1539 7.03 0.35 1.0000
-2 17 -9 960 323
1516 7.03 0.35 1.0000
2 -17 9 793 302
1302 7.03 -0.35 1.0000
-4 17 -9 615 294
933 5.03 -0.48 1.0000
-4 17 -9 464 186
744 5.03 -0.48 1.0000
4 -17 9 284 266
511 5.03 0.48 1.0000
-6 17 -9 553 204

490 3.16 0.23 1.0000
-6 17 -9 338 291
287 3.16 0.23 1.0000
6 -17 9 549 262
486 3.16 -0.23 1.0000
-8 17 -9 163 188
269 3.64 0.83 1.0000
-8 17 -9 431 319
598 3.64 0.83 1.0000
8 -17 9 122 264
216 3.64 -0.83 1.0000
-10 17 -9 0 190
-136 0.01 0.36 1.0000
-10 17 -9 0 300
-127 0.01 0.36 1.0000
10 -17 9 0 244
-197 0.01 -0.36 1.0000
-12 17 -9 17 203
-158 1.59 3.18 1.0000
-12 17 -9 53 321
106 1.59 3.18 1.0000
12 -17 9 239 300
351 1.59 -3.18 1.0000
13 16 -9 646 364
1059 0.79 -6.12 1.0000
13 16 -9 238 318
509 0.79 -6.12 1.0000
11 16 -9 148 284
172 -4.20 1.66 1.0000
11 16 -9 297 297
342 -4.20 1.66 1.0000
9 16 -9 670 268
732 -0.62 -4.43 1.0000
7 16 -9 33 205
44 -1.07 -0.30 1.0000
-7 -16 9 67 301
82 -1.07 0.30 1.0000
5 16 -9 105 220
89 0.82 -0.76 1.0000
-5 -16 9 370 306
340 0.82 0.76 1.0000
3 16 -9 6 219
-93 -1.39 -1.68 1.0000
-3 -16 9 80 257

116   -1.39   1.68 1.0000
   1 16 -9   143   210
103   -1.40   2.68 1.0000
  -1 -16   9   305   268
249   -1.40   -2.68 1.0000
  -1 16 -9   143   150
113   -8.05   -2.81 1.0000
  -1 16 -9   297   237
311   -8.05   -2.81 1.0000
   1 -16   9   176   243
174   -8.05   2.81 1.0000
  -3 16 -9   1222   275
914   -6.79   2.22 1.0000
  -3 16 -9   753   187
507   -6.79   2.22 1.0000
   3 -16   9   738   252
492   -6.79   -2.22 1.0000
  -5 16 -9   334   179
476   -4.25   1.42 1.0000
  -5 16 -9   350   271
495   -4.25   1.42 1.0000
   5 -16   9   27   231
-35   -4.25   -1.42 1.0000
  -7 16 -9   231   271
203   -1.41   -1.36 1.0000
  -7 16 -9   330   188
297   -1.41   -1.36 1.0000
   7 -16   9   87   244
69   -1.41   1.36 1.0000
  -9 16 -9   712   311
841   1.84   0.92 1.0000
  -9 16 -9   216   196
289   1.84   0.92 1.0000
   9 -16   9   271   261
353   1.84   -0.92 1.0000
 -11 16 -9   329   306
439   0.01   2.51 1.0000
 -11 16 -9   3   194
20   0.01   2.51 1.0000
  11 -16   9   8   261
-64   0.01   -2.51 1.0000
 -13 16 -9   160   301
358   1.21   -5.31 1.0000
 -13 16 -9   25   200

127 1.21 -5.31 1.0000
13 -16 9 44 285
167 1.21 5.31 1.0000
14 15 -9 1470 403
1443 1.65 3.01 1.0000
14 15 -9 729 319
705 1.65 3.01 1.0000
12 15 -9 348 307
458 3.45 -0.30 1.0000
12 15 -9 274 292
372 3.45 -0.30 1.0000
10 15 -9 43 261
134 5.31 0.43 1.0000
8 15 -9 41 222
-40 5.28 1.77 1.0000
-8 -15 9 101 308
262 5.28 -1.77 1.0000
6 15 -9 233 230
236 0.96 -0.24 1.0000
-6 -15 9 107 287
109 0.96 0.24 1.0000
4 15 -9 452 213
345 0.87 -2.87 1.0000
-4 -15 9 12 270
-73 0.87 2.87 1.0000
2 15 -9 211 208
154 3.84 3.06 1.0000
-2 -15 9 310 254
250 3.84 -3.06 1.0000
0 15 -9 216 149
196 2.38 -3.58 1.0000
0 15 -9 25 211
-148 2.38 -3.58 1.0000
0 -15 9 75 238
53 2.38 3.58 1.0000
-2 15 -9 182 264
706 11.54 -0.88 1.0000
-2 15 -9 390 181
1082 11.54 -0.88 1.0000
2 -15 9 182 242
707 11.54 0.88 1.0000
-4 15 -9 722 184
831 7.23 -1.54 1.0000
-4 15 -9 803 275

922 7.23 -1.54 1.0000
4 -15 9 735 244
845 7.23 1.54 1.0000
-6 15 -9 588 272
737 4.06 -0.27 1.0000
-6 15 -9 327 185
437 4.06 -0.27 1.0000
6 -15 9 368 243
483 4.06 0.27 1.0000
-8 15 -9 123 178
133 0.27 -0.57 1.0000
-8 15 -9 652 276
676 0.27 -0.57 1.0000
8 -15 9 179 240
192 0.27 0.57 1.0000
-10 15 -9 107 273
134 0.82 0.86 1.0000
-10 15 -9 2 182
-84 0.82 0.86 1.0000
10 -15 9 51 248
70 0.82 -0.86 1.0000
-12 15 -9 2 172
-224 -1.31 -0.07 1.0000
-12 15 -9 88 272
65 -1.31 -0.07 1.0000
12 -15 9 410 267
358 -1.31 0.07 1.0000
-14 15 -9 261 201
265 -1.03 4.57 1.0000
-14 15 -9 29 261
-135 -1.03 4.57 1.0000
14 -15 9 48 284
28 -1.03 -4.57 1.0000
15 14 -9 1003 395
1031 -1.04 -4.60 1.0000
15 14 -9 30 276
-185 -1.04 -4.60 1.0000
13 14 -9 19 278
-74 -3.37 1.79 1.0000
13 14 -9 378 313
330 -3.37 1.79 1.0000
11 14 -9 109 275
226 -0.97 -3.90 1.0000
11 14 -9 47 254

131	-0.97	-3.90	1.0000	
9	14	-9	149	242
276	-8.97	-1.22	1.0000	
7	14	-9	927	244
809	-1.82	-3.25	1.0000	
-7	-14	9	970	318
852	-1.82	3.25	1.0000	
5	14	-9	705	240
763	-0.97	0.03	1.0000	
-5	-14	9	1151	277
1225	-0.97	-0.03	1.0000	
3	14	-9	1349	166
1223	1.32	1.22	1.0000	
3	14	-9	1085	247
972	1.32	1.22	1.0000	
-3	-14	9	1032	253
923	1.32	-1.22	1.0000	
1	14	-9	385	219
359	-2.74	-2.65	1.0000	
1	14	-9	281	138
256	-2.74	-2.65	1.0000	
-1	-14	9	147	197
123	-2.74	2.65	1.0000	
-1	14	-9	1707	340
1840	-2.10	0.64	1.0000	
-1	14	-9	1620	194
1749	-2.10	0.64	1.0000	
1	-14	9	1491	246
1615	-2.10	-0.64	1.0000	
-3	14	-9	69	181
401	-10.69	1.24	1.0000	
-3	14	-9	24	233
265	-10.69	1.24	1.0000	
3	-14	9	81	215
423	-10.69	-1.24	1.0000	
-5	14	-9	508	187
556	-2.05	-0.41	1.0000	
-5	14	-9	463	256
509	-2.05	-0.41	1.0000	
5	-14	9	106	210
126	-2.05	0.41	1.0000	
-7	14	-9	420	181
405	-0.44	0.82	1.0000	
-7	14	-9	589	266

572 -0.44 0.82 1.0000
7 -14 9 246 226
234 -0.44 -0.82 1.0000
-9 14 -9 8 247
-7 1.56 1.89 1.0000
-9 14 -9 25 172
25 1.56 1.89 1.0000
9 -14 9 202 227
217 1.56 -1.89 1.0000
-11 14 -9 224 194
457 5.43 -1.52 1.0000
-11 14 -9 23 268
124 5.43 -1.52 1.0000
11 -14 9 147 256
343 5.43 1.52 1.0000
-13 14 -9 61 268
168 4.69 1.87 1.0000
-13 14 -9 15 183
76 4.69 1.87 1.0000
13 -14 9 86 253
207 4.69 -1.87 1.0000
-15 14 -9 658 216
631 1.62 -3.03 1.0000
15 -14 9 429 292
406 1.62 3.03 1.0000
16 13 -9 636 408
989 1.32 5.26 1.0000
16 13 -9 163 301
361 1.32 5.26 1.0000
14 13 -9 10 267
61 4.81 -1.88 1.0000
14 13 -9 358 337
590 4.81 -1.88 1.0000
12 13 -9 203 250
95 5.43 -0.26 1.0000
12 13 -9 451 281
291 5.43 -0.26 1.0000
10 13 -9 163 255
493 8.15 1.96 1.0000
10 13 -9 3 243
109 8.15 1.96 1.0000
8 13 -9 1209 374
1940 8.04 -0.51 1.0000
6 13 -9 1282 333

1651	4.04	1.23	1.0000	
-6	-13	9	1203	290
1561	4.04	-1.23	1.0000	
4	13	-9	293	209
281	0.23	-0.83	1.0000	
4	13	-9	281	144
269	0.23	-0.83	1.0000	
-4	-13	9	414	240
400	0.23	0.83	1.0000	
2	13	-9	280	216
339	-0.33	-4.25	1.0000	
2	13	-9	582	147
675	-0.33	-4.25	1.0000	
-2	-13	9	469	210
549	-0.33	4.25	1.0000	
0	13	-9	254	147
414	5.82	3.96	1.0000	
0	13	-9	52	200
94	5.82	3.96	1.0000	
0	-13	9	69	194
128	5.82	-3.96	1.0000	
-2	13	-9	685	165
1002	1.47	-5.39	1.0000	
-2	13	-9	806	251
1148	1.47	-5.39	1.0000	
2	-13	9	501	206
773	1.47	5.39	1.0000	
-4	13	-9	1213	169
692	9.75	0.55	1.0000	
-4	13	-9	1260	252
729	9.75	0.55	1.0000	
4	-13	9	924	190
476	9.75	-0.55	1.0000	
-6	13	-9	130	165
83	1.54	-1.28	1.0000	
-6	13	-9	362	245
281	1.54	-1.28	1.0000	
6	-13	9	5	193
-22	1.54	1.28	1.0000	
-8	13	-9	4	161
-50	1.77	0.11	1.0000	
-8	13	-9	125	238
114	1.77	0.11	1.0000	
8	-13	9	4	203

-22 1.77 -0.11 1.0000
-10 13 -9 1001 284
1312 -4.04 0.23 1.0000
-10 13 -9 534 194
766 -4.04 0.23 1.0000
10 -13 9 481 248
703 -4.04 -0.23 1.0000
-12 13 -9 437 251
395 -3.53 1.54 1.0000
-12 13 -9 358 186
318 -3.53 1.54 1.0000
12 -13 9 145 241
113 -3.53 -1.54 1.0000
-14 13 -9 332 246
285 -3.47 -1.90 1.0000
-14 13 -9 376 200
327 -3.47 -1.90 1.0000
14 -13 9 332 251
289 -3.47 1.90 1.0000
-16 13 -9 59 202
220 0.71 6.13 1.0000
16 -13 9 51 274
-202 0.71 -6.13 1.0000
17 12 -9 18 308
-220 1.60 -3.32 1.0000
17 12 -9 710 389
908 1.60 -3.32 1.0000
15 12 -9 2 257
-158 -1.23 0.03 1.0000
15 12 -9 271 317
232 -1.23 0.03 1.0000
13 12 -9 86 234
59 -3.52 -1.44 1.0000
13 12 -9 366 279
329 -3.52 -1.44 1.0000
11 12 -9 134 240
114 -7.89 -1.77 1.0000
11 12 -9 183 245
179 -7.89 -1.77 1.0000
9 12 -9 432 237
289 -2.56 -2.74 1.0000
9 12 -9 315 198
194 -2.56 -2.74 1.0000
7 12 -9 4445 391

3536 -6.51 -1.00 1.0000
-7 -12 9 4502 387
3590 -6.51 1.00 1.0000
5 12 -9 819 246
950 3.26 -1.27 1.0000
5 12 -9 1197 169
1357 3.26 -1.27 1.0000
-5 -12 9 1238 253
1403 3.26 1.27 1.0000
3 12 -9 0 195
13 2.85 -1.04 1.0000
3 12 -9 12 128
-59 2.85 -1.04 1.0000
-3 -12 9 15 185
55 2.85 1.04 1.0000
1 12 -9 1028 259
1328 3.97 -0.31 1.0000
1 12 -9 1340 186
1681 3.97 -0.31 1.0000
-1 -12 9 1336 258
1675 3.97 0.31 1.0000
-1 12 -9 335 134
222 -1.94 2.47 1.0000
-1 12 -9 13 200
-85 -1.94 2.47 1.0000
1 -12 9 13 157
-80 -1.94 -2.47 1.0000
-3 12 -9 1156 209
1389 1.14 -2.66 1.0000
-3 12 -9 1340 325
1590 1.14 -2.66 1.0000
3 -12 9 1372 252
1625 1.14 2.66 1.0000
-5 12 -9 478 173
788 -5.04 -1.93 1.0000
-5 12 -9 382 252
663 -5.04 -1.93 1.0000
5 -12 9 309 187
565 -5.04 1.93 1.0000
-7 12 -9 183 166
146 0.75 1.65 1.0000
-7 12 -9 697 255
626 0.75 1.65 1.0000
7 -12 9 149 199

116 0.75 -1.65 1.0000
-9 12 -9 320 164
207 6.05 -2.09 1.0000
-9 12 -9 766 256
611 6.05 -2.09 1.0000
9 -12 9 342 211
223 6.05 2.09 1.0000
-11 12 -9 468 242
156 8.23 1.22 1.0000
-11 12 -9 92 160
-120 8.23 1.22 1.0000
11 -12 9 430 222
132 8.23 -1.22 1.0000
-13 12 -9 286 176
152 5.45 0.35 1.0000
-13 12 -9 304 227
166 5.45 0.35 1.0000
13 -12 9 40 234
-3 5.45 -0.35 1.0000
-15 12 -9 150 190
220 3.39 0.27 1.0000
15 -12 9 429 276
548 3.39 -0.27 1.0000
-17 12 -9 132 191
136 1.87 -2.52 1.0000
17 -12 9 159 282
164 1.87 2.52 1.0000
18 11 -9 581 297
660 0.13 3.03 1.0000
18 11 -9 295 327
349 0.13 3.03 1.0000
16 11 -9 23 241
57 0.12 -2.45 1.0000
16 11 -9 358 305
470 0.12 -2.45 1.0000
14 11 -9 43 217
-62 5.50 1.49 1.0000
14 11 -9 43 231
-171 5.50 1.49 1.0000
12 11 -9 251 214
39 8.37 -1.47 1.0000
12 11 -9 556 238
198 8.37 -1.47 1.0000
10 11 -9 99 194

-23	8.57	0.99	1.0000	
10	11	-9	354	212
356	8.57	0.99	1.0000	
8	11	-9	639	228
739	5.11	2.10	1.0000	
6	11	-9	9	193
-10	1.15	-2.27	1.0000	
6	11	-9	131	135
123	1.15	-2.27	1.0000	
-6	-11	9	20	207
12	1.15	2.27	1.0000	
4	11	-9	14	205
-46	-2.08	2.52	1.0000	
4	11	-9	112	134
206	-2.08	2.52	1.0000	
-4	-11	9	14	188
-21	-2.08	-2.52	1.0000	
2	11	-9	315	219
147	-5.81	2.97	1.0000	
2	11	-9	780	148
514	-5.81	2.97	1.0000	
-2	-11	9	544	170
318	-5.81	-2.97	1.0000	
0	11	-9	155	123
-114	-4.67	-9.71	1.0000	
0	11	-9	155	197
-226	-4.67	-9.71	1.0000	
0	-11	9	155	148
-8	-4.67	9.71	1.0000	
-2	11	-9	150	131
29	3.98	5.49	1.0000	
-2	11	-9	198	213
55	3.98	5.49	1.0000	
2	-11	9	469	149
238	3.98	-5.49	1.0000	
-4	11	-9	1012	243
773	-3.36	-1.95	1.0000	
-4	11	-9	1364	180
1084	-3.36	-1.95	1.0000	
4	-11	9	1364	194
1084	-3.36	1.95	1.0000	
-6	11	-9	2211	338
2222	6.41	1.28	1.0000	
-6	11	-9	2085	223

2094 6.41 1.28 1.0000
6 -11 9 1966 259
1973 6.41 -1.28 1.0000
-8 11 -9 562 252
817 -4.43 0.94 1.0000
-8 11 -9 313 177
509 -4.43 0.94 1.0000
8 -11 9 241 212
414 -4.43 -0.94 1.0000
-10 11 -9 534 200
768 -4.13 0.02 1.0000
-10 11 -9 818 248
1103 -4.13 0.02 1.0000
10 -11 9 356 229
553 -4.13 -0.02 1.0000
-12 11 -9 89 206
-21 -8.02 1.71 1.0000
-12 11 -9 89 165
-31 -8.02 1.71 1.0000
12 -11 9 191 226
190 -8.02 -1.71 1.0000
-14 11 -9 267 210
442 -1.05 3.94 1.0000
-14 11 -9 237 191
403 -1.05 3.94 1.0000
14 -11 9 326 255
517 -1.05 -3.94 1.0000
-16 11 -9 149 179
174 -4.22 -1.71 1.0000
16 -11 9 113 268
129 -4.22 1.71 1.0000
-18 11 -9 11 180
-180 1.41 2.58 1.0000
18 -11 9 11 279
-239 1.41 -2.58 1.0000
19 10 -9 689 310
638 2.17 -1.84 1.0000
19 10 -9 353 365
314 2.17 -1.84 1.0000
17 10 -9 80 291
82 0.08 -0.41 1.0000
17 10 -9 447 282
452 0.08 -0.41 1.0000
15 10 -9 212 269

253 0.99 -0.90 1.0000

15 10 -9 172 263

209 0.99 -0.90 1.0000

13 10 -9 549 261

780 -3.96 -0.31 1.0000

13 10 -9 487 237

706 -3.96 -0.31 1.0000

11 10 -9 451 216

676 -4.21 0.09 1.0000

11 10 -9 427 254

646 -4.21 0.09 1.0000

9 10 -9 556 254

963 -6.76 -2.20 1.0000

9 10 -9 753 216

1219 -6.76 -2.20 1.0000

7 10 -9 68 200

124 3.67 -1.82 1.0000

5 10 -9 2463 193

2125 2.88 -1.12 1.0000

5 10 -9 1970 302

1669 2.88 -1.12 1.0000

-5 -10 9 2022 275

1716 2.88 1.12 1.0000

3 10 -9 169 202

-81 10.83 -3.08 1.0000

3 10 -9 236 135

18 10.83 -3.08 1.0000

-3 -10 9 354 157

82 10.83 3.08 1.0000

1 10 -9 1919 222

2982 11.58 2.35 1.0000

1 10 -9 1327 350

2218 11.58 2.35 1.0000

-1 -10 9 2072 255

3161 11.58 -2.35 1.0000

-1 10 -9 993 244

672 4.28 -2.58 1.0000

-1 10 -9 1467 158

1070 4.28 -2.58 1.0000

1 -10 9 1252 162

887 4.28 2.58 1.0000

-3 10 -9 395 159

593 2.86 -2.61 1.0000

-3 10 -9 126 221

246 2.86 -2.61 1.0000

3 -10 9 240 158

399 2.86 2.61 1.0000

-5 10 -9 97 215

106 -0.59 -0.16 1.0000

-5 10 -9 324 158

341 -0.59 -0.16 1.0000

5 -10 9 250 154

266 -0.59 0.16 1.0000

-7 10 -9 46 220

-21 0.80 -5.81 1.0000

-7 10 -9 40 153

144 0.80 -5.81 1.0000

7 -10 9 46 164

-9 0.80 5.81 1.0000

-9 10 -9 736 232

551 5.86 1.77 1.0000

-9 10 -9 627 172

455 5.86 1.77 1.0000

9 -10 9 368 202

233 5.86 -1.77 1.0000

-11 10 -9 291 171

288 8.54 -0.87 1.0000

-11 10 -9 138 208

95 8.54 -0.87 1.0000

11 -10 9 95 208

24 8.54 0.87 1.0000

-13 10 -9 24 164

200 8.09 -2.05 1.0000

-13 10 -9 116 219

407 8.09 -2.05 1.0000

13 -10 9 55 226

281 8.09 2.05 1.0000

-15 10 -9 39 165

-19 5.37 -0.44 1.0000

-15 10 -9 58 185

169 5.37 -0.44 1.0000

15 -10 9 15 243

71 5.37 0.44 1.0000

-17 10 -9 549 196

542 0.22 1.45 1.0000

17 -10 9 181 266

177 0.22 -1.45 1.0000

-19 10 -9 111 204

125 -0.69 -1.30 1.0000
19 -10 9 222 305
242 -0.69 1.30 1.0000
18 9 -9 574 308
569 -1.41 0.02 1.0000
18 9 -9 90 316
86 -1.41 0.02 1.0000
16 9 -9 187 294
255 1.81 -0.95 1.0000
16 9 -9 311 285
398 1.81 -0.95 1.0000
14 9 -9 120 259
131 1.60 -1.96 1.0000
14 9 -9 9 248
-134 1.60 -1.96 1.0000
12 9 -9 391 196
265 6.19 2.14 1.0000
12 9 -9 547 260
404 6.19 2.14 1.0000
10 9 -9 482 180
332 5.93 -1.89 1.0000
10 9 -9 153 242
60 5.93 -1.89 1.0000
8 9 -9 5 225
-25 1.37 1.25 1.0000
8 9 -9 5 159
-84 1.37 1.25 1.0000
6 9 -9 1131 267
1442 -3.23 5.85 1.0000
4 9 -9 1364 337
2068 -10.10 -2.80 1.0000
4 9 -9 1620 203
2387 -10.10 -2.80 1.0000
-4 -9 9 1795 260
2594 -10.10 2.80 1.0000
2 9 -9 436 192
39 -12.48 -2.81 1.0000
2 9 -9 480 137
53 -12.48 -2.81 1.0000
0 9 -9 297 229
693 -9.80 7.56 1.0000
0 9 -9 528 155
1070 -9.80 7.56 1.0000
0 -9 9 387 212

850	-9.80	-7.56	1.0000	
0	-9	9	588	163
1165	-9.80	-7.56	1.0000	
-2	9	-9	155	222
531	-8.53	-5.58	1.0000	
-2	9	-9	257	161
715	-8.53	-5.58	1.0000	
2	-9	9	299	154
788	-8.53	5.58	1.0000	
-4	9	-9	443	149
526	2.64	3.99	1.0000	
-4	9	-9	267	213
325	2.64	3.99	1.0000	
4	-9	9	359	147
426	2.64	-3.99	1.0000	
-6	9	-9	2314	333
1989	-1.71	2.88	1.0000	
-6	9	-9	2414	216
2082	-1.71	2.88	1.0000	
6	-9	9	2149	235
1836	-1.71	-2.88	1.0000	
-8	9	-9	1009	258
975	1.37	0.13	1.0000	
-8	9	-9	583	163
557	1.37	0.13	1.0000	
8	-9	9	502	190
478	1.37	-0.13	1.0000	
-10	9	-9	980	303
1513	-6.85	2.22	1.0000	
-10	9	-9	717	207
1180	-6.85	2.22	1.0000	
10	-9	9	717	238
1181	-6.85	-2.22	1.0000	
-12	9	-9	627	180
456	-2.57	2.64	1.0000	
-12	9	-9	638	208
465	-2.57	2.64	1.0000	
12	-9	9	67	214
18	-2.57	-2.64	1.0000	
-14	9	-9	211	176
378	-9.07	1.39	1.0000	
-14	9	-9	275	183
477	-9.07	1.39	1.0000	
14	-9	9	260	244

453 -9.07 -1.39 1.0000
-16 9 -9 904 205
978 -0.65 4.45 1.0000
16 -9 9 460 271
508 -0.65 -4.45 1.0000
-18 9 -9 142 189
165 0.62 -1.03 1.0000
18 -9 9 167 278
192 0.62 1.03 1.0000
19 8 -9 40 325
65 0.18 -1.50 1.0000
19 8 -9 3 321
-66 0.18 -1.50 1.0000
17 8 -9 346 309
505 3.76 -0.86 1.0000
17 8 -9 79 300
159 3.76 -0.86 1.0000
15 8 -9 520 275
542 0.30 0.49 1.0000
15 8 -9 546 296
569 0.30 0.49 1.0000
13 8 -9 5 203
-19 1.84 -0.12 1.0000
13 8 -9 9 254
3 1.84 -0.12 1.0000
11 8 -9 197 247
350 -4.24 -1.02 1.0000
11 8 -9 136 167
266 -4.24 -1.02 1.0000
9 8 -9 531 176
505 1.35 -0.15 1.0000
9 8 -9 325 239
304 1.35 -0.15 1.0000
7 8 -9 74 239
170 2.36 -3.12 1.0000
5 8 -9 3683 384
2760 10.18 -4.21 1.0000
3 8 -9 1376 141
384 14.86 4.13 1.0000
3 8 -9 973 205
186 14.86 4.13 1.0000
1 8 -9 531 308
1502 14.37 -4.00 1.0000
1 8 -9 1055 191

2342	14.37	-4.00	1.0000	
-1	8	-9	2738	292
1577	15.57	-3.01	1.0000	
-1	8	-9	3816	194
2451	15.57	-3.01	1.0000	
-3	8	-9	555	160
595	0.79	0.15	1.0000	
-3	8	-9	295	221
325	0.79	0.15	1.0000	
3	-8	9	594	144
636	0.79	-0.15	1.0000	
-5	8	-9	927	185
1347	-0.60	-7.29	1.0000	
-5	8	-9	1159	262
1627	-0.60	-7.29	1.0000	
5	-8	9	715	194
1088	-0.60	7.29	1.0000	
-7	8	-9	2212	216
2163	-1.10	-0.77	1.0000	
-7	8	-9	2401	336
2350	-1.10	-0.77	1.0000	
7	-8	9	1883	238
1838	-1.10	0.77	1.0000	
-9	8	-9	5	213
-185	1.41	-1.34	1.0000	
-9	8	-9	5	137
-16	1.41	-1.34	1.0000	
9	-8	9	5	159
-131	1.41	1.34	1.0000	
-11	8	-9	989	204
1116	5.00	-2.05	1.0000	
-11	8	-9	1425	279
1583	5.00	-2.05	1.0000	
11	-8	9	875	235
995	5.00	2.05	1.0000	
-13	8	-9	1765	286
2630	8.03	0.46	1.0000	
-13	8	-9	1159	252
1876	8.03	0.46	1.0000	
13	-8	9	1439	329
2227	8.03	-0.46	1.0000	
-15	8	-9	128	173
309	5.36	-1.83	1.0000	
15	-8	9	43	234

-54 5.36 1.83 1.0000
-17 8 -9 353 189
329 -0.29 -1.41 1.0000
17 -8 9 277 261
255 -0.29 1.41 1.0000
-19 8 -9 3 189
4 -2.06 2.29 1.0000
19 -8 9 13 283
-92 -2.06 -2.29 1.0000
20 7 -9 2 348
8 -1.74 -0.35 1.0000
20 7 -9 4 381
-90 -1.74 -0.35 1.0000
18 7 -9 69 302
99 -1.03 -2.35 1.0000
18 7 -9 551 336
642 -1.03 -2.35 1.0000
16 7 -9 4 308
-3 -1.26 1.32 1.0000
16 7 -9 359 261
331 -1.26 1.32 1.0000
14 7 -9 68 286
62 -0.40 -0.88 1.0000
14 7 -9 274 215
263 -0.40 -0.88 1.0000
12 7 -9 364 197
314 0.70 -1.74 1.0000
12 7 -9 89 271
64 0.70 -1.74 1.0000
10 7 -9 17 259
93 0.84 5.85 1.0000
10 7 -9 37 157
142 0.84 5.85 1.0000
8 7 -9 1780 324
1742 -1.09 0.85 1.0000
6 7 -9 658 254
762 -8.26 1.13 1.0000
4 7 -9 1029 344
2025 -11.95 4.98 1.0000
2 7 -9 30 225
552 -16.51 -0.35 1.0000
2 7 -9 114 142
847 -16.51 -0.35 1.0000
0 7 -9 2293 257

4502	-16.29	-4.49	1.0000	
0	7	-9	1794	369
3789	-16.29	-4.49	1.0000	
-2	7	-9	1450	247
1550	-6.53	7.67	1.0000	
-2	7	-9	1865	193
1996	-6.53	7.67	1.0000	
-4	7	-9	158	212
130	-0.82	0.58	1.0000	
-4	7	-9	416	147
370	-0.82	0.58	1.0000	
4	-7	9	515	132
464	-0.82	-0.58	1.0000	
-6	7	-9	2244	225
2633	3.82	1.72	1.0000	
-6	7	-9	2586	338
3003	3.82	1.72	1.0000	
6	-7	9	2627	246
3045	3.82	-1.72	1.0000	
-8	7	-9	238	212
392	2.31	3.13	1.0000	
-8	7	-9	70	141
162	2.31	3.13	1.0000	
8	-7	9	106	161
215	2.31	-3.13	1.0000	
-10	7	-9	54	146
101	3.60	1.80	1.0000	
-10	7	-9	324	200
447	3.60	1.80	1.0000	
10	-7	9	192	163
287	3.60	-1.80	1.0000	
-12	7	-9	4984	321
4028	-6.50	1.12	1.0000	
-12	7	-9	4224	281
3347	-6.50	1.12	1.0000	
12	-7	9	3948	338
3099	-6.50	-1.12	1.0000	
-14	7	-9	872	202
759	-1.83	3.29	1.0000	
-14	7	-9	1061	175
937	-1.83	3.29	1.0000	
14	-7	9	876	253
760	-1.83	-3.29	1.0000	
-16	7	-9	95	166

116 -1.13 0.39 1.0000
16 -7 9 302 256
339 -1.13 -0.39 1.0000
-18 7 -9 350 184
391 0.45 1.65 1.0000
18 -7 9 62 256
77 0.45 -1.65 1.0000
-20 7 -9 262 199
321 1.66 0.36 1.0000
20 -7 9 230 295
285 1.66 -0.36 1.0000
19 6 -9 20 341
-102 3.89 -0.46 1.0000
19 6 -9 352 357
210 3.89 -0.46 1.0000
17 6 -9 510 328
447 3.15 -0.27 1.0000
17 6 -9 151 296
113 3.15 -0.27 1.0000
15 6 -9 201 291
283 4.10 0.21 1.0000
15 6 -9 321 252
427 4.10 0.21 1.0000
13 6 -9 6 198
-181 1.73 1.30 1.0000
13 6 -9 79 262
42 1.73 1.30 1.0000
11 6 -9 1481 258
1493 6.35 -1.43 1.0000
11 6 -9 1481 305
1493 6.35 -1.43 1.0000
9 6 -9 2567 404
2232 -1.57 -2.82 1.0000
7 6 -9 2417 400
2824 3.90 -1.90 1.0000
5 6 -9 1258 363
1805 10.52 0.21 1.0000
3 6 -9 232 255
892 16.20 -9.09 1.0000
1 6 -9 567 153
1084 17.68 4.24 1.0000
1 6 -9 575 245
1099 17.68 4.24 1.0000
-1 6 -9 3436 342

2418	9.03	-0.27	1.0000	
-1	6	-9	4091	215
2974	9.03	-0.27	1.0000	
-3	6	-9	514	213
670	3.87	-8.70	1.0000	
-3	6	-9	826	163
1052	3.87	-8.70	1.0000	
-5	6	-9	499	141
291	-4.95	0.74	1.0000	
-5	6	-9	443	199
248	-4.95	0.74	1.0000	
-5	6	-9	326	171
162	-4.95	0.74	1.0000	
5	-6	9	326	126
162	-4.95	-0.74	1.0000	
-7	6	-9	692	155
808	-8.27	-1.10	1.0000	
-7	6	-9	568	219
665	-8.27	-1.10	1.0000	
7	-6	9	604	170
701	-8.27	1.10	1.0000	
-9	6	-9	1571	214
1956	-3.31	-5.80	1.0000	
-9	6	-9	1600	299
1989	-3.31	-5.80	1.0000	
9	-6	9	1051	238
1356	-3.31	5.80	1.0000	
-11	6	-9	165	195
160	1.19	2.25	1.0000	
-11	6	-9	156	149
151	1.19	2.25	1.0000	
11	-6	9	9	167
-93	1.19	-2.25	1.0000	
-13	6	-9	1219	226
1578	4.02	-1.27	1.0000	
13	-6	9	1058	260
1394	4.02	1.27	1.0000	
-15	6	-9	107	165
107	0.87	0.20	1.0000	
15	-6	9	1	223
-17	0.87	-0.20	1.0000	
-17	6	-9	3	159
-58	-0.01	1.56	1.0000	
17	-6	9	3	242

-48	-0.01	-1.56	1.0000	
-19	6	-9	25	177
-15	-2.48	-3.56	1.0000	
19	-6	9	25	258
-104	-2.48	3.56	1.0000	
20	5	-9	10	365
-157	-2.68	0.07	1.0000	
20	5	-9	10	366
-67	-2.68	0.07	1.0000	
18	5	-9	221	350
397	-4.46	-1.02	1.0000	
18	5	-9	447	343
688	-4.46	-1.02	1.0000	
16	5	-9	58	294
111	-4.21	-1.45	1.0000	
16	5	-9	26	296
-50	-4.21	-1.45	1.0000	
14	5	-9	218	280
247	-2.06	0.34	1.0000	
14	5	-9	292	236
326	-2.06	0.34	1.0000	
12	5	-9	336	271
597	-4.94	1.96	1.0000	
12	5	-9	575	207
905	-4.94	1.96	1.0000	
10	5	-9	560	260
582	-0.53	0.11	1.0000	
8	5	-9	888	286
1307	-0.62	7.35	1.0000	
6	5	-9	845	254
557	-5.05	-0.49	1.0000	
4	5	-9	1733	240
765	-12.94	2.82	1.0000	
2	5	-9	1420	222
393	-15.04	3.64	1.0000	
0	5	-9	1177	181
1987	-9.20	0.27	1.0000	
0	5	-9	1185	324
1997	-9.20	0.27	1.0000	
-2	5	-9	1727	324
2307	-5.74	0.00	1.0000	
-2	5	-9	1639	204
2205	-5.74	0.00	1.0000	
-4	5	-9	1287	292

1931	5.58		4.51	1.0000
-4	5	-9	1802	209
2553	5.58		4.51	1.0000
-6	5	-9	1231	200
1768	10.47		-0.28	1.0000
-6	5	-9	1404	257
1981	10.47		-0.28	1.0000
6	-5	9	1189	200
1710	10.47		0.28	1.0000
-8	5	-9	4105	237
3127	10.10		4.23	1.0000
-8	5	-9	3749	321
2813	10.10		4.23	1.0000
8	-5	9	3729	262
2811	10.10		-4.23	1.0000
-10	5	-9	2423	278
2079	2.96		1.12	1.0000
-10	5	-9	1968	211
1659	2.96		1.12	1.0000
10	-5	9	1723	246
1435	2.96		-1.12	1.0000
-12	5	-9	696	184
809	3.15		1.36	1.0000
-12	5	-9	1153	241
1300	3.15		1.36	1.0000
12	-5	9	786	235
905	3.15		-1.36	1.0000
-14	5	-9	961	205
1030	-0.98		-0.03	1.0000
14	-5	9	549	234
601	-0.98		0.03	1.0000
-16	5	-9	2	147
-122	0.84		0.81	1.0000
16	-5	9	218	232
195	0.84		-0.81	1.0000
-18	5	-9	66	166
93	2.14		0.91	1.0000
18	-5	9	7	249
-164	2.14		-0.91	1.0000
-20	5	-9	86	187
107	0.04		1.18	1.0000
20	-5	9	2	285
-284	0.04		-1.18	1.0000
21	4	-9	1	377

-481	0.02	-0.78	1.0000	
21	4	-9	97	393
105	0.02	-0.78	1.0000	
19	4	-9	35	349
-226	5.07	-0.67	1.0000	
19	4	-9	35	319
-435	5.07	-0.67	1.0000	
17	4	-9	231	318
442	5.09	0.41	1.0000	
17	4	-9	497	309
790	5.09	0.41	1.0000	
15	4	-9	770	261
888	7.39	1.59	1.0000	
15	4	-9	771	311
889	7.39	1.59	1.0000	
13	4	-9	746	276
351	9.71	-0.70	1.0000	
13	4	-9	1202	221
697	9.71	-0.70	1.0000	
11	4	-9	1333	300
1060	-3.23	1.96	1.0000	
9	4	-9	601	263
694	2.63	-4.13	1.0000	
7	4	-9	516	245
465	-0.82	-0.57	1.0000	
5	4	-9	1913	354
2704	5.73	-4.59	1.0000	
3	4	-9	4534	441
6182	11.22	3.48	1.0000	
1	4	-9	1800	378
3422	12.53	-6.80	1.0000	
-1	4	-9	1955	325
1635	7.99	-2.95	1.0000	
-1	4	-9	1909	179
1592	7.99	-2.95	1.0000	
-3	4	-9	1442	228
1037	-4.06	2.94	1.0000	
-3	4	-9	1701	171
1258	-4.06	2.94	1.0000	
-5	4	-9	2080	168
1007	-12.96	-2.82	1.0000	
-5	4	-9	1242	196
448	-12.96	-2.82	1.0000	
-7	4	-9	695	293

1546 -11.96 -4.94 1.0000
-7 4 -9 1021 204
2027 -11.96 -4.94 1.0000
7 -4 9 1097 217
2123 -11.96 4.94 1.0000
-9 4 -9 1477 288
2201 -10.10 2.81 1.0000
-9 4 -9 1535 216
2273 -10.10 2.81 1.0000
9 -4 9 1450 249
2175 -10.10 -2.81 1.0000
-11 4 -9 222 190
351 -2.15 -2.55 1.0000
-11 4 -9 38 145
100 -2.15 -2.55 1.0000
11 -4 9 102 159
194 -2.15 2.55 1.0000
-13 4 -9 289 155
276 0.25 0.82 1.0000
13 -4 9 300 197
287 0.25 -0.82 1.0000
-15 4 -9 270 161
186 0.86 2.91 1.0000
15 -4 9 149 224
88 0.86 -2.91 1.0000
-17 4 -9 38 153
82 -1.22 -3.00 1.0000
17 -4 9 15 247
44 -1.22 3.00 1.0000
-19 4 -9 160 177
268 -2.76 2.07 1.0000
19 -4 9 181 293
296 -2.76 -2.07 1.0000
-21 4 -9 3 196
7 -0.86 -1.87 1.0000
21 -4 9 46 310
72 -0.86 1.87 1.0000
20 3 -9 11 348
-377 -0.67 -2.79 1.0000
20 3 -9 11 342
-635 -0.67 -2.79 1.0000
18 3 -9 754 342
907 -4.82 3.47 1.0000
18 3 -9 123 332

160	-4.82	3.47	1.0000	
16	3	-9	552	287
335	-6.92	-2.31	1.0000	
16	3	-9	1237	309
924	-6.92	-2.31	1.0000	
14	3	-9	9	260
134	-10.61	-1.33	1.0000	
14	3	-9	22	232
245	-10.61	-1.33	1.0000	
12	3	-9	1547	374
1817	1.19	2.67	1.0000	
12	3	-9	1388	279
1645	1.19	2.67	1.0000	
10	3	-9	466	254
677	2.79	2.67	1.0000	
8	3	-9	514	232
554	0.88	-0.05	1.0000	
6	3	-9	772	235
976	3.92	8.74	1.0000	
4	3	-9	2137	315
1639	-4.12	-2.83	1.0000	
2	3	-9	4533	374
3630	-6.10	-1.52	1.0000	
0	3	-9	93	209
503	-7.34	8.31	1.0000	
-2	3	-9	527	208
559	4.76	-0.68	1.0000	
-2	3	-9	421	143
446	4.76	-0.68	1.0000	
-4	3	-9	3503	311
4952	11.25	-3.43	1.0000	
-4	3	-9	4928	409
6634	11.25	-3.43	1.0000	
-6	3	-9	183	209
747	16.18	9.21	1.0000	
-6	3	-9	196	154
787	16.18	9.21	1.0000	
-6	3	-9	225	160
870	16.18	9.21	1.0000	
6	-3	9	285	174
1032	16.18	-9.21	1.0000	
-8	3	-9	1362	150
374	14.88	-4.13	1.0000	
-8	3	-9	1069	183

228 14.88 -4.13 1.0000
8 -3 9 1144 153
265 14.88 4.13 1.0000
-10 3 -9 201 126
6 10.72 3.07 1.0000
-10 3 -9 496 183
172 10.72 3.07 1.0000
10 -3 9 165 153
-188 10.72 -3.07 1.0000
-12 3 -9 170 150
273 2.86 1.04 1.0000
12 -3 9 203 194
315 2.86 -1.04 1.0000
-14 3 -9 1085 193
974 1.33 -1.10 1.0000
14 -3 9 1144 248
1029 1.33 1.10 1.0000
-16 3 -9 6 166
-104 -1.38 1.61 1.0000
16 -3 9 21 240
41 -1.38 -1.61 1.0000
-18 3 -9 51 161
62 0.57 -0.28 1.0000
18 -3 9 253 275
276 0.57 0.28 1.0000
-20 3 -9 64 178
50 1.87 0.47 1.0000
20 -3 9 403 317
374 1.87 -0.47 1.0000
21 2 -9 7 394
-12 1.95 1.17 1.0000
19 2 -9 110 352
110 -0.28 -0.10 1.0000
19 2 -9 0 323
-190 -0.28 -0.10 1.0000
17 2 -9 1433 345
2103 7.08 -0.29 1.0000
17 2 -9 771 342
1278 7.08 -0.29 1.0000
15 2 -9 127 271
597 11.62 0.82 1.0000
15 2 -9 287 263
909 11.62 0.82 1.0000
13 2 -9 295 260

506	1.51	5.36	1.0000	
13	2	-9	442	232
696	1.51	5.36	1.0000	
11	2	-9	507	231
268	4.00	-5.44	1.0000	
9	2	-9	243	224
689	-8.45	5.55	1.0000	
7	2	-9	1681	320
1816	-6.50	-7.75	1.0000	
5	2	-9	1804	318
2418	-5.89	0.16	1.0000	
3	2	-9	306	201
330	4.89	0.58	1.0000	
1	2	-9	648	225
1048	5.74	-1.79	1.0000	
-1	2	-9	954	214
797	1.34	-3.21	1.0000	
-3	2	-9	4279	233
3418	-6.00	1.71	1.0000	
-3	2	-9	4317	341
3452	-6.00	1.71	1.0000	
-5	2	-9	1789	203
594	-15.12	-3.62	1.0000	
-5	2	-9	1246	136
302	-15.12	-3.62	1.0000	
-7	2	-9	80	157
746	-16.54	0.27	1.0000	
-7	2	-9	148	204
941	-16.54	0.27	1.0000	
7	-2	9	121	162
867	-16.54	-0.27	1.0000	
-9	2	-9	740	136
156	-12.57	2.81	1.0000	
-9	2	-9	221	175
-23	-12.57	2.81	1.0000	
9	-2	9	1132	164
362	-12.57	-2.81	1.0000	
-11	2	-9	724	155
464	-5.78	-2.96	1.0000	
11	-2	9	626	190
389	-5.78	2.96	1.0000	
-13	2	-9	488	168
577	-0.45	4.26	1.0000	
13	-2	9	367	226

443 -0.45 -4.26 1.0000
-15 2 -9 312 173
250 3.82 -2.96 1.0000
15 -2 9 250 225
188 3.82 2.96 1.0000
-17 2 -9 224 186
335 0.84 3.98 1.0000
17 -2 9 212 254
320 0.84 -3.98 1.0000
-19 2 -9 202 190
259 -0.26 -2.03 1.0000
19 -2 9 170 287
222 -0.26 2.03 1.0000
-21 2 -9 329 222
472 -3.09 0.63 1.0000
21 -2 9 235 307
359 -3.09 -0.63 1.0000
20 1 -9 16 330
-196 -3.23 1.34 1.0000
18 1 -9 24 293
-130 -2.36 -3.51 1.0000
18 1 -9 326 290
385 -2.36 -3.51 1.0000
16 1 -9 86 255
24 -7.95 2.77 1.0000
16 1 -9 110 245
72 -7.95 2.77 1.0000
14 1 -9 1097 276
1196 -2.09 -0.55 1.0000
12 1 -9 289 228
183 -1.91 -2.61 1.0000
12 1 -9 291 188
185 -1.91 -2.61 1.0000
10 1 -9 1259 241
892 4.32 2.55 1.0000
8 1 -9 3462 319
2148 15.51 3.19 1.0000
6 1 -9 4380 328
3226 9.07 0.22 1.0000
4 1 -9 2124 285
1797 8.01 3.06 1.0000
2 1 -9 914 209
752 1.43 3.27 1.0000
0 1 -9 5700 388

5722	1.91	-3.23	1.0000	
-2	1	-9	1155	276
1673	5.71	1.87	1.0000	
-4	1	-9	1689	318
3262	12.47	6.84	1.0000	
-4	1	-9	1416	222
2876	12.47	6.84	1.0000	
-6	1	-9	756	227
1406	17.60	-4.23	1.0000	
-6	1	-9	744	184
1386	17.60	-4.23	1.0000	
6	-1	9	603	189
1144	17.60	4.23	1.0000	
-8	1	-9	811	283
1962	14.32	4.06	1.0000	
-8	1	-9	710	205
1800	14.32	4.06	1.0000	
8	-1	9	945	227
2169	14.32	-4.06	1.0000	
-10	1	-9	1896	238
2938	11.53	-2.26	1.0000	
10	-1	9	2149	285
3269	11.53	2.26	1.0000	
-12	1	-9	1335	214
1680	4.02	0.36	1.0000	
12	-1	9	1490	278
1855	4.02	-0.36	1.0000	
-14	1	-9	320	165
300	-2.80	2.49	1.0000	
14	-1	9	421	222
402	-2.80	-2.49	1.0000	
-16	1	-9	12	163
-89	-1.31	-2.71	1.0000	
16	-1	9	223	227
172	-1.31	2.71	1.0000	
-18	1	-9	23	180
-42	-3.84	1.65	1.0000	
18	-1	9	23	267
-3	-3.84	-1.65	1.0000	
-20	1	-9	100	185
123	-0.26	-1.01	1.0000	
20	-1	9	156	295
185	-0.26	1.01	1.0000	
21	0	-9	0	348

-507	0.12	-0.60	1.0000	
19	0	-9	12	275
-341	2.82	1.12	1.0000	
19	0	-9	12	312
-474	2.82	1.12	1.0000	
17	0	-9	128	273
258	4.47	-0.22	1.0000	
17	0	-9	236	294
406	4.47	-0.22	1.0000	
15	0	-9	665	255
646	2.41	3.53	1.0000	
13	0	-9	94	229
173	5.80	-3.93	1.0000	
13	0	-9	58	197
107	5.80	-3.93	1.0000	
11	0	-9	559	194
184	-4.67	9.71	1.0000	
9	0	-9	736	283
1384	-9.77	-7.59	1.0000	
7	0	-9	2280	359
4487	-16.35	4.47	1.0000	
5	0	-9	1116	272
1908	-9.16	-0.12	1.0000	
3	0	-9	82	193
485	-7.51	-8.36	1.0000	
1	0	-9	6412	406
6426	1.96	3.29	1.0000	
-1	0	-9	5818	376
5817	2.08	3.33	1.0000	
-3	0	-9	112	179
552	-7.42	-8.36	1.0000	
-3	0	-9	1	120
194	-7.42	-8.36	1.0000	
-5	0	-9	1677	296
2628	-9.21	-0.25	1.0000	
-5	0	-9	1139	200
1940	-9.21	-0.25	1.0000	
-7	0	-9	1997	333
4074	-16.26	4.53	1.0000	
-7	0	-9	1686	260
3623	-16.26	4.53	1.0000	
-7	0	-9	1751	260
3718	-16.26	4.53	1.0000	
7	0	9	2036	274

4134 -16.26 -4.53 1.0000
-9 0 -9 414 171
899 -9.79 -7.63 1.0000
-9 0 -9 437 199
936 -9.79 -7.63 1.0000
9 0 9 396 193
866 -9.79 7.63 1.0000
-11 0 -9 298 132
39 -4.80 9.62 1.0000
11 0 9 550 188
158 -4.80 -9.62 1.0000
-13 0 -9 90 145
169 5.88 -3.89 1.0000
13 0 9 115 220
212 5.88 3.89 1.0000
-15 0 -9 25 153
-84 2.32 3.64 1.0000
15 0 9 78 227
57 2.32 -3.64 1.0000
-17 0 -9 248 175
421 4.42 -0.14 1.0000
17 0 9 15 255
72 4.42 0.14 1.0000
-19 0 -9 41 177
71 2.65 1.06 1.0000
19 0 9 9 286
18 2.65 -1.06 1.0000
-21 0 -9 1 190
-48 0.28 -0.59 1.0000
21 0 9 150 312
141 0.28 0.59 1.0000
20 -1 -9 1 331
-120 -0.30 -0.99 1.0000
18 -1 -9 23 283
-7 -3.81 1.60 1.0000
18 -1 -9 23 258
-38 -3.81 1.60 1.0000
16 -1 -9 610 251
537 -1.42 -2.63 1.0000
14 -1 -9 291 225
265 -2.73 2.67 1.0000
14 -1 -9 563 213
534 -2.73 2.67 1.0000
12 -1 -9 978 253

1274 4.03 0.28 1.0000
10 -1 -9 2146 348
3263 11.65 -2.34 1.0000
8 -1 -9 1024 298
2296 14.35 4.01 1.0000
6 -1 -9 684 229
1284 17.62 -4.18 1.0000
4 -1 -9 1500 297
3023 12.70 6.90 1.0000
2 -1 -9 840 227
1281 5.62 1.86 1.0000
0 -1 -9 6504 397
6506 2.10 -3.30 1.0000
-2 -1 -9 1081 185
910 1.34 3.32 1.0000
-4 -1 -9 1701 179
1405 7.92 3.05 1.0000
-4 -1 -9 2118 261
1796 7.92 3.05 1.0000
-6 -1 -9 3975 229
2880 9.03 0.28 1.0000
-6 -1 -9 4503 298
3333 9.03 0.28 1.0000
6 1 9 3946 235
2850 9.03 -0.28 1.0000
-8 -1 -9 2982 208
1772 15.46 3.09 1.0000
-8 -1 -9 3669 290
2330 15.46 3.09 1.0000
8 1 9 3627 239
2292 15.46 -3.09 1.0000
-10 -1 -9 1028 170
700 4.30 2.54 1.0000
10 1 9 1144 194
797 4.30 -2.54 1.0000
-12 -1 -9 93 131
36 -2.10 -2.49 1.0000
12 1 9 384 191
257 -2.10 2.49 1.0000
-14 -1 -9 1237 221
1344 -2.04 -0.58 1.0000
14 1 9 1196 270
1301 -2.04 0.58 1.0000
-16 -1 -9 87 150

4	-8.10	2.89	1.0000	
16	1	9	98	238
-232	-8.10	-2.89	1.0000	
-18	-1	-9	90	192
114	-2.20	-3.56	1.0000	
18	1	9	20	266
14	-2.20	3.56	1.0000	
-20	-1	-9	184	194
235	-3.20	1.30	1.0000	
20	1	9	16	295
-60	-3.20	-1.30	1.0000	
21	-2	-9	184	367
293	-3.06	0.66	1.0000	
19	-2	-9	6	257
-177	-0.36	-2.01	1.0000	
19	-2	-9	6	311
-274	-0.36	-2.01	1.0000	
17	-2	-9	141	242
233	0.95	4.06	1.0000	
17	-2	-9	74	271
141	0.95	4.06	1.0000	
15	-2	-9	76	208
35	3.79	-3.19	1.0000	
15	-2	-9	363	235
306	3.79	-3.19	1.0000	
13	-2	-9	772	209
875	-0.28	4.20	1.0000	
13	-2	-9	702	237
800	-0.28	4.20	1.0000	
11	-2	-9	728	207
469	-5.77	-3.00	1.0000	
9	-2	-9	615	181
100	-12.60	2.85	1.0000	
7	-2	-9	86	193
755	-16.38	0.49	1.0000	
5	-2	-9	1524	174
438	-15.21	-3.88	1.0000	
3	-2	-9	4016	301
3164	-6.15	1.63	1.0000	
1	-2	-9	1162	202
980	1.45	-3.29	1.0000	
-1	-2	-9	851	198
1292	5.58	-1.87	1.0000	
-3	-2	-9	566	170

603	4.79	0.67	1.0000	
-5	-2	-9	1437	204
1969	-5.70	0.07	1.0000	
-5	-2	-9	1936	269
2547	-5.70	0.07	1.0000	
-7	-2	-9	1283	189
1401	-6.70	-7.70	1.0000	
-7	-2	-9	1826	266
1993	-6.70	-7.70	1.0000	
7	2	9	1473	218
1587	-6.70	7.70	1.0000	
-9	-2	-9	203	187
623	-8.58	5.55	1.0000	
-9	-2	-9	240	169
689	-8.58	5.55	1.0000	
9	2	9	161	176
542	-8.58	-5.55	1.0000	
-11	-2	-9	584	141
332	4.07	-5.33	1.0000	
11	2	9	302	184
124	4.07	5.33	1.0000	
-13	-2	-9	366	172
606	1.42	5.46	1.0000	
13	2	9	507	246
787	1.42	-5.46	1.0000	
-15	-2	-9	308	201
936	11.47	0.79	1.0000	
15	2	9	437	285
1152	11.47	-0.79	1.0000	
-17	-2	-9	642	211
1110	7.09	-0.36	1.0000	
17	2	9	736	311
1234	7.09	0.36	1.0000	
-19	-2	-9	0	190
-20	-0.26	-0.08	1.0000	
19	2	9	0	288
-9	-0.26	0.08	1.0000	
-21	-2	-9	7	196
-82	1.97	1.10	1.0000	
21	2	9	162	315
118	1.97	-1.10	1.0000	
20	-3	-9	385	349
356	1.90	0.47	1.0000	
20	-3	-9	5	279

-142 1.90 0.47 1.0000

18 -3 -9 0 242

-82 0.53 -0.24 1.0000

18 -3 -9 71 269

82 0.53 -0.24 1.0000

16 -3 -9 15 229

30 -1.33 1.68 1.0000

16 -3 -9 30 246

51 -1.33 1.68 1.0000

14 -3 -9 2067 300

1905 1.38 -1.22 1.0000

14 -3 -9 2304 339

2133 1.38 -1.22 1.0000

12 -3 -9 172 172

276 2.81 1.19 1.0000

12 -3 -9 320 204

457 2.81 1.19 1.0000

10 -3 -9 433 178

132 10.74 3.10 1.0000

8 -3 -9 1282 179

327 14.93 -4.32 1.0000

6 -3 -9 223 202

866 16.21 9.21 1.0000

4 -3 -9 4172 376

5737 11.19 -3.44 1.0000

2 -3 -9 232 164

250 4.89 -0.53 1.0000

0 -3 -9 20 161

307 -7.55 8.49 1.0000

-2 -3 -9 3928 275

3095 -6.06 -1.71 1.0000

-4 -3 -9 1553 168

1136 -4.06 -2.85 1.0000

-4 -3 -9 1548 230

1131 -4.06 -2.85 1.0000

-6 -3 -9 572 150

737 3.83 8.76 1.0000

-6 -3 -9 720 178

918 3.83 8.76 1.0000

-6 -3 -9 724 169

923 3.83 8.76 1.0000

6 3 9 696 170

900 3.83 -8.76 1.0000

-8 -3 -9 346 148

380 0.82 -0.20 1.0000
-8 -3 -9 506 176
547 0.82 -0.20 1.0000
8 3 9 557 173
600 0.82 0.20 1.0000
-10 -3 -9 108 144
220 2.84 2.60 1.0000
10 3 9 100 178
209 2.84 -2.60 1.0000
-12 -3 -9 1555 211
1818 1.15 2.60 1.0000
12 3 9 1263 269
1501 1.15 -2.60 1.0000
-14 -3 -9 46 169
329 -10.66 -1.31 1.0000
14 3 9 47 243
340 -10.66 1.31 1.0000
-16 -3 -9 911 193
641 -6.75 -2.21 1.0000
16 3 9 629 269
403 -6.75 2.21 1.0000
-18 -3 -9 257 197
337 -4.95 3.62 1.0000
18 3 9 247 282
325 -4.95 -3.62 1.0000
-20 -3 -9 11 193
-53 -0.61 -2.78 1.0000
20 3 9 11 296
-25 -0.61 2.78 1.0000
21 -4 -9 5 305
-222 -0.86 -1.82 1.0000
21 -4 -9 179 369
229 -0.86 -1.82 1.0000
19 -4 -9 17 305
-280 -2.87 2.13 1.0000
19 -4 -9 17 269
-54 -2.87 2.13 1.0000
17 -4 -9 15 236
-5 -1.15 -3.10 1.0000
17 -4 -9 15 242
-44 -1.15 -3.10 1.0000
15 -4 -9 403 217
296 0.90 2.99 1.0000
15 -4 -9 409 228

301 0.90 2.99 1.0000
13 -4 -9 433 211
419 0.22 0.79 1.0000
13 -4 -9 481 190
466 0.22 0.79 1.0000
11 -4 -9 195 186
315 -2.09 -2.55 1.0000
11 -4 -9 221 161
348 -2.09 -2.55 1.0000
9 -4 -9 1562 295
2297 -9.97 2.74 1.0000
7 -4 -9 823 264
1751 -12.09 -4.94 1.0000
5 -4 -9 1718 195
761 -12.90 -2.78 1.0000
3 -4 -9 1355 198
966 -4.10 2.84 1.0000
1 -4 -9 1629 240
1344 7.91 -3.08 1.0000
-1 -4 -9 1283 259
2710 12.68 -6.88 1.0000
-3 -4 -9 4803 382
6503 11.24 3.42 1.0000
-5 -4 -9 1456 247
2128 5.50 -4.51 1.0000
-5 -4 -9 1649 209
2360 5.50 -4.51 1.0000
-5 -4 -9 1343 200
1991 5.50 -4.51 1.0000
5 4 9 1300 221
1939 5.50 4.51 1.0000
-7 -4 -9 333 151
296 -0.73 -0.51 1.0000
-7 -4 -9 450 156
407 -0.73 -0.51 1.0000
7 4 9 436 162
394 -0.73 0.51 1.0000
-9 -4 -9 375 171
443 2.62 -3.86 1.0000
-9 -4 -9 263 154
316 2.62 -3.86 1.0000
9 4 9 234 171
287 2.62 3.86 1.0000
-11 -4 -9 1093 185

844	-3.40	1.91	1.0000	
11	4	9	718	205
519	-3.40	-1.91	1.0000	
-13	-4	-9	1216	170
697	9.74	-0.47	1.0000	
13	4	9	1076	241
584	9.74	0.47	1.0000	
-15	-4	-9	573	186
658	7.27	1.64	1.0000	
15	4	9	430	259
495	7.27	-1.64	1.0000	
-17	-4	-9	334	191
575	4.98	0.44	1.0000	
17	4	9	135	271
299	4.98	-0.44	1.0000	
-19	-4	-9	95	203
15	5.05	-0.82	1.0000	
19	4	9	35	272
-38	5.05	0.82	1.0000	
-21	-4	-9	191	222
202	0.00	-0.79	1.0000	
21	4	9	1	295
-319	0.00	0.79	1.0000	
20	-5	-9	2	262
-378	0.00	1.16	1.0000	
20	-5	-9	2	321
-12	0.00	1.16	1.0000	
18	-5	-9	89	286
124	2.27	0.93	1.0000	
18	-5	-9	8	234
-107	2.27	0.93	1.0000	
16	-5	-9	128	237
112	0.74	0.82	1.0000	
16	-5	-9	2	214
-52	0.74	0.82	1.0000	
14	-5	-9	1674	287
1764	-0.98	-0.06	1.0000	
14	-5	-9	1122	258
1196	-0.98	-0.06	1.0000	
12	-5	-9	1565	247
1758	3.26	1.13	1.0000	
12	-5	-9	1320	300
1497	3.26	1.13	1.0000	
10	-5	-9	1860	216

1570	2.84		1.14	1.0000
10	-5	-9	1955	285
1657	2.84		1.14	1.0000
8	-5	-9	3486	311
2589	10.09		4.34	1.0000
6	-5	-9	1022	269
1508	10.54		-0.32	1.0000
4	-5	-9	1314	272
1975	5.65		4.60	1.0000
2	-5	-9	1088	253
1567	-5.81		-0.21	1.0000
0	-5	-9	1122	258
1912	-9.15		0.16	1.0000
-2	-5	-9	1572	157
462	-15.25		3.90	1.0000
-4	-5	-9	1777	176
795	-12.90		2.85	1.0000
-4	-5	-9	1784	148
800	-12.90		2.85	1.0000
4	5	9	2239	168
1122	-12.90		-2.85	1.0000
-6	-5	-9	574	151
346	-5.04		-0.79	1.0000
-6	-5	-9	452	150
252	-5.04		-0.79	1.0000
6	5	9	632	166
392	-5.04		0.79	1.0000
-8	-5	-9	708	183
1078	-0.54		7.23	1.0000
-8	-5	-9	1065	206
1517	-0.54		7.23	1.0000
8	5	9	995	220
1429	-0.54		-7.23	1.0000
-10	-5	-9	153	153
168	-0.69		0.17	1.0000
10	5	9	265	205
284	-0.69		-0.17	1.0000
-12	-5	-9	428	180
717	-4.96		1.87	1.0000
12	5	9	315	249
569	-4.96		-1.87	1.0000
-14	-5	-9	353	177
395	-2.10		0.44	1.0000
14	5	9	518	252

569 -2.10 -0.44 1.0000
-16 -5 -9 48 170
97 -4.25 -1.39 1.0000
16 5 9 27 243
-51 -4.25 1.39 1.0000
-18 -5 -9 399 213
625 -4.42 -1.02 1.0000
18 5 9 204 288
371 -4.42 1.02 1.0000
-20 -5 -9 118 223
192 -2.65 0.18 1.0000
20 5 9 9 295
-156 -2.65 -0.18 1.0000
19 -6 -9 25 248
-242 -2.42 -3.55 1.0000
19 -6 -9 25 298
-42 -2.42 -3.55 1.0000
17 -6 -9 3 227
-33 -0.22 1.53 1.0000
17 -6 -9 3 237
-317 -0.22 1.53 1.0000
15 -6 -9 214 219
217 0.99 0.26 1.0000
15 -6 -9 10 225
10 0.99 0.26 1.0000
13 -6 -9 1221 239
1571 3.94 -1.19 1.0000
13 -6 -9 1474 326
1857 3.94 -1.19 1.0000
11 -6 -9 141 188
136 1.21 2.31 1.0000
11 -6 -9 393 167
390 1.21 2.31 1.0000
9 -6 -9 1404 214
1751 -3.14 -5.81 1.0000
9 -6 -9 1425 283
1775 -3.14 -5.81 1.0000
7 -6 -9 653 205
751 -8.20 -1.29 1.0000
5 -6 -9 94 163
16 -5.10 0.61 1.0000
3 -6 -9 551 188
717 3.88 -8.73 1.0000
1 -6 -9 4056 290

2944 9.08 -0.18 1.0000
-1 -6 -9 708 240
1322 17.56 4.23 1.0000
-3 -6 -9 254 181
961 16.23 -9.29 1.0000
-5 -6 -9 1153 198
1666 10.55 0.35 1.0000
-5 -6 -9 1206 236
1731 10.55 0.35 1.0000
5 6 9 1137 223
1651 10.55 -0.35 1.0000
-7 -6 -9 2320 242
2711 3.81 -1.72 1.0000
-7 -6 -9 2292 261
2681 3.81 -1.72 1.0000
7 6 9 2659 284
3079 3.81 1.72 1.0000
-9 -6 -9 2097 238
1795 -1.75 -2.80 1.0000
-9 -6 -9 1962 210
1670 -1.75 -2.80 1.0000
9 6 9 1906 274
1618 -1.75 2.80 1.0000
-11 -6 -9 2078 236
2080 6.52 -1.23 1.0000
11 6 9 1962 286
1963 6.52 1.23 1.0000
-13 -6 -9 6 150
-2 1.62 1.32 1.0000
13 6 9 242 226
174 1.62 -1.32 1.0000
-15 -6 -9 484 192
616 4.06 0.25 1.0000
15 6 9 227 269
316 4.06 -0.25 1.0000
-17 -6 -9 186 197
144 3.18 -0.27 1.0000
17 6 9 370 294
315 3.18 0.27 1.0000
-19 -6 -9 449 215
288 3.84 -0.38 1.0000
19 6 9 564 318
380 3.84 0.38 1.0000
20 -7 -9 438 334

512 1.59 0.39 1.0000
20 -7 -9 18 273
34 1.59 0.39 1.0000
18 -7 -9 146 291
174 0.51 1.68 1.0000
18 -7 -9 109 247
133 0.51 1.68 1.0000
16 -7 -9 89 219
106 -1.00 0.32 1.0000
16 -7 -9 1 226
-168 -1.00 0.32 1.0000
14 -7 -9 1005 236
890 -1.74 3.25 1.0000
14 -7 -9 857 244
750 -1.74 3.25 1.0000
12 -7 -9 4087 306
3216 -6.54 0.98 1.0000
12 -7 -9 4291 355
3398 -6.54 0.98 1.0000
10 -7 -9 64 173
116 3.65 1.76 1.0000
10 -7 -9 46 157
88 3.65 1.76 1.0000
8 -7 -9 13 179
61 2.24 3.11 1.0000
8 -7 -9 133 140
251 2.24 3.11 1.0000
6 -7 -9 2052 299
2426 3.87 1.88 1.0000
4 -7 -9 187 174
160 -0.75 0.49 1.0000
2 -7 -9 1160 253
1239 -6.65 7.78 1.0000
0 -7 -9 2352 347
4581 -16.27 -4.49 1.0000
-2 -7 -9 116 191
844 -16.38 -0.37 1.0000
-2 -7 -9 81 164
739 -16.38 -0.37 1.0000
-4 -7 -9 902 244
1857 -12.10 4.96 1.0000
4 7 9 632 218
1460 -12.10 -4.96 1.0000
-6 -7 -9 598 173

682 -8.14 1.26 1.0000
-6 -7 -9 612 175
698 -8.14 1.26 1.0000
6 7 9 891 211
1020 -8.14 -1.26 1.0000
-8 -7 -9 2131 246
2087 -1.05 0.77 1.0000
-8 -7 -9 1911 227
1869 -1.05 0.77 1.0000
8 7 9 2339 283
2292 -1.05 -0.77 1.0000
-10 -7 -9 49 173
162 0.87 5.71 1.0000
10 7 9 8 182
55 0.87 -5.71 1.0000
-12 -7 -9 4 160
-17 0.63 -1.63 1.0000
12 7 9 223 227
186 0.63 1.63 1.0000
-14 -7 -9 162 163
152 -0.44 -0.80 1.0000
14 7 9 1 244
-92 -0.44 0.80 1.0000
-16 -7 -9 629 206
586 -1.42 1.35 1.0000
16 7 9 19 269
9 -1.42 -1.35 1.0000
-18 -7 -9 151 199
198 -1.04 -2.33 1.0000
18 7 9 180 297
231 -1.04 2.33 1.0000
-20 -7 -9 4 223
-205 -1.75 -0.43 1.0000
20 7 9 4 318
-325 -1.75 0.43 1.0000
19 -8 -9 13 255
-156 -2.11 2.26 1.0000
19 -8 -9 179 312
277 -2.11 2.26 1.0000
17 -8 -9 689 291
658 -0.30 -1.35 1.0000
17 -8 -9 182 238
165 -0.30 -1.35 1.0000
15 -8 -9 20 206

107	5.14	-1.91	1.0000	
15	-8	-9	40	227
-190	5.14	-1.91	1.0000	
13	-8	-9	1651	346
2494	8.06	0.60	1.0000	
13	-8	-9	1957	309
2866	8.06	0.60	1.0000	
11	-8	-9	1318	245
1471	5.09	-2.11	1.0000	
11	-8	-9	1084	249
1220	5.09	-2.11	1.0000	
9	-8	-9	5	169
-91	1.30	-1.32	1.0000	
9	-8	-9	113	148
72	1.30	-1.32	1.0000	
7	-8	-9	1701	285
1666	-1.05	-0.86	1.0000	
7	-8	-9	1968	217
1931	-1.05	-0.86	1.0000	
5	-8	-9	597	213
934	-0.57	-7.26	1.0000	
5	-8	-9	820	168
1214	-0.57	-7.26	1.0000	
3	-8	-9	207	180
235	0.91	0.15	1.0000	
1	-8	-9	2767	262
1589	15.44	-3.21	1.0000	
-1	-8	-9	729	231
1834	14.38	-4.04	1.0000	
-1	-8	-9	761	263
1886	14.38	-4.04	1.0000	
-3	-8	-9	1177	167
274	14.99	4.29	1.0000	
3	8	9	1368	151
369	14.99	-4.29	1.0000	
-5	-8	-9	3871	282
2948	10.07	-4.36	1.0000	
5	8	9	3715	272
2794	10.07	4.36	1.0000	
-7	-8	-9	83	154
180	2.23	-3.12	1.0000	
-7	-8	-9	20	159
-42	2.23	-3.12	1.0000	
7	8	9	20	185

-12	2.23	3.12	1.0000		
-9	-8	-9	802	171	
766	1.52	-0.19	1.0000		
-9	-8	-9	1133	192	
1091	1.52	-0.19	1.0000		
9	8	9	473	212	
445	1.52	0.19	1.0000		
-11	-8	-9	91	165	
203	-4.44	-0.87	1.0000		
11	8	9	90	201	
202	-4.44	0.87	1.0000		
-13	-8	-9	4	154	
-40	1.77	-0.10	1.0000		
13	8	9	4	236	
-27	1.77	0.10	1.0000		
-15	-8	-9	77	175	
86	0.34	0.50	1.0000		
15	8	9	294	273	
312	0.34	-0.50	1.0000		
-17	-8	-9	122	206	
213	3.62	-0.70	1.0000		
17	8	9	61	296	
126	3.62	0.70	1.0000		
-19	-8	-9	3	224	
-31	0.11	-1.46	1.0000		
19	8	9	3	297	
-252	0.11	1.46	1.0000		
18	-9	-9	2	284	
-149	0.57	-0.95	1.0000		
18	-9	-9	78	262	
94	0.57	-0.95	1.0000		
16	-9	-9	732	250	
790	-0.56	4.42	1.0000		
16	-9	-9	1034	300	
1107	-0.56	4.42	1.0000		
14	-9	-9	196	234	
355	-8.97	1.28	1.0000		
14	-9	-9	177	220	
324	-8.97	1.28	1.0000		
12	-9	-9	1203	211	
961	-2.58	2.68	1.0000		
12	-9	-9	906	239	
697	-2.58	2.68	1.0000		
10	-9	-9	1425	311	

2051		-6.78	2.22	1.0000
10	-9	-9	1027	251
1566		-6.78	2.22	1.0000
8	-9	-9	690	217
656		1.48	0.21	1.0000
8	-9	-9	774	174
738		1.48	0.21	1.0000
6	-9	-9	1961	287
1676		-1.63	2.74	1.0000
6	-9	-9	2079	219
1785		-1.63	2.74	1.0000
4	-9	-9	132	179
164		2.62	3.96	1.0000
4	-9	-9	312	145
377		2.62	3.96	1.0000
2	-9	-9	116	186
446		-8.44	-5.51	1.0000
2	-9	-9	129	149
473		-8.44	-5.51	1.0000
0	-9	-9	462	205
973		-9.75	7.65	1.0000
0	-9	-9	423	182
911		-9.75	7.65	1.0000
-2	-9	-9	684	170
130		-12.65	-2.81	1.0000
-4	-9	-9	1803	292
2597		-9.93	-2.74	1.0000
4	9	9	1544	262
2270		-9.93	2.74	1.0000
-6	-9	-9	1383	213
1725		-3.20	5.79	1.0000
-6	-9	-9	1409	253
1755		-3.20	5.79	1.0000
6	9	9	1208	258
1533		-3.20	-5.79	1.0000
-8	-9	-9	5	150
-192		1.39	1.38	1.0000
-8	-9	-9	5	159
-118		1.39	1.38	1.0000
8	9	9	5	188
-239		1.39	-1.38	1.0000
-10	-9	-9	425	155
281		5.79	-1.72	1.0000
-10	-9	-9	425	162

281 5.79 -1.72 1.0000
10 9 9 138 223
49 5.79 1.72 1.0000
-12 -9 -9 688 190
527 6.17 2.00 1.0000
12 9 9 212 214
110 6.17 -2.00 1.0000
-14 -9 -9 8 171
-10 1.55 -1.84 1.0000
14 9 9 8 231
-156 1.55 1.84 1.0000
-16 -9 -9 316 197
401 1.71 -0.98 1.0000
16 9 9 5 281
19 1.71 0.98 1.0000
-18 -9 -9 248 227
247 -1.53 -0.06 1.0000
18 9 9 307 319
306 -1.53 0.06 1.0000
19 -10 -9 3 311
-298 -0.76 -1.37 1.0000
19 -10 -9 18 269
22 -0.76 -1.37 1.0000
17 -10 -9 319 254
307 0.38 1.45 1.0000
17 -10 -9 564 298
549 0.38 1.45 1.0000
15 -10 -9 189 265
367 5.25 -0.40 1.0000
15 -10 -9 37 210
-71 5.25 -0.40 1.0000
13 -10 -9 70 221
316 8.13 -2.02 1.0000
13 -10 -9 23 208
198 8.13 -2.02 1.0000
11 -10 -9 776 198
838 8.57 -0.96 1.0000
11 -10 -9 471 214
495 8.57 -0.96 1.0000
9 -10 -9 148 186
56 5.81 1.78 1.0000
9 -10 -9 168 176
71 5.81 1.78 1.0000
7 -10 -9 118 153

281 0.91 -5.76 1.0000
7 -10 -9 102 192
255 0.91 -5.76 1.0000
5 -10 -9 209 153
225 -0.66 -0.15 1.0000
5 -10 -9 238 189
255 -0.66 -0.15 1.0000
3 -10 -9 418 157
618 2.78 -2.63 1.0000
3 -10 -9 144 186
269 2.78 -2.63 1.0000
1 -10 -9 1330 180
952 4.39 -2.49 1.0000
1 -10 -9 963 208
646 4.39 -2.49 1.0000
-1 -10 -9 2181 325
3320 11.62 2.27 1.0000
-1 -10 -9 2155 302
3287 11.62 2.27 1.0000
-3 -10 -9 402 171
110 10.73 -3.10 1.0000
-5 -10 -9 2182 298
1856 2.97 -1.12 1.0000
5 10 9 2177 247
1851 2.97 1.12 1.0000
-7 -10 -9 22 162
-19 3.64 -1.78 1.0000
-7 -10 -9 22 156
-47 3.64 -1.78 1.0000
7 10 9 33 181
67 3.64 1.78 1.0000
-9 -10 -9 633 202
1069 -6.83 -2.25 1.0000
-9 -10 -9 622 207
1054 -6.83 -2.25 1.0000
9 10 9 424 258
789 -6.83 2.25 1.0000
-11 -10 -9 344 174
535 -4.07 -0.01 1.0000
-11 -10 -9 382 203
582 -4.07 -0.01 1.0000
11 10 9 398 254
600 -4.07 0.01 1.0000
-13 -10 -9 381 198

583 -4.07 -0.26 1.0000
13 10 9 419 264
629 -4.07 0.26 1.0000
-15 -10 -9 204 205
238 0.88 -0.75 1.0000
15 10 9 2 241
-125 0.88 0.75 1.0000
-17 -10 -9 93 223
94 0.01 -0.32 1.0000
17 10 9 267 312
268 0.01 0.32 1.0000
-19 -10 -9 314 254
276 2.14 -1.76 1.0000
19 10 9 139 321
110 2.14 1.76 1.0000
18 -11 -9 11 256
-18 1.30 2.54 1.0000
18 -11 -9 35 311
79 1.30 2.54 1.0000
16 -11 -9 165 244
197 -4.25 -1.59 1.0000
16 -11 -9 179 285
213 -4.25 -1.59 1.0000
14 -11 -9 95 224
206 -0.97 3.87 1.0000
14 -11 -9 132 239
258 -0.97 3.87 1.0000
12 -11 -9 239 215
255 -7.96 1.82 1.0000
12 -11 -9 220 205
231 -7.96 1.82 1.0000
10 -11 -9 708 209
979 -4.16 -0.09 1.0000
10 -11 -9 436 234
653 -4.16 -0.09 1.0000
8 -11 -9 130 182
257 -4.30 0.89 1.0000
8 -11 -9 222 203
384 -4.30 0.89 1.0000
6 -11 -9 1876 252
1892 6.44 1.38 1.0000
6 -11 -9 1836 317
1851 6.44 1.38 1.0000
4 -11 -9 928 222

705	-3.23	-1.92	1.0000	
4	-11	-9	1302	192
1035	-3.23	-1.92	1.0000	
2	-11	-9	230	157
78	4.08	5.28	1.0000	
2	-11	-9	429	195
216	4.08	5.28	1.0000	
0	-11	-9	332	182
49	-4.71	-9.59	1.0000	
0	-11	-9	539	180
155	-4.71	-9.59	1.0000	
-2	-11	-9	852	204
581	-5.70	2.99	1.0000	
-2	-11	-9	645	228
409	-5.70	2.99	1.0000	
-4	-11	-9	150	186
258	-2.09	2.59	1.0000	
4	11	9	90	174
177	-2.09	-2.59	1.0000	
-6	-11	-9	9	196
-11	1.27	-2.30	1.0000	
-6	-11	-9	244	172
240	1.27	-2.30	1.0000	
6	11	9	9	174
-64	1.27	2.30	1.0000	
-8	-11	-9	960	210
1090	5.03	2.02	1.0000	
-8	-11	-9	1015	218
1150	5.03	2.02	1.0000	
8	11	9	709	235
814	5.03	-2.02	1.0000	
-10	-11	-9	447	189
469	8.60	0.86	1.0000	
-10	-11	-9	188	178
159	8.60	0.86	1.0000	
10	11	9	299	254
299	8.60	-0.86	1.0000	
-12	-11	-9	510	190
180	8.17	-1.20	1.0000	
12	11	9	519	257
188	8.17	1.20	1.0000	
-14	-11	-9	8	188
13	5.31	1.42	1.0000	
14	11	9	96	270

256 5.31 -1.42 1.0000
-16 -11 -9 169 224
252 0.00 -2.57 1.0000
16 11 9 9 252
-112 0.00 2.57 1.0000
-18 -11 -9 190 253
229 0.14 2.92 1.0000
18 11 9 174 291
213 0.14 -2.92 1.0000
17 -12 -9 14 301
-52 1.89 -2.58 1.0000
17 -12 -9 241 259
252 1.89 -2.58 1.0000
15 -12 -9 18 230
39 3.48 0.26 1.0000
15 -12 -9 37 271
71 3.48 0.26 1.0000
13 -12 -9 38 221
-118 5.36 0.27 1.0000
13 -12 -9 118 209
35 5.36 0.27 1.0000
11 -12 -9 94 207
-50 8.30 1.38 1.0000
11 -12 -9 685 203
286 8.30 1.38 1.0000
9 -12 -9 416 215
286 6.27 -2.07 1.0000
9 -12 -9 628 215
477 6.27 -2.07 1.0000
7 -12 -9 138 175
111 0.52 1.67 1.0000
7 -12 -9 4 182
-182 0.52 1.67 1.0000
5 -12 -9 303 204
547 -4.86 -1.90 1.0000
5 -12 -9 319 219
569 -4.86 -1.90 1.0000
3 -12 -9 1165 251
1397 1.21 -2.61 1.0000
3 -12 -9 1283 229
1526 1.21 -2.61 1.0000
1 -12 -9 83 188
30 -2.04 2.61 1.0000
1 -12 -9 464 197

323   -2.04   2.61 1.0000
  -1 -12   -9   1419   268
1786   4.14   -0.34 1.0000
  -1 -12   -9   1306   305
1659   4.14   -0.34 1.0000
  -3 -12   -9   125   203
216   2.85   -1.12 1.0000
  -5 -12   -9   869   235
1003   3.22   -1.24 1.0000
   5 12   9   822   227
954   3.22   1.24 1.0000
  -7 -12   -9   4233   358
3355   -6.46   -1.07 1.0000
  -7 -12   -9   4224   285
3347   -6.46   -1.07 1.0000
   7 12   9   3508   344
2716   -6.46   1.07 1.0000
  -9 -12   -9   121   198
51   -2.56   -2.64 1.0000
  -9 -12   -9   118   186
49   -2.56   -2.64 1.0000
   9 12   9   184   215
96   -2.56   2.64 1.0000
 -11 -12   -9   130   193
108   -8.05   -1.76 1.0000
 -11 -12   -9   90   184
-13   -8.05   -1.76 1.0000
  11 12   9   90   246
-160   -8.05   1.76 1.0000
 -13 -12   -9   36   205
13   -3.43   -1.53 1.0000
  13 12   9   197   266
162   -3.43   1.53 1.0000
 -15 -12   -9   39   217
23   -1.35   0.11 1.0000
  15 12   9   2   271
-413   -1.35   -0.11 1.0000
 -17 -12   -9   6   241
5   1.56   -3.17 1.0000
  17 12   9   17   299
-52   1.56   3.17 1.0000
  16 -13   -9   104   303
302   0.73   6.19 1.0000
  16 -13   -9   36   258

```
173     0.73     6.19  1.0000
  14 -13   -9       19       252
-194    -3.38    -1.80  1.0000
  14 -13   -9       19       236
-69    -3.38    -1.80  1.0000
  12 -13   -9      165       213
135    -3.48     1.42  1.0000
  12 -13   -9      334       246
298    -3.48     1.42  1.0000
  10 -13   -9      750       240
1022    -4.05     0.29  1.0000
  10 -13   -9      594       238
838    -4.05     0.29  1.0000
   8 -13   -9      133       195
122     1.83     0.13  1.0000
   8 -13   -9      177       221
164     1.83     0.13  1.0000
   6 -13   -9      358       216
269     1.78    -1.37  1.0000
   6 -13   -9      443       201
344     1.78    -1.37  1.0000
   4 -13   -9     1108       228
616     9.70     0.61  1.0000
   4 -13   -9     1103       204
612     9.70     0.61  1.0000
   2 -13   -9      232       198
423     1.49    -5.40  1.0000
   2 -13   -9      204       215
384     1.49    -5.40  1.0000
   0 -13   -9       50       196
92     5.89     3.86  1.0000
   0 -13   -9       35       199
44     5.89     3.86  1.0000
  -2 -13   -9      338       250
405    -0.35    -4.19  1.0000
  -2 -13   -9      289       217
349    -0.35    -4.19  1.0000
  -4 -13   -9      473       220
455     0.29    -0.82  1.0000
   4  13    9      165       214
154     0.29     0.82  1.0000
   6  13    9      916       260
1226     3.99    -1.17  1.0000
  -8 -13   -9     1285       339
```

2040 8.08 -0.51 1.0000
-8 -13 -9 1150 262
1869 8.08 -0.51 1.0000
8 13 9 1212 337
1948 8.08 0.51 1.0000
-10 -13 -9 50 196
269 8.08 2.03 1.0000
-10 -13 -9 58 231
286 8.08 2.03 1.0000
10 13 9 17 231
176 8.08 -2.03 1.0000
-12 -13 -9 39 199
-95 5.39 -0.34 1.0000
-12 -13 -9 39 193
-267 5.39 -0.34 1.0000
12 13 9 39 261
0 5.39 0.34 1.0000
-14 -13 -9 87 217
207 4.67 -1.86 1.0000
14 13 9 22 274
93 4.67 1.86 1.0000
-16 -13 -9 87 256
243 1.20 5.31 1.0000
16 13 9 49 301
176 1.20 -5.31 1.0000
15 -14 -9 584 265
555 1.57 -3.07 1.0000
15 -14 -9 437 295
409 1.57 -3.07 1.0000
13 -14 -9 13 259
73 4.80 1.88 1.0000
13 -14 -9 11 221
65 4.80 1.88 1.0000
11 -14 -9 41 207
-10 5.38 -1.42 1.0000
11 -14 -9 6 241
16 5.38 -1.42 1.0000
9 -14 -9 28 210
28 1.56 1.87 1.0000
9 -14 -9 253 229
270 1.56 1.87 1.0000
7 -14 -9 102 197
96 -0.39 0.83 1.0000
7 -14 -9 197 215

188 -0.39 0.83 1.0000
5 -14 -9 341 226
382 -2.15 -0.42 1.0000
5 -14 -9 264 215
300 -2.15 -0.42 1.0000
3 -14 -9 45 223
332 -10.57 1.38 1.0000
3 -14 -9 64 208
383 -10.57 1.38 1.0000
1 -14 -9 1543 291
1653 -2.02 0.49 1.0000
1 -14 -9 1732 315
1849 -2.02 0.49 1.0000
-1 -14 -9 18 250
-58 -2.72 -2.48 1.0000
-1 -14 -9 369 216
348 -2.72 -2.48 1.0000
-3 -14 -9 1525 320
1382 1.45 1.10 1.0000
-3 -14 -9 1294 245
1162 1.45 1.10 1.0000
-5 -14 -9 876 258
941 -0.94 0.13 1.0000
5 14 9 909 253
975 -0.94 -0.13 1.0000
-7 -14 -9 676 218
578 -1.73 -3.27 1.0000
-7 -14 -9 896 256
785 -1.73 -3.27 1.0000
7 14 9 764 254
663 -1.73 3.27 1.0000
-9 -14 -9 207 233
369 -9.03 -1.40 1.0000
-9 -14 -9 240 220
421 -9.03 -1.40 1.0000
9 14 9 178 242
324 -9.03 1.40 1.0000
-11 -14 -9 179 250
326 -1.01 -3.95 1.0000
-11 -14 -9 128 221
255 -1.01 -3.95 1.0000
11 14 9 197 245
350 -1.01 3.95 1.0000
-13 -14 -9 162 236

126   -3.43   1.85 1.0000
  13 14   9   39   281
13   -3.43   -1.85 1.0000
 -15 -14   -9   234   251
229   -1.13   -4.61 1.0000
  15 14   9   380   328
389   -1.13   4.61 1.0000
  14 -15   -9   30   232
-206   -1.15   4.62 1.0000
  14 -15   -9   300   290
304   -1.15   4.62 1.0000
  12 -15   -9   157   254
126   -1.27   -0.12 1.0000
  12 -15   -9   2   227
-20   -1.27   -0.12 1.0000
  10 -15   -9   2   235
-7   0.97   0.79 1.0000
  10 -15   -9   2   214
-24   0.97   0.79 1.0000
   8 -15   -9   23   223
28   0.31   -0.45 1.0000
   8 -15   -9   428   225
448   0.31   -0.45 1.0000
   6 -15   -9   453   236
581   4.08   -0.20 1.0000
   6 -15   -9   249   216
342   4.08   -0.20 1.0000
   4 -15   -9   346   217
398   7.40   -1.67 1.0000
   4 -15   -9   709   238
816   7.40   -1.67 1.0000
   2 -15   -9   239   250
818   11.59   -0.75 1.0000
   2 -15   -9   187   233
718   11.59   -0.75 1.0000
   0 -15   -9   262   213
242   2.36   -3.56 1.0000
   0 -15   -9   24   234
-6   2.36   -3.56 1.0000
  -2 -15   -9   254   260
195   3.79   3.08 1.0000
  -2 -15   -9   228   223
171   3.79   3.08 1.0000
  -4 -15   -9   348   218

249 0.95 -3.03 1.0000
-6 -15 -9 1 219
-31 0.94 -0.25 1.0000
6 15 9 1 222
-101 0.94 0.25 1.0000
-8 -15 -9 42 238
-182 5.26 1.87 1.0000
-8 -15 -9 13 214
91 5.26 1.87 1.0000
8 15 9 27 250
126 5.26 -1.87 1.0000
-10 -15 -9 25 245
97 5.34 0.42 1.0000
-10 -15 -9 270 230
477 5.34 0.42 1.0000
10 15 9 23 256
99 5.34 -0.42 1.0000
-12 -15 -9 152 240
221 3.40 -0.20 1.0000
-12 -15 -9 52 262
90 3.40 -0.20 1.0000
12 15 9 15 251
-103 3.40 0.20 1.0000
-14 -15 -9 979 258
948 1.59 3.08 1.0000
14 15 9 383 316
357 1.59 -3.08 1.0000
13 -16 -9 39 241
-33 1.27 -5.26 1.0000
13 -16 -9 95 289
255 1.27 -5.26 1.0000
11 -16 -9 8 253
-49 0.08 2.51 1.0000
11 -16 -9 143 233
218 0.08 2.51 1.0000
9 -16 -9 138 226
195 1.70 0.95 1.0000
9 -16 -9 219 267
289 1.70 0.95 1.0000
7 -16 -9 219 219
195 -1.28 -1.37 1.0000
7 -16 -9 662 260
623 -1.28 -1.37 1.0000
5 -16 -9 37 200

79 -4.23 1.40 1.0000
5 -16 -9 12 249
20 -4.23 1.40 1.0000
3 -16 -9 945 230
669 -6.87 2.24 1.0000
3 -16 -9 891 255
623 -6.87 2.24 1.0000
1 -16 -9 95 210
-54 -7.97 -2.84 1.0000
1 -16 -9 260 250
260 -7.97 -2.84 1.0000
-1 -16 -9 12 264
-131 -1.29 2.65 1.0000
-1 -16 -9 299 220
242 -1.29 2.65 1.0000
-3 -16 -9 5 276
-390 -1.26 -1.59 1.0000
-3 -16 -9 5 220
0 -1.26 -1.59 1.0000
-5 -16 -9 2 230
-29 0.80 -0.81 1.0000
-7 -16 -9 34 245
46 -1.05 -0.37 1.0000
-7 -16 -9 2 221
-284 -1.05 -0.37 1.0000
7 16 9 423 268
464 -1.05 0.37 1.0000
-9 -16 -9 806 258
873 -0.59 -4.41 1.0000
-9 -16 -9 1040 294
1120 -0.59 -4.41 1.0000
9 16 9 802 296
865 -0.59 4.41 1.0000
-11 -16 -9 416 254
476 -4.25 1.63 1.0000
-11 -16 -9 127 269
149 -4.25 1.63 1.0000
11 16 9 226 280
266 -4.25 -1.63 1.0000
-13 -16 -9 28 265
156 0.63 -6.20 1.0000
-13 -16 -9 66 267
234 0.63 -6.20 1.0000
13 16 9 26 281

152 0.63 6.20 1.0000
12 -17 -9 60 234
116 1.51 3.26 1.0000
12 -17 -9 6 274
17 1.51 3.26 1.0000
10 -17 -9 154 241
156 0.07 0.33 1.0000
10 -17 -9 0 273
-235 0.07 0.33 1.0000
8 -17 -9 73 214
146 3.67 0.72 1.0000
8 -17 -9 128 266
222 3.67 0.72 1.0000
6 -17 -9 494 225
430 3.14 0.32 1.0000
6 -17 -9 532 271
466 3.14 0.32 1.0000
4 -17 -9 418 275
686 5.04 -0.39 1.0000
4 -17 -9 277 231
501 5.04 -0.39 1.0000
2 -17 -9 387 254
769 7.13 0.34 1.0000
2 -17 -9 726 303
1225 7.13 0.34 1.0000
0 -17 -9 26 221
-68 4.41 0.16 1.0000
0 -17 -9 44 278
128 4.41 0.16 1.0000
-2 -17 -9 130 232
217 0.92 -4.02 1.0000
-2 -17 -9 23 284
-165 0.92 -4.02 1.0000
-4 -17 -9 15 230
-24 -1.22 3.09 1.0000
-4 -17 -9 15 313
-255 -1.22 3.09 1.0000
-6 -17 -9 3 240
-89 -0.06 -1.56 1.0000
6 17 9 3 244
-98 -0.06 1.56 1.0000
-8 -17 -9 168 252
151 -0.27 1.37 1.0000
-8 -17 -9 212 271

193	-0.27	1.37	1.0000	
8	17	9	3	255
-51	-0.27	-1.37	1.0000	
-10	-17	-9	437	291
425	0.37	-1.44	1.0000	
-10	-17	-9	725	262
710	0.37	-1.44	1.0000	
10	17	9	652	301
636	0.37	1.44	1.0000	
-12	-17	-9	207	280
215	1.91	2.57	1.0000	
-12	-17	-9	48	296
44	1.91	2.57	1.0000	
12	17	9	85	278
85	1.91	-2.57	1.0000	
11	-18	-9	234	258
281	0.15	-2.97	1.0000	
11	-18	-9	95	288
122	0.15	-2.97	1.0000	
9	-18	-9	3	216
-173	-1.44	0.02	1.0000	
9	-18	-9	3	265
-333	-1.44	0.02	1.0000	
7	-18	-9	112	278
153	-1.07	2.34	1.0000	
7	-18	-9	164	242
214	-1.07	2.34	1.0000	
5	-18	-9	215	289
388	-4.44	0.98	1.0000	
5	-18	-9	435	255
671	-4.44	0.98	1.0000	
3	-18	-9	164	228
219	-4.92	-3.62	1.0000	
3	-18	-9	379	287
485	-4.92	-3.62	1.0000	
1	-18	-9	24	234
-26	-2.20	3.60	1.0000	
1	-18	-9	375	292
451	-2.20	3.60	1.0000	
-1	-18	-9	75	308
16	-3.75	-1.66	1.0000	
-3	-18	-9	57	305
68	0.54	0.32	1.0000	
-7	-18	-9	199	266

231 0.53 -1.68 1.0000
-7 -18 -9 286 265
325 0.53 -1.68 1.0000
7 18 9 384 274
431 0.53 1.68 1.0000
-9 -18 -9 455 275
499 0.54 1.05 1.0000
-9 -18 -9 272 293
306 0.54 1.05 1.0000
9 18 9 153 287
179 0.54 -1.05 1.0000
-11 -18 -9 11 284
-214 1.37 -2.59 1.0000
-11 -18 -9 283 309
398 1.37 -2.59 1.0000
11 18 9 339 317
464 1.37 2.59 1.0000
10 -19 -9 44 293
26 2.05 1.80 1.0000
10 -19 -9 10 217
-234 2.05 1.80 1.0000
8 -19 -9 28 261
48 0.17 1.46 1.0000
8 -19 -9 72 284
103 0.17 1.46 1.0000
6 -19 -9 226 297
115 3.88 0.38 1.0000
6 -19 -9 116 241
41 3.88 0.38 1.0000
4 -19 -9 547 290
305 5.08 0.75 1.0000
4 -19 -9 35 223
-208 5.08 0.75 1.0000
2 -19 -9 0 279
-66 -0.28 0.08 1.0000
0 -19 -9 132 299
188 2.72 -1.05 1.0000
-2 -19 -9 5 311
-107 -0.26 2.01 1.0000
-4 -19 -9 17 333
-321 -2.81 -2.15 1.0000
-6 -19 -9 22 269
1 -2.50 3.67 1.0000
-8 -19 -9 12 270

-66 -2.01 -2.31 1.0000
8 19 9 12 276
-106 -2.01 2.31 1.0000
-10 -19 -9 387 286
416 -0.71 1.35 1.0000
-10 -19 -9 108 292
122 -0.71 1.35 1.0000
10 19 9 70 303
81 -0.71 -1.35 1.0000
7 -20 -9 240 271
299 -1.82 0.32 1.0000
7 -20 -9 37 299
61 -1.82 0.32 1.0000
5 -20 -9 45 244
93 -2.67 -0.10 1.0000
5 -20 -9 3 291
22 -2.67 -0.10 1.0000
3 -20 -9 178 301
241 -0.60 2.80 1.0000
1 -20 -9 343 316
419 -3.28 -1.35 1.0000
-1 -20 -9 1 323
-275 -0.26 1.01 1.0000
-3 -20 -9 5 334
-228 1.97 -0.49 1.0000
-5 -20 -9 73 376
92 0.06 -1.18 1.0000
-7 -20 -9 373 294
443 1.69 -0.34 1.0000
4 -21 -9 100 320
109 -0.05 0.77 1.0000
2 -21 -9 7 311
-132 1.96 -1.16 1.0000
0 -21 -9 68 334
62 0.29 0.59 1.0000
-2 -21 -9 490 382
662 -3.09 -0.59 1.0000
-4 -21 -9 6 373
-253 -0.82 1.89 1.0000
2 20 -10 314 361
298 1.13 0.42 1.0000
-2 -20 10 88 352
79 1.13 -0.42 1.0000
0 20 -10 141 351

186   -1.65   -0.61 1.0000
0 -20 10   4   338
-89   -1.65   0.61 1.0000
-2 20 -10   254   372
351   -0.42   2.52 1.0000
2 -20 10   280   334
381   -0.42   -2.52 1.0000
7 19 -10   900   338
845   -0.70   0.42 1.0000
-7 -19 10   89   395
72   -0.70   -0.42 1.0000
5 19 -10   325   302
340   -0.15   0.93 1.0000
-5 -19 10   1   349
1   -0.15   -0.93 1.0000
3 19 -10   592   317
523   -1.36   1.04 1.0000
-3 -19 10   343   338
290   -1.36   -1.04 1.0000
1 19 -10   305   342
323   0.65   -1.24 1.0000
-1 -19 10   188   332
201   0.65   1.24 1.0000
-1 19 -10   184   318
152   -0.78   -0.71 1.0000
1 -19 10   253   312
216   -0.78   0.71 1.0000
-3 19 -10   47   362
28   -0.18   -1.82 1.0000
-3 19 -10   185   205
146   -0.18   -1.82 1.0000
3 -19 10   33   302
17   -0.18   1.82 1.0000
-5 19 -10   192   220
110   2.11   -2.34 1.0000
-5 19 -10   403   374
280   2.11   -2.34 1.0000
5 -19 10   13   300
-15   2.11   2.34 1.0000
-7 19 -10   254   228
281   -0.18   0.78 1.0000
-7 19 -10   382   394
415   -0.18   0.78 1.0000
7 -19 10   16   303

23 -0.18 -0.78 1.0000
8 18 -10 943 339
925 -0.20 -1.11 1.0000
-8 -18 10 2 369
-486 -0.20 1.11 1.0000
6 18 -10 828 310
712 -2.04 -0.30 1.0000
-6 -18 10 202 372
146 -2.04 0.30 1.0000
4 18 -10 0 272
-40 -0.09 0.32 1.0000
-4 -18 10 374 326
359 -0.09 -0.32 1.0000
2 18 -10 665 301
697 -2.94 -1.00 1.0000
-2 -18 10 348 320
367 -2.94 1.00 1.0000
0 18 -10 426 319
555 2.08 4.48 1.0000
0 -18 10 32 286
-412 2.08 -4.48 1.0000
-2 18 -10 292 201
267 -0.40 1.04 1.0000
-2 18 -10 179 329
160 -0.40 1.04 1.0000
2 -18 10 415 298
385 -0.40 -1.04 1.0000
-4 18 -10 41 212
137 -3.07 3.50 1.0000
-4 18 -10 576 350
860 -3.07 3.50 1.0000
4 -18 10 210 284
392 -3.07 -3.50 1.0000
-6 18 -10 11 342
-91 1.31 2.49 1.0000
-6 18 -10 186 226
267 1.31 2.49 1.0000
6 -18 10 104 277
165 1.31 -2.49 1.0000
-8 18 -10 404 234
482 -0.42 1.70 1.0000
-8 18 -10 286 385
352 -0.42 1.70 1.0000
8 -18 10 46 290

74 -0.42 -1.70 1.0000
11 17 -10 979 356
1010 -0.21 0.61 1.0000
9 17 -10 547 324
522 -0.30 -2.25 1.0000
-9 -17 10 59 377
46 -0.30 2.25 1.0000
7 17 -10 395 285
496 1.59 1.41 1.0000
-7 -17 10 228 343
306 1.59 -1.41 1.0000
5 17 -10 595 293
524 2.21 -0.44 1.0000
-5 -17 10 583 329
512 2.21 0.44 1.0000
3 17 -10 329 282
443 1.36 -2.15 1.0000
-3 -17 10 493 310
632 1.36 2.15 1.0000
1 17 -10 242 276
290 1.10 -1.15 1.0000
-1 -17 10 165 283
205 1.10 1.15 1.0000
-1 17 -10 195 199
307 -2.32 -4.32 1.0000
-1 17 -10 467 316
643 -2.32 -4.32 1.0000
1 -17 10 228 288
349 -2.32 4.32 1.0000
-3 17 -10 290 206
445 2.73 -3.13 1.0000
-3 17 -10 632 331
856 2.73 -3.13 1.0000
3 -17 10 196 274
325 2.73 3.13 1.0000
-5 17 -10 428 216
430 -0.13 -2.63 1.0000
-5 17 -10 572 330
575 -0.13 -2.63 1.0000
5 -17 10 25 270
17 -0.13 2.63 1.0000
-7 17 -10 247 215
341 2.06 -1.51 1.0000
-7 17 -10 357 336

468	2.06	-1.51	1.0000	
7	-17	10	348	274
458	2.06	1.51	1.0000	
-9	17	-10	97	360
143	1.75	0.57	1.0000	
-9	17	-10	105	214
153	1.75	0.57	1.0000	
9	-17	10	90	277
134	1.75	-0.57	1.0000	
-11	17	-10	14	227
2	2.07	-0.14	1.0000	
-11	17	-10	165	365
110	2.07	-0.14	1.0000	
11	-17	10	330	301
249	2.07	0.14	1.0000	
12	16	-10	433	351
534	-2.13	0.83	1.0000	
10	16	-10	131	297
134	1.70	1.80	1.0000	
-10	-16	10	149	372
154	1.70	-1.80	1.0000	
8	16	-10	175	272
266	-2.58	-0.94	1.0000	
-8	-16	10	103	342
175	-2.58	0.94	1.0000	
6	16	-10	615	262
649	-2.56	0.33	1.0000	
-6	-16	10	497	329
527	-2.56	-0.33	1.0000	
4	16	-10	185	243
278	-3.30	0.05	1.0000	
-4	-16	10	45	273
93	-3.30	-0.05	1.0000	
2	16	-10	55	250
11	-1.77	4.88	1.0000	
-2	-16	10	520	276
435	-1.77	-4.88	1.0000	
0	16	-10	214	176
301	-2.36	1.56	1.0000	
0	16	-10	174	277
253	-2.36	1.56	1.0000	
0	-16	10	6	245
22	-2.36	-1.56	1.0000	
-2	16	-10	648	281

316	3.37	5.78	1.0000	
-2	16	-10	572	183
264	3.37	5.78	1.0000	
2	-16	10	546	252
246	3.37	-5.78	1.0000	
-4	16	-10	1624	341
1289	-0.02	4.76	1.0000	
-4	16	-10	946	217
692	-0.02	4.76	1.0000	
4	-16	10	496	258
317	-0.02	-4.76	1.0000	
-6	16	-10	419	202
326	-0.45	2.60	1.0000	
-6	16	-10	527	325
422	-0.45	2.60	1.0000	
6	-16	10	9	244
-303	-0.45	-2.60	1.0000	
-8	16	-10	616	338
676	1.16	0.31	1.0000	
-8	16	-10	296	213
338	1.16	0.31	1.0000	
8	-16	10	138	258
166	1.16	-0.31	1.0000	
-10	16	-10	475	343
460	-0.73	0.45	1.0000	
-10	16	-10	291	216
279	-0.73	0.45	1.0000	
10	-16	10	288	281
276	-0.73	-0.45	1.0000	
-12	16	-10	141	216
126	-0.55	0.37	1.0000	
-12	16	-10	671	350
637	-0.55	0.37	1.0000	
12	-16	10	352	292
328	-0.55	-0.37	1.0000	
13	15	-10	717	380
915	3.31	-0.52	1.0000	
13	15	-10	255	305
377	3.31	-0.52	1.0000	
11	15	-10	397	321
598	-0.67	-5.10	1.0000	
9	15	-10	286	271
299	2.65	-2.23	1.0000	
-9	-15	10	37	334

30 2.65 2.23 1.0000
7 15 -10 354 257
628 4.57 -2.91 1.0000
-7 -15 10 281 311
529 4.57 2.91 1.0000
5 15 -10 742 246
621 2.58 -0.57 1.0000
-5 -15 10 277 292
203 2.58 0.57 1.0000
3 15 -10 3 225
56 5.07 -0.96 1.0000
-3 -15 10 74 266
210 5.07 0.96 1.0000
1 15 -10 350 167
494 2.21 -5.39 1.0000
1 15 -10 401 261
556 2.21 -5.39 1.0000
-1 -15 10 251 261
373 2.21 5.39 1.0000
-1 15 -10 485 186
402 2.11 -3.12 1.0000
-1 15 -10 281 272
215 2.11 -3.12 1.0000
1 -15 10 189 233
135 2.11 3.12 1.0000
-3 15 -10 840 312
1487 -3.45 -7.57 1.0000
-3 15 -10 604 214
1167 -3.45 -7.57 1.0000
3 -15 10 288 236
704 -3.45 7.57 1.0000
-5 15 -10 240 307
266 1.20 -3.20 1.0000
-5 15 -10 182 193
203 1.20 -3.20 1.0000
5 -15 10 438 243
475 1.20 3.20 1.0000
-7 15 -10 672 214
705 -0.09 -1.53 1.0000
-7 15 -10 859 338
897 -0.09 -1.53 1.0000
7 -15 10 322 259
344 -0.09 1.53 1.0000
-9 15 -10 241 200

305   2.93   1.42 1.0000
  -9 15 -10   198   308
256   2.93   1.42 1.0000
   9 -15 10   197   265
256   2.93   -1.42 1.0000
 -11 15 -10   868   344
762   0.68   2.12 1.0000
 -11 15 -10   539   216
455   0.68   2.12 1.0000
  11 -15 10   166   268
120   0.68   -2.12 1.0000
 -13 15 -10   201   212
359   3.92   1.37 1.0000
 -13 15 -10   293   338
480   3.92   1.37 1.0000
  13 -15 10   23   284
-115   3.92   -1.37 1.0000
  14 14 -10   358   373
445   -1.75   -1.02 1.0000
  14 14 -10   407   334
499   -1.75   -1.02 1.0000
  12 14 -10   450   282
410   2.89   2.83 1.0000
  12 14 -10   749   339
703   2.89   2.83 1.0000
  10 14 -10   287   288
205   -1.79   1.55 1.0000
   8 14 -10   207   247
254   -2.58   5.61 1.0000
  -8 -14 10   51   309
-34   -2.58   -5.61 1.0000
   6 14 -10   15   240
27   -2.24   0.38 1.0000
  -6 -14 10   7   255
-255   -2.24   -0.38 1.0000
   4 14 -10   549   175
609   -4.52   1.17 1.0000
   4 14 -10   381   251
426   -4.52   1.17 1.0000
  -4 -14 10   343   226
387   -4.52   -1.17 1.0000
   2 14 -10   277   152
199   -2.25   0.62 1.0000
   2 14 -10   42   240

15 -2.25 0.62 1.0000
-2 -14 10 195 226
130 -2.25 -0.62 1.0000
0 14 -10 723 269
950 -0.71 3.40 1.0000
0 14 -10 898 203
1149 -0.71 3.40 1.0000
0 -14 10 880 232
1129 -0.71 -3.40 1.0000
-2 14 -10 240 264
352 -1.08 5.37 1.0000
-2 14 -10 339 178
476 -1.08 5.37 1.0000
2 -14 10 274 202
396 -1.08 -5.37 1.0000
-4 14 -10 237 291
414 5.72 6.52 1.0000
-4 14 -10 78 181
137 5.72 6.52 1.0000
4 -14 10 84 226
153 5.72 -6.52 1.0000
-6 14 -10 245 193
302 2.35 1.67 1.0000
-6 14 -10 518 300
604 2.35 1.67 1.0000
6 -14 10 130 217
169 2.35 -1.67 1.0000
-8 14 -10 447 206
455 -0.19 0.51 1.0000
-8 14 -10 414 309
422 -0.19 0.51 1.0000
8 -14 10 138 241
142 -0.19 -0.51 1.0000
-10 14 -10 33 302
62 1.42 -1.20 1.0000
-10 14 -10 5 187
-67 1.42 -1.20 1.0000
10 -14 10 188 259
251 1.42 1.20 1.0000
-12 14 -10 11 300
56 1.88 -3.97 1.0000
-12 14 -10 26 198
-137 1.88 -3.97 1.0000
12 -14 10 69 276

164 1.88 3.97 1.0000
-14 14 -10 229 216
300 -1.82 0.99 1.0000
-14 14 -10 5 290
19 -1.82 0.99 1.0000
14 -14 10 123 275
176 -1.82 -0.99 1.0000
15 13 -10 575 395
830 3.95 -1.42 1.0000
15 13 -10 170 310
320 3.95 -1.42 1.0000
13 13 -10 238 291
374 -1.42 -3.16 1.0000
13 13 -10 299 324
449 -1.42 -3.16 1.0000
11 13 -10 739 291
1056 4.29 -3.72 1.0000
11 13 -10 561 309
839 4.29 -3.72 1.0000
9 13 -10 176 264
418 3.46 -5.55 1.0000
7 13 -10 253 249
525 2.51 -5.55 1.0000
-7 -13 10 174 264
409 2.51 5.55 1.0000
5 13 -10 486 163
251 5.78 -1.60 1.0000
5 13 -10 316 235
132 5.78 -1.60 1.0000
-5 -13 10 708 250
416 5.78 1.60 1.0000
3 13 -10 422 150
340 3.79 -0.97 1.0000
3 13 -10 585 243
491 3.79 -0.97 1.0000
-3 -13 10 259 208
191 3.79 0.97 1.0000
1 13 -10 181 163
287 3.22 2.56 1.0000
1 13 -10 200 233
311 3.22 2.56 1.0000
-1 -13 10 245 203
370 3.22 -2.56 1.0000
-1 13 -10 2135 227

1572	0.24	-5.94	1.0000	
-1	13	-10	2376	301
1780	0.24	-5.94	1.0000	
1	-13	10	2285	273
1702	0.24	5.94	1.0000	
-3	13	-10	718	190
706	-0.53	-4.16	1.0000	
-3	13	-10	1225	297
1217	-0.53	-4.16	1.0000	
3	-13	10	747	200
733	-0.53	4.16	1.0000	
-5	13	-10	824	306
1134	-4.62	-5.43	1.0000	
-5	13	-10	591	205
851	-4.62	-5.43	1.0000	
5	-13	10	558	231
812	-4.62	5.43	1.0000	
-7	13	-10	119	188
156	1.68	0.95	1.0000	
-7	13	-10	559	295
638	1.68	0.95	1.0000	
7	-13	10	85	211
116	1.68	-0.95	1.0000	
-9	13	-10	471	290
436	0.06	1.51	1.0000	
-9	13	-10	503	191
467	0.06	1.51	1.0000	
9	-13	10	413	242
381	0.06	-1.51	1.0000	
-11	13	-10	190	288
185	2.74	4.81	1.0000	
-11	13	-10	41	188
-79	2.74	4.81	1.0000	
11	-13	10	217	258
223	2.74	-4.81	1.0000	
-13	13	-10	217	212
349	-1.45	3.19	1.0000	
-13	13	-10	201	287
328	-1.45	3.19	1.0000	
13	-13	10	373	286
541	-1.45	-3.19	1.0000	
-15	13	-10	71	211
141	3.37	0.46	1.0000	
-15	13	-10	15	252

-55 3.37 0.46 1.0000
15 -13 10 171 288
275 3.37 -0.46 1.0000
16 12 -10 302 381
278 -0.53 -0.42 1.0000
16 12 -10 1 302
-143 -0.53 -0.42 1.0000
14 12 -10 27 263
-67 1.91 4.05 1.0000
14 12 -10 48 232
131 1.91 4.05 1.0000
12 12 -10 344 265
309 -0.37 0.79 1.0000
12 12 -10 177 273
152 -0.37 0.79 1.0000
10 12 -10 43 245
0 -1.02 5.61 1.0000
8 12 -10 234 242
173 -0.29 2.28 1.0000
8 12 -10 7 156
-215 -0.29 2.28 1.0000
-8 -12 10 190 261
135 -0.29 -2.28 1.0000
6 12 -10 315 225
256 -3.11 4.00 1.0000
6 12 -10 374 165
313 -3.11 4.00 1.0000
-6 -12 10 34 228
-46 -3.11 -4.00 1.0000
4 12 -10 756 251
868 -1.82 -0.58 1.0000
4 12 -10 1349 186
1498 -1.82 -0.58 1.0000
-4 -12 10 831 228
950 -1.82 0.58 1.0000
2 12 -10 1169 279
1440 -4.62 -4.63 1.0000
2 12 -10 1111 199
1374 -4.62 -4.63 1.0000
-2 -12 10 1299 268
1586 -4.62 4.63 1.0000
0 12 -10 887 186
880 2.82 -0.17 1.0000
0 12 -10 767 253

760 2.82 -0.17 1.0000
0 -12 10 622 189
614 2.82 0.17 1.0000
-2 12 -10 2036 242
2403 1.95 3.59 1.0000
-2 12 -10 1990 378
2353 1.95 3.59 1.0000
2 -12 10 1765 263
2107 1.95 -3.59 1.0000
-4 12 -10 807 186
749 2.62 0.85 1.0000
-4 12 -10 1049 278
984 2.62 0.85 1.0000
4 -12 10 681 204
627 2.62 -0.85 1.0000
-6 12 -10 367 290
641 4.49 3.05 1.0000
-6 12 -10 342 185
608 4.49 3.05 1.0000
6 -12 10 180 190
382 4.49 -3.05 1.0000
-8 12 -10 649 194
516 1.47 -2.01 1.0000
-8 12 -10 1409 325
1209 1.47 -2.01 1.0000
8 -12 10 815 228
665 1.47 2.01 1.0000
-10 12 -10 508 181
262 2.74 -5.06 1.0000
-10 12 -10 599 270
329 2.74 -5.06 1.0000
10 -12 10 175 232
46 2.74 5.06 1.0000
-12 12 -10 119 188
98 -0.50 -0.81 1.0000
-12 12 -10 260 277
229 -0.50 -0.81 1.0000
12 -12 10 220 246
192 -0.50 0.81 1.0000
-14 12 -10 152 197
121 2.80 -2.78 1.0000
-14 12 -10 368 266
331 2.80 -2.78 1.0000
14 -12 10 238 272

```
201      2.80      2.78  1.0000
 -16   12 -10        59       204
99    -2.15    -0.87  1.0000
  16 -12   10       104       274
156    -2.15      0.87  1.0000
  17   11 -10       458       278
360      2.13      0.18  1.0000
  17   11 -10       432       308
337      2.13      0.18  1.0000
  15   11 -10       558       254
473      0.76    -2.19  1.0000
  15   11 -10        85       269
52      0.76    -2.19  1.0000
  13   11 -10       145       259
145      2.72    -4.88  1.0000
  13   11 -10        71       226
55      2.72    -4.88  1.0000
  11   11 -10        85       226
188      0.92    -4.43  1.0000
  11   11 -10       319       231
505      0.92    -4.43  1.0000
   9   11 -10       524       222
361      2.98    -6.18  1.0000
   7   11 -10       451       214
546      1.95    -2.02  1.0000
  -7 -11   10       700       248
820      1.95      2.02  1.0000
   5   11 -10        35       211
30      2.52    -0.72  1.0000
   5   11 -10       186       153
188      2.52    -0.72  1.0000
  -5 -11   10        31       192
26      2.52      0.72  1.0000
   3   11 -10       673       229
296      5.86      6.20  1.0000
   3   11 -10       899       156
455      5.86      6.20  1.0000
  -3 -11   10       749       181
348      5.86    -6.20  1.0000
   1   11 -10      1286       194
1200      0.18      1.22  1.0000
   1   11 -10       951       257
877      0.18      1.22  1.0000
  -1 -11   10      1122       192
```

1042   0.18   -1.22 1.0000
  -1 11 -10   857   170
687   -2.21   2.93 1.0000
  -1 11 -10   709   251
554   -2.21   2.93 1.0000
   1 -11 10   682   167
531   -2.21   -2.93 1.0000
  -3 11 -10   653   178
791   -1.93   -2.55 1.0000
  -3 11 -10   893   277
1054   -1.93   -2.55 1.0000
   3 -11 10   635   180
771   -1.93   2.55 1.0000
  -5 11 -10   77   167
76   -3.20   0.01 1.0000
  -5 11 -10   435   261
453   -3.20   0.01 1.0000
   5 -11 10   13   167
2   -3.20   -0.01 1.0000
  -7 11 -10   794   288
753   -2.35   -0.35 1.0000
  -7 11 -10   400   180
369   -2.35   -0.35 1.0000
   7 -11 10   439   185
407   -2.35   0.35 1.0000
  -9 11 -10   215   279
391   -0.36   5.57 1.0000
  -9 11 -10   41   182
121   -0.36   5.57 1.0000
   9 -11 10   119   212
255   -0.36   -5.57 1.0000
 -11 11 -10   505   274
737   0.90   4.45 1.0000
 -11 11 -10   324   198
513   0.90   4.45 1.0000
  11 -11 10   418   252
631   0.90   -4.45 1.0000
 -13 11 -10   1186   301
1580   4.33   3.64 1.0000
 -13 11 -10   714   225
1023   4.33   3.64 1.0000
  13 -11 10   582   283
860   4.33   -3.64 1.0000
 -15 11 -10   137   239

254 -0.73 5.11 1.0000

-15 11 -10 19 189

54 -0.73 5.11 1.0000

15 -11 10 12 262

26 -0.73 -5.11 1.0000

-17 11 -10 139 210

150 -0.16 -0.66 1.0000

17 -11 10 1 295

-74 -0.16 0.66 1.0000

16 10 -10 87 303

81 -0.77 -0.40 1.0000

16 10 -10 451 277

439 -0.77 -0.40 1.0000

14 10 -10 190 262

256 1.53 1.17 1.0000

14 10 -10 175 258

238 1.53 1.17 1.0000

12 10 -10 349 226

149 2.81 5.10 1.0000

12 10 -10 191 241

53 2.81 5.10 1.0000

10 10 -10 168 243

241 0.91 3.15 1.0000

10 10 -10 235 195

321 0.91 3.15 1.0000

8 10 -10 471 241

677 -0.04 4.17 1.0000

6 10 -10 67 198

92 1.16 -2.73 1.0000

4 10 -10 713 197

1349 -4.18 -7.65 1.0000

4 10 -10 331 224

797 -4.18 -7.65 1.0000

2 10 -10 628 234

674 1.64 -5.70 1.0000

2 10 -10 1076 189

1150 1.64 -5.70 1.0000

0 10 -10 477 164

267 -2.51 -5.04 1.0000

0 10 -10 539 229

314 -2.51 -5.04 1.0000

-2 10 -10 1204 216

1819 4.44 -5.28 1.0000

-2 10 -10 1128 355

1726	4.44	-5.28	1.0000	
2	-10	10	1432	223
2097	4.44	5.28	1.0000	
-4	10	-10	608	170
685	3.82	2.23	1.0000	
-4	10	-10	761	262
850	3.82	2.23	1.0000	
4	-10	10	428	171
490	3.82	-2.23	1.0000	
-6	10	-10	1139	285
1186	1.67	-1.54	1.0000	
-6	10	-10	674	177
709	1.67	-1.54	1.0000	
6	-10	10	670	186
707	1.67	1.54	1.0000	
-8	10	-10	481	293
685	5.60	-2.87	1.0000	
-8	10	-10	287	177
440	5.60	-2.87	1.0000	
8	-10	10	120	186
210	5.60	2.87	1.0000	
-10	10	-10	456	267
572	0.88	-3.06	1.0000	
-10	10	-10	136	178
199	0.88	-3.06	1.0000	
10	-10	10	79	197
127	0.88	3.06	1.0000	
-12	10	-10	390	245
288	-0.98	-5.58	1.0000	
-12	10	-10	220	184
137	-0.98	-5.58	1.0000	
12	-10	10	256	228
166	-0.98	5.58	1.0000	
-14	10	-10	316	216
229	-1.89	-1.49	1.0000	
-14	10	-10	38	178
12	-1.89	-1.49	1.0000	
14	-10	10	8	231
-416	-1.89	1.49	1.0000	
-16	10	-10	8	183
-217	1.69	-1.82	1.0000	
16	-10	10	244	286
252	1.69	1.82	1.0000	
17	9	-10	188	308

```
251      1.77   -0.57  1.0000
  17    9 -10        84       304
128      1.77   -0.57  1.0000
  15    9 -10       140       295
191      3.02   -1.44  1.0000
  15    9 -10        39       272
62     3.02   -1.44  1.0000
  13    9 -10       328       221
297      0.10   -1.59  1.0000
  13    9 -10       176       267
153      0.10   -1.59  1.0000
  11    9 -10        24       195
86    -0.36    -5.59  1.0000
  11    9 -10        69       266
174    -0.36    -5.59  1.0000
   9    9 -10       850       271
708    -2.04    -2.83  1.0000
   7    9 -10       460       236
412      0.34    -1.03  1.0000
   5    9 -10       292       231
460      1.21     8.33  1.0000
   3    9 -10       128       161
365    -2.30     6.47  1.0000
   3    9 -10        12       206
123    -2.30     6.47  1.0000
   1    9 -10        62       217
447      2.79    11.36  1.0000
   1    9 -10       219       176
791      2.79    11.36  1.0000
  -1    9 -10        33       153
36    -0.06     4.18  1.0000
  -1    9 -10        23       228
-19    -0.06     4.18  1.0000
  -3    9 -10      4349       401
3731    -6.89     4.31  1.0000
  -3    9 -10      3973       264
3381    -6.89     4.31  1.0000
  -5    9 -10       995       271
827    -1.16    -2.12  1.0000
  -5    9 -10       927       179
765    -1.16    -2.12  1.0000
   5   -9  10       831       171
678    -1.16     2.12  1.0000
  -7    9 -10       170       163
```

232	-4.81	1.72	1.0000	
-7	9 -10	258		256
341	-4.81	1.72	1.0000	
7	-9 10	107		172
150	-4.81	-1.72	1.0000	
-9	9 -10	1392		211
1209	-1.98	2.85	1.0000	
9	-9 10	1244		226
1070	-1.98	-2.85	1.0000	
-11	9 -10	427		178
278	2.88	6.15	1.0000	
-11	9 -10	599		248
429	2.88	6.15	1.0000	
11	-9 10	593		220
419	2.88	-6.15	1.0000	
-13	9 -10	484		248
850	3.40	5.55	1.0000	
-13	9 -10	192		192
440	3.40	5.55	1.0000	
13	-9 10	8		240
88	3.40	-5.55	1.0000	
-15	9 -10	131		178
134	2.67	2.30	1.0000	
-15	9 -10	183		212
190	2.67	2.30	1.0000	
15	-9 10	17		258
-23	2.67	-2.30	1.0000	
-17	9 -10	170		195
153	-0.29	2.26	1.0000	
17	-9 10	90		291
76	-0.29	-2.26	1.0000	
18	8 -10	4		335
-63	-0.39	-1.69	1.0000	
18	8 -10	374		338
449	-0.39	-1.69	1.0000	
16	8 -10	147		293
178	1.22	-0.34	1.0000	
16	8 -10	200		263
236	1.22	-0.34	1.0000	
14	8 -10	265		230
273	-0.22	-0.60	1.0000	
14	8 -10	108		276
113	-0.22	-0.60	1.0000	
12	8 -10	939		203

773 1.52 2.06 1.0000
12 8 -10 483 272
366 1.52 2.06 1.0000
10 8 -10 86 245
156 5.77 2.86 1.0000
8 8 -10 1489 296
1357 2.80 -1.03 1.0000
6 8 -10 427 241
255 -1.85 -5.02 1.0000
4 8 -10 5 244
177 3.31 -9.08 1.0000
2 8 -10 2903 204
1595 -2.60 -12.30 1.0000
2 8 -10 2024 262
961 -2.60 -12.30 1.0000
0 8 -10 698 225
238 1.22 -13.89 1.0000
0 8 -10 1207 165
623 1.22 -13.89 1.0000
-2 8 -10 1965 229
2267 0.15 -2.84 1.0000
-2 8 -10 2243 367
2565 0.15 -2.84 1.0000
-4 8 -10 12 149
-2 1.12 -2.81 1.0000
-4 8 -10 36 241
81 1.12 -2.81 1.0000
4 -8 10 107 144
180 1.12 2.81 1.0000
-6 8 -10 437 155
298 4.19 2.29 1.0000
-6 8 -10 637 251
470 4.19 2.29 1.0000
6 -8 10 127 150
51 4.19 -2.29 1.0000
-8 8 -10 1920 223
1762 2.92 1.10 1.0000
8 -8 10 1829 253
1675 2.92 -1.10 1.0000
-10 8 -10 713 197
963 -0.12 -4.15 1.0000
10 -8 10 747 221
1003 -0.12 4.15 1.0000
-12 8 -10 184 176

132 -0.31 -2.12 1.0000
-12 8 -10 476 248
391 -0.31 -2.12 1.0000
12 -8 10 6 204
-123 -0.31 2.12 1.0000
-14 8 -10 241 198
291 -2.58 -5.63 1.0000
-14 8 -10 112 184
129 -2.58 -5.63 1.0000
14 -8 10 143 246
173 -2.58 5.63 1.0000
-16 8 -10 10 184
-20 -2.62 0.92 1.0000
16 -8 10 23 263
62 -2.62 -0.92 1.0000
-18 8 -10 2 197
-135 -0.16 1.07 1.0000
18 -8 10 2 289
-23 -0.16 -1.07 1.0000
19 7 -10 277 346
304 -0.21 -0.77 1.0000
19 7 -10 1 332
-208 -0.21 -0.77 1.0000
17 7 -10 331 323
440 2.14 1.37 1.0000
17 7 -10 9 279
-27 2.14 1.37 1.0000
15 7 -10 178 238
193 -0.12 1.52 1.0000
15 7 -10 405 313
429 -0.12 1.52 1.0000
13 7 -10 5 279
-154 1.69 -0.88 1.0000
13 7 -10 218 210
266 1.69 -0.88 1.0000
11 7 -10 395 286
367 -2.32 0.29 1.0000
9 7 -10 45 257
60 -4.76 -1.71 1.0000
7 7 -10 146 251
196 -2.59 6.02 1.0000
5 7 -10 122 239
148 -2.26 3.41 1.0000
3 7 -10 787 357

1926	2.38	14.01	1.0000	
1	7	-10	738	202
1635	-3.45	11.07	1.0000	
1	7	-10	334	274
994	-3.45	11.07	1.0000	
-1	7	-10	743	272
1636	-1.86	11.61	1.0000	
-1	7	-10	683	205
1546	-1.86	11.61	1.0000	
-3	7	-10	563	221
707	2.56	0.03	1.0000	
-3	7	-10	697	182
856	2.56	0.03	1.0000	
-5	7	-10	1673	219
1908	-7.41	1.06	1.0000	
-5	7	-10	2153	324
2428	-7.41	1.06	1.0000	
5	-7	10	1662	217
1900	-7.41	-1.06	1.0000	
-7	7	-10	266	158
352	-2.58	-5.93	1.0000	
-7	7	-10	210	237
281	-2.58	-5.93	1.0000	
7	-7	10	56	149
-41	-2.58	5.93	1.0000	
-9	7	-10	1148	279
1070	0.19	1.03	1.0000	
-9	7	-10	734	181
672	0.19	1.03	1.0000	
9	-7	10	700	202
639	0.19	-1.03	1.0000	
-11	7	-10	424	189
514	1.89	2.05	1.0000	
11	-7	10	481	230
576	1.89	-2.05	1.0000	
-13	7	-10	36	185
171	2.51	5.60	1.0000	
-13	7	-10	129	192
340	2.51	5.60	1.0000	
13	-7	10	50	218
-86	2.51	-5.60	1.0000	
-15	7	-10	416	215
713	4.62	2.94	1.0000	
15	-7	10	221	269

449 4.62 -2.94 1.0000
-17 7 -10 157 186
220 1.53 -1.35 1.0000
17 -7 10 119 277
175 1.53 1.35 1.0000
-19 7 -10 194 207
169 -0.70 -0.41 1.0000
19 -7 10 1 279
-25 -0.70 0.41 1.0000
18 6 -10 10 333
-428 1.36 -2.44 1.0000
18 6 -10 48 319
91 1.36 -2.44 1.0000
16 6 -10 9 315
-18 -0.44 -2.61 1.0000
16 6 -10 9 275
-162 -0.44 -2.61 1.0000
14 6 -10 12 281
-205 2.48 -1.67 1.0000
14 6 -10 244 235
304 2.48 -1.67 1.0000
12 6 -10 62 282
197 4.51 -3.17 1.0000
10 6 -10 437 278
463 1.62 1.55 1.0000
8 6 -10 623 264
450 4.36 -2.37 1.0000
6 6 -10 611 284
809 2.79 -6.65 1.0000
4 6 -10 559 282
999 -4.47 -7.98 1.0000
2 6 -10 256 249
776 -0.02 -16.51 1.0000
0 6 -10 2118 361
2360 -0.35 -7.43 1.0000
0 6 -10 2457 228
2723 -0.35 -7.43 1.0000
-2 6 -10 55 149
238 -3.36 -7.96 1.0000
-2 6 -10 42 209
16 -3.36 -7.96 1.0000
-4 6 -10 1422 320
1578 1.07 1.68 1.0000
-4 6 -10 1344 200

1496 1.07 1.68 1.0000
-6 6 -10 372 168
510 2.87 6.73 1.0000
-6 6 -10 372 259
510 2.87 6.73 1.0000
-8 6 -10 792 262
559 -1.95 4.99 1.0000
-8 6 -10 386 143
225 -1.95 4.99 1.0000
8 -6 10 336 160
186 -1.95 -4.99 1.0000
-10 6 -10 348 241
421 1.28 2.86 1.0000
-10 6 -10 26 151
39 1.28 2.86 1.0000
10 -6 10 144 183
189 1.28 -2.86 1.0000
-12 6 -10 318 173
261 -3.08 -4.04 1.0000
-12 6 -10 665 228
597 -3.08 -4.04 1.0000
12 -6 10 274 218
219 -3.08 4.04 1.0000
-14 6 -10 27 174
44 -2.31 -0.27 1.0000
14 -6 10 201 241
250 -2.31 0.27 1.0000
-16 6 -10 292 193
313 -2.58 -0.34 1.0000
16 -6 10 234 260
252 -2.58 0.34 1.0000
-18 6 -10 514 198
424 -2.03 0.27 1.0000
18 -6 10 681 311
578 -2.03 -0.27 1.0000
19 5 -10 13 349
-117 2.19 2.30 1.0000
19 5 -10 198 376
113 2.19 2.30 1.0000
17 5 -10 11 331
3 -0.15 2.59 1.0000
17 5 -10 226 310
222 -0.15 2.59 1.0000
15 5 -10 15 312

-56 1.18 3.08 1.0000
15 5 -10 15 223
-143 1.18 3.08 1.0000
13 5 -10 244 301
402 -4.60 5.39 1.0000
11 5 -10 150 263
154 -3.14 0.02 1.0000
9 5 -10 663 283
520 -1.22 2.25 1.0000
7 5 -10 2152 414
2426 -7.37 -1.03 1.0000
5 5 -10 578 275
359 0.45 10.74 1.0000
3 5 -10 907 297
1362 -0.64 7.25 1.0000
1 5 -10 329 242
40 4.53 11.83 1.0000
-1 5 -10 655 150
382 3.28 4.17 1.0000
-1 5 -10 375 225
177 3.28 4.17 1.0000
-3 5 -10 350 234
598 -3.84 3.30 1.0000
-3 5 -10 202 150
398 -3.84 3.30 1.0000
-5 5 -10 151 196
-276 0.40 -10.66 1.0000
-5 5 -10 180 141
25 0.40 -10.66 1.0000
-7 5 -10 372 162
444 -2.34 -3.40 1.0000
-7 5 -10 113 243
144 -2.34 -3.40 1.0000
7 -5 10 70 145
86 -2.34 3.40 1.0000
-9 5 -10 800 259
1116 1.23 -8.29 1.0000
-9 5 -10 485 172
721 1.23 -8.29 1.0000
9 -5 10 486 184
717 1.23 8.29 1.0000
-11 5 -10 785 248
803 2.42 0.81 1.0000
-11 5 -10 220 156

226 2.42 0.81 1.0000

11 -5 10 11 171

4 2.42 -0.81 1.0000

-13 5 -10 579 172

320 5.73 1.63 1.0000

13 -5 10 47 211

-7 5.73 -1.63 1.0000

-15 5 -10 9 164

-264 2.52 0.65 1.0000

15 -5 10 115 242

68 2.52 -0.65 1.0000

-17 5 -10 639 206

561 2.24 0.51 1.0000

17 -5 10 48 271

26 2.24 -0.51 1.0000

-19 5 -10 1 177

-224 -0.09 -0.99 1.0000

19 -5 10 1 301

-121 -0.09 0.99 1.0000

18 4 -10 200 322

379 -3.13 -3.50 1.0000

18 4 -10 29 315

-573 -3.13 -3.50 1.0000

16 4 -10 520 314

336 0.02 -4.73 1.0000

16 4 -10 672 286

462 0.02 -4.73 1.0000

14 4 -10 55 280

62 5.70 -6.55 1.0000

14 4 -10 100 198

-144 5.70 -6.55 1.0000

12 4 -10 163 277

133 2.63 -0.87 1.0000

10 4 -10 619 271

698 3.89 -2.35 1.0000

8 4 -10 191 257

288 1.09 2.83 1.0000

6 4 -10 1257 277

1405 1.11 -1.69 1.0000

4 4 -10 235 265

749 -0.17 -10.84 1.0000

2 4 -10 1541 278

1076 -5.10 -5.29 1.0000

0 4 -10 2126 406

3769	-9.00	-9.81	1.0000	
0	4	-10	2268	257
3957	-9.00	-9.81	1.0000	
-2	4	-10	62	144
36	-1.45	-0.75	1.0000	
-2	4	-10	129	235
90	-1.45	-0.75	1.0000	
-4	4	-10	363	254
972	-0.18	10.90	1.0000	
-4	4	-10	319	176
899	-0.18	10.90	1.0000	
-6	4	-10	604	190
1058	-4.47	7.96	1.0000	
-6	4	-10	588	239
1037	-4.47	7.96	1.0000	
-8	4	-10	36	132
27	3.22	9.17	1.0000	
-8	4	-10	48	214
329	3.22	9.17	1.0000	
8	-4	10	5	151
79	3.22	-9.17	1.0000	
-10	4	-10	694	201
1321	-4.21	7.61	1.0000	
-10	4	-10	1067	303
1822	-4.21	7.61	1.0000	
10	-4	10	620	222
1219	-4.21	-7.61	1.0000	
-12	4	-10	1064	208
1210	-1.88	0.74	1.0000	
12	-4	10	681	243
798	-1.88	-0.74	1.0000	
-14	4	-10	338	174
377	-4.44	-1.23	1.0000	
14	-4	10	98	235
104	-4.44	1.23	1.0000	
-16	4	-10	103	185
176	-3.31	-0.01	1.0000	
16	-4	10	15	254
-155	-3.31	0.01	1.0000	
-18	4	-10	239	184
228	-0.13	-0.29	1.0000	
18	-4	10	301	290
289	-0.13	0.29	1.0000	
19	3	-10	5	337

-314	-0.28	1.85	1.0000	
19	3	-10	5	356
-8	-0.28	1.85	1.0000	
17	3	-10	23	324
-119	2.74	3.08	1.0000	
17	3	-10	388	335
564	2.74	3.08	1.0000	
15	3	-10	161	308
491	-3.37	7.50	1.0000	
15	3	-10	420	281
898	-3.37	7.50	1.0000	
13	3	-10	415	275
396	-0.47	4.11	1.0000	
11	3	-10	195	272
271	-2.06	2.62	1.0000	
9	3	-10	3705	404
3132	-6.85	-4.31	1.0000	
7	3	-10	885	267
1064	2.58	0.04	1.0000	
5	3	-10	431	250
703	-3.87	-3.26	1.0000	
3	3	-10	1485	377
2064	3.32	9.03	1.0000	
1	3	-10	4043	391
3496	7.79	4.28	1.0000	
-1	3	-10	4249	393
3732	7.20	3.87	1.0000	
-1	3	-10	3658	238
3174	7.20	3.87	1.0000	
-3	3	-10	1040	200
1515	3.29	-9.03	1.0000	
-3	3	-10	1420	354
1980	3.29	-9.03	1.0000	
-5	3	-10	673	180
1063	-0.67	-7.23	1.0000	
-5	3	-10	714	246
1115	-0.67	-7.23	1.0000	
-7	3	-10	684	299
1751	2.33	-13.94	1.0000	
-7	3	-10	901	221
2093	2.33	-13.94	1.0000	
7	-3	10	687	225
1758	2.33	13.94	1.0000	
-9	3	-10	179	157

446	-2.29	-6.42	1.0000	
-9	3	-10	310	233
643	-2.29	-6.42	1.0000	
9	-3	10	138	172
381	-2.29	6.42	1.0000	
-11	3	-10	578	155
231	5.85	-6.25	1.0000	
11	-3	10	257	187
47	5.85	6.25	1.0000	
-13	3	-10	85	159
43	3.78	1.07	1.0000	
13	-3	10	216	217
157	3.78	-1.07	1.0000	
-15	3	-10	20	175
107	5.00	1.04	1.0000	
15	-3	10	1	246
47	5.00	-1.04	1.0000	
-17	3	-10	422	221
548	1.37	2.08	1.0000	
17	-3	10	454	281
584	1.37	-2.08	1.0000	
-19	3	-10	116	202
86	-1.32	-1.03	1.0000	
19	-3	10	4	296
-60	-1.32	1.03	1.0000	
20	2	-10	8	384
-573	-0.37	-2.47	1.0000	
20	2	-10	8	358
-505	-0.37	-2.47	1.0000	
18	2	-10	2	334
-68	-0.30	-1.11	1.0000	
18	2	-10	2	309
-462	-0.30	-1.11	1.0000	
16	2	-10	821	280
442	3.26	-5.79	1.0000	
16	2	-10	59	297
-2	3.26	-5.79	1.0000	
14	2	-10	43	266
73	-1.16	-5.41	1.0000	
14	2	-10	306	235
440	-1.16	-5.41	1.0000	
12	2	-10	1726	384
2071	1.98	-3.65	1.0000	
10	2	-10	844	285

1373	4.50	5.28	1.0000	
8	2 -10	2075	362	
2383	0.10	2.83	1.0000	
6	2 -10	31	225	
171	-3.34	8.02	1.0000	
4	2 -10	269	230	
210	-1.44	0.84	1.0000	
2	2 -10	2250	269	
1314	-7.78	-6.09	1.0000	
0	2 -10	1229	343	
1829	-7.10	-1.70	1.0000	
-2	2 -10	2255	188	
1306	-7.87	6.17	1.0000	
-2	2 -10	2137	255	
1217	-7.87	6.17	1.0000	
-4	2 -10	1965	265	
1445	-5.08	5.24	1.0000	
-4	2 -10	1752	199	
1262	-5.08	5.24	1.0000	
-6	2 -10	346	232	
983	-0.13	16.56	1.0000	
-6	2 -10	498	188	
1274	-0.13	16.56	1.0000	
-8	2 -10	2427	222	
1253	-2.58	12.26	1.0000	
-8	2 -10	2687	201	
1443	-2.58	12.26	1.0000	
8	-2 10	2327	191	
1178	-2.58	-12.26	1.0000	
-10	2 -10	792	182	
850	1.55	5.82	1.0000	
10	-2 10	669	191	
713	1.55	-5.82	1.0000	
-12	2 -10	1139	214	
1410	-4.60	4.67	1.0000	
12	-2 10	949	237	
1194	-4.60	-4.67	1.0000	
-14	2 -10	26	156	
6	-2.32	-0.54	1.0000	
14	-2 10	8	221	
-100	-2.32	0.54	1.0000	
-16	2 -10	182	178	
122	-1.68	-4.81	1.0000	
16	-2 10	154	246	

96	-1.68	4.81	1.0000	
-18	2 -10	581	219	
611	-3.05	1.04	1.0000	
18	-2 10	279	293	
296	-3.05	-1.04	1.0000	
-20	2 -10	2	199	
-153	1.12	-0.40	1.0000	
20	-2 10	160	297	
148	1.12	0.40	1.0000	
19	1 -10	383	378	
333	-0.87	0.74	1.0000	
19	1 -10	2	333	
-478	-0.87	0.74	1.0000	
17	1 -10	31	272	
-394	-2.29	4.24	1.0000	
17	1 -10	31	310	
-110	-2.29	4.24	1.0000	
15	1 -10	124	260	
79	2.20	3.13	1.0000	
15	1 -10	235	264	
178	2.20	3.13	1.0000	
13	1 -10	1491	269	
1032	0.20	5.90	1.0000	
11	1 -10	511	226	
381	-2.20	-2.94	1.0000	
9	1 -10	170	215	
212	-0.05	-4.08	1.0000	
7	1 -10	796	319	
1719	-1.85	-11.65	1.0000	
5	1 -10	643	201	
375	3.20	-4.23	1.0000	
3	1 -10	4113	375	
3600	7.14	-3.90	1.0000	
1	1 -10	490	225	
669	8.40	2.67	1.0000	
-1	1 -10	709	235	
941	8.37	-2.72	1.0000	
-3	1 -10	4090	260	
3566	7.78	-4.10	1.0000	
-3	1 -10	4746	377	
4187	7.78	-4.10	1.0000	
-5	1 -10	1133	226	
622	4.52	-11.80	1.0000	
-5	1 -10	602	149	

214	4.52	-11.80	1.0000	
-7	1	-10	447	201
1177	-3.37	-11.01	1.0000	
-7	1	-10	496	239
1257	-3.37	-11.01	1.0000	
-9	1	-10	130	174
606	2.65	-11.28	1.0000	
-9	1	-10	105	187
550	2.65	-11.28	1.0000	
9	-1	10	133	190
613	2.65	11.28	1.0000	
-11	1	-10	719	170
655	0.18	-1.21	1.0000	
11	-1	10	746	222
681	0.18	1.21	1.0000	
-13	1	-10	190	162
300	3.23	-2.53	1.0000	
13	-1	10	22	218
-48	3.23	2.53	1.0000	
-15	1	-10	168	168
261	2.16	5.38	1.0000	
15	-1	10	140	254
219	2.16	-5.38	1.0000	
-17	1	-10	106	190
137	1.08	1.11	1.0000	
17	-1	10	345	283
400	1.08	-1.11	1.0000	
-19	1	-10	130	201
140	0.67	1.23	1.0000	
19	-1	10	3	286
-335	0.67	-1.23	1.0000	
20	0	-10	22	366
42	-1.67	0.59	1.0000	
18	0	-10	32	297
-273	2.09	-4.42	1.0000	
16	0	-10	60	267
110	-2.39	-1.62	1.0000	
16	0	-10	11	256
-77	-2.39	-1.62	1.0000	
14	0	-10	781	252
1017	-0.78	-3.39	1.0000	
14	0	-10	411	264
586	-0.78	-3.39	1.0000	
12	0	-10	523	235

516	2.76	0.15	1.0000	
10	0	-10	624	215
387	-2.57	4.93	1.0000	
8	0	-10	1436	239
819	1.12	13.91	1.0000	
6	0	-10	1990	322
2223	-0.35	7.51	1.0000	
4	0	-10	2241	369
3926	-8.88	9.92	1.0000	
2	0	-10	875	249
1407	-7.20	1.80	1.0000	
0	0	-10	3994	398
5208	-7.77	0.01	1.0000	
-2	0	-10	1359	322
1982	-7.03	1.59	1.0000	
-4	0	-10	2204	272
3896	-9.12	9.91	1.0000	
-4	0	-10	2447	373
4218	-9.12	9.91	1.0000	
-6	0	-10	2738	336
3019	-0.40	7.33	1.0000	
-6	0	-10	1981	227
2212	-0.40	7.33	1.0000	
-8	0	-10	930	213
414	1.17	13.91	1.0000	
-8	0	-10	1004	161
471	1.17	13.91	1.0000	
8	0	10	1209	166
627	1.17	-13.91	1.0000	
-10	0	-10	537	175
315	-2.54	5.01	1.0000	
-10	0	-10	550	158
325	-2.54	5.01	1.0000	
10	0	10	173	170
55	-2.54	-5.01	1.0000	
-12	0	-10	302	161
292	2.73	0.21	1.0000	
12	0	10	445	215
435	2.73	-0.21	1.0000	
-14	0	-10	677	199
891	-0.62	-3.34	1.0000	
14	0	10	655	270
866	-0.62	3.34	1.0000	
-16	0	-10	270	189

365   -2.43   -1.44 1.0000
  16   0   10   160   269
233   -2.43   1.44 1.0000
 -18   0 -10   56   190
86   2.16   -4.42 1.0000
  18   0   10   32   274
-229   2.16   4.42 1.0000
 -20   0 -10   14   203
30   -1.72   0.61 1.0000
  20   0   10   183   314
236   -1.72   -0.61 1.0000
  19   -1 -10   3   319
-230   0.70   1.18 1.0000
  19   -1 -10   239   349
251   0.70   1.18 1.0000
  17   -1 -10   3   267
-93   1.01   1.11 1.0000
  17   -1 -10   297   310
348   1.01   1.11 1.0000
  15   -1 -10   226   254
340   2.19   5.41 1.0000
  15   -1 -10   45   250
-25   2.19   5.41 1.0000
  13   -1 -10   272   244
409   3.29   -2.65 1.0000
  11   -1 -10   813   238
749   0.18   -1.15 1.0000
   9   -1 -10   189   221
735   2.80   -11.38 1.0000
   7   -1 -10   650   308
1501   -3.41   -11.06 1.0000
   5   -1 -10   467   197
120   4.48   -11.85 1.0000
   3   -1 -10   3698   335
3167   7.83   -4.33 1.0000
   1   -1 -10   306   201
424   8.36   -2.83 1.0000
  -1   -1 -10   653   215
859   8.30   2.86 1.0000
  -3   -1 -10   4001   341
3487   7.22   -3.97 1.0000
  -3   -1 -10   3844   247
3339   7.22   -3.97 1.0000
  -5   -1 -10   1104   207

746 3.23 -4.10 1.0000
-5 -1 -10 462 149
242 3.23 -4.10 1.0000
-7 -1 -10 1226 318
2317 -2.00 -11.52 1.0000
-7 -1 -10 756 207
1647 -2.00 -11.52 1.0000
7 1 10 648 223
1484 -2.00 11.52 1.0000
-9 -1 -10 24 190
-14 0.03 -4.24 1.0000
-9 -1 -10 98 146
129 0.03 -4.24 1.0000
9 1 10 41 165
49 0.03 4.24 1.0000
-11 -1 -10 614 161
474 -2.17 -2.77 1.0000
11 1 10 390 194
278 -2.17 2.77 1.0000
-13 -1 -10 2046 212
1493 0.18 6.01 1.0000
13 1 10 1944 273
1405 0.18 -6.01 1.0000
-15 -1 -10 454 178
377 2.11 3.10 1.0000
15 1 10 325 261
255 2.11 -3.10 1.0000
-17 -1 -10 102 185
182 -2.30 4.37 1.0000
17 1 10 32 276
-63 -2.30 -4.37 1.0000
-19 -1 -10 1 202
-87 -0.81 0.59 1.0000
19 1 10 1 287
-359 -0.81 -0.59 1.0000
20 -2 -10 2 367
-520 1.15 -0.43 1.0000
18 -2 -10 13 269
-19 -2.97 1.04 1.0000
18 -2 -10 567 309
597 -2.97 1.04 1.0000
16 -2 -10 368 260
292 -1.76 -4.84 1.0000
16 -2 -10 1481 287

1358	-1.76	-4.84	1.0000	
14	-2	-10	262	243
183	-2.35	-0.73	1.0000	
14	-2	-10	350	230
258	-2.35	-0.73	1.0000	
12	-2	-10	1563	345
1879	-4.67	4.63	1.0000	
10	-2	-10	397	203
423	1.53	5.66	1.0000	
8	-2	-10	2371	234
1208	-2.63	12.31	1.0000	
6	-2	-10	380	222
1048	-0.09	16.49	1.0000	
4	-2	-10	1665	226
1178	-5.20	5.32	1.0000	
2	-2	-10	1830	227
997	-7.75	6.09	1.0000	
0	-2	-10	1086	289
1656	-7.12	-1.72	1.0000	
-2	-2	-10	2705	286
1657	-7.89	-6.14	1.0000	
-4	-2	-10	288	143
230	-1.41	0.71	1.0000	
-4	-2	-10	319	179
257	-1.41	0.71	1.0000	
-6	-2	-10	58	143
7	-3.27	8.03	1.0000	
-6	-2	-10	67	192
268	-3.27	8.03	1.0000	
-8	-2	-10	2254	324
2576	0.05	2.85	1.0000	
-8	-2	-10	2323	236
2650	0.05	2.85	1.0000	
8	2	10	2367	260
2697	0.05	-2.85	1.0000	
-10	-2	-10	971	232
1544	4.50	5.43	1.0000	
-10	-2	-10	1145	223
1762	4.50	5.43	1.0000	
10	2	10	1086	266
1688	4.50	-5.43	1.0000	
-12	-2	-10	1459	223
1779	1.89	-3.67	1.0000	
12	2	10	1529	277

1857	1.89	3.67	1.0000	
-14	-2	-10	118	159
187	-1.05	-5.26	1.0000	
14	2	10	79	239
130	-1.05	5.26	1.0000	
-16	-2	-10	733	190
377	3.37	-5.77	1.0000	
16	2	10	59	250
-97	3.37	5.77	1.0000	
-18	-2	-10	162	203
143	-0.40	-1.00	1.0000	
18	2	10	93	280
79	-0.40	1.00	1.0000	
-20	-2	-10	164	238
242	-0.38	-2.48	1.0000	
20	2	10	51	320
97	-0.38	2.48	1.0000	
19	-3	-10	50	335
30	-1.46	-1.05	1.0000	
19	-3	-10	40	301
22	-1.46	-1.05	1.0000	
17	-3	-10	233	273
333	1.39	2.20	1.0000	
17	-3	-10	380	308
506	1.39	2.20	1.0000	
15	-3	-10	8	221
73	5.00	0.84	1.0000	
15	-3	-10	185	250
377	5.00	0.84	1.0000	
13	-3	-10	21	212
-70	3.78	1.08	1.0000	
11	-3	-10	995	222
522	5.85	-6.25	1.0000	
9	-3	-10	55	195
232	-2.27	-6.50	1.0000	
7	-3	-10	1061	309
2339	2.30	-14.00	1.0000	
5	-3	-10	608	220
986	-0.60	-7.30	1.0000	
3	-3	-10	1036	285
1514	3.28	-9.08	1.0000	
1	-3	-10	3199	309
2727	7.14	4.00	1.0000	
-1	-3	-10	3784	316

3272	7.86		4.14	1.0000
-3	-3	-10	1649	293
2266	3.26		9.06	1.0000
-5	-3	-10	454	190
732	-3.92		-3.22	1.0000
-5	-3	-10	344	165
590	-3.92		-3.22	1.0000
-7	-3	-10	625	208
774	2.51		-0.07	1.0000
-7	-3	-10	493	177
626	2.51		-0.07	1.0000
7	3	10	711	177
869	2.51		0.07	1.0000
-9	-3	-10	3652	257
3082	-6.99		-4.33	1.0000
-9	-3	-10	3173	323
2639	-6.99		-4.33	1.0000
9	3	10	4026	292
3428	-6.99		4.33	1.0000
-11	-3	-10	411	165
520	-1.88		2.54	1.0000
11	3	10	723	218
868	-1.88		-2.54	1.0000
-13	-3	-10	616	172
601	-0.54		4.28	1.0000
13	3	10	464	238
450	-0.54		-4.28	1.0000
-15	-3	-10	358	196
811	-3.41		7.57	1.0000
15	3	10	356	287
808	-3.41		-7.57	1.0000
-17	-3	-10	384	216
562	2.71		3.16	1.0000
17	3	10	153	288
269	2.71		-3.16	1.0000
-19	-3	-10	4	213
-31	-0.20		1.83	1.0000
19	3	10	4	297
-236	-0.20		-1.83	1.0000
18	-4	-10	0	245
-281	-0.11		-0.30	1.0000
18	-4	-10	322	320
309	-0.11		-0.30	1.0000
16	-4	-10	14	244

-19 -3.28 -0.20 1.0000
16 -4 -10 31 265
69 -3.28 -0.20 1.0000
14 -4 -10 686 244
760 -4.49 -1.13 1.0000
14 -4 -10 267 238
303 -4.49 -1.13 1.0000
12 -4 -10 849 248
971 -1.93 0.50 1.0000
10 -4 -10 537 241
1103 -4.24 7.63 1.0000
8 -4 -10 42 175
21 3.24 9.06 1.0000
6 -4 -10 628 239
1092 -4.41 7.96 1.0000
4 -4 -10 376 216
987 -0.18 10.82 1.0000
2 -4 -10 186 178
139 -1.40 -0.84 1.0000
0 -4 -10 2110 330
3766 -8.98 -9.98 1.0000
-2 -4 -10 1813 225
1305 -5.13 -5.33 1.0000
-4 -4 -10 493 205
1178 -0.19 -10.86 1.0000
-6 -4 -10 1420 210
1579 1.09 -1.71 1.0000
-6 -4 -10 1659 273
1830 1.09 -1.71 1.0000
-8 -4 -10 307 162
433 1.34 2.94 1.0000
-8 -4 -10 152 185
243 1.34 2.94 1.0000
8 4 10 14 167
-9 1.34 -2.94 1.0000
-10 -4 -10 250 197
287 3.67 -2.26 1.0000
-10 -4 -10 394 172
447 3.67 -2.26 1.0000
10 4 10 649 220
721 3.67 2.26 1.0000
-12 -4 -10 573 183
514 2.74 -0.74 1.0000
12 4 10 702 252

637	2.74	0.74	1.0000	
-14	-4	-10	259	177
449	5.70	-6.56	1.0000	
14	4	10	202	250
361	5.70	6.56	1.0000	
-16	-4	-10	770	195
549	-0.04	-4.67	1.0000	
16	4	10	615	276
416	-0.04	4.67	1.0000	
-18	-4	-10	37	213
128	-3.01	-3.47	1.0000	
18	4	10	28	297
-54	-3.01	3.47	1.0000	
19	-5	-10	192	354
205	-0.13	-1.00	1.0000	
19	-5	-10	1	275
-208	-0.13	-1.00	1.0000	
17	-5	-10	199	250
158	2.13	0.44	1.0000	
17	-5	-10	847	285
764	2.13	0.44	1.0000	
15	-5	-10	553	254
442	2.55	0.73	1.0000	
15	-5	-10	987	249
838	2.55	0.73	1.0000	
13	-5	-10	722	224
430	5.67	1.48	1.0000	
11	-5	-10	279	206
286	2.46	0.78	1.0000	
9	-5	-10	327	214
512	1.22	-8.33	1.0000	
7	-5	-10	99	186
126	-2.29	-3.44	1.0000	
5	-5	-10	260	174
104	0.34	-10.72	1.0000	
3	-5	-10	383	200
639	-3.92	3.19	1.0000	
1	-5	-10	335	174
153	3.18	4.13	1.0000	
-1	-5	-10	796	176
355	4.50	11.84	1.0000	
-3	-5	-10	723	213
1134	-0.62	7.28	1.0000	
-5	-5	-10	288	153

102	0.31		10.61	1.0000
-5	-5	-10	150	157
-37	0.31		10.61	1.0000
-7	-5	-10	1459	222
1683	-7.48		-1.09	1.0000
-7	-5	-10	1545	276
1777	-7.48		-1.09	1.0000
7	5	10	1701	258
1944	-7.48		1.09	1.0000
-9	-5	-10	597	165
465	-1.12		2.24	1.0000
-9	-5	-10	654	186
516	-1.12		2.24	1.0000
9	5	10	633	197
497	-1.12		-2.24	1.0000
-11	-5	-10	176	167
181	-3.23		0.07	1.0000
11	5	10	246	202
254	-3.23		-0.07	1.0000
-13	-5	-10	309	180
491	-4.61		5.45	1.0000
13	5	10	805	262
1111	-4.61		-5.45	1.0000
-15	-5	-10	86	175
94	1.31		3.34	1.0000
15	5	10	59	262
64	1.31		-3.34	1.0000
-17	-5	-10	9	197
-16	-0.11		2.64	1.0000
17	5	10	177	294
174	-0.11		-2.64	1.0000
-19	-5	-10	13	212
-86	2.05		2.39	1.0000
19	5	10	426	321
301	2.05		-2.39	1.0000
18	-6	-10	73	316
42	-1.94		0.31	1.0000
18	-6	-10	107	267
69	-1.94		0.31	1.0000
16	-6	-10	143	238
156	-2.64		-0.34	1.0000
16	-6	-10	239	271
258	-2.64		-0.34	1.0000
14	-6	-10	220	218

273 -2.26 -0.32 1.0000

14 -6 -10 23 210

38 -2.26 -0.32 1.0000

12 -6 -10 35 176

-190 -3.03 -4.10 1.0000

12 -6 -10 35 183

-39 -3.03 -4.10 1.0000

10 -6 -10 37 191

52 1.12 2.71 1.0000

8 -6 -10 313 190

169 -1.94 4.96 1.0000

6 -6 -10 324 208

444 2.82 6.57 1.0000

4 -6 -10 1500 297

1665 1.14 1.73 1.0000

2 -6 -10 101 173

-43 -3.24 -8.07 1.0000

0 -6 -10 2184 292

2433 -0.35 -7.47 1.0000

-2 -6 -10 362 207

1000 -0.14 -16.54 1.0000

-4 -6 -10 652 213

1124 -4.36 -7.95 1.0000

-6 -6 -10 467 176

629 2.97 -6.66 1.0000

-6 -6 -10 215 166

307 2.97 -6.66 1.0000

6 6 10 418 180

561 2.97 6.66 1.0000

-8 -6 -10 359 173

232 4.10 -2.19 1.0000

-8 -6 -10 517 179

365 4.10 -2.19 1.0000

8 6 10 229 185

129 4.10 2.19 1.0000

-10 -6 -10 388 184

421 1.80 1.50 1.0000

-10 -6 -10 525 177

564 1.80 1.50 1.0000

10 6 10 542 190

580 1.80 -1.50 1.0000

-12 -6 -10 279 191

525 4.53 -3.13 1.0000

12 6 10 260 238

500 4.53 3.13 1.0000
-14 -6 -10 249 172
304 2.29 -1.54 1.0000
14 6 10 227 271
281 2.29 1.54 1.0000
-16 -6 -10 209 188
143 -0.47 -2.70 1.0000
16 6 10 344 284
258 -0.47 2.70 1.0000
-18 -6 -10 11 202
-22 1.37 -2.51 1.0000
18 6 10 11 291
-270 1.37 2.51 1.0000
19 -7 -10 1 321
-275 -0.69 -0.38 1.0000
19 -7 -10 42 276
31 -0.69 -0.38 1.0000
17 -7 -10 87 249
138 1.62 -1.41 1.0000
17 -7 -10 6 277
-60 1.62 -1.41 1.0000
15 -7 -10 241 237
469 4.52 2.80 1.0000
15 -7 -10 299 269
549 4.52 2.80 1.0000
13 -7 -10 53 210
204 2.43 5.57 1.0000
13 -7 -10 42 208
182 2.43 5.57 1.0000
11 -7 -10 295 217
369 1.86 2.04 1.0000
11 -7 -10 512 186
610 1.86 2.04 1.0000
9 -7 -10 506 211
455 0.29 1.04 1.0000
7 -7 -10 218 196
292 -2.57 -6.05 1.0000
5 -7 -10 1490 293
1709 -7.41 1.07 1.0000
3 -7 -10 544 216
682 2.50 0.00 1.0000
1 -7 -10 558 234
1352 -1.91 11.58 1.0000
-1 -7 -10 529 227

1306	-3.29	11.00	1.0000	
-3	-7	-10	639	276
1679	2.29	13.94	1.0000	
-5	-7	-10	155	157
189	-2.33	3.44	1.0000	
-5	-7	-10	82	176
100	-2.33	3.44	1.0000	
5	7	10	23	152
-123	-2.33	-3.44	1.0000	
-7	-7	-10	174	166
231	-2.55	5.94	1.0000	
-7	-7	-10	260	161
342	-2.55	5.94	1.0000	
7	7	10	191	185
255	-2.55	-5.94	1.0000	
-9	-7	-10	50	157
70	-4.80	-1.81	1.0000	
-9	-7	-10	35	164
-10	-4.80	-1.81	1.0000	
9	7	10	133	201
187	-4.80	1.81	1.0000	
-11	-7	-10	491	193
449	-2.35	0.52	1.0000	
11	7	10	336	217
300	-2.35	-0.52	1.0000	
-13	-7	-10	102	166
136	1.81	-0.87	1.0000	
13	7	10	103	248
136	1.81	0.87	1.0000	
-15	-7	-10	519	197
551	-0.07	1.59	1.0000	
15	7	10	561	295
593	-0.07	-1.59	1.0000	
-17	-7	-10	212	218
300	2.04	1.60	1.0000	
17	7	10	9	297
-47	2.04	-1.60	1.0000	
-19	-7	-10	237	241
261	-0.24	-0.75	1.0000	
19	7	10	1	305
-18	-0.24	0.75	1.0000	
18	-8	-10	207	308
200	-0.27	1.09	1.0000	
18	-8	-10	2	247

-26 -0.27 1.09 1.0000
16 -8 -10 17 255
51 -2.57 1.02 1.0000
16 -8 -10 10 226
-94 -2.57 1.02 1.0000
14 -8 -10 52 224
-103 -2.60 -5.69 1.0000
14 -8 -10 79 238
83 -2.60 -5.69 1.0000
12 -8 -10 131 204
86 -0.28 -2.23 1.0000
12 -8 -10 7 206
-20 -0.28 -2.23 1.0000
10 -8 -10 748 198
1002 -0.08 -4.14 1.0000
10 -8 -10 446 216
645 -0.08 -4.14 1.0000
8 -8 -10 1621 293
1479 2.81 1.07 1.0000
6 -8 -10 247 194
141 4.23 2.32 1.0000
4 -8 -10 14 188
-31 1.31 -2.97 1.0000
2 -8 -10 2123 316
2437 0.05 -2.85 1.0000
0 -8 -10 1001 194
466 1.11 -13.97 1.0000
-2 -8 -10 2754 278
1490 -2.53 -12.27 1.0000
-4 -8 -10 126 172
-64 3.21 -9.20 1.0000
4 8 10 4 151
87 3.21 9.20 1.0000
-6 -8 -10 251 185
124 -1.97 -4.92 1.0000
-6 -8 -10 471 167
295 -1.97 -4.92 1.0000
6 8 10 310 162
168 -1.97 4.92 1.0000
-8 -8 -10 1685 265
1534 2.99 -1.12 1.0000
-8 -8 -10 1948 235
1786 2.99 -1.12 1.0000
8 8 10 1994 297

```
1830     2.99     1.12  1.0000
 -10   -8 -10       38       163
65     5.63     2.88  1.0000
 -10   -8 -10      166       178
278     5.63     2.88  1.0000
  10    8  10      127       231
220     5.63    -2.88  1.0000
 -12   -8 -10      805       200
651     1.45     2.11  1.0000
  12    8  10      611       234
478     1.45    -2.11  1.0000
 -14   -8 -10      241       183
246    -0.27    -0.57  1.0000
  14    8  10      395       274
402    -0.27     0.57  1.0000
 -16   -8 -10        2       193
-38     1.24    -0.26  1.0000
  16    8  10        2       291
-41     1.24     0.26  1.0000
 -18   -8 -10        4       222
-137    -0.36    -1.69  1.0000
  18    8  10      115       318
158    -0.36     1.69  1.0000
  17   -9 -10      310       307
291    -0.35     2.30  1.0000
  17   -9 -10      145       254
130    -0.35     2.30  1.0000
  15   -9 -10      290       248
306     2.71     2.20  1.0000
  15   -9 -10       74       251
73     2.71     2.20  1.0000
  13   -9 -10      247       242
525     3.37     5.68  1.0000
  13   -9 -10      103       220
298     3.37     5.68  1.0000
  11   -9 -10      523       206
364     2.93     6.12  1.0000
  11   -9 -10      510       202
353     2.93     6.12  1.0000
   9   -9 -10     1548       203
1372    -2.04     2.66  1.0000
   9   -9 -10      936       239
798    -2.04     2.66  1.0000
   7   -9 -10       56       197
```

78 -4.79 1.77 1.0000
7 -9 -10 131 151
184 -4.79 1.77 1.0000
5 -9 -10 398 210
286 -1.20 -2.36 1.0000
3 -9 -10 3864 342
3274 -6.91 4.35 1.0000
1 -9 -10 206 193
255 0.06 4.17 1.0000
-1 -9 -10 42 202
386 2.71 11.32 1.0000
-3 -9 -10 150 190
400 -2.17 6.47 1.0000
-5 -9 -10 197 200
318 1.29 8.31 1.0000
5 9 10 356 176
548 1.29 -8.31 1.0000
-7 -9 -10 462 185
413 0.24 -1.03 1.0000
-7 -9 -10 662 207
603 0.24 -1.03 1.0000
7 9 10 399 189
354 0.24 1.03 1.0000
-9 -9 -10 1222 214
1062 -1.98 -2.68 1.0000
-9 -9 -10 1118 198
964 -1.98 -2.68 1.0000
9 9 10 1205 264
1047 -1.98 2.68 1.0000
-11 -9 -10 95 181
217 -0.31 -5.61 1.0000
11 9 10 20 240
73 -0.31 5.61 1.0000
-13 -9 -10 388 205
355 0.11 -1.53 1.0000
13 9 10 397 264
363 0.11 1.53 1.0000
-15 -9 -10 393 204
483 2.98 -1.33 1.0000
15 9 10 168 271
224 2.98 1.33 1.0000
-17 -9 -10 6 228
19 1.65 -0.55 1.0000
17 9 10 4 303

-154 1.65 0.55 1.0000
16 -10 -10 9 235
-52 1.78 -1.85 1.0000
16 -10 -10 9 283
-33 1.78 -1.85 1.0000
14 -10 -10 30 239
8 -1.84 -1.52 1.0000
14 -10 -10 212 227
142 -1.84 -1.52 1.0000
12 -10 -10 338 218
239 -1.02 -5.69 1.0000
12 -10 -10 190 210
109 -1.02 -5.69 1.0000
10 -10 -10 56 189
96 0.85 -3.10 1.0000
10 -10 -10 103 209
159 0.85 -3.10 1.0000
8 -10 -10 139 207
236 5.74 -2.81 1.0000
8 -10 -10 205 174
329 5.74 -2.81 1.0000
6 -10 -10 601 208
638 1.72 -1.49 1.0000
6 -10 -10 587 169
623 1.72 -1.49 1.0000
4 -10 -10 517 220
577 3.72 2.38 1.0000
2 -10 -10 1068 227
1670 4.57 -5.44 1.0000
2 -10 -10 964 302
1539 4.57 -5.44 1.0000
0 -10 -10 351 194
177 -2.49 -4.91 1.0000
0 -10 -10 598 171
366 -2.49 -4.91 1.0000
-2 -10 -10 708 212
750 1.48 -5.84 1.0000
-2 -10 -10 461 208
485 1.48 -5.84 1.0000
-4 -10 -10 700 241
1329 -4.18 -7.61 1.0000
4 10 10 759 240
1410 -4.18 7.61 1.0000
-6 -10 -10 66 162

93 1.26 -2.84 1.0000
-6 -10 -10 23 189
35 1.26 -2.84 1.0000
6 10 10 40 190
59 1.26 2.84 1.0000
-8 -10 -10 619 227
851 -0.09 4.12 1.0000
-8 -10 -10 894 225
1170 -0.09 4.12 1.0000
8 10 10 711 246
958 -0.09 -4.12 1.0000
-10 -10 -10 121 184
180 0.92 3.06 1.0000
-10 -10 -10 14 174
-83 0.92 3.06 1.0000
10 10 10 44 248
78 0.92 -3.06 1.0000
-12 -10 -10 625 196
346 2.72 5.12 1.0000
12 10 10 45 243
-191 2.72 -5.12 1.0000
-14 -10 -10 139 203
197 1.50 1.24 1.0000
14 10 10 209 275
279 1.50 -1.24 1.0000
-16 -10 -10 265 225
254 -0.72 -0.44 1.0000
16 10 10 236 276
225 -0.72 0.44 1.0000
17 -11 -10 0 262
-44 -0.19 -0.58 1.0000
17 -11 -10 326 320
342 -0.19 -0.58 1.0000
15 -11 -10 295 296
469 -0.78 5.14 1.0000
15 -11 -10 138 246
257 -0.78 5.14 1.0000
13 -11 -10 757 262
1078 4.25 3.72 1.0000
13 -11 -10 483 260
742 4.25 3.72 1.0000
11 -11 -10 306 229
490 0.94 4.47 1.0000
11 -11 -10 174 213

316   0.94   4.47 1.0000
    9 -11 -10   32   187
1   -0.39   5.59 1.0000
    9 -11 -10   38   202
116   -0.39   5.59 1.0000
    7 -11 -10   271   198
240   -2.35   -0.47 1.0000
    7 -11 -10   411   174
375   -2.35   -0.47 1.0000
    5 -11 -10   96   163
95   -3.17   -0.08 1.0000
    5 -11 -10   179   207
183   -3.17   -0.08 1.0000
    3 -11 -10   531   235
658   -1.96   -2.60 1.0000
    3 -11 -10   471   185
590   -1.96   -2.60 1.0000
    1 -11 -10   402   217
290   -2.12   2.82 1.0000
    1 -11 -10   761   181
606   -2.12   2.82 1.0000
   -1 -11 -10   760   226
698   0.24   1.18 1.0000
   -1 -11 -10   862   207
796   0.24   1.18 1.0000
   -3 -11 -10   466   208
159   5.92   6.31 1.0000
   -3 -11 -10   466   229
159   5.92   6.31 1.0000
   -5 -11 -10   9   194
-233   2.48   -0.85 1.0000
    5 11 10   228   188
235   2.48   0.85 1.0000
   -7 -11 -10   308   213
382   1.85   -2.07 1.0000
   -7 -11 -10   441   197
530   1.85   -2.07 1.0000
    7 11 10   354   222
434   1.85   2.07 1.0000
   -9 -11 -10   200   188
89   2.94   -6.09 1.0000
   -9 -11 -10   115   206
27   2.94   -6.09 1.0000
    9 11 10   238   225

121 2.94 6.09 1.0000
-11 -11 -10 160 194
298 0.93 -4.49 1.0000
-11 -11 -10 202 207
356 0.93 -4.49 1.0000
11 11 10 69 262
165 0.93 4.49 1.0000
-13 -11 -10 40 204
-103 2.70 -4.76 1.0000
13 11 10 40 262
-69 2.70 4.76 1.0000
-15 -11 -10 470 235
391 0.58 -2.11 1.0000
15 11 10 157 289
111 0.58 2.11 1.0000
-17 -11 -10 278 255
204 2.09 0.18 1.0000
17 11 10 6 300
-90 2.09 -0.18 1.0000
16 -12 -10 109 271
163 -2.22 -0.80 1.0000
16 -12 -10 176 324
243 -2.22 -0.80 1.0000
14 -12 -10 309 255
281 3.03 -2.89 1.0000
14 -12 -10 379 270
350 3.03 -2.89 1.0000
12 -12 -10 496 238
454 -0.48 -0.78 1.0000
12 -12 -10 1 218
-10 -0.48 -0.78 1.0000
10 -12 -10 175 214
45 2.79 -5.10 1.0000
10 -12 -10 45 220
-61 2.79 -5.10 1.0000
8 -12 -10 810 237
656 1.47 -2.10 1.0000
8 -12 -10 1064 214
886 1.47 -2.10 1.0000
6 -12 -10 78 212
226 4.49 3.25 1.0000
6 -12 -10 219 189
443 4.49 3.25 1.0000
4 -12 -10 427 191

376 2.77 0.79 1.0000
4 -12 -10 408 218
358 2.77 0.79 1.0000
2 -12 -10 1840 276
2202 1.94 3.71 1.0000
2 -12 -10 1685 336
2032 1.94 3.71 1.0000
0 -12 -10 546 224
535 2.70 -0.22 1.0000
0 -12 -10 452 219
441 2.70 -0.22 1.0000
-2 -12 -10 928 260
1171 -4.56 -4.68 1.0000
-2 -12 -10 865 257
1099 -4.56 -4.68 1.0000
-4 -12 -10 1293 272
1450 -1.91 -0.67 1.0000
-6 -12 -10 146 213
101 -2.95 4.10 1.0000
-6 -12 -10 234 188
185 -2.95 4.10 1.0000
6 12 10 388 216
335 -2.95 -4.10 1.0000
-8 -12 -10 6 200
-97 -0.23 2.12 1.0000
-8 -12 -10 47 192
23 -0.23 2.12 1.0000
8 12 10 6 209
-54 -0.23 -2.12 1.0000
-10 -12 -10 241 222
152 -1.01 5.68 1.0000
-10 -12 -10 101 192
35 -1.01 5.68 1.0000
10 12 10 374 238
273 -1.01 -5.68 1.0000
-12 -12 -10 117 209
97 -0.44 0.81 1.0000
12 12 10 31 270
21 -0.44 -0.81 1.0000
-14 -12 -10 192 219
342 1.94 3.99 1.0000
14 12 10 26 273
-12 1.94 -3.99 1.0000
-16 -12 -10 1 231

-444 -0.55 -0.37 1.0000
16 12 10 64 319
54 -0.55 0.37 1.0000
15 -13 -10 6 246
3 3.35 0.50 1.0000
15 -13 -10 3 294
19 3.35 0.50 1.0000
13 -13 -10 93 262
183 -1.46 3.15 1.0000
13 -13 -10 41 232
106 -1.46 3.15 1.0000
11 -13 -10 41 234
0 2.66 4.86 1.0000
11 -13 -10 41 209
-69 2.66 4.86 1.0000
9 -13 -10 221 215
195 0.08 1.59 1.0000
9 -13 -10 138 227
117 0.08 1.59 1.0000
7 -13 -10 190 223
232 1.76 0.80 1.0000
7 -13 -10 64 207
88 1.76 0.80 1.0000
5 -13 -10 412 204
624 -4.61 -5.42 1.0000
5 -13 -10 397 243
605 -4.61 -5.42 1.0000
3 -13 -10 910 210
898 -0.51 -4.20 1.0000
3 -13 -10 437 238
421 -0.51 -4.20 1.0000
1 -13 -10 2152 284
1587 0.19 -5.95 1.0000
1 -13 -10 2097 281
1540 0.19 -5.95 1.0000
-1 -13 -10 148 216
249 3.43 2.63 1.0000
-1 -13 -10 164 221
271 3.43 2.63 1.0000
-3 -13 -10 226 227
167 3.80 -1.14 1.0000
-3 -13 -10 21 260
-124 3.80 -1.14 1.0000
-5 -13 -10 274 228

107	5.67	-1.53	1.0000	
5	13	10	457	215
231	5.67	1.53	1.0000	
-7	-13	-10	0	234
45	2.53	-5.61	1.0000	
-7	-13	-10	134	202
348	2.53	-5.61	1.0000	
7	13	10	75	231
248	2.53	5.61	1.0000	
-9	-13	-10	13	205
106	3.34	-5.66	1.0000	
-9	-13	-10	76	221
249	3.34	-5.66	1.0000	
9	13	10	82	241
261	3.34	5.66	1.0000	
-11	-13	-10	944	261
1297	4.31	-3.67	1.0000	
-11	-13	-10	535	252
804	4.31	-3.67	1.0000	
11	13	10	745	279
1062	4.31	3.67	1.0000	
-13	-13	-10	428	243
604	-1.45	-3.14	1.0000	
13	13	10	172	294
289	-1.45	3.14	1.0000	
-15	-13	-10	270	259
451	3.93	-1.35	1.0000	
15	13	10	45	310
133	3.93	1.35	1.0000	
14	-14	-10	36	245
69	-1.91	1.07	1.0000	
14	-14	-10	6	280
-75	-1.91	1.07	1.0000	
12	-14	-10	5	261
40	1.98	-4.06	1.0000	
12	-14	-10	18	249
75	1.98	-4.06	1.0000	
10	-14	-10	268	247
346	1.50	-1.24	1.0000	
10	-14	-10	95	241
144	1.50	-1.24	1.0000	
8	-14	-10	402	233
410	-0.33	0.69	1.0000	
8	-14	-10	237	234

243 -0.33 0.69 1.0000
6 -14 -10 182 231
234 2.42 1.59 1.0000
6 -14 -10 11 221
-43 2.42 1.59 1.0000
4 -14 -10 103 219
192 5.67 6.59 1.0000
4 -14 -10 101 228
-102 5.67 6.59 1.0000
2 -14 -10 102 222
165 -1.07 5.28 1.0000
2 -14 -10 233 227
341 -1.07 5.28 1.0000
0 -14 -10 673 256
888 -0.67 3.34 1.0000
0 -14 -10 593 266
795 -0.67 3.34 1.0000
-2 -14 -10 251 232
173 -2.38 0.63 1.0000
-2 -14 -10 191 270
124 -2.38 0.63 1.0000
-4 -14 -10 318 247
353 -4.35 1.16 1.0000
-6 -14 -10 235 207
285 -2.23 0.22 1.0000
-6 -14 -10 7 238
-26 -2.23 0.22 1.0000
6 14 10 204 230
252 -2.23 -0.22 1.0000
-8 -14 -10 179 219
221 -2.57 5.70 1.0000
-8 -14 -10 126 255
151 -2.57 5.70 1.0000
8 14 10 52 235
-75 -2.57 -5.70 1.0000
-10 -14 -10 401 234
305 -1.80 1.49 1.0000
-10 -14 -10 354 230
264 -1.80 1.49 1.0000
10 14 10 94 250
50 -1.80 -1.49 1.0000
-12 -14 -10 418 242
379 2.90 2.82 1.0000
-12 -14 -10 22 238

-73 2.90 2.82 1.0000
12 14 10 22 260
-137 2.90 -2.82 1.0000
-14 -14 -10 119 245
174 -1.91 -1.05 1.0000
14 14 10 135 304
193 -1.91 1.05 1.0000
13 -15 -10 210 294
372 3.93 1.41 1.0000
13 -15 -10 0 248
28 3.93 1.41 1.0000
11 -15 -10 371 281
299 0.62 2.18 1.0000
11 -15 -10 179 250
129 0.62 2.18 1.0000
9 -15 -10 182 256
241 3.04 1.38 1.0000
9 -15 -10 15 243
-16 3.04 1.38 1.0000
7 -15 -10 650 248
681 -0.14 -1.57 1.0000
7 -15 -10 119 226
131 -0.14 -1.57 1.0000
5 -15 -10 23 228
18 1.23 -3.23 1.0000
5 -15 -10 180 222
201 1.23 -3.23 1.0000
3 -15 -10 518 260
1037 -3.35 -7.48 1.0000
3 -15 -10 288 255
697 -3.35 -7.48 1.0000
1 -15 -10 144 245
96 2.24 -3.12 1.0000
1 -15 -10 146 240
98 2.24 -3.12 1.0000
-1 -15 -10 232 246
342 2.14 -5.41 1.0000
-1 -15 -10 158 257
244 2.14 -5.41 1.0000
-3 -15 -10 105 251
259 5.05 -0.93 1.0000
-3 -15 -10 104 289
258 5.05 -0.93 1.0000
-5 -15 -10 388 257

294 2.54 -0.79 1.0000
-7 -15 -10 219 273
442 4.58 -2.84 1.0000
-7 -15 -10 305 236
561 4.58 -2.84 1.0000
7 15 10 10 256
87 4.58 2.84 1.0000
-9 -15 -10 17 233
-181 2.72 -2.29 1.0000
-9 -15 -10 17 261
-88 2.72 -2.29 1.0000
9 15 10 70 269
68 2.72 2.29 1.0000
-11 -15 -10 85 250
181 -0.79 -5.10 1.0000
-11 -15 -10 15 256
9 -0.79 -5.10 1.0000
11 15 10 148 270
270 -0.79 5.10 1.0000
-13 -15 -10 16 264
-331 3.44 -0.46 1.0000
13 15 10 42 286
100 3.44 0.46 1.0000
12 -16 -10 55 244
45 -0.59 0.38 1.0000
12 -16 -10 1 279
-76 -0.59 0.38 1.0000
10 -16 -10 1 252
-5 -0.77 0.42 1.0000
10 -16 -10 7 271
5 -0.77 0.42 1.0000
8 -16 -10 236 279
277 1.24 0.26 1.0000
8 -16 -10 2 222
-197 1.24 0.26 1.0000
6 -16 -10 131 253
79 -0.50 2.71 1.0000
6 -16 -10 10 232
-70 -0.50 2.71 1.0000
4 -16 -10 936 266
688 0.02 4.62 1.0000
4 -16 -10 802 274
573 0.02 4.62 1.0000
2 -16 -10 588 256

```
276      3.26     5.79  1.0000
   2 -16 -10      328       234
110      3.26     5.79  1.0000
   0 -16 -10       99       233
159     -2.39     1.51  1.0000
   0 -16 -10      169       268
246     -2.39     1.51  1.0000
  -2 -16 -10       34       286
-151     -1.62     4.76  1.0000
  -2 -16 -10       34       237
-169     -1.62     4.76  1.0000
  -4 -16 -10      193       340
285     -3.26     0.15  1.0000
  -4 -16 -10       87       258
150     -3.26     0.15  1.0000
  -6 -16 -10      181       289
196     -2.59     0.34  1.0000
  -6 -16 -10      209       232
226     -2.59     0.34  1.0000
  -8 -16 -10       10       282
-27     -2.52    -1.01  1.0000
  -8 -16 -10       12       240
42    -2.52    -1.01  1.0000
   8  16  10       62       270
119     -2.52     1.01  1.0000
 -10 -16 -10      181       260
186      1.79     1.84  1.0000
 -10 -16 -10        9       284
-251      1.79     1.84  1.0000
  10  16  10      209       289
217      1.79    -1.84  1.0000
 -12 -16 -10       90       280
140     -2.22     0.85  1.0000
 -12 -16 -10        3       277
12    -2.22     0.85  1.0000
  12  16  10      297       285
385     -2.22    -0.85  1.0000
  11 -17 -10        6       242
-46      2.09    -0.20  1.0000
  11 -17 -10      266       316
194      2.09    -0.20  1.0000
   9 -17 -10      204       260
264      1.61     0.56  1.0000
   9 -17 -10       61       298
```

95 1.61 0.56 1.0000
7 -17 -10 137 279
209 2.10 -1.45 1.0000
7 -17 -10 9 254
-50 2.10 -1.45 1.0000
5 -17 -10 422 287
424 -0.16 -2.59 1.0000
5 -17 -10 16 239
9 -0.16 -2.59 1.0000
3 -17 -10 331 260
496 2.72 -3.13 1.0000
3 -17 -10 219 279
355 2.72 -3.13 1.0000
1 -17 -10 55 249
109 -2.29 -4.31 1.0000
1 -17 -10 32 277
-44 -2.29 -4.31 1.0000
-1 -17 -10 12 293
23 1.07 -1.08 1.0000
-1 -17 -10 3 243
-38 1.07 -1.08 1.0000
-3 -17 -10 359 344
480 1.41 -2.15 1.0000
-3 -17 -10 403 271
530 1.41 -2.15 1.0000
-5 -17 -10 680 303
600 2.21 -0.51 1.0000
-5 -17 -10 340 372
283 2.21 -0.51 1.0000
-7 -17 -10 152 254
215 1.57 1.36 1.0000
-7 -17 -10 38 300
72 1.57 1.36 1.0000
7 17 10 309 283
397 1.57 -1.36 1.0000
-9 -17 -10 119 268
105 -0.33 -2.28 1.0000
-9 -17 -10 238 305
220 -0.33 -2.28 1.0000
9 17 10 85 286
72 -0.33 2.28 1.0000
-11 -17 -10 367 325
382 -0.11 0.62 1.0000
-11 -17 -10 115 279

123 -0.11 0.62 1.0000
11 17 10 3 292
4 -0.11 -0.62 1.0000
8 -18 -10 206 295
262 -0.37 1.67 1.0000
8 -18 -10 194 257
248 -0.37 1.67 1.0000
6 -18 -10 11 258
-27 1.37 2.45 1.0000
6 -18 -10 226 294
315 1.37 2.45 1.0000
4 -18 -10 29 278
-521 -3.10 3.46 1.0000
4 -18 -10 16 252
2 -3.10 3.46 1.0000
2 -18 -10 69 228
58 -0.30 1.11 1.0000
2 -18 -10 2 284
-176 -0.30 1.11 1.0000
0 -18 -10 178 315
255 2.19 4.35 1.0000
0 -18 -10 25 233
31 2.19 4.35 1.0000
-2 -18 -10 886 346
928 -3.06 -1.05 1.0000
-2 -18 -10 418 273
443 -3.06 -1.05 1.0000
-4 -18 -10 129 279
122 -0.09 0.27 1.0000
-4 -18 -10 456 358
442 -0.09 0.27 1.0000
-6 -18 -10 93 268
58 -1.90 -0.32 1.0000
-6 -18 -10 404 301
328 -1.90 -0.32 1.0000
-8 -18 -10 2 277
-21 -0.17 -1.09 1.0000
-8 -18 -10 2 320
-216 -0.17 -1.09 1.0000
8 18 10 236 270
226 -0.17 1.09 1.0000
7 -19 -10 234 305
258 -0.32 0.76 1.0000
7 -19 -10 1 255

-41 -0.32 0.76 1.0000
5 -19 -10 13 309
-131 2.11 -2.35 1.0000
5 -19 -10 95 258
40 2.11 -2.35 1.0000
3 -19 -10 32 238
15 -0.28 -1.87 1.0000
3 -19 -10 61 306
37 -0.28 -1.87 1.0000
1 -19 -10 2 314
-9 -0.87 -0.65 1.0000
1 -19 -10 2 254
-37 -0.87 -0.65 1.0000
-1 -19 -10 3 325
-86 0.73 -1.21 1.0000
-1 -19 -10 3 259
-18 0.73 -1.21 1.0000
-3 -19 -10 4 344
-234 -1.37 1.00 1.0000
-3 -19 -10 97 279
70 -1.37 1.00 1.0000
-5 -19 -10 145 385
159 -0.06 1.05 1.0000
-5 -19 -10 78 292
88 -0.06 1.05 1.0000
-7 -19 -10 393 306
358 -0.67 0.40 1.0000
-7 -19 -10 1 287
-7 -0.67 0.40 1.0000
2 -20 -10 8 316
-215 -0.36 2.43 1.0000
2 -20 -10 154 275
228 -0.36 2.43 1.0000
0 -20 -10 4 269
-58 -1.73 -0.56 1.0000
0 -20 -10 4 323
-291 -1.73 -0.56 1.0000
-2 -20 -10 452 358
432 1.19 0.45 1.0000
-2 -20 -10 2 276
-125 1.19 0.45 1.0000
3 18 -11 558 333
581 0.87 -0.23 1.0000
-3 -18 11 248 346

```
264     0.87     0.23  1.0000
   1  18 -11      466      349
593    -0.84     2.58  1.0000
  -1 -18  11       10      316
-183    -0.84    -2.58  1.0000
  -1  18 -11      979      410
994    -0.20    -0.08  1.0000
  -1  18 -11      381      233
391    -0.20    -0.08  1.0000
   1 -18  11      256      301
264    -0.20     0.08  1.0000
  -3  18 -11       10      230
-75     2.29     1.40  1.0000
  -3  18 -11      493      392
636     2.29     1.40  1.0000
   3 -18  11       42      294
91     2.29    -1.40  1.0000
   6  17 -11      364      303
331     0.24    -2.30  1.0000
  -6 -17  11        7      354
-132     0.24     2.30  1.0000
   4  17 -11      426      314
525    -0.71    -1.94  1.0000
  -4 -17  11      502      342
609    -0.71     1.94  1.0000
   2  17 -11      395      333
501    -2.44    -0.61  1.0000
  -2 -17  11      214      320
295    -2.44     0.61  1.0000
   0  17 -11      482      334
573     0.17    -1.83  1.0000
   0  17 -11      403      223
487     0.17    -1.83  1.0000
   0 -17  11      275      286
344     0.17     1.83  1.0000
  -2  17 -11      544      227
553    -0.17     0.23  1.0000
  -2  17 -11     1204      374
1218    -0.17     0.23  1.0000
   2 -17  11      343      294
351    -0.17    -0.23  1.0000
  -4  17 -11      752      385
901    -2.57    -0.42  1.0000
  -4  17 -11       82      229
```

134	-2.57	-0.42	1.0000	
4	-17	11	110	296
171	-2.57	0.42	1.0000	
-6	17	-11	956	413
992	0.15	-0.54	1.0000	
-6	17	-11	411	243
435	0.15	-0.54	1.0000	
6	-17	11	243	300
261	0.15	0.54	1.0000	
9	16	-11	507	331
557	2.22	-1.16	1.0000	
-9	-16	11	60	375
74	2.22	1.16	1.0000	
7	16	-11	359	298
395	-2.19	3.46	1.0000	
-7	-16	11	641	354
689	-2.19	-3.46	1.0000	
5	16	-11	391	299
663	1.76	4.91	1.0000	
-5	-16	11	36	297
-143	1.76	-4.91	1.0000	
3	16	-11	615	302
449	0.50	3.11	1.0000	
-3	-16	11	462	291
320	0.50	-3.11	1.0000	
1	16	-11	30	196
98	2.46	3.32	1.0000	
1	16	-11	156	321
289	2.46	3.32	1.0000	
-1	-16	11	118	258
235	2.46	-3.32	1.0000	
-1	16	-11	146	207
103	1.91	1.41	1.0000	
-1	16	-11	189	306
140	1.91	1.41	1.0000	
1	-16	11	543	255
461	1.91	-1.41	1.0000	
-3	16	-11	831	361
969	1.14	-1.66	1.0000	
-3	16	-11	155	224
218	1.14	-1.66	1.0000	
3	-16	11	151	273
213	1.14	1.66	1.0000	
-5	16	-11	60	213

```
49     2.06     1.55  1.0000
  -5  16 -11      915       384
895     2.06     1.55  1.0000
   5 -16  11       34       276
24     2.06   -1.55  1.0000
  -7  16 -11        3       222
4     1.75     0.04  1.0000
  -7  16 -11      584       388
651     1.75     0.04  1.0000
   7 -16  11      210       290
251     1.75   -0.04  1.0000
  -9  16 -11      347       384
274     0.57     2.18  1.0000
  -9  16 -11      252       223
190     0.57     2.18  1.0000
   9 -16  11      181       302
130     0.57   -2.18  1.0000
  10  15 -11      382       323
467    -3.62     0.44  1.0000
 -10 -15  11       83       377
117    -3.62   -0.44  1.0000
   8  15 -11      391       309
300     2.31   -2.87  1.0000
  -8 -15  11      561       350
455     2.31     2.87  1.0000
   6  15 -11     1097       297
739    -1.38   -5.01  1.0000
  -6 -15  11      718       309
435    -1.38     5.01  1.0000
   4  15 -11      870       307
1103    -1.02   -4.35  1.0000
  -4 -15  11      302       263
442    -1.02     4.35  1.0000
   2  15 -11      394       196
208     0.45   -4.99  1.0000
   2  15 -11      172       278
58     0.45   -4.99  1.0000
  -2 -15  11      359       236
183     0.45     4.99  1.0000
   0  15 -11      435       278
282    -4.28   -1.86  1.0000
   0  15 -11      341       194
206    -4.28   -1.86  1.0000
   0 -15  11      474       239
```

313   -4.28   1.86 1.0000
  -2 15 -11   6   202
24   -1.55   1.60 1.0000
  -2 15 -11   217   310
297   -1.55   1.60 1.0000
   2 -15 11   265   237
352   -1.55   -1.60 1.0000
  -4 15 -11   591   350
599   -0.49   1.66 1.0000
  -4 15 -11   173   212
175   -0.49   1.66 1.0000
   4 -15 11   176   240
180   -0.49   -1.66 1.0000
  -6 15 -11   226   217
334   -2.84   0.31 1.0000
  -6 15 -11   824   367
1021   -2.84   0.31 1.0000
   6 -15 11   130   260
215   -2.84   -0.31 1.0000
  -8 15 -11   244   224
244   1.56   -1.25 1.0000
  -8 15 -11   155   365
154   1.56   -1.25 1.0000
   8 -15 11   5   276
-48   1.56   1.25 1.0000
 -10 15 -11   226   234
219   2.04   -0.21 1.0000
 -10 15 -11   299   373
292   2.04   -0.21 1.0000
  10 -15 11   6   281
-255   2.04   0.21 1.0000
  11 14 -11   386   355
590   5.40   -1.65 1.0000
 -11 -14 11   324   366
515   5.40   1.65 1.0000
   9 14 -11   346   317
419   -1.04   2.66 1.0000
  -9 -14 11   11   341
-75   -1.04   -2.66 1.0000
   7 14 -11   56   278
77   3.40   3.60 1.0000
  -7 -14 11   51   274
69   3.40   -3.60 1.0000
   5 14 -11   290   276

443 0.31 3.77 1.0000
5 14 -11 167 190
286 0.31 3.77 1.0000
-5 -14 11 143 253
254 0.31 -3.77 1.0000
3 14 -11 236 284
550 1.80 9.67 1.0000
3 14 -11 255 204
581 1.80 9.67 1.0000
-3 -14 11 207 241
496 1.80 -9.67 1.0000
1 14 -11 1085 320
1330 0.23 3.05 1.0000
1 14 -11 1364 247
1637 0.23 3.05 1.0000
-1 -14 11 702 242
902 0.23 -3.05 1.0000
-1 14 -11 625 302
548 4.85 -0.42 1.0000
-1 14 -11 268 198
209 4.85 -0.42 1.0000
1 -14 11 371 198
305 4.85 0.42 1.0000
-3 14 -11 87 195
134 1.87 0.66 1.0000
-3 14 -11 533 326
642 1.87 0.66 1.0000
3 -14 11 117 219
171 1.87 -0.66 1.0000
-5 14 -11 221 200
100 3.97 -5.25 1.0000
-5 14 -11 727 338
524 3.97 -5.25 1.0000
5 -14 11 145 217
46 3.97 5.25 1.0000
-7 14 -11 708 231
710 1.77 1.86 1.0000
-7 14 -11 303 352
301 1.77 1.86 1.0000
7 -14 11 334 254
332 1.77 -1.86 1.0000
-9 14 -11 256 218
283 -0.63 0.40 1.0000
-9 14 -11 602 362

```
644    -0.63     0.40  1.0000
   9 -14  11          1       259
-231    -0.63    -0.40  1.0000
 -11  14 -11         36       342
38    -0.09    -0.13  1.0000
 -11  14 -11        100       219
104    -0.09    -0.13  1.0000
  11 -14  11         65       285
68    -0.09     0.13  1.0000
  12  13 -11        858       375
907    -1.85    -0.16  1.0000
  10  13 -11        495       329
707     2.07    -3.44  1.0000
 -10 -13  11        178       320
312     2.07     3.44  1.0000
   8  13 -11        500       217
693    -4.42    -2.99  1.0000
   8  13 -11        450       288
632    -4.42    -2.99  1.0000
  -8 -13  11        186       287
297    -4.42     2.99  1.0000
   6  13 -11        393       201
481    -0.23    -2.41  1.0000
   6  13 -11        214       264
279    -0.23    -2.41  1.0000
   4  13 -11       1080       327
1715    -0.78    -7.44  1.0000
   4  13 -11       1199       235
1864    -0.78    -7.44  1.0000
   2  13 -11        508       183
452    -0.34    -4.80  1.0000
   2  13 -11        271       257
222    -0.34    -4.80  1.0000
   0  13 -11        145       272
174     1.15     0.65  1.0000
   0  13 -11          2       182
-147     1.15     0.65  1.0000
   0 -13  11          2       181
-29     1.15    -0.65  1.0000
  -2  13 -11        561       208
513    -3.94     0.10  1.0000
  -2  13 -11        736       298
683    -3.94     0.10  1.0000
   2 -13  11        369       207
```

327 -3.94 -0.10 1.0000
-4 13 -11 315 197
302 -1.96 4.18 1.0000
-4 13 -11 824 349
822 -1.96 4.18 1.0000
4 -13 11 28 192
-39 -1.96 -4.18 1.0000
-6 13 -11 224 207
273 -0.59 4.07 1.0000
-6 13 -11 357 331
423 -0.59 4.07 1.0000
6 -13 11 141 232
170 -0.59 -4.07 1.0000
-8 13 -11 261 213
310 1.20 0.41 1.0000
-8 13 -11 515 353
584 1.20 0.41 1.0000
8 -13 11 114 246
147 1.20 -0.41 1.0000
-10 13 -11 427 336
440 1.55 0.14 1.0000
-10 13 -11 292 199
302 1.55 0.14 1.0000
10 -13 11 3 258
-192 1.55 -0.14 1.0000
-12 13 -11 150 341
298 4.72 0.50 1.0000
-12 13 -11 54 222
151 4.72 0.50 1.0000
12 -13 11 30 274
-27 4.72 -0.50 1.0000
13 12 -11 42 256
130 4.74 -0.45 1.0000
13 12 -11 126 281
264 4.74 -0.45 1.0000
11 12 -11 383 252
295 -0.41 2.13 1.0000
9 12 -11 35 266
109 4.43 2.51 1.0000
7 12 -11 182 258
126 2.23 1.30 1.0000
7 12 -11 294 205
222 2.23 1.30 1.0000
5 12 -11 682 273

313 1.49 7.21 1.0000
5 12 -11 1054 197
578 1.49 7.21 1.0000
3 12 -11 1048 194
908 -0.90 3.39 1.0000
3 12 -11 911 281
780 -0.90 3.39 1.0000
1 12 -11 231 184
370 2.56 2.45 1.0000
1 12 -11 339 271
505 2.56 2.45 1.0000
-1 12 -11 513 197
572 -1.90 1.09 1.0000
-1 12 -11 945 308
1026 -1.90 1.09 1.0000
-3 12 -11 1087 238
1673 4.13 -5.50 1.0000
-3 12 -11 1379 361
2030 4.13 -5.50 1.0000
3 -12 11 1117 268
1710 4.13 5.50 1.0000
-5 12 -11 764 333
822 1.40 -3.14 1.0000
-5 12 -11 371 196
408 1.40 -3.14 1.0000
5 -12 11 131 198
151 1.40 3.14 1.0000
-7 12 -11 565 195
546 -0.98 -5.27 1.0000
-7 12 -11 1056 364
1045 -0.98 -5.27 1.0000
7 -12 11 596 232
587 -0.98 5.27 1.0000
-9 12 -11 466 338
488 0.46 -0.05 1.0000
-9 12 -11 352 215
371 0.46 -0.05 1.0000
9 -12 11 2 238
4 0.46 0.05 1.0000
-11 12 -11 592 328
644 -0.09 1.41 1.0000
-11 12 -11 380 208
421 -0.09 1.41 1.0000
11 -12 11 342 268

381 -0.09 -1.41 1.0000
-13 12 -11 78 219
93 -1.92 0.26 1.0000
-13 12 -11 610 333
659 -1.92 0.26 1.0000
13 -12 11 228 290
256 -1.92 -0.26 1.0000
14 11 -11 136 273
140 -0.07 0.15 1.0000
14 11 -11 509 297
518 -0.07 0.15 1.0000
12 11 -11 434 266
479 -0.06 -1.42 1.0000
12 11 -11 712 240
769 -0.06 -1.42 1.0000
10 11 -11 460 258
526 -3.31 -1.90 1.0000
8 11 -11 224 221
182 0.03 -1.32 1.0000
6 11 -11 47 205
171 -2.62 -4.70 1.0000
6 11 -11 153 182
345 -2.62 -4.70 1.0000
4 11 -11 1641 242
1846 3.24 -2.09 1.0000
4 11 -11 1407 309
1597 3.24 -2.09 1.0000
2 11 -11 174 262
243 0.26 -2.23 1.0000
2 11 -11 191 188
263 0.26 -2.23 1.0000
0 11 -11 96 179
95 -0.61 -0.48 1.0000
0 11 -11 560 286
559 -0.61 -0.48 1.0000
-2 11 -11 411 184
389 2.55 5.01 1.0000
-2 11 -11 419 272
398 2.55 5.01 1.0000
-4 11 -11 723 308
893 -3.89 5.82 1.0000
-4 11 -11 471 190
599 -3.89 5.82 1.0000
-6 11 -11 330 204

687 3.05 6.31 1.0000
-6 11 -11 1008 349
1584 3.05 6.31 1.0000
6 -11 11 406 214
795 3.05 -6.31 1.0000
-8 11 -11 755 225
1028 1.36 3.98 1.0000
-8 11 -11 1333 363
1690 1.36 3.98 1.0000
8 -11 11 505 238
731 1.36 -3.98 1.0000
-10 11 -11 48 205
120 3.44 0.75 1.0000
-10 11 -11 402 329
580 3.44 0.75 1.0000
10 -11 11 183 246
308 3.44 -0.75 1.0000
-12 11 -11 1391 332
1223 -0.28 -2.14 1.0000
-12 11 -11 638 223
526 -0.28 -2.14 1.0000
12 -11 11 510 273
410 -0.28 2.14 1.0000
-14 11 -11 156 296
283 5.28 1.58 1.0000
-14 11 -11 105 212
208 5.28 1.58 1.0000
14 -11 11 179 293
312 5.28 -1.58 1.0000
15 10 -11 194 284
187 2.02 0.22 1.0000
15 10 -11 100 285
93 2.02 0.22 1.0000
13 10 -11 228 238
234 1.53 -0.21 1.0000
13 10 -11 58 262
59 1.53 -0.21 1.0000
11 10 -11 69 266
150 3.41 -0.68 1.0000
9 10 -11 252 251
200 2.94 -1.41 1.0000
7 10 -11 170 231
267 1.35 2.76 1.0000
5 10 -11 7 200

```
-107    -2.19     0.73  1.0000
    5  10 -11      240       181
182    -2.19     0.73  1.0000
    3  10 -11       10       184
40    -2.55     1.25  1.0000
    3  10 -11      134       209
220    -2.55     1.25  1.0000
    1  10 -11     1374       229
1594    -3.61     3.43  1.0000
    1  10 -11     1098       305
1293    -3.61     3.43  1.0000
   -1  10 -11     1377       237
1614    -0.30    -4.55  1.0000
   -1  10 -11     2137       394
2435    -0.30    -4.55  1.0000
   -3  10 -11      386       276
386    -0.75    -5.55  1.0000
   -3  10 -11      185       166
171    -0.75    -5.55  1.0000
   -5  10 -11      241       178
260    -1.57    -7.92  1.0000
   -5  10 -11      372       299
419    -1.57    -7.92  1.0000
    5 -10  11      160       172
149    -1.57     7.92  1.0000
   -7  10 -11      306       319
480    -1.59    -3.84  1.0000
   -7  10 -11       23       175
-52    -1.59    -3.84  1.0000
    7 -10  11        4       183
25    -1.59     3.84  1.0000
   -9  10 -11      128       198
222    -0.95    -3.86  1.0000
   -9  10 -11      286       333
425    -0.95    -3.86  1.0000
    9 -10  11      102       222
186    -0.95     3.86  1.0000
  -11  10 -11     1213       336
1329    -3.32     1.89  1.0000
  -11  10 -11      690       223
774    -3.32     1.89  1.0000
   11 -10  11      438       254
502    -3.32    -1.89  1.0000
  -13  10 -11        8       211
```

47 2.10 3.46 1.0000
-13 10 -11 91 291
191 2.10 3.46 1.0000
13 -10 11 3 279
24 2.10 -3.46 1.0000
-15 10 -11 18 213
-27 -3.61 -0.40 1.0000
-15 10 -11 434 259
524 -3.61 -0.40 1.0000
15 -10 11 109 286
148 -3.61 0.40 1.0000
16 9 -11 7 277
-83 0.62 -2.21 1.0000
16 9 -11 7 305
-3 0.62 -2.21 1.0000
14 9 -11 27 296
37 -0.65 -0.37 1.0000
14 9 -11 69 236
84 -0.65 -0.37 1.0000
12 9 -11 57 279
64 0.44 0.05 1.0000
10 9 -11 146 263
247 -0.89 3.96 1.0000
8 9 -11 521 267
532 -4.11 0.56 1.0000
6 9 -11 1514 366
1775 3.40 0.97 1.0000
4 9 -11 230 176
225 0.77 -0.98 1.0000
4 9 -11 260 224
255 0.77 -0.98 1.0000
2 9 -11 924 197
825 5.58 -0.27 1.0000
2 9 -11 439 225
359 5.58 -0.27 1.0000
0 9 -11 391 172
239 4.71 -0.18 1.0000
0 9 -11 995 267
748 4.71 -0.18 1.0000
-2 9 -11 55 167
-6 -2.63 5.88 1.0000
-2 9 -11 286 267
417 -2.63 5.88 1.0000
-4 9 -11 210 303

600 2.91 9.87 1.0000
-4 9 -11 111 172
407 2.91 9.87 1.0000
-6 9 -11 1978 382
2354 -1.68 5.28 1.0000
6 -9 11 1141 247
1429 -1.68 -5.28 1.0000
-8 9 -11 589 189
367 1.73 5.08 1.0000
-8 9 -11 1344 338
1002 1.73 5.08 1.0000
8 -9 11 663 197
430 1.73 -5.08 1.0000
-10 9 -11 442 194
374 2.94 1.46 1.0000
-10 9 -11 1438 343
1323 2.94 1.46 1.0000
10 -9 11 736 235
651 2.94 -1.46 1.0000
-12 9 -11 119 195
244 4.40 -2.44 1.0000
-12 9 -11 354 286
561 4.40 -2.44 1.0000
12 -9 11 16 256
66 4.40 2.44 1.0000
-14 9 -11 581 266
680 -1.11 -2.62 1.0000
-14 9 -11 77 216
110 -1.11 -2.62 1.0000
14 -9 11 11 258
-249 -1.11 2.62 1.0000
-16 9 -11 141 213
167 2.25 1.13 1.0000
16 -9 11 8 287
6 2.25 -1.13 1.0000
15 8 -11 50 299
46 1.59 1.26 1.0000
15 8 -11 5 233
-197 1.59 1.26 1.0000
13 8 -11 160 281
196 1.13 -0.40 1.0000
11 8 -11 304 284
483 1.34 -3.97 1.0000
9 8 -11 400 257

223 1.67 -5.12 1.0000
7 8 -11 497 255
333 -1.66 -3.11 1.0000
5 8 -11 887 258
728 -3.46 -3.11 1.0000
3 8 -11 691 239
505 -5.23 2.49 1.0000
3 8 -11 630 177
451 -5.23 2.49 1.0000
1 8 -11 290 170
76 -9.14 -3.01 1.0000
1 8 -11 520 228
237 -9.14 -3.01 1.0000
-1 8 -11 218 162
207 -3.95 -0.90 1.0000
-1 8 -11 219 210
208 -3.95 -0.90 1.0000
-3 8 -11 1221 281
785 -2.74 -5.79 1.0000
-3 8 -11 817 179
468 -2.74 -5.79 1.0000
-5 8 -11 78 150
-14 -2.06 -7.38 1.0000
-5 8 -11 835 296
453 -2.06 -7.38 1.0000
-7 8 -11 251 189
528 2.27 -6.02 1.0000
7 -8 11 250 181
526 2.27 6.02 1.0000
-9 8 -11 1644 353
1676 -4.09 -0.51 1.0000
-9 8 -11 1053 213
1074 -4.09 -0.51 1.0000
9 -8 11 790 215
805 -4.09 0.51 1.0000
-11 8 -11 465 309
401 0.01 1.37 1.0000
-11 8 -11 99 184
71 0.01 1.37 1.0000
11 -8 11 158 219
122 0.01 -1.37 1.0000
-13 8 -11 721 266
943 -4.31 3.03 1.0000
-13 8 -11 374 211

531	-4.31		3.03	1.0000	
13	-8	11	21		263
32	-4.31		-3.03	1.0000	
-15	8	-11	108		204
58	2.29		2.86	1.0000	
15	-8	11	308		282
226	2.29		-2.86	1.0000	
16	7	-11	4		306
-142	1.64		-0.06	1.0000	
16	7	-11	168		283
201	1.64		-0.06	1.0000	
14	7	-11	201		317
199	1.74		-1.88	1.0000	
12	7	-11	38		287
-16	-0.92		5.25	1.0000	
10	7	-11	49		277
128	-1.57		3.93	1.0000	
8	7	-11	223		264
489	2.29		6.08	1.0000	
6	7	-11	384		258
527	-0.95		3.65	1.0000	
4	7	-11	137		242
275	4.36		0.07	1.0000	
2	7	-11	61		237
194	4.97		-1.92	1.0000	
2	7	-11	206		172
419	4.97		-1.92	1.0000	
0	7	-11	1273		198
1044	4.74		4.13	1.0000	
0	7	-11	1087		247
873	4.74		4.13	1.0000	
-2	7	-11	272		218
314	3.31		1.26	1.0000	
-2	7	-11	236		161
275	3.31		1.26	1.0000	
-4	7	-11	34		163
116	-1.54		4.35	1.0000	
-4	7	-11	91		251
213	-1.54		4.35	1.0000	
-6	7	-11	377		182
705	-3.60		9.19	1.0000	
-6	7	-11	881		331
1396	-3.60		9.19	1.0000	
-8	7	-11	531		164

356	-1.73	3.19	1.0000	
8	-7	11	330	172
196	-1.73	-3.19	1.0000	
-10	7	-11	578	319
746	1.40	-2.71	1.0000	
-10	7	-11	188	184
288	1.40	-2.71	1.0000	
10	-7	11	12	183
-1	1.40	2.71	1.0000	
-12	7	-11	777	294
666	2.15	-1.22	1.0000	
-12	7	-11	55	170
26	2.15	-1.22	1.0000	
12	-7	11	30	227
9	2.15	1.22	1.0000	
-14	7	-11	174	238
233	3.44	-3.58	1.0000	
-14	7	-11	33	181
-187	3.44	-3.58	1.0000	
14	-7	11	191	260
255	3.44	3.58	1.0000	
-16	7	-11	22	202
-128	-2.27	-3.34	1.0000	
16	-7	11	22	269
-180	-2.27	3.34	1.0000	
17	6	-11	640	320
665	0.29	0.47	1.0000	
17	6	-11	119	339
130	0.29	0.47	1.0000	
15	6	-11	1	334
19	-2.86	-0.32	1.0000	
13	6	-11	22	288
-282	-0.63	-4.01	1.0000	
11	6	-11	4	295
100	3.02	-6.36	1.0000	
9	6	-11	1087	313
1373	-1.76	-5.25	1.0000	
7	6	-11	309	287
600	-3.59	-9.20	1.0000	
5	6	-11	558	291
612	-4.66	0.33	1.0000	
3	6	-11	1495	305
1532	-8.60	-1.18	1.0000	
1	6	-11	25	225

-189 -4.27 0.76 1.0000
1 6 -11 249 162
139 -4.27 0.76 1.0000
-1 6 -11 140 164
81 -7.84 0.60 1.0000
-1 6 -11 582 245
561 -7.84 0.60 1.0000
-3 6 -11 712 232
487 -4.20 -2.62 1.0000
-3 6 -11 493 165
307 -4.20 -2.62 1.0000
-5 6 -11 1621 287
1259 3.50 -2.80 1.0000
-5 6 -11 1409 203
1073 3.50 -2.80 1.0000
-7 6 -11 745 307
938 -0.87 -3.55 1.0000
-7 6 -11 313 168
439 -0.87 -3.55 1.0000
-9 6 -11 1387 234
1638 3.43 -0.94 1.0000
9 -6 11 1348 264
1594 3.43 0.94 1.0000
-11 6 -11 26 182
127 -2.60 4.71 1.0000
11 -6 11 3 201
61 -2.60 -4.71 1.0000
-13 6 -11 314 192
393 -0.24 2.43 1.0000
13 -6 11 246 253
316 -0.24 -2.43 1.0000
-15 6 -11 750 210
460 -1.39 5.02 1.0000
15 -6 11 766 286
472 -1.39 -5.02 1.0000
-17 6 -11 80 204
63 0.25 2.31 1.0000
17 -6 11 7 278
-462 0.25 -2.31 1.0000
16 5 -11 8 340
-279 1.88 -1.55 1.0000
14 5 -11 57 298
-357 4.00 5.18 1.0000
12 5 -11 210 312

239   1.37   3.24 1.0000
  10   5 -11   196   294
194   -1.51   7.85 1.0000
   8   5 -11   574   277
262   -2.04   7.51 1.0000
   6   5 -11   819   284
563   3.56   2.84 1.0000
   4   5 -11   1105   283
947   2.32   -0.96 1.0000
   2   5 -11   1070   287
804   4.85   -1.95 1.0000
   0   5 -11   58   239
235   1.10   -6.62 1.0000
   0   5 -11   138   164
379   1.10   -6.62 1.0000
  -2   5 -11   1591   225
1884   2.70   1.48 1.0000
  -2   5 -11   1428   294
1706   2.70   1.48 1.0000
  -4   5 -11   1670   341
2019   -6.01   2.62 1.0000
  -4   5 -11   1656   227
2003   -6.01   2.62 1.0000
  -6   5 -11   957   244
1040   -4.74   -0.34 1.0000
  -6   5 -11   858   197
935   -4.74   -0.34 1.0000
  -8   5 -11   1311   194
1123   -3.45   3.08 1.0000
   8   -5 11   1115   198
938   -3.45   -3.08 1.0000
 -10   5 -11   219   176
166   -2.29   -0.60 1.0000
 -10   5 -11   801   320
699   -2.29   -0.60 1.0000
  10   -5 11   133   199
92   -2.29   0.60 1.0000
 -12   5 -11   426   175
151   1.51   -7.13 1.0000
  12   -5 11   218   235
41   1.51   7.13 1.0000
 -14   5 -11   18   181
62   0.33   -3.67 1.0000
  14   -5 11   37   271

97 0.33 3.67 1.0000
-16 5 -11 42 200
155 1.80 -4.91 1.0000
16 -5 11 106 280
266 1.80 4.91 1.0000
17 4 -11 15 285
39 -2.50 0.46 1.0000
17 4 -11 9 333
-264 -2.50 0.46 1.0000
15 4 -11 4 318
-245 -0.43 -1.69 1.0000
13 4 -11 320 300
302 -1.99 -4.23 1.0000
11 4 -11 138 274
175 -4.00 -5.80 1.0000
9 4 -11 59 261
287 2.90 -9.92 1.0000
7 4 -11 28 244
-4 -1.56 -4.34 1.0000
5 4 -11 1615 390
1956 -5.99 -2.59 1.0000
3 4 -11 3452 451
4091 -4.70 1.88 1.0000
1 4 -11 998 309
1603 -8.18 9.21 1.0000
-1 4 -11 1553 201
1290 -1.43 3.41 1.0000
-1 4 -11 1711 297
1434 -1.43 3.41 1.0000
-3 4 -11 166 156
210 1.87 3.28 1.0000
-3 4 -11 84 232
111 1.87 3.28 1.0000
-5 4 -11 1579 257
1375 2.49 1.05 1.0000
-5 4 -11 1064 192
897 2.49 1.05 1.0000
-7 4 -11 18 163
80 4.29 0.03 1.0000
-7 4 -11 92 213
208 4.29 0.03 1.0000
-9 4 -11 413 170
407 0.79 1.03 1.0000
-9 4 -11 998 309

```
990     0.79     1.03  1.0000
   9  -4  11      219       183
213     0.79    -1.03  1.0000
 -11   4 -11     1275       223
1463     3.37     2.17  1.0000
  11  -4  11     1428       283
1627     3.37    -2.17  1.0000
 -13   4 -11     1034       244
1661    -0.84     7.48  1.0000
  13  -4  11      968       293
1577    -0.84    -7.48  1.0000
 -15   4 -11      226       204
349    -1.10     4.36  1.0000
  15  -4  11       92       281
168    -1.10    -4.36  1.0000
 -17   4 -11      426       237
525    -0.74     1.91  1.0000
  17  -4  11        0       277
6    -0.74    -1.91  1.0000
  18   3 -11        9       352
-219     2.18    -1.43  1.0000
  18   3 -11        9       346
-35     2.18    -1.43  1.0000
  16   3 -11        5       327
-516     1.16     1.63  1.0000
  14   3 -11       20       290
42     1.70    -0.63  1.0000
  12   3 -11      584       312
1025     4.16     5.40  1.0000
  10   3 -11       44       257
-199    -0.75     5.71  1.0000
   8   3 -11      776       256
439    -2.72     5.76  1.0000
   6   3 -11      382       256
215    -4.24     2.73  1.0000
   4   3 -11      171       259
215     2.00    -3.30  1.0000
   2   3 -11      509       271
810     3.01    -7.71  1.0000
   0   3 -11      828       307
1370     3.42    -8.31  1.0000
  -2   3 -11     1956       389
2487    -6.56    -8.94  1.0000
  -2   3 -11     1557       226
```

2020	-6.56	-8.94	1.0000	
-4	3	-11	3644	292
4306	-4.69	-1.96	1.0000	
-4	3	-11	3300	402
3931	-4.69	-1.96	1.0000	
-6	3	-11	1566	220
1605	-8.65	1.23	1.0000	
-6	3	-11	1476	266
1510	-8.65	1.23	1.0000	
-8	3	-11	966	192
738	-5.27	-2.44	1.0000	
-8	3	-11	1183	251
932	-5.27	-2.44	1.0000	
-10	3	-11	780	271
972	-2.54	-1.27	1.0000	
-10	3	-11	26	150
70	-2.54	-1.27	1.0000	
10	-3	11	25	186
68	-2.54	1.27	1.0000	
-12	3	-11	773	196
651	-0.97	-3.35	1.0000	
12	-3	11	554	234
448	-0.97	3.35	1.0000	
-14	3	-11	213	197
504	1.85	-9.60	1.0000	
14	-3	11	62	250
200	1.85	9.60	1.0000	
-16	3	-11	176	192
94	0.51	-3.06	1.0000	
16	-3	11	205	276
116	0.51	3.06	1.0000	
-18	3	-11	38	204
44	0.91	0.23	1.0000	
18	-3	11	430	317
450	0.91	-0.23	1.0000	
17	2	-11	295	302
305	-0.23	-0.28	1.0000	
17	2	-11	0	341
-127	-0.23	-0.28	1.0000	
15	2	-11	7	296
-155	-1.53	-1.60	1.0000	
13	2	-11	82	273
52	-3.92	-0.12	1.0000	
11	2	-11	66	265

35 2.50 -5.05 1.0000
9 2 -11 56 248
-111 -2.66 -5.91 1.0000
7 2 -11 207 239
241 3.35 -1.23 1.0000
5 2 -11 1399 298
1671 2.64 -1.54 1.0000
3 2 -11 1784 389
2291 -6.45 9.01 1.0000
1 2 -11 1052 258
578 -1.36 8.08 1.0000
-1 2 -11 1963 266
940 -1.32 13.23 1.0000
-1 2 -11 1980 181
952 -1.32 13.23 1.0000
-3 2 -11 341 176
583 3.08 7.80 1.0000
-3 2 -11 315 257
546 3.08 7.80 1.0000
-5 2 -11 1031 248
771 4.73 1.98 1.0000
-5 2 -11 954 181
704 4.73 1.98 1.0000
-7 2 -11 208 229
424 4.99 1.89 1.0000
-7 2 -11 211 166
428 4.99 1.89 1.0000
-9 2 -11 699 181
601 5.68 0.37 1.0000
-9 2 -11 1024 230
913 5.68 0.37 1.0000
9 -2 11 481 187
394 5.68 -0.37 1.0000
-11 2 -11 218 164
295 0.20 2.24 1.0000
11 -2 11 228 208
306 0.20 -2.24 1.0000
-13 2 -11 388 185
343 -0.26 4.90 1.0000
13 -2 11 134 228
93 -0.26 -4.90 1.0000
-15 2 -11 33 175
-41 0.40 5.00 1.0000
15 -2 11 33 265

-117	0.40	-5.00	1.0000	
-17	2	-11	54	216
98	-2.46	0.66	1.0000	
17	-2	11	9	278
-525	-2.46	-0.66	1.0000	
18	1	-11	317	357
324	-0.13	0.11	1.0000	
18	1	-11	0	324
-241	-0.13	0.11	1.0000	
16	1	-11	7	294
-143	1.76	-1.47	1.0000	
14	1	-11	31	257
-346	4.84	0.39	1.0000	
12	1	-11	231	275
273	-2.00	-1.08	1.0000	
10	1	-11	1642	373
1892	-0.26	4.52	1.0000	
8	1	-11	41	216
23	-3.99	0.91	1.0000	
6	1	-11	429	247
396	-7.84	-0.63	1.0000	
4	1	-11	1490	270
1242	-1.48	-3.31	1.0000	
2	1	-11	2086	262
1028	-1.35	-13.27	1.0000	
0	1	-11	411	256
762	-1.05	-13.03	1.0000	
-2	1	-11	605	149
260	-1.27	-8.16	1.0000	
-2	1	-11	722	242
340	-1.27	-8.16	1.0000	
-4	1	-11	916	214
1517	-8.26	-9.17	1.0000	
-4	1	-11	1261	372
1976	-8.26	-9.17	1.0000	
-6	1	-11	277	228
163	-4.17	-0.81	1.0000	
-6	1	-11	24	146
-31	-4.17	-0.81	1.0000	
-8	1	-11	212	149
34	-9.16	3.08	1.0000	
-8	1	-11	192	205
23	-9.16	3.08	1.0000	
-10	1	-11	1336	229

1570	-3.73	-3.36	1.0000	
10	-1	11	1306	230
1532	-3.73	3.36	1.0000	
-12	1	-11	145	163
259	2.62	-2.42	1.0000	
12	-1	11	17	213
-71	2.62	2.42	1.0000	
-14	1	-11	787	225
995	0.29	-3.00	1.0000	
14	-1	11	991	289
1222	0.29	3.00	1.0000	
-16	1	-11	115	184
229	2.42	-3.28	1.0000	
16	-1	11	167	284
301	2.42	3.28	1.0000	
-18	1	-11	190	213
278	-0.80	-2.62	1.0000	
18	-1	11	10	302
-28	-0.80	2.62	1.0000	
17	0	-11	4	294
-229	0.23	1.79	1.0000	
17	0	-11	4	310
-185	0.23	1.79	1.0000	
15	0	-11	29	251
-366	-4.32	1.83	1.0000	
13	0	-11	2	233
-219	1.12	-0.66	1.0000	
11	0	-11	293	245
293	-0.62	0.46	1.0000	
9	0	-11	633	237
438	4.62	0.25	1.0000	
7	0	-11	1253	268
1024	4.74	-4.13	1.0000	
5	0	-11	169	233
431	1.18	6.62	1.0000	
3	0	-11	713	259
1209	3.35	8.21	1.0000	
1	0	-11	608	257
1075	-0.97	13.06	1.0000	
-1	0	-11	512	254
918	-1.16	12.98	1.0000	
-3	0	-11	937	273
1508	3.41	8.29	1.0000	
-3	0	-11	1067	223

1674	3.41	8.29	1.0000	
-5	0	-11	66	163
253	1.08	6.64	1.0000	
-5	0	-11	74	237
267	1.08	6.64	1.0000	
-7	0	-11	1385	206
1151	4.73	-4.06	1.0000	
-7	0	-11	1289	257
1062	4.73	-4.06	1.0000	
-9	0	-11	1018	184
770	4.70	0.16	1.0000	
-9	0	-11	798	216
579	4.70	0.16	1.0000	
9	0	11	539	186
360	4.70	-0.16	1.0000	
-11	0	-11	384	165
375	-0.51	0.61	1.0000	
11	0	11	1	191
-73	-0.51	-0.61	1.0000	
-13	0	-11	195	171
223	1.02	-0.63	1.0000	
13	0	11	2	226
-186	1.02	0.63	1.0000	
-15	0	-11	346	189
207	-4.23	1.93	1.0000	
15	0	11	29	263
-322	-4.23	-1.93	1.0000	
-17	0	-11	377	224
461	0.11	1.88	1.0000	
17	0	11	5	290
-109	0.11	-1.88	1.0000	
18	-1	-11	11	365
37	-0.89	-2.61	1.0000	
18	-1	-11	10	282
-289	-0.89	-2.61	1.0000	
16	-1	-11	22	268
-228	2.46	-3.29	1.0000	
14	-1	-11	421	269
581	0.13	-3.10	1.0000	
12	-1	-11	48	236
119	2.53	-2.45	1.0000	
10	-1	-11	771	256
937	-3.66	-3.52	1.0000	
8	-1	-11	246	201

52   -9.17   2.92 1.0000
6   -1 -11   206   216
108   -4.22   -0.81 1.0000
4   -1 -11   1009   280
1635   -8.18   -9.19 1.0000
2   -1 -11   427   219
146   -1.28   -8.15 1.0000
0   -1 -11   467   234
852   -1.09   -13.10 1.0000
-2   -1 -11   1930   242
919   -1.34   -13.24 1.0000
-4   -1 -11   1321   252
1083   -1.41   -3.35 1.0000
-4   -1 -11   1765   216
1489   -1.41   -3.35 1.0000
-6   -1 -11   440   163
414   -7.86   -0.50 1.0000
-6   -1 -11   562   243
544   -7.86   -0.50 1.0000
-8   -1 -11   132   153
119   -4.02   0.90 1.0000
-8   -1 -11   23   205
-29   -4.02   0.90 1.0000
-10   -1 -11   1544   241
1788   -0.26   4.55 1.0000
-10   -1 -11   1901   317
2173   -0.26   4.55 1.0000
10   1 11   1376   245
1611   -0.26   -4.55 1.0000
-12   -1 -11   620   186
685   -1.87   -1.05 1.0000
12   1 11   45   222
60   -1.87   1.05 1.0000
-14   -1 -11   326   175
261   4.86   0.47 1.0000
14   1 11   32   241
-194   4.86   -0.47 1.0000
-16   -1 -11   112   184
75   2.01   -1.37 1.0000
16   1 11   8   271
-27   2.01   1.37 1.0000
-18   -1 -11   589   232
601   -0.23   0.06 1.0000
18   1 11   226   317

234	-0.23	-0.06	1.0000	
17	-2	-11	9	264
-385	-2.54	0.60	1.0000	
17	-2	-11	9	294
-256	-2.54	0.60	1.0000	
15	-2	-11	32	253
-112	0.39	4.92	1.0000	
13	-2	-11	30	241
-50	-0.32	4.76	1.0000	
11	-2	-11	6	223
-7	0.18	2.19	1.0000	
9	-2	-11	718	241
622	5.67	0.32	1.0000	
7	-2	-11	60	215
190	4.85	2.06	1.0000	
5	-2	-11	672	219
467	4.80	1.86	1.0000	
3	-2	-11	328	230
559	2.98	7.67	1.0000	
1	-2	-11	1861	228
869	-1.39	13.18	1.0000	
-1	-2	-11	914	216
477	-1.26	8.16	1.0000	
-3	-2	-11	1810	333
2307	-6.54	8.91	1.0000	
-3	-2	-11	1876	232
2384	-6.54	8.91	1.0000	
-5	-2	-11	1487	220
1775	2.72	-1.56	1.0000	
-5	-2	-11	1417	338
1699	2.72	-1.56	1.0000	
-7	-2	-11	391	171
440	3.29	-1.29	1.0000	
-7	-2	-11	84	217
98	3.29	-1.29	1.0000	
-9	-2	-11	54	208
-180	-2.51	-5.85	1.0000	
-9	-2	-11	128	160
214	-2.51	-5.85	1.0000	
9	2	11	93	183
146	-2.51	5.85	1.0000	
-11	-2	-11	252	175
241	2.58	-4.92	1.0000	
11	2	11	114	200

82 2.58 4.92 1.0000
-13 -2 -11 349 166
310 -3.90 -0.02 1.0000
13 2 11 324 234
286 -3.90 0.02 1.0000
-15 -2 -11 48 184
87 -1.55 -1.55 1.0000
15 2 11 6 258
-363 -1.55 1.55 1.0000
-17 -2 -11 157 202
165 -0.26 -0.17 1.0000
17 2 11 0 294
-9 -0.26 0.17 1.0000
18 -3 -11 1 269
-305 0.83 0.24 1.0000
18 -3 -11 124 343
135 0.83 0.24 1.0000
16 -3 -11 13 255
-42 0.48 -3.11 1.0000
16 -3 -11 39 278
7 0.48 -3.11 1.0000
14 -3 -11 60 251
188 1.79 -9.68 1.0000
12 -3 -11 717 253
595 -1.04 -3.38 1.0000
10 -3 -11 44 197
98 -2.60 -1.24 1.0000
8 -3 -11 793 241
591 -5.29 -2.65 1.0000
6 -3 -11 1497 273
1534 -8.60 1.20 1.0000
4 -3 -11 3094 363
3705 -4.62 -2.01 1.0000
2 -3 -11 1419 316
1867 -6.44 -9.02 1.0000
0 -3 -11 733 247
1242 3.43 -8.29 1.0000
-2 -3 -11 228 209
424 3.06 -7.84 1.0000
-4 -3 -11 274 203
336 1.86 -3.29 1.0000
-4 -3 -11 257 167
316 1.86 -3.29 1.0000
-6 -3 -11 417 218

```
243    -4.23     2.70  1.0000
  -6   -3 -11      725       176
492    -4.23     2.70  1.0000
  -8   -3 -11      696       173
374    -2.75     5.89  1.0000
  -8   -3 -11      439       212
192    -2.75     5.89  1.0000
 -10   -3 -11       43       209
-226    -0.83     5.61  1.0000
 -10   -3 -11       43       150
-28    -0.83     5.61  1.0000
  10    3  11       45       192
10   -0.83    -5.61  1.0000
 -12   -3 -11      894       227
1436     4.16     5.54  1.0000
  12    3  11      934       287
1486     4.16    -5.54  1.0000
 -14   -3 -11        5       169
-8     1.88    -0.60  1.0000
  14    3  11        3       263
15     1.88     0.60  1.0000
 -16   -3 -11      151       195
213     1.16     1.64  1.0000
  16    3  11        5       270
-178     1.16    -1.64  1.0000
 -18   -3 -11      235       228
339     2.38    -1.31  1.0000
  18    3  11       10       293
-314     2.38     1.31  1.0000
  17   -4 -11      172       330
235    -0.74     1.85  1.0000
  17   -4 -11       14       282
35   -0.74     1.85  1.0000
  15   -4 -11       32       246
73   -1.05     4.26  1.0000
  13   -4 -11      563       270
1045    -0.88     7.49  1.0000
  11   -4 -11     1126       266
1290     3.26     2.03  1.0000
   9   -4 -11      251       210
246     0.73     0.98  1.0000
   7   -4 -11       33       198
117     4.46    -0.17  1.0000
   5   -4 -11     1152       238
```

997	2.21	0.96	1.0000	
3	-4	-11	216	194
265	2.01	3.28	1.0000	
1	-4	-11	1448	238
1211	-1.51	3.21	1.0000	
-1	-4	-11	969	301
1573	-8.28	9.08	1.0000	
-3	-4	-11	3555	371
4205	-4.62	1.97	1.0000	
-5	-4	-11	1574	226
1912	-6.04	-2.60	1.0000	
-5	-4	-11	1686	315
2037	-6.04	-2.60	1.0000	
-7	-4	-11	88	156
206	-1.41	-4.41	1.0000	
-7	-4	-11	28	200
-154	-1.41	-4.41	1.0000	
-9	-4	-11	216	180
610	2.94	-9.82	1.0000	
-9	-4	-11	68	206
17	2.94	-9.82	1.0000	
9	4	11	63	169
292	2.94	9.82	1.0000	
-11	-4	-11	118	171
148	-3.91	-5.81	1.0000	
11	4	11	175	208
228	-3.91	5.81	1.0000	
-13	-4	-11	42	175
17	-1.90	-4.17	1.0000	
13	4	11	218	236
204	-1.90	4.17	1.0000	
-15	-4	-11	3	187
-54	-0.45	-1.49	1.0000	
15	4	11	3	277
-9	-0.45	1.49	1.0000	
-17	-4	-11	56	213
100	-2.54	0.44	1.0000	
17	4	11	9	295
-148	-2.54	-0.44	1.0000	
16	-5	-11	3	283
55	1.85	-4.81	1.0000	
16	-5	-11	4	269
63	1.85	-4.81	1.0000	
14	-5	-11	19	225

-166 0.15 -3.80 1.0000
12 -5 -11 968 242
513 1.52 -7.24 1.0000
10 -5 -11 8 199
-111 -2.30 -0.70 1.0000
8 -5 -11 872 230
714 -3.51 3.14 1.0000
6 -5 -11 374 205
414 -4.66 -0.34 1.0000
4 -5 -11 1736 317
2083 -6.02 2.53 1.0000
2 -5 -11 1560 306
1849 2.68 1.49 1.0000
0 -5 -11 143 203
391 1.16 -6.68 1.0000
-2 -5 -11 871 210
636 4.73 -1.91 1.0000
-4 -5 -11 1297 227
1123 2.39 -1.10 1.0000
-6 -5 -11 1131 194
827 3.51 2.85 1.0000
-6 -5 -11 772 216
525 3.51 2.85 1.0000
-8 -5 -11 478 164
196 -2.13 7.41 1.0000
-8 -5 -11 79 186
-326 -2.13 7.41 1.0000
8 5 11 79 180
-67 -2.13 -7.41 1.0000
-10 -5 -11 267 181
282 -1.47 7.96 1.0000
10 5 11 199 190
203 -1.47 -7.96 1.0000
-12 -5 -11 178 169
206 1.46 3.26 1.0000
12 5 11 229 237
257 1.46 -3.26 1.0000
-14 -5 -11 214 179
96 3.99 5.25 1.0000
14 5 11 426 273
264 3.99 -5.25 1.0000
-16 -5 -11 148 204
137 2.12 -1.57 1.0000
16 5 11 107 275

95	2.12	1.57	1.0000	
17	-6	-11	7	316
-68	0.18	2.25	1.0000	
17	-6	-11	7	258
-141	0.18	2.25	1.0000	
15	-6	-11	37	246
-60	-1.50	5.05	1.0000	
15	-6	-11	37	243
-72	-1.50	5.05	1.0000	
13	-6	-11	174	246
228	-0.17	2.30	1.0000	
11	-6	-11	207	229
427	-2.67	4.73	1.0000	
9	-6	-11	1404	316
1656	3.37	-0.97	1.0000	
7	-6	-11	113	203
193	-1.00	-3.70	1.0000	
5	-6	-11	1096	229
794	3.62	-2.88	1.0000	
3	-6	-11	540	207
337	-4.29	-2.79	1.0000	
1	-6	-11	329	195
294	-7.84	0.51	1.0000	
-1	-6	-11	88	187
27	-4.12	0.75	1.0000	
-3	-6	-11	1872	303
1916	-8.66	-1.29	1.0000	
-5	-6	-11	696	211
761	-4.69	0.32	1.0000	
-5	-6	-11	424	178
468	-4.69	0.32	1.0000	
-7	-6	-11	437	195
788	-3.57	-9.16	1.0000	
-7	-6	-11	266	220
532	-3.57	-9.16	1.0000	
7	6	11	357	188
673	-3.57	9.16	1.0000	
-9	-6	-11	1274	232
1574	-1.63	-5.25	1.0000	
-9	-6	-11	1051	231
1322	-1.63	-5.25	1.0000	
9	6	11	1078	250
1348	-1.63	5.25	1.0000	
-11	-6	-11	177	186

456	3.02	-6.35	1.0000	
11	6	11	264	232
591	3.02	6.35	1.0000	
-13	-6	-11	473	193
538	-0.57	-3.89	1.0000	
13	6	11	21	249
-73	-0.57	3.89	1.0000	
-15	-6	-11	163	206
259	-2.89	-0.26	1.0000	
15	6	11	11	271
-4	-2.89	0.26	1.0000	
-17	-6	-11	432	232
458	0.18	0.58	1.0000	
17	6	11	270	315
291	0.18	-0.58	1.0000	
16	-7	-11	214	262
234	-2.26	-3.54	1.0000	
16	-7	-11	23	285
-45	-2.26	-3.54	1.0000	
14	-7	-11	32	227
-59	3.43	-3.54	1.0000	
12	-7	-11	194	220
139	2.13	-1.19	1.0000	
10	-7	-11	190	212
294	1.30	-2.82	1.0000	
8	-7	-11	503	210
335	-1.69	3.17	1.0000	
6	-7	-11	273	216
545	-3.59	9.12	1.0000	
4	-7	-11	37	197
121	-1.47	4.35	1.0000	
2	-7	-11	287	198
332	3.33	1.23	1.0000	
0	-7	-11	1067	235
865	4.76	3.98	1.0000	
-2	-7	-11	127	203
300	4.87	-2.05	1.0000	
-4	-7	-11	13	195
72	4.40	0.07	1.0000	
-6	-7	-11	359	195
497	-0.87	3.60	1.0000	
-6	-7	-11	406	207
552	-0.87	3.60	1.0000	
6	7	11	261	176

379 -0.87 -3.60 1.0000
-8 -7 -11 88 203
272 2.21 6.07 1.0000
-8 -7 -11 191 188
440 2.21 6.07 1.0000
8 7 11 87 200
270 2.21 -6.07 1.0000
-10 -7 -11 129 190
245 -1.50 3.89 1.0000
10 7 11 23 206
-273 -1.50 -3.89 1.0000
-12 -7 -11 661 211
656 -0.94 5.28 1.0000
12 7 11 368 234
347 -0.94 -5.28 1.0000
-14 -7 -11 180 191
176 1.84 -1.89 1.0000
14 7 11 189 282
185 1.84 1.89 1.0000
-16 -7 -11 369 224
427 1.75 0.07 1.0000
16 7 11 89 301
116 1.75 -0.07 1.0000
15 -8 -11 395 275
303 2.34 2.94 1.0000
15 -8 -11 244 285
170 2.34 2.94 1.0000
13 -8 -11 141 249
241 -4.55 2.96 1.0000
11 -8 -11 43 193
26 0.00 1.29 1.0000
9 -8 -11 507 242
516 -3.98 -0.54 1.0000
7 -8 -11 40 214
179 2.25 -6.07 1.0000
5 -8 -11 81 194
-67 -2.12 -7.50 1.0000
3 -8 -11 957 217
574 -2.70 -5.87 1.0000
1 -8 -11 23 196
-18 -4.05 -0.94 1.0000
-1 -8 -11 123 194
-196 -9.14 -2.99 1.0000
-3 -8 -11 474 210

316   -5.30   2.55 1.0000
-5   -8 -11   1028   199
856   -3.50   -3.16 1.0000
-5   -8 -11   1321   234
1128   -3.50   -3.16 1.0000
5   8 11   1387   200
1193   -3.50   3.16 1.0000
-7   -8 -11   553   188
373   -1.77   -3.20 1.0000
-7   -8 -11   327   198
193   -1.77   -3.20 1.0000
7   8 11   313   184
182   -1.77   3.20 1.0000
-9   -8 -11   578   186
363   1.67   -5.11 1.0000
-9   -8 -11   519   211
316   1.67   -5.11 1.0000
9   8 11   446   208
256   1.67   5.11 1.0000
-11   -8 -11   645   223
892   1.39   -3.85 1.0000
11   8 11   319   260
499   1.39   3.85 1.0000
-13   -8 -11   57   193
82   1.23   -0.39 1.0000
13   8 11   370   267
430   1.23   0.39 1.0000
-15   -8 -11   162   213
160   1.65   1.31 1.0000
15   8 11   6   265
-51   1.65   -1.31 1.0000
16   -9 -11   12   261
13   2.25   1.17 1.0000
16   -9 -11   9   286
-107   2.25   1.17 1.0000
14   -9 -11   146   249
194   -1.08   -2.70 1.0000
14   -9 -11   183   273
237   -1.08   -2.70 1.0000
12   -9 -11   8   240
31   4.49   -2.43 1.0000
12   -9 -11   35   218
-65   4.49   -2.43 1.0000
10   -9 -11   399   229

334	2.85	1.45	1.0000	
8	-9	-11	480	219
284	1.59	5.15	1.0000	
6	-9	-11	1029	262
1301	-1.75	5.24	1.0000	
4	-9	-11	54	211
266	2.91	9.83	1.0000	
2	-9	-11	54	195
-156	-2.53	5.86	1.0000	
0	-9	-11	475	215
308	4.60	-0.23	1.0000	
-2	-9	-11	793	224
685	5.76	-0.48	1.0000	
-4	-9	-11	87	211
83	0.80	-1.05	1.0000	
-6	-9	-11	1347	244
1594	3.40	0.97	1.0000	
-6	-9	-11	1474	266
1732	3.40	0.97	1.0000	
6	9	11	1363	236
1613	3.40	-0.97	1.0000	
-8	-9	-11	727	210
740	-3.86	0.51	1.0000	
-8	-9	-11	481	215
488	-3.86	0.51	1.0000	
8	9	11	935	220
953	-3.86	-0.51	1.0000	
-10	-9	-11	22	198
-151	-0.92	3.93	1.0000	
-10	-9	-11	8	188
25	-0.92	3.93	1.0000	
10	9	11	34	234
84	-0.92	-3.93	1.0000	
-12	-9	-11	214	201
229	0.46	0.03	1.0000	
12	9	11	255	273
271	0.46	-0.03	1.0000	
-14	-9	-11	215	216
241	-0.70	-0.32	1.0000	
14	9	11	115	272
135	-0.70	0.32	1.0000	
-16	-9	-11	31	233
11	0.56	-2.21	1.0000	
16	9	11	36	286

```
14     0.56     2.21  1.0000
  15 -10 -11      308       284
383    -3.60    -0.46  1.0000
  15 -10 -11       13       266
11    -3.60    -0.46  1.0000
  13 -10 -11      166       254
296     1.97     3.49  1.0000
  13 -10 -11      218       272
365     1.97     3.49  1.0000
  11 -10 -11      276       228
325    -3.35     1.93  1.0000
  11 -10 -11       76       214
93    -3.35     1.93  1.0000
   9 -10 -11      265       229
402    -0.92    -3.96  1.0000
   7 -10 -11       23       192
-27    -1.51    -3.92  1.0000
   5 -10 -11      172       213
166    -1.42    -7.86  1.0000
   3 -10 -11       45       211
-7    -0.83    -5.74  1.0000
   1 -10 -11     1377       328
1605    -0.19    -4.53  1.0000
  -1 -10 -11     1326       279
1555    -3.76     3.42  1.0000
  -3 -10 -11       73       211
139    -2.56     1.25  1.0000
  -5 -10 -11        8       205
-226    -2.39     0.61  1.0000
   5  10  11       78       181
45    -2.39    -0.61  1.0000
  -7 -10 -11      190       185
292     1.38     2.77  1.0000
  -7 -10 -11      136       219
224     1.38     2.77  1.0000
   7  10  11      301       201
427     1.38    -2.77  1.0000
  -9 -10 -11      392       219
327     2.82    -1.46  1.0000
  -9 -10 -11      346       199
285     2.82    -1.46  1.0000
   9  10  11      881       244
788     2.82     1.46  1.0000
 -11 -10 -11       17       193
```

-162 3.48 -0.76 1.0000
-11 -10 -11 38 203
105 3.48 -0.76 1.0000
11 10 11 101 273
199 3.48 0.76 1.0000
-13 -10 -11 369 225
383 1.63 -0.11 1.0000
13 10 11 57 262
60 1.63 0.11 1.0000
-15 -10 -11 6 225
-51 2.08 0.24 1.0000
15 10 11 6 297
-216 2.08 -0.24 1.0000
14 -11 -11 37 251
95 5.41 1.73 1.0000
14 -11 -11 150 275
281 5.41 1.73 1.0000
12 -11 -11 627 256
514 -0.37 -2.14 1.0000
12 -11 -11 681 278
563 -0.37 -2.14 1.0000
10 -11 -11 75 207
161 3.43 0.76 1.0000
10 -11 -11 47 227
118 3.43 0.76 1.0000
8 -11 -11 476 212
692 1.33 3.90 1.0000
8 -11 -11 294 245
467 1.33 3.90 1.0000
6 -11 -11 7 225
117 2.99 6.43 1.0000
4 -11 -11 231 215
300 -4.02 5.80 1.0000
2 -11 -11 521 237
497 2.51 4.95 1.0000
0 -11 -11 41 207
38 -0.51 -0.60 1.0000
0 -11 -11 347 184
339 -0.51 -0.60 1.0000
-2 -11 -11 177 205
247 0.16 -2.23 1.0000
-2 -11 -11 34 222
67 0.16 -2.23 1.0000
-4 -11 -11 1579 345

```
1785     3.40    -2.13  1.0000
  -6 -11 -11        95       201
255    -2.57    -4.77  1.0000
  -6 -11 -11         5       230
73    -2.57    -4.77  1.0000
   6  11  11       435       214
735    -2.57     4.77  1.0000
  -8 -11 -11         2       189
-145    -0.01    -1.32  1.0000
  -8 -11 -11         2       211
-202    -0.01    -1.32  1.0000
   8  11  11         2       217
-44    -0.01     1.32  1.0000
 -10 -11 -11       546       215
619    -3.29    -1.90  1.0000
 -10 -11 -11       350       234
405    -3.29    -1.90  1.0000
  10  11  11       390       257
449    -3.29     1.90  1.0000
 -12 -11 -11       550       243
596    -0.12    -1.36  1.0000
  12  11  11       389       289
428    -0.12     1.36  1.0000
 -14 -11 -11         0       215
-10    -0.08     0.10  1.0000
  14  11  11        45       290
47    -0.08    -0.10  1.0000
  13 -12 -11       371       259
403    -1.87     0.16  1.0000
  13 -12 -11         5       270
-49    -1.87     0.16  1.0000
  11 -12 -11       288       256
323    -0.16     1.46  1.0000
  11 -12 -11       191       260
219    -0.16     1.46  1.0000
   9 -12 -11        15       214
19     0.42    -0.01  1.0000
   9 -12 -11       103       223
112     0.42    -0.01  1.0000
   7 -12 -11       345       208
323    -0.94    -5.25  1.0000
   7 -12 -11       218       218
192    -0.94    -5.25  1.0000
   5 -12 -11       200       199
```

224 1.42 -3.32 1.0000
5 -12 -11 28 209
24 1.42 -3.32 1.0000
3 -12 -11 1104 271
1692 4.20 -5.42 1.0000
3 -12 -11 962 277
1515 4.20 -5.42 1.0000
1 -12 -11 419 202
474 -1.94 1.05 1.0000
1 -12 -11 425 230
481 -1.94 1.05 1.0000
-1 -12 -11 150 222
265 2.60 2.40 1.0000
-1 -12 -11 230 230
368 2.60 2.40 1.0000
-3 -12 -11 651 240
531 -1.08 3.37 1.0000
-3 -12 -11 278 245
198 -1.08 3.37 1.0000
-5 -12 -11 482 228
183 1.56 7.17 1.0000
-7 -12 -11 71 232
39 2.06 1.16 1.0000
-7 -12 -11 58 208
29 2.06 1.16 1.0000
7 12 11 330 229
260 2.06 -1.16 1.0000
-9 -12 -11 35 222
-88 4.51 2.42 1.0000
-9 -12 -11 57 205
151 4.51 2.42 1.0000
9 12 11 13 245
56 4.51 -2.42 1.0000
-11 -12 -11 514 241
415 -0.24 2.12 1.0000
-11 -12 -11 165 224
111 -0.24 2.12 1.0000
11 12 11 859 283
731 -0.24 -2.12 1.0000
-13 -12 -11 31 242
-142 4.77 -0.47 1.0000
13 12 11 199 301
369 4.77 0.47 1.0000
12 -13 -11 31 271

109	4.75	0.42	1.0000	
12	-13	-11	30	239
-112	4.75	0.42	1.0000	
10	-13	-11	93	249
96	1.60	0.21	1.0000	
10	-13	-11	431	253
442	1.60	0.21	1.0000	
8	-13	-11	102	228
132	1.14	0.47	1.0000	
8	-13	-11	454	241
516	1.14	0.47	1.0000	
6	-13	-11	31	224
35	-0.64	3.91	1.0000	
6	-13	-11	196	217
239	-0.64	3.91	1.0000	
4	-13	-11	164	223
149	-1.94	4.27	1.0000	
4	-13	-11	435	211
429	-1.94	4.27	1.0000	
2	-13	-11	684	223
634	-3.89	0.07	1.0000	
2	-13	-11	605	245
557	-3.89	0.07	1.0000	
0	-13	-11	26	213
36	1.00	0.62	1.0000	
0	-13	-11	2	221
-183	1.00	0.62	1.0000	
-2	-13	-11	373	246
327	-0.20	-4.89	1.0000	
-2	-13	-11	77	230
40	-0.20	-4.89	1.0000	
-4	-13	-11	1010	342
1640	-0.91	-7.58	1.0000	
-4	-13	-11	895	298
1492	-0.91	-7.58	1.0000	
-6	-13	-11	74	248
110	-0.13	-2.38	1.0000	
-6	-13	-11	264	206
332	-0.13	-2.38	1.0000	
6	13	11	349	237
426	-0.13	2.38	1.0000	
-8	-13	-11	346	236
503	-4.39	-2.99	1.0000	
-8	-13	-11	98	247

176 -4.39 -2.99 1.0000
8 13 11 473 276
655 -4.39 2.99 1.0000
-10 -13 -11 22 242
-63 2.01 -3.48 1.0000
-10 -13 -11 91 229
192 2.01 -3.48 1.0000
10 13 11 214 273
360 2.01 3.48 1.0000
-12 -13 -11 93 244
108 -1.90 -0.20 1.0000
-12 -13 -11 124 249
142 -1.90 -0.20 1.0000
12 13 11 674 296
721 -1.90 0.20 1.0000
11 -14 -11 0 238
-20 -0.10 -0.08 1.0000
11 -14 -11 832 299
839 -0.10 -0.08 1.0000
9 -14 -11 49 243
63 -0.75 0.34 1.0000
9 -14 -11 226 243
255 -0.75 0.34 1.0000
7 -14 -11 68 239
63 1.77 1.94 1.0000
7 -14 -11 9 244
-235 1.77 1.94 1.0000
5 -14 -11 310 238
168 4.00 -5.19 1.0000
5 -14 -11 244 236
116 4.00 -5.19 1.0000
3 -14 -11 9 233
25 1.72 0.55 1.0000
3 -14 -11 13 242
31 1.72 0.55 1.0000
1 -14 -11 516 248
441 4.86 -0.47 1.0000
1 -14 -11 158 246
105 4.86 -0.47 1.0000
-1 -14 -11 831 301
1048 0.23 3.07 1.0000
-1 -14 -11 629 272
820 0.23 3.07 1.0000
-3 -14 -11 341 271

716 1.87 9.59 1.0000
-3 -14 -11 41 273
65 1.87 9.59 1.0000
-5 -14 -11 112 253
211 0.21 3.74 1.0000
-7 -14 -11 173 265
232 3.48 3.55 1.0000
-7 -14 -11 69 229
94 3.48 3.55 1.0000
7 14 11 184 268
244 3.48 -3.55 1.0000
-9 -14 -11 17 257
28 -1.13 2.65 1.0000
-9 -14 -11 11 239
-62 -1.13 2.65 1.0000
9 14 11 11 262
-55 -1.13 -2.65 1.0000
-11 -14 -11 94 246
191 5.35 -1.63 1.0000
-11 -14 -11 42 259
-166 5.35 -1.63 1.0000
11 14 11 261 282
430 5.35 1.63 1.0000
10 -15 -11 6 234
-121 2.02 -0.24 1.0000
10 -15 -11 18 285
12 2.02 -0.24 1.0000
8 -15 -11 262 259
264 1.64 -1.28 1.0000
8 -15 -11 6 255
-46 1.64 -1.28 1.0000
6 -15 -11 136 250
225 -2.92 0.33 1.0000
6 -15 -11 518 248
681 -2.92 0.33 1.0000
4 -15 -11 4 231
-10 -0.42 1.61 1.0000
4 -15 -11 204 250
208 -0.42 1.61 1.0000
2 -15 -11 6 250
-133 -1.51 1.59 1.0000
2 -15 -11 68 247
114 -1.51 1.59 1.0000
0 -15 -11 321 244

188 -4.27 -1.92 1.0000
0 -15 -11 29 252
-75 -4.27 -1.92 1.0000
-2 -15 -11 892 284
598 0.33 -4.96 1.0000
-2 -15 -11 349 252
175 0.33 -4.96 1.0000
-4 -15 -11 326 282
469 -1.08 -4.32 1.0000
-4 -15 -11 26 328
-51 -1.08 -4.32 1.0000
-6 -15 -11 158 214
42 -1.49 -5.10 1.0000
-6 -15 -11 516 279
277 -1.49 -5.10 1.0000
-8 -15 -11 303 256
220 2.37 -2.97 1.0000
-8 -15 -11 19 275
-48 2.37 -2.97 1.0000
8 15 11 171 271
108 2.37 2.97 1.0000
-10 -15 -11 43 270
63 -3.56 0.47 1.0000
-10 -15 -11 238 258
302 -3.56 0.47 1.0000
10 15 11 17 280
-33 -3.56 -0.47 1.0000
9 -16 -11 7 283
-21 0.59 2.28 1.0000
9 -16 -11 7 260
-227 0.59 2.28 1.0000
7 -16 -11 50 248
68 1.65 -0.03 1.0000
7 -16 -11 72 280
94 1.65 -0.03 1.0000
5 -16 -11 191 269
175 1.94 1.56 1.0000
5 -16 -11 8 267
-217 1.94 1.56 1.0000
3 -16 -11 5 268
-25 1.15 -1.67 1.0000
3 -16 -11 281 268
364 1.15 -1.67 1.0000
1 -16 -11 319 261

255 1.87 1.43 1.0000
1 -16 -11 326 269
261 1.87 1.43 1.0000
-1 -16 -11 176 315
313 2.46 3.22 1.0000
-1 -16 -11 25 258
87 2.46 3.22 1.0000
-3 -16 -11 211 313
121 0.54 3.04 1.0000
-3 -16 -11 13 276
-152 0.54 3.04 1.0000
-5 -16 -11 2 279
20 1.91 4.82 1.0000
-5 -16 -11 36 367
-106 1.91 4.82 1.0000
-7 -16 -11 23 251
-23 -2.31 3.44 1.0000
-7 -16 -11 166 307
181 -2.31 3.44 1.0000
-9 -16 -11 128 298
152 2.30 -1.15 1.0000
-9 -16 -11 9 264
-160 2.30 -1.15 1.0000
9 16 11 322 297
367 2.30 1.15 1.0000
6 -17 -11 725 309
751 0.24 -0.46 1.0000
6 -17 -11 233 269
248 0.24 -0.46 1.0000
4 -17 -11 45 292
83 -2.48 -0.43 1.0000
4 -17 -11 37 275
71 -2.48 -0.43 1.0000
2 -17 -11 221 293
232 -0.32 0.25 1.0000
2 -17 -11 239 285
251 -0.32 0.25 1.0000
0 -17 -11 161 283
215 0.19 -1.82 1.0000
0 -17 -11 665 310
771 0.19 -1.82 1.0000
-2 -17 -11 9 278
-172 -2.53 -0.68 1.0000
-2 -17 -11 9 330

-233 -2.53 -0.68 1.0000
-4 -17 -11 109 365
160 -0.76 -1.85 1.0000
-4 -17 -11 165 294
227 -0.76 -1.85 1.0000
-6 -17 -11 7 258
-58 0.21 -2.30 1.0000
-6 -17 -11 78 298
59 0.21 -2.30 1.0000
-6 -17 -11 95 393
75 0.21 -2.30 1.0000
3 -18 -11 99 274
167 2.27 1.36 1.0000
3 -18 -11 9 310
-477 2.27 1.36 1.0000
1 -18 -11 473 311
482 -0.14 -0.12 1.0000
1 -18 -11 407 290
415 -0.14 -0.12 1.0000
-1 -18 -11 10 322
-6 -0.87 2.60 1.0000
-1 -18 -11 71 294
124 -0.87 2.60 1.0000
-3 -18 -11 418 348
438 0.90 -0.23 1.0000
-3 -18 -11 165 312
177 0.90 -0.23 1.0000
4 16 -12 344 349
511 3.15 -1.70 1.0000
-4 -16 12 445 329
634 3.15 1.70 1.0000
2 16 -12 619 359
692 2.04 3.06 1.0000
2 16 -12 561 238
630 2.04 3.06 1.0000
-2 -16 12 18 290
-123 2.04 -3.06 1.0000
0 16 -12 1080 410
1091 -0.05 -0.56 1.0000
0 16 -12 424 244
431 -0.05 -0.56 1.0000
0 -16 12 96 271
99 -0.05 0.56 1.0000
-2 16 -12 497 409

676 3.50 -0.16 1.0000
-2 16 -12 281 238
418 3.50 -0.16 1.0000
2 -16 12 250 275
381 3.50 0.16 1.0000
-4 16 -12 13 242
-264 -2.40 2.09 1.0000
-4 16 -12 644 429
838 -2.40 2.09 1.0000
4 -16 12 216 289
334 -2.40 -2.09 1.0000
7 15 -12 621 358
936 -3.57 -3.48 1.0000
-7 -15 12 181 422
365 -3.57 3.48 1.0000
5 15 -12 375 227
378 -3.33 3.54 1.0000
5 15 -12 565 327
575 -3.33 3.54 1.0000
3 15 -12 1051 356
780 -3.96 0.43 1.0000
3 15 -12 936 238
681 -3.96 0.43 1.0000
1 15 -12 339 349
485 -2.67 -1.64 1.0000
1 15 -12 1 229
21 -2.67 -1.64 1.0000
-1 -15 12 378 230
531 -2.67 1.64 1.0000
-1 15 -12 209 231
333 0.37 3.28 1.0000
-1 15 -12 599 366
800 0.37 3.28 1.0000
1 -15 12 89 240
176 0.37 -3.28 1.0000
-3 15 -12 515 241
486 -0.33 -1.09 1.0000
-3 15 -12 1167 416
1123 -0.33 -1.09 1.0000
3 -15 12 347 273
323 -0.33 1.09 1.0000
-5 15 -12 216 245
326 2.92 0.00 1.0000
-5 15 -12 532 418

698 2.92 0.00 1.0000
5 -15 12 11 272
-18 2.92 0.00 1.0000
-7 15 -12 788 267
904 -1.70 2.00 1.0000
-7 15 -12 1275 446
1423 -1.70 2.00 1.0000
7 -15 12 592 310
694 -1.70 -2.00 1.0000
8 14 -12 351 341
452 0.72 3.55 1.0000
8 14 -12 17 227
-246 0.72 3.55 1.0000
6 14 -12 280 325
408 4.00 -1.79 1.0000
6 14 -12 264 223
388 4.00 -1.79 1.0000
4 14 -12 903 354
1308 5.25 1.00 1.0000
4 14 -12 525 239
843 5.25 1.00 1.0000
2 14 -12 150 220
256 3.98 0.75 1.0000
2 14 -12 434 331
610 3.98 0.75 1.0000
0 14 -12 343 339
289 4.02 -0.12 1.0000
0 14 -12 257 225
208 4.02 -0.12 1.0000
-2 14 -12 1111 378
921 -2.75 -0.04 1.0000
-2 14 -12 480 223
358 -2.75 -0.04 1.0000
2 -14 12 10 211
-52 -2.75 0.04 1.0000
-4 14 -12 634 231
635 -0.01 0.44 1.0000
-4 14 -12 1147 411
1149 -0.01 0.44 1.0000
4 -14 12 239 257
239 -0.01 -0.44 1.0000
-6 14 -12 1043 411
894 -2.12 -0.62 1.0000
-6 14 -12 261 235

189   -2.12   -0.62 1.0000
   6 -14 12   33   251
11   -2.12   0.62 1.0000
  -8 14 -12   435   412
533   1.82   -0.66 1.0000
  -8 14 -12   151   234
210   1.82   -0.66 1.0000
   8 -14 12   159   291
220   1.82   0.66 1.0000
   9 13 -12   751   255
894   -2.40   -3.19 1.0000
   9 13 -12   905   381
1062   -2.40   -3.19 1.0000
   7 13 -12   256   241
326   -0.96   3.94 1.0000
   7 13 -12   406   335
497   -0.96   3.94 1.0000
   5 13 -12   178   317
374   -5.38   -1.23 1.0000
   5 13 -12   67   213
199   -5.38   -1.23 1.0000
   3 13 -12   384   313
593   -4.19   0.98 1.0000
   3 13 -12   83   223
190   -4.19   0.98 1.0000
   1 13 -12   1025   340
1368   -2.03   3.83 1.0000
   1 13 -12   608   231
878   -2.03   3.83 1.0000
  -1 13 -12   975   354
900   -1.43   -0.58 1.0000
  -1 13 -12   3   203
-186   -1.43   -0.58 1.0000
  -3 13 -12   302   219
354   4.45   1.28 1.0000
  -3 13 -12   746   365
841   4.45   1.28 1.0000
  -5 13 -12   618   385
732   1.92   -0.11 1.0000
  -5 13 -12   266   221
343   1.92   -0.11 1.0000
   5 -13 12   88   222
134   1.92   0.11 1.0000
  -7 13 -12   546   396

609 3.41 0.17 1.0000
-7 13 -12 15 222
-82 3.41 0.17 1.0000
7 -13 12 60 253
71 3.41 -0.17 1.0000
-9 13 -12 135 239
147 -0.22 2.28 1.0000
-9 13 -12 1007 407
1052 -0.22 2.28 1.0000
9 -13 12 7 277
-122 -0.22 -2.28 1.0000
10 12 -12 758 339
849 3.23 2.40 1.0000
8 12 -12 606 244
562 1.50 -3.60 1.0000
8 12 -12 879 344
830 1.50 -3.60 1.0000
6 12 -12 439 295
278 3.43 1.42 1.0000
6 12 -12 288 229
161 3.43 1.42 1.0000
4 12 -12 370 321
587 3.87 2.36 1.0000
4 12 -12 422 228
652 3.87 2.36 1.0000
2 12 -12 226 200
106 4.22 -4.23 1.0000
2 12 -12 207 304
92 4.22 -4.23 1.0000
0 12 -12 480 334
496 0.37 0.77 1.0000
0 12 -12 1 213
-45 0.37 0.77 1.0000
-2 12 -12 1104 353
927 -1.75 1.70 1.0000
-2 12 -12 394 211
291 -1.75 1.70 1.0000
-4 12 -12 347 220
518 -4.32 -2.34 1.0000
-4 12 -12 660 368
894 -4.32 -2.34 1.0000
-6 12 -12 23 204
-110 -3.29 2.53 1.0000
-6 12 -12 132 378

234	-3.29	2.53	1.0000	
6	-12	12	23	206
-35	-3.29	-2.53	1.0000	
-8	12	-12	224	216
104	-4.61	0.23	1.0000	
-8	12	-12	1019	403
745	-4.61	0.23	1.0000	
8	-12	12	28	248
-31	-4.61	-0.23	1.0000	
-10	12	-12	70	231
109	2.66	-2.18	1.0000	
10	-12	12	15	276
28	2.66	2.18	1.0000	
11	11	-12	924	286
853	-0.78	-2.71	1.0000	
9	11	-12	119	240
251	-0.88	4.18	1.0000	
7	11	-12	288	227
413	-2.97	0.02	1.0000	
7	11	-12	535	262
701	-2.97	0.02	1.0000	
5	11	-12	11	212
-131	-2.91	-0.01	1.0000	
5	11	-12	11	257
-198	-2.91	-0.01	1.0000	
3	11	-12	214	289
158	-3.05	2.67	1.0000	
3	11	-12	249	199
190	-3.05	2.67	1.0000	
1	11	-12	350	323
513	-2.32	2.55	1.0000	
1	11	-12	167	202
284	-2.32	2.55	1.0000	
-1	11	-12	281	327
470	4.31	0.18	1.0000	
-1	11	-12	146	206
288	4.31	0.18	1.0000	
-3	11	-12	503	327
445	2.81	1.31	1.0000	
-3	11	-12	378	210
327	2.81	1.31	1.0000	
-5	11	-12	830	215
534	6.17	2.40	1.0000	
-5	11	-12	826	355

531   6.17   2.40 1.0000
  -7 11 -12   650   401
1047   5.19   -3.89 1.0000
  -7 11 -12   53   208
191   5.19   -3.89 1.0000
   7 -11 12   82   202
247   5.19   3.89 1.0000
  -9 11 -12   1410   256
1626   2.42   -0.30 1.0000
   9 -11 12   847   276
1016   2.42   0.30 1.0000
 -11 11 -12   450   239
404   -0.70   2.61 1.0000
  11 -11 12   470   285
421   -0.70   -2.61 1.0000
  12 10 -12   400   294
508   2.68   2.22 1.0000
  10 10 -12   330   268
182   -3.78   -2.20 1.0000
   8 10 -12   151   253
166   1.96   -1.70 1.0000
   6 10 -12   330   240
281   0.66   1.26 1.0000
   6 10 -12   3   204
-137   0.66   1.26 1.0000
   4 10 -12   590   256
671   1.94   -3.76 1.0000
   4 10 -12   792   229
889   1.94   -3.76 1.0000
   2 10 -12   17   188
-3   2.05   2.94 1.0000
   2 10 -12   192   285
288   2.05   2.94 1.0000
   0 10 -12   319   306
242   -2.28   -0.96 1.0000
   0 10 -12   357   195
275   -2.28   -0.96 1.0000
  -2 10 -12   207   318
337   -4.51   -1.70 1.0000
  -2 10 -12   120   192
219   -4.51   -1.70 1.0000
  -4 10 -12   1127   249
1810   -7.65   1.68 1.0000
  -4 10 -12   1591   406

```
2388    -7.65     1.68  1.0000
  -6   10 -12      244      216
588    -8.30    -0.29  1.0000
  -6   10 -12      561      381
1052    -8.30    -0.29  1.0000
  -8   10 -12        8      193
-156    -1.43     2.03  1.0000
  -8   10 -12      594      383
466    -1.43     2.03  1.0000
   8 -10  12        8      187
-178    -1.43    -2.03  1.0000
 -10   10 -12      975      398
713    -3.73     2.29  1.0000
 -10   10 -12      658      209
445    -3.73     2.29  1.0000
  10 -10  12      122      252
40    -3.73    -2.29  1.0000
 -12   10 -12       54      222
62      3.23    -2.31  1.0000
 -12   10 -12      582      364
654      3.23    -2.31  1.0000
  12 -10  12       19      278
9      3.23     2.31  1.0000
  13    9 -12       35      297
37    -0.25    -2.33  1.0000
  11    9 -12      962      322
1135     2.34     0.31  1.0000
   9    9 -12       60      262
130      3.02     1.95  1.0000
   7    9 -12      123      262
152    -1.11     0.27  1.0000
   5    9 -12      434      210
443      0.14     3.02  1.0000
   5    9 -12      366      251
373      0.14     3.02  1.0000
   3    9 -12      326      205
225    -2.59     0.56  1.0000
   3    9 -12        9      217
-77    -2.59     0.56  1.0000
   1    9 -12      704      206
787    -0.12    -1.35  1.0000
   1    9 -12      533      238
605    -0.12    -1.35  1.0000
  -1    9 -12      215      296
```

313 1.94 2.42 1.0000
-1 9 -12 117 185
191 1.94 2.42 1.0000
-3 9 -12 610 340
781 6.77 1.92 1.0000
-3 9 -12 157 189
216 6.77 1.92 1.0000
-5 9 -12 1387 342
1246 8.01 -3.41 1.0000
-5 9 -12 663 198
536 8.01 -3.41 1.0000
-7 9 -12 141 196
130 2.99 3.26 1.0000
-7 9 -12 623 368
629 2.99 3.26 1.0000
7 -9 12 26 187
-142 2.99 -3.26 1.0000
-9 9 -12 161 200
268 3.00 -1.93 1.0000
-9 9 -12 539 389
727 3.00 -1.93 1.0000
9 -9 12 208 201
329 3.00 1.93 1.0000
-11 9 -12 638 391
911 -0.85 -4.19 1.0000
-11 9 -12 126 210
261 -0.85 -4.19 1.0000
11 -9 12 24 243
-199 -0.85 4.19 1.0000
-13 9 -12 1511 349
1715 -2.40 3.20 1.0000
-13 9 -12 637 237
766 -2.40 3.20 1.0000
13 -9 12 820 312
970 -2.40 -3.20 1.0000
14 8 -12 5 290
-122 1.82 0.78 1.0000
12 8 -12 250 288
123 -4.63 -0.22 1.0000
10 8 -12 30 274
7 -1.50 -1.97 1.0000
8 8 -12 49 274
191 -5.77 1.62 1.0000
6 8 -12 57 261

49 -2.40 -4.42 1.0000
4 8 -12 1255 226
1242 -1.71 0.68 1.0000
4 8 -12 1078 295
1065 -1.71 0.68 1.0000
2 8 -12 12 185
11 -1.05 -0.37 1.0000
2 8 -12 156 236
156 -1.05 -0.37 1.0000
0 8 -12 388 229
372 -1.48 0.82 1.0000
0 8 -12 285 187
271 -1.48 0.82 1.0000
-2 8 -12 21 291
98 -5.53 0.43 1.0000
-2 8 -12 41 176
-55 -5.53 0.43 1.0000
-4 8 -12 1242 421
2218 -10.27 1.46 1.0000
-4 8 -12 878 252
1720 -10.27 1.46 1.0000
-6 8 -12 431 339
603 -6.23 1.78 1.0000
-6 8 -12 94 171
153 -6.23 1.78 1.0000
-8 8 -12 78 195
244 -5.73 -1.62 1.0000
-8 8 -12 591 382
967 -5.73 -1.62 1.0000
8 -8 12 47 191
-59 -5.73 1.62 1.0000
-10 8 -12 9 186
-112 2.00 1.73 1.0000
-10 8 -12 1095 388
1151 2.00 1.73 1.0000
10 -8 12 9 215
-31 2.00 -1.73 1.0000
-12 8 -12 1261 244
1192 1.62 3.63 1.0000
-12 8 -12 1682 378
1605 1.62 3.63 1.0000
-14 8 -12 20 218
36 0.75 -3.53 1.0000
-14 8 -12 604 291

737 0.75 -3.53 1.0000
14 -8 12 258 281
343 0.75 3.53 1.0000
15 7 -12 9 308
-101 -1.78 -1.93 1.0000
13 7 -12 15 281
-308 3.33 -0.24 1.0000
11 7 -12 22 270
121 5.15 3.95 1.0000
9 7 -12 60 271
44 2.95 -3.22 1.0000
7 7 -12 546 277
419 4.48 2.04 1.0000
5 7 -12 444 266
396 -0.19 1.04 1.0000
3 7 -12 130 248
137 -0.48 0.00 1.0000
3 7 -12 0 167
-191 -0.48 0.00 1.0000
1 7 -12 495 255
524 0.21 -0.57 1.0000
1 7 -12 455 197
483 0.21 -0.57 1.0000
-1 7 -12 446 243
405 -0.68 1.22 1.0000
-1 7 -12 375 185
337 -0.68 1.22 1.0000
-3 7 -12 134 187
301 4.44 -2.84 1.0000
-3 7 -12 367 254
623 4.44 -2.84 1.0000
-5 7 -12 778 326
888 4.59 1.93 1.0000
-5 7 -12 643 203
741 4.59 1.93 1.0000
-7 7 -12 1199 361
1023 4.50 -1.98 1.0000
-7 7 -12 730 194
589 4.50 -1.98 1.0000
-9 7 -12 38 183
54 -1.08 -0.21 1.0000
-9 7 -12 932 380
1008 -1.08 -0.21 1.0000
9 -7 12 34 201

50 -1.08 0.21 1.0000
-11 7 -12 619 206
794 -2.96 0.05 1.0000
11 -7 12 315 265
444 -2.96 -0.05 1.0000
-13 7 -12 95 204
137 -1.07 -3.93 1.0000
-15 7 -12 3 224
17 -3.53 3.50 1.0000
15 -7 12 55 292
171 -3.53 -3.50 1.0000
14 6 -12 7 339
-201 -2.14 0.56 1.0000
12 6 -12 23 313
-40 -3.36 -2.43 1.0000
10 6 -12 73 302
289 -8.23 0.27 1.0000
8 6 -12 219 289
329 -6.31 -1.75 1.0000
6 6 -12 841 301
602 -5.99 -0.17 1.0000
4 6 -12 104 257
130 -1.09 -0.86 1.0000
2 6 -12 12 250
22 -1.77 -0.64 1.0000
2 6 -12 5 169
-29 -1.77 -0.64 1.0000
0 6 -12 819 271
767 -0.65 0.50 1.0000
0 6 -12 1031 206
973 -0.65 0.50 1.0000
-2 6 -12 1252 203
923 -3.87 2.12 1.0000
-2 6 -12 1766 271
1371 -3.87 2.12 1.0000
-4 6 -12 243 248
394 -4.68 0.44 1.0000
-4 6 -12 184 179
316 -4.68 0.44 1.0000
-6 6 -12 2170 359
1794 -5.97 0.17 1.0000
-6 6 -12 1407 219
1102 -5.97 0.17 1.0000
-8 6 -12 456 347

515	-2.33	4.45	1.0000	
-8	6	-12	198	168
226	-2.33	4.45	1.0000	
-10	6	-12	174	187
139	0.66	-1.27	1.0000	
10	-6	12	78	214
55	0.66	1.27	1.0000	
-12	6	-12	420	195
261	3.48	-1.41	1.0000	
12	-6	12	19	240
-148	3.48	1.41	1.0000	
-14	6	-12	14	209
31	4.13	1.80	1.0000	
14	-6	12	69	279
134	4.13	-1.80	1.0000	
15	5	-12	11	329
-229	2.86	-0.02	1.0000	
13	5	-12	5	319
-361	1.88	0.23	1.0000	
11	5	-12	58	298
-79	6.12	-2.46	1.0000	
9	5	-12	565	331
434	7.95	3.40	1.0000	
7	5	-12	143	294
177	4.63	-1.84	1.0000	
5	5	-12	16	281
21	-0.51	0.39	1.0000	
3	5	-12	1018	305
1063	-2.84	-1.66	1.0000	
1	5	-12	718	295
984	-4.70	1.31	1.0000	
1	5	-12	926	211
1227	-4.70	1.31	1.0000	
-1	5	-12	1464	285
1223	-1.56	-2.56	1.0000	
-1	5	-12	1731	226
1468	-1.56	-2.56	1.0000	
-3	5	-12	191	172
272	-0.96	2.52	1.0000	
-3	5	-12	323	250
427	-0.96	2.52	1.0000	
-5	5	-12	313	240
328	-0.38	-0.28	1.0000	
-5	5	-12	245	174

258	-0.38	-0.28	1.0000	
-7	5	-12	1182	330
1102	-0.29	-1.05	1.0000	
-9	5	-12	1814	375
1843	0.20	-2.94	1.0000	
-13	5	-12	28	195
122	-5.34	1.30	1.0000	
13	-5	12	40	240
-226	-5.34	-1.30	1.0000	
-15	5	-12	223	205
215	-3.39	-3.57	1.0000	
15	-5	12	32	273
-85	-3.39	3.57	1.0000	
16	4	-12	14	360
-89	-2.51	-2.11	1.0000	
14	4	-12	32	320
31	-0.09	-0.48	1.0000	
12	4	-12	31	292
-143	-4.25	2.30	1.0000	
10	4	-12	420	310
874	-7.75	-1.59	1.0000	
8	4	-12	532	316
1211	-10.19	-1.43	1.0000	
6	4	-12	17	278
53	-4.67	-0.47	1.0000	
4	4	-12	1264	324
1366	-1.25	-1.11	1.0000	
2	4	-12	2083	322
1569	5.04	2.00	1.0000	
0	4	-12	174	270
150	1.21	-0.12	1.0000	
0	4	-12	249	177
221	1.21	-0.12	1.0000	
-2	4	-12	195	171
162	2.72	-1.04	1.0000	
-2	4	-12	238	247
203	2.72	-1.04	1.0000	
-4	4	-12	1357	283
1460	-1.24	1.12	1.0000	
-4	4	-12	1094	204
1187	-1.24	1.12	1.0000	
-6	4	-12	264	236
306	-1.10	0.87	1.0000	
-6	4	-12	3	162

-20 -1.10 0.87 1.0000
-8 4 -12 1513 235
1494 -1.70 -0.64 1.0000
-10 4 -12 694 201
796 2.09 3.76 1.0000
10 -4 12 465 213
542 2.09 -3.76 1.0000
-12 4 -12 195 192
362 3.95 -2.34 1.0000
-14 4 -12 359 221
623 5.17 -0.93 1.0000
14 -4 12 109 274
270 5.17 0.93 1.0000
-16 4 -12 160 235
283 3.25 1.67 1.0000
16 -4 12 521 327
729 3.25 -1.67 1.0000
15 3 -12 2 321
-474 -0.21 1.16 1.0000
13 3 -12 28 318
-401 4.39 -1.24 1.0000
11 3 -12 34 299
14 2.74 -1.29 1.0000
9 3 -12 182 295
250 6.81 -1.88 1.0000
7 3 -12 4 263
53 4.30 2.88 1.0000
5 3 -12 99 265
160 -0.88 -2.55 1.0000
3 3 -12 459 276
678 -6.19 0.35 1.0000
1 3 -12 1197 339
1747 -8.48 -1.80 1.0000
-1 3 -12 47 169
252 -8.35 1.14 1.0000
-1 3 -12 49 259
257 -8.35 1.14 1.0000
-3 3 -12 509 269
745 -6.12 -0.30 1.0000
-3 3 -12 784 209
1078 -6.12 -0.30 1.0000
-5 3 -12 1474 232
1538 -2.95 1.62 1.0000
-5 3 -12 1502 295

```
1566    -2.95     1.62  1.0000
  -7    3 -12      288       241
292    -0.29     0.07  1.0000
  -7    3 -12      123       178
125    -0.29     0.07  1.0000
  -9    3 -12      311       174
211    -2.67    -0.49  1.0000
  -9    3 -12     1406       294
1183    -2.67    -0.49  1.0000
 -13    3 -12       28       189
99    -4.16    -0.98  1.0000
  13   -3  12       24       252
-4    -4.16     0.98  1.0000
 -15    3 -12      387       207
232    -3.94    -0.39  1.0000
  15   -3  12       21       301
-64    -3.94     0.39  1.0000
  16    2 -12       16       319
-467     3.43     0.14  1.0000
  14    2 -12       10       317
-132    -2.80     0.00  1.0000
  12    2 -12        9       293
-37    -1.88    -1.74  1.0000
  10    2 -12       34       266
82    -4.43     1.69  1.0000
   8    2 -12       41       264
-27    -5.54    -0.50  1.0000
   6    2 -12      918       292
645    -3.77    -2.09  1.0000
   4    2 -12      348       262
306     2.69     1.07  1.0000
   2    2 -12       40       264
79     3.29    -1.70  1.0000
   0    2 -12      702       317
1786    13.65     0.70  1.0000
  -2    2 -12       18       258
-29     3.26     1.70  1.0000
  -2    2 -12      226       182
324     3.26     1.70  1.0000
  -4    2 -12     2255       229
1722     5.04    -1.98  1.0000
  -4    2 -12     1969       296
1473     5.04    -1.98  1.0000
  -6    2 -12        9       162
```

18 -1.78 0.72 1.0000
-6 2 -12 5 231
-78 -1.78 0.72 1.0000
-8 2 -12 267 221
268 -1.05 0.34 1.0000
-8 2 -12 47 166
47 -1.05 0.34 1.0000
-14 2 -12 53 189
121 4.04 -0.72 1.0000
14 -2 12 33 291
85 4.04 0.72 1.0000
-16 2 -12 59 206
67 2.06 -3.03 1.0000
16 -2 12 29 300
29 2.06 3.03 1.0000
15 1 -12 14 320
-364 0.29 -3.29 1.0000
13 1 -12 3 266
-427 -1.35 0.55 1.0000
11 1 -12 25 257
-106 4.33 -0.07 1.0000
9 1 -12 12 243
-19 1.84 -2.37 1.0000
7 1 -12 490 267
444 -0.72 -1.21 1.0000
5 1 -12 1315 294
1087 -1.57 2.55 1.0000
3 1 -12 8 251
119 -8.33 -1.16 1.0000
1 1 -12 201 247
-54 -12.28 0.58 1.0000
-1 1 -12 433 246
88 -12.28 -0.58 1.0000
-1 1 -12 794 176
319 -12.28 -0.58 1.0000
-3 1 -12 1138 307
1676 -8.54 1.72 1.0000
-3 1 -12 1107 229
1638 -8.54 1.72 1.0000
-5 1 -12 938 217
1235 -4.63 -1.28 1.0000
-5 1 -12 686 279
942 -4.63 -1.28 1.0000
-7 1 -12 363 182

388 0.16 0.57 1.0000
-7 1 -12 456 246
484 0.16 0.57 1.0000
-9 1 -12 906 239
1003 0.00 1.44 1.0000
-9 1 -12 781 198
871 0.00 1.44 1.0000
-11 1 -12 272 189
413 -2.25 -2.49 1.0000
-13 1 -12 237 200
413 -2.06 -3.77 1.0000
13 -1 12 181 254
338 -2.06 3.77 1.0000
-15 1 -12 13 208
-4 -2.67 1.62 1.0000
15 -1 12 13 291
-137 -2.67 -1.62 1.0000
16 0 -12 0 307
-243 -0.10 0.47 1.0000
14 0 -12 20 267
-417 3.92 0.15 1.0000
12 0 -12 1 244
-179 0.41 -0.80 1.0000
10 0 -12 8 239
-80 -2.35 0.89 1.0000
8 0 -12 187 233
173 -1.33 -0.84 1.0000
6 0 -12 324 242
291 -0.66 -0.50 1.0000
4 0 -12 166 241
140 1.32 0.16 1.0000
2 0 -12 910 322
2103 13.59 -0.78 1.0000
0 0 -12 1828 386
2394 5.35 -0.03 1.0000
-2 0 -12 684 222
1756 13.64 -0.68 1.0000
-2 0 -12 846 315
2011 13.64 -0.68 1.0000
-4 0 -12 181 244
156 1.22 0.14 1.0000
-4 0 -12 589 184
544 1.22 0.14 1.0000
-6 0 -12 739 183

693 -0.60 -0.47 1.0000
-6 0 -12 481 262
444 -0.60 -0.47 1.0000
-8 0 -12 157 176
145 -1.44 -0.82 1.0000
-8 0 -12 103 238
93 -1.44 -0.82 1.0000
-10 0 -12 727 223
615 -2.32 0.99 1.0000
-10 0 -12 8 172
-79 -2.32 0.99 1.0000
10 0 12 8 190
-138 -2.32 -0.99 1.0000
-12 0 -12 143 174
151 0.39 -0.75 1.0000
12 0 12 81 221
87 0.39 0.75 1.0000
-14 0 -12 328 200
274 4.15 0.16 1.0000
14 0 12 172 285
128 4.15 -0.16 1.0000
-16 0 -12 358 231
364 -0.05 0.59 1.0000
16 0 12 7 294
8 -0.05 -0.59 1.0000
15 -1 -12 12 279
-400 -2.58 1.62 1.0000
13 -1 -12 163 287
314 -2.09 -3.76 1.0000
11 -1 -12 36 261
100 -2.35 -2.61 1.0000
9 -1 -12 291 246
350 -0.18 1.44 1.0000
7 -1 -12 252 235
272 0.19 0.54 1.0000
5 -1 -12 694 265
962 -4.71 -1.41 1.0000
3 -1 -12 971 289
1461 -8.44 1.69 1.0000
1 -1 -12 994 245
464 -12.29 -0.57 1.0000
-1 -1 -12 834 240
346 -12.32 0.46 1.0000
-3 -1 -12 27 245

195	-8.29	-1.04	1.0000	
-3	-1	-12	132	171
425	-8.29	-1.04	1.0000	
-5	-1	-12	1307	281
1079	-1.58	2.56	1.0000	
-5	-1	-12	1827	227
1556	-1.58	2.56	1.0000	
-7	-1	-12	445	178
404	-0.65	-1.17	1.0000	
-7	-1	-12	164	252
139	-0.65	-1.17	1.0000	
-9	-1	-12	13	242
-17	1.99	-2.39	1.0000	
-9	-1	-12	165	178
252	1.99	-2.39	1.0000	
-11	-1	-12	26	175
-45	4.39	-0.13	1.0000	
-11	-1	-12	461	197
699	4.39	-0.13	1.0000	
-13	-1	-12	273	179
233	-1.40	0.61	1.0000	
13	1	12	358	264
312	-1.40	-0.61	1.0000	
-15	-1	-12	63	193
136	0.33	-3.21	1.0000	
15	1	12	14	294
-95	0.33	3.21	1.0000	
16	-2	-12	18	276
-453	1.99	-3.06	1.0000	
14	-2	-12	22	254
-166	3.95	-0.82	1.0000	
12	-2	-12	47	255
-189	4.15	4.21	1.0000	
10	-2	-12	150	256
242	2.14	-2.97	1.0000	
8	-2	-12	50	223
50	-0.99	0.33	1.0000	
6	-2	-12	5	214
-136	-1.69	0.72	1.0000	
4	-2	-12	1860	289
1386	4.95	-2.06	1.0000	
2	-2	-12	233	242
338	3.40	1.74	1.0000	
0	-2	-12	780	297

```
1901    13.58     0.73  1.0000
  -2   -2 -12      125       229
200     3.34   -1.80  1.0000
  -4   -2 -12      444       175
401     2.69     1.00  1.0000
  -4   -2 -12       46       238
27     2.69     1.00  1.0000
  -6   -2 -12      584       255
369    -3.83   -2.08  1.0000
  -6   -2 -12     1245       193
920    -3.83   -2.08  1.0000
  -8   -2 -12       36       167
124    -5.57   -0.45  1.0000
 -10   -2 -12       24       165
62   -4.50     1.69  1.0000
 -10   -2 -12       31       227
-159    -4.50     1.69  1.0000
  10    2  12       20       195
54   -4.50   -1.69  1.0000
 -12   -2 -12      494       184
381    -1.70   -1.65  1.0000
  12    2  12        7       231
-2   -1.70     1.65  1.0000
 -14   -2 -12      394       192
284    -2.77     0.04  1.0000
  14    2  12       10       272
-7   -2.77   -0.04  1.0000
 -16   -2 -12       15       205
56     3.56     0.20  1.0000
  16    2  12       27       309
77     3.56   -0.20  1.0000
  15   -3 -12       21       266
-77    -3.98   -0.40  1.0000
  13   -3 -12       25       249
-106    -4.21   -0.95  1.0000
  11   -3 -12       22       247
-15    -3.04   -2.64  1.0000
   9   -3 -12      149       247
83   -2.64   -0.60  1.0000
   7   -3 -12        0       224
-10    -0.50     0.00  1.0000
   5   -3 -12     1478       287
1532    -2.78     1.61  1.0000
   3   -3 -12      408       245
```

```
618    -6.24   -0.47  1.0000
   1   -3 -12       20      218
171    -8.30    0.93  1.0000
  -1   -3 -12     1244      288
1799    -8.49   -1.72  1.0000
  -3   -3 -12      524      247
759    -6.23    0.37  1.0000
  -3   -3 -12      711      199
987    -6.23    0.37  1.0000
  -5   -3 -12      132      163
200    -0.94   -2.53  1.0000
  -5   -3 -12       31      236
66   -0.94   -2.53  1.0000
  -7   -3 -12       54      161
174     4.46    2.91  1.0000
  -7   -3 -12       38      241
-89     4.46    2.91  1.0000
  -9   -3 -12      245      184
333     6.85   -1.86  1.0000
  -9   -3 -12       67      254
-14     6.85   -1.86  1.0000
 -11   -3 -12       13      218
-111     2.84   -1.22  1.0000
 -11   -3 -12      373      188
326     2.84   -1.22  1.0000
  11    3  12       13      204
-43     2.84    1.22  1.0000
 -13   -3 -12       71      189
80     4.56   -1.28  1.0000
  13    3  12       30      247
-278     4.56    1.28  1.0000
 -15   -3 -12      573      215
545    -0.28    1.16  1.0000
  15    3  12      290      301
269    -0.28   -1.16  1.0000
  16   -4 -12       17      276
-47     3.11    1.76  1.0000
  14   -4 -12      345      303
615     5.30   -1.14  1.0000
  12   -4 -12       38      242
122     3.87   -2.36  1.0000
  10   -4 -12      499      250
570     1.87    3.80  1.0000
   8   -4 -12      956      263
```

```
948    -1.68    -0.74  1.0000
   6   -4 -12        56       221
76    -1.13     0.83  1.0000
   4   -4 -12      1074       259
1154    -1.08     1.10  1.0000
   2   -4 -12       166       215
134     2.62    -1.07  1.0000
   0   -4 -12       174       220
147     1.31    -0.20  1.0000
  -2   -4 -12      1652       259
1202     4.99     1.95  1.0000
  -4   -4 -12      1069       208
1148    -1.06    -1.18  1.0000
  -4   -4 -12       950       256
1025    -1.06    -1.18  1.0000
  -6   -4 -12        30       229
-345    -4.74    -0.51  1.0000
  -6   -4 -12       185       171
318    -4.74    -0.51  1.0000
  -8   -4 -12       337       268
908   -10.23    -1.44  1.0000
 -10   -4 -12       369       267
790    -7.58    -1.65  1.0000
 -10   -4 -12       859       232
1459    -7.58    -1.65  1.0000
  10    4  12       698       236
1246    -7.58     1.65  1.0000
 -12   -4 -12        71       188
147    -4.43     2.36  1.0000
  12    4  12        33       248
-199    -4.43    -2.36  1.0000
 -14   -4 -12       516       214
518     0.02    -0.41  1.0000
  14    4  12         1       287
1     0.02     0.41  1.0000
 -16   -4 -12        91       211
170    -2.40    -2.05  1.0000
  16    4  12        13       295
-1    -2.40     2.05  1.0000
  15   -5 -12        55       268
32    -3.39    -3.56  1.0000
  13   -5 -12        42       263
-38    -5.45     1.29  1.0000
  11   -5 -12        11       233
```

-39	-2.91	0.05	1.0000	
9	-5	-12	343	247
349	0.19	-3.00	1.0000	
7	-5	-12	285	218
245	-0.12	-1.07	1.0000	
5	-5	-12	141	216
158	-0.54	-0.47	1.0000	
3	-5	-12	99	212
158	-0.87	2.48	1.0000	
1	-5	-12	1746	276
1478	-1.58	-2.60	1.0000	
-1	-5	-12	776	252
1052	-4.67	1.31	1.0000	
-3	-5	-12	1072	258
1126	-2.95	-1.63	1.0000	
-5	-5	-12	1	218
-38	-0.49	0.39	1.0000	
-5	-5	-12	307	179
327	-0.49	0.39	1.0000	
-7	-5	-12	42	234
42	4.63	-1.85	1.0000	
-7	-5	-12	441	192
524	4.63	-1.85	1.0000	
-9	-5	-12	599	200
469	7.96	3.44	1.0000	
-9	-5	-12	100	244
-251	7.96	3.44	1.0000	
9	5	12	501	198
380	7.96	-3.44	1.0000	
-11	-5	-12	166	191
40	6.30	-2.42	1.0000	
11	5	12	61	205
-30	6.30	2.42	1.0000	
-13	-5	-12	173	193
237	1.97	0.17	1.0000	
13	5	12	5	263
-85	1.97	-0.17	1.0000	
-15	-5	-12	284	225
410	2.96	-0.04	1.0000	
15	5	12	12	287
-321	2.96	0.04	1.0000	
14	-6	-12	26	267
-206	4.01	1.88	1.0000	
12	-6	-12	211	252

105	3.39	-1.50	1.0000	
10	-6	-12	93	226
67	0.69	-1.31	1.0000	
8	-6	-12	37	218
20	-2.46	4.51	1.0000	
6	-6	-12	770	250
541	-5.96	0.14	1.0000	
4	-6	-12	30	219
-15	-4.71	0.51	1.0000	
2	-6	-12	1109	253
812	-3.74	1.99	1.0000	
0	-6	-12	658	231
615	-0.64	0.39	1.0000	
-2	-6	-12	15	209
28	-1.72	-0.80	1.0000	
-4	-6	-12	72	182
95	-1.13	-0.89	1.0000	
-4	-6	-12	2	212
4	-1.13	-0.89	1.0000	
-6	-6	-12	968	202
715	-5.92	-0.18	1.0000	
-6	-6	-12	551	233
358	-5.92	-0.18	1.0000	
-8	-6	-12	276	175
404	-6.19	-1.87	1.0000	
-8	-6	-12	56	221
-208	-6.19	-1.87	1.0000	
-10	-6	-12	125	190
399	-8.40	0.31	1.0000	
10	6	12	211	203
541	-8.40	-0.31	1.0000	
-12	-6	-12	22	187
-99	-3.18	-2.47	1.0000	
12	6	12	22	247
-61	-3.18	2.47	1.0000	
-14	-6	-12	366	206
278	-2.19	0.72	1.0000	
14	6	12	139	286
87	-2.19	-0.72	1.0000	
15	-7	-12	12	271
83	-3.65	3.47	1.0000	
13	-7	-12	168	257
225	-0.94	-3.95	1.0000	
11	-7	-12	298	252

424	-2.98	0.05	1.0000	
9	-7	-12	2	216
-67	-1.12	-0.22	1.0000	
7	-7	-12	236	215
147	4.50	-2.07	1.0000	
5	-7	-12	290	218
354	4.66	1.74	1.0000	
3	-7	-12	282	235
507	4.35	-2.94	1.0000	
1	-7	-12	328	222
291	-0.74	1.15	1.0000	
-1	-7	-12	347	226
371	0.07	-0.56	1.0000	
-3	-7	-12	0	197
-253	-0.37	-0.11	1.0000	
-5	-7	-12	643	196
584	-0.27	1.06	1.0000	
-5	-7	-12	2	218
-124	-0.27	1.06	1.0000	
-7	-7	-12	516	199
392	4.47	2.03	1.0000	
-7	-7	-12	334	236
232	4.47	2.03	1.0000	
-9	-7	-12	127	178
115	3.07	-3.18	1.0000	
-9	-7	-12	26	221
-270	3.07	-3.18	1.0000	
9	7	12	78	196
60	3.07	3.18	1.0000	
-11	-7	-12	55	179
-252	5.20	3.83	1.0000	
11	7	12	136	244
336	5.20	-3.83	1.0000	
-13	-7	-12	59	209
71	3.45	-0.19	1.0000	
13	7	12	16	270
-48	3.45	0.19	1.0000	
-15	-7	-12	338	231
414	-1.68	-1.93	1.0000	
15	7	12	229	286
290	-1.68	1.93	1.0000	
14	-8	-12	17	257
-144	0.71	-3.50	1.0000	
12	-8	-12	540	257

495 1.51 3.72 1.0000
10 -8 -12 147 230
161 1.92 1.66 1.0000
8 -8 -12 117 228
312 -5.81 -1.61 1.0000
6 -8 -12 57 208
-45 -6.26 1.85 1.0000
4 -8 -12 810 333
1616 -10.19 1.41 1.0000
2 -8 -12 42 212
-53 -5.59 0.47 1.0000
0 -8 -12 166 213
155 -1.31 0.75 1.0000
-2 -8 -12 2 206
-115 -1.00 -0.37 1.0000
-4 -8 -12 801 248
791 -1.70 0.71 1.0000
-6 -8 -12 35 218
-10 -2.41 -4.53 1.0000
-6 -8 -12 35 176
-13 -2.41 -4.53 1.0000
-8 -8 -12 64 201
220 -5.78 1.65 1.0000
-8 -8 -12 1 224
58 -5.78 1.65 1.0000
8 8 12 65 202
221 -5.78 -1.65 1.0000
-10 -8 -12 297 195
211 -1.41 -1.97 1.0000
10 8 12 212 242
140 -1.41 1.97 1.0000
-12 -8 -12 294 219
156 -4.67 -0.17 1.0000
12 8 12 367 259
209 -4.67 0.17 1.0000
-14 -8 -12 151 223
211 1.89 0.55 1.0000
14 8 12 5 290
-116 1.89 -0.55 1.0000
13 -9 -12 454 275
562 -2.42 3.21 1.0000
11 -9 -12 15 252
79 -0.89 -4.20 1.0000
9 -9 -12 28 224

79 3.06 -1.84 1.0000
7 -9 -12 25 219
-72 3.02 3.16 1.0000
5 -9 -12 508 243
386 7.90 -3.40 1.0000
3 -9 -12 323 235
429 6.84 1.80 1.0000
1 -9 -12 91 226
154 1.88 2.36 1.0000
-1 -9 -12 497 245
576 -0.14 -1.51 1.0000
-3 -9 -12 75 216
29 -2.79 0.52 1.0000
-5 -9 -12 78 225
77 0.22 2.91 1.0000
-5 -9 -12 498 196
512 0.22 2.91 1.0000
-7 -9 -12 2 220
-174 -1.10 0.18 1.0000
-7 -9 -12 2 194
-24 -1.10 0.18 1.0000
7 9 12 458 209
513 -1.10 -0.18 1.0000
-9 -9 -12 84 203
166 3.06 1.81 1.0000
-9 -9 -12 17 218
-213 3.06 1.81 1.0000
9 9 12 32 235
85 3.06 -1.81 1.0000
-11 -9 -12 646 249
795 2.43 0.32 1.0000
11 9 12 907 291
1083 2.43 -0.32 1.0000
-13 -9 -12 78 221
85 -0.17 -2.23 1.0000
13 9 12 275 289
296 -0.17 2.23 1.0000
12 -10 -12 173 233
204 3.13 -2.29 1.0000
10 -10 -12 26 236
-154 -3.74 2.32 1.0000
8 -10 -12 8 231
-315 -1.48 1.99 1.0000
6 -10 -12 14 229

```
113    -8.33    -0.24  1.0000
   4 -10 -12      704       279
1261    -7.66     1.56  1.0000
   2 -10 -12       87       224
169    -4.47    -1.73  1.0000
   0 -10 -12      193       228
135    -2.43    -0.93  1.0000
  -2 -10 -12       17       228
-71     2.14     2.90  1.0000
  -4 -10 -12      806       261
908     2.00    -3.84  1.0000
  -6 -10 -12        3       231
-68     0.66     1.33  1.0000
  -6 -10 -12      237       203
194     0.66     1.33  1.0000
  -8 -10 -12      106       234
117     1.90    -1.63  1.0000
  -8 -10 -12      110       208
121     1.90    -1.63  1.0000
   8  10  12      292       237
315     1.90     1.63  1.0000
 -10 -10 -12       25       225
-88    -3.71    -2.30  1.0000
 -10 -10 -12      233       209
113    -3.71    -2.30  1.0000
  10  10  12       25       241
-3    -3.71     2.30  1.0000
 -12 -10 -12      100       222
151     2.63     2.10  1.0000
  12  10  12      115       289
166     2.63    -2.10  1.0000
  11 -11 -12      335       262
292    -0.75     2.73  1.0000
   9 -11 -12      542       267
676     2.35    -0.34  1.0000
   7 -11 -12       55       231
-16     5.15    -3.88  1.0000
   5 -11 -12       59       228
-138     6.20     2.49  1.0000
   3 -11 -12       12       230
-127     2.76     1.28  1.0000
   1 -11 -12       50       229
143     4.34     0.02  1.0000
  -1 -11 -12      196       246
```

320 -2.31 2.52 1.0000
-3 -11 -12 22 231
-96 -3.13 2.65 1.0000
-5 -11 -12 316 285
236 -3.00 -0.09 1.0000
-5 -11 -12 12 241
-45 -3.00 -0.09 1.0000
-5 -11 -12 115 209
67 -3.00 -0.09 1.0000
-7 -11 -12 217 264
325 -2.96 -0.11 1.0000
-7 -11 -12 250 216
365 -2.96 -0.11 1.0000
7 11 12 567 258
734 -2.96 0.11 1.0000
-9 -11 -12 24 241
-127 -0.84 4.18 1.0000
-9 -11 -12 24 211
-127 -0.84 4.18 1.0000
9 11 12 24 252
-98 -0.84 -4.18 1.0000
-11 -11 -12 760 243
701 -0.67 -2.62 1.0000
-11 -11 -12 250 240
213 -0.67 -2.62 1.0000
11 11 12 300 270
262 -0.67 2.62 1.0000
10 -12 -12 12 257
21 2.62 -2.05 1.0000
8 -12 -12 436 247
260 -4.68 0.24 1.0000
6 -12 -12 22 246
-35 -3.26 2.45 1.0000
4 -12 -12 50 235
113 -4.39 -2.33 1.0000
2 -12 -12 234 242
155 -1.84 1.68 1.0000
0 -12 -12 1 244
-226 0.38 0.76 1.0000
-2 -12 -12 165 236
64 4.16 -4.27 1.0000
-2 -12 -12 47 239
-181 4.16 -4.27 1.0000
-4 -12 -12 27 253

-61	3.92	2.30	1.0000	
-4	-12	-12	27	259
-18	3.92	2.30	1.0000	
-6	-12	-12	19	247
-136	3.43	1.50	1.0000	
-6	-12	-12	258	217
138	3.43	1.50	1.0000	
-8	-12	-12	280	275
241	1.61	-3.71	1.0000	
-8	-12	-12	744	252
692	1.61	-3.71	1.0000	
8	12	12	676	270
622	1.61	3.71	1.0000	
-10	-12	-12	311	230
357	3.11	2.26	1.0000	
-10	-12	-12	20	263
-121	3.11	2.26	1.0000	
10	12	12	20	268
-16	3.11	-2.26	1.0000	
9	-13	-12	154	270
169	-0.19	2.31	1.0000	
9	-13	-12	445	268
475	-0.19	2.31	1.0000	
7	-13	-12	15	260
-145	3.35	0.27	1.0000	
5	-13	-12	44	251
79	1.92	-0.24	1.0000	
3	-13	-12	69	256
78	4.47	1.26	1.0000	
1	-13	-12	143	254
115	-1.34	-0.57	1.0000	
1	-13	-12	426	228
378	-1.34	-0.57	1.0000	
-1	-13	-12	375	282
590	-2.14	3.72	1.0000	
-1	-13	-12	399	245
620	-2.14	3.72	1.0000	
-3	-13	-12	79	259
185	-4.22	0.93	1.0000	
-3	-13	-12	3	263
14	-4.22	0.93	1.0000	
-5	-13	-12	5	266
56	-5.44	-1.35	1.0000	
-5	-13	-12	42	317

-21	-5.44	-1.35	1.0000	
-7	-13	-12	188	275
249	-1.06	3.92	1.0000	
-7	-13	-12	22	232
-18	-1.06	3.92	1.0000	
-9	-13	-12	442	282
552	-2.44	-3.18	1.0000	
-9	-13	-12	560	267
685	-2.44	-3.18	1.0000	
9	13	12	903	296
1061	-2.44	3.18	1.0000	
8	-14	-12	190	275
258	1.88	-0.59	1.0000	
8	-14	-12	5	272
-54	1.88	-0.59	1.0000	
6	-14	-12	282	264
204	-2.21	-0.61	1.0000	
6	-14	-12	311	241
229	-2.21	-0.61	1.0000	
4	-14	-12	392	267
391	-0.06	0.49	1.0000	
4	-14	-12	0	228
-72	-0.06	0.49	1.0000	
2	-14	-12	11	254
-166	-2.86	-0.01	1.0000	
2	-14	-12	395	236
280	-2.86	-0.01	1.0000	
0	-14	-12	21	249
-94	3.99	-0.23	1.0000	
0	-14	-12	263	251
209	3.99	-0.23	1.0000	
-2	-14	-12	6	269
10	3.99	0.77	1.0000	
-2	-14	-12	22	293
-89	3.99	0.77	1.0000	
-4	-14	-12	348	317
610	5.18	1.06	1.0000	
-4	-14	-12	354	294
618	5.18	1.06	1.0000	
-6	-14	-12	27	235
-108	4.12	-1.91	1.0000	
-6	-14	-12	27	274
-28	4.12	-1.91	1.0000	
-8	-14	-12	151	254

213   0.76   3.51 1.0000
  -8 -14 -12   11   279
3   0.76   3.51 1.0000
   8 14 12   237   283
318   0.76   -3.51 1.0000
   7 -15 -12   73   263
109   -1.77   1.89 1.0000
   7 -15 -12   527   281
625   -1.77   1.89 1.0000
   5 -15 -12   11   263
-35   2.90   0.08 1.0000
   5 -15 -12   11   243
-192   2.90   0.08 1.0000
   3 -15 -12   158   263
144   -0.19   -1.22 1.0000
   3 -15 -12   432   276
410   -0.19   -1.22 1.0000
   1 -15 -12   14   249
-206   0.24   3.23 1.0000
   1 -15 -12   87   271
171   0.24   3.23 1.0000
  -1 -15 -12   12   275
-53   -2.62   -1.59 1.0000
  -1 -15 -12   38   272
93   -2.62   -1.59 1.0000
  -3 -15 -12   131   304
48   -3.97   0.34 1.0000
  -3 -15 -12   466   284
293   -3.97   0.34 1.0000
  -5 -15 -12   33   279
-148   -3.45   3.57 1.0000
  -5 -15 -12   33   344
-497   -3.45   3.57 1.0000
  -7 -15 -12   159   299
337   -3.66   -3.47 1.0000
  -7 -15 -12   89   270
230   -3.66   -3.47 1.0000
   4 -16 -12   269   277
403   -2.52   2.10 1.0000
   4 -16 -12   14   274
-136   -2.52   2.10 1.0000
   2 -16 -12   49   287
112   3.48   -0.16 1.0000
   2 -16 -12   246   290

374 3.48 -0.16 1.0000
0 -16 -12 331 294
339 -0.10 -0.53 1.0000
0 -16 -12 74 288
78 -0.10 -0.53 1.0000
-2 -16 -12 17 320
-71 1.96 2.99 1.0000
-2 -16 -12 17 271
-202 1.96 2.99 1.0000
-4 -16 -12 149 310
266 3.18 -1.74 1.0000
-4 -16 -12 50 355
125 3.18 -1.74 1.0000
1 14 -13 1332 440
1573 -0.14 -2.76 1.0000
1 14 -13 584 257
747 -0.14 -2.76 1.0000
-1 14 -13 483 252
547 2.50 -0.11 1.0000
-1 14 -13 375 412
431 2.50 -0.11 1.0000
4 13 -13 350 236
366 -0.81 1.84 1.0000
4 13 -13 737 393
763 -0.81 1.84 1.0000
2 13 -13 600 378
499 2.84 1.20 1.0000
2 13 -13 332 242
256 2.84 1.20 1.0000
0 13 -13 2054 416
1599 -0.39 4.63 1.0000
-2 13 -13 923 425
1081 -0.18 -2.69 1.0000
-2 13 -13 414 250
521 -0.18 -2.69 1.0000
-4 13 -13 1435 452
1390 -0.64 -0.34 1.0000
-4 13 -13 216 241
199 -0.64 -0.34 1.0000
7 12 -13 310 261
464 -2.01 -2.76 1.0000
7 12 -13 540 390
738 -2.01 -2.76 1.0000
5 12 -13 66 239

```
84     2.20     0.00  1.0000
   5  12 -13      461       354
515     2.20     0.00  1.0000
   3  12 -13      582       247
582    -2.98    -2.92  1.0000
   3  12 -13      518       368
516    -2.98    -2.92  1.0000
   1  12 -13      914       392
929     0.36    -0.28  1.0000
   1  12 -13      239       237
247     0.36    -0.28  1.0000
  -1  12 -13     1047       402
1190    -1.67     0.94  1.0000
  -1  12 -13      354       231
439    -1.67     0.94  1.0000
  -3  12 -13      266       243
386     1.32     3.28  1.0000
  -3  12 -13     1536       436
1816     1.32     3.28  1.0000
  -5  12 -13     3042       497
2992     0.96     0.76  1.0000
  -7  12 -13      203       243
156     1.18     2.90  1.0000
  -7  12 -13     1936       486
1808     1.18     2.90  1.0000
   7 -12  13       13       343
-59     1.18    -2.90  1.0000
   8  11 -13      406       242
266     3.21     2.11  1.0000
   8  11 -13       20       244
-172     3.21     2.11  1.0000
   6  11 -13        9       246
-141    -2.41     1.03  1.0000
   6  11 -13      234       244
321    -2.41     1.03  1.0000
   4  11 -13      377       360
515     3.18     3.28  1.0000
   4  11 -13      264       237
378     3.18     3.28  1.0000
   2  11 -13      615       371
707     0.99     4.90  1.0000
   2  11 -13      249       236
297     0.99     4.90  1.0000
   0  11 -13      156       222
```

```
269     2.25   -3.09  1.0000
   0  11 -13      773      400
1009     2.25   -3.09  1.0000
  -2  11 -13      897      389
879     1.38   -0.07  1.0000
  -2  11 -13      758      233
741     1.38   -0.07  1.0000
  -4  11 -13     1162      412
1051    -0.58   -2.93  1.0000
  -4  11 -13       64      219
34   -0.58   -2.93  1.0000
  -6  11 -13     2123      472
2490    -0.36   -3.34  1.0000
  -8  11 -13     1134      477
1419    -2.89   -2.61  1.0000
   9  10 -13      231      279
387    -3.84    1.28  1.0000
   7  10 -13        4      244
-59     1.09    1.39  1.0000
   7  10 -13      133      250
115     1.09    1.39  1.0000
   5  10 -13      394      242
496    -4.32   -2.41  1.0000
   5  10 -13      329      269
420    -4.32   -2.41  1.0000
   3  10 -13      265      224
249    -1.24   -1.91  1.0000
   3  10 -13      300      244
283    -1.24   -1.91  1.0000
   1  10 -13       12      340
-53    -2.91    0.73  1.0000
   1  10 -13      171      227
234    -2.91    0.73  1.0000
  -1  10 -13        3      209
20   -1.00    2.31  1.0000
  -1  10 -13      562      386
707    -1.00    2.31  1.0000
  -3  10 -13      132      205
203    -1.58    1.81  1.0000
  -3  10 -13      719      401
874    -1.58    1.81  1.0000
  -5  10 -13     1624      464
2426    -0.82    7.96  1.0000
  -7  10 -13      229      232
```

```
300     0.06     1.98  1.0000
  -7  10 -13     1639      452
1823     0.06     1.98  1.0000
  -9  10 -13     1253      463
1458     2.19     1.06  1.0000
  -9  10 -13       23      233
58     2.19     1.06  1.0000
  10   9 -13      185      292
268     2.20    -0.99  1.0000
   8   9 -13       76      281
131    -1.74    -2.00  1.0000
   6   9 -13       13      212
-174     3.14    -0.28  1.0000
   6   9 -13       13      257
-43     3.14    -0.28  1.0000
   4   9 -13      425      267
550     1.98     2.05  1.0000
   4   9 -13       70      227
124     1.98     2.05  1.0000
   2   9 -13      728      231
725     2.25     0.18  1.0000
   2   9 -13      800      279
798     2.25     0.18  1.0000
   0   9 -13      237      211
305     2.59     0.08  1.0000
   0   9 -13      318      347
396     2.59     0.08  1.0000
  -2   9 -13      141      200
116     0.74    -1.11  1.0000
  -2   9 -13      591      363
541     0.74    -1.11  1.0000
  -4   9 -13      246      204
163    -0.57    -5.34  1.0000
  -4   9 -13      772      402
645    -0.57    -5.34  1.0000
  -6   9 -13     2738      442
2371    -1.47    -5.04  1.0000
  -8   9 -13     1697      443
1533     1.07    -1.49  1.0000
  -8   9 -13      208      222
153     1.07    -1.49  1.0000
 -10   9 -13       22      217
-66    -3.80    -1.37  1.0000
 -10   9 -13      936      468
```

1236 -3.80 -1.37 1.0000
10 -9 13 5 271
7 -3.80 1.37 1.0000
11 8 -13 55 303
126 -2.98 2.54 1.0000
9 8 -13 5 266
-86 1.00 1.59 1.0000
7 8 -13 61 273
37 -4.68 2.82 1.0000
5 8 -13 63 212
80 -3.27 0.72 1.0000
5 8 -13 50 280
64 -3.27 0.72 1.0000
3 8 -13 80 208
169 -3.91 0.33 1.0000
3 8 -13 263 269
413 -3.91 0.33 1.0000
1 8 -13 380 252
356 -3.47 0.21 1.0000
1 8 -13 332 207
308 -3.47 0.21 1.0000
-1 8 -13 718 250
539 -2.94 1.49 1.0000
-1 8 -13 571 201
413 -2.94 1.49 1.0000
-3 8 -13 1173 363
810 -0.23 5.38 1.0000
-3 8 -13 39 195
-61 -0.23 5.38 1.0000
-5 8 -13 1278 416
1279 0.34 3.58 1.0000
-5 8 -13 508 214
502 0.34 3.58 1.0000
-7 8 -13 1010 244
1347 -0.13 4.28 1.0000
-9 8 -13 65 214
118 -1.69 2.02 1.0000
-9 8 -13 1295 446
1507 -1.69 2.02 1.0000
-11 8 -13 28 228
1 3.24 -2.11 1.0000
11 -8 13 20 276
-247 3.24 2.11 1.0000
12 7 -13 145 312

108 1.18 -2.77 1.0000
10 7 -13 28 291
56 0.02 -2.00 1.0000
8 7 -13 508 314
755 -0.07 -4.28 1.0000
6 7 -13 68 281
147 2.97 -2.14 1.0000
4 7 -13 169 203
226 2.00 -0.37 1.0000
4 7 -13 370 278
454 2.00 -0.37 1.0000
2 7 -13 255 270
264 2.26 -1.56 1.0000
2 7 -13 568 211
587 2.26 -1.56 1.0000
0 7 -13 951 294
1022 2.98 1.07 1.0000
0 7 -13 907 227
976 2.98 1.07 1.0000
-2 7 -13 1456 259
1693 -0.51 -3.54 1.0000
-2 7 -13 1794 416
2057 -0.51 -3.54 1.0000
-4 7 -13 1012 245
1254 -2.00 -3.46 1.0000
-4 7 -13 1737 404
2053 -2.00 -3.46 1.0000
-6 7 -13 1209 411
1390 -2.49 -1.60 1.0000
-8 7 -13 1164 413
1217 -4.62 -2.88 1.0000
-8 7 -13 103 194
88 -4.62 -2.88 1.0000
-10 7 -13 2343 459
2288 1.14 -1.34 1.0000
-12 7 -13 185 225
308 -1.99 2.78 1.0000
-12 7 -13 1427 433
1740 -1.99 2.78 1.0000
12 -7 13 146 276
256 -1.99 -2.78 1.0000
11 6 -13 56 315
128 -0.38 3.35 1.0000
9 6 -13 476 333

320	-1.56	5.02	1.0000	
7	6	-13	12	303
-213	-2.55	1.66	1.0000	
5	6	-13	439	305
508	-5.74	2.46	1.0000	
3	6	-13	290	197
376	-3.47	-0.10	1.0000	
3	6	-13	122	281
176	-3.47	-0.10	1.0000	
1	6	-13	11	255
41	-2.59	-0.78	1.0000	
1	6	-13	46	192
98	-2.59	-0.78	1.0000	
-1	6	-13	717	212
646	-3.57	1.78	1.0000	
-1	6	-13	551	276
487	-3.57	1.78	1.0000	
-3	6	-13	351	192
215	-0.56	3.49	1.0000	
-3	6	-13	543	247
370	-0.56	3.49	1.0000	
-5	6	-13	2887	463
2599	0.74	2.29	1.0000	
-5	6	-13	1752	246
1529	0.74	2.29	1.0000	
-7	6	-13	1336	415
1648	3.00	2.18	1.0000	
-9	6	-13	1614	435
1841	3.13	0.27	1.0000	
-11	6	-13	97	200
153	-2.37	-0.99	1.0000	
12	5	-13	170	326
157	0.87	-0.71	1.0000	
10	5	-13	4	329
81	-0.74	-7.91	1.0000	
8	5	-13	51	299
38	0.32	-3.56	1.0000	
6	5	-13	970	330
811	0.63	-2.23	1.0000	
4	5	-13	78	287
31	2.57	-3.20	1.0000	
2	5	-13	817	308
686	2.02	2.73	1.0000	
2	5	-13	746	201

621	2.02	2.73	1.0000	
0	5	-13	452	198
466	-0.81	0.02	1.0000	
0	5	-13	469	280
483	-0.81	0.02	1.0000	
-2	5	-13	1678	244
1651	1.24	-0.23	1.0000	
-2	5	-13	2099	413
2069	1.24	-0.23	1.0000	
-4	5	-13	247	197
290	-2.16	-0.37	1.0000	
-4	5	-13	653	280
725	-2.16	-0.37	1.0000	
-6	5	-13	947	300
1076	-5.80	-2.53	1.0000	
-6	5	-13	648	210
746	-5.80	-2.53	1.0000	
-8	5	-13	1558	421
1686	-3.24	-0.69	1.0000	
13	4	-13	1	354
-195	-0.70	0.40	1.0000	
11	4	-13	12	335
-218	-0.63	2.87	1.0000	
9	4	-13	38	310
-300	-0.57	5.34	1.0000	
7	4	-13	378	337
527	-1.96	3.44	1.0000	
5	4	-13	125	325
156	-2.13	0.39	1.0000	
3	4	-13	168	288
246	-2.69	-0.43	1.0000	
1	4	-13	40	272
-235	-5.02	-2.14	1.0000	
1	4	-13	40	186
-52	-5.02	-2.14	1.0000	
-1	4	-13	541	204
414	1.69	-3.21	1.0000	
-1	4	-13	597	275
464	1.69	-3.21	1.0000	
-3	4	-13	617	279
647	-1.90	-0.62	1.0000	
-3	4	-13	444	196
468	-1.90	-0.62	1.0000	
-5	4	-13	324	183

233	2.51	3.22	1.0000	
-5	4	-13	901	259
755	2.51	3.22	1.0000	
-7	4	-13	741	290
868	2.16	0.40	1.0000	
-7	4	-13	521	211
628	2.16	0.40	1.0000	
-9	4	-13	2111	445
2382	2.02	-1.97	1.0000	
-13	4	-13	298	209
308	-0.81	-1.76	1.0000	
13	-4	13	31	273
31	-0.81	1.76	1.0000	
12	3	-13	17	311
-250	1.47	-3.28	1.0000	
10	3	-13	84	308
142	-1.64	-1.75	1.0000	
8	3	-13	38	279
-268	-0.22	-5.32	1.0000	
6	3	-13	16	295
-91	-0.51	-3.42	1.0000	
4	3	-13	412	308
437	-2.01	0.65	1.0000	
2	3	-13	12	298
-24	2.32	1.97	1.0000	
0	3	-13	15	280
92	1.45	4.75	1.0000	
0	3	-13	73	187
204	1.45	4.75	1.0000	
-2	3	-13	929	219
1287	-3.77	3.64	1.0000	
-2	3	-13	535	304
811	-3.77	3.64	1.0000	
-4	3	-13	337	272
443	-2.66	0.40	1.0000	
-4	3	-13	287	188
386	-2.66	0.40	1.0000	
-6	3	-13	12	244
18	-3.44	0.14	1.0000	
-6	3	-13	16	177
-2	-3.44	0.14	1.0000	
-8	3	-13	748	289
996	-3.97	-0.37	1.0000	
-8	3	-13	127	191

```
237    -3.97    -0.37  1.0000
  13    2 -13       10       309
-555    -0.25     2.72  1.0000
  11    2 -13      152       305
143     1.32     0.10  1.0000
   9    2 -13        2       277
-411     0.74     1.10  1.0000
   7    2 -13      834       322
1002    -0.51     3.35  1.0000
   5    2 -13     1254       351
1224     1.24     0.32  1.0000
   3    2 -13      444       315
697    -3.66    -3.65  1.0000
   1    2 -13       61       282
197     1.04    -5.57  1.0000
  -1    2 -13       62       162
4    -2.64    -4.44  1.0000
  -1    2 -13       36       280
-84    -2.64    -4.44  1.0000
  -3    2 -13      290       183
195     2.32    -1.97  1.0000
  -3    2 -13       12       270
-165     2.32    -1.97  1.0000
  -5    2 -13      608       192
493     2.09    -2.66  1.0000
  -5    2 -13      667       275
547     2.09    -2.66  1.0000
  -7    2 -13      733       203
755     2.33     1.63  1.0000
  -7    2 -13      423       261
437     2.33     1.63  1.0000
  -9    2 -13      963       217
963     2.37    -0.21  1.0000
 -13    2 -13      199       203
137     2.83    -1.28  1.0000
  13   -2  13      212       274
148     2.83     1.28  1.0000
  14    1 -13        8       316
-465     2.48     0.11  1.0000
  12    1 -13        5       298
-449    -1.66    -0.95  1.0000
  10    1 -13        8       289
-348    -0.91    -2.27  1.0000
   8    1 -13      129       288
```

61	-2.87	-1.31	1.0000		
6	1	-13	94	293	
57	-3.54	-1.76	1.0000		
4	1	-13	350	285	
249	1.70	3.21	1.0000		
2	1	-13	36	257	
-407	-2.61	4.47	1.0000		
0	1	-13	50	262	
-42	-0.79	6.07	1.0000		
-2	1	-13	3	270	
54	1.13	5.60	1.0000		
-2	1	-13	65	185	
206	1.13	5.60	1.0000		
-4	1	-13	40	265	
-261	-5.00	2.19	1.0000		
-4	1	-13	342	182	
219	-5.00	2.19	1.0000		
-6	1	-13	10	173	
-115	-2.69	0.77	1.0000		
-6	1	-13	10	267	
-126	-2.69	0.77	1.0000		
-8	1	-13	240	256	
217	-3.43	-0.20	1.0000		
-8	1	-13	16	180	
-15	-3.43	-0.20	1.0000		
-14	1	-13	148	209	
238	-0.10	2.83	1.0000		
14	-1	13	122	310	
204	-0.10	-2.83	1.0000		
13	0	-13	29	286	
-298	-0.37	-4.68	1.0000		
11	0	-13	20	284	
-301	2.32	3.12	1.0000		
9	0	-13	9	273	
-88	2.55	-0.20	1.0000		
7	0	-13	513	288	
565	2.99	-1.03	1.0000		
5	0	-13	280	272	
294	-0.75	-0.12	1.0000		
3	0	-13	32	267	
-70	1.41	-4.70	1.0000		
1	0	-13	50	255	
-135	-0.76	-6.08	1.0000		
-1	0	-13	50	167	

-136	-0.76	-6.10	1.0000	
-1	0	-13	50	262
-289	-0.76	-6.10	1.0000	
-3	0	-13	78	175
208	1.39	-4.68	1.0000	
-3	0	-13	32	270
-52	1.39	-4.68	1.0000	
-5	0	-13	778	202
796	-0.72	-0.05	1.0000	
-5	0	-13	184	285
192	-0.72	-0.05	1.0000	
-7	0	-13	588	285
647	3.03	-1.01	1.0000	
-7	0	-13	1128	224
1214	3.03	-1.01	1.0000	
-9	0	-13	208	183
273	2.68	-0.05	1.0000	
-9	0	-13	479	260
578	2.68	-0.05	1.0000	
-13	0	-13	602	206
370	-0.41	-4.59	1.0000	
13	0	13	372	278
196	-0.41	4.59	1.0000	
12	-1	-13	0	270
-127	0.34	0.41	1.0000	
10	-1	-13	12	256
-131	-2.88	-0.67	1.0000	
8	-1	-13	16	242
-50	-3.44	-0.18	1.0000	
6	-1	-13	10	245
-107	-2.56	0.81	1.0000	
4	-1	-13	39	251
-108	-4.95	2.15	1.0000	
2	-1	-13	116	271
288	1.04	5.51	1.0000	
0	-1	-13	50	258
-180	-0.72	6.10	1.0000	
-2	-1	-13	35	261
-117	-2.63	4.40	1.0000	
-2	-1	-13	274	162
118	-2.63	4.40	1.0000	
-4	-1	-13	467	186
349	1.69	3.25	1.0000	
-4	-1	-13	276	277

185	1.69	3.25	1.0000	
-6	-1	-13	21	284
-14	-3.51	-1.75	1.0000	
-6	-1	-13	491	185
428	-3.51	-1.75	1.0000	
-8	-1	-13	14	262
-157	-2.94	-1.47	1.0000	
-8	-1	-13	608	185
443	-2.94	-1.47	1.0000	
-10	-1	-13	9	187
-107	-1.05	-2.34	1.0000	
-10	-1	-13	298	244
408	-1.05	-2.34	1.0000	
-12	-1	-13	57	195
91	-1.59	-0.87	1.0000	
12	1	13	53	237
86	-1.59	0.87	1.0000	
-14	-1	-13	46	191
63	2.53	0.10	1.0000	
14	1	13	9	290
-47	2.53	-0.10	1.0000	
13	-2	-13	13	277
-248	2.87	-1.19	1.0000	
11	-2	-13	124	281
149	1.03	-5.02	1.0000	
9	-2	-13	306	283
304	2.31	-0.23	1.0000	
7	-2	-13	60	255
57	2.36	1.54	1.0000	
5	-2	-13	202	271
136	2.05	-2.68	1.0000	
3	-2	-13	13	238
-136	2.34	-2.06	1.0000	
1	-2	-13	35	239
-222	-2.59	-4.41	1.0000	
-1	-2	-13	4	257
60	1.09	-5.56	1.0000	
-3	-2	-13	785	227
1115	-3.74	-3.63	1.0000	
-5	-2	-13	868	299
843	1.25	0.31	1.0000	
-5	-2	-13	1647	238
1613	1.25	0.31	1.0000	
-7	-2	-13	1500	257

1738	-0.48		3.50	1.0000
-7	-2	-13	939	312
1128	-0.48		3.50	1.0000
-9	-2	-13	433	214
387	0.77		1.20	1.0000
-11	-2	-13	838	257
819	1.45		0.09	1.0000
-11	-2	-13	661	212
643	1.45		0.09	1.0000
-13	-2	-13	93	201
145	-0.13		2.69	1.0000
13	2	13	10	262
-155	-0.13		-2.69	1.0000
12	-3	-13	23	278
-28	-2.93		2.97	1.0000
10	-3	-13	8	250
-183	-1.30		1.99	1.0000
8	-3	-13	19	240
-94	-3.79		-0.25	1.0000
6	-3	-13	16	237
-176	-3.51		0.03	1.0000
4	-3	-13	190	257
271	-2.67		0.41	1.0000
2	-3	-13	499	267
762	-3.62		3.62	1.0000
0	-3	-13	31	244
-51	1.35		4.62	1.0000
-2	-3	-13	13	245
-116	2.34		2.05	1.0000
-4	-3	-13	366	190
388	-1.89		0.61	1.0000
-4	-3	-13	30	265
33	-1.89		0.61	1.0000
-6	-3	-13	16	270
-209	-0.51		-3.43	1.0000
-6	-3	-13	582	202
406	-0.51		-3.43	1.0000
-8	-3	-13	39	176
-23	-0.23		-5.40	1.0000
-10	-3	-13	72	262
125	-1.47		-1.80	1.0000
-10	-3	-13	247	210
338	-1.47		-1.80	1.0000
-12	-3	-13	389	224

536 1.20 -3.33 1.0000
12 3 13 11 264
38 1.20 3.33 1.0000
13 -4 -13 6 277
-36 -0.82 -1.90 1.0000
11 -4 -13 31 271
56 3.26 -3.22 1.0000
9 -4 -13 50 261
96 1.99 -2.06 1.0000
7 -4 -13 18 260
39 2.03 0.36 1.0000
5 -4 -13 130 239
73 2.70 3.14 1.0000
3 -4 -13 432 268
459 -1.98 -0.60 1.0000
1 -4 -13 735 260
583 1.65 -3.27 1.0000
-1 -4 -13 39 238
-167 -4.97 -2.18 1.0000
-3 -4 -13 10 249
-20 -2.68 -0.44 1.0000
-3 -4 -13 88 190
146 -2.68 -0.44 1.0000
-5 -4 -13 206 183
246 -2.20 0.35 1.0000
-5 -4 -13 7 262
-380 -2.20 0.35 1.0000
-7 -4 -13 867 231
1094 -2.05 3.50 1.0000
-7 -4 -13 142 280
236 -2.05 3.50 1.0000
-9 -4 -13 38 168
-222 -0.53 5.31 1.0000
-11 -4 -13 12 209
-129 -0.56 2.96 1.0000
-11 -4 -13 12 257
-272 -0.56 2.96 1.0000
11 4 13 12 221
-81 -0.56 -2.96 1.0000
-13 -4 -13 12 205
8 -0.58 0.36 1.0000
13 4 13 189 277
175 -0.58 -0.36 1.0000
12 -5 -13 6 274

-68 2.17 -0.02 1.0000
10 -5 -13 204 264
273 -4.16 2.49 1.0000
8 -5 -13 15 236
-173 -3.31 -0.81 1.0000
6 -5 -13 68 243
56 -5.72 -2.41 1.0000
4 -5 -13 6 229
-124 -2.15 -0.40 1.0000
2 -5 -13 1455 300
1412 1.23 -0.44 1.0000
0 -5 -13 412 259
429 -0.68 0.15 1.0000
-2 -5 -13 357 251
268 2.06 2.62 1.0000
-4 -5 -13 434 196
335 2.61 -3.12 1.0000
-4 -5 -13 22 236
-365 2.61 -3.12 1.0000
-6 -5 -13 1028 291
858 0.75 -2.30 1.0000
-6 -5 -13 1345 228
1150 0.75 -2.30 1.0000
-8 -5 -13 197 190
186 0.44 -3.53 1.0000
-8 -5 -13 17 273
-278 0.44 -3.53 1.0000
-10 -5 -13 83 202
322 -0.86 -7.98 1.0000
-12 -5 -13 801 238
776 1.01 -0.75 1.0000
12 5 13 429 269
410 1.01 0.75 1.0000
11 -6 -13 10 245
-182 -2.47 -1.04 1.0000
9 -6 -13 14 239
-97 3.22 0.33 1.0000
7 -6 -13 19 248
-1 3.06 2.16 1.0000
5 -6 -13 1311 294
1124 0.65 2.25 1.0000
3 -6 -13 357 244
225 -0.46 3.37 1.0000
1 -6 -13 281 248

231	-3.52		1.76	1.0000
-1	-6	-13	11	230
-167	-2.70		-0.85	1.0000
-3	-6	-13	17	240
-97	-3.52		-0.11	1.0000
-5	-6	-13	119	186
127	-5.84		2.47	1.0000
-5	-6	-13	70	255
58	-5.84		2.47	1.0000
-7	-6	-13	12	201
-40	-2.49		1.63	1.0000
-7	-6	-13	12	254
-444	-2.49		1.63	1.0000
-9	-6	-13	339	193
207	-1.58		5.02	1.0000
-11	-6	-13	18	227
63	-0.35		3.31	1.0000
-11	-6	-13	15	261
-472	-0.35		3.31	1.0000
11	6	13	200	271
321	-0.35		-3.31	1.0000
12	-7	-13	16	266
62	-1.90		2.83	1.0000
10	-7	-13	4	252
-262	1.08		-1.44	1.0000
8	-7	-13	39	245
-16	-4.66		-2.73	1.0000
6	-7	-13	157	238
227	-2.52		-1.73	1.0000
4	-7	-13	602	288
792	-2.02		-3.49	1.0000
2	-7	-13	1124	287
1317	-0.53		-3.34	1.0000
0	-7	-13	665	270
726	2.94		0.96	1.0000
-2	-7	-13	326	253
336	2.36		-1.56	1.0000
-4	-7	-13	125	252
178	2.11		-0.38	1.0000
-4	-7	-13	257	193
331	2.11		-0.38	1.0000
-6	-7	-13	19	196
-59	3.06		-2.21	1.0000
-6	-7	-13	19	256

-116	3.06	-2.21	1.0000	
-8	-7 -13	847	241	
1163	-0.10	-4.34	1.0000	
-10	-7 -13	252	227	
326	0.10	-1.96	1.0000	
10	7 13	368	222	
457	0.10	1.96	1.0000	
-12	-7 -13	646	242	
569	1.14	-2.97	1.0000	
12	7 13	316	285	
257	1.14	2.97	1.0000	
11	-8 -13	19	265	
-56	3.25	-2.02	1.0000	
9	-8 -13	10	245	
-150	-1.77	2.02	1.0000	
7	-8 -13	536	286	
794	-0.02	4.37	1.0000	
5	-8 -13	87	243	
73	0.42	3.54	1.0000	
3	-8 -13	56	226	
2	-0.22	5.34	1.0000	
1	-8 -13	463	245	
325	-2.89	1.27	1.0000	
-1	-8 -13	377	250	
355	-3.47	0.15	1.0000	
-3	-8 -13	18	248	
65	-3.91	0.27	1.0000	
-5	-8 -13	16	206	
-117	-3.34	0.75	1.0000	
-5	-8 -13	16	252	
-46	-3.34	0.75	1.0000	
-7	-8 -13	138	201	
127	-4.66	2.84	1.0000	
-9	-8 -13	226	218	
167	1.08	1.54	1.0000	
9	8 13	53	208	
27	1.08	-1.54	1.0000	
-11	-8 -13	112	231	
209	-2.86	2.61	1.0000	
11	8 13	272	253	
417	-2.86	-2.61	1.0000	
10	-9 -13	21	261	
-153	-3.77	-1.21	1.0000	
8	-9 -13	58	251	

29 1.06 -1.65 1.0000
6 -9 -13 265 257
150 -1.61 -4.98 1.0000
4 -9 -13 38 230
-82 -0.59 -5.34 1.0000
2 -9 -13 2 241
-15 0.68 -1.18 1.0000
0 -9 -13 9 239
-198 2.59 0.17 1.0000
-2 -9 -13 341 253
340 2.30 0.24 1.0000
-4 -9 -13 10 247
-153 1.97 1.99 1.0000
-6 -9 -13 175 218
250 3.14 -0.31 1.0000
-6 -9 -13 16 257
36 3.14 -0.31 1.0000
-8 -9 -13 5 207
1 -1.76 -2.06 1.0000
-10 -9 -13 8 218
-136 2.17 -1.17 1.0000
-10 -9 -13 8 272
-337 2.17 -1.17 1.0000
10 9 13 113 243
181 2.17 1.17 1.0000
9 -10 -13 234 273
328 2.19 1.10 1.0000
7 -10 -13 5 255
-68 0.05 1.99 1.0000
5 -10 -13 5 248
66 -0.76 7.94 1.0000
3 -10 -13 120 249
186 -1.56 1.73 1.0000
1 -10 -13 90 245
153 -1.02 2.30 1.0000
-1 -10 -13 95 247
141 -2.92 0.66 1.0000
-3 -10 -13 8 249
-16 -1.40 -2.03 1.0000
-5 -10 -13 146 269
201 -4.19 -2.48 1.0000
-5 -10 -13 211 212
282 -4.19 -2.48 1.0000
-7 -10 -13 4 259

-134	1.06	1.38	1.0000	
-7	-10	-13	4	224
-228	1.06	1.38	1.0000	
-9	-10	-13	21	218
-63	-3.78	1.37	1.0000	
9	10	13	216	260
369	-3.78	-1.37	1.0000	
8	-11	-13	92	282
178	-2.91	-2.52	1.0000	
6	-11	-13	188	282
306	-0.40	-3.30	1.0000	
4	-11	-13	12	253
-324	-0.61	-2.90	1.0000	
2	-11	-13	857	286
836	1.36	-0.13	1.0000	
0	-11	-13	19	246
-21	2.19	-3.12	1.0000	
-2	-11	-13	234	271
278	1.02	4.90	1.0000	
-4	-11	-13	114	272
190	3.31	3.34	1.0000	
-6	-11	-13	10	264
-192	-2.48	1.01	1.0000	
-6	-11	-13	10	226
-43	-2.48	1.01	1.0000	
-8	-11	-13	28	269
1	3.25	2.01	1.0000	
-8	-11	-13	242	247
135	3.25	2.01	1.0000	
8	11	13	661	259
480	3.25	-2.01	1.0000	
7	-12	-13	139	262
100	1.18	2.85	1.0000	
5	-12	-13	347	282
331	0.90	0.69	1.0000	
3	-12	-13	70	266
135	1.29	3.33	1.0000	
1	-12	-13	4	269
-58	-1.60	0.89	1.0000	
-1	-12	-13	69	285
75	0.37	-0.37	1.0000	
-3	-12	-13	24	256
-52	-3.01	-3.03	1.0000	
-5	-12	-13	106	215

```
128      2.09     0.00  1.0000
  -5 -12 -13      318      290
359      2.09     0.00  1.0000
  -7 -12 -13      149      250
261     -1.95    -2.82  1.0000
  -7 -12 -13       28      290
86     -1.95    -2.82  1.0000
   4 -13 -13        1      264
-20     -0.65    -0.39  1.0000
   2 -13 -13       10      265
-146     -0.28    -2.73  1.0000
   0 -13 -13      693      288
441     -0.42     4.61  1.0000
  -2 -13 -13       64      278
29      2.75     1.29  1.0000
  -4 -13 -13       41      284
42     -0.89     1.82  1.0000
   1 -14 -13      365      304
419      2.46    -0.11  1.0000
  -1 -14 -13      247      297
360     -0.11    -2.84  1.0000
   4  10 -14      785      289
819     -1.91     1.73  1.0000
   4  10 -14      698      277
730     -1.91     1.73  1.0000
   2  10 -14      354      250
257     -0.72     2.76  1.0000
   2  10 -14      992      426
826     -0.72     2.76  1.0000
   0  10 -14      227      238
299      1.17     1.59  1.0000
   0  10 -14      973      443
1117      1.17     1.59  1.0000
  -2  10 -14      324      246
367     -0.68     0.95  1.0000
  -2  10 -14     1944      487
2049     -0.68     0.95  1.0000
  -4  10 -14     1375      452
1674      3.64     0.18  1.0000
  -4  10 -14       53      237
120      3.64     0.18  1.0000
   5   9 -14      533      281
452     -0.86    -2.96  1.0000
   5   9 -14      241      243
```

185	-0.86	-2.96	1.0000	
3	9 -14	3	243	
-115	1.17	-0.84	1.0000	
3	9 -14	223	272	
216	1.17	-0.84	1.0000	
1	9 -14	821	301	
1132	-0.62	-4.51	1.0000	
1	9 -14	614	257	
886	-0.62	-4.51	1.0000	
-1	9 -14	463	233	
480	0.72	0.09	1.0000	
-1	9 -14	1300	440	
1328	0.72	0.09	1.0000	
-3	9 -14	1	217	
-114	-0.05	-1.00	1.0000	
-3	9 -14	1243	452	
1164	-0.05	-1.00	1.0000	
-5	9 -14	2346	471	
2325	-4.83	-0.16	1.0000	
6	8 -14	482	317	
451	0.61	1.97	1.0000	
6	8 -14	18	231	
9	0.61	1.97	1.0000	
4	8 -14	292	282	
312	-0.58	3.24	1.0000	
4	8 -14	183	239	
196	-0.58	3.24	1.0000	
2	8 -14	921	260	
796	0.86	1.71	1.0000	
2	8 -14	706	296	
597	0.86	1.71	1.0000	
0	8 -14	472	285	
643	-0.37	3.74	1.0000	
0	8 -14	14	223	
46	-0.37	3.74	1.0000	
-2	8 -14	608	222	
538	1.80	0.57	1.0000	
-2	8 -14	1440	428	
1333	1.80	0.57	1.0000	
-4	8 -14	338	231	
313	-1.02	0.54	1.0000	
-4	8 -14	1717	462	
1661	-1.02	0.54	1.0000	
-6	8 -14	1799	486	

2126 3.06 -1.46 1.0000
-6 8 -14 401 240
561 3.06 -1.46 1.0000
7 7 -14 229 315
450 -4.72 -2.79 1.0000
5 7 -14 3 289
-136 0.98 -1.25 1.0000
5 7 -14 3 230
-68 0.98 -1.25 1.0000
3 7 -14 428 244
686 -2.47 -4.59 1.0000
3 7 -14 663 320
978 -2.47 -4.59 1.0000
1 7 -14 583 241
688 1.49 -2.50 1.0000
1 7 -14 865 297
993 1.49 -2.50 1.0000
-1 7 -14 397 275
336 -1.96 -1.96 1.0000
-1 7 -14 700 228
620 -1.96 -1.96 1.0000
-3 7 -14 610 294
789 -2.91 -0.47 1.0000
-3 7 -14 152 215
247 -2.91 -0.47 1.0000
-7 7 -14 2369 499
3011 -4.77 2.82 1.0000
8 6 -14 1 325
14 3.04 1.42 1.0000
6 6 -14 49 307
81 -0.80 2.55 1.0000
4 6 -14 335 241
417 1.77 1.46 1.0000
4 6 -14 235 301
304 1.77 1.46 1.0000
2 6 -14 25 204
-359 -0.36 4.34 1.0000
2 6 -14 183 279
86 -0.36 4.34 1.0000
0 6 -14 255 278
270 -0.32 1.49 1.0000
0 6 -14 3 197
-154 -0.32 1.49 1.0000
-2 6 -14 245 207

190 1.50 1.73 1.0000
-2 6 -14 336 271
271 1.50 1.73 1.0000
-4 6 -14 404 220
470 2.18 -0.72 1.0000
-4 6 -14 656 299
741 2.18 -0.72 1.0000
-6 6 -14 171 214
231 -0.80 -2.57 1.0000
-6 6 -14 1415 436
1589 -0.80 -2.57 1.0000
-8 6 -14 3113 490
3039 0.68 -2.03 1.0000
9 5 -14 31 317
-354 -4.78 0.20 1.0000
7 5 -14 5 315
-38 1.24 -1.47 1.0000
5 5 -14 142 312
30 -4.86 -3.00 1.0000
3 5 -14 356 224
363 0.87 -2.46 1.0000
3 5 -14 603 322
615 0.87 -2.46 1.0000
1 5 -14 274 219
383 -1.02 -3.18 1.0000
1 5 -14 87 288
150 -1.02 -3.18 1.0000
-1 5 -14 286 286
256 0.48 -2.06 1.0000
-1 5 -14 6 196
-407 0.48 -2.06 1.0000
-3 5 -14 251 205
216 -0.52 1.06 1.0000
-3 5 -14 448 272
401 -0.52 1.06 1.0000
-5 5 -14 1711 334
1222 -4.84 3.08 1.0000
-7 5 -14 2696 484
2679 1.02 1.29 1.0000
-9 5 -14 3035 492
2849 -0.88 2.99 1.0000
10 4 -14 17 343
-317 3.61 -0.16 1.0000
8 4 -14 2 338

-335 -0.92 -0.58 1.0000
6 4 -14 7 310
-40 2.18 0.71 1.0000
4 4 -14 4 296
-449 0.88 1.49 1.0000
2 4 -14 5 305
-65 -0.21 1.98 1.0000
2 4 -14 474 213
555 -0.21 1.98 1.0000
0 4 -14 718 228
859 -0.07 2.18 1.0000
0 4 -14 757 314
901 -0.07 2.18 1.0000
-2 4 -14 189 213
215 -0.95 -0.36 1.0000
-2 4 -14 1 280
-124 -0.95 -0.36 1.0000
-4 4 -14 158 198
212 0.89 -1.52 1.0000
-4 4 -14 249 276
316 0.89 -1.52 1.0000
-6 4 -14 2120 469
2316 1.71 -1.47 1.0000
9 3 -14 1 321
-235 -0.13 0.95 1.0000
7 3 -14 11 306
-360 -2.85 0.56 1.0000
3 3 -14 2 311
-483 -0.74 -0.90 1.0000
1 3 -14 5 291
-390 0.98 -1.75 1.0000
1 3 -14 5 190
-86 0.98 -1.75 1.0000
-1 3 -14 195 209
191 0.93 -0.03 1.0000
-1 3 -14 529 318
523 0.93 -0.03 1.0000
-3 3 -14 184 286
185 -0.71 0.99 1.0000
-3 3 -14 2 186
-100 -0.71 0.99 1.0000
-5 3 -14 437 214
448 0.88 2.48 1.0000
-5 3 -14 631 291

646 0.88 2.48 1.0000
-7 3 -14 1787 449
2296 -2.42 4.67 1.0000
10 2 -14 2 329
-253 -0.61 -0.93 1.0000
8 2 -14 4 313
-347 1.72 -0.51 1.0000
6 2 -14 7 287
-466 1.53 -1.78 1.0000
4 2 -14 1 295
-363 -0.91 0.36 1.0000
2 2 -14 4 303
-358 -1.56 -0.65 1.0000
0 2 -14 582 213
606 0.79 0.35 1.0000
0 2 -14 161 319
173 0.79 0.35 1.0000
-2 2 -14 27 307
33 -1.54 0.71 1.0000
-2 2 -14 63 198
73 -1.54 0.71 1.0000
-4 2 -14 235 290
290 -0.27 -1.97 1.0000
-4 2 -14 486 197
564 -0.27 -1.97 1.0000
-6 2 -14 25 193
-4 -0.29 -4.35 1.0000
-6 2 -14 521 292
355 -0.29 -4.35 1.0000
-8 2 -14 1503 313
1342 0.86 -1.73 1.0000
-8 2 -14 946 240
819 0.86 -1.73 1.0000
9 1 -14 1 303
-412 0.88 0.00 1.0000
5 1 -14 6 292
-310 0.43 2.16 1.0000
3 1 -14 1 307
-104 1.00 0.01 1.0000
-1 1 -14 7 197
-23 2.04 -0.87 1.0000
-1 1 -14 7 306
-132 2.04 -0.87 1.0000
-3 1 -14 5 288

-363	1.01	1.76	1.0000	
-3	1	-14	5	179
-56	1.01	1.76	1.0000	
-5	1	-14	15	188
-70	-0.97	3.24	1.0000	
-5	1	-14	5	297
7	-0.97	3.24	1.0000	
-7	1	-14	397	307
485	1.43	2.55	1.0000	
-7	1	-14	367	220
452	1.43	2.55	1.0000	
-9	1	-14	2464	452
2988	-0.55	4.55	1.0000	
8	0	-14	19	304
-388	-0.38	-3.74	1.0000	
6	0	-14	77	311
84	-0.29	-1.42	1.0000	
4	0	-14	432	320
542	-0.07	-2.16	1.0000	
2	0	-14	244	316
257	0.82	-0.29	1.0000	
0	0	-14	40	299
-241	-5.47	0.02	1.0000	
-2	0	-14	561	209
582	0.77	-0.31	1.0000	
-2	0	-14	92	306
100	0.77	-0.31	1.0000	
-4	0	-14	692	336
827	-0.01	-2.12	1.0000	
-4	0	-14	893	231
1045	-0.01	-2.12	1.0000	
-6	0	-14	3	288
-51	-0.30	-1.47	1.0000	
-6	0	-14	3	182
-8	-0.30	-1.47	1.0000	
-8	0	-14	69	206
139	-0.34	-3.72	1.0000	
-8	0	-14	210	299
326	-0.34	-3.72	1.0000	
9	-1	-14	27	308
-83	-0.55	4.44	1.0000	
7	-1	-14	69	312
105	1.52	2.63	1.0000	
5	-1	-14	14	289

```
-10    -0.91     3.10  1.0000
    1   -1 -14         7       290
-107     2.04    -0.92  1.0000
   -1   -1 -14        60       185
29     2.06     0.94  1.0000
   -1   -1 -14         7       285
-420     2.06     0.94  1.0000
   -3   -1 -14       656       208
648     0.87     0.06  1.0000
   -3   -1 -14       119       296
115     0.87     0.06  1.0000
   -5   -1 -14         6       184
-126     0.43     2.07  1.0000
   -5   -1 -14         6       297
-535     0.43     2.07  1.0000
   -7   -1 -14       151       199
110    -1.93     1.99  1.0000
   -7   -1 -14        10       293
-288    -1.93     1.99  1.0000
   -9   -1 -14       330       224
342     0.74    -0.03  1.0000
   -9   -1 -14       401       292
414     0.74    -0.03  1.0000
   10   -2 -14        11       272
-312    -0.72    -2.79  1.0000
    8   -2 -14       158       288
109     0.87    -1.69  1.0000
    6   -2 -14        25       274
-253    -0.32    -4.33  1.0000
    4   -2 -14         5       274
-8    -0.16    -1.98  1.0000
    2   -2 -14         4       279
-149    -1.51     0.68  1.0000
    0   -2 -14       175       296
188     0.79     0.34  1.0000
   -2   -2 -14        61       187
71    -1.48    -0.69  1.0000
   -4   -2 -14         1       289
-449    -0.88     0.48  1.0000
   -4   -2 -14       117       203
135    -0.88     0.48  1.0000
   -6   -2 -14         7       191
-17     1.57    -1.74  1.0000
   -6   -2 -14         7       287
```

-508	1.57	-1.74	1.0000	
-8	-2	-14	375	214
320	1.84	-0.62	1.0000	
-8	-2	-14	5	304
-483	1.84	-0.62	1.0000	
-10	-2	-14	240	297
276	-0.73	-0.91	1.0000	
-10	-2	-14	410	222
457	-0.73	-0.91	1.0000	
9	-3	-14	3	284
-176	1.28	0.93	1.0000	
7	-3	-14	69	285
184	-2.37	4.51	1.0000	
5	-3	-14	72	285
70	0.88	2.56	1.0000	
3	-3	-14	2	261
-315	-0.72	0.90	1.0000	
1	-3	-14	189	284
186	0.96	0.01	1.0000	
-1	-3	-14	5	277
-393	0.93	-1.73	1.0000	
-3	-3	-14	55	187
55	-0.69	-0.97	1.0000	
-3	-3	-14	2	291
-362	-0.69	-0.97	1.0000	
-5	-3	-14	23	187
13	-0.54	-1.04	1.0000	
-7	-3	-14	220	196
333	-2.95	0.51	1.0000	
-9	-3	-14	1	299
-519	-0.07	1.01	1.0000	
-9	-3	-14	390	214
346	-0.07	1.01	1.0000	
10	-4	-14	9	282
-77	-1.90	-1.67	1.0000	
8	-4	-14	32	283
29	-0.52	-3.18	1.0000	
6	-4	-14	143	273
198	1.79	-1.51	1.0000	
4	-4	-14	4	266
-89	0.87	-1.46	1.0000	
2	-4	-14	101	278
117	-0.86	-0.38	1.0000	
0	-4	-14	408	297

514	-0.01	2.15	1.0000	
-2	-4	-14	424	215
502	-0.24	2.04	1.0000	
-2	-4	-14	6	269
-268	-0.24	2.04	1.0000	
-4	-4	-14	4	286
-387	0.89	1.51	1.0000	
-4	-4	-14	97	205
140	0.89	1.51	1.0000	
-6	-4	-14	146	207
188	2.23	0.79	1.0000	
-8	-4	-14	2	191
-93	-1.03	-0.51	1.0000	
-10	-4	-14	157	219
268	3.65	-0.21	1.0000	
-10	-4	-14	18	301
-181	3.65	-0.21	1.0000	
9	-5	-14	12	263
-378	-0.81	2.89	1.0000	
7	-5	-14	4	274
-156	0.98	1.30	1.0000	
5	-5	-14	42	269
-23	-4.77	3.00	1.0000	
3	-5	-14	2	262
-193	-0.50	1.05	1.0000	
1	-5	-14	6	257
-359	0.34	-2.14	1.0000	
-1	-5	-14	14	271
-103	-0.90	-3.14	1.0000	
-3	-5	-14	390	201
397	0.86	-2.53	1.0000	
-3	-5	-14	9	279
-272	0.86	-2.53	1.0000	
-5	-5	-14	777	209
464	-4.82	-3.04	1.0000	
-5	-5	-14	43	299
-192	-4.82	-3.04	1.0000	
-7	-5	-14	348	227
283	1.32	-1.54	1.0000	
-9	-5	-14	150	205
120	-4.77	0.11	1.0000	
8	-6	-14	242	286
217	0.68	-1.95	1.0000	
6	-6	-14	9	260

-245 -0.78 -2.52 1.0000
4 -6 -14 146 284
186 2.18 -0.76 1.0000
2 -6 -14 115 270
76 1.57 1.78 1.0000
0 -6 -14 3 265
-66 -0.31 1.45 1.0000
-2 -6 -14 26 264
-326 -0.27 4.38 1.0000
-4 -6 -14 331 215
410 1.72 1.53 1.0000
-4 -6 -14 229 297
295 1.72 1.53 1.0000
-6 -6 -14 9 292
-527 -0.80 2.54 1.0000
-6 -6 -14 76 205
116 -0.80 2.54 1.0000
-8 -6 -14 290 216
432 3.10 1.51 1.0000
7 -7 -14 141 278
320 -4.66 2.81 1.0000
5 -7 -14 5 271
-62 1.24 1.52 1.0000
3 -7 -14 11 262
-27 -2.85 -0.58 1.0000
1 -7 -14 9 259
-117 -1.88 -1.84 1.0000
-1 -7 -14 76 283
115 1.44 -2.63 1.0000
-3 -7 -14 304 222
524 -2.42 -4.58 1.0000
-3 -7 -14 8 281
55 -2.42 -4.58 1.0000
-5 -7 -14 4 205
-5 0.98 -1.31 1.0000
-7 -7 -14 169 225
367 -4.76 -2.84 1.0000
6 -8 -14 28 284
78 3.08 -1.45 1.0000
4 -8 -14 107 264
92 -0.94 0.55 1.0000
2 -8 -14 181 272
144 1.73 0.53 1.0000
0 -8 -14 18 261

-165 -0.42 3.69 1.0000
-2 -8 -14 501 281
411 0.82 1.72 1.0000
-4 -8 -14 212 216
224 -0.50 3.23 1.0000
-4 -8 -14 14 276
-312 -0.50 3.23 1.0000
-6 -8 -14 6 293
-138 0.73 2.02 1.0000
-6 -8 -14 492 244
455 0.73 2.02 1.0000
5 -9 -14 29 261
-141 -4.70 -0.17 1.0000
3 -9 -14 1 277
-121 -0.21 -0.92 1.0000
1 -9 -14 98 265
104 0.89 -0.01 1.0000
-1 -9 -14 203 287
365 -0.55 -4.45 1.0000
-3 -9 -14 3 280
-103 1.21 -0.94 1.0000
-5 -9 -14 13 232
-32 -0.91 -2.95 1.0000
-5 -9 -14 13 284
-205 -0.91 -2.95 1.0000
4 -10 -14 4 283
27 3.63 0.17 1.0000
2 -10 -14 151 290
178 -0.71 0.87 1.0000
0 -10 -14 5 273
-86 1.09 1.64 1.0000
-2 -10 -14 11 268
-195 -0.74 2.78 1.0000
-4 -10 -14 9 290
-65 -1.90 1.74 1.0000
-4 -10 -14 9 207
-46 -1.90 1.74 1.0000
0 5 -15 234 244
275 -0.96 0.61 1.0000
0 5 -15 318 315
365 -0.96 0.61 1.0000
3 4 -15 582 261
676 1.29 1.09 1.0000
3 4 -15 391 359

469 1.29 1.09 1.0000
1 4 -15 8 315
-18 1.39 -2.00 1.0000
1 4 -15 8 228
-100 1.39 -2.00 1.0000
-1 4 -15 3 215
-251 1.43 0.01 1.0000
-1 4 -15 3 308
-14 1.43 0.01 1.0000
-3 4 -15 567 316
614 0.94 -0.97 1.0000
-3 4 -15 417 228
457 0.94 -0.97 1.0000
4 3 -15 3 342
-287 1.00 1.05 1.0000
2 3 -15 68 347
49 -1.04 -0.43 1.0000
2 3 -15 235 222
199 -1.04 -0.43 1.0000
0 3 -15 32 213
28 0.51 0.45 1.0000
0 3 -15 1 325
-212 0.51 0.45 1.0000
-2 3 -15 56 217
49 -0.46 1.87 1.0000
-2 3 -15 85 308
77 -0.46 1.87 1.0000
-4 3 -15 1060 251
1188 1.32 -1.09 1.0000
-4 3 -15 780 342
891 1.32 -1.09 1.0000
3 2 -15 5 337
-286 -0.45 -1.80 1.0000
1 2 -15 6 220
-51 1.94 0.68 1.0000
1 2 -15 6 317
-265 1.94 0.68 1.0000
-1 2 -15 172 226
162 0.80 -0.97 1.0000
-1 2 -15 2 317
-305 0.80 -0.97 1.0000
-3 2 -15 92 311
70 -1.05 0.46 1.0000
-3 2 -15 459 214

408 -1.05 0.46 1.0000
2 1 -15 2 327
-323 0.82 1.05 1.0000
0 1 -15 186 348
190 0.01 -0.63 1.0000
0 1 -15 920 248
930 0.01 -0.63 1.0000
-2 1 -15 35 210
14 1.94 -0.65 1.0000
-2 1 -15 6 314
-533 1.94 -0.65 1.0000
-4 1 -15 85 322
101 1.39 2.03 1.0000
-4 1 -15 460 218
505 1.39 2.03 1.0000
5 0 -15 2 324
-470 -0.94 -0.50 1.0000
1 0 -15 86 329
89 -0.02 0.69 1.0000
-1 0 -15 497 236
504 -0.01 0.66 1.0000
-1 0 -15 245 347
250 -0.01 0.66 1.0000
-3 0 -15 1 315
-452 0.51 -0.46 1.0000
-3 0 -15 90 219
83 0.51 -0.46 1.0000
-5 0 -15 2 321
0 -0.94 -0.51 1.0000
-5 0 -15 278 231
320 -0.94 -0.51 1.0000
4 -1 -15 8 321
-357 1.42 2.03 1.0000
0 -1 -15 1 336
-102 0.01 -0.63 1.0000
0 -1 -15 897 236
907 0.01 -0.63 1.0000
-2 -1 -15 3 318
-565 0.85 1.09 1.0000
-2 -1 -15 3 213
-112 0.85 1.09 1.0000
-4 -1 -15 3 208
-78 1.38 0.04 1.0000
3 -2 -15 2 328

```
-336   -1.09    0.54  1.0000
  -1  -2 -15      18      199
4     1.90    0.66  1.0000
  -3  -2 -15      34      206
27   -0.42   -1.81  1.0000
   4  -3 -15     138      328
187     1.34   -1.11  1.0000
   2  -3 -15       5      321
-388   -0.40    1.82  1.0000
   0  -3 -15       1      312
-398    0.56    0.55  1.0000
  -2  -3 -15     177      210
145   -1.07   -0.49  1.0000
  -4  -3 -15     279      244
312     0.93    1.02  1.0000
  -1  -4 -15       8      316
-338    1.41   -1.97  1.0000
  -3  -4 -15     258      361
326     1.37    1.16  1.0000
  -3  -4 -15     955      254
1083    1.37    1.16  1.0000
   0  -5 -15       1      312
-257   -0.93    0.46  1.0000
   0   0   0       5        0
0   -0.38   -1.85  1.0000
;
_shelx_hkl_checksum   70824
```
