# Supplementary material for: Solvent-controlled self-assembly of tetrapodal [4 + 4] phosphate organic molecular cage
Source: Sci Rep. 2020 Mar 13;10:4712. doi: 10.1038/s41598-020-61813-6 (PMC7070053; doi:10.1038/s41598-020-61813-6)
Supplement: Supplementary file 1 — Supplementary information [file 41598_2020_61813_MOESM1_ESM.doc]

## Coordination Chemistry Institute Tel: +86 25 83686526

**Nanjing 210093	Fax: +86 25 83314502**

**P. R. China	Email: whuang@nju.edu.cn**

**_____________________________________________________________________**

February, 16, 2020

Dear editor: Prof. Enrico Dalcanale,

Could you please find our revised version of the manuscript entitled “Solvent-controlled self-assembly of tetrapodal [4+4] phosphate organic molecular cage” for your consideration for publication as an article in *Scientific Reports*? First of all, we would like to thank the reviewers for their comments and suggestions to improve our manuscript. The revised version of the manuscript has been set up according to their comments and suggestions as follows:

Reviewer Comments:

Page 2, fourth line from the bottom: precipitate instead of participate.

Figure 1. Figure 1d is not mentioned in the text. Please add.

Response: We apologize for our carelessness here. In the manuscript “participate” has been replaced by “precipitate” and Figure 1d has been also added in the text.

We do hope that our revised version of the manuscript will be suitable for publication in Scientific Reports. I also would like to take this opportunity to thank you for your time and consideration of our manuscript.

With best regards,

Wei Huang

===================================
Prof. Dr. Wei Huang
State Key Laboratory of Coordination Chemistry
Nanjing National Laboratory of Microstructures
Coordination Chemistry Institute
School of Chemistry and Chemical Engineering
Nanjing University
210093, P.R.China
[whuang@nju.edu.cn](mailto:whuang@nju.edu.cn)
